# Orthology-Based Estimate of the Contribution of Horizontal Gene Transfer from Distantly Related Bacteria to the Intraspecific Diversity and Differentiation of *Xylella fastidiosa*

**DOI:** 10.3390/pathogens10010046

**Published:** 2021-01-07

**Authors:** Giuseppe Firrao, Marco Scortichini, Laura Pagliari

**Affiliations:** 1 Dipartimento di Scienze Agroalimentari, Ambientali e Animali, University of Udine, Udine, Italy; 2 Istituto Nazionale Biostrutture e Biosistemi, Rome, Italy; 3 Consiglio per la Ricerca in Agricoltura e l’Analisi dell’Economia Agraria-Centro di ricerca per l’Olivicoltura, Frutticoltura e Agrumicoltura, Via di Fioranello, 52, 00134 Rome, Italy; marco.scortichini@crea.gov.it

**Keywords:** hierarchical orthologous group, pangenome, HGT, *Xanthomonadales*, *Xanthomonas*, *vasicola*, *albilineans*

## Abstract

*Xylella fastidiosa* is a xylem-limited bacterium phylogenetically related to the xanthomonads, with an unusually large and diversified range of plant hosts. To ascertain the origin of its peculiarities, its pan-genome was scanned to identify the genes that are not coherent with its phylogenetic position within the order *Xanthomonadales*. The results of the analysis revealed that a large fraction of the genes of the *Xylella* pan-genome have no ortholog or close paralog in the order *Xanthomonadales*. For a significant part of the genes, the closest homologue was found in bacteria belonging to distantly related taxonomic groups, most frequently in the *Betaproteobacteria*. Other species, such as *Xanthomonas vasicola* and *Xanthomonas albilineans* which were investigated for comparison, did not show a similar genetic contribution from distant branches of the prokaryotic tree of life. This finding indicates that the process of acquisition of DNA from the environment is still a relevant component of *Xylella fastidiosa* evolution. Although the ability of *Xylella fastidiosa* strains to recombine among themselves is well known, the results of the pan-genome analyses stressed the additional relevance of environmental DNA in shaping their genomes, with potential consequences on their phytopathological features.

## 1. Introduction

*Xylella fastidiosa* is a Gram-stain-negative, xylem-limited, vector-borne, nutritionally fastidious phytopathogen that can colonize many wild and cultivated plant species. The host range of *Xylella fastidiosa* is unusually large among plant pathogenic bacteria, and comprises 563 plant species grouped into 82 botanical families, including many of socio-economic interest (i.e., grapevine, citrus, coffee, and olive trees) [1]. Nevertheless, only a limited number of infected plant species shows the typical wilting of leaves, twigs and branches and the subsequent plant death, caused by the pathogen-induced blockage of xylem vessels [2]. The currently available data suggest that *Xylella fastidiosa* is associated with a large number of plant species as a commensalist, but only particular clades -or even genotypes- are responsible for a small number of plant diseases [3,4,5].

According to the taxonomy based on ribosomal RNA sequence analysis, *Xylella fastidiosa* is a member of the *Xanthomonadaceae* family [6]. Genome analyses have confirmed that the status of *Xylella fastidiosa* as a member of the family is phylogenetically well supported [7], even if its physiological, cultural, morphological and phytopathological features greatly differ from *Xanthomonas* spp. Three subspecies are recognized within the *Xylella fastidiosa* species, namely *fastidiosa*, *multiplex* and *pauca*, while a closely related species is *Xylella taiwanensis* which causes pear leaf scorch in Taiwan [8,9,10,11].

The study of the phylogenetic relationships among *Xylella fastidiosa* isolates and the clade organization in relation to both host plant and geographical location has been promoted by MultiLocus Sequence Type analysis (MLST) [12]. The same method also revealed recombination between *Xylella fastidiosa* strains [13] and genetic introgression of different subspecies within the native *Xylella fastidiosa* strains of Central America; substantial recombination was observed between some *Xylella fastidiosa* subsp. *multiplex* and subsp. *fastidiosa* strains of the United States, likely associated with host shifts of the strains [14]. Relevant gene flow between *Xylella fastidiosa* subspecies has also been detected for strains of subsp. *pauca* and *multiplex* in Brazil [15]. Despite the occurrence of recombination events between strains, *Xylella fastidiosa* shows a low ratio of recombination to point mutation on a per housekeeping gene basis when compared to other bacterial species [12]. Genome-wide approaches also revealed recombination events among strains, so that ancestral and more recent recombination was detected for all subspecies, with some strains showing a high recombination rate [16,17]⁠.

Most studies published so far have focused on intraspecific recombination events, therefore little is known about the proportion of genes possibly acquired by *Xylella fastidiosa* from related or taxonomically distant species. Denancé et al. [11], by assessing the *Xylella fastidiosa* strain k-mers of the genomes, confirmed the variability of the species and found (in one strain isolated from *Hibiscus fragilis*) unique regions both in the chromosome and in the plasmid with high homology to genomic regions of other bacterial genera, such as *Comamaonas*, *Cupriavidus*, *Pseudomonas*, *Klebsiella*, *Bordetella*, *Burkholderia* as well as *Xanthomonas euvesicatoria*.

To investigate whether parts of the *Xylella fastidiosa* genome could have originated by horizontal transfers, we applied a genomic approach that allows the comparison of either related or distantly related bacterial strains. To accomplish the task, we built up the pan-genome of *Xylella fastidiosa* from a large sample of genome drafts, and scanned it asserting orthology for each gene. As comparison references, we applied the same approach to other two species of the *Xanthomonadaceae*, i.e., *Xanthomonas vasicola* and *Xanthomonas albilineans*, which are a typical and an atypical member species of the genus *Xanthomonas*, respectively [18]. Similarly to *Xylella fastidiosa*, *Xanthomonas vasicola* colonizes many different host plants such as corn, sugarcane, sorghum, banana, ensete, areca nut and *Tripsacum laxum* [19], thus showing strong adaptive potential. On the other hand, *Xylella fastidiosa* shares with *Xanthomonas albilineans* some relevant features: both actively colonize the plant xylem, they do not have *hrp* genes encoding for the type III secretion system, and both experienced a reductive genome evolution during their descent from the common ancestor, presumably due to adaptation to the nutritionally poor xylem niche [20]. We show here that, from a genomic point of view, *Xylella fastidiosa*, differently from the two other *Xanthomodales*, has a significant portion of its genome that was acquired by horizontal genetic transfer (HGT) from distantly related bacteria.

## 2. Results

### 2.1. Orthology Assertion

Each putative protein-encoding gene of the pan-genome of *Xylella fastidiosa* and, for comparison, of the pan-genomes of *Xanthomonas vasicola* and *Xanthomonas albilineans*, was investigated to ascertain whether or not it shares a common ancestry with genes of other members of the order *Xanthomonadales*, a taxon that includes all three named species.

To gain an insight into the ancestry of the genes, we exploited the OMA (Orthologous Matrix), which distinguishes itself through high-quality orthology inferences, the broad coverage of bacteria, and the availability of a programmatic access [21,22]. We systematically searched the pan-genomes for genes missing orthologs of close paralogs in the class *Xanthomonadales.*

The procedure outlined in Figure S1 integrates the results of sequence similarity searches, orthology inferences, and global alignments, to select, from each pan-genome, the genes whose origin cannot be traced back to the *Xanthomonadales*. Genes that were most similar to genes carried by phylogenetically distant bacteria were not included in the selection, when a putative hierarchical ortholog at the *Xanthomonadales* level was detected. This conservative approach implies discharging a priori hypotheses of independent acquisition or loss and gain events, but provided a non-subjective, well-defined frame for comparison.

The results of the assignments are hereafter presented in detail, first restricted to phylogenetically conserved subsets, and then extended to the entire pan-genome.

### 2.2. Analysis of the Core Genome

The first set investigated was restricted to the so-called BUSCO (Benchmarking Universal Single-Copy Orthologs) genes [23]. BUSCO sets for the major bacterial lineages, available through OrthoDB (www.orthodb.org), are selected from orthologous groups with genes present as single-copy orthologs in at least 90% of the species, and are hence regarded as having strict evolutionary coherence. Within the BUSCO dataset compiled for the *Gammaproteobacteria* there are 452 genes. In the genomes of *Xanthomonas vasicola* and *Xanthomonas albilineans*, seven and eight of these genes, respectively, were missing from all genomes. Considering the genomes of *Xylella fastidiosa*, as many as 15 BUSCO genes were missing, including RecX, an inhibitor of RecA (Table 1). All BUSCO genes of all three species belong to a hierarchical orthologous group (HOG) at the level of *Xanthomonadales*.

A second set, named Core, is a subset of the pan-genome that includes the genes shared by all genomes of each species examined. The number of genes included reflects the average size and diversity of the genomes used for the construction of the pan-genome, and also the completeness and quality of the drafts used [24].

For *Xanthomonas vasicola*, which has an average genome size of about 4.9 Mbp containing 3888 genes (max: 4068, min: 3174), the core genome constructed using 65 genome drafts comprises 1828 orthologous groups (OGs) of protein encoding genes, (47% of the average genome), all having HOGs at the *Xanthomonadales* level and all having their best BLAST hit within the *Xanthomonadales* (Table 2, Figure S2).

The genome of *Xanthomonas albilineans* has an average size of 3.7 Mbp and contains 2919 genes (max: 3083, min: 2764); its core genome constructed from 16 genome drafts comprises 2386 OGs of protein-encoding genes (81.7% of the average genome). Among them, 2371 (99.4%) have HOGs at the *Xanthomonadales* level; the remaining 15 genes (corresponding to 0.6% of the total genes of the core genome) included several degrading enzymes, enzymes connected with chorysmate metabolism, a non-ribosomal peptite synthetase, and a methyl transferase (Table 2). According to our analysis, most of those genes have homologs in the genomes of organisms in the beta- and delta-subdivision of the Proteobacteria (Figure S3). Moreover, for 14 of the 2371 genes, the detected HOG is not the best BLAST hit in the database and may therefore be the results of gene loss and gain from taxonomically unrelated bacteria.

In *Xylella fastidiosa*, the average genome is 2.54 Mbp in size and contains, on average, 2176 genes (min: 1930, max: 2413), and the core genome comprises 1045 genes (55.6% of the average genome) (Table 2). Among them, there were seven genes (corresponding to 0.7% of the total genes of the core genome) for which no ortholog or close paralog could be found in the *Xanthomonadales*, including a membrane protein and a quinolinate synthase NadA. In addition, there were 15 detected HOGs for which the best BLAST hit in the database was not in the *Xanthomonadales* and may therefore be the results of gene loss and gain from taxonomically unrelated bacteria (Table 2, Figure S4).

### 2.3. Analysis of the Pan-Genome

Although the analysis of the core genome may provide clues of ancient HGTs that have been fixed within the genome, thus contributing to the definition of the ecological features of the species, the pan-genome analysis includes genes that were recorded in some (at least two) but not all genomes. Thus, it defines features that may affect the host range or the differences among subspecies or strains [24].

**Figure 1:**
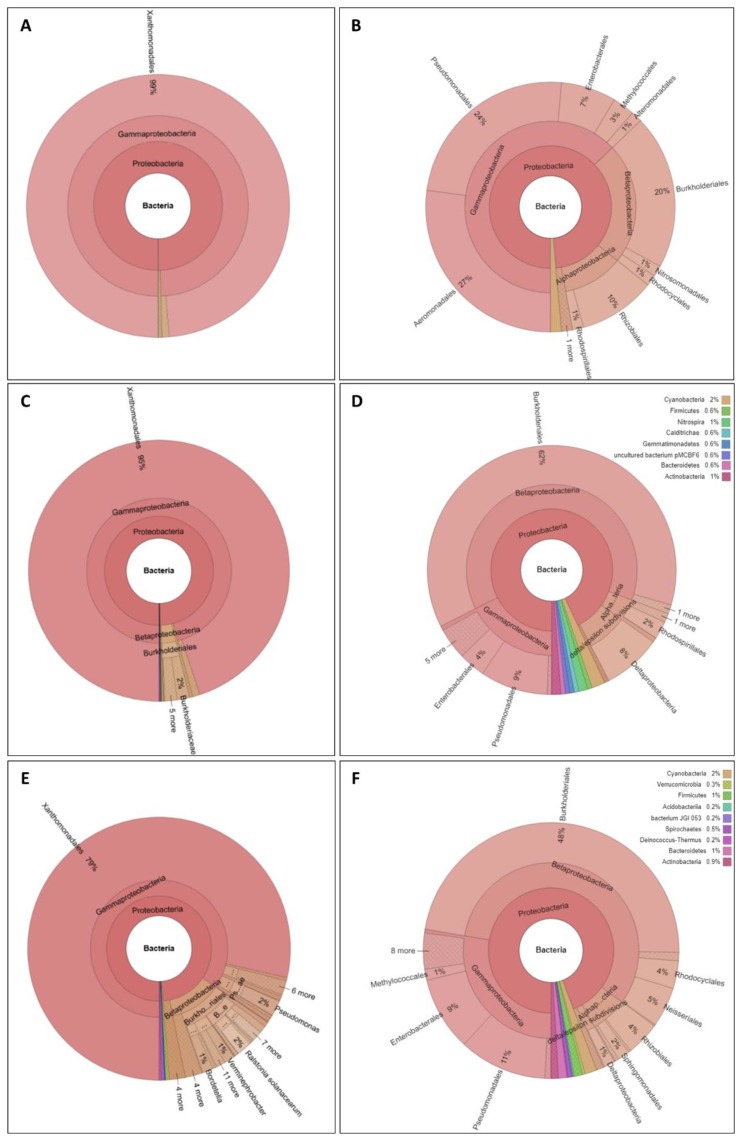
Taxonomic subdivision of the nearest ortholog assignment of all genes in the pan-genome (**A**,**C**,**E**) and of the subset of genes that do not have an ortholog in the *Xanthomonadales* (**B**,**D**,**F**) as detected in the pan-genomes of *Xanthomonas vasicola* (**A**,**B**), *Xanthomonas albilineans* (**C**,**D**) and *Xylella fastidiosa* (**E**,**F**). For better reading, the same graphics are available in interactive form as Supplementary Figures S2–S4.

The pan-genome of *Xanthomonas vasicola* comprises 5296 genes, 141 (2.6%) of which had no significant homology with other proteins in the database and no associated putative function. Among the remaining 5155 genes, there were 70 genes (1.4%) for which no HOG at the *Xanthomonadales* level was found (Figure 1); with the sole exception of a membrane protein, they encode proteins presumably associated with phages or plasmids (Figure S2).

The pan-genome of *Xanthomonas albilineans* comprises 3536 genes, 167 of which (4.7%) had no significant homology with other proteins in the database and no associated putative function. Among the remaining 3369 genes, 95% have HOGs *Xanthomonadales*, while the remaining 171 OGs included coding sequences that could be traced primarily to the *Betaproteobacteria,* and also to other classes (Figure 1 and Figure S3). Most of these proteins are annotated as hypothetical proteins, phage and plasmid related proteins, or DNA processing enzymes, reducing the number of genes with putative associated function and possible impact on adaptation to those already found as part of the core genome set and eight additional proteins, which included two components of membrane transport system, one adhesin, two enzymes with putative biosynthetic activities, and another non ribosomal peptide synthetase. Interestingly, the *Xanthomonas albilineans* genome includes genes encoding non-ribosomal peptide synthetases that are absent in other *Xanthomonas* spp. but present in a wide range of bacteria.

The pan-genome of *Xylella fastidiosa* comprises 3673 protein genes, among which as many as 616 (nearly one-fifth of the putative coding sequences of the pan-genome) had no significant homology with other proteins in the database, and no associated predicted function. Among the others (3049, i.e., 83% of the pan-genome), 75% have homologs in the order *Xanthomonadales,* while as many as 654 (17.8% of the pan-genome) could not be tracked to the class (Figure 1; Figure S4). Among them, 473 (12.8%) encode functions that could not be tracked to the *Gammaproteobacteria.* Although a significant fraction of the genes with no HOG in common to *Xanthomonadales* encodes phage/transposon-related proteins or putative proteins with no function determined, we identified a set of 313 genes with predicted function.

### 2.4. Genomic Profiling of Pathway Activity for Xylella fastidiosa

**Figure 2:**
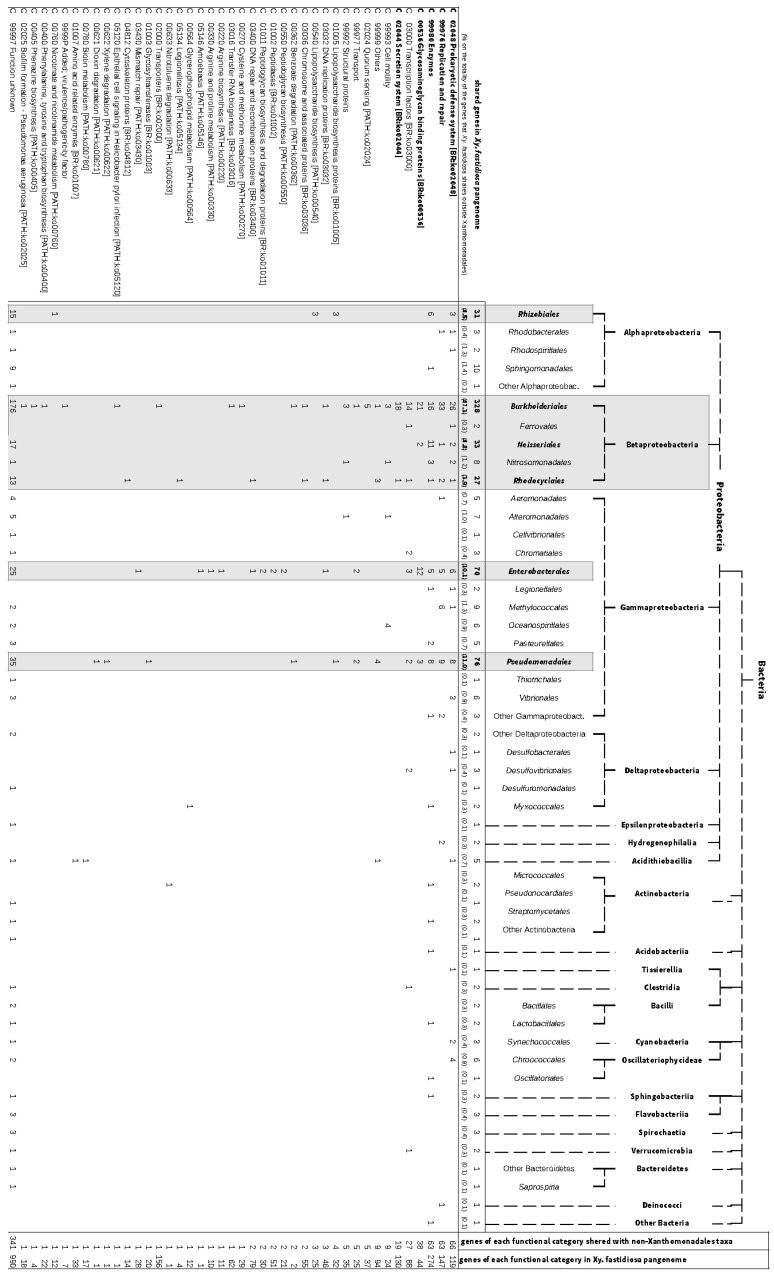
Assignment of genes of the pan-genome of *Xylella fastidiosa* without a hierarchical orthologous group (HOG) in the *Xanthamonadales* according to their KEGG pathways or GO terms and their taxonomically closest homologue. A simplified taxonomic tree of the taxa is shown at the top of the figure. The last column of the table reports the assignment of all genes of the *Xylella fastidiosa* pan-genome to the given KEGG pathway or GO term.

To obtain clues about the function of this gene set, we enriched the annotation with ko-terms using the KEGG facilities (KAAS) and classified the CDSs according to their function and their taxonomically closest relative. A summary of the KEGG annotation is shown in Figure 2, where the CDSs that were not assigned by the KAAS annotator were manually assigned to additional categories according to the results of BLAST searches.

According to the analysis, the largest contribution to the diversification of *Xylella fastidiosa* strains was given by the *Betaproteobacteria,* particularly from the orders *Burkholderiales* (47.3% of the gene set), *Neisseriales* (4.8%) and *Rhodocyclales* (3.9%) (Figure 2). Additionally, members of *Gammaproteobacteria* and the *Alphaproteobacteria* played a role in shaping the *Xylella fastidiosa* pan-genome (28 and 6.8%, respectively), in particular the orders *Enterobacterales* (10.1%), *Pseudomonadales* (11.0%) and *Rhizobiales* (4.5%) (Figure 2). The predominant input of *Burkholderiales* genes is recognizable in the five most populated functional categories, i.e., “Prokaryotic defense system”, “Replication and repair”, “Enzymes”, “Secretion system” and “Glycosaminoglycan binding proteins” (Figure 2). “Prokaryotic defense system” includes proteins of the type II toxin–antitoxin system (roughly 40% of the total proteins annotated within this category), among which 15 out of 25 (i.e., 60%) are from the *Burkholderiales*. Additionally, ATP-dependent helicases, a major group within the “Replication and repair” category (27%), constitutes proteins probably derived from HGT with members of *Burkholderiales* (Table S2). “Enzymes” collects the long list of various genes encoding biosynthetic or degrading enzymes that for their limited functional characterization were reported in this general group. They include hydroxylases, acyltransferases, deaminases, aminomutase, decarboxylase, superoxide dismutase, lipase, tautomerase, quinolinate synthase, anthranilate synthase, alkene reductase, ketoreductases, and peptidase of various families, as summarized in Table S3.

Roughly one-fifth of them had their closest orthologous groups within the *Burkholderiales* order (Table S2). Additionally, the genes for hemagglutinins, which cover more than the 50% of the “Glycosaminoglycan binding proteins” category, are similar to the homologous genes of members of the *Burkholderiales* (Table S2). Interestingly, all of the approximately 20 genes in the “Secretion systems” had their closest orthologs within the *Burkholderiales* and are associated with conjugal transfer (Table S2). Additionally, “Prokaryotic defense systems” and “Replication and repair” include many genes associated with plasmids and transposons (Table S2). Furthermore, an analysis of the sequences deposited as plasmids in the databases showed that 431 out of the 654 (66%) OGs that we identified as missing an ortholog in the *Xanthamonadales* included at least one member that has been found on a plasmid (Table S2). Beside *Burkholderiales*, other bacterial orders contributed to *Xylella fastidiosa* diversity. For example, several genes encoding proteins of the toxin–antitoxin (TA) system, which are listed under the descriptor heading “Prokaryotic defense systems”, had their closest relative distributed over a wide taxonomic range (i.e., *Xanthomonadales, Rhizobiales, Bulkholderiales*, *Enterobacterales*, *Pseudomonadales, Nitrosomonadales, Methylococcales,* and *Desulfobacterales*).

### 2.5. Evidence of Recent HGT in Xylella fastidiosa Genomes

The 6697 nts region of the genome of *Xylella fastidiosa* subsp. *fastidiosa* 9a5c, from positions 1,644,305 to 1,651,001 (NCBI reference sequence: NC_002488.3), is almost identical (90% identity at the DNA level) to the 6758 nts region, from position 6,187,137 to 6192834, of the distantly related *Pseudomonas* sp. CCA1 (NCBI reference sequence: NZ_BDGS01000001.1). The region includes three genes encoding aldo/keto reductases, and genes encoding a 4-oxalocrotonate tautomerase, a glucose 1-dehydrogenase, an NADP–alcohol dehydrogenase, and an MFS transporter, in the same strictly conserved order. The region is common to the *Xylella fastidiosa* subsp. *pauca* strains isolated from plum, citrus and coffee in South America, and not to *Xylella fastidiosa* subsp. *fastidiosa* from North America nor to other subspecies.

**Figure 3:**
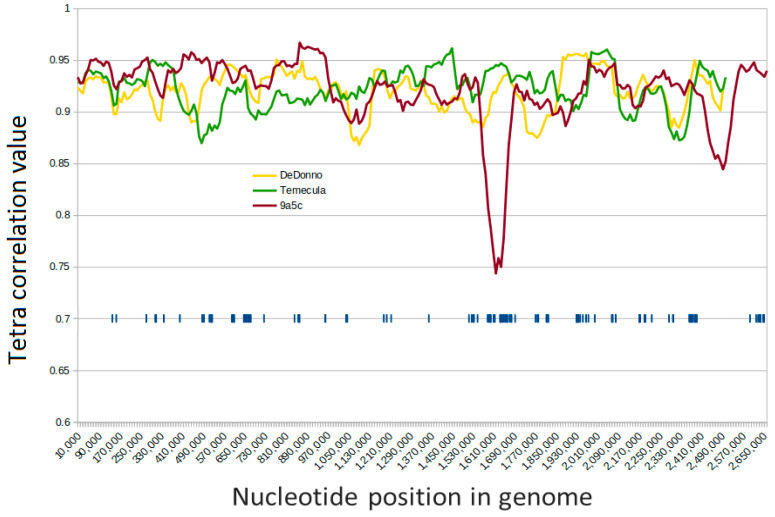
Sliding window graph of the tetra correlation values between a 100 k window and the entire chromosome. The DNA sequence of the chromosomes of three *X. fastidiosa* strains (*Xylella fastidiosa* subsp. *fastidiosa* strain Temecula, green line; *Xylella fastidiosa* subsp. *pauca* strain DeDonno, yellow line; *Xylella fastidiosa* subsp. *pauca* strain 9a5c, red line) were scanned and for each position (*x*-axis) the tetra correlation value calculated between the 100,000 nucleotides following that position and the entire chromosome sequence was reported in the *y*-axis. The blue ticks mark the position on the chromosome of strain 9a5c of the ORFs for the proteins that were found to be missing an ortholog in the *Xanthomonodales* in this work.

In addition to the high similarity at the DNA level and the conservation of the gene order, further evidence that this region represents a recent introduction into the *Xylella fastidiosa* chromosome was gained by comparing the tetranucleotide patterns in the region and in the entire chromosome. The analysis was performed on 15 genomes (Figure S5 and Figure 3); Figure 3 shows a sliding window graph of the tetra correlation values between the 100,000 nts windows and the chromosome of three representative strains. Drops of the correlation values occurred between positions 1,610,000 and 1,670,000 and between positions 2,480,000 and 2,520,000 in the chromosome of *Xylella fastidiosa* subsp. *pauca* 9a5c. Divergence in the tetranucleotide patterns is regarded as a hallmark of recent horizontal gene transfer events [25]: DNA sequences that are introduced through recent horizontal transfer often bear distinct sequence characteristics and can be distinguished from ancestral DNA but, over time, these sequences ameliorate to reflect the DNA composition of the new genome [26].

## 3. Discussion

In this work, by applying an orthology grouping approach on different gene sets and different genomes, we showed that at least one-fifth of the genes of the pan-genome of *Xylella fasidiosa* originated in distant branches of the evolutionary tree and was presumably acquired by HGT. The pan-genome defines characteristics that may affect the host range or the differences among subspecies or strains [24], therefore several features that delineate the ecological and physiological peculiarity of *Xylella fastidiosa* may indeed rely on genetic material common to organisms that share its niche rather than its phylogeny. HGT has played an important role in bacterial evolution and in facilitating the origins of bacterial diversity, on which the concept of adaptive HGT has been built [27]. Further investigation of the time when the introduction of the genetic determinants of diversity impacted the evolutionary history of a pathogen may shed light on the key phases of its adaptation to the environment.

### 3.1. BUSCO Analysis Confirmed the Reduction of Xylella fastidiosa Genome

The BUSCO gene dataset compiled for the *Gammaproteobacteria* was compared with the genes borne by *Xanthomonas vasicola, Xanthomonas albilineans* and *Xylella fastidiosa* genomes, showing that these three species are missing allegedly ubiquitous genes, accordingly with the genome reduction that characterized their evolution [7,20]. The extensive genome reduction observed in some xanthomonads has been connected with their endophytic lifestyle and their typical commitment to a reduced host range [28]. It is therefore noticeable that *Xylella fastidiosa* was the species with the highest number of missing genes, despite being characterized by a broad host range. *Xylella fastidiosa* misses the regulatory protein RecX, a small protein inhibiting RecA [29]. RecA is involved in the induction of the SOS response [30], essential to increase bacterial tolerance to DNA damage associated with several factors including HGT [31]. Thus, for *Xylella fastidiosa* the absence of RecX may imply enhanced exchange of genetic material even in the absence of strong selective pressure.

### 3.2. Distantly Related Bacteria Contributed to the Shape of the Xylella fastidiosa Accessory Genome

For the genome comparison of the three species considered, we preliminary focused the analysis on the core genome, which is the pool of genes shared by all the strains of the same taxon and hence differs from the BUSCO set for the inclusion of genes that are not ubiquitous, but typical of that species. When compared to the BUSCO set, the core genome set highlights the genes that were exploited by the species to gain access to the ecological niche that allowed speciation. Instead, the third set, the pan-genome, highlights the strain-specific features that were exploited for strain differentiation, likely associated with adaptation to a new host or environment.

*Xanthomonas vasicola* was found to have a compact core genome (representing roughly half of the whole genome), as already pointed out by Studholme and coworkers [32], who associated the reductive evolution tendency of *Xanthomonas vasicola* core genome with the genomic gains arisen by recent genetic exchange with other bacteria. Rodriguez-R and coworkers [7] reported a level of genomic loss and gain that was almost twice as large as that exhibited by *Xanthomonas albilineans*, suggesting that the *Xanthomonas vasicola* genomes are very dynamic. Our study detected no gene of the core genome without an ortholog or close paralog within the *Xanthomanadales*, while about 100 genes (2%) in the pan-genome were the result of HGT from distant taxa. In summary, the dynamic of the large accessory genomes of *Xanthomonas vasicola* is more relevant in the diversification of strains and their virulence features rather than in the context of the definition of the primary ecological niche that leads to speciation.

Differently from *Xanthomonas vasicola,* both the core genome and pan-genome of *Xanthomonas albilineans* are characterized by the presence of genes with origin by HGT from distant taxa. Nevertheless, in the *Xanthomonas albilineans* pan-genome only a few of these genes may have an impact on adaptation and most of them were actually found within the core genome set.

Consistently with the narrow host range of *Xanthomonas albilineans,* limited to sugarcane and few other monocots in the *Poaceae* family [33], here the contribution of HGT from distant taxa has been more relevant in defining the characteristics of the species rather than in strain-to-strain diversification. Some of the genes inherited from distant taxa, such as serine hydrolase, different degrading enzymes, non-ribosomal peptite synthetases and the two methyl transferases, are regarded as virulence factors [34,35,36,37,38], suggesting the possibility that *Xanthomonas albilineans* exploited external genomic resources to cope with host defense in a phytopathological context that is not typical of the xanthomonads.

*Xylella fastidiosa* is yet another case. Similarly to *Xanthomonas albilineans,* a few genes of the core genome were identified as not originating within the *Xanthomonadales*. On the other hand, in the pan-genome the number of the genes presumably transferred horizontally from distant taxa is extremely large, suggesting the intense trading with environmental bacteria as a major determinant of strain diversity. Further evidence of recent introduction in the genomes comes from the detection of DNA stretches with very high sequence similarity to bacteria widely spread in the environment, although not typically associated to the xylem: only a very recent gene exchange may explain the extremely high similarity at the DNA level of the 6697 nts region that we detected in the genome of *Xylella fastidiosa* subsp. *pauca* 9a5c and related strains with the corresponding region in the genome of the distantly related *Pseudomonas* sp. CCA1, a lignin-degrading bacterium isolated from soil in Japan for which the name *Pseudomonas humi* has been proposed [39]. Connections between rhizosphere and phyllosphere bacterial populations have already been reported [40].

The genome plasticity of *Xylella fastidiosa* is well known, as it was for *Xanthomonas vasicola*; it was shown in ref 7 that a large gene flux characterizes strain diversity. We confirmed the large size of the accessory genomes, but we also showed that the gene reservoir accessible to *Xanthomonas vasicola* is primarily contained within the order *Xanthomonadales*. Conversely, *Xylella fastidiosa* showed a similar largeness of the accessory genome, despite its reduced genome size, but the reference reservoir for this species does not appear to be confined within the order boundaries, because it extensively draws genes from the bacterial divisions that are most abundant in the plant endosphere.

The pool of genes that has been gained from distantly related bacteria is part primarily of the accessory and only to a lesser extent of the core genome, therefore we deduce that those genes play a role in strain-to-strain differentiation even more markedly than in shaping the features needed for the access to the habitat that is typical of the whole species. In other words, the results presented here highlight the relevance of the genes gained from distantly related environmental bacteria in subspecies and strain differentiation, and in determining host specificity.

### 3.3. Xylella fastidiosa Gained Different Functions from Various Clades

*Xylella fastidiosa* genes with homology only to genes found in distantly related bacteria were classified, enriching the annotation with ko-terms using the KEGG facilities. The analysis showed that bacteria belonging to the order *Burkholderiales* contributed to almost one-tenth of the entire *Xylella fastidiosa* pan-genome, covering different functional categories. These data match with the fact that the genes of *Xylella fastidiosa* associated with the type IV secretion system were particularly abundant and were similar to their homologs in the *Burkholderiales*. Type IV secretion system genes mediate the transfer of DNA and proteins from cell to cell in various biological contexts, including conjugation [40]. Conceivably, the availability of a conjugation system in common with the *Betaproteobacteria* results from, but also promotes, the HGT with bacteria belonging to this large and diverse bacterial division.

Concerning the contributions to *Xylella fastidiosa* diversity given by other bacterial orders, beside *Burkholderiales*, it is worth naming the genes of the TA system, which encode a toxin protein capable of inhibiting cell growth and an antitoxin that counteracts the toxin. In *Xylella fastidiosa*, the TA system genes can be encoded by both plasmids and chromosomes, but all genes coding for TA systems derived by HGT with phylogenetically distant bacteria are located on plasmids. Plasmid-encoded TA systems confer stable plasmid inheritance by post-segregational killing of plasmid-free daughter cells [41]. Their diverse origin is in line with the great variability characterizing TA genes in the *Xylella fastidiosa* genome, which can contain multiple copies of the TA prophage region, with each copy bearing a different TA system, depending on the strain or the subspecies [42]. A further relevant finding concerns the relatively large number of genes encoding putative degrading and biosynthetic activities that have no ortholog or close paralog within the *Xanthomonadales*, and appear to originate in widely diverse regions of the prokaryotic tree of life. These genes add to the significant fraction of the genome of *Xylella fastidiosa* that is not annotated due to the lack of similarity with proteins with known function.

### 3.4. Xylella fastidiosa and the Xylem Sap Microbiome

Sequence similarity between *Xylella fastidiosa* and bacteria belonging to the orders *Burkholderiales*, *Rhizobiales,* and *Rhodocyclales* has already been reported [43,44,45,46,47]. Nevertheless, the impact on the *Xylella fastidiosa* pan-genome given by genes coming from evolutionary distant bacteria, systemically analyzed in this study, may appear in contrast with the pathogen lifestyle, confined into xylem vessels.

Xylem is populated by many other xylem-limited bacteria [48,49], and also by endophytes that use it as the main transport route for systemic colonization of the whole plant [50,51,52]. Studies on the composition of the xylem sap microbiome in citrus plants and grapevines showed not only that *Xylella fastidiosa* shares its ecological niche with many other bacteria endophytes, but also that it interacts with them modulating its growth and its gene expression [53,54]. The interconnections between *Xylella fastidiosa* and other bacterial components of the phyllosphere and the natural inclination of *Xylella fastidiosa* to take up DNA from its environment [55] allow us to hypothesize, in the evolutionary history of *Xylella fastidiosa*, frequent genomic exchanges with a variety of phyllosphere inhabiting bacteria, possibly favored by the recruitment of a plasmid conjugation machinery from the *Betaproteobacteria* (that prompted genetic exchange with phyllosphere bacteria) and by the loss of RecX (that enhanced the plasticity of the genome).

The access to this large gene reservoir may have had (and still may have) a role in *Xylella fastidiosa* adaptation capability. It is well known that *Xylella fastidiosa* strains can recombine with each other [56] and recombination among strains is regarded as a pivotal condition for the emergence of new variants with an enlarged or modified host range. For this reason, after the introduction of *Xylella fastidiosa* subsp. *pauca* in Apulia, a major concern in Europe has been the prevention of the introduction of new strains with which the current epidemic strain may recombine and enlarge the host range [46]. Our work, however, stresses the relevance of genes that originated outside the genus *Xylella*. Without questioning the relevance of the risk of creating new genotypes by intraspecific recombination, we evidenced the potential role of HGT from the environmental microbiota for the phytopathogen adaptation and the possibility that preventing intraspecific recombination may not guarantee the avoidance of shifts to new hosts.

## 4. Materials and Methods

### 4.1. Obtaining the Pan-Genomes

Datasets were constructed for three groups of bacteria representing taxonomically and genomically well defined clusters: (i) 65 genomes of *Xanthomonas vasicola*, including *Xanthomonas vasicola* pv. *holcicola* (22 genomes), *Xanthomonas vasicola* pv. *vasculorum* (43 genomes, and 8 genomes of the maize isolates previously named *Xanthomonas vasicola* pv. *zeae*); (ii) 16 genomes of *Xanthomonas albilineans*; (iii) 45 representative genomes of *Xylella fastidiosa* (this figure is lower than the number of all assemblies presently available from public database, which would exceed the computational capacity). Table S1 lists the assemblies used and the total sizes of the predicted proteomes, which range from about 730,000 to about 880,000 amino acids.

The downloaded NCBI-annotated sequences of the genomes of each species (*Xanthomonas vasicola, Xanthomonas albilineans, Xylella fastidiosa*) were submitted to the standalone version of the Orthologous Matrix tool (OMA-standalone; http://omabrowser.org/standalone/) to identify orthologous groups (OGs) of genes. The output was further analyzed with several custom Perl/Bash scripts for the construction of three gene sets, named BUSCO, Core, and Pan-genome. Gene sequences found in only one strain (singletons) were not included in the above-mentioned gene sets. Hence, the pan-genomes identified in this work differ from those publicly available from UNIPROT (ftp://ftp.uniprot.org/pub/databases/uniprot/current_release/knowledgebase/pan_proteomes/) and roughly correspond to the concept of the shell genome used by Vanhove and coworkers [56].

### 4.2. Classification of Gene According to Orthology and Homology Searches

As a reference representative gene product of each OGs, the centroid amino acid sequence resulting from the pairwise distance calculation carried out with ClustalW2 [57] was selected (OGRP: orthologous group reference protein). Each OGRP was submitted to the analysis flow outlined in Figure S1 and described later on in this section, for the identification of genes not shared with other *Xanthomonadals*. The procedure in Figure S1 is based on the integration of the results of BLASTP searches and interrogations of the OMA database, obtained as follows.

Each OG was submitted to BLASTP analysis using a custom database from which all the entries from the genus/species investigated were excluded, to identify the bacteria carrying the most similar genes. The preliminary filtering of the database was carried out with an ad hoc BASH script on the NCBI reference sequence (RefSeq) database. Three BLASTP search sets were carried out on customized databases made with all prokaryotic sequences excluding: (i) for the *Xanthomonas vasicola* pan-genome, all sequences deposited as belonging to *Xanthomonas vasicola* and its pathovars or to *Xanthomonas campestris* pv. *musacearum*, because comparative phylogenomic studies supported its phylogenetic relatedness to *Xanthomonas vasicola* [32,58]; (ii) for the *Xylella fastidiosa* pan-genome, all sequences deposited as belonging to the genus *Xylella*, including *Xylella taiwanensis*; (iii) for the *Xanthomonas albilineans* pan-genome, all sequences deposited as belonging to *Xanthomonas albilineans*, *Xanthomonas pseudoalbilineans*, and the related strains *Xanthomonas* sp. MUS 60 and GPE39, for which the name “*Xanthomonas pseudoalbilineans*” has been proposed. The results of the BLASTP search were then filtered by keeping only hits with e-values lower than 1 × 10^−6^. The BLASTP results were reformatted and each homologous group was thus provided with a taxonomic identifier using the Entrez e-service [59]. When a BLASTP search indicated a best match in a taxon not comprised in the *Xanthomonadales*, the result was further validated with a tBLASTn search in custom databases built from all *Xanthomonadales* genomes included in the JGI database (downloaded in December 2019).

All identifiers assigned by the OMA database (OMAdb; [21]) to the species under investigation were downloaded and assigned to the matching OGRP using the cross-reference service provided by the OMAdb. Due to the limitation of the number of genomes included in the OMAdb, several OGRPs had no identifier with which they could be associated. In such cases, the OMAdb was searched by BLASTP and the best fitting identifier was provisionally associated with the querying OGRP.

As illustrated in Figure S1, the first step of the analysis of each OGRP was to check whether or not its best BLASTP hit was within the class *Xanthomonadales*; in this case the taxID of the class was assigned and the OGRP was not further investigated. The second step was to check whether or not the OGRP under examination had an existing entry in the OMAdb. If the corresponding identifier belonged to a hierarchical orthologous group (HOG; [60]) at the level of the *Xanthomonadales*, the taxID of the class was assigned and the OGRP was not further investigated. If an existing entry could not be found, the OMAdb was homology searched for the most similar identifier: if the best fitting identifier itself or one of its orthologs belonged to an HOG at the level of the *Xanthomonadales*, again the taxID of the class was assigned and the OGRP was not further investigated (step 3).

The OGRPs that have not been excluded to this point were further investigated with the aim of determining the taxon of their putative origin. Using the Python interface to the REST-API of the OMAdb [22] for each OG, all protein sequences recorded as orthologs of the OG-associated identifier were then downloaded and aligned with the OGRP using FASMA [61]. In each global alignment, the four sequences with highest identity scores were selected and their lower common ancestor rank determined using the ETE3 toolkit [62].

Finally, the information obtained from the OMAdb was merged with the results of the BlastP searches of the RefSeq database and checked for taxonomic congruence. In the case of conflict, the taxID assigned to the OG was determined by choosing the higher sequence identity score in global alignment to the OG centroid after alignment of the best BlastP hit sequence and that of the closest protein among the putative ortholog list provided by OMAdb. The results were visualized using the Krona tools [63].

### 4.3. Functional Assignments and Other Analyses

The program SplitByTaxa of the suite BBMap, available at: https//sourceforge.net/projects/bbmap/, was used for discarding the representative gene sequence of each OG in agreement to its taxonomic position according to the previously assigned taxID. An ad hoc Perl script was then used to interrogate the KAAS service [64] for functional classification and KEGG pathway assignment of the representative gene sequences of each OG.

The sliding window plot of tetra correlation values was built using an ad hoc script that calculate tetra z-scores according to [65], and used a window size of 100,000 nts, increasing the positions for calculation by 10,000 nts for each plotted point.

## 5. Conclusions

*Xylella fastidiosa* is phylogenetically strictly related to other xanthomonads, but with a rather atypical, unique phytopathogenic behavior and very wide host range and combination of host–pathogen relationship. In this paper, we show that the *Xylella fastidiosa* pan-genome is not peculiar for its size (it is less than 1.5 times the average genome) nor for its ratio accessory versus core genome. The pan-genome of *Xylella fastidiosa* is instead peculiar for the origin of the genes that it includes, which in a large fraction have not evolved within the *Xanthomonadales* but have rather been acquired by horizontal transfer from distantly related bacteria. The access to the environmental gene pool may have been the key for the species success and, as such, actively pursued.

## Figures and Tables

**Table 1:** Benchmarking Universal Single-Copy Orthologs (BUSCO) genes missing in the genomes of *Xanthomonas vasicola*, *Xanthomonas albilineans*, and *Xylella fastidiosa*.

*Xylella fastidiosa*	*Xanthomonas vasicola*	*Xanthomonas albilineans*	Description
POG0909014H	POG0909014H	POG0909014H	UDP-*N*-acetylmuramoylalanine-d-glutamate ligase
POG090901AA	POG090901AA	POG090901AA	Small ribosomal subunit biogenesis GTPase RsgA
POG090901YD	POG090901YD	POG090901YD	membrane protein
POG09090233	POG09090233	POG09090233	apolipoprotein *N*-acyltransferase
POG090902S9	POG090902S9	POG090902S9	threonylcarbamoyl-AMP synthase
POG090903DC	POG090903DC	POG090903DC	argininosuccinate lyase
POG0909029O	POG0909029O		Thymidylate kinase
		POG090901QT	peptide chain release factor 1
		POG090900DY	tRNA N6-adenosine threonylcarbamoyltransferase
POG090900A4			tRNA modification GTPase MnmE
POG090900LM			Bifunctional uridylyltransferase/uridylyl-removing enzyme
POG0909004H			Bifunctional glutamine synthetase adenylyltransferase/adenylyl-removing enzyme
POG090901JJ			d-aminoacyl-tRNA deacylase
POG090901Q3			RNA-binding protein
POG09090284			tRNA-dihydrouridine synthase B
POG090902SD			membrane protein
POG090903LW			Regulatory protein RecX

**Table 2:** Comparison of the relevant data in the pan-genome analysis of *Xanthomonas vasicola*, *Xanthomonas albilineans* and *Xylella fastidiosa.*

*Xanthomonas vasicola*	*Xanthomonas albilineans*	*Xylella fastidiosa*	
4.9	3.7	2.5	Average genome size (Mbp)
3888	2919	2176	Average number of predicted proteins
			**Core genome:**
1828	2386	1045	Genes searched for orthology and homology
1828 (100%)	2371 (99.4%)	1038 (99.3%)	Genes that have orthologs or close paralogs in the *Xanthomonadales*
0 (0%)	15 (0.6%)	7 (0.7%)	Genes that have orthologs in taxa not belonging to *Xanthomonadales*
			**Pan-genome:**
5296	3536	3673	Protein encoding genes in pan-genome
5155	3369	3049	Genes searched for orthology and homology
5085 (98.6%)	3198 (94.9%)	2395 (78.6%)	Genes that have orthologs or close paralogs in the *Xanthomonadales*
70 (1.4%)	171 (5.1%)	654 (21.4%)	Genes that have orthologs in taxa not belonging to *Xanthomonadales* (Figure 1)

## Data Availability

The study analyzes publicly available data.

## Supplementary Materials

The following are available online at https://www.mdpi.com/2076-0817/10/1/46/s1. Figure S1: Schematic representation of the procedure used for classification of gene according to orthology and homology searches. Figure S2: Taxonomic subdivision of their nearest ortholog assignment of genes in the pan-genome of *Xanthomonas vasicola*. Figure S3: Taxonomic subdivision of their nearest ortholog assignment of genes in the pan-genome of *Xanthomonas albilineans.* Figure S4: Taxonomic subdivision of their nearest ortholog assignment of genes in the pan-genome of *Xylella fastidiosa.* Figure S5: Sliding window graphs of the tetra correlation values between a 100 k window and the entire chromosome. The DNA sequence of the chromosomes of 12 *X. fastidiosa* strains were scanned and for each position (*x*-axis, nucleotide position in genome) the tetra correlation value calculated between the 100,000 nucleotides following that position and the entire chromosome sequence was reported in the *y*-axis. Table S1: List of assemblies used for the construction of the pan-genomes of *Xanthomonas vasicola, Xanthomonas albilineans* and *Xylella fastidiosa* and total sizes of their predicted proteomes. Table S2: Genes of the pan-genome of *Xylella fastidiosa* without HOG in the *Xanthamonadales,* listed according to their KEGG pathways or GO terms. Table S3: List of the annotation of degrading and synthetic enzyme encoding genes in the pan-genome of *Xylella fastidiosa* that have no homologues in the *Xanthomonadales*. The column on the right reports the detected number of groups of ortholog genes found analyzing 45 genomes of *Xylella fastidiosa.*
