# Supplementary material for: Orthology-Based Estimate of the Contribution of Horizontal Gene Transfer from Distantly Related Bacteria to the Intraspecific Diversity and Differentiation of Xylella fastidiosa
Source: Pathogens. 2021 Jan 7;10(1):46. doi: 10.3390/pathogens10010046 (PMC7828034; doi:10.3390/pathogens10010046)
Supplement: Supplementary file 1 [file pathogens-10-00046-s001.zip › pathogens-1031631-supplementary-final/pathogens-1031631-Figure S1.docx]

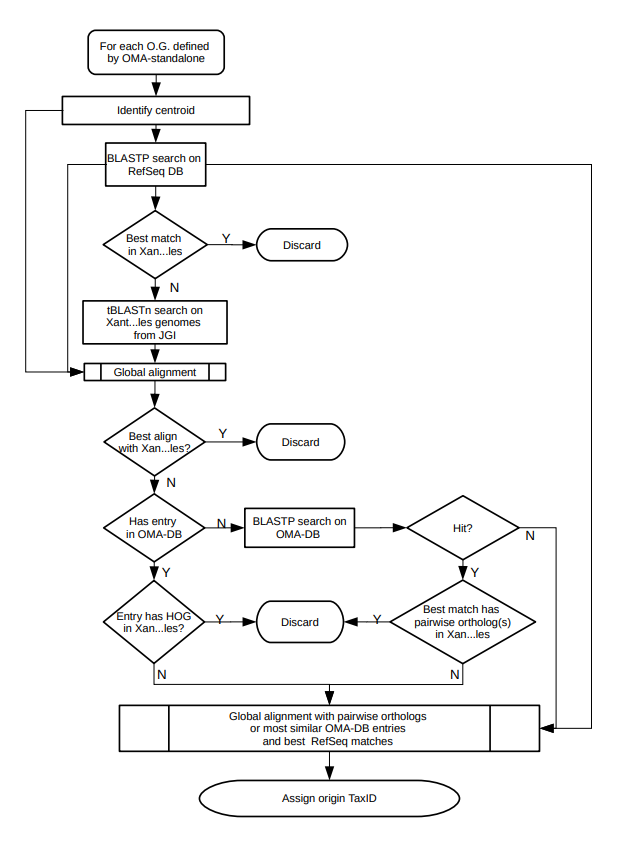


**Figure S1**. Schematic representation of the procedure used for classification of gene according to orthology and homology searches.
