# Supplementary material for: Orthology-Based Estimate of the Contribution of Horizontal Gene Transfer from Distantly Related Bacteria to the Intraspecific Diversity and Differentiation of Xylella fastidiosa
Source: Pathogens. 2021 Jan 7;10(1):46. doi: 10.3390/pathogens10010046 (PMC7828034; doi:10.3390/pathogens10010046)
Supplement: Supplementary file 1 [file pathogens-10-00046-s001.zip › pathogens-1031631-supplementary-final/pathogens-1031631-Figure S2.html]

Javascript must be enabled to view this page.

members
magnitude
magnitudeUnassigned
count
unassigned
taxon
rank

Busco
Core
Pangenome

44518285155

44518285155
superkingdom
2

phylum
1224
44518285154

1
68525
subphylum

1
class
28221

1
order
29

1
suborder
80811

1
family
39

40
genus
1

41 
species


OG02326|WP\_010365224.1 | MULTISPECIES: hypothetical protein | taxID used:41 
1

28211
class
8

order
204441
1

433
family
1

1
genus
522

1
62140 


OG00186|WP\_039442996.1 | hypothetical protein [Xanthomonas v | taxID used:62140 
species

7
356
order

82115
family
1

genus
357
1

1183400
species group
1

1
species


OG03694|WP\_080764585.1 | GGDEF domain-containing protein [Xa | taxID used:358 
358 

1
family
69277

1


OG04654|WP\_017170373.1 | hypothetical protein [Xanthomonas v | taxID used:2267839 
species
2267839 

4
family
119045

4
genus
407


OG01507|WP\_010363042.1 | hypothetical protein [Xanthomonas v | taxID used:31998 OG04406|WP\_010368841.1 | hypothetical protein [Xanthomonas v | taxID used:31998 OG05198|WP\_010368860.1 | hypothetical protein [Xanthomonas v | taxID used:31998 
species
31998 
3

1
1736253 
species


OG05266|WP\_017116858.1 | MULTISPECIES: IS3 family transposas | taxID used:1736253 

1
335928
family

204476
genus
1

1
species


OG04421|WP\_017173689.1 | MULTISPECIES: type II toxin-antitox | taxID used:1737983 
1737983 

44518285129
1236
class

135622
order
1

267893
family
1

1
genus
135575

species


OG04099|WP\_052263122.1 | type III toxin-antitoxin system Tox | taxID used:86102 
86102 
1

5
91347
order

1903411
family
1

1
629
genus

species group
1649845
1

1


OG01561|WP\_087911173.1 | hypothetical protein [Xanthomonas v | taxID used:633 
species
633 

1
family
1903409

1
551
genus

1


OG04362|WP\_050557313.1 | hypothetical protein [Xanthomonas v | taxID used:65700 
species
65700 

3
family
543

570
genus
1

1
species


OG04296|WP\_017154708.1 | MULTISPECIES: hypothetical protein | taxID used:1463165 
1463165 

590
genus
1

species


OG01802|WP\_017117962.1 | IS5/IS1182 family transposase, part | taxID used:28901 
28901 
1

1
547
genus

1594172 
species


OG05174|WP\_010370227.1 | MULTISPECIES: hypothetical protein | taxID used:1594172 
1


OG03971|WP\_010366350.1 | MULTISPECIES: outer membrane protei | taxID used:135614 OG01000|WP\_003486674.1 | MULTISPECIES: preprotein translocas | taxID used:135614 OG01010|WP\_017116753.1 | MULTISPECIES: RIP metalloprotease R | taxID used:135614 OG01036|WP\_010364810.1 | MULTISPECIES: chromosomal replicati | taxID used:135614 OG01049|WP\_010366656.1 | MULTISPECIES: phosphoglucosamine mu | taxID used:135614 OG01054|WP\_010364364.1 | MULTISPECIES: 3-deoxy-D-manno-octul | taxID used:135614 OG01058|WP\_010363992.1 | MULTISPECIES: Tol-Pal system beta p | taxID used:135614 OG01062|WP\_026113071.1 | rod shape-determining protein MreC, | taxID used:135614 OG01063|WP\_010372660.1 | MULTISPECIES: exodeoxyribonuclease | taxID used:135614 OG01070|WP\_054393923.1 | lipid-A-disaccharide synthase, part | taxID used:135614 OG01074|WP\_026112865.1 | MULTISPECIES: tRNA lysidine(34) syn | taxID used:135614 OG01081|WP\_010364845.1 | MULTISPECIES: tRNA uridine-5-carbox | taxID used:135614 OG01089|WP\_010370038.1 | MULTISPECIES: 16S rRNA (cytosine(96 | taxID used:135614 OG01095|WP\_010365705.1 | MULTISPECIES: GTPase HflX [Xanthomo | taxID used:135614 OG01107|WP\_010364452.1 | glutamate-1-semialdehyde 2,1-aminom | taxID used:135614 OG01108|WP\_010368527.1 | MULTISPECIES: trigger factor [Xanth | taxID used:135614 OG01116|WP\_010368449.1 | MULTISPECIES: adenylosuccinate synt | taxID used:135614 OG01119|WP\_010365207.1 | MULTISPECIES: serine--tRNA ligase [ | taxID used:135614 OG00111|WP\_010367825.1 | MULTISPECIES: valine--tRNA ligase [ | taxID used:135614 OG01128|WP\_010368119.1 | phosphoribosylamine--glycine ligase | taxID used:135614 OG01129|WP\_010374708.1 | MULTISPECIES: MFS transporter [Xant | taxID used:135614 OG01131|WP\_010368578.1 | bifunctional tetrahydrofolate synth | taxID used:135614 OG01142|WP\_010367319.1 | MULTISPECIES: glutamyl-tRNA reducta | taxID used:135614 OG01146|WP\_010365670.1 | phosphopyruvate hydratase [Xanthomo | taxID used:135614 OG01148|WP\_003483788.1 | MULTISPECIES: ATP-dependent Clp pro | taxID used:135614 OG00114|WP\_010365817.1 | MULTISPECIES: isoleucine--tRNA liga | taxID used:135614 OG01162|WP\_010371421.1 | flavodoxin-dependent (E)-4-hydroxy- | taxID used:135614 OG00116|WP\_017116155.1 | MULTISPECIES: 2-oxoglutarate dehydr | taxID used:135614 OG01175|WP\_026112776.1 | MULTISPECIES: undecaprenyldiphospho | taxID used:135614 OG01199|WP\_010369231.1 | MULTISPECIES: UDP-N-acetylglucosami | taxID used:135614 OG01202|WP\_010374741.1 | MULTISPECIES: bifunctional phosphop | taxID used:135614 OG01225|WP\_003485272.1 | MULTISPECIES: cell division protein | taxID used:135614 OG01235|WP\_010367620.1 | MULTISPECIES: cell division protein | taxID used:135614 OG01242|WP\_010367140.1 | MULTISPECIES: phosphoglycerate dehy | taxID used:135614 OG01243|WP\_010368176.1 | MULTISPECIES: multifunctional CCA a | taxID used:135614 OG01252|WP\_010373542.1 | MULTISPECIES: 23S rRNA (adenine(250 | taxID used:135614 OG01262|WP\_010367557.1 | MULTISPECIES: methionine adenosyltr | taxID used:135614 OG01284|WP\_026112729.1 | beta-ketoacyl-[acyl-carrier-protein | taxID used:135614 OG00128|WP\_026112731.1 | MULTISPECIES: bifunctional [glutama | taxID used:135614 OG00134|WP\_010367615.1 | MULTISPECIES: preprotein translocas | taxID used:135614 OG01361|WP\_010364501.1 | MULTISPECIES: radical SAM family he | taxID used:135614 OG01379|WP\_010366159.1 | 1-deoxy-D-xylulose-5-phosphate redu | taxID used:135614 OG01382|WP\_010372314.1 | MULTISPECIES: tRNA guanosine(34) tr | taxID used:135614 OG01389|WP\_010366204.1 | MULTISPECIES: ADP-forming succinate | taxID used:135614 OG01401|WP\_010370862.1 | MULTISPECIES: phosphoglycerate kina | taxID used:135614 OG01406|WP\_010373581.1 | tRNA 2-thiouridine(34) synthase Mnm | taxID used:135614 OG01416|WP\_010367166.1 | MULTISPECIES: bifunctional histidin | taxID used:135614 OG01422|WP\_010366364.1 | MULTISPECIES: molecular chaperone D | taxID used:135614 OG00142|WP\_010371657.1 | MULTISPECIES: pyruvate dehydrogenas | taxID used:135614 OG01431|WP\_010367112.1 | MULTISPECIES: carbamoyl-phosphate s | taxID used:135614 OG01434|WP\_017116901.1 | MULTISPECIES: DNA-protecting protei | taxID used:135614 OG01436|WP\_017116805.1 | MULTISPECIES: 5-(carboxyamino)imida | taxID used:135614 OG01450|WP\_010366193.1 | MULTISPECIES: succinyl-diaminopimel | taxID used:135614 OG01455|WP\_097370619.1 | MULTISPECIES: peptide chain release | taxID used:135614 OG01475|WP\_010374699.1 | MULTISPECIES: anhydro-N-acetylmuram | taxID used:135614 OG00147|WP\_054393990.1 | translation initiation factor IF-2, | taxID used:135614 OG01495|WP\_010367803.1 | MULTISPECIES: LPS export ABC transp | taxID used:135614 OG01500|WP\_010366687.1 | chorismate synthase, partial [Xanth | taxID used:135614 OG01512|WP\_010364805.1 | MULTISPECIES: DNA replication/repai | taxID used:135614 OG01533|WP\_010367705.1 | MULTISPECIES: bifunctional diaminoh | taxID used:135614 OG01535|WP\_010369115.1 | 3-dehydroquinate synthase, partial | taxID used:135614 OG01539|WP\_010363474.1 | MULTISPECIES: A/G-specific adenine | taxID used:135614 OG00153|WP\_010377530.1 | leucine--tRNA ligase, partial [Xant | taxID used:135614 OG01546|WP\_010367638.1 | MULTISPECIES: phospho-N-acetylmuram | taxID used:135614 OG01551|WP\_017115279.1 | MULTISPECIES: 3-phosphoserine/phosp | taxID used:135614 OG01557|WP\_010364809.1 | MULTISPECIES: DNA polymerase III su | taxID used:135614 OG01575|WP\_010367805.1 | MULTISPECIES: LPS export ABC transp | taxID used:135614 OG01576|WP\_010367320.1 | MULTISPECIES: peptide chain release | taxID used:135614 OG00157|WP\_010366603.1 | MULTISPECIES: DNA gyrase subunit A | taxID used:135614 OG01590|WP\_010367296.1 | MULTISPECIES: redox-regulated ATPas | taxID used:135614 OG01604|WP\_010372318.1 | MULTISPECIES: tRNA preQ1(34) S-aden | taxID used:135614 OG01611|WP\_010368412.1 | endolytic transglycosylase MltG, pa | taxID used:135614 OG01617|WP\_010364384.1 | MULTISPECIES: 3-isopropylmalate deh | taxID used:135614 OG01618|WP\_010369111.1 | MULTISPECIES: uroporphyrinogen deca | taxID used:135614 OG01633|WP\_010371434.1 | MULTISPECIES: UDP-N-acetylmuramate | taxID used:135614 OG01646|WP\_010365815.1 | bifunctional riboflavin kinase/FAD | taxID used:135614 OG01649|WP\_010369242.1 | MULTISPECIES: phosphoribosylformylg | taxID used:135614 OG01651|WP\_010368929.1 | MULTISPECIES: biotin synthase BioB | taxID used:135614 OG01654|WP\_054394003.1 | MULTISPECIES: tRNA (adenosine(37)-N | taxID used:135614 OG01656|WP\_010367649.1 | MULTISPECIES: 16S rRNA (cytosine(14 | taxID used:135614 OG01666|WP\_010365805.1 | MULTISPECIES: GTPase ObgE [Xanthomo | taxID used:135614 OG01672|WP\_010373330.1 | MULTISPECIES: tetraacyldisaccharide | taxID used:135614 OG01678|WP\_010368259.1 | MULTISPECIES: rod shape-determining | taxID used:135614 OG00167|WP\_010365719.1 | MULTISPECIES: alanine--tRNA ligase | taxID used:135614 OG01685|WP\_010371435.1 | MULTISPECIES: quinone-dependent dih | taxID used:135614 OG01695|WP\_010368064.1 | MULTISPECIES: anthranilate phosphor | taxID used:135614 OG01696|WP\_010368229.1 | MULTISPECIES: lipoyl synthase [Xant | taxID used:135614 OG01702|WP\_010363979.1 | MULTISPECIES: Holliday junction bra | taxID used:135614 OG01718|WP\_010365715.1 | MULTISPECIES: recombinase RecA [Xan | taxID used:135614 OG01725|WP\_010368675.1 | MULTISPECIES: nucleoside triphospha | taxID used:135614 OG01734|WP\_010374916.1 | MULTISPECIES: tRNA dihydrouridine(2 | taxID used:135614 OG01740|WP\_010366823.1 | MULTISPECIES: DNA polymerase III su | taxID used:135614 OG01747|WP\_017112367.1 | MULTISPECIES: RNA polymerase-bindin | taxID used:135614 OG01751|WP\_010370589.1 | MULTISPECIES: NAD(P)-dependent glyc | taxID used:135614 OG01765|WP\_010367549.1 | MULTISPECIES: tRNA dihydrouridine s | taxID used:135614 OG01773|WP\_010366151.1 | UDP-3-O-(3-hydroxymyristoyl)glucosa | taxID used:135614 OG01780|WP\_010363591.1 | MULTISPECIES: phenylalanine--tRNA l | taxID used:135614 OG01787|WP\_010373500.1 | MULTISPECIES: exodeoxyribonuclease | taxID used:135614 OG01819|WP\_010366210.1 | MULTISPECIES: 23S rRNA pseudouridin | taxID used:135614 OG00181|WP\_010366185.1 | MULTISPECIES: bifunctional uridylyl | taxID used:135614 OG01827|WP\_002811635.1 | MULTISPECIES: DNA-directed RNA poly | taxID used:135614 OG01829|WP\_010369386.1 | MULTISPECIES: octaprenyl-diphosphat | taxID used:135614 OG01857|WP\_010365699.1 | MULTISPECIES: tRNA (adenosine(37)-N | taxID used:135614 OG01858|WP\_017115996.1 | MULTISPECIES: ferrochelatase [Xanth | taxID used:135614 OG01859|WP\_010367690.1 | MULTISPECIES: thiamine-phosphate ki | taxID used:135614 OG00185|WP\_017116712.1 | DNA mismatch repair protein MutS, p | taxID used:135614 OG01864|WP\_010373361.1 | 23S rRNA pseudouridine(955/2504/258 | taxID used:135614 OG01871|WP\_003482910.1 | MULTISPECIES: thioredoxin-disulfide | taxID used:135614 OG01875|WP\_010367471.1 | MULTISPECIES: symmetrical bis(5'-nu | taxID used:135614 OG00188|WP\_010366466.1 | MULTISPECIES: ribonuclease R [Xanth | taxID used:135614 OG01903|WP\_010367467.1 | 4-hydroxythreonine-4-phosphate dehy | taxID used:135614 OG00190|WP\_010363370.1 | MULTISPECIES: ATP-dependent chapero | taxID used:135614 OG01923|WP\_010366140.1 | MULTISPECIES: acetyl-CoA carboxylas | taxID used:135614 OG01928|WP\_010367308.1 | MULTISPECIES: ribose-phosphate pyro | taxID used:135614 OG01938|WP\_010365823.1 | 4-hydroxy-3-methylbut-2-enyl diphos | taxID used:135614 OG01968|WP\_017112290.1 | MULTISPECIES: bifunctional biotin-- | taxID used:135614 OG00197|WP\_010364539.1 | MULTISPECIES: penicillin-binding pr | taxID used:135614 OG01985|WP\_010368132.1 | MULTISPECIES: 50S ribosomal protein | taxID used:135614 OG01988|WP\_010374452.1 | MULTISPECIES: glycine--tRNA ligase | taxID used:135614 OG01993|WP\_010374500.1 | MULTISPECIES: tRNA 2-thiocytidine(3 | taxID used:135614 OG00019|WP\_010367275.1 | DNA-directed RNA polymerase subunit | taxID used:135614 OG02008|WP\_010366690.1 | MULTISPECIES: 50S ribosomal protein | taxID used:135614 OG02027|WP\_010370042.1 | methionyl-tRNA formyltransferase, p | taxID used:135614 OG02031|WP\_010367619.1 | MULTISPECIES: UDP-3-O-[3-hydroxymyr | taxID used:135614 OG02034|WP\_017115756.1 | MULTISPECIES: 4-hydroxybenzoate oct | taxID used:135614 OG00204|WP\_010366593.1 | MULTISPECIES: NAD-dependent DNA lig | taxID used:135614 OG02057|WP\_010371225.1 | MULTISPECIES: tRNA pseudouridine(55 | taxID used:135614 OG00206|WP\_084818999.1 | MULTISPECIES: LPS-assembly protein | taxID used:135614 OG02070|WP\_010366209.1 | MULTISPECIES: outer membrane protei | taxID used:135614 OG02074|WP\_010367173.1 | MULTISPECIES: ATP phosphoribosyltra | taxID used:135614 OG02081|WP\_010367474.1 | MULTISPECIES: prolipoprotein diacyl | taxID used:135614 OG02083|WP\_010368308.1 | MULTISPECIES: hydroxymethylbilane s | taxID used:135614 OG02093|WP\_010366658.1 | acetyl-CoA carboxylase carboxyltran | taxID used:135614 OG00210|WP\_010370069.1 | MULTISPECIES: DNA topoisomerase I [ | taxID used:135614 OG02119|WP\_026113080.1 | MULTISPECIES: GTPase Era [Xanthomon | taxID used:135614 OG02128|WP\_039431545.1 | MULTISPECIES: SPOR domain-containin | taxID used:135614 OG02142|WP\_010368716.1 | MULTISPECIES: Hsp33 family molecula | taxID used:135614 OG02150|WP\_010370120.1 | MULTISPECIES: RNA polymerase sigma | taxID used:135614 OG02157|WP\_010369215.1 | MULTISPECIES: RNase adapter RapZ [X | taxID used:135614 OG02163|WP\_010372697.1 | MULTISPECIES: protease HtpX [Xantho | taxID used:135614 OG02165|WP\_010367313.1 | MULTISPECIES: 4-(cytidine 5'-diphos | taxID used:135614 OG02176|WP\_010371995.1 | MULTISPECIES: F0F1 ATP synthase sub | taxID used:135614 OG02178|WP\_026112837.1 | MULTISPECIES: folate-binding protei | taxID used:135614 OG02179|WP\_008571326.1 | MULTISPECIES: CBS domain-containing | taxID used:135614 OG02185|WP\_010366205.1 | MULTISPECIES: succinate--CoA ligase | taxID used:135614 OG00218|WP\_010366154.1 | MULTISPECIES: outer membrane protei | taxID used:135614 OG00021|WP\_010367276.1 | MULTISPECIES: DNA-directed RNA poly | taxID used:135614 OG02204|WP\_017117278.1 | MULTISPECIES: cell division protein | taxID used:135614 OG02205|WP\_010366766.1 | MULTISPECIES: polyprenyl synthetase | taxID used:135614 OG02210|WP\_010368294.1 | MULTISPECIES: diaminopimelate epime | taxID used:135614 OG02214|WP\_010366169.1 | MULTISPECIES: elongation factor Ts | taxID used:135614 OG02224|WP\_010373538.1 | MULTISPECIES: helix-turn-helix doma | taxID used:135614 OG02252|WP\_010366694.1 | MULTISPECIES: phosphatidylserine de | taxID used:135614 OG02264|WP\_010369318.1 | MULTISPECIES: pyrroline-5-carboxyla | taxID used:135614 OG02265|WP\_010371383.1 | MULTISPECIES: pantoate--beta-alanin | taxID used:135614 OG02271|WP\_010366728.1 | MULTISPECIES: iron-sulfur cluster c | taxID used:135614 OG02278|WP\_010367388.1 | MULTISPECIES: peptide chain release | taxID used:135614 OG02288|WP\_010365668.1 | MULTISPECIES: 2-dehydro-3-deoxyphos | taxID used:135614 OG02290|WP\_010374937.1 | MULTISPECIES: shikimate dehydrogena | taxID used:135614 OG02292|WP\_010371059.1 | MULTISPECIES: 50S ribosomal protein | taxID used:135614 OG00229|WP\_010368518.1 | MULTISPECIES: endopeptidase La [Xan | taxID used:135614 OG00230|WP\_010364803.1 | MULTISPECIES: DNA topoisomerase (AT | taxID used:135614 OG02335|WP\_010363730.1 | MULTISPECIES: NADPH-dependent 7-cya | taxID used:135614 OG02336|WP\_010372010.1 | MULTISPECIES: F0F1 ATP synthase sub | taxID used:135614 OG02339|WP\_010374685.1 | MULTISPECIES: bifunctional DNA-form | taxID used:135614 OG02352|WP\_010367473.1 | MULTISPECIES: thymidylate synthase | taxID used:135614 OG02366|WP\_010367663.1 | MULTISPECIES: 16S rRNA (cytidine(14 | taxID used:135614 OG02389|WP\_017116796.1 | MULTISPECIES: tryptophan synthase s | taxID used:135614 OG02391|WP\_010366170.1 | MULTISPECIES: 30S ribosomal protein | taxID used:135614 OG02406|WP\_080762822.1 | MULTISPECIES: uroporphyrinogen-III | taxID used:135614 OG02418|WP\_010365674.1 | MULTISPECIES: 2-C-methyl-D-erythrit | taxID used:135614 OG02421|WP\_010366148.1 | MULTISPECIES: acyl-ACP--UDP-N-acety | taxID used:135614 OG02441|WP\_010368065.1 | MULTISPECIES: indole-3-glycerol pho | taxID used:135614 OG02443|WP\_010366672.1 | MULTISPECIES: tRNA pseudouridine(38 | taxID used:135614 OG02446|WP\_010367469.1 | MULTISPECIES: 16S rRNA (adenine(151 | taxID used:135614 OG02448|WP\_010373332.1 | MULTISPECIES: 3-deoxy-manno-octulos | taxID used:135614 OG02465|WP\_010364916.1 | MULTISPECIES: ATP-binding cassette | taxID used:135614 OG02468|WP\_010367160.1 | MULTISPECIES: imidazole glycerol ph | taxID used:135614 OG02478|WP\_010365897.1 | MULTISPECIES: membrane protein [Xan | taxID used:135614 OG02485|WP\_010380112.1 | MULTISPECIES: NAD kinase [Xanthomon | taxID used:135614 OG02492|WP\_010368574.1 | MULTISPECIES: colicin V biosynthesi | taxID used:135614 OG02495|WP\_010366164.1 | MULTISPECIES: di-trans,poly-cis-dec | taxID used:135614 OG00024|WP\_010367790.1 | MULTISPECIES: phosphoribosylformylg | taxID used:135614 OG02507|WP\_010368508.1 | MULTISPECIES: hydroxyacylglutathion | taxID used:135614 OG02508|WP\_010370997.1 | tRNA (guanosine(46)-N7)-methyltrans | taxID used:135614 OG02534|WP\_010365910.1 | MULTISPECIES: tRNA (guanosine(37)-N | taxID used:135614 OG02575|WP\_010372752.1 | MULTISPECIES: RNA methyltransferase | taxID used:135614 OG00025|WP\_039432078.1 | MULTISPECIES: exodeoxyribonuclease | taxID used:135614 OG02604|WP\_010371152.1 | MULTISPECIES: triose-phosphate isom | taxID used:135614 OG02607|WP\_010368561.1 | MULTISPECIES: UDP-2,3-diacylglucosa | taxID used:135614 OG02615|WP\_010368928.1 | MULTISPECIES: amidophosphoribosyltr | taxID used:135614 OG02623|WP\_010370124.1 | MULTISPECIES: uracil-DNA glycosylas | taxID used:135614 OG02625|WP\_005993372.1 | MULTISPECIES: 30S ribosomal protein | taxID used:135614 OG02628|WP\_010366472.1 | MULTISPECIES: 23S rRNA (guanosine(2 | taxID used:135614 OG02632|WP\_010366586.1 | MULTISPECIES: cell division protein | taxID used:135614 OG02636|WP\_010368405.1 | 3-oxoacyl-ACP reductase FabG, parti | taxID used:135614 OG02640|WP\_010364914.1 | MULTISPECIES: ABC transporter perme | taxID used:135614 OG02645|WP\_010366145.1 | MULTISPECIES: ribonuclease HII [Xan | taxID used:135614 OG02650|WP\_010374583.1 | orotidine-5'-phosphate decarboxylas | taxID used:135614 OG02652|WP\_010368502.1 | MULTISPECIES: DNA polymerase III su | taxID used:135614 OG02660|WP\_010367163.1 | MULTISPECIES: 1-(5-phosphoribosyl)- | taxID used:135614 OG02664|WP\_010368667.1 | MULTISPECIES: 16S rRNA (uracil(1498 | taxID used:135614 OG02679|WP\_010372777.1 | MULTISPECIES: bifunctional 2-polypr | taxID used:135614 OG00267|WP\_010363589.1 | MULTISPECIES: phenylalanine--tRNA l | taxID used:135614 OG02691|WP\_010364507.1 | MULTISPECIES: ribonuclease PH [Xant | taxID used:135614 OG02694|WP\_010365972.1 | MULTISPECIES: DNA repair protein Re | taxID used:135614 OG02698|WP\_010366166.1 | MULTISPECIES: UMP kinase [Xanthomon | taxID used:135614 OG02699|WP\_010366501.1 | MULTISPECIES: endonuclease III [Xan | taxID used:135614 OG02725|WP\_008572666.1 | MULTISPECIES: LPS export ABC transp | taxID used:135614 OG02734|WP\_010367114.1 | MULTISPECIES: 4-hydroxy-tetrahydrod | taxID used:135614 OG02747|WP\_010368230.1 | MULTISPECIES: lipoyl(octanoyl) tran | taxID used:135614 OG02755|WP\_010364199.1 | tRNA (adenosine(37)-N6)-threonylcar | taxID used:135614 OG02769|WP\_010369321.1 | MULTISPECIES: YggS family pyridoxal | taxID used:135614 OG02796|WP\_010367280.1 | MULTISPECIES: 50S ribosomal protein | taxID used:135614 OG02828|WP\_010365969.1 | MULTISPECIES: ribonuclease III [Xan | taxID used:135614 OG02836|WP\_017112868.1 | MULTISPECIES: outer membrane lipopr | taxID used:135614 OG02837|WP\_010368049.1 | MULTISPECIES: ribulose-phosphate 3- | taxID used:135614 OG02840|WP\_010369238.1 | MULTISPECIES: phosphoribosylglycina | taxID used:135614 OG02858|WP\_010373008.1 | MULTISPECIES: (d)CMP kinase [Xantho | taxID used:135614 OG02861|WP\_010364121.1 | MULTISPECIES: 7-cyano-7-deazaguanin | taxID used:135614 OG02901|WP\_010367315.1 | MULTISPECIES: lipoprotein localizat | taxID used:135614 OG02921|WP\_010366476.1 | MULTISPECIES: ribonuclease T [Xanth | taxID used:135614 OG02936|WP\_010371052.1 | MULTISPECIES: 50S ribosomal protein | taxID used:135614 OG02952|WP\_054393896.1 | MULTISPECIES: ribose-5-phosphate is | taxID used:135614 OG02958|WP\_010374714.1 | MULTISPECIES: orotate phosphoribosy | taxID used:135614 OG02962|WP\_010373338.1 | MULTISPECIES: CDP-diacylglycerol--g | taxID used:135614 OG02975|WP\_010372598.1 | MULTISPECIES: stringent starvation | taxID used:135614 OG02984|WP\_010365694.1 | MULTISPECIES: 23S rRNA (uridine(255 | taxID used:135614 OG02987|WP\_010363800.1 | MULTISPECIES: dephospho-CoA kinase | taxID used:135614 OG02997|WP\_010367304.1 | MULTISPECIES: 50S ribosomal protein | taxID used:135614 OG03020|WP\_010374347.1 | 16S rRNA (guanine(527)-N(7))-methyl | taxID used:135614 OG03026|WP\_010373536.1 | MULTISPECIES: tetratricopeptide rep | taxID used:135614 OG03029|WP\_002806026.1 | MULTISPECIES: ATP-dependent Clp pro | taxID used:135614 OG03034|WP\_010371121.1 | MULTISPECIES: 30S ribosomal protein | taxID used:135614 OG03037|WP\_054393919.1 | MULTISPECIES: YihA family ribosome | taxID used:135614 OG03050|WP\_010364511.1 | MULTISPECIES: guanylate kinase [Xan | taxID used:135614 OG03051|WP\_010368084.1 | MULTISPECIES: RNA pyrophosphohydrol | taxID used:135614 OG03060|WP\_010372373.1 | MULTISPECIES: 16S rRNA (guanine(966 | taxID used:135614 OG00308|WP\_010366217.1 | DNA topoisomerase IV subunit A, par | taxID used:135614 OG03093|WP\_026112871.1 | MULTISPECIES: lysogenization regula | taxID used:135614 OG03095|WP\_010364503.1 | MULTISPECIES: non-canonical purine | taxID used:135614 OG03101|WP\_010373369.1 | MULTISPECIES: NfuA family Fe-S biog | taxID used:135614 OG03119|WP\_010367164.1 | MULTISPECIES: imidazole glycerol ph | taxID used:135614 OG03142|WP\_010371054.1 | MULTISPECIES: 50S ribosomal protein | taxID used:135614 OG03143|WP\_010364471.1 | MULTISPECIES: 5-formyltetrahydrofol | taxID used:135614 OG03155|WP\_010373214.1 | MULTISPECIES: oligoribonuclease [Xa | taxID used:135614 OG00315|WP\_010370101.1 | MULTISPECIES: primosomal protein N' | taxID used:135614 OG03171|WP\_010365139.1 | MULTISPECIES: recombination protein | taxID used:135614 OG03187|WP\_010366360.1 | MULTISPECIES: nucleotide exchange f | taxID used:135614 OG03200|WP\_010371208.1 | MULTISPECIES: ribosome maturation f | taxID used:135614 OG03212|WP\_010367298.1 | MULTISPECIES: aminoacyl-tRNA hydrol | taxID used:135614 OG03235|WP\_010369220.1 | MULTISPECIES: lipopolysaccharide tr | taxID used:135614 OG03236|WP\_010366776.1 | MULTISPECIES: DUF615 domain-contain | taxID used:135614 OG03241|WP\_010363969.1 | MULTISPECIES: Holliday junction bra | taxID used:135614 OG00324|WP\_010374449.1 | MULTISPECIES: glycine--tRNA ligase | taxID used:135614 OG03272|WP\_010369349.1 | MULTISPECIES: YqgE/AlgH family prot | taxID used:135614 OG03281|WP\_010367286.1 | MULTISPECIES: transcription termina | taxID used:135614 OG03291|WP\_010370072.1 | MULTISPECIES: tRNA threonylcarbamoy | taxID used:135614 OG03322|WP\_080762940.1 | MULTISPECIES: translation initiatio | taxID used:135614 OG03325|WP\_010366165.1 | MULTISPECIES: ribosome recycling fa | taxID used:135614 OG03336|WP\_010369223.1 | 3-deoxy-D-manno-octulosonate 8-phos | taxID used:135614 OG03338|WP\_010364422.1 | MULTISPECIES: adenylate kinase [Xan | taxID used:135614 OG00333|WP\_010364523.1 | MULTISPECIES: bifunctional (p)ppGpp | taxID used:135614 OG03343|WP\_010369117.1 | MULTISPECIES: shikimate kinase [Xan | taxID used:135614 OG03345|WP\_010371090.1 | MULTISPECIES: 50S ribosomal protein | taxID used:135614 OG03379|WP\_010364486.1 | MULTISPECIES: hypothetical protein | taxID used:135614 OG03385|WP\_003486682.1 | MULTISPECIES: 30S ribosomal protein | taxID used:135614 OG03392|WP\_010373213.1 | MULTISPECIES: tRNA adenosine(34) de | taxID used:135614 OG00033|WP\_017115681.1 | MULTISPECIES: Rne/Rng family ribonu | taxID used:135614 OG03414|WP\_003490311.1 | MULTISPECIES: transcriptional regul | taxID used:135614 OG03431|WP\_010363966.1 | MULTISPECIES: crossover junction en | taxID used:135614 OG03435|WP\_010367278.1 | MULTISPECIES: 50S ribosomal protein | taxID used:135614 OG03449|WP\_010365908.1 | ribosome maturation factor RimM, pa | taxID used:135614 OG03454|WP\_010371099.1 | MULTISPECIES: 50S ribosomal protein | taxID used:135614 OG03460|WP\_010371999.1 | MULTISPECIES: F0F1 ATP synthase sub | taxID used:135614 OG03489|WP\_010370592.1 | MULTISPECIES: protein-export chaper | taxID used:135614 OG03494|WP\_010368399.1 | MULTISPECIES: characterized ACR pro | taxID used:135614 OG03497|WP\_010366344.1 | MULTISPECIES: SsrA-binding protein | taxID used:135614 OG00034|WP\_010366143.1 | DNA polymerase III subunit alpha, p | taxID used:135614 OG03517|WP\_010372369.1 | pantetheine-phosphate adenylyltrans | taxID used:135614 OG03519|WP\_010371236.1 | MULTISPECIES: 5-(carboxyamino)imida | taxID used:135614 OG00351|WP\_010364181.1 | MULTISPECIES: bifunctional (p)ppGpp | taxID used:135614 OG03551|WP\_010367472.1 | MULTISPECIES: dihydrofolate reducta | taxID used:135614 OG03552|WP\_010365676.1 | MULTISPECIES: 2-C-methyl-D-erythrit | taxID used:135614 OG03555|WP\_010372669.1 | MULTISPECIES: tRNA (adenosine(37)-N | taxID used:135614 OG03567|WP\_019768432.1 | MULTISPECIES: ribosomal-protein-ala | taxID used:135614 OG00356|WP\_017115723.1 | MULTISPECIES: bifunctional 23S rRNA | taxID used:135614 OG03585|WP\_010365717.1 | MULTISPECIES: recombination regulat | taxID used:135614 OG03587|WP\_010368256.1 | MULTISPECIES: rod shape-determining | taxID used:135614 OG03588|WP\_010368146.1 | MULTISPECIES: acetyl-CoA carboxylas | taxID used:135614 OG03589|WP\_010372569.1 | MULTISPECIES: rRNA maturation RNase | taxID used:135614 OG00359|WP\_010364527.1 | ATP-dependent DNA helicase RecG, pa | taxID used:135614 OG03613|WP\_010370568.1 | MULTISPECIES: tRNA (cytidine(34)-2' | taxID used:135614 OG03638|WP\_010366796.1 | 23S rRNA (pseudouridine(1915)-N(3)) | taxID used:135614 OG03652|WP\_005917592.1 | MULTISPECIES: 30S ribosomal protein | taxID used:135614 OG03661|WP\_010367695.1 | MULTISPECIES: transcription antiter | taxID used:135614 OG03665|WP\_011408858.1 | MULTISPECIES: transcription elongat | taxID used:135614 OG03676|WP\_010375880.1 | MULTISPECIES: preprotein translocas | taxID used:135614 OG03677|WP\_010366149.1 | MULTISPECIES: 3-hydroxyacyl-[acyl-c | taxID used:135614 OG03698|WP\_010372001.1 | MULTISPECIES: F0F1 ATP synthase sub | taxID used:135614 OG03700|WP\_010369353.1 | MULTISPECIES: Holliday junction res | taxID used:135614 OG03773|WP\_017117508.1 | MULTISPECIES: RnfABCDGE type electr | taxID used:135614 OG03780|WP\_017116181.1 | MULTISPECIES: ClpXP protease specif | taxID used:135614 OG03796|WP\_010366584.1 | MULTISPECIES: 50S ribosomal protein | taxID used:135614 OG03797|WP\_010364838.1 | MULTISPECIES: ribonuclease P protei | taxID used:135614 OG03812|WP\_005991245.1 | MULTISPECIES: 30S ribosomal protein | taxID used:135614 OG03817|WP\_010370007.1 | MULTISPECIES: D-tyrosyl-tRNA(Tyr) d | taxID used:135614 OG03825|WP\_002804358.1 | MULTISPECIES: ferric iron uptake tr | taxID used:135614 OG03832|WP\_003483082.1 | MULTISPECIES: 50S ribosomal protein | taxID used:135614 OG03834|WP\_003486676.1 | MULTISPECIES: 50S ribosomal protein | taxID used:135614 OG00384|WP\_010371227.1 | MULTISPECIES: polyribonucleotide nu | taxID used:135614 OG00385|WP\_010366105.1 | MULTISPECIES: methionine--tRNA liga | taxID used:135614 OG03866|WP\_003490202.1 | DNA polymerase III subunit chi, par | taxID used:135614 OG03881|WP\_010373544.1 | MULTISPECIES: nucleoside-diphosphat | taxID used:135614 OG03890|WP\_010367282.1 | MULTISPECIES: 50S ribosomal protein | taxID used:135614 OG03892|WP\_003486706.1 | MULTISPECIES: 50S ribosomal protein | taxID used:135614 OG03916|WP\_010371989.1 | MULTISPECIES: F0F1 ATP synthase sub | taxID used:135614 OG03924|WP\_005912943.1 | MULTISPECIES: ribosome silencing fa | taxID used:135614 OG03941|WP\_010367292.1 | MULTISPECIES: preprotein translocas | taxID used:135614 OG03964|WP\_010365921.1 | MULTISPECIES: RNA-binding S4 domain | taxID used:135614 OG03977|WP\_005989873.1 | MULTISPECIES: 50S ribosomal protein | taxID used:135614 OG04031|WP\_003486671.1 | MULTISPECIES: 30S ribosomal protein | taxID used:135614 OG04036|WP\_010371223.1 | MULTISPECIES: 30S ribosome-binding | taxID used:135614 OG04037|WP\_010371096.1 | MULTISPECIES: 30S ribosomal protein | taxID used:135614 OG04049|WP\_005990700.1 | MULTISPECIES: 30S ribosomal protein | taxID used:135614 OG04082|WP\_003486665.1 | MULTISPECIES: 50S ribosomal protein | taxID used:135614 OG04105|WP\_010367274.1 | MULTISPECIES: 30S ribosomal protein | taxID used:135614 OG04107|WP\_017115517.1 | MULTISPECIES: DUF423 domain-contain | taxID used:135614 OG04123|WP\_010365672.1 | MULTISPECIES: cell division protein | taxID used:135614 OG04134|WP\_002811674.1 | MULTISPECIES: 50S ribosomal protein | taxID used:135614 OG04140|WP\_010367666.1 | MULTISPECIES: YraN family protein [ | taxID used:135614 OG00414|WP\_010371293.1 | MULTISPECIES: excinuclease ABC subu | taxID used:135614 OG04151|WP\_010366191.1 | MULTISPECIES: arsenate reductase [X | taxID used:135614 OG04189|WP\_003484828.1 | MULTISPECIES: 50S ribosomal protein | taxID used:135614 OG04195|WP\_003486672.1 | MULTISPECIES: 30S ribosomal protein | taxID used:135614 OG04207|WP\_010367277.1 | MULTISPECIES: 50S ribosomal protein | taxID used:135614 OG04212|WP\_010371104.1 | MULTISPECIES: 50S ribosomal protein | taxID used:135614 OG04238|WP\_010372311.1 | MULTISPECIES: preprotein translocas | taxID used:135614 OG04260|WP\_010370143.1 | MULTISPECIES: thioredoxin TrxA [Xan | taxID used:135614 OG00428|WP\_010374597.1 | ATP-dependent DNA helicase Rep [Xan | taxID used:135614 OG04300|WP\_008571672.1 | MULTISPECIES: 30S ribosomal protein | taxID used:135614 OG04328|WP\_002811686.1 | MULTISPECIES: 50S ribosomal protein | taxID used:135614 OG04335|WP\_010371249.1 | MULTISPECIES: monothiol glutaredoxi | taxID used:135614 OG04373|WP\_005913896.1 | MULTISPECIES: YbaB/EbfC family nucl | taxID used:135614 OG04386|WP\_003484328.1 | MULTISPECIES: 50S ribosomal protein | taxID used:135614 OG04412|WP\_006450639.1 | MULTISPECIES: 50S ribosomal protein | taxID used:135614 OG04418|WP\_010373014.1 | MULTISPECIES: integration host fact | taxID used:135614 OG04435|WP\_010371092.1 | MULTISPECIES: 30S ribosomal protein | taxID used:135614 OG04464|WP\_010365692.1 | MULTISPECIES: ribosome assembly RNA | taxID used:135614 OG04469|WP\_002812428.1 | MULTISPECIES: DNA-directed RNA poly | taxID used:135614 OG04481|WP\_002811694.1 | MULTISPECIES: 50S ribosomal protein | taxID used:135614 OG04483|WP\_002811076.1 | MULTISPECIES: integration host fact | taxID used:135614 OG04502|WP\_010372004.1 | MULTISPECIES: F0F1 ATP synthase sub | taxID used:135614 OG04553|WP\_010363477.1 | MULTISPECIES: oxidative damage prot | taxID used:135614 OG04572|WP\_003483210.1 | MULTISPECIES: molecular chaperone G | taxID used:135614 OG04574|WP\_010368232.1 | MULTISPECIES: DUF493 domain-contain | taxID used:135614 OG00457|WP\_017116437.1 | MULTISPECIES: threonine--tRNA ligas | taxID used:135614 OG04583|WP\_005915027.1 | MULTISPECIES: RNA-binding protein H | taxID used:135614 OG04628|WP\_002804983.1 | MULTISPECIES: DNA-binding transcrip | taxID used:135614 OG04632|WP\_005993369.1 | MULTISPECIES: 30S ribosomal protein | taxID used:135614 OG04636|WP\_010371082.1 | MULTISPECIES: 30S ribosomal protein | taxID used:135614 OG04640|WP\_010374776.1 | MULTISPECIES: accessory factor UbiK | taxID used:135614 OG04660|WP\_010366347.1 | MULTISPECIES: RnfH family protein [ | taxID used:135614 OG04673|WP\_005991913.1 | MULTISPECIES: cell division protein | taxID used:135614 OG04682|WP\_003484323.1 | MULTISPECIES: 30S ribosomal protein | taxID used:135614 OG00470|WP\_010363501.1 | 1-deoxy-D-xylulose-5-phosphate synt | taxID used:135614 OG04712|WP\_010371226.1 | MULTISPECIES: 30S ribosomal protein | taxID used:135614 OG00471|WP\_010366902.1 | hypothetical protein, partial [Xant | taxID used:135614 OG04726|WP\_010365906.1 | MULTISPECIES: 30S ribosomal protein | taxID used:135614 OG04727|WP\_010365804.1 | MULTISPECIES: 50S ribosomal protein | taxID used:135614 OG00472|WP\_010367018.1 | ABC-F family ATP-binding cassette d | taxID used:135614 OG04745|WP\_010378796.1 | MULTISPECIES: succinate dehydrogena | taxID used:135614 OG04775|WP\_010364531.1 | MULTISPECIES: type B 50S ribosomal | taxID used:135614 OG00479|WP\_010368958.1 | MULTISPECIES: tRNA uridine-5-carbox | taxID used:135614 OG00047|WP\_010364931.1 | MULTISPECIES: exodeoxyribonuclease | taxID used:135614 OG00481|WP\_017120201.1 | transcription termination factor Rh | taxID used:135614 OG00483|WP\_010366362.1 | MULTISPECIES: molecular chaperone D | taxID used:135614 OG04840|WP\_002809459.1 | MULTISPECIES: 50S ribosomal protein | taxID used:135614 OG00484|WP\_010372379.1 | molecular chaperone HtpG, partial [ | taxID used:135614 OG04853|WP\_010368407.1 | MULTISPECIES: acyl carrier protein | taxID used:135614 OG04878|WP\_005991243.1 | MULTISPECIES: 30S ribosomal protein | taxID used:135614 OG00490|WP\_010365664.1 | MULTISPECIES: DNA topoisomerase IV | taxID used:135614 OG00494|WP\_010372732.1 | MULTISPECIES: ATP-binding cassette | taxID used:135614 OG04957|WP\_002813418.1 | MULTISPECIES: translation initiatio | taxID used:135614 OG00495|WP\_010369999.1 | MULTISPECIES: RNA polymerase sigma | taxID used:135614 OG00496|WP\_017116188.1 | DNA mismatch repair endonuclease Mu | taxID used:135614 OG00049|WP\_010369524.1 | transcription-repair coupling facto | taxID used:135614 OG05009|WP\_002808376.1 | MULTISPECIES: 30S ribosomal protein | taxID used:135614 OG00513|WP\_010373336.1 | excinuclease ABC subunit UvrC, part | taxID used:135614 OG05149|WP\_010368401.1 | MULTISPECIES: 50S ribosomal protein | taxID used:135614 OG00519|WP\_010369020.1 | dihydroxy-acid dehydratase, partial | taxID used:135614 OG05206|WP\_003486678.1 | MULTISPECIES: 50S ribosomal protein | taxID used:135614 OG05215|WP\_003486703.1 | MULTISPECIES: 50S ribosomal protein | taxID used:135614 OG05260|WP\_002809462.1 | MULTISPECIES: 50S ribosomal protein | taxID used:135614 OG00528|WP\_010368633.1 | MULTISPECIES: translational GTPase | taxID used:135614 OG00530|WP\_010371960.1 | MULTISPECIES: glutamine--fructose-6 | taxID used:135614 OG00547|WP\_010365963.1 | MULTISPECIES: elongation factor 4 [ | taxID used:135614 OG00554|WP\_026112789.1 | MULTISPECIES: outer membrane protei | taxID used:135614 OG00569|WP\_010363955.1 | MULTISPECIES: aspartate--tRNA ligas | taxID used:135614 OG00573|WP\_010367421.1 | MULTISPECIES: glutamine--tRNA ligas | taxID used:135614 OG00578|WP\_010374654.1 | DNA primase, partial [Xanthomonas v | taxID used:135614 OG00579|WP\_010364840.1 | membrane protein insertase YidC, pa | taxID used:135614 OG00601|WP\_010367100.1 | MULTISPECIES: single-stranded-DNA-s | taxID used:135614 OG00061|WP\_017115231.1 | carbamoyl-phosphate synthase large | taxID used:135614 OG00620|WP\_017115265.1 | ATP-dependent RNA helicase RhlB, pa | taxID used:135614 OG00630|WP\_010365592.1 | MULTISPECIES: proline--tRNA ligase | taxID used:135614 OG00641|WP\_003489652.1 | MULTISPECIES: 30S ribosomal protein | taxID used:135614 OG00648|WP\_010365666.1 | MULTISPECIES: CTP synthase [Xanthom | taxID used:135614 OG00650|WP\_010370534.1 | MULTISPECIES: ubiquinone biosynthes | taxID used:135614 OG00654|WP\_010367724.1 | MULTISPECIES: energy-dependent tran | taxID used:135614 OG00673|WP\_017116147.1 | MULTISPECIES: DNA repair protein Re | taxID used:135614 OG00677|WP\_043089358.1 | MULTISPECIES: ribosomal large subun | taxID used:135614 OG00689|WP\_010363469.1 | signal recognition particle-docking | taxID used:135614 OG00697|WP\_010368731.1 | peptide chain release factor 3, par | taxID used:135614 OG00699|WP\_010368157.1 | MULTISPECIES: chaperonin GroEL [Xan | taxID used:135614 OG00716|WP\_010365813.1 | MULTISPECIES: murein biosynthesis i | taxID used:135614 OG00733|WP\_010373041.1 | MULTISPECIES: glutamine-hydrolyzing | taxID used:135614 OG00741|WP\_010368122.1 | MULTISPECIES: bifunctional phosphor | taxID used:135614 OG00766|WP\_010371997.1 | MULTISPECIES: F0F1 ATP synthase sub | taxID used:135614 OG00767|WP\_010367085.1 | MULTISPECIES: lysine--tRNA ligase [ | taxID used:135614 OG00782|WP\_010371397.1 | MULTISPECIES: glucose-6-phosphate i | taxID used:135614 OG00796|WP\_010371209.1 | transcription termination/antitermi | taxID used:135614 OG00800|WP\_010366785.1 | MULTISPECIES: ribonuclease G [Xanth | taxID used:135614 OG00815|WP\_010367639.1 | MULTISPECIES: UDP-N-acetylmuramoyla | taxID used:135614 OG00819|WP\_010368572.1 | MULTISPECIES: amidophosphoribosyltr | taxID used:135614 OG00820|WP\_010367641.1 | MULTISPECIES: UDP-N-acetylmuramoyl- | taxID used:135614 OG00840|WP\_010372574.1 | MULTISPECIES: tRNA (N6-isopentenyl | taxID used:135614 OG00841|WP\_010367806.1 | leucyl aminopeptidase, partial [Xan | taxID used:135614 OG00855|WP\_010373039.1 | MULTISPECIES: IMP dehydrogenase [Xa | taxID used:135614 OG00857|WP\_017116206.1 | MULTISPECIES: cysteine--tRNA ligase | taxID used:135614 OG00865|WP\_010368669.1 | MULTISPECIES: adenosylmethionine--8 | taxID used:135614 OG00879|WP\_010367632.1 | MULTISPECIES: UDP-N-acetylmuramate- | taxID used:135614 OG00881|WP\_017113115.1 | MULTISPECIES: histidine--tRNA ligas | taxID used:135614 OG00893|WP\_010369556.1 | MULTISPECIES: glutamate--tRNA ligas | taxID used:135614 OG00897|WP\_010371991.1 | MULTISPECIES: F0F1 ATP synthase sub | taxID used:135614 OG00928|WP\_010373532.1 | MULTISPECIES: ribosome biogenesis G | taxID used:135614 OG00961|WP\_010365835.1 | DNA repair protein RadA, partial [X | taxID used:135614 OG00965|WP\_017116706.1 | MULTISPECIES: signal recognition pa | taxID used:135614 OG00967|WP\_026112498.1 | replication-associated recombinatio | taxID used:135614 OG00096|WP\_010365794.1 | excinuclease ABC subunit A, partial | taxID used:135614 OG00971|WP\_010367636.1 | MULTISPECIES: putative lipid II fli | taxID used:135614 OG00975|WP\_010371376.1 | polynucleotide adenylyltransferase | taxID used:135614 OG00995|WP\_010371982.1 | MULTISPECIES: UDP-N-acetylglucosami | taxID used:135614 OG00996|WP\_010366415.1 | adenylosuccinate lyase, partial [Xa | taxID used:135614 OG00997|WP\_010364426.1 | MULTISPECIES: UDP-N-acetylmuramate: | taxID used:135614 
OG03971|WP\_010366350.1 | MULTISPECIES: outer membrane protei | taxID used:135614 OG01000|WP\_003486674.1 | MULTISPECIES: preprotein translocas | taxID used:135614 OG01004|WP\_010371500.1 | MULTISPECIES: endonuclease/exonucle | taxID used:135614 OG01006|WP\_010366774.1 | MULTISPECIES: metalloprotease PmbA | taxID used:135614 OG01007|WP\_010370858.1 | DUF3999 domain-containing protein, | taxID used:135614 OG00100|WP\_010374562.1 | MULTISPECIES: TonB-dependent recept | taxID used:135614 OG01014|WP\_010365480.1 | MULTISPECIES: dicarboxylate/amino a | taxID used:135614 OG01015|WP\_010365621.1 | MULTISPECIES: phosphomannomutase/ph | taxID used:135614 OG01016|WP\_010364488.1 | MULTISPECIES: M24 family metallopep | taxID used:135614 OG01017|WP\_026112311.1 | MULTISPECIES: MFS transporter [Xant | taxID used:135614 OG01018|WP\_010374761.1 | glycosyltransferase, partial [Xanth | taxID used:135614 OG01019|WP\_010364492.1 | MULTISPECIES: Xaa-Pro dipeptidase [ | taxID used:135614 OG00101|WP\_010371599.1 | MULTISPECIES: glycine dehydrogenase | taxID used:135614 OG01020|WP\_010365977.1 | MULTISPECIES: 23S rRNA (uracil(1939 | taxID used:135614 OG01021|WP\_010376388.1 | MULTISPECIES: membrane protein [Xan | taxID used:135614 OG01022|WP\_054393858.1 | MULTISPECIES: DUF763 domain-contain | taxID used:135614 OG01023|WP\_010371171.1 | MULTISPECIES: NADH-quinone oxidored | taxID used:135614 OG01025|WP\_010368545.1 | MULTISPECIES: phosphate regulon sen | taxID used:135614 OG01029|WP\_026112300.1 | MULTISPECIES: NAD(P)/FAD-dependent | taxID used:135614 OG01030|WP\_010372847.1 | MULTISPECIES: acetylglutamate kinas | taxID used:135614 OG01033|WP\_010365507.1 | MULTISPECIES: MFS transporter [Xant | taxID used:135614 OG01035|WP\_010371673.1 | MULTISPECIES: lipase [Xanthomonas] | taxID used:135614 OG01036|WP\_010364810.1 | MULTISPECIES: chromosomal replicati | taxID used:135614 OG01038|WP\_010373410.1 | type VI secretion system baseplate | taxID used:135614 OG01040|WP\_010363803.1 | MULTISPECIES: HAMP domain-containin | taxID used:135614 OG01043|WP\_010363357.1 | MULTISPECIES: LLM class flavin-depe | taxID used:135614 OG01047|WP\_010366758.1 | dicarboxylate/amino acid:cation sym | taxID used:135614 OG01048|WP\_010373438.1 | MULTISPECIES: sorbosone dehydrogena | taxID used:135614 OG01049|WP\_010366656.1 | MULTISPECIES: phosphoglucosamine mu | taxID used:135614 OG01054|WP\_010364364.1 | MULTISPECIES: 3-deoxy-D-manno-octul | taxID used:135614 OG01055|WP\_010371977.1 | MULTISPECIES: sigma-54-dependent Fi | taxID used:135614 OG01059|WP\_010365503.1 | MULTISPECIES: TRAP transporter larg | taxID used:135614 OG01063|WP\_010372660.1 | MULTISPECIES: exodeoxyribonuclease | taxID used:135614 OG01066|WP\_026112809.1 | MULTISPECIES: MFS transporter [Xant | taxID used:135614 OG01067|WP\_010373211.1 | MULTISPECIES: divalent metal cation | taxID used:135614 OG00106|WP\_017116464.1 | MULTISPECIES: TonB-dependent recept | taxID used:135614 OG01071|WP\_017117349.1 | MULTISPECIES: hypothetical protein | taxID used:135614 OG01072|WP\_010365643.1 | MULTISPECIES: FAD-binding oxidoredu | taxID used:135614 OG01073|WP\_010366419.1 | MULTISPECIES: HlyC/CorC family tran | taxID used:135614 OG01074|WP\_026112865.1 | MULTISPECIES: tRNA lysidine(34) syn | taxID used:135614 OG01075|WP\_010372473.1 | MULTISPECIES: DUF445 domain-contain | taxID used:135614 OG01076|WP\_010364866.1 | MULTISPECIES: MFS transporter [Xant | taxID used:135614 OG01080|WP\_017115386.1 | ABC transporter permease, partial [ | taxID used:135614 OG01083|WP\_010371969.1 | MULTISPECIES: FtsX-like permease fa | taxID used:135614 OG01092|WP\_010364535.1 | MULTISPECIES: citrate synthase [Xan | taxID used:135614 OG01095|WP\_010365705.1 | MULTISPECIES: GTPase HflX [Xanthomo | taxID used:135614 OG01096|WP\_010366079.1 | MULTISPECIES: class I SAM-dependent | taxID used:135614 OG01097|WP\_010366457.1 | MULTISPECIES: L-fucose:H+ symporter | taxID used:135614 OG01098|WP\_010373409.1 | MULTISPECIES: type VI secretion sys | taxID used:135614 OG01100|WP\_010368616.1 | MULTISPECIES: membrane protein [Xan | taxID used:135614 OG01104|WP\_010374398.1 | MULTISPECIES: L-fucose:H+ symporter | taxID used:135614 OG01105|WP\_010373340.1 | nucleotide sugar dehydrogenase, par | taxID used:135614 OG01106|WP\_010372559.1 | MULTISPECIES: DUF4105 domain-contai | taxID used:135614 OG01107|WP\_010364452.1 | glutamate-1-semialdehyde 2,1-aminom | taxID used:135614 OG01108|WP\_010368527.1 | MULTISPECIES: trigger factor [Xanth | taxID used:135614 OG01110|WP\_010363761.1 | MULTISPECIES: amino acid permease [ | taxID used:135614 OG01112|WP\_010372121.1 | MULTISPECIES: D-amino acid dehydrog | taxID used:135614 OG01113|WP\_010368184.1 | MULTISPECIES: poly-beta-1,6 N-acety | taxID used:135614 OG01116|WP\_010368449.1 | MULTISPECIES: adenylosuccinate synt | taxID used:135614 OG01117|WP\_026112896.1 | diguanylate cyclase response regula | taxID used:135614 OG01118|WP\_010373633.1 | MULTISPECIES: flagellar protein [Xa | taxID used:135614 OG00111|WP\_010367825.1 | MULTISPECIES: valine--tRNA ligase [ | taxID used:135614 OG01120|WP\_010372592.1 | MULTISPECIES: cytochrome bc complex | taxID used:135614 OG01121|WP\_010367182.1 | threonine synthase, partial [Xantho | taxID used:135614 OG01122|WP\_010365994.1 | MULTISPECIES: FAD-dependent monooxy | taxID used:135614 OG01124|WP\_010374868.1 | MULTISPECIES: tryptophan--tRNA liga | taxID used:135614 OG01126|WP\_010370034.1 | MULTISPECIES: O-antigen ligase fami | taxID used:135614 OG01128|WP\_010368119.1 | phosphoribosylamine--glycine ligase | taxID used:135614 OG01129|WP\_010374708.1 | MULTISPECIES: MFS transporter [Xant | taxID used:135614 OG01131|WP\_010368578.1 | bifunctional tetrahydrofolate synth | taxID used:135614 OG01134|WP\_010374510.1 | MULTISPECIES: D-galactonate dehydra | taxID used:135614 OG01135|WP\_010371564.1 | MULTISPECIES: HAMP domain-containin | taxID used:135614 OG01136|WP\_010367329.1 | MULTISPECIES: YihY family inner mem | taxID used:135614 OG01140|WP\_010372855.1 | argininosuccinate lyase, partial [X | taxID used:135614 OG01142|WP\_010367319.1 | MULTISPECIES: glutamyl-tRNA reducta | taxID used:135614 OG01146|WP\_010365670.1 | phosphopyruvate hydratase [Xanthomo | taxID used:135614 OG01148|WP\_003483788.1 | MULTISPECIES: ATP-dependent Clp pro | taxID used:135614 OG00114|WP\_010365817.1 | MULTISPECIES: isoleucine--tRNA liga | taxID used:135614 OG01150|WP\_010367171.1 | MULTISPECIES: histidinol dehydrogen | taxID used:135614 OG01151|WP\_010364293.1 | MULTISPECIES: cardiolipin synthase | taxID used:135614 OG01156|WP\_010372931.1 | MULTISPECIES: SMC-Scp complex subun | taxID used:135614 OG01157|WP\_010363503.1 | hypothetical protein, partial [Xant | taxID used:135614 OG01158|WP\_010366744.1 | MULTISPECIES: FAD-dependent oxidore | taxID used:135614 OG01159|WP\_010372653.1 | MULTISPECIES: virulence factor [Xan | taxID used:135614 OG01162|WP\_010371421.1 | flavodoxin-dependent (E)-4-hydroxy- | taxID used:135614 OG01164|WP\_010365349.1 | MULTISPECIES: DUF3526 domain-contai | taxID used:135614 OG01166|WP\_010368190.1 | MULTISPECIES: glucose/galactose MFS | taxID used:135614 OG01168|WP\_010366884.1 | MULTISPECIES: potassium transporter | taxID used:135614 OG01169|WP\_010366703.1 | MULTISPECIES: membrane protein [Xan | taxID used:135614 OG01176|WP\_010373342.1 | MULTISPECIES: aminoacetone oxidase | taxID used:135614 OG01177|WP\_010371845.1 | ATP-binding cassette domain-contain | taxID used:135614 OG01182|WP\_010367709.1 | MULTISPECIES: serine hydroxymethylt | taxID used:135614 OG01184|WP\_010368565.1 | MULTISPECIES: peptidase [Xanthomona | taxID used:135614 OG01187|WP\_010374342.1 | MULTISPECIES: HlyD family efflux tr | taxID used:135614 OG01192|WP\_010366459.1 | MULTISPECIES: AGE family epimerase/ | taxID used:135614 OG01196|WP\_010364250.1 | MULTISPECIES: tetracycline resistan | taxID used:135614 OG01200|WP\_010364418.1 | MULTISPECIES: 6-phosphofructokinase | taxID used:135614 OG01201|WP\_010366747.1 | MULTISPECIES: DUF418 domain-contain | taxID used:135614 OG01203|WP\_010374604.1 | MULTISPECIES: GAF domain-containing | taxID used:135614 OG01204|WP\_010368409.1 | MULTISPECIES: beta-ketoacyl-[acyl-c | taxID used:135614 OG01205|WP\_017112838.1 | MULTISPECIES: putative DNA modifica | taxID used:135614 OG01216|WP\_010373234.1 | MULTISPECIES: class III poly(R)-hyd | taxID used:135614 OG01220|WP\_010378794.1 | MULTISPECIES: lipoprotein-releasing | taxID used:135614 OG01222|WP\_010363732.1 | efflux RND transporter periplasmic | taxID used:135614 OG01223|WP\_010367324.1 | hypothetical protein, partial [Xant | taxID used:135614 OG01225|WP\_003485272.1 | MULTISPECIES: cell division protein | taxID used:135614 OG01226|WP\_010365595.1 | MULTISPECIES: threonine/serine expo | taxID used:135614 OG01227|WP\_010365008.1 | MULTISPECIES: chemotaxis protein Ch | taxID used:135614 OG01233|WP\_017117441.1 | MULTISPECIES: argininosuccinate syn | taxID used:135614 OG01235|WP\_010367620.1 | MULTISPECIES: cell division protein | taxID used:135614 OG01237|WP\_010373598.1 | sensor histidine kinase, partial [X | taxID used:135614 OG01238|WP\_010371974.1 | MULTISPECIES: FtsX-like permease fa | taxID used:135614 OG01242|WP\_010367140.1 | MULTISPECIES: phosphoglycerate dehy | taxID used:135614 OG01243|WP\_010368176.1 | MULTISPECIES: multifunctional CCA a | taxID used:135614 OG01248|WP\_010368265.1 | MULTISPECIES: phosphonopyruvate dec | taxID used:135614 OG01250|WP\_010373246.1 | MULTISPECIES: FAD-binding oxidoredu | taxID used:135614 OG01252|WP\_010373542.1 | MULTISPECIES: 23S rRNA (adenine(250 | taxID used:135614 OG01253|WP\_010376381.1 | MULTISPECIES: peptidase M23 [Xantho | taxID used:135614 OG01254|WP\_010363526.1 | MULTISPECIES: glycosyltransferase f | taxID used:135614 OG01255|WP\_010368235.1 | MULTISPECIES: D-alanyl-D-alanine ca | taxID used:135614 OG01257|WP\_010374848.1 | MULTISPECIES: efflux RND transporte | taxID used:135614 OG01258|WP\_017116375.1 | MULTISPECIES: aspartate aminotransf | taxID used:135614 OG01259|WP\_010372869.1 | MULTISPECIES: glutamate-5-semialdeh | taxID used:135614 OG00125|WP\_010367562.1 | TonB-dependent receptor, partial [X | taxID used:135614 OG01260|WP\_010365414.1 | FAD-binding oxidoreductase, partial | taxID used:135614 OG01262|WP\_010367557.1 | MULTISPECIES: methionine adenosyltr | taxID used:135614 OG01263|WP\_010372969.1 | MULTISPECIES: TraB/GumN family prot | taxID used:135614 OG01264|WP\_010366666.1 | tryptophan synthase subunit beta, p | taxID used:135614 OG01265|WP\_010363442.1 | MULTISPECIES: aminopeptidase P fami | taxID used:135614 OG01268|WP\_010364434.1 | MULTISPECIES: aspartate aminotransf | taxID used:135614 OG01270|WP\_010373528.1 | MULTISPECIES: molybdopterin molybde | taxID used:135614 OG01271|WP\_010367044.1 | enoyl-[acyl-carrier-protein] reduct | taxID used:135614 OG01272|WP\_010372890.1 | MULTISPECIES: homoserine O-acetyltr | taxID used:135614 OG01277|WP\_010365501.1 | MULTISPECIES: porin [Xanthomonas] [ | taxID used:135614 OG00127|WP\_010374443.1 | MULTISPECIES: TonB-dependent recept | taxID used:135614 OG01280|WP\_010374689.1 | MULTISPECIES: tyrosine--tRNA ligase | taxID used:135614 OG01281|WP\_010368601.1 | MULTISPECIES: glycine C-acetyltrans | taxID used:135614 OG01286|WP\_017118858.1 | MULTISPECIES: pyridoxal phosphate-d | taxID used:135614 OG01287|WP\_010366882.1 | MULTISPECIES: HAMP domain-containin | taxID used:135614 OG01292|WP\_010366187.1 | MULTISPECIES: 2,3,4,5-tetrahydropyr | taxID used:135614 OG00129|WP\_010373588.1 | MULTISPECIES: GGDEF domain-containi | taxID used:135614 OG01300|WP\_010367736.1 | MULTISPECIES: nicotinate phosphorib | taxID used:135614 OG01302|WP\_010373627.1 | MULTISPECIES: flagellar hook-associ | taxID used:135614 OG01305|WP\_010367780.1 | MULTISPECIES: type II secretion sys | taxID used:135614 OG01306|WP\_010371857.1 | MULTISPECIES: cystathionine gamma-s | taxID used:135614 OG01313|WP\_010371506.1 | MULTISPECIES: formate-dependent pho | taxID used:135614 OG01314|WP\_010371541.1 | MULTISPECIES: sensor histidine kina | taxID used:135614 OG01315|WP\_010368426.1 | 2-methylaconitate cis-trans isomera | taxID used:135614 OG01318|WP\_010367069.1 | MULTISPECIES: aspartate/tyrosine/ar | taxID used:135614 OG01322|WP\_010367455.1 | MULTISPECIES: 2-octaprenyl-6-methox | taxID used:135614 OG01323|WP\_010370903.1 | MULTISPECIES: sensor histidine kina | taxID used:135614 OG01324|WP\_033004672.1 | MULTISPECIES: chemotaxis protein [X | taxID used:135614 OG01326|WP\_010373507.1 | MULTISPECIES: glutathione-dependent | taxID used:135614 OG01328|WP\_010365939.1 | MULTISPECIES: acyl-CoA dehydrogenas | taxID used:135614 OG01332|WP\_010364859.1 | MULTISPECIES: class I SAM-dependent | taxID used:135614 OG01333|WP\_010365482.1 | MULTISPECIES: porin [Xanthomonas] [ | taxID used:135614 OG01334|WP\_010374907.1 | MULTISPECIES: DUF1501 domain-contai | taxID used:135614 OG01335|WP\_010364796.1 | MULTISPECIES: tetratricopeptide rep | taxID used:135614 OG01337|WP\_010373020.1 | MULTISPECIES: lipopolysaccharide as | taxID used:135614 OG01338|WP\_010366225.1 | MULTISPECIES: HlyD family efflux tr | taxID used:135614 OG01339|WP\_010368421.1 | MULTISPECIES: 2-methylcitrate synth | taxID used:135614 OG01341|WP\_026112535.1 | MULTISPECIES: hypothetical protein | taxID used:135614 OG01342|WP\_010364700.1 | MULTISPECIES: DNA topoisomerase IB | taxID used:135614 OG01343|WP\_010371534.1 | MULTISPECIES: polyketide cyclase [X | taxID used:135614 OG01346|WP\_010373665.1 | aromatic ring-hydroxylating dioxyge | taxID used:135614 OG01347|WP\_010368204.1 | MULTISPECIES: class I SAM-dependent | taxID used:135614 OG01348|WP\_010363769.1 | MULTISPECIES: PQQ-dependent sugar d | taxID used:135614 OG00134|WP\_010367615.1 | MULTISPECIES: preprotein translocas | taxID used:135614 OG01350|WP\_010365379.1 | MULTISPECIES: UDP-glucose 4-epimera | taxID used:135614 OG01352|WP\_010365943.1 | MULTISPECIES: enoyl-CoA hydratase/i | taxID used:135614 OG01357|WP\_017112297.1 | MULTISPECIES: sensor histidine kina | taxID used:135614 OG01363|WP\_010373629.1 | MULTISPECIES: flagellin [Xanthomona | taxID used:135614 OG01366|WP\_010366046.1 | MULTISPECIES: alkene reductase [Xan | taxID used:135614 OG01367|WP\_010374662.1 | MULTISPECIES: heme A synthase [Xant | taxID used:135614 OG01370|WP\_010371312.1 | MULTISPECIES: TrbI/VirB10 family pr | taxID used:135614 OG01372|WP\_026112737.1 | MULTISPECIES: LacI family DNA-bindi | taxID used:135614 OG01373|WP\_010367453.1 | 2-octaprenyl-3-methyl-6-methoxy-1,4 | taxID used:135614 OG01376|WP\_010368113.1 | MULTISPECIES: MFS transporter [Xant | taxID used:135614 OG01378|WP\_010366014.1 | MULTISPECIES: acetyl-CoA C-acyltran | taxID used:135614 OG00137|WP\_017117099.1 | MULTISPECIES: polysaccharide biosyn | taxID used:135614 OG01388|WP\_010367057.1 | MULTISPECIES: alpha-hydroxy-acid ox | taxID used:135614 OG01389|WP\_010366204.1 | MULTISPECIES: ADP-forming succinate | taxID used:135614 OG01391|WP\_010365884.1 | MULTISPECIES: PAS domain-containing | taxID used:135614 OG01392|WP\_010374852.1 | MULTISPECIES: ABC transporter perme | taxID used:135614 OG01393|WP\_017116067.1 | MULTISPECIES: N-acetyltransferase [ | taxID used:135614 OG01394|WP\_010363508.1 | ceramide glucosyltransferase [Xanth | taxID used:135614 OG00139|WP\_010364582.1 | MULTISPECIES: TonB-dependent recept | taxID used:135614 OG01401|WP\_010370862.1 | MULTISPECIES: phosphoglycerate kina | taxID used:135614 OG01403|WP\_010363532.1 | MULTISPECIES: glycosyltransferase f | taxID used:135614 OG01404|WP\_010368238.1 | MULTISPECIES: lytic murein transgly | taxID used:135614 OG01405|WP\_082337890.1 | molybdopterin-synthase adenylyltran | taxID used:135614 OG01406|WP\_010373581.1 | tRNA 2-thiouridine(34) synthase Mnm | taxID used:135614 OG01407|WP\_010372148.1 | MULTISPECIES: motility protein MotB | taxID used:135614 OG01408|WP\_010374787.1 | MULTISPECIES: acyl-CoA desaturase [ | taxID used:135614 OG01411|WP\_010372544.1 | MULTISPECIES: polyamine ABC transpo | taxID used:135614 OG01412|WP\_010371893.1 | type IV pilus twitching motility pr | taxID used:135614 OG01414|WP\_010367083.1 | MULTISPECIES: two-component system | taxID used:135614 OG01416|WP\_010367166.1 | MULTISPECIES: bifunctional histidin | taxID used:135614 OG01418|WP\_010368187.1 | MULTISPECIES: N-acetylglucosamine-6 | taxID used:135614 OG01420|WP\_010370048.1 | MULTISPECIES: LysM peptidoglycan-bi | taxID used:135614 OG01422|WP\_010366364.1 | MULTISPECIES: molecular chaperone D | taxID used:135614 OG01423|WP\_010368556.1 | MULTISPECIES: glycosyltransferase f | taxID used:135614 OG00142|WP\_010371657.1 | MULTISPECIES: pyruvate dehydrogenas | taxID used:135614 OG01431|WP\_010367112.1 | MULTISPECIES: carbamoyl-phosphate s | taxID used:135614 OG01439|WP\_010367699.1 | 3,4-dihydroxy-2-butanone-4-phosphat | taxID used:135614 OG01447|WP\_010374356.1 | ROK family transcriptional regulato | taxID used:135614 OG01448|WP\_010365874.1 | MULTISPECIES: hybrid sensor histidi | taxID used:135614 OG01450|WP\_010366193.1 | MULTISPECIES: succinyl-diaminopimel | taxID used:135614 OG01451|WP\_010368253.1 | MULTISPECIES: rod shape-determining | taxID used:135614 OG01452|WP\_026112542.1 | MULTISPECIES: HDOD domain-containin | taxID used:135614 OG01453|WP\_010367431.1 | MULTISPECIES: gfo/Idh/MocA family o | taxID used:135614 OG01454|WP\_010373187.1 | MULTISPECIES: HAMP domain-containin | taxID used:135614 OG01456|WP\_010372866.1 | MULTISPECIES: glutamate 5-kinase [X | taxID used:135614 OG01459|WP\_054393955.1 | MULTISPECIES: FtsH protease activit | taxID used:135614 OG00145|WP\_010374514.1 | glucan 1,4-alpha-glucosidase [Xanth | taxID used:135614 OG01461|WP\_010366368.1 | MULTISPECIES: prephenate dehydrogen | taxID used:135614 OG01462|WP\_010371145.1 | mechanosensitive ion channel family | taxID used:135614 OG01463|WP\_010374856.1 | MULTISPECIES: alpha/beta fold hydro | taxID used:135614 OG01464|WP\_010365569.1 | MULTISPECIES: S-(hydroxymethyl)glut | taxID used:135614 OG01465|WP\_010372337.1 | MULTISPECIES: phytase [Xanthomonas] | taxID used:135614 OG01467|WP\_010372531.1 | MULTISPECIES: polyamine ABC transpo | taxID used:135614 OG01468|WP\_010373650.1 | MULTISPECIES: DegT/DnrJ/EryC1/StrS | taxID used:135614 OG01473|WP\_010371935.1 | MULTISPECIES: PLP-dependent cystein | taxID used:135614 OG01475|WP\_010374699.1 | MULTISPECIES: anhydro-N-acetylmuram | taxID used:135614 OG01476|WP\_017115450.1 | MULTISPECIES: hypothetical protein | taxID used:135614 OG00147|WP\_054393990.1 | translation initiation factor IF-2, | taxID used:135614 OG01481|WP\_010367553.1 | metal-dependent hydrolase, partial | taxID used:135614 OG01484|WP\_010370020.1 | GTP cyclohydrolase II RibA, partial | taxID used:135614 OG01487|WP\_010371184.1 | MULTISPECIES: NADH-quinone oxidored | taxID used:135614 OG01489|WP\_010365573.1 | MULTISPECIES: glycosyl transferase | taxID used:135614 OG01490|WP\_010373173.1 | MULTISPECIES: YeiH family putative | taxID used:135614 OG01495|WP\_010367803.1 | MULTISPECIES: LPS export ABC transp | taxID used:135614 OG01500|WP\_010366687.1 | chorismate synthase, partial [Xanth | taxID used:135614 OG01503|WP\_010368597.1 | MULTISPECIES: sulfate ABC transport | taxID used:135614 OG01508|WP\_010364390.1 | MULTISPECIES: threonine/serine dehy | taxID used:135614 OG01510|WP\_010368610.1 | MULTISPECIES: OmpA family protein [ | taxID used:135614 OG01512|WP\_010364805.1 | MULTISPECIES: DNA replication/repai | taxID used:135614 OG01515|WP\_010372118.1 | MULTISPECIES: alanine racemase [Xan | taxID used:135614 OG01516|WP\_010375294.1 | MULTISPECIES: aliphatic sulfonate A | taxID used:135614 OG01520|WP\_010366071.1 | MULTISPECIES: class I SAM-dependent | taxID used:135614 OG01524|WP\_010376411.1 | MULTISPECIES: glycerophosphoryl die | taxID used:135614 OG01525|WP\_010365597.1 | MULTISPECIES: ABC transporter perme | taxID used:135614 OG01526|WP\_010372844.1 | MULTISPECIES: acetylornithine deace | taxID used:135614 OG01527|WP\_010364355.1 | hypothetical protein, partial [Xant | taxID used:135614 OG01528|WP\_054394006.1 | MULTISPECIES: AI-2E family transpor | taxID used:135614 OG01534|WP\_010373300.1 | MULTISPECIES: sn-glycerol-3-phospha | taxID used:135614 OG01536|WP\_010374790.1 | MULTISPECIES: ferredoxin reductase | taxID used:135614 OG01537|WP\_010365516.1 | MULTISPECIES: ribosome small subuni | taxID used:135614 OG01539|WP\_010363474.1 | MULTISPECIES: A/G-specific adenine | taxID used:135614 OG01543|WP\_010363755.1 | MULTISPECIES: dipeptide epimerase [ | taxID used:135614 OG01544|WP\_010372663.1 | MULTISPECIES: tRNA epoxyqueuosine(3 | taxID used:135614 OG01546|WP\_010367638.1 | MULTISPECIES: phospho-N-acetylmuram | taxID used:135614 OG01547|WP\_010366489.1 | MULTISPECIES: phosphate ABC transpo | taxID used:135614 OG00154|WP\_010373519.1 | MULTISPECIES: TonB-dependent recept | taxID used:135614 OG01550|WP\_010373324.1 | MULTISPECIES: NAD-dependent epimera | taxID used:135614 OG01553|WP\_010366378.1 | MULTISPECIES: HlyD family efflux tr | taxID used:135614 OG01554|WP\_017115586.1 | NAD(P)(+) transhydrogenase (Re/Si-s | taxID used:135614 OG01558|WP\_010373238.1 | MULTISPECIES: class III poly(R)-hyd | taxID used:135614 OG01560|WP\_010364715.1 | MULTISPECIES: glycoside hydrolase f | taxID used:135614 OG01564|WP\_010368283.1 | MULTISPECIES: HlyD family secretion | taxID used:135614 OG01568|WP\_010363342.1 | MULTISPECIES: DUF4432 domain-contai | taxID used:135614 OG01570|WP\_010371921.1 | DNA polymerase IV, partial [Xanthom | taxID used:135614 OG01575|WP\_010367805.1 | MULTISPECIES: LPS export ABC transp | taxID used:135614 OG01577|WP\_010375808.1 | MULTISPECIES: P-type DNA transfer A | taxID used:135614 OG01578|WP\_010367169.1 | MULTISPECIES: histidinol-phosphate | taxID used:135614 OG01579|WP\_010364711.1 | MULTISPECIES: NAD(P)-dependent alco | taxID used:135614 OG01580|WP\_010371942.1 | MULTISPECIES: copper resistance pro | taxID used:135614 OG01582|WP\_010363534.1 | polysaccharide biosynthesis protein | taxID used:135614 OG01583|WP\_017116184.1 | MULTISPECIES: sensor domain-contain | taxID used:135614 OG01584|WP\_026112077.1 | MULTISPECIES: hypothetical protein | taxID used:135614 OG01587|WP\_010371818.1 | MULTISPECIES: 3-deoxy-7-phosphohept | taxID used:135614 OG01589|WP\_010367154.1 | MULTISPECIES: hypothetical protein | taxID used:135614 OG01590|WP\_010367296.1 | MULTISPECIES: redox-regulated ATPas | taxID used:135614 OG01593|WP\_010364723.1 | MULTISPECIES: glycoside hydrolase f | taxID used:135614 OG01598|WP\_010366196.1 | MULTISPECIES: right-handed parallel | taxID used:135614 OG00159|WP\_010374571.1 | glycerol-3-phosphate 1-O-acyltransf | taxID used:135614 OG01601|WP\_010365494.1 | MULTISPECIES: aldo/keto reductase [ | taxID used:135614 OG01607|WP\_010368225.1 | MULTISPECIES: bifunctional nicotina | taxID used:135614 OG01608|WP\_010368214.1 | MULTISPECIES: oxidoreductase [Xanth | taxID used:135614 OG01611|WP\_010368412.1 | endolytic transglycosylase MltG, pa | taxID used:135614 OG01613|WP\_010374564.1 | MULTISPECIES: alpha-N-arabinofurano | taxID used:135614 OG01616|WP\_010364905.1 | MULTISPECIES: VacJ family lipoprote | taxID used:135614 OG01621|WP\_054393996.1 | MULTISPECIES: arabinogalactan endo- | taxID used:135614 OG01622|WP\_010365631.1 | MULTISPECIES: dTDP-glucose 4,6-dehy | taxID used:135614 OG01628|WP\_010366458.1 | MULTISPECIES: carbohydrate kinase [ | taxID used:135614 OG01629|WP\_010366927.1 | MULTISPECIES: alcohol dehydrogenase | taxID used:135614 OG01631|WP\_010363292.1 | MULTISPECIES: lipopolysaccharide he | taxID used:135614 OG01632|WP\_010377901.1 | MULTISPECIES: LacI family DNA-bindi | taxID used:135614 OG01633|WP\_010371434.1 | MULTISPECIES: UDP-N-acetylmuramate | taxID used:135614 OG01636|WP\_010363530.1 | MULTISPECIES: GDP-mannose--glycolip | taxID used:135614 OG01637|WP\_010369593.1 | MULTISPECIES: sulfotransferase [Xan | taxID used:135614 OG01638|WP\_017116281.1 | MULTISPECIES: type IV secretion sys | taxID used:135614 OG01639|WP\_010370932.1 | MULTISPECIES: MexH family multidrug | taxID used:135614 OG01642|WP\_010372979.1 | MULTISPECIES: agmatine deiminase fa | taxID used:135614 OG01644|WP\_010368057.1 | MULTISPECIES: aminotransferase clas | taxID used:135614 OG01646|WP\_010365815.1 | bifunctional riboflavin kinase/FAD | taxID used:135614 OG01648|WP\_010374576.1 | MULTISPECIES: LacI family transcrip | taxID used:135614 OG01650|WP\_010372838.1 | MULTISPECIES: N-acetylornithine car | taxID used:135614 OG01652|WP\_010365894.1 | MULTISPECIES: galactose mutarotase | taxID used:135614 OG01656|WP\_010367649.1 | MULTISPECIES: 16S rRNA (cytosine(14 | taxID used:135614 OG01658|WP\_010370971.1 | MULTISPECIES: LacI family DNA-bindi | taxID used:135614 OG01661|WP\_010367517.1 | MULTISPECIES: ABC transporter subst | taxID used:135614 OG01664|WP\_010374656.1 | MULTISPECIES: bile acid:sodium symp | taxID used:135614 OG01666|WP\_010365805.1 | MULTISPECIES: GTPase ObgE [Xanthomo | taxID used:135614 OG01667|WP\_010366003.1 | MULTISPECIES: ligase-associated DNA | taxID used:135614 OG00166|WP\_026112758.1 | MULTISPECIES: DUF802 domain-contain | taxID used:135614 OG01672|WP\_010373330.1 | MULTISPECIES: tetraacyldisaccharide | taxID used:135614 OG01676|WP\_010366041.1 | MULTISPECIES: cation transporter [X | taxID used:135614 OG01682|WP\_010366358.1 | MULTISPECIES: heat-inducible transc | taxID used:135614 OG01695|WP\_010368064.1 | MULTISPECIES: anthranilate phosphor | taxID used:135614 OG01696|WP\_010368229.1 | MULTISPECIES: lipoyl synthase [Xant | taxID used:135614 OG01697|WP\_010366310.1 | MULTISPECIES: LysR family transcrip | taxID used:135614 OG01698|WP\_010365384.1 | MULTISPECIES: cytochrome d ubiquino | taxID used:135614 OG01704|WP\_010368612.1 | MULTISPECIES: zinc-binding alcohol | taxID used:135614 OG01711|WP\_017117506.1 | acyl-CoA desaturase, partial [Xanth | taxID used:135614 OG01712|WP\_017117276.1 | hypothetical protein, partial [Xant | taxID used:135614 OG01715|WP\_010368591.1 | MULTISPECIES: sulfate/molybdate ABC | taxID used:135614 OG01716|WP\_017118524.1 | MULTISPECIES: glycerophosphodiester | taxID used:135614 OG01718|WP\_010365715.1 | MULTISPECIES: recombinase RecA [Xan | taxID used:135614 OG01719|WP\_010365505.1 | MULTISPECIES: LacI family transcrip | taxID used:135614 OG01722|WP\_010364560.1 | MULTISPECIES: VWA domain-containing | taxID used:135614 OG01731|WP\_010364623.1 | MULTISPECIES: class 1 fructose-bisp | taxID used:135614 OG01732|WP\_010366490.1 | MULTISPECIES: phosphate ABC transpo | taxID used:135614 OG01734|WP\_010374916.1 | MULTISPECIES: tRNA dihydrouridine(2 | taxID used:135614 OG01735|WP\_010373744.1 | MULTISPECIES: flagellar motor switc | taxID used:135614 OG01740|WP\_010366823.1 | MULTISPECIES: DNA polymerase III su | taxID used:135614 OG01744|WP\_010372556.1 | MULTISPECIES: magnesium and cobalt | taxID used:135614 OG01754|WP\_010364554.1 | MULTISPECIES: MoxR family ATPase [X | taxID used:135614 OG00175|WP\_010365486.1 | MULTISPECIES: hybrid sensor histidi | taxID used:135614 OG01762|WP\_010370873.1 | MULTISPECIES: fructose-bisphosphate | taxID used:135614 OG01766|WP\_010372078.1 | MULTISPECIES: alpha/beta fold hydro | taxID used:135614 OG00176|WP\_017116096.1 | 1,4-beta-D-glucan glucohydrolase, p | taxID used:135614 OG01773|WP\_010366151.1 | UDP-3-O-(3-hydroxymyristoyl)glucosa | taxID used:135614 OG01777|WP\_010365988.1 | MULTISPECIES: beta-N-acetylhexosami | taxID used:135614 OG01778|WP\_010372891.1 | tetratricopeptide repeat protein, p | taxID used:135614 OG01779|WP\_010364395.1 | MULTISPECIES: ketol-acid reductoiso | taxID used:135614 OG01780|WP\_010363591.1 | MULTISPECIES: phenylalanine--tRNA l | taxID used:135614 OG01781|WP\_003490161.1 | MULTISPECIES: type I glyceraldehyde | taxID used:135614 OG01795|WP\_010373388.1 | MULTISPECIES: cytochrome-c peroxida | taxID used:135614 OG01797|WP\_010368534.1 | MULTISPECIES: NAD-dependent isocitr | taxID used:135614 OG01811|WP\_010366544.1 | MULTISPECIES: aldo/keto reductase [ | taxID used:135614 OG01812|WP\_010366081.1 | DUF1365 domain-containing protein, | taxID used:135614 OG01814|WP\_010367373.1 | MULTISPECIES: exodeoxyribonuclease | taxID used:135614 OG01816|WP\_010372468.1 | MULTISPECIES: two-component system | taxID used:135614 OG01819|WP\_010366210.1 | MULTISPECIES: 23S rRNA pseudouridin | taxID used:135614 OG01820|WP\_010378578.1 | MULTISPECIES: glycoside hydrolase f | taxID used:135614 OG01821|WP\_010371899.1 | fumarylacetoacetate hydrolase, part | taxID used:135614 OG01822|WP\_010366840.1 | MULTISPECIES: peptidylprolyl isomer | taxID used:135614 OG01827|WP\_002811635.1 | MULTISPECIES: DNA-directed RNA poly | taxID used:135614 OG01828|WP\_010366429.1 | MULTISPECIES: hypothetical protein | taxID used:135614 OG01834|WP\_010366417.1 | CAP domain-containing protein, part | taxID used:135614 OG01838|WP\_010364737.1 | MULTISPECIES: D-2-hydroxyacid dehyd | taxID used:135614 OG00183|WP\_010368424.1 | MULTISPECIES: Fe/S-dependent 2-meth | taxID used:135614 OG01840|WP\_010363199.1 | MULTISPECIES: helix-turn-helix doma | taxID used:135614 OG01846|WP\_010364573.1 | MULTISPECIES: LysR family transcrip | taxID used:135614 OG01847|WP\_010365731.1 | MULTISPECIES: membrane protein [Xan | taxID used:135614 OG01853|WP\_010373023.1 | MULTISPECIES: glycosyltransferase f | taxID used:135614 OG01854|WP\_010374727.1 | MULTISPECIES: NAD-dependent epimera | taxID used:135614 OG01856|WP\_010373215.1 | MULTISPECIES: mechanosensitive ion | taxID used:135614 OG01857|WP\_010365699.1 | MULTISPECIES: tRNA (adenosine(37)-N | taxID used:135614 OG01858|WP\_017115996.1 | MULTISPECIES: ferrochelatase [Xanth | taxID used:135614 OG01861|WP\_010363163.1 | MULTISPECIES: KR domain-containing | taxID used:135614 OG01863|WP\_010370892.1 | MULTISPECIES: LysR family transcrip | taxID used:135614 OG01864|WP\_010373361.1 | 23S rRNA pseudouridine(955/2504/258 | taxID used:135614 OG01865|WP\_026112883.1 | MULTISPECIES: LysR family transcrip | taxID used:135614 OG01868|WP\_010367537.1 | MULTISPECIES: aldo/keto reductase [ | taxID used:135614 OG01869|WP\_010367738.1 | MULTISPECIES: hypothetical protein | taxID used:135614 OG01871|WP\_003482910.1 | MULTISPECIES: thioredoxin-disulfide | taxID used:135614 OG01875|WP\_010367471.1 | MULTISPECIES: symmetrical bis(5'-nu | taxID used:135614 OG01876|WP\_026112517.1 | MULTISPECIES: FKBP-type peptidyl-pr | taxID used:135614 OG01878|WP\_026112080.1 | MULTISPECIES: alcohol dehydrogenase | taxID used:135614 OG01880|WP\_017116725.1 | MULTISPECIES: cation transporter [X | taxID used:135614 OG01881|WP\_010368402.1 | MULTISPECIES: ketoacyl-ACP synthase | taxID used:135614 OG01882|WP\_010373680.1 | MULTISPECIES: flagellar motor switc | taxID used:135614 OG01883|WP\_010366113.1 | MULTISPECIES: homocysteine S-methyl | taxID used:135614 OG01888|WP\_010367603.1 | MULTISPECIES: NAD(P)H quinone oxido | taxID used:135614 OG01890|WP\_010367378.1 | MULTISPECIES: prolyl aminopeptidase | taxID used:135614 OG01891|WP\_010366487.1 | MULTISPECIES: phosphate ABC transpo | taxID used:135614 OG01893|WP\_010363510.1 | MULTISPECIES: NAD(P)-dependent oxid | taxID used:135614 OG01895|WP\_010366325.1 | MULTISPECIES: GGDEF domain-containi | taxID used:135614 OG01896|WP\_054393860.1 | MULTISPECIES: alpha/beta hydrolase | taxID used:135614 OG00189|WP\_017116259.1 | TonB-dependent receptor, partial [X | taxID used:135614 OG01901|WP\_010364857.1 | TerC/Alx family metal homeostasis m | taxID used:135614 OG01902|WP\_010372305.1 | MULTISPECIES: protein translocase s | taxID used:135614 OG01903|WP\_010367467.1 | 4-hydroxythreonine-4-phosphate dehy | taxID used:135614 OG01909|WP\_010365646.1 | anchor NAD-dependent epimerase/dehy | taxID used:135614 OG01911|WP\_033009780.1 | MULTISPECIES: 5'-nucleotidase, lipo | taxID used:135614 OG01912|WP\_010366595.1 | MULTISPECIES: EF-P lysine aminoacyl | taxID used:135614 OG01913|WP\_010367059.1 | MULTISPECIES: Ku protein [Xanthomon | taxID used:135614 OG01916|WP\_010366512.1 | MULTISPECIES: DUF1684 domain-contai | taxID used:135614 OG01918|WP\_010372851.1 | MULTISPECIES: N-acetyl-gamma-glutam | taxID used:135614 OG01920|WP\_010366538.1 | MULTISPECIES: 5'-nucleotidase [Xant | taxID used:135614 OG01921|WP\_003482735.1 | MULTISPECIES: carbohydrate kinase f | taxID used:135614 OG01922|WP\_017115626.1 | MULTISPECIES: sulfotransferase doma | taxID used:135614 OG01923|WP\_010366140.1 | MULTISPECIES: acetyl-CoA carboxylas | taxID used:135614 OG01925|WP\_010366654.1 | MULTISPECIES: isopenicillin N synth | taxID used:135614 OG01927|WP\_010365147.1 | MULTISPECIES: DUF58 domain-containi | taxID used:135614 OG01928|WP\_010367308.1 | MULTISPECIES: ribose-phosphate pyro | taxID used:135614 OG01929|WP\_010368593.1 | MULTISPECIES: sulfate ABC transport | taxID used:135614 OG01931|WP\_010368413.1 | MULTISPECIES: DNA polymerase III su | taxID used:135614 OG01934|WP\_010372425.1 | MULTISPECIES: LysR family transcrip | taxID used:135614 OG01935|WP\_017118495.1 | MULTISPECIES: hypothetical protein | taxID used:135614 OG01936|WP\_010370887.1 | MULTISPECIES: cysteine synthase A [ | taxID used:135614 OG01938|WP\_010365823.1 | 4-hydroxy-3-methylbut-2-enyl diphos | taxID used:135614 OG01940|WP\_010364362.1 | MULTISPECIES: LpxL/LpxP family Kdo( | taxID used:135614 OG01942|WP\_010374652.1 | MULTISPECIES: YihY/virulence factor | taxID used:135614 OG01943|WP\_010367629.1 | MULTISPECIES: D-alanine--D-alanine | taxID used:135614 OG01946|WP\_010372683.1 | MULTISPECIES: DUF1684 domain-contai | taxID used:135614 OG01951|WP\_017115932.1 | MULTISPECIES: glycerophosphodiester | taxID used:135614 OG01952|WP\_010368216.1 | MULTISPECIES: 6-phosphogluconate de | taxID used:135614 OG01953|WP\_010363456.1 | MULTISPECIES: hydroxyproline-2-epim | taxID used:135614 OG01954|WP\_010378548.1 | MULTISPECIES: homoserine kinase [Xa | taxID used:135614 OG01956|WP\_017122563.1 | MULTISPECIES: phosphoribosylaminoim | taxID used:135614 OG01957|WP\_010367593.1 | MULTISPECIES: gfo/Idh/MocA family o | taxID used:135614 OG01960|WP\_017117312.1 | MULTISPECIES: arginyltransferase [X | taxID used:135614 OG01964|WP\_010364529.1 | MULTISPECIES: inosine-uridine prefe | taxID used:135614 OG01965|WP\_010367233.1 | MULTISPECIES: ABC transporter ATP-b | taxID used:135614 OG01966|WP\_010367395.1 | MULTISPECIES: LysR family transcrip | taxID used:135614 OG01974|WP\_017112362.1 | MULTISPECIES: phosphate/phosphite/p | taxID used:135614 OG01975|WP\_010373602.1 | MULTISPECIES: chemotaxis protein Ch | taxID used:135614 OG01976|WP\_010373479.1 | DUF2817 domain-containing protein, | taxID used:135614 OG01978|WP\_010365148.1 | MULTISPECIES: MoxR family ATPase [X | taxID used:135614 OG01979|WP\_010367514.1 | MULTISPECIES: TauD/TfdA family diox | taxID used:135614 OG00197|WP\_010364539.1 | MULTISPECIES: penicillin-binding pr | taxID used:135614 OG01984|WP\_010365430.1 | MULTISPECIES: DUF2236 domain-contai | taxID used:135614 OG01985|WP\_010368132.1 | MULTISPECIES: 50S ribosomal protein | taxID used:135614 OG01987|WP\_033009644.1 | SPOR domain-containing protein, par | taxID used:135614 OG01988|WP\_010374452.1 | MULTISPECIES: glycine--tRNA ligase | taxID used:135614 OG01990|WP\_010367542.1 | MULTISPECIES: ribokinase [Xanthomon | taxID used:135614 OG01991|WP\_054393921.1 | MULTISPECIES: SMP-30/gluconolactona | taxID used:135614 OG01992|WP\_010370132.1 | MULTISPECIES: ABC transporter perme | taxID used:135614 OG01993|WP\_010374500.1 | MULTISPECIES: tRNA 2-thiocytidine(3 | taxID used:135614 OG01997|WP\_010366896.1 | MULTISPECIES: EamA/RhaT family tran | taxID used:135614 OG01999|WP\_054393917.1 | MULTISPECIES: hypothetical protein | taxID used:135614 OG00199|WP\_017117128.1 | ATP-binding cassette domain-contain | taxID used:135614 OG00019|WP\_010367275.1 | DNA-directed RNA polymerase subunit | taxID used:135614 OG02000|WP\_010364283.1 | MULTISPECIES: efflux RND transporte | taxID used:135614 OG02001|WP\_010367425.1 | MULTISPECIES: EamA/RhaT family tran | taxID used:135614 OG02006|WP\_010365637.1 | electron transfer flavoprotein subu | taxID used:135614 OG02008|WP\_010366690.1 | MULTISPECIES: 50S ribosomal protein | taxID used:135614 OG00200|WP\_010364663.1 | DUF1998 domain-containing protein [ | taxID used:135614 OG02012|WP\_010368289.1 | MULTISPECIES: tyrosine recombinase | taxID used:135614 OG02019|WP\_010374883.1 | MULTISPECIES: hypothetical protein | taxID used:135614 OG02025|WP\_010368054.1 | MULTISPECIES: lipid kinase YegS [Xa | taxID used:135614 OG02026|WP\_010367461.1 | MULTISPECIES: histone deacetylase f | taxID used:135614 OG02027|WP\_010370042.1 | methionyl-tRNA formyltransferase, p | taxID used:135614 OG02029|WP\_010365789.1 | MULTISPECIES: acyl-CoA thioesterase | taxID used:135614 OG00202|WP\_033011892.1 | MULTISPECIES: membrane-bound PQQ-de | taxID used:135614 OG02031|WP\_010367619.1 | MULTISPECIES: UDP-3-O-[3-hydroxymyr | taxID used:135614 OG02035|WP\_010371371.1 | GTP cyclohydrolase I FolE2, partial | taxID used:135614 OG02040|WP\_010371433.1 | EamA/RhaT family transporter [Xanth | taxID used:135614 OG02042|WP\_010365603.1 | MULTISPECIES: MCE family protein [X | taxID used:135614 OG02043|WP\_010372547.1 | spermidine/putrescine ABC transport | taxID used:135614 OG02047|WP\_010371711.1 | MULTISPECIES: malonate decarboxylas | taxID used:135614 OG02049|WP\_010374720.1 | MULTISPECIES: ParB/RepB/Spo0J famil | taxID used:135614 OG02050|WP\_010374858.1 | MULTISPECIES: EamA family transport | taxID used:135614 OG02056|WP\_010371722.1 | MULTISPECIES: biotin-independent ma | taxID used:135614 OG02061|WP\_010372694.1 | MULTISPECIES: tRNA glutamyl-Q(34) s | taxID used:135614 OG02068|WP\_010367433.1 | MULTISPECIES: gluconolactonase [Xan | taxID used:135614 OG02069|WP\_010368280.1 | MULTISPECIES: GGDEF domain-containi | taxID used:135614 OG00206|WP\_084818999.1 | MULTISPECIES: LPS-assembly protein | taxID used:135614 OG02070|WP\_010366209.1 | MULTISPECIES: outer membrane protei | taxID used:135614 OG02075|WP\_010365625.1 | MULTISPECIES: dTDP-4-dehydrorhamnos | taxID used:135614 OG02076|WP\_010374670.1 | MULTISPECIES: cytochrome c oxidase | taxID used:135614 OG02078|WP\_010365492.1 | LysR family transcriptional regulat | taxID used:135614 OG02081|WP\_010367474.1 | MULTISPECIES: prolipoprotein diacyl | taxID used:135614 OG02082|WP\_010372808.1 | MULTISPECIES: M23 family metallopep | taxID used:135614 OG02086|WP\_010368105.1 | MULTISPECIES: membrane protein [Xan | taxID used:135614 OG02089|WP\_010373037.1 | MULTISPECIES: bifunctional methylen | taxID used:135614 OG02090|WP\_010364403.1 | MULTISPECIES: helix-turn-helix doma | taxID used:135614 OG02091|WP\_010365629.1 | MULTISPECIES: glucose-1-phosphate t | taxID used:135614 OG02092|WP\_010368103.1 | NAD(+) diphosphatase, partial [Xant | taxID used:135614 OG02093|WP\_010366658.1 | acetyl-CoA carboxylase carboxyltran | taxID used:135614 OG02094|WP\_017115997.1 | MULTISPECIES: hypothetical protein | taxID used:135614 OG02095|WP\_010372975.1 | MULTISPECIES: apolipoprotein acyltr | taxID used:135614 OG02098|WP\_010365697.1 | MULTISPECIES: dihydropteroate synth | taxID used:135614 OG02099|WP\_010363448.1 | MULTISPECIES: dihydrodipicolinate s | taxID used:135614 OG02103|WP\_010372957.1 | MULTISPECIES: sugar ABC transporter | taxID used:135614 OG02106|WP\_010374498.1 | MULTISPECIES: recombination-associa | taxID used:135614 OG02107|WP\_026112734.1 | MULTISPECIES: hypothetical protein | taxID used:135614 OG02108|WP\_010365733.1 | MULTISPECIES: alpha/beta fold hydro | taxID used:135614 OG00210|WP\_010370069.1 | MULTISPECIES: DNA topoisomerase I [ | taxID used:135614 OG02110|WP\_017116192.1 | alpha/beta hydrolase, partial [Xant | taxID used:135614 OG02111|WP\_010368112.1 | MULTISPECIES: LysR family transcrip | taxID used:135614 OG02112|WP\_010374476.1 | MULTISPECIES: hypothetical protein | taxID used:135614 OG02115|WP\_010365440.1 | MULTISPECIES: LysR family transcrip | taxID used:135614 OG02119|WP\_026113080.1 | MULTISPECIES: GTPase Era [Xanthomon | taxID used:135614 OG02121|WP\_010375549.1 | MULTISPECIES: clavaminate synthase | taxID used:135614 OG02122|WP\_010365750.1 | MULTISPECIES: 4-hydroxy-tetrahydrod | taxID used:135614 OG02124|WP\_010368419.1 | MULTISPECIES: methylisocitrate lyas | taxID used:135614 OG02125|WP\_010374815.1 | MULTISPECIES: TIGR01777 family prot | taxID used:135614 OG02127|WP\_010364428.1 | MULTISPECIES: membrane protein [Xan | taxID used:135614 OG02130|WP\_010367130.1 | MULTISPECIES: hydroxymethylglutaryl | taxID used:135614 OG02135|WP\_010366382.1 | hydrogen peroxide-inducible genes a | taxID used:135614 OG02137|WP\_010378449.1 | MULTISPECIES: enoyl-CoA hydratase [ | taxID used:135614 OG02139|WP\_010368226.1 | MULTISPECIES: endonuclease/exonucle | taxID used:135614 OG02140|WP\_010364433.1 | MULTISPECIES: ion transporter [Xant | taxID used:135614 OG02147|WP\_010374763.1 | MULTISPECIES: polyamine aminopropyl | taxID used:135614 OG02150|WP\_010370120.1 | MULTISPECIES: RNA polymerase sigma | taxID used:135614 OG02152|WP\_033009735.1 | MULTISPECIES: hypothetical protein | taxID used:135614 OG02156|WP\_010365759.1 | MULTISPECIES: 2-dehydro-3-deoxygala | taxID used:135614 OG02160|WP\_010363798.1 | MULTISPECIES: prepilin peptidase [X | taxID used:135614 OG02162|WP\_010373162.1 | MULTISPECIES: LysR family transcrip | taxID used:135614 OG02163|WP\_010372697.1 | MULTISPECIES: protease HtpX [Xantho | taxID used:135614 OG02165|WP\_010367313.1 | MULTISPECIES: 4-(cytidine 5'-diphos | taxID used:135614 OG02170|WP\_010365268.1 | MULTISPECIES: chemotaxis protein Ch | taxID used:135614 OG02176|WP\_010371995.1 | MULTISPECIES: F0F1 ATP synthase sub | taxID used:135614 OG02177|WP\_010374360.1 | MULTISPECIES: peptidylprolyl isomer | taxID used:135614 OG02178|WP\_026112837.1 | MULTISPECIES: folate-binding protei | taxID used:135614 OG02179|WP\_008571326.1 | MULTISPECIES: CBS domain-containing | taxID used:135614 OG00217|WP\_010365509.1 | MULTISPECIES: TonB-dependent recept | taxID used:135614 OG02183|WP\_010367522.1 | MULTISPECIES: ABC transporter perme | taxID used:135614 OG02185|WP\_010366205.1 | MULTISPECIES: succinate--CoA ligase | taxID used:135614 OG02186|WP\_010365381.1 | MULTISPECIES: SDR family NAD(P)-dep | taxID used:135614 OG02188|WP\_010374804.1 | MULTISPECIES: alpha/beta fold hydro | taxID used:135614 OG02189|WP\_010365861.1 | MULTISPECIES: histidine kinase [Xan | taxID used:135614 OG02194|WP\_010364377.1 | MULTISPECIES: LysR family transcrip | taxID used:135614 OG02196|WP\_010371714.1 | triphosphoribosyl-dephospho-CoA syn | taxID used:135614 OG00021|WP\_010367276.1 | MULTISPECIES: DNA-directed RNA poly | taxID used:135614 OG02212|WP\_010366485.1 | MULTISPECIES: phosphate ABC transpo | taxID used:135614 OG02213|WP\_010374389.1 | MULTISPECIES: FAA hydrolase family | taxID used:135614 OG02214|WP\_010366169.1 | MULTISPECIES: elongation factor Ts | taxID used:135614 OG02216|WP\_010364847.1 | MULTISPECIES: phosphatase PAP2 fami | taxID used:135614 OG02217|WP\_010372922.1 | MULTISPECIES: glutaminyl-peptide cy | taxID used:135614 OG00221|WP\_054394007.1 | MULTISPECIES: NdvB protein [Xanthom | taxID used:135614 OG02222|WP\_010371886.1 | MULTISPECIES: membrane protein [Xan | taxID used:135614 OG02224|WP\_010373538.1 | MULTISPECIES: helix-turn-helix doma | taxID used:135614 OG02225|WP\_010366646.1 | MULTISPECIES: DUF3034 domain-contai | taxID used:135614 OG02226|WP\_010368448.1 | MULTISPECIES: protease modulator Hf | taxID used:135614 OG02227|WP\_010371601.1 | MULTISPECIES: aldo/keto reductase [ | taxID used:135614 OG02235|WP\_010368595.1 | MULTISPECIES: sulfate ABC transport | taxID used:135614 OG02236|WP\_017117070.1 | MULTISPECIES: tetratricopeptide rep | taxID used:135614 OG02241|WP\_010365273.1 | 2-succinyl-6-hydroxy-2,4-cyclohexad | taxID used:135614 OG02242|WP\_026112938.1 | MULTISPECIES: restriction endonucle | taxID used:135614 OG02244|WP\_043096298.1 | MULTISPECIES: hypothetical protein | taxID used:135614 OG02246|WP\_017116390.1 | MULTISPECIES: DUF72 domain-containi | taxID used:135614 OG02252|WP\_010366694.1 | MULTISPECIES: phosphatidylserine de | taxID used:135614 OG02253|WP\_010366832.1 | MULTISPECIES: thioredoxin [Xanthomo | taxID used:135614 OG02254|WP\_010373751.1 | MULTISPECIES: flagellar biosyntheti | taxID used:135614 OG02257|WP\_010365566.1 | MULTISPECIES: S-formylglutathione h | taxID used:135614 OG00225|WP\_010368195.1 | MULTISPECIES: alpha-N-acetylglucosa | taxID used:135614 OG02261|WP\_026112506.1 | MULTISPECIES: DUF3298 domain-contai | taxID used:135614 OG02263|WP\_010370030.1 | glycosyltransferase family 2 protei | taxID used:135614 OG02265|WP\_010371383.1 | MULTISPECIES: pantoate--beta-alanin | taxID used:135614 OG02271|WP\_010366728.1 | MULTISPECIES: iron-sulfur cluster c | taxID used:135614 OG02272|WP\_010368470.1 | NAD(P)H-hydrate dehydratase, partia | taxID used:135614 OG02274|WP\_010364600.1 | MULTISPECIES: MBL fold metallo-hydr | taxID used:135614 OG02275|WP\_010372500.1 | MULTISPECIES: formate dehydrogenase | taxID used:135614 OG02276|WP\_010364509.1 | hypothetical protein, partial [Xant | taxID used:135614 OG02277|WP\_010366483.1 | MULTISPECIES: phosphate ABC transpo | taxID used:135614 OG02279|WP\_010367438.1 | MULTISPECIES: p-hydroxycinnamoyl Co | taxID used:135614 OG02280|WP\_010367606.1 | MULTISPECIES: methylenetetrahydrofo | taxID used:135614 OG02283|WP\_026112894.1 | MULTISPECIES: spermidine/putrescine | taxID used:135614 OG02284|WP\_010367770.1 | MULTISPECIES: general secretion pat | taxID used:135614 OG02286|WP\_010365879.1 | MULTISPECIES: chemotaxis protein Ch | taxID used:135614 OG02288|WP\_010365668.1 | MULTISPECIES: 2-dehydro-3-deoxyphos | taxID used:135614 OG02289|WP\_010365601.1 | MULTISPECIES: ABC transporter ATP-b | taxID used:135614 OG02292|WP\_010371059.1 | MULTISPECIES: 50S ribosomal protein | taxID used:135614 OG02297|WP\_017115654.1 | MULTISPECIES: ferritin-like domain- | taxID used:135614 OG02302|WP\_010373220.1 | MULTISPECIES: phosphoenolpyruvate s | taxID used:135614 OG02306|WP\_010367520.1 | MULTISPECIES: ABC transporter ATP-b | taxID used:135614 OG02307|WP\_017117497.1 | MULTISPECIES: STAS domain-containin | taxID used:135614 OG02309|WP\_010374712.1 | MULTISPECIES: exodeoxyribonuclease | taxID used:135614 OG02312|WP\_010366286.1 | MULTISPECIES: tRNA threonylcarbamoy | taxID used:135614 OG02315|WP\_010372920.1 | amidohydrolase, partial [Xanthomona | taxID used:135614 OG02320|WP\_010365584.1 | thiol:disulfide interchange protein | taxID used:135614 OG02328|WP\_010370076.1 | hypothetical protein, partial [Xant | taxID used:135614 OG02332|WP\_010365736.1 | MULTISPECIES: bifunctional hydroxym | taxID used:135614 OG02335|WP\_010363730.1 | MULTISPECIES: NADPH-dependent 7-cya | taxID used:135614 OG02336|WP\_010372010.1 | MULTISPECIES: F0F1 ATP synthase sub | taxID used:135614 OG02338|WP\_010363348.1 | MULTISPECIES: DUF3014 domain-contai | taxID used:135614 OG00233|WP\_010366900.1 | MULTISPECIES: GGDEF domain-containi | taxID used:135614 OG02344|WP\_010365965.1 | MULTISPECIES: signal peptidase I [X | taxID used:135614 OG02345|WP\_010372015.1 | MULTISPECIES: hypothetical protein | taxID used:135614 OG02351|WP\_010374818.1 | histidine biosynthesis protein HisI | taxID used:135614 OG02352|WP\_010367473.1 | MULTISPECIES: thymidylate synthase | taxID used:135614 OG02355|WP\_017115556.1 | MULTISPECIES: N-acetylmuramoyl-L-al | taxID used:135614 OG00235|WP\_010366199.1 | MULTISPECIES: penicillin acylase fa | taxID used:135614 OG02361|WP\_054393998.1 | MULTISPECIES: sulfotransferase [Xan | taxID used:135614 OG02362|WP\_010374358.1 | MULTISPECIES: hypothetical protein | taxID used:135614 OG02364|WP\_010373311.1 | MULTISPECIES: succinate dehydrogena | taxID used:135614 OG02365|WP\_010372082.1 | SDR family NAD(P)-dependent oxidore | taxID used:135614 OG02369|WP\_010364431.1 | MULTISPECIES: NAD(P)-dependent oxid | taxID used:135614 OG00236|WP\_033009909.1 | MULTISPECIES: penicillin-binding pr | taxID used:135614 OG02370|WP\_026112683.1 | MULTISPECIES: siderophore-interacti | taxID used:135614 OG02374|WP\_017113519.1 | MULTISPECIES: M48 family peptidase | taxID used:135614 OG02375|WP\_010363366.1 | MULTISPECIES: aliphatic sulfonate A | taxID used:135614 OG02377|WP\_010368069.1 | MULTISPECIES: S-adenosylmethionine | taxID used:135614 OG02381|WP\_010380616.1 | MULTISPECIES: response regulator tr | taxID used:135614 OG02383|WP\_017115867.1 | MULTISPECIES: flagellar brake prote | taxID used:135614 OG02387|WP\_010363524.1 | MULTISPECIES: polysaccharide pyruvy | taxID used:135614 OG02388|WP\_017115589.1 | MULTISPECIES: N-formylglutamate ami | taxID used:135614 OG02389|WP\_017116796.1 | MULTISPECIES: tryptophan synthase s | taxID used:135614 OG02391|WP\_010366170.1 | MULTISPECIES: 30S ribosomal protein | taxID used:135614 OG02400|WP\_010368220.1 | MULTISPECIES: DUF2242 domain-contai | taxID used:135614 OG02401|WP\_010371150.1 | SDR family NAD(P)-dependent oxidore | taxID used:135614 OG02403|WP\_010366073.1 | MULTISPECIES: DUF1295 domain-contai | taxID used:135614 OG02404|WP\_010379135.1 | MULTISPECIES: D-alanyl-D-alanine ca | taxID used:135614 OG02408|WP\_010367323.1 | MULTISPECIES: polyphosphate kinase | taxID used:135614 OG02409|WP\_010366855.1 | MULTISPECIES: DUF4349 domain-contai | taxID used:135614 OG02413|WP\_010367795.1 | MULTISPECIES: hypothetical protein | taxID used:135614 OG02414|WP\_010366428.1 | MULTISPECIES: hypothetical protein | taxID used:135614 OG02415|WP\_010371553.1 | MULTISPECIES: septum site-determini | taxID used:135614 OG02416|WP\_017116708.1 | MULTISPECIES: hypothetical protein | taxID used:135614 OG02418|WP\_010365674.1 | MULTISPECIES: 2-C-methyl-D-erythrit | taxID used:135614 OG02421|WP\_010366148.1 | MULTISPECIES: acyl-ACP--UDP-N-acety | taxID used:135614 OG02423|WP\_010365527.1 | MULTISPECIES: polysaccharide deacet | taxID used:135614 OG02424|WP\_010366184.1 | MULTISPECIES: type I methionyl amin | taxID used:135614 OG02427|WP\_010366292.1 | MULTISPECIES: glycine zipper 2TM do | taxID used:135614 OG02428|WP\_010374496.1 | DUF45 domain-containing protein [Xa | taxID used:135614 OG02429|WP\_010363758.1 | hypothetical protein [Xanthomonas v | taxID used:135614 OG02431|WP\_010364675.1 | MULTISPECIES: DUF1868 domain-contai | taxID used:135614 OG02433|WP\_010365688.1 | MULTISPECIES: LysM peptidoglycan-bi | taxID used:135614 OG02435|WP\_010372053.1 | MULTISPECIES: MetQ/NlpA family ABC | taxID used:135614 OG02436|WP\_026112294.1 | hypothetical protein, partial [Xant | taxID used:135614 OG02437|WP\_010364383.1 | MULTISPECIES: class I SAM-dependent | taxID used:135614 OG02439|WP\_010374354.1 | MULTISPECIES: exodeoxyribonuclease | taxID used:135614 OG02441|WP\_010368065.1 | MULTISPECIES: indole-3-glycerol pho | taxID used:135614 OG02443|WP\_010366672.1 | MULTISPECIES: tRNA pseudouridine(38 | taxID used:135614 OG02444|WP\_010363185.1 | MULTISPECIES: SDR family NAD(P)-dep | taxID used:135614 OG02445|WP\_010374718.1 | MULTISPECIES: ParA family protein [ | taxID used:135614 OG02446|WP\_010367469.1 | MULTISPECIES: 16S rRNA (adenine(151 | taxID used:135614 OG02447|WP\_010366270.1 | MULTISPECIES: ferredoxin--NADP redu | taxID used:135614 OG02448|WP\_010373332.1 | MULTISPECIES: 3-deoxy-manno-octulos | taxID used:135614 OG02449|WP\_010371848.1 | MULTISPECIES: ABC transporter perme | taxID used:135614 OG02457|WP\_017115411.1 | MULTISPECIES: aliphatic sulfonate A | taxID used:135614 OG02459|WP\_017112429.1 | MULTISPECIES: rhomboid family intra | taxID used:135614 OG02461|WP\_010364602.1 | MULTISPECIES: endonuclease [Xanthom | taxID used:135614 OG02462|WP\_010371000.1 | MULTISPECIES: thiazole synthase [Xa | taxID used:135614 OG02465|WP\_010364916.1 | MULTISPECIES: ATP-binding cassette | taxID used:135614 OG02468|WP\_010367160.1 | MULTISPECIES: imidazole glycerol ph | taxID used:135614 OG02471|WP\_026112801.1 | enoyl-CoA hydratase, partial [Xanth | taxID used:135614 OG02472|WP\_010373757.1 | MULTISPECIES: flagellar biosyntheti | taxID used:135614 OG02474|WP\_026112304.1 | MULTISPECIES: response regulator tr | taxID used:135614 OG02476|WP\_017117237.1 | EcsC family protein [Xanthomonas va | taxID used:135614 OG02477|WP\_010365682.1 | MULTISPECIES: 5'/3'-nucleotidase Su | taxID used:135614 OG02482|WP\_010371574.1 | MULTISPECIES: phosphatase PAP2 fami | taxID used:135614 OG02486|WP\_010366119.1 | MULTISPECIES: VUT family protein [X | taxID used:135614 OG02488|WP\_010371311.1 | type IV secretion pathway protein V | taxID used:135614 OG02490|WP\_010365590.1 | MULTISPECIES: CDP-diacylglycerol--s | taxID used:135614 OG02492|WP\_010368574.1 | MULTISPECIES: colicin V biosynthesi | taxID used:135614 OG02493|WP\_010368469.1 | MULTISPECIES: histidine phosphatase | taxID used:135614 OG02494|WP\_010366288.1 | MULTISPECIES: TatD family deoxyribo | taxID used:135614 OG02500|WP\_010373540.1 | MULTISPECIES: type IV pilus biogene | taxID used:135614 OG02504|WP\_010365419.1 | MULTISPECIES: response regulator tr | taxID used:135614 OG02505|WP\_010371951.1 | MULTISPECIES: hypothetical protein | taxID used:135614 OG02506|WP\_010374387.1 | MULTISPECIES: SDR family NAD(P)-dep | taxID used:135614 OG02507|WP\_010368508.1 | MULTISPECIES: hydroxyacylglutathion | taxID used:135614 OG02508|WP\_010370997.1 | tRNA (guanosine(46)-N7)-methyltrans | taxID used:135614 OG02510|WP\_010363620.1 | MULTISPECIES: YdcF family protein [ | taxID used:135614 OG02516|WP\_010367653.1 | MULTISPECIES: hypothetical protein | taxID used:135614 OG02517|WP\_010374473.1 | MULTISPECIES: glutamine amidotransf | taxID used:135614 OG02518|WP\_010373170.1 | MULTISPECIES: SDR family NAD(P)-dep | taxID used:135614 OG02519|WP\_026112891.1 | ubiquinol cytochrome C oxidoreducta | taxID used:135614 OG02521|WP\_010366504.1 | MULTISPECIES: enoyl-CoA hydratase [ | taxID used:135614 OG02522|WP\_010364597.1 | MULTISPECIES: molybdate ABC transpo | taxID used:135614 OG02524|WP\_017115456.1 | MULTISPECIES: GntR family transcrip | taxID used:135614 OG02528|WP\_010367232.1 | sugar ABC transporter permease [Xan | taxID used:135614 OG02529|WP\_010368281.1 | bifunctional demethylmenaquinone me | taxID used:135614 OG02530|WP\_010374297.1 | FliA/WhiG family RNA polymerase sig | taxID used:135614 OG02534|WP\_010365910.1 | MULTISPECIES: tRNA (guanosine(37)-N | taxID used:135614 OG02535|WP\_010368390.1 | MULTISPECIES: transporter [Xanthomo | taxID used:135614 OG02541|WP\_010372609.1 | MULTISPECIES: SDR family NAD(P)-dep | taxID used:135614 OG02542|WP\_010368059.1 | MULTISPECIES: DUF541 domain-contain | taxID used:135614 OG02543|WP\_010373348.1 | MULTISPECIES: sulfurtransferase [Xa | taxID used:135614 OG02547|WP\_010371168.1 | MULTISPECIES: NADH-quinone oxidored | taxID used:135614 OG02549|WP\_017116068.1 | MULTISPECIES: leucyl/phenylalanyl-t | taxID used:135614 OG02552|WP\_005912445.1 | MULTISPECIES: MotA/TolQ/ExbB proton | taxID used:135614 OG02555|WP\_010373611.1 | MULTISPECIES: flagellar basal-body | taxID used:135614 OG02557|WP\_010367131.1 | MULTISPECIES: 3-hydroxy-2-methylbut | taxID used:135614 OG02560|WP\_017116728.1 | MULTISPECIES: S-methyl-5'-thioinosi | taxID used:135614 OG02561|WP\_010363294.1 | MULTISPECIES: 3-deoxy-D-manno-octul | taxID used:135614 OG02562|WP\_010364763.1 | MULTISPECIES: pyridoxine 5'-phospha | taxID used:135614 OG02565|WP\_010368532.1 | MULTISPECIES: Bax inhibitor-1/YccA | taxID used:135614 OG02571|WP\_010363335.1 | trehalose-phosphatase, partial [Xan | taxID used:135614 OG02573|WP\_010364574.1 | MULTISPECIES: SDR family NAD(P)-dep | taxID used:135614 OG02578|WP\_010363742.1 | MULTISPECIES: haloacid dehalogenase | taxID used:135614 OG02580|WP\_010374400.1 | MULTISPECIES: transcriptional regul | taxID used:135614 OG02585|WP\_010373659.1 | MULTISPECIES: SDR family NAD(P)-dep | taxID used:135614 OG02586|WP\_010373229.1 | MULTISPECIES: 3-hydroxybutyrate deh | taxID used:135614 OG02588|WP\_010365555.1 | MULTISPECIES: cellulase [Xanthomona | taxID used:135614 OG02591|WP\_010371764.1 | MULTISPECIES: polyisoprenoid-bindin | taxID used:135614 OG02592|WP\_010382354.1 | MULTISPECIES: beta-ketoacyl-ACP red | taxID used:135614 OG02593|WP\_010374757.1 | MULTISPECIES: NADP-dependent 3-hydr | taxID used:135614 OG02595|WP\_010374478.1 | MULTISPECIES: twin-arginine translo | taxID used:135614 OG02603|WP\_010377815.1 | EEP domain-containing protein [Xant | taxID used:135614 OG02604|WP\_010371152.1 | MULTISPECIES: triose-phosphate isom | taxID used:135614 OG02607|WP\_010368561.1 | MULTISPECIES: UDP-2,3-diacylglucosa | taxID used:135614 OG00260|WP\_010373216.1 | phosphoenolpyruvate synthase, parti | taxID used:135614 OG02610|WP\_026112428.1 | MULTISPECIES: DUF3348 domain-contai | taxID used:135614 OG02611|WP\_010365504.1 | MULTISPECIES: KR domain-containing | taxID used:135614 OG02612|WP\_002811889.1 | MULTISPECIES: response regulator tr | taxID used:135614 OG02614|WP\_017112764.1 | MULTISPECIES: TetR/AcrR family tran | taxID used:135614 OG00261|WP\_010373548.1 | MULTISPECIES: 3-hydroxyacyl-CoA deh | taxID used:135614 OG02620|WP\_010370916.1 | MULTISPECIES: phosphoadenylyl-sulfa | taxID used:135614 OG02621|WP\_010366752.1 | MULTISPECIES: NlpC/P60 family prote | taxID used:135614 OG02622|WP\_010371422.1 | MULTISPECIES: phosphatase PAP2 fami | taxID used:135614 OG02623|WP\_010370124.1 | MULTISPECIES: uracil-DNA glycosylas | taxID used:135614 OG02625|WP\_005993372.1 | MULTISPECIES: 30S ribosomal protein | taxID used:135614 OG02627|WP\_054393944.1 | MULTISPECIES: hypothetical protein | taxID used:135614 OG02628|WP\_010366472.1 | MULTISPECIES: 23S rRNA (guanosine(2 | taxID used:135614 OG00262|WP\_010364401.1 | MULTISPECIES: biopolymer transporte | taxID used:135614 OG02632|WP\_010366586.1 | MULTISPECIES: cell division protein | taxID used:135614 OG02634|WP\_010365002.1 | MULTISPECIES: flagellar motor prote | taxID used:135614 OG02636|WP\_010368405.1 | 3-oxoacyl-ACP reductase FabG, parti | taxID used:135614 OG02638|WP\_010373661.1 | MULTISPECIES: SDR family NAD(P)-dep | taxID used:135614 OG02639|WP\_010367757.1 | MULTISPECIES: GntR family transcrip | taxID used:135614 OG02640|WP\_010364914.1 | MULTISPECIES: ABC transporter perme | taxID used:135614 OG02641|WP\_010366605.1 | hypothetical protein, partial [Xant | taxID used:135614 OG02642|WP\_010368205.1 | MULTISPECIES: KR domain-containing | taxID used:135614 OG02646|WP\_010371630.1 | MULTISPECIES: AraC family transcrip | taxID used:135614 OG02649|WP\_003489074.1 | MULTISPECIES: glycosyltransferase f | taxID used:135614 OG02650|WP\_010374583.1 | orotidine-5'-phosphate decarboxylas | taxID used:135614 OG02651|WP\_010368278.1 | MULTISPECIES: hypothetical protein | taxID used:135614 OG02652|WP\_010368502.1 | MULTISPECIES: DNA polymerase III su | taxID used:135614 OG02653|WP\_010365633.1 | MULTISPECIES: electron transfer fla | taxID used:135614 OG02654|WP\_010366320.1 | MULTISPECIES: SDR family NAD(P)-dep | taxID used:135614 OG02658|WP\_010366789.1 | MULTISPECIES: SIMPL domain-containi | taxID used:135614 OG02659|WP\_033009660.1 | MULTISPECIES: hypothetical protein | taxID used:135614 OG02660|WP\_010367163.1 | MULTISPECIES: 1-(5-phosphoribosyl)- | taxID used:135614 OG02663|WP\_007965956.1 | MULTISPECIES: AraC family transcrip | taxID used:135614 OG02667|WP\_010366898.1 | MULTISPECIES: 23S rRNA pseudouridin | taxID used:135614 OG02669|WP\_010364576.1 | MULTISPECIES: M23 family metallopep | taxID used:135614 OG02671|WP\_010366453.1 | transporter, partial [Xanthomonas v | taxID used:135614 OG02672|WP\_010365620.1 | MULTISPECIES: CoA transferase subun | taxID used:135614 OG02675|WP\_010373322.1 | MULTISPECIES: lipoprotein-releasing | taxID used:135614 OG02679|WP\_010372777.1 | MULTISPECIES: bifunctional 2-polypr | taxID used:135614 OG02680|WP\_084818994.1 | MULTISPECIES: DUF998 domain-contain | taxID used:135614 OG02684|WP\_010374666.1 | MULTISPECIES: SURF1 family protein | taxID used:135614 OG02685|WP\_010366308.1 | MULTISPECIES: cell envelope biogene | taxID used:135614 OG02686|WP\_010368589.1 | MULTISPECIES: hypothetical protein | taxID used:135614 OG02687|WP\_010365645.1 | MULTISPECIES: short-chain dehydroge | taxID used:135614 OG02688|WP\_010368066.1 | MULTISPECIES: haloacid dehalogenase | taxID used:135614 OG02691|WP\_010364507.1 | MULTISPECIES: ribonuclease PH [Xant | taxID used:135614 OG02694|WP\_010365972.1 | MULTISPECIES: DNA repair protein Re | taxID used:135614 OG02696|WP\_010373404.1 | serine/threonine-protein phosphatas | taxID used:135614 OG02698|WP\_010366166.1 | MULTISPECIES: UMP kinase [Xanthomon | taxID used:135614 OG02699|WP\_010366501.1 | MULTISPECIES: endonuclease III [Xan | taxID used:135614 OG02701|WP\_010379111.1 | MULTISPECIES: response regulator tr | taxID used:135614 OG02703|WP\_010366481.1 | MULTISPECIES: phosphate transport s | taxID used:135614 OG02705|WP\_010370980.1 | FAA hydrolase family protein, parti | taxID used:135614 OG02706|WP\_026112491.1 | MULTISPECIES: DUF1349 domain-contai | taxID used:135614 OG02707|WP\_010372714.1 | MULTISPECIES: glutathione S-transfe | taxID used:135614 OG02708|WP\_010371966.1 | MULTISPECIES: ABC transporter ATP-b | taxID used:135614 OG02709|WP\_010367435.1 | MULTISPECIES: hypothetical protein | taxID used:135614 OG02710|WP\_017115332.1 | MULTISPECIES: molecular chaperone [ | taxID used:135614 OG02713|WP\_010365725.1 | MULTISPECIES: pirin family protein | taxID used:135614 OG02714|WP\_010366597.1 | MULTISPECIES: DUF3011 domain-contai | taxID used:135614 OG02716|WP\_010368200.1 | MULTISPECIES: type II secretion sys | taxID used:135614 OG00271|WP\_010367710.1 | MULTISPECIES: TonB-dependent recept | taxID used:135614 OG02721|WP\_010367669.1 | MULTISPECIES: response regulator tr | taxID used:135614 OG02722|WP\_010367485.1 | MULTISPECIES: hypothetical protein | taxID used:135614 OG02723|WP\_010371432.1 | MULTISPECIES: aspartate/glutamate r | taxID used:135614 OG02727|WP\_010379721.1 | MULTISPECIES: ATP-binding cassette | taxID used:135614 OG02728|WP\_010367152.1 | MULTISPECIES: acireductone synthase | taxID used:135614 OG02734|WP\_010367114.1 | MULTISPECIES: 4-hydroxy-tetrahydrod | taxID used:135614 OG02736|WP\_010368498.1 | MULTISPECIES: serine/threonine-prot | taxID used:135614 OG02737|WP\_010372086.1 | MULTISPECIES: rRNA pseudouridine sy | taxID used:135614 OG02739|WP\_010367580.1 | MULTISPECIES: hypothetical protein | taxID used:135614 OG02740|WP\_010371677.1 | MULTISPECIES: hypothetical protein | taxID used:135614 OG02742|WP\_010366213.1 | MULTISPECIES: thiopurine S-methyltr | taxID used:135614 OG02743|WP\_005914015.1 | polysaccharide biosynthesis protein | taxID used:135614 OG02744|WP\_033009707.1 | MULTISPECIES: hypothetical protein | taxID used:135614 OG02746|WP\_010367411.1 | MULTISPECIES: response regulator tr | taxID used:135614 OG02747|WP\_010368230.1 | MULTISPECIES: lipoyl(octanoyl) tran | taxID used:135614 OG02751|WP\_010365433.1 | MULTISPECIES: glycosyl transferase | taxID used:135614 OG02752|WP\_010368273.1 | MULTISPECIES: class I SAM-dependent | taxID used:135614 OG00275|WP\_010373251.1 | RNA-binding transcriptional accesso | taxID used:135614 OG02762|WP\_010366505.1 | MULTISPECIES: peptidylprolyl isomer | taxID used:135614 OG02763|WP\_002806631.1 | MULTISPECIES: phosphate regulon tra | taxID used:135614 OG02765|WP\_010365500.1 | MULTISPECIES: response regulator tr | taxID used:135614 OG02766|WP\_010364271.1 | MULTISPECIES: DUF4194 domain-contai | taxID used:135614 OG02767|WP\_010372780.1 | MULTISPECIES: phosphoglycolate phos | taxID used:135614 OG02770|WP\_010366497.1 | carbonic anhydrase, partial [Xantho | taxID used:135614 OG02773|WP\_010371719.1 | biotin-independent malonate decarbo | taxID used:135614 OG02778|WP\_010372034.1 | MULTISPECIES: membrane protein [Xan | taxID used:135614 OG02779|WP\_017115557.1 | MULTISPECIES: D-alanyl-D-alanine di | taxID used:135614 OG02780|WP\_010374850.1 | MULTISPECIES: ABC transporter ATP-b | taxID used:135614 OG02784|WP\_010368067.1 | MULTISPECIES: cAMP-activated global | taxID used:135614 OG02785|WP\_010364746.1 | MULTISPECIES: rhomboid family intra | taxID used:135614 OG02791|WP\_010366652.1 | MULTISPECIES: methylamine utilizati | taxID used:135614 OG02792|WP\_010374716.1 | MULTISPECIES: hypothetical protein | taxID used:135614 OG02796|WP\_010367280.1 | MULTISPECIES: 50S ribosomal protein | taxID used:135614 OG02798|WP\_010372057.1 | MULTISPECIES: ABC transporter perme | taxID used:135614 OG02804|WP\_010369588.1 | MULTISPECIES: hypothetical protein | taxID used:135614 OG02807|WP\_010365426.1 | MULTISPECIES: hypothetical protein | taxID used:135614 OG02809|WP\_017112818.1 | MULTISPECIES: response regulator tr | taxID used:135614 OG00280|WP\_033012264.1 | MULTISPECIES: TonB-dependent recept | taxID used:135614 OG02812|WP\_017113985.1 | MULTISPECIES: hemolysin III [Xantho | taxID used:135614 OG02813|WP\_010363483.1 | MULTISPECIES: methyltransferase dom | taxID used:135614 OG02814|WP\_010366880.1 | MULTISPECIES: response regulator tr | taxID used:135614 OG02819|WP\_002808458.1 | MULTISPECIES: response regulator tr | taxID used:135614 OG02820|WP\_010371528.1 | MULTISPECIES: DNA-3-methyladenine g | taxID used:135614 OG02826|WP\_010376017.1 | MULTISPECIES: DUF484 domain-contain | taxID used:135614 OG02827|WP\_010380413.1 | MULTISPECIES: DUF502 domain-contain | taxID used:135614 OG02828|WP\_010365969.1 | MULTISPECIES: ribonuclease III [Xan | taxID used:135614 OG02829|WP\_010365512.1 | MULTISPECIES: type 1 glutamine amid | taxID used:135614 OG02834|WP\_017116218.1 | MULTISPECIES: energy transducer Ton | taxID used:135614 OG02835|WP\_010370134.1 | MULTISPECIES: cell division ATP-bin | taxID used:135614 OG02836|WP\_017112868.1 | MULTISPECIES: outer membrane lipopr | taxID used:135614 OG02837|WP\_010368049.1 | MULTISPECIES: ribulose-phosphate 3- | taxID used:135614 OG02841|WP\_010371896.1 | MULTISPECIES: maleylacetoacetate is | taxID used:135614 OG02847|WP\_010366669.1 | MULTISPECIES: N-(5'-phosphoribosyl) | taxID used:135614 OG02849|WP\_003490678.1 | MULTISPECIES: response regulator tr | taxID used:135614 OG02855|WP\_010365684.1 | MULTISPECIES: protein-L-isoaspartat | taxID used:135614 OG02857|WP\_010364430.1 | MULTISPECIES: serine/threonine prot | taxID used:135614 OG02859|WP\_010367363.1 | MULTISPECIES: RNA polymerase sigma | taxID used:135614 OG02860|WP\_010364546.1 | MULTISPECIES: fimbrial protein [Xan | taxID used:135614 OG02865|WP\_010372906.1 | MULTISPECIES: heme exporter protein | taxID used:135614 OG02866|WP\_010364371.1 | MULTISPECIES: TetR/AcrR family tran | taxID used:135614 OG02869|WP\_010373406.1 | MULTISPECIES: type VI secretion sys | taxID used:135614 OG02870|WP\_010367374.1 | MULTISPECIES: NUDIX hydrolase [Xant | taxID used:135614 OG02872|WP\_010367403.1 | MULTISPECIES: peptide-methionine (S | taxID used:135614 OG02876|WP\_010366714.1 | MULTISPECIES: dienelactone hydrolas | taxID used:135614 OG02877|WP\_026112296.1 | MULTISPECIES: class I SAM-dependent | taxID used:135614 OG02880|WP\_010365357.1 | MULTISPECIES: membrane protein [Xan | taxID used:135614 OG02882|WP\_010374767.1 | MULTISPECIES: kinase [Xanthomonas] | taxID used:135614 OG02884|WP\_010368507.1 | MULTISPECIES: hypothetical protein | taxID used:135614 OG02886|WP\_010363741.1 | MULTISPECIES: hypothetical protein | taxID used:135614 OG02887|WP\_017112139.1 | MULTISPECIES: NADH-quinone oxidored | taxID used:135614 OG00288|WP\_010367762.1 | type II secretion system protein Gs | taxID used:135614 OG02891|WP\_010364369.1 | protein-L-isoaspartate O-methyltran | taxID used:135614 OG02892|WP\_010368073.1 | MULTISPECIES: 2-polyprenyl-3-methyl | taxID used:135614 OG02894|WP\_010373663.1 | MULTISPECIES: acetyltransferase [Xa | taxID used:135614 OG02895|WP\_010364381.1 | MULTISPECIES: 3-isopropylmalate deh | taxID used:135614 OG02897|WP\_010364628.1 | MULTISPECIES: DUF938 domain-contain | taxID used:135614 OG00289|WP\_010368087.1 | hybrid sensor histidine kinase/resp | taxID used:135614 OG00028|WP\_010364265.1 | MULTISPECIES: ATP-binding protein [ | taxID used:135614 OG02900|WP\_010367148.1 | MULTISPECIES: methylthioribulose 1- | taxID used:135614 OG02904|WP\_010364909.1 | MULTISPECIES: organic solvent ABC t | taxID used:135614 OG02906|WP\_017113228.1 | MULTISPECIES: glutathione S-transfe | taxID used:135614 OG02907|WP\_010365583.1 | MULTISPECIES: thiol:disulfide inter | taxID used:135614 OG02908|WP\_010373608.1 | MULTISPECIES: flagellar basal body | taxID used:135614 OG02909|WP\_010366692.1 | MULTISPECIES: SCO family protein [X | taxID used:135614 OG02912|WP\_010365382.1 | MULTISPECIES: hypothetical protein | taxID used:135614 OG02917|WP\_010366273.1 | MULTISPECIES: glutathione S-transfe | taxID used:135614 OG02918|WP\_017115600.1 | MULTISPECIES: glutathione S-transfe | taxID used:135614 OG02919|WP\_010371919.1 | MULTISPECIES: phosphoglycolate phos | taxID used:135614 OG02921|WP\_010366476.1 | MULTISPECIES: ribonuclease T [Xanth | taxID used:135614 OG02925|WP\_010364537.1 | MULTISPECIES: hypothetical protein | taxID used:135614 OG02929|WP\_010371717.1 | malonate decarboxylase holo-[acyl-c | taxID used:135614 OG02934|WP\_010367766.1 | MULTISPECIES: general secretion pat | taxID used:135614 OG02939|WP\_010365995.1 | MULTISPECIES: ligase-associated DNA | taxID used:135614 OG02940|WP\_010365521.1 | MULTISPECIES: SGNH/GDSL hydrolase f | taxID used:135614 OG02943|WP\_010364732.1 | MULTISPECIES: hypothetical protein | taxID used:135614 OG02944|WP\_010370864.1 | MULTISPECIES: haloacid dehalogenase | taxID used:135614 OG02949|WP\_010364275.1 | MULTISPECIES: GTP cyclohydrolase I | taxID used:135614 OG02951|WP\_010365606.1 | MULTISPECIES: ABC transporter [Xant | taxID used:135614 OG02952|WP\_054393896.1 | MULTISPECIES: ribose-5-phosphate is | taxID used:135614 OG02953|WP\_010367506.1 | MULTISPECIES: arylesterase [Xanthom | taxID used:135614 OG02958|WP\_010374714.1 | MULTISPECIES: orotate phosphoribosy | taxID used:135614 OG02962|WP\_010373338.1 | MULTISPECIES: CDP-diacylglycerol--g | taxID used:135614 OG02968|WP\_010368581.1 | MULTISPECIES: histidine phosphatase | taxID used:135614 OG02970|WP\_010372025.1 | MULTISPECIES: hypothetical protein | taxID used:135614 OG02972|WP\_010368062.1 | MULTISPECIES: flavin reductase fami | taxID used:135614 OG02973|WP\_017112913.1 | MULTISPECIES: HAD family hydrolase | taxID used:135614 OG02974|WP\_010374793.1 | MULTISPECIES: HTH-type transcriptio | taxID used:135614 OG02975|WP\_010372598.1 | MULTISPECIES: stringent starvation | taxID used:135614 OG02979|WP\_010373667.1 | MULTISPECIES: UDP-3-O-(3-hydroxymyr | taxID used:135614 OG02982|WP\_010363150.1 | MULTISPECIES: HD domain-containing | taxID used:135614 OG02984|WP\_010365694.1 | MULTISPECIES: 23S rRNA (uridine(255 | taxID used:135614 OG02985|WP\_010374599.1 | MULTISPECIES: thymidine kinase [Xan | taxID used:135614 OG02986|WP\_010365618.1 | MULTISPECIES: CoA transferase subun | taxID used:135614 OG02987|WP\_010363800.1 | MULTISPECIES: dephospho-CoA kinase | taxID used:135614 OG02988|WP\_010366825.1 | MULTISPECIES: hypothetical protein | taxID used:135614 OG02991|WP\_010367528.1 | MULTISPECIES: hypothetical protein | taxID used:135614 OG02993|WP\_010367772.1 | MULTISPECIES: general secretion pat | taxID used:135614 OG02997|WP\_010367304.1 | MULTISPECIES: 50S ribosomal protein | taxID used:135614 OG03000|WP\_010373546.1 | MULTISPECIES: TetR/AcrR family tran | taxID used:135614 OG03003|WP\_010365497.1 | MULTISPECIES: response regulator tr | taxID used:135614 OG03005|WP\_033009934.1 | MULTISPECIES: cytochrome o ubiquino | taxID used:135614 OG03008|WP\_010371538.1 | MULTISPECIES: response regulator tr | taxID used:135614 OG03010|WP\_033006739.1 | MULTISPECIES: glycine cleavage syst | taxID used:135614 OG03013|WP\_003482986.1 | MULTISPECIES: response regulator tr | taxID used:135614 OG03017|WP\_010371403.1 | MULTISPECIES: hypothetical protein | taxID used:135614 OG03020|WP\_010374347.1 | 16S rRNA (guanine(527)-N(7))-methyl | taxID used:135614 OG03021|WP\_010373601.1 | MULTISPECIES: flagellar basal body | taxID used:135614 OG03023|WP\_010363624.1 | MULTISPECIES: DUF47 domain-containi | taxID used:135614 OG03027|WP\_017112508.1 | hypothetical protein, partial [Xant | taxID used:135614 OG03028|WP\_007962205.1 | MULTISPECIES: RNA polymerase sigma | taxID used:135614 OG03029|WP\_002806026.1 | MULTISPECIES: ATP-dependent Clp pro | taxID used:135614 OG00302|WP\_010368388.1 | methyl-accepting chemotaxis protein | taxID used:135614 OG03031|WP\_010368467.1 | MULTISPECIES: TetR/AcrR family tran | taxID used:135614 OG03034|WP\_010371121.1 | MULTISPECIES: 30S ribosomal protein | taxID used:135614 OG03035|WP\_010372037.1 | MULTISPECIES: hypothetical protein | taxID used:135614 OG03037|WP\_054393919.1 | MULTISPECIES: YihA family ribosome | taxID used:135614 OG03039|WP\_010367703.1 | MULTISPECIES: hypothetical protein | taxID used:135614 OG00303|WP\_050556469.1 | MULTISPECIES: alpha-glucuronidase [ | taxID used:135614 OG03042|WP\_010365768.1 | MULTISPECIES: 2-dehydro-3-deoxy-6-p | taxID used:135614 OG03043|WP\_010374480.1 | MULTISPECIES: twin-arginine translo | taxID used:135614 OG03045|WP\_010373231.1 | MULTISPECIES: CDP-diacylglycerol--s | taxID used:135614 OG03046|WP\_010372887.1 | MULTISPECIES: DUF1294 domain-contai | taxID used:135614 OG03047|WP\_080990582.1 | MULTISPECIES: hypothetical protein | taxID used:135614 OG03050|WP\_010364511.1 | MULTISPECIES: guanylate kinase [Xan | taxID used:135614 OG03051|WP\_010368084.1 | MULTISPECIES: RNA pyrophosphohydrol | taxID used:135614 OG03056|WP\_010372342.1 | MULTISPECIES: uracil phosphoribosyl | taxID used:135614 OG03058|WP\_010365612.1 | MULTISPECIES: alpha-ketoglutarate-d | taxID used:135614 OG03063|WP\_010366248.1 | MULTISPECIES: rhomboid family intra | taxID used:135614 OG03065|WP\_010374309.1 | MULTISPECIES: chemotaxis protein [X | taxID used:135614 OG03067|WP\_010382263.1 | MULTISPECIES: N-acetyltransferase [ | taxID used:135614 OG03068|WP\_010364604.1 | MULTISPECIES: OmpW family protein [ | taxID used:135614 OG03071|WP\_010364594.1 | MULTISPECIES: molybdenum ABC transp | taxID used:135614 OG03076|WP\_010368185.1 | MULTISPECIES: poly-beta-1,6-N-acety | taxID used:135614 OG03077|WP\_010367327.1 | MULTISPECIES: TlpA family protein d | taxID used:135614 OG03080|WP\_017118338.1 | MULTISPECIES: potassium-transportin | taxID used:135614 OG03081|WP\_010364459.1 | MULTISPECIES: thiamine phosphate sy | taxID used:135614 OG03087|WP\_010368625.1 | MULTISPECIES: glutathione S-transfe | taxID used:135614 OG00308|WP\_010366217.1 | DNA topoisomerase IV subunit A, par | taxID used:135614 OG03090|WP\_010373184.1 | MULTISPECIES: hypothetical protein | taxID used:135614 OG03091|WP\_010371547.1 | MULTISPECIES: GNAT family N-acetylt | taxID used:135614 OG03092|WP\_017116551.1 | MULTISPECIES: hydrolase [Xanthomona | taxID used:135614 OG03095|WP\_010364503.1 | MULTISPECIES: non-canonical purine | taxID used:135614 OG03096|WP\_010373682.1 | MULTISPECIES: flagellar assembly pr | taxID used:135614 OG03097|WP\_010374764.1 | MULTISPECIES: hypothetical protein | taxID used:135614 OG03100|WP\_010366391.1 | MULTISPECIES: TIGR00730 family Ross | taxID used:135614 OG03101|WP\_010373369.1 | MULTISPECIES: NfuA family Fe-S biog | taxID used:135614 OG03104|WP\_010374349.1 | MULTISPECIES: 4'-phosphopantetheiny | taxID used:135614 OG03105|WP\_010366055.1 | MULTISPECIES: RNA 2',3'-cyclic phos | taxID used:135614 OG03107|WP\_010368420.1 | MULTISPECIES: DUF1003 domain-contai | taxID used:135614 OG03110|WP\_010366101.1 | MULTISPECIES: HAD-IB family hydrola | taxID used:135614 OG03111|WP\_017115910.1 | TlpA family protein disulfide reduc | taxID used:135614 OG03114|WP\_010372046.1 | DUF1453 domain-containing protein [ | taxID used:135614 OG03115|WP\_010366869.1 | MULTISPECIES: plasmid pRiA4b ORF-3 | taxID used:135614 OG03116|WP\_026112604.1 | DUF3106 domain-containing protein, | taxID used:135614 OG03119|WP\_010367164.1 | MULTISPECIES: imidazole glycerol ph | taxID used:135614 OG03122|WP\_010372897.1 | DsbE family thiol:disulfide interch | taxID used:135614 OG03124|WP\_054393968.1 | MULTISPECIES: trimeric intracellula | taxID used:135614 OG03125|WP\_017112795.1 | MULTISPECIES: DUF1949 domain-contai | taxID used:135614 OG03127|WP\_017117417.1 | MULTISPECIES: DUF998 domain-contain | taxID used:135614 OG03129|WP\_010371494.1 | MULTISPECIES: hypothetical protein | taxID used:135614 OG00312|WP\_010371182.1 | MULTISPECIES: NADH-quinone oxidored | taxID used:135614 OG03131|WP\_010369585.1 | AAA family ATPase [Xanthomonas vasi | taxID used:135614 OG03137|WP\_010365269.1 | MULTISPECIES: chemoreceptor glutami | taxID used:135614 OG03139|WP\_010374671.1 | MULTISPECIES: cytochrome c oxidase | taxID used:135614 OG00313|WP\_010367530.1 | MULTISPECIES: TonB-dependent hemogl | taxID used:135614 OG03141|WP\_010364248.1 | MULTISPECIES: biliverdin-producing | taxID used:135614 OG03142|WP\_010371054.1 | MULTISPECIES: 50S ribosomal protein | taxID used:135614 OG03143|WP\_010364471.1 | MULTISPECIES: 5-formyltetrahydrofol | taxID used:135614 OG03145|WP\_010363108.1 | MULTISPECIES: repressor LexA [Xanth | taxID used:135614 OG00314|WP\_010368428.1 | MULTISPECIES: TonB-dependent recept | taxID used:135614 OG03154|WP\_010371261.1 | MULTISPECIES: YhgN family NAAT tran | taxID used:135614 OG03155|WP\_010373214.1 | MULTISPECIES: oligoribonuclease [Xa | taxID used:135614 OG03159|WP\_010366514.1 | N-acetylmuramoyl-L-alanine amidase, | taxID used:135614 OG03163|WP\_010367457.1 | MULTISPECIES: hypothetical protein | taxID used:135614 OG03166|WP\_010367701.1 | MULTISPECIES: riboflavin synthase [ | taxID used:135614 OG03167|WP\_010366029.1 | MULTISPECIES: mechanosensitive ion | taxID used:135614 OG03171|WP\_010365139.1 | MULTISPECIES: recombination protein | taxID used:135614 OG03172|WP\_010374843.1 | MULTISPECIES: response regulator tr | taxID used:135614 OG03175|WP\_010367755.1 | MULTISPECIES: nicotinamide riboside | taxID used:135614 OG03180|WP\_010368061.1 | MULTISPECIES: aminodeoxychorismate/ | taxID used:135614 OG03182|WP\_010371766.1 | MULTISPECIES: malonic semialdehyde | taxID used:135614 OG03183|WP\_017112356.1 | polyhydroxyalkanoate synthesis repr | taxID used:135614 OG03184|WP\_010367508.1 | MULTISPECIES: ATP-binding cassette | taxID used:135614 OG03186|WP\_010366559.1 | MULTISPECIES: 3-hydroxyanthranilate | taxID used:135614 OG03187|WP\_010366360.1 | MULTISPECIES: nucleotide exchange f | taxID used:135614 OG03188|WP\_010363129.1 | MULTISPECIES: 2Fe-2S iron-sulfur cl | taxID used:135614 OG03191|WP\_010366727.1 | MULTISPECIES: deoxycytidine triphos | taxID used:135614 OG03194|WP\_010374814.1 | MULTISPECIES: DUF4142 domain-contai | taxID used:135614 OG03198|WP\_017113148.1 | MULTISPECIES: sugar O-acetyltransfe | taxID used:135614 OG00319|WP\_010368115.1 | MULTISPECIES: TIGR01666 family memb | taxID used:135614 OG00031|WP\_010374431.1 | MULTISPECIES: translocation/assembl | taxID used:135614 OG03200|WP\_010371208.1 | MULTISPECIES: ribosome maturation f | taxID used:135614 OG03206|WP\_033009547.1 | MULTISPECIES: DUF924 domain-contain | taxID used:135614 OG03210|WP\_010374415.1 | MULTISPECIES: gluconokinase [Xantho | taxID used:135614 OG03212|WP\_010367298.1 | MULTISPECIES: aminoacyl-tRNA hydrol | taxID used:135614 OG03214|WP\_010367150.1 | MULTISPECIES: acireductone dioxygen | taxID used:135614 OG03216|WP\_010372112.1 | MULTISPECIES: DUF3016 domain-contai | taxID used:135614 OG03217|WP\_010368398.1 | MULTISPECIES: septum formation inhi | taxID used:135614 OG03221|WP\_026112797.1 | MULTISPECIES: hypothetical protein | taxID used:135614 OG03222|WP\_010374664.1 | hypothetical protein, partial [Xant | taxID used:135614 OG03228|WP\_010372152.1 | MULTISPECIES: hypothetical protein | taxID used:135614 OG03229|WP\_017116116.1 | MULTISPECIES: hypothetical protein | taxID used:135614 OG00322|WP\_010367067.1 | TonB-dependent receptor, partial [X | taxID used:135614 OG03230|WP\_010372769.1 | MULTISPECIES: elongation factor P [ | taxID used:135614 OG03232|WP\_010367372.1 | MULTISPECIES: nitroreductase [Xanth | taxID used:135614 OG03233|WP\_010374404.1 | MULTISPECIES: manganese efflux pump | taxID used:135614 OG03236|WP\_010366776.1 | MULTISPECIES: DUF615 domain-contain | taxID used:135614 OG03238|WP\_010365437.1 | MULTISPECIES: flavodoxin family pro | taxID used:135614 OG00323|WP\_026112753.1 | MULTISPECIES: glycerophosphodiester | taxID used:135614 OG03243|WP\_010365877.1 | chemotaxis protein CheB, partial [X | taxID used:135614 OG00324|WP\_010374449.1 | MULTISPECIES: glycine--tRNA ligase | taxID used:135614 OG03253|WP\_017118740.1 | MULTISPECIES: molybdenum cofactor g | taxID used:135614 OG03259|WP\_010367427.1 | MULTISPECIES: nucleoside deaminase | taxID used:135614 OG03260|WP\_010367401.1 | MULTISPECIES: glutamine amidotransf | taxID used:135614 OG03261|WP\_010367393.1 | MULTISPECIES: peroxiredoxin [Xantho | taxID used:135614 OG03262|WP\_010367585.1 | MULTISPECIES: DUF3228 domain-contai | taxID used:135614 OG03263|WP\_010366787.1 | MULTISPECIES: Maf-like protein [Xan | taxID used:135614 OG03267|WP\_005993551.1 | MULTISPECIES: elongation factor P-l | taxID used:135614 OG03268|WP\_026112282.1 | MULTISPECIES: glutathione peroxidas | taxID used:135614 OG03269|WP\_010365627.1 | MULTISPECIES: dTDP-4-dehydrorhamnos | taxID used:135614 OG03270|WP\_010371987.1 | chorismate mutase AroQ, gamma subcl | taxID used:135614 OG03274|WP\_010372031.1 | MULTISPECIES: DNA-deoxyinosine glyc | taxID used:135614 OG03275|WP\_010368425.1 | MULTISPECIES: hypothetical protein | taxID used:135614 OG03276|WP\_010368439.1 | MULTISPECIES: bacterioferritin [Xan | taxID used:135614 OG03279|WP\_010366088.1 | MULTISPECIES: sigma-70 family RNA p | taxID used:135614 OG03281|WP\_010367286.1 | MULTISPECIES: transcription termina | taxID used:135614 OG03283|WP\_010373639.1 | MULTISPECIES: PilZ domain-containin | taxID used:135614 OG03287|WP\_010366542.1 | MULTISPECIES: DUF2939 domain-contai | taxID used:135614 OG03288|WP\_010367352.1 | MULTISPECIES: putative Fe-S cluster | taxID used:135614 OG03293|WP\_010371417.1 | MULTISPECIES: response regulator tr | taxID used:135614 OG03295|WP\_010366443.1 | MULTISPECIES: glutathione peroxidas | taxID used:135614 OG00329|WP\_010371201.1 | MULTISPECIES: NADH-quinone oxidored | taxID used:135614 OG03300|WP\_010372080.1 | MULTISPECIES: DUF1415 domain-contai | taxID used:135614 OG03301|WP\_010372706.1 | MULTISPECIES: YaeQ family protein [ | taxID used:135614 OG03306|WP\_010374593.1 | MULTISPECIES: N-acetyltransferase [ | taxID used:135614 OG03310|WP\_010365991.1 | MULTISPECIES: hypoxanthine-guanine | taxID used:135614 OG03313|WP\_010366302.1 | MULTISPECIES: NAD(P)H-dependent oxi | taxID used:135614 OG03315|WP\_010374840.1 | MULTISPECIES: cytochrome b [Xanthom | taxID used:135614 OG03316|WP\_010370878.1 | MULTISPECIES: O-acetyl-ADP-ribose d | taxID used:135614 OG03319|WP\_010366007.1 | polymer-forming cytoskeletal family | taxID used:135614 OG03322|WP\_080762940.1 | MULTISPECIES: translation initiatio | taxID used:135614 OG03325|WP\_010366165.1 | MULTISPECIES: ribosome recycling fa | taxID used:135614 OG03329|WP\_017113121.1 | MULTISPECIES: NUDIX domain-containi | taxID used:135614 OG03331|WP\_010365495.1 | MULTISPECIES: nuclear transport fac | taxID used:135614 OG03332|WP\_010370954.1 | MULTISPECIES: N-acetyltransferase [ | taxID used:135614 OG03334|WP\_010367459.1 | MULTISPECIES: cob(I)yrinic acid a,c | taxID used:135614 OG03337|WP\_017117875.1 | MULTISPECIES: hypothetical protein | taxID used:135614 OG03338|WP\_010364422.1 | MULTISPECIES: adenylate kinase [Xan | taxID used:135614 OG03339|WP\_010371290.1 | MULTISPECIES: prepilin-type N-termi | taxID used:135614 OG03341|WP\_010379039.1 | Slp family lipoprotein, partial [Xa | taxID used:135614 OG03342|WP\_010374565.1 | MULTISPECIES: membrane protein [Xan | taxID used:135614 OG03344|WP\_010366921.1 | methylated-DNA--protein-cysteine me | taxID used:135614 OG03345|WP\_010371090.1 | MULTISPECIES: 50S ribosomal protein | taxID used:135614 OG03346|WP\_010379770.1 | MULTISPECIES: hypothetical protein | taxID used:135614 OG03350|WP\_017115475.1 | MULTISPECIES: hypothetical protein | taxID used:135614 OG03351|WP\_017115323.1 | MULTISPECIES: bacterioferritin [Xan | taxID used:135614 OG03352|WP\_010368558.1 | MULTISPECIES: phosphatase PAP2 fami | taxID used:135614 OG03355|WP\_010365680.1 | MULTISPECIES: hypothetical protein | taxID used:135614 OG03356|WP\_010364912.1 | MULTISPECIES: outer membrane lipid | taxID used:135614 OG03357|WP\_010365456.1 | MULTISPECIES: fasciclin domain-cont | taxID used:135614 OG03358|WP\_010364408.1 | MULTISPECIES: inorganic pyrophospha | taxID used:135614 OG00335|WP\_010367082.1 | hybrid sensor histidine kinase/resp | taxID used:135614 OG03361|WP\_010367667.1 | MULTISPECIES: metal-dependent hydro | taxID used:135614 OG03362|WP\_017113015.1 | DUF45 domain-containing protein [Xa | taxID used:135614 OG03366|WP\_010367587.1 | MULTISPECIES: DUF2058 domain-contai | taxID used:135614 OG03369|WP\_005997221.1 | MULTISPECIES: HslU--HslV peptidase | taxID used:135614 OG00336|WP\_010368228.1 | MULTISPECIES: PDZ domain-containing | taxID used:135614 OG03370|WP\_010374885.1 | MULTISPECIES: GTPase [Xanthomonas] | taxID used:135614 OG03379|WP\_010364486.1 | MULTISPECIES: hypothetical protein | taxID used:135614 OG03383|WP\_010371945.1 | MULTISPECIES: lactoylglutathione ly | taxID used:135614 OG03385|WP\_003486682.1 | MULTISPECIES: 30S ribosomal protein | taxID used:135614 OG03389|WP\_010368394.1 | MULTISPECIES: hypothetical protein | taxID used:135614 OG03392|WP\_010373213.1 | MULTISPECIES: tRNA adenosine(34) de | taxID used:135614 OG03393|WP\_010371802.1 | MULTISPECIES: hypothetical protein | taxID used:135614 OG03394|WP\_010371131.1 | MULTISPECIES: disulfide bond format | taxID used:135614 OG03395|WP\_010364547.1 | MULTISPECIES: fimbrial protein [Xan | taxID used:135614 OG03401|WP\_010370013.1 | MULTISPECIES: N-acetyltransferase [ | taxID used:135614 OG03402|WP\_010373243.1 | MULTISPECIES: hypothetical protein | taxID used:135614 OG03403|WP\_010373509.1 | MULTISPECIES: hypothetical protein | taxID used:135614 OG03404|WP\_017115583.1 | MULTISPECIES: protease [Xanthomonas | taxID used:135614 OG03406|WP\_010365821.1 | MULTISPECIES: lipoprotein signal pe | taxID used:135614 OG03411|WP\_017112801.1 | MULTISPECIES: DUF2884 domain-contai | taxID used:135614 OG03414|WP\_003490311.1 | MULTISPECIES: transcriptional regul | taxID used:135614 OG03417|WP\_010372834.1 | MULTISPECIES: hypothetical protein | taxID used:135614 OG00341|WP\_010366732.1 | MULTISPECIES: peptidase [Xanthomona | taxID used:135614 OG03422|WP\_010372972.1 | GNAT family N-acetyltransferase [Xa | taxID used:135614 OG03424|WP\_010371924.1 | MULTISPECIES: 3-hydroxyacyl-[acyl-c | taxID used:135614 OG00342|WP\_010367752.1 | TonB-dependent siderophore receptor | taxID used:135614 OG03430|WP\_010367322.1 | MULTISPECIES: molybdenum cofactor b | taxID used:135614 OG03435|WP\_010367278.1 | MULTISPECIES: 50S ribosomal protein | taxID used:135614 OG03436|WP\_010373224.1 | MULTISPECIES: DUF1249 domain-contai | taxID used:135614 OG03437|WP\_010378642.1 | MULTISPECIES: type VI secretion sys | taxID used:135614 OG03441|WP\_010367726.1 | MULTISPECIES: peptide deformylase [ | taxID used:135614 OG03444|WP\_010370045.1 | MULTISPECIES: peptide deformylase [ | taxID used:135614 OG03447|WP\_010367717.1 | MULTISPECIES: hypothetical protein | taxID used:135614 OG03448|WP\_026112710.1 | MULTISPECIES: membrane protein [Xan | taxID used:135614 OG03449|WP\_010365908.1 | ribosome maturation factor RimM, pa | taxID used:135614 OG03451|WP\_010372566.1 | MULTISPECIES: hypothetical protein | taxID used:135614 OG03452|WP\_010373481.1 | MULTISPECIES: ferritin-like domain- | taxID used:135614 OG03454|WP\_010371099.1 | MULTISPECIES: 50S ribosomal protein | taxID used:135614 OG03458|WP\_010368206.1 | MULTISPECIES: 2-amino-4-hydroxy-6-h | taxID used:135614 OG03459|WP\_010367367.1 | MULTISPECIES: DUF3106 domain-contai | taxID used:135614 OG03460|WP\_010371999.1 | MULTISPECIES: F0F1 ATP synthase sub | taxID used:135614 OG03462|WP\_010365667.1 | MULTISPECIES: acetyltransferase [Xa | taxID used:135614 OG03465|WP\_010371772.1 | MULTISPECIES: hypothetical protein | taxID used:135614 OG03472|WP\_010367776.1 | MULTISPECIES: type II secretion sys | taxID used:135614 OG00347|WP\_010366197.1 | MULTISPECIES: TonB-dependent sidero | taxID used:135614 OG03481|WP\_017112600.1 | MULTISPECIES: hypothetical protein | taxID used:135614 OG03493|WP\_017113044.1 | MULTISPECIES: pathogenicity-like pr | taxID used:135614 OG03494|WP\_010368399.1 | MULTISPECIES: characterized ACR pro | taxID used:135614 OG03497|WP\_010366344.1 | MULTISPECIES: SsrA-binding protein | taxID used:135614 OG03498|WP\_010373226.1 | MULTISPECIES: 8-oxo-dGTP diphosphat | taxID used:135614 OG03501|WP\_026112454.1 | MULTISPECIES: purine-binding chemot | taxID used:135614 OG03503|WP\_010372107.1 | MULTISPECIES: hypothetical protein | taxID used:135614 OG03507|WP\_010373427.1 | MULTISPECIES: type VI secretion sys | taxID used:135614 OG03509|WP\_010366256.1 | MULTISPECIES: MarR family transcrip | taxID used:135614 OG03516|WP\_010365979.1 | MULTISPECIES: CYTH domain-containin | taxID used:135614 OG03517|WP\_010372369.1 | pantetheine-phosphate adenylyltrans | taxID used:135614 OG03518|WP\_010366754.1 | MULTISPECIES: acyl-CoA thioesterase | taxID used:135614 OG03519|WP\_010371236.1 | MULTISPECIES: 5-(carboxyamino)imida | taxID used:135614 OG03523|WP\_017112914.1 | MULTISPECIES: GNAT family N-acetylt | taxID used:135614 OG03524|WP\_010363300.1 | MULTISPECIES: cyclic pyranopterin m | taxID used:135614 OG03525|WP\_010366889.1 | MULTISPECIES: putative 4-hydroxy-4- | taxID used:135614 OG03530|WP\_010371560.1 | MULTISPECIES: hypothetical protein | taxID used:135614 OG03533|WP\_003485557.1 | MULTISPECIES: NADH-quinone oxidored | taxID used:135614 OG03535|WP\_010366521.1 | MULTISPECIES: N-acetyltransferase [ | taxID used:135614 OG03536|WP\_017112785.1 | GNAT family N-acetyltransferase [Xa | taxID used:135614 OG03540|WP\_017112754.1 | MULTISPECIES: membrane protein [Xan | taxID used:135614 OG03546|WP\_010365408.1 | MULTISPECIES: ferritin-like domain- | taxID used:135614 OG03550|WP\_010372366.1 | MULTISPECIES: hypothetical protein | taxID used:135614 OG03551|WP\_010367472.1 | MULTISPECIES: dihydrofolate reducta | taxID used:135614 OG03552|WP\_010365676.1 | MULTISPECIES: 2-C-methyl-D-erythrit | taxID used:135614 OG03553|WP\_010364647.1 | MULTISPECIES: NUDIX domain-containi | taxID used:135614 OG03554|WP\_010368051.1 | MULTISPECIES: GNAT family N-acetylt | taxID used:135614 OG03558|WP\_010366269.1 | MULTISPECIES: glutathione peroxidas | taxID used:135614 OG03561|WP\_010365740.1 | MULTISPECIES: hypothetical protein | taxID used:135614 OG03563|WP\_010372798.1 | MULTISPECIES: general stress protei | taxID used:135614 OG03567|WP\_019768432.1 | MULTISPECIES: ribosomal-protein-ala | taxID used:135614 OG03568|WP\_010364633.1 | SRPBCC domain-containing protein [X | taxID used:135614 OG03569|WP\_010365710.1 | MULTISPECIES: CinA family protein [ | taxID used:135614 OG03572|WP\_010367341.1 | MULTISPECIES: asparaginase [Xanthom | taxID used:135614 OG03573|WP\_010364412.1 | MULTISPECIES: hypothetical protein | taxID used:135614 OG03574|WP\_010366469.1 | MULTISPECIES: GFA family protein [X | taxID used:135614 OG03576|WP\_010366748.1 | MULTISPECIES: peptidylprolyl isomer | taxID used:135614 OG00357|WP\_010367255.1 | MULTISPECIES: elongation factor G [ | taxID used:135614 OG03582|WP\_010365579.1 | MULTISPECIES: hypothetical protein | taxID used:135614 OG03583|WP\_010378894.1 | MULTISPECIES: AraC family transcrip | taxID used:135614 OG03584|WP\_010366517.1 | MULTISPECIES: MOSC domain-containin | taxID used:135614 OG03585|WP\_010365717.1 | MULTISPECIES: recombination regulat | taxID used:135614 OG03586|WP\_010368441.1 | MULTISPECIES: Hsp20/alpha crystalli | taxID used:135614 OG03587|WP\_010368256.1 | MULTISPECIES: rod shape-determining | taxID used:135614 OG03588|WP\_010368146.1 | MULTISPECIES: acetyl-CoA carboxylas | taxID used:135614 OG03589|WP\_010372569.1 | MULTISPECIES: rRNA maturation RNase | taxID used:135614 OG03592|WP\_010364468.1 | MULTISPECIES: EVE domain-containing | taxID used:135614 OG03594|WP\_010368440.1 | MULTISPECIES: peroxiredoxin [Xantho | taxID used:135614 OG03596|WP\_017113275.1 | MULTISPECIES: RDD family protein [X | taxID used:135614 OG03597|WP\_010365744.1 | MULTISPECIES: peroxiredoxin [Xantho | taxID used:135614 OG03598|WP\_010372066.1 | MULTISPECIES: YajQ family cyclic di | taxID used:135614 OG03599|WP\_010363763.1 | MULTISPECIES: hypothetical protein | taxID used:135614 OG00359|WP\_010364527.1 | ATP-dependent DNA helicase RecG, pa | taxID used:135614 OG03604|WP\_010366275.1 | MULTISPECIES: DUF456 domain-contain | taxID used:135614 OG03605|WP\_010370854.1 | MULTISPECIES: hypothetical protein | taxID used:135614 OG03606|WP\_080762920.1 | MULTISPECIES: hypothetical protein | taxID used:135614 OG03607|WP\_010370057.1 | MULTISPECIES: DUF494 family protein | taxID used:135614 OG00360|WP\_010371134.1 | MULTISPECIES: TonB-dependent recept | taxID used:135614 OG03615|WP\_010371915.1 | MULTISPECIES: GAF domain-containing | taxID used:135614 OG03619|WP\_017115548.1 | MULTISPECIES: DUF2244 domain-contai | taxID used:135614 OG00361|WP\_026112719.1 | MULTISPECIES: oligopeptidase B [Xan | taxID used:135614 OG03620|WP\_010374352.1 | aldehyde-activating protein [Xantho | taxID used:135614 OG03624|WP\_010381413.1 | MULTISPECIES: AraC family transcrip | taxID used:135614 OG03629|WP\_010363341.1 | DUF3574 domain-containing protein, | taxID used:135614 OG03631|WP\_010374823.1 | MULTISPECIES: hypothetical protein | taxID used:135614 OG03632|WP\_010372124.1 | MULTISPECIES: AsnC family transcrip | taxID used:135614 OG03634|WP\_002806488.1 | MULTISPECIES: chemotaxis protein Ch | taxID used:135614 OG03638|WP\_010366796.1 | 23S rRNA (pseudouridine(1915)-N(3)) | taxID used:135614 OG03640|WP\_010372135.1 | MULTISPECIES: peptide-methionine (R | taxID used:135614 OG03642|WP\_010366189.1 | MULTISPECIES: hypothetical protein | taxID used:135614 OG03644|WP\_010373416.1 | MULTISPECIES: hypothetical protein | taxID used:135614 OG03647|WP\_010365775.1 | MULTISPECIES: hypothetical protein | taxID used:135614 OG00364|WP\_010371604.1 | MULTISPECIES: catalase HPII [Xantho | taxID used:135614 OG03650|WP\_010363493.1 | MULTISPECIES: hypothetical protein | taxID used:135614 OG03651|WP\_010371959.1 | MULTISPECIES: hypothetical protein | taxID used:135614 OG03652|WP\_005917592.1 | MULTISPECIES: 30S ribosomal protein | taxID used:135614 OG03653|WP\_010366039.1 | MULTISPECIES: peptidylprolyl isomer | taxID used:135614 OG03654|WP\_010372901.1 | MULTISPECIES: cytochrome c biogenes | taxID used:135614 OG03656|WP\_003483093.1 | MULTISPECIES: bacterioferritin [Xan | taxID used:135614 OG03658|WP\_010373333.1 | MULTISPECIES: low molecular weight | taxID used:135614 OG03659|WP\_010367342.1 | MULTISPECIES: Cys-tRNA(Pro) deacyla | taxID used:135614 OG03661|WP\_010367695.1 | MULTISPECIES: transcription antiter | taxID used:135614 OG03665|WP\_011408858.1 | MULTISPECIES: transcription elongat | taxID used:135614 OG03666|WP\_017112466.1 | MULTISPECIES: DUF4870 domain-contai | taxID used:135614 OG03667|WP\_010363518.1 | MULTISPECIES: cupin domain-containi | taxID used:135614 OG03668|WP\_010372841.1 | MULTISPECIES: N-acetyltransferase [ | taxID used:135614 OG03670|WP\_010368619.1 | MULTISPECIES: cell wall hydrolase [ | taxID used:135614 OG03673|WP\_010368505.1 | MULTISPECIES: ribonuclease HI [Xant | taxID used:135614 OG03677|WP\_010366149.1 | MULTISPECIES: 3-hydroxyacyl-[acyl-c | taxID used:135614 OG00367|WP\_010366734.1 | MULTISPECIES: M13 family peptidase | taxID used:135614 OG03684|WP\_010368635.1 | MULTISPECIES: DUF2127 domain-contai | taxID used:135614 OG03689|WP\_010373579.1 | MULTISPECIES: NUDIX hydrolase [Xant | taxID used:135614 OG03690|WP\_026112833.1 | MULTISPECIES: hypothetical protein | taxID used:135614 OG03691|WP\_010373262.1 | MULTISPECIES: cytochrome c biogenes | taxID used:135614 OG03698|WP\_010372001.1 | MULTISPECIES: F0F1 ATP synthase sub | taxID used:135614 OG03701|WP\_010368285.1 | MULTISPECIES: nucleoside deaminase | taxID used:135614 OG03704|WP\_010367697.1 | MULTISPECIES: 6,7-dimethyl-8-ribity | taxID used:135614 OG03707|WP\_026112480.1 | MULTISPECIES: hypothetical protein | taxID used:135614 OG03708|WP\_010363305.1 | MULTISPECIES: molybdenum cofactor b | taxID used:135614 OG03709|WP\_010372141.1 | hypothetical protein, partial [Xant | taxID used:135614 OG03710|WP\_010372073.1 | MULTISPECIES: hypothetical protein | taxID used:135614 OG03711|WP\_010367437.1 | MULTISPECIES: MarR family transcrip | taxID used:135614 OG03712|WP\_010373212.1 | MULTISPECIES: manganese-binding tra | taxID used:135614 OG03713|WP\_010366834.1 | MULTISPECIES: DUF4442 domain-contai | taxID used:135614 OG03719|WP\_010364446.1 | MULTISPECIES: azurin [Xanthomonas] | taxID used:135614 OG00372|WP\_010363719.1 | methyl-accepting chemotaxis protein | taxID used:135614 OG03732|WP\_010381317.1 | MULTISPECIES: 3-dehydroquinate dehy | taxID used:135614 OG03733|WP\_033006748.1 | MULTISPECIES: division/cell wall cl | taxID used:135614 OG03737|WP\_010366052.1 | MULTISPECIES: PA2169 family four-he | taxID used:135614 OG03738|WP\_017112974.1 | MULTISPECIES: DUF192 domain-contain | taxID used:135614 OG00373|WP\_010364429.1 | MULTISPECIES: phosphatase PAP2 fami | taxID used:135614 OG03740|WP\_010365869.1 | MULTISPECIES: thioredoxin TrxC [Xan | taxID used:135614 OG03747|WP\_010371503.1 | MULTISPECIES: hypothetical protein | taxID used:135614 OG03749|WP\_010366107.1 | MULTISPECIES: DUF2147 domain-contai | taxID used:135614 OG03750|WP\_010366134.1 | MULTISPECIES: membrane protein [Xan | taxID used:135614 OG03761|WP\_010363425.1 | MULTISPECIES: hypothetical protein | taxID used:135614 OG03768|WP\_026112749.1 | PepSY domain-containing protein [Xa | taxID used:135614 OG03770|WP\_010364278.1 | MULTISPECIES: MarR family transcrip | taxID used:135614 OG03773|WP\_017117508.1 | MULTISPECIES: RnfABCDGE type electr | taxID used:135614 OG03776|WP\_010374650.1 | MULTISPECIES: GatB/YqeY domain-cont | taxID used:135614 OG03777|WP\_026112341.1 | MULTISPECIES: DUF721 domain-contain | taxID used:135614 OG03778|WP\_010374879.1 | MULTISPECIES: hypothetical protein | taxID used:135614 OG03779|WP\_010368147.1 | MULTISPECIES: type II 3-dehydroquin | taxID used:135614 OG03781|WP\_010372828.1 | Fe-S cluster assembly protein SufE | taxID used:135614 OG03782|WP\_010368403.1 | MULTISPECIES: MEKHLA domain-contain | taxID used:135614 OG03784|WP\_010370906.1 | MULTISPECIES: DUF2946 domain-contai | taxID used:135614 OG03790|WP\_010366717.1 | MULTISPECIES: HIT domain-containing | taxID used:135614 OG03793|WP\_010366644.1 | group 1 truncated hemoglobin, parti | taxID used:135614 OG03796|WP\_010366584.1 | MULTISPECIES: 50S ribosomal protein | taxID used:135614 OG03797|WP\_010364838.1 | MULTISPECIES: ribonuclease P protei | taxID used:135614 OG03800|WP\_054393882.1 | MULTISPECIES: hypothetical protein | taxID used:135614 OG03807|WP\_010372894.1 | cytochrome c-type biogenesis protei | taxID used:135614 OG03808|WP\_003482689.1 | MULTISPECIES: response regulator [X | taxID used:135614 OG03809|WP\_010366346.1 | MULTISPECIES: type II toxin-antitox | taxID used:135614 OG03810|WP\_010366136.1 | MULTISPECIES: DUF2628 domain-contai | taxID used:135614 OG03812|WP\_005991245.1 | MULTISPECIES: 30S ribosomal protein | taxID used:135614 OG03815|WP\_010367426.1 | MULTISPECIES: hypothetical protein | taxID used:135614 OG03816|WP\_010366284.1 | hypothetical protein, partial [Xant | taxID used:135614 OG03817|WP\_010370007.1 | MULTISPECIES: D-tyrosyl-tRNA(Tyr) d | taxID used:135614 OG03820|WP\_010371365.1 | MULTISPECIES: hypothetical protein | taxID used:135614 OG03823|WP\_026112661.1 | MULTISPECIES: cytochrome c biogenes | taxID used:135614 OG03824|WP\_010367396.1 | MULTISPECIES: nucleoside diphosphat | taxID used:135614 OG03825|WP\_002804358.1 | MULTISPECIES: ferric iron uptake tr | taxID used:135614 OG03826|WP\_010366221.1 | MULTISPECIES: MarR family transcrip | taxID used:135614 OG03827|WP\_010371685.1 | MULTISPECIES: ATP-binding protein [ | taxID used:135614 OG03828|WP\_033006772.1 | MULTISPECIES: hypothetical protein | taxID used:135614 OG00382|WP\_010372758.1 | MULTISPECIES: GGDEF domain-containi | taxID used:135614 OG03832|WP\_003483082.1 | MULTISPECIES: 50S ribosomal protein | taxID used:135614 OG03833|WP\_010363767.1 | MULTISPECIES: hypothetical protein | taxID used:135614 OG03834|WP\_003486676.1 | MULTISPECIES: 50S ribosomal protein | taxID used:135614 OG03839|WP\_010373202.1 | MULTISPECIES: DoxX family protein [ | taxID used:135614 OG03841|WP\_010365792.1 | MULTISPECIES: copper chaperone PCu( | taxID used:135614 OG03848|WP\_010367778.1 | MULTISPECIES: type II secretion sys | taxID used:135614 OG00384|WP\_010371227.1 | MULTISPECIES: polyribonucleotide nu | taxID used:135614 OG03851|WP\_010370977.1 | MULTISPECIES: large-conductance mec | taxID used:135614 OG03858|WP\_017117902.1 | MULTISPECIES: hypothetical protein | taxID used:135614 OG00385|WP\_010366105.1 | MULTISPECIES: methionine--tRNA liga | taxID used:135614 OG03861|WP\_010372925.1 | MULTISPECIES: hypothetical protein | taxID used:135614 OG03864|WP\_010366424.1 | MULTISPECIES: hypothetical protein | taxID used:135614 OG03865|WP\_010368563.1 | MULTISPECIES: hypothetical protein | taxID used:135614 OG03866|WP\_003490202.1 | DNA polymerase III subunit chi, par | taxID used:135614 OG03869|WP\_010372321.1 | MULTISPECIES: Lrp/AsnC family trans | taxID used:135614 OG03872|WP\_010368108.1 | MULTISPECIES: DUF2752 domain-contai | taxID used:135614 OG03881|WP\_010373544.1 | MULTISPECIES: nucleoside-diphosphat | taxID used:135614 OG03882|WP\_010364790.1 | MULTISPECIES: biopolymer transporte | taxID used:135614 OG03884|WP\_010371332.1 | hypothetical protein, partial [Xant | taxID used:135614 OG03885|WP\_005912449.1 | MULTISPECIES: biopolymer transporte | taxID used:135614 OG03889|WP\_010367728.1 | MULTISPECIES: arsenate reductase (g | taxID used:135614 OG00388|WP\_010366557.1 | FUSC family protein, partial [Xanth | taxID used:135614 OG03890|WP\_010367282.1 | MULTISPECIES: 50S ribosomal protein | taxID used:135614 OG03892|WP\_003486706.1 | MULTISPECIES: 50S ribosomal protein | taxID used:135614 OG03894|WP\_010381522.1 | MULTISPECIES: hypothetical protein | taxID used:135614 OG03895|WP\_010373366.1 | energy transducer TonB, partial [Xa | taxID used:135614 OG03896|WP\_010367774.1 | MULTISPECIES: prepilin-type N-termi | taxID used:135614 OG03903|WP\_026112522.1 | integral membrane protein [Xanthomo | taxID used:135614 OG03908|WP\_082320278.1 | MULTISPECIES: acyl-CoA thioesterase | taxID used:135614 OG03909|WP\_010368296.1 | MULTISPECIES: DUF454 domain-contain | taxID used:135614 OG00390|WP\_010373764.1 | bifunctional diguanylate cyclase/ph | taxID used:135614 OG03913|WP\_010365967.1 | MULTISPECIES: DUF4845 domain-contai | taxID used:135614 OG03916|WP\_010371989.1 | MULTISPECIES: F0F1 ATP synthase sub | taxID used:135614 OG03921|WP\_017116020.1 | MULTISPECIES: hypothetical protein | taxID used:135614 OG03922|WP\_010368203.1 | MULTISPECIES: VanZ family protein [ | taxID used:135614 OG03924|WP\_005912943.1 | MULTISPECIES: ribosome silencing fa | taxID used:135614 OG03930|WP\_017115424.1 | CopG family transcriptional regulat | taxID used:135614 OG03931|WP\_054393890.1 | MULTISPECIES: DUF2384 domain-contai | taxID used:135614 OG03932|WP\_010373636.1 | MULTISPECIES: flagellar export chap | taxID used:135614 OG03933|WP\_010371304.1 | MULTISPECIES: hypothetical protein | taxID used:135614 OG00393|WP\_010368254.1 | penicillin-binding protein 2, parti | taxID used:135614 OG03941|WP\_010367292.1 | MULTISPECIES: preprotein translocas | taxID used:135614 OG03942|WP\_010365650.1 | MULTISPECIES: GtrA family protein [ | taxID used:135614 OG03946|WP\_010367436.1 | MULTISPECIES: hypothetical protein | taxID used:135614 OG03947|WP\_010373606.1 | MULTISPECIES: flagellar basal body | taxID used:135614 OG03949|WP\_010366673.1 | MULTISPECIES: VOC family protein [X | taxID used:135614 OG03950|WP\_003486318.1 | MULTISPECIES: type II toxin-antitox | taxID used:135614 OG03956|WP\_010373306.1 | MULTISPECIES: succinate dehydrogena | taxID used:135614 OG03958|WP\_010366740.1 | MULTISPECIES: methylated-DNA--prote | taxID used:135614 OG03963|WP\_010368496.1 | MULTISPECIES: hypothetical protein | taxID used:135614 OG03964|WP\_010365921.1 | MULTISPECIES: RNA-binding S4 domain | taxID used:135614 OG03966|WP\_010364490.1 | MULTISPECIES: hypothetical protein | taxID used:135614 OG03967|WP\_010371581.1 | MULTISPECIES: hypothetical protein | taxID used:135614 OG03972|WP\_010373264.1 | cytochrome c4, partial [Xanthomonas | taxID used:135614 OG03973|WP\_003485983.1 | MULTISPECIES: virulence regulator [ | taxID used:135614 OG03977|WP\_005989873.1 | MULTISPECIES: 50S ribosomal protein | taxID used:135614 OG00397|WP\_054393992.1 | MULTISPECIES: peptidase M2 family p | taxID used:135614 OG03984|WP\_084818987.1 | hypothetical protein, partial [Xant | taxID used:135614 OG03987|WP\_010373604.1 | MULTISPECIES: flagellar basal body | taxID used:135614 OG03989|WP\_010371917.1 | MULTISPECIES: transcriptional regul | taxID used:135614 OG00398|WP\_010368268.1 | MULTISPECIES: asparagine synthase ( | taxID used:135614 OG03993|WP\_010363844.1 | MULTISPECIES: histone-like nucleoid | taxID used:135614 OG03995|WP\_010365591.1 | MULTISPECIES: DUF4124 domain-contai | taxID used:135614 OG03997|WP\_026112875.1 | MULTISPECIES: fluoride efflux trans | taxID used:135614 OG04001|WP\_010366909.1 | MULTISPECIES: membrane protein [Xan | taxID used:135614 OG04003|WP\_010364644.1 | MULTISPECIES: host attachment prote | taxID used:135614 OG04005|WP\_010373749.1 | MULTISPECIES: flagellar biosyntheti | taxID used:135614 OG04007|WP\_010364875.1 | MULTISPECIES: DUF2628 domain-contai | taxID used:135614 OG04018|WP\_002813536.1 | MULTISPECIES: chemotaxis protein Ch | taxID used:135614 OG00401|WP\_010365949.1 | MULTISPECIES: 3-hydroxyacyl-CoA deh | taxID used:135614 OG04020|WP\_010367470.1 | MULTISPECIES: Co2+/Mg2+ efflux prot | taxID used:135614 OG04023|WP\_017119420.1 | MULTISPECIES: DUF4424 domain-contai | taxID used:135614 OG04027|WP\_010380421.1 | MULTISPECIES: VOC family protein [X | taxID used:135614 OG00402|WP\_010365827.1 | MULTISPECIES: cytochrome o ubiquino | taxID used:135614 OG04030|WP\_010365837.1 | MULTISPECIES: DUF805 domain-contain | taxID used:135614 OG04031|WP\_003486671.1 | MULTISPECIES: 30S ribosomal protein | taxID used:135614 OG04035|WP\_010372604.1 | MULTISPECIES: DUF3301 domain-contai | taxID used:135614 OG04036|WP\_010371223.1 | MULTISPECIES: 30S ribosome-binding | taxID used:135614 OG04037|WP\_010371096.1 | MULTISPECIES: 30S ribosomal protein | taxID used:135614 OG04038|WP\_010365857.1 | MULTISPECIES: anti-sigma regulatory | taxID used:135614 OG04039|WP\_010367129.1 | hypothetical protein, partial [Xant | taxID used:135614 OG04043|WP\_010368445.1 | MULTISPECIES: response regulator [X | taxID used:135614 OG04049|WP\_005990700.1 | MULTISPECIES: 30S ribosomal protein | taxID used:135614 OG00404|WP\_010370910.1 | MULTISPECIES: TonB-dependent recept | taxID used:135614 OG04057|WP\_005997154.1 | MULTISPECIES: response regulator tr | taxID used:135614 OG04059|WP\_010374345.1 | MULTISPECIES: hypothetical protein | taxID used:135614 OG04061|WP\_010364690.1 | MULTISPECIES: BlaI/MecI/CopY family | taxID used:135614 OG04062|WP\_010366588.1 | MULTISPECIES: hypothetical protein | taxID used:135614 OG04065|WP\_010368095.1 | MULTISPECIES: iron-sulfur cluster i | taxID used:135614 OG04068|WP\_010373308.1 | MULTISPECIES: succinate dehydrogena | taxID used:135614 OG04069|WP\_003486337.1 | MULTISPECIES: hypothetical protein | taxID used:135614 OG00406|WP\_010368614.1 | MULTISPECIES: NADPH-dependent 2,4-d | taxID used:135614 OG04070|WP\_010374825.1 | MULTISPECIES: hypothetical protein | taxID used:135614 OG04074|WP\_010364890.1 | MULTISPECIES: hypothetical protein | taxID used:135614 OG04075|WP\_003481969.1 | MULTISPECIES: aspartate 1-decarboxy | taxID used:135614 OG04081|WP\_010371890.1 | MULTISPECIES: DUF4398 domain-contai | taxID used:135614 OG04082|WP\_003486665.1 | MULTISPECIES: 50S ribosomal protein | taxID used:135614 OG04085|WP\_010365870.1 | MULTISPECIES: DUF1428 domain-contai | taxID used:135614 OG00408|WP\_026113069.1 | MULTISPECIES: M1 family peptidase [ | taxID used:135614 OG04090|WP\_010366502.1 | MULTISPECIES: hypothetical protein, | taxID used:135614 OG04094|WP\_010364526.1 | MULTISPECIES: RidA family protein [ | taxID used:135614 OG04096|WP\_010367484.1 | MULTISPECIES: diacylglycerol kinase | taxID used:135614 OG04097|WP\_010372858.1 | MULTISPECIES: cupin domain-containi | taxID used:135614 OG00409|WP\_010371933.1 | MULTISPECIES: oligopeptidase A [Xan | taxID used:135614 OG04105|WP\_010367274.1 | MULTISPECIES: 30S ribosomal protein | taxID used:135614 OG04107|WP\_017115517.1 | MULTISPECIES: DUF423 domain-contain | taxID used:135614 OG04109|WP\_010365690.1 | hypothetical protein, partial [Xant | taxID used:135614 OG04112|WP\_010373676.1 | MULTISPECIES: flagellar hook-basal | taxID used:135614 OG04116|WP\_010368136.1 | MULTISPECIES: hypothetical protein | taxID used:135614 OG04119|WP\_010363745.1 | MULTISPECIES: membrane protein [Xan | taxID used:135614 OG00411|WP\_054393997.1 | MULTISPECIES: PAS domain-containing | taxID used:135614 OG04120|WP\_082347496.1 | MULTISPECIES: 4-oxalomesaconate hyd | taxID used:135614 OG04121|WP\_010368130.1 | MULTISPECIES: helix-turn-helix doma | taxID used:135614 OG04122|WP\_026112592.1 | MULTISPECIES: aldehyde-activating p | taxID used:135614 OG04123|WP\_010365672.1 | MULTISPECIES: cell division protein | taxID used:135614 OG04127|WP\_010373258.1 | MULTISPECIES: response regulator [X | taxID used:135614 OG04130|WP\_010365366.1 | MULTISPECIES: YkgJ family cysteine | taxID used:135614 OG04133|WP\_017113116.1 | MULTISPECIES: DNA-binding transcrip | taxID used:135614 OG04134|WP\_002811674.1 | MULTISPECIES: 50S ribosomal protein | taxID used:135614 OG04135|WP\_010371905.1 | MULTISPECIES: 6-carboxytetrahydropt | taxID used:135614 OG04136|WP\_010366091.1 | MULTISPECIES: DUF4345 domain-contai | taxID used:135614 OG04138|WP\_010365887.1 | MULTISPECIES: response regulator [X | taxID used:135614 OG00413|WP\_010366735.1 | MULTISPECIES: M13 family peptidase | taxID used:135614 OG04140|WP\_010367666.1 | MULTISPECIES: YraN family protein [ | taxID used:135614 OG04142|WP\_010365930.1 | MULTISPECIES: hypothetical protein | taxID used:135614 OG04145|WP\_003483692.1 | MULTISPECIES: GntR family transcrip | taxID used:135614 OG04146|WP\_017115452.1 | MULTISPECIES: BON domain-containing | taxID used:135614 OG04148|WP\_017118542.1 | MULTISPECIES: response regulator [X | taxID used:135614 OG00414|WP\_010371293.1 | MULTISPECIES: excinuclease ABC subu | taxID used:135614 OG04150|WP\_002806565.1 | MULTISPECIES: response regulator [X | taxID used:135614 OG04151|WP\_010366191.1 | MULTISPECIES: arsenate reductase [X | taxID used:135614 OG04153|WP\_010374566.1 | MULTISPECIES: ribonuclease E inhibi | taxID used:135614 OG04154|WP\_010373493.1 | hypothetical protein, partial [Xant | taxID used:135614 OG04158|WP\_010367524.1 | MULTISPECIES: hypothetical protein | taxID used:135614 OG04159|WP\_010373368.1 | MULTISPECIES: 4a-hydroxytetrahydrob | taxID used:135614 OG04164|WP\_010371162.1 | MULTISPECIES: NADH-quinone oxidored | taxID used:135614 OG04169|WP\_026112483.1 | MULTISPECIES: hypothetical protein | taxID used:135614 OG00416|WP\_010364568.1 | transketolase, partial [Xanthomonas | taxID used:135614 OG04172|WP\_010363548.1 | MULTISPECIES: MerR family transcrip | taxID used:135614 OG04175|WP\_010365746.1 | MULTISPECIES: ribonuclease E inhibi | taxID used:135614 OG04185|WP\_010365855.1 | MULTISPECIES: STAS domain-containin | taxID used:135614 OG04189|WP\_003484828.1 | MULTISPECIES: 50S ribosomal protein | taxID used:135614 OG04195|WP\_003486672.1 | MULTISPECIES: 30S ribosomal protein | taxID used:135614 OG04200|WP\_010363774.1 | MULTISPECIES: dihydroneopterin aldo | taxID used:135614 OG04203|WP\_010368109.1 | MULTISPECIES: CD225/dispanin family | taxID used:135614 OG04207|WP\_010367277.1 | MULTISPECIES: 50S ribosomal protein | taxID used:135614 OG04210|WP\_010364497.1 | MULTISPECIES: PilZ domain-containin | taxID used:135614 OG04211|WP\_010374733.1 | MULTISPECIES: membrane protein [Xan | taxID used:135614 OG04212|WP\_010371104.1 | MULTISPECIES: 50S ribosomal protein | taxID used:135614 OG04215|WP\_010365142.1 | MULTISPECIES: histidine triad nucle | taxID used:135614 OG04217|WP\_003483889.1 | MULTISPECIES: type IV fimbriae asse | taxID used:135614 OG04222|WP\_017112827.1 | MULTISPECIES: hypothetical protein | taxID used:135614 OG04227|WP\_010374821.1 | MULTISPECIES: hypothetical protein | taxID used:135614 OG04229|WP\_010374881.1 | MULTISPECIES: hypothetical protein | taxID used:135614 OG04230|WP\_017112262.1 | MULTISPECIES: hypothetical protein | taxID used:135614 OG04233|WP\_010366067.1 | MULTISPECIES: RidA family protein [ | taxID used:135614 OG04238|WP\_010372311.1 | MULTISPECIES: preprotein translocas | taxID used:135614 OG04239|WP\_010367365.1 | hypothetical protein, partial [Xant | taxID used:135614 OG00423|WP\_010367674.1 | MULTISPECIES: K(+)-transporting ATP | taxID used:135614 OG04244|WP\_010364330.1 | MULTISPECIES: hypothetical protein | taxID used:135614 OG04249|WP\_010367338.1 | DUF2069 domain-containing protein, | taxID used:135614 OG04250|WP\_010365402.1 | MULTISPECIES: hypothetical protein | taxID used:135614 OG04251|WP\_010365831.1 | MULTISPECIES: cytochrome o ubiquino | taxID used:135614 OG04252|WP\_010372985.1 | MULTISPECIES: hypothetical protein | taxID used:135614 OG04255|WP\_017112813.1 | MULTISPECIES: hypothetical protein | taxID used:135614 OG04260|WP\_010370143.1 | MULTISPECIES: thioredoxin TrxA [Xan | taxID used:135614 OG04263|WP\_010363124.1 | MULTISPECIES: cupin domain-containi | taxID used:135614 OG04264|WP\_054393989.1 | MULTISPECIES: Rieske (2Fe-2S) prote | taxID used:135614 OG04267|WP\_010374888.1 | MULTISPECIES: hypothetical protein | taxID used:135614 OG00426|WP\_054393946.1 | type III secretion system effector | taxID used:135614 OG04273|WP\_010366576.1 | MULTISPECIES: iron-sulfur cluster a | taxID used:135614 OG04276|WP\_010364698.1 | MULTISPECIES: hypothetical protein | taxID used:135614 OG04277|WP\_010371400.1 | MULTISPECIES: type II toxin-antitox | taxID used:135614 OG00427|WP\_026112813.1 | MULTISPECIES: 9-O-acetylesterase [X | taxID used:135614 OG00428|WP\_010374597.1 | ATP-dependent DNA helicase Rep [Xan | taxID used:135614 OG04290|WP\_010363744.1 | EF-hand domain-containing protein [ | taxID used:135614 OG04291|WP\_006453280.1 | MULTISPECIES: ferredoxin family pro | taxID used:135614 OG04295|WP\_010371908.1 | hypothetical protein, partial [Xant | taxID used:135614 OG04300|WP\_008571672.1 | MULTISPECIES: 30S ribosomal protein | taxID used:135614 OG04303|WP\_010368153.1 | MULTISPECIES: divalent-cation toler | taxID used:135614 OG04305|WP\_010363736.1 | MULTISPECIES: glyoxalase [Xanthomon | taxID used:135614 OG04307|WP\_010365945.1 | MULTISPECIES: hypothetical protein | taxID used:135614 OG04311|WP\_010373746.1 | MULTISPECIES: flagellar motor switc | taxID used:135614 OG04316|WP\_010368250.1 | MULTISPECIES: hypothetical protein | taxID used:135614 OG04318|WP\_010364642.1 | MULTISPECIES: thioredoxin [Xanthomo | taxID used:135614 OG04320|WP\_010373035.1 | MULTISPECIES: DUF1244 domain-contai | taxID used:135614 OG04323|WP\_010373167.1 | MULTISPECIES: nuclear transport fac | taxID used:135614 OG04328|WP\_002811686.1 | MULTISPECIES: 50S ribosomal protein | taxID used:135614 OG04329|WP\_010368045.1 | J domain-containing protein, partia | taxID used:135614 OG04333|WP\_010366013.1 | MULTISPECIES: hypothetical protein | taxID used:135614 OG04335|WP\_010371249.1 | MULTISPECIES: monothiol glutaredoxi | taxID used:135614 OG04343|WP\_010373599.1 | MULTISPECIES: hypothetical protein | taxID used:135614 OG04346|WP\_054393969.1 | MULTISPECIES: hypothetical protein | taxID used:135614 OG04347|WP\_010366566.1 | MULTISPECIES: hypothetical protein | taxID used:135614 OG04351|WP\_010367184.1 | MULTISPECIES: EthD family reductase | taxID used:135614 OG04353|WP\_010374903.1 | MULTISPECIES: hypothetical protein | taxID used:135614 OG04354|WP\_010371334.1 | MULTISPECIES: hypothetical protein | taxID used:135614 OG04355|WP\_010368417.1 | MULTISPECIES: hypothetical protein | taxID used:135614 OG04359|WP\_010364861.1 | MULTISPECIES: hypothetical protein | taxID used:135614 OG00435|WP\_010374769.1 | MULTISPECIES: beta-N-acetylglucosam | taxID used:135614 OG04365|WP\_008572943.1 | MULTISPECIES: DUF1820 domain-contai | taxID used:135614 OG04373|WP\_005913896.1 | MULTISPECIES: YbaB/EbfC family nucl | taxID used:135614 OG00437|WP\_010371408.1 | MULTISPECIES: DUF839 domain-contain | taxID used:135614 OG04384|WP\_026112678.1 | MULTISPECIES: hypothetical protein | taxID used:135614 OG04386|WP\_003484328.1 | MULTISPECIES: 50S ribosomal protein | taxID used:135614 OG04392|WP\_010366253.1 | MULTISPECIES: N-acetyltransferase [ | taxID used:135614 OG04395|WP\_010372097.1 | MULTISPECIES: DUF3247 domain-contai | taxID used:135614 OG04398|WP\_010374366.1 | MULTISPECIES: DUF485 domain-contain | taxID used:135614 OG00439|WP\_054393988.1 | MULTISPECIES: sulfate adenylyltrans | taxID used:135614 OG04404|WP\_010364649.1 | MULTISPECIES: hypothetical protein | taxID used:135614 OG04405|WP\_010363352.1 | MULTISPECIES: GIY-YIG nuclease fami | taxID used:135614 OG04408|WP\_010368071.1 | MULTISPECIES: QacE family quaternar | taxID used:135614 OG04412|WP\_006450639.1 | MULTISPECIES: 50S ribosomal protein | taxID used:135614 OG04413|WP\_010368538.1 | MULTISPECIES: glutaredoxin 3 [Xanth | taxID used:135614 OG04418|WP\_010373014.1 | MULTISPECIES: integration host fact | taxID used:135614 OG04427|WP\_010371724.1 | MULTISPECIES: malonate decarboxylas | taxID used:135614 OG04429|WP\_010372813.1 | membrane protein insertion efficien | taxID used:135614 OG00442|WP\_010368512.1 | peptidylprolyl isomerase, partial [ | taxID used:135614 OG04430|WP\_010379215.1 | MULTISPECIES: hypothetical protein | taxID used:135614 OG04435|WP\_010371092.1 | MULTISPECIES: 30S ribosomal protein | taxID used:135614 OG04436|WP\_010373600.1 | MULTISPECIES: flagellar biosynthesi | taxID used:135614 OG04439|WP\_010366326.1 | MULTISPECIES: hypothetical protein | taxID used:135614 OG00443|WP\_010368088.1 | GGDEF domain-containing response re | taxID used:135614 OG04446|WP\_010366568.1 | MULTISPECIES: hypothetical protein | taxID used:135614 OG04450|WP\_017115923.1 | MULTISPECIES: hypothetical protein | taxID used:135614 OG04452|WP\_010365014.1 | MULTISPECIES: STAS domain-containin | taxID used:135614 OG04453|WP\_003484103.1 | MULTISPECIES: DUF465 domain-contain | taxID used:135614 OG00445|WP\_010374370.1 | MULTISPECIES: acetate--CoA ligase [ | taxID used:135614 OG04462|WP\_010364907.1 | MULTISPECIES: STAS domain-containin | taxID used:135614 OG04463|WP\_017118761.1 | MULTISPECIES: hypothetical protein | taxID used:135614 OG04464|WP\_010365692.1 | MULTISPECIES: ribosome assembly RNA | taxID used:135614 OG04465|WP\_003489796.1 | MULTISPECIES: YfhL family 4Fe-4S di | taxID used:135614 OG04466|WP\_026112291.1 | MULTISPECIES: hypothetical protein | taxID used:135614 OG04467|WP\_017113053.1 | MULTISPECIES: type II secretion sys | taxID used:135614 OG04469|WP\_002812428.1 | MULTISPECIES: DNA-directed RNA poly | taxID used:135614 OG04473|WP\_010372935.1 | hypothetical protein, partial [Xant | taxID used:135614 OG04481|WP\_002811694.1 | MULTISPECIES: 50S ribosomal protein | taxID used:135614 OG04482|WP\_010366207.1 | MULTISPECIES: type II toxin-antitox | taxID used:135614 OG04483|WP\_002811076.1 | MULTISPECIES: integration host fact | taxID used:135614 OG04486|WP\_010367065.1 | MULTISPECIES: hypothetical protein | taxID used:135614 OG04490|WP\_010366835.1 | MULTISPECIES: hypothetical protein | taxID used:135614 OG04491|WP\_005914274.1 | MULTISPECIES: NADH-quinone oxidored | taxID used:135614 OG04492|WP\_010372018.1 | MULTISPECIES: hypothetical protein | taxID used:135614 OG00449|WP\_010365929.1 | alpha-L-fucosidase, partial [Xantho | taxID used:135614 OG04502|WP\_010372004.1 | MULTISPECIES: F0F1 ATP synthase sub | taxID used:135614 OG04504|WP\_010373017.1 | MULTISPECIES: DUF1049 domain-contai | taxID used:135614 OG04507|WP\_010377645.1 | MULTISPECIES: hypothetical protein | taxID used:135614 OG04508|WP\_010364474.1 | MULTISPECIES: cell division protein | taxID used:135614 OG00450|WP\_010366300.1 | MULTISPECIES: asparagine synthase ( | taxID used:135614 OG04510|WP\_010367136.1 | MULTISPECIES: DUF2388 domain-contai | taxID used:135614 OG04515|WP\_010363729.1 | MULTISPECIES: LysM peptidoglycan-bi | taxID used:135614 OG00451|WP\_010364587.1 | MULTISPECIES: acetyl-CoA hydrolase | taxID used:135614 OG04523|WP\_017112333.1 | MULTISPECIES: hypothetical protein | taxID used:135614 OG04526|WP\_054394005.1 | MULTISPECIES: hypothetical protein | taxID used:135614 OG04530|WP\_010363428.1 | MULTISPECIES: hypothetical protein | taxID used:135614 OG04532|WP\_010372801.1 | MULTISPECIES: hypothetical protein | taxID used:135614 OG04534|WP\_003486531.1 | MULTISPECIES: hypothetical protein | taxID used:135614 OG00453|WP\_010365695.1 | MULTISPECIES: ATP-dependent metallo | taxID used:135614 OG04547|WP\_010365390.1 | MULTISPECIES: hypothetical protein | taxID used:135614 OG04553|WP\_010363477.1 | MULTISPECIES: oxidative damage prot | taxID used:135614 OG04560|WP\_017112493.1 | MULTISPECIES: hypothetical protein | taxID used:135614 OG04563|WP\_010364340.1 | MULTISPECIES: addiction module anti | taxID used:135614 OG04564|WP\_010372155.1 | MULTISPECIES: DUF4031 domain-contai | taxID used:135614 OG04566|WP\_011347158.1 | MULTISPECIES: DUF4190 domain-contai | taxID used:135614 OG04567|WP\_010371513.1 | MULTISPECIES: hypothetical protein | taxID used:135614 OG04572|WP\_003483210.1 | MULTISPECIES: molecular chaperone G | taxID used:135614 OG04574|WP\_010368232.1 | MULTISPECIES: DUF493 domain-contain | taxID used:135614 OG04575|WP\_042506054.1 | MULTISPECIES: hypothetical protein | taxID used:135614 OG04578|WP\_010370895.1 | MULTISPECIES: hypothetical protein | taxID used:135614 OG04580|WP\_010372506.1 | MULTISPECIES: hypothetical protein | taxID used:135614 OG04583|WP\_005915027.1 | MULTISPECIES: RNA-binding protein H | taxID used:135614 OG04584|WP\_010363490.1 | MULTISPECIES: hypothetical protein | taxID used:135614 OG04596|WP\_010373586.1 | MULTISPECIES: hypothetical protein | taxID used:135614 OG00460|WP\_010365510.1 | MULTISPECIES: amylosucrase [Xanthom | taxID used:135614 OG04612|WP\_010370983.1 | MULTISPECIES: hypothetical protein | taxID used:135614 OG04615|WP\_080762877.1 | MULTISPECIES: DUF1674 domain-contai | taxID used:135614 OG04618|WP\_010365571.1 | MULTISPECIES: metal/formaldehyde-se | taxID used:135614 OG04628|WP\_002804983.1 | MULTISPECIES: DNA-binding transcrip | taxID used:135614 OG04632|WP\_005993369.1 | MULTISPECIES: 30S ribosomal protein | taxID used:135614 OG04636|WP\_010371082.1 | MULTISPECIES: 30S ribosomal protein | taxID used:135614 OG00463|WP\_010365863.1 | MULTISPECIES: hybrid sensor histidi | taxID used:135614 OG04640|WP\_010374776.1 | MULTISPECIES: accessory factor UbiK | taxID used:135614 OG00464|WP\_010370186.1 | MULTISPECIES: 9-O-acetylesterase [X | taxID used:135614 OG04656|WP\_082345118.1 | MULTISPECIES: hypothetical protein | taxID used:135614 OG04657|WP\_010367231.1 | MULTISPECIES: DUF3297 domain-contai | taxID used:135614 OG00465|WP\_010364550.1 | MULTISPECIES: type IV pilus secreti | taxID used:135614 OG04660|WP\_010366347.1 | MULTISPECIES: RnfH family protein [ | taxID used:135614 OG00466|WP\_010368183.1 | MULTISPECIES: poly-beta-1,6-N-acety | taxID used:135614 OG04670|WP\_010376393.1 | MULTISPECIES: hypothetical protein | taxID used:135614 OG04671|WP\_010372091.1 | MULTISPECIES: hypothetical protein | taxID used:135614 OG04673|WP\_005991913.1 | MULTISPECIES: cell division protein | taxID used:135614 OG04680|WP\_010368415.1 | MULTISPECIES: Txe/YoeB family addic | taxID used:135614 OG04682|WP\_003484323.1 | MULTISPECIES: 30S ribosomal protein | taxID used:135614 OG04685|WP\_010367325.1 | acylphosphatase, partial [Xanthomon | taxID used:135614 OG04692|WP\_002806049.1 | MULTISPECIES: HU family DNA-binding | taxID used:135614 OG04707|WP\_005917968.1 | MULTISPECIES: flagellar biosynthesi | taxID used:135614 OG04712|WP\_010371226.1 | MULTISPECIES: 30S ribosomal protein | taxID used:135614 OG04715|WP\_010364255.1 | hypothetical protein, partial [Xant | taxID used:135614 OG04717|WP\_010366025.1 | MULTISPECIES: DUF2789 domain-contai | taxID used:135614 OG04725|WP\_010372862.1 | MULTISPECIES: hypothetical protein | taxID used:135614 OG04726|WP\_010365906.1 | MULTISPECIES: 30S ribosomal protein | taxID used:135614 OG04727|WP\_010365804.1 | MULTISPECIES: 50S ribosomal protein | taxID used:135614 OG04728|WP\_010367122.1 | MULTISPECIES: hypothetical protein | taxID used:135614 OG04731|WP\_010373484.1 | MULTISPECIES: stress-induced protei | taxID used:135614 OG04733|WP\_010364392.1 | MULTISPECIES: acetolactate synthase | taxID used:135614 OG04736|WP\_010366215.1 | MULTISPECIES: hypothetical protein | taxID used:135614 OG00473|WP\_010364246.1 | MULTISPECIES: GAF domain-containing | taxID used:135614 OG04741|WP\_010366764.1 | MULTISPECIES: exodeoxyribonuclease | taxID used:135614 OG04745|WP\_010378796.1 | MULTISPECIES: succinate dehydrogena | taxID used:135614 OG04746|WP\_017112973.1 | MULTISPECIES: rubredoxin [Xanthomon | taxID used:135614 OG04748|WP\_003484370.1 | MULTISPECIES: cell division topolog | taxID used:135614 OG04753|WP\_080762923.1 | MULTISPECIES: hypothetical protein | taxID used:135614 OG04759|WP\_010366915.1 | MULTISPECIES: hypothetical protein | taxID used:135614 OG04762|WP\_010366333.1 | MULTISPECIES: hypothetical protein | taxID used:135614 OG04765|WP\_010373381.1 | MULTISPECIES: hypothetical protein | taxID used:135614 OG00476|WP\_010364399.1 | MULTISPECIES: phosphomethylpyrimidi | taxID used:135614 OG04773|WP\_010367654.1 | MULTISPECIES: CcdB antidote-like pr | taxID used:135614 OG04775|WP\_010364531.1 | MULTISPECIES: type B 50S ribosomal | taxID used:135614 OG04778|WP\_005914591.1 | MULTISPECIES: GlsB/YeaQ/YmgE family | taxID used:135614 OG00477|WP\_010368111.1 | MULTISPECIES: MFS transporter [Xant | taxID used:135614 OG04780|WP\_010366438.1 | MULTISPECIES: hypothetical protein | taxID used:135614 OG04799|WP\_010367534.1 | MULTISPECIES: hemin uptake protein | taxID used:135614 OG04804|WP\_010372708.1 | MULTISPECIES: hypothetical protein | taxID used:135614 OG00480|WP\_010373025.1 | polysaccharide biosynthesis protein | taxID used:135614 OG04812|WP\_010371412.1 | hypothetical protein, partial [Xant | taxID used:135614 OG04815|WP\_017117127.1 | MULTISPECIES: hypothetical protein | taxID used:135614 OG04816|WP\_010367126.1 | MULTISPECIES: ferrous iron transpor | taxID used:135614 OG04817|WP\_010363303.1 | MULTISPECIES: molybdopterin convert | taxID used:135614 OG04818|WP\_010371296.1 | MULTISPECIES: hypothetical protein | taxID used:135614 OG00481|WP\_017120201.1 | transcription termination factor Rh | taxID used:135614 OG04823|WP\_017116402.1 | MULTISPECIES: DUF378 domain-contain | taxID used:135614 OG04831|WP\_003486316.1 | MULTISPECIES: AbrB/MazE/SpoVT famil | taxID used:135614 OG04834|WP\_010365394.1 | MULTISPECIES: hypothetical protein | taxID used:135614 OG04840|WP\_002809459.1 | MULTISPECIES: 50S ribosomal protein | taxID used:135614 OG04845|WP\_010365665.1 | MULTISPECIES: hypothetical protein | taxID used:135614 OG04849|WP\_010368295.1 | MULTISPECIES: hypothetical protein | taxID used:135614 OG00484|WP\_010372379.1 | molecular chaperone HtpG, partial [ | taxID used:135614 OG04853|WP\_010368407.1 | MULTISPECIES: acyl carrier protein | taxID used:135614 OG04854|WP\_010374567.1 | MULTISPECIES: DUF5076 domain-contai | taxID used:135614 OG04862|WP\_010366906.1 | MULTISPECIES: RNA-binding S4 domain | taxID used:135614 OG00486|WP\_010367223.1 | protease, partial [Xanthomonas vasi | taxID used:135614 OG04872|WP\_010366000.1 | MULTISPECIES: hypothetical protein | taxID used:135614 OG04878|WP\_005991243.1 | MULTISPECIES: 30S ribosomal protein | taxID used:135614 OG04886|WP\_010366449.1 | MULTISPECIES: hypothetical protein | taxID used:135614 OG00488|WP\_010374762.1 | MULTISPECIES: arginine decarboxylas | taxID used:135614 OG04892|WP\_010365337.1 | MULTISPECIES: DUF1653 domain-contai | taxID used:135614 OG00048|WP\_010373272.1 | MULTISPECIES: efflux RND transporte | taxID used:135614 OG04904|WP\_010374420.1 | MULTISPECIES: hypothetical protein | taxID used:135614 OG04906|WP\_010364883.1 | MULTISPECIES: hypothetical protein | taxID used:135614 OG00490|WP\_010365664.1 | MULTISPECIES: DNA topoisomerase IV | taxID used:135614 OG04911|WP\_082345081.1 | MULTISPECIES: hypothetical protein | taxID used:135614 OG04914|WP\_010373694.1 | MULTISPECIES: hypothetical protein | taxID used:135614 OG04916|WP\_010367414.1 | MULTISPECIES: DUF2007 domain-contai | taxID used:135614 OG04926|WP\_080762968.1 | MULTISPECIES: hypothetical protein | taxID used:135614 OG04927|WP\_010373249.1 | MULTISPECIES: hypothetical protein | taxID used:135614 OG04928|WP\_003484969.1 | MULTISPECIES: Sec-independent prote | taxID used:135614 OG04929|WP\_003489057.1 | MULTISPECIES: hypothetical protein | taxID used:135614 OG00492|WP\_010370087.1 | MULTISPECIES: signal peptide peptid | taxID used:135614 OG00493|WP\_010371953.1 | MULTISPECIES: peptidoglycan-binding | taxID used:135614 OG04952|WP\_010366907.1 | MULTISPECIES: hypothetical protein | taxID used:135614 OG04955|WP\_010373652.1 | MULTISPECIES: acyl carrier protein | taxID used:135614 OG04957|WP\_002813418.1 | MULTISPECIES: translation initiatio | taxID used:135614 OG00495|WP\_010369999.1 | MULTISPECIES: RNA polymerase sigma | taxID used:135614 OG04970|WP\_010373517.1 | MULTISPECIES: hypothetical protein | taxID used:135614 OG04971|WP\_017112409.1 | MULTISPECIES: DUF465 domain-contain | taxID used:135614 OG04980|WP\_010370084.1 | MULTISPECIES: hypothetical protein | taxID used:135614 OG04981|WP\_003486122.1 | MULTISPECIES: TIGR02449 family prot | taxID used:135614 OG04982|WP\_010365365.1 | MULTISPECIES: hypothetical protein | taxID used:135614 OG04991|WP\_003483561.1 | MULTISPECIES: cold-shock protein [X | taxID used:135614 OG04992|WP\_010365327.1 | MULTISPECIES: hypothetical protein | taxID used:135614 OG04998|WP\_010374668.1 | MULTISPECIES: twin transmembrane he | taxID used:135614 OG00499|WP\_026112507.1 | MULTISPECIES: ABC transporter ATP-b | taxID used:135614 OG05009|WP\_002808376.1 | MULTISPECIES: 30S ribosomal protein | taxID used:135614 OG00500|WP\_010371939.1 | MULTISPECIES: copper resistance sys | taxID used:135614 OG05014|WP\_010374870.1 | MULTISPECIES: CsbD family protein [ | taxID used:135614 OG05016|WP\_010363114.1 | MULTISPECIES: AbrB/MazE/SpoVT famil | taxID used:135614 OG05023|WP\_010365364.1 | MULTISPECIES: hypothetical protein | taxID used:135614 OG05026|WP\_009597534.1 | MULTISPECIES: cold-shock protein [X | taxID used:135614 OG05027|WP\_082337878.1 | MULTISPECIES: hypothetical protein | taxID used:135614 OG00502|WP\_010368160.1 | type III secretion system effector | taxID used:135614 OG05032|WP\_010368085.1 | MULTISPECIES: bacterioferritin [Xan | taxID used:135614 OG05039|WP\_010366290.1 | MULTISPECIES: hypothetical protein | taxID used:135614 OG05048|WP\_010374380.1 | MULTISPECIES: hypothetical protein | taxID used:135614 OG05049|WP\_010365721.1 | MULTISPECIES: carbon storage regula | taxID used:135614 OG05061|WP\_003488188.1 | MULTISPECIES: cold-shock protein [X | taxID used:135614 OG05065|WP\_082345101.1 | MULTISPECIES: hypothetical protein | taxID used:135614 OG00507|WP\_010365149.1 | glycosyltransferase family 39 prote | taxID used:135614 OG05080|WP\_010363746.1 | MULTISPECIES: CPXCG motif-containin | taxID used:135614 OG00508|WP\_010370994.1 | MULTISPECIES: SLC13 family permease | taxID used:135614 OG05091|WP\_010364297.1 | MULTISPECIES: hypothetical protein | taxID used:135614 OG05092|WP\_026112988.1 | MULTISPECIES: hypothetical protein | taxID used:135614 OG00509|WP\_010371415.1 | MULTISPECIES: sensor domain-contain | taxID used:135614 OG05100|WP\_010364353.1 | MULTISPECIES: zinc-finger domain-co | taxID used:135614 OG05110|WP\_010366955.1 | MULTISPECIES: hypothetical protein | taxID used:135614 OG05116|WP\_010363489.1 | MULTISPECIES: general stress protei | taxID used:135614 OG05121|WP\_002811096.1 | MULTISPECIES: 50S ribosomal protein | taxID used:135614 OG05143|WP\_010366369.1 | MULTISPECIES: hypothetical protein | taxID used:135614 OG05149|WP\_010368401.1 | MULTISPECIES: 50S ribosomal protein | taxID used:135614 OG05155|WP\_010371003.1 | MULTISPECIES: sulfur carrier protei | taxID used:135614 OG05157|WP\_010373240.1 | MULTISPECIES: PspC domain-containin | taxID used:135614 OG00515|WP\_010367124.1 | ferrous iron transporter B, partial | taxID used:135614 OG05162|WP\_010365383.1 | MULTISPECIES: hypothetical protein | taxID used:135614 OG05181|WP\_017112433.1 | MULTISPECIES: hypothetical protein | taxID used:135614 OG05192|WP\_010365412.1 | MULTISPECIES: hypothetical protein | taxID used:135614 OG05196|WP\_010366250.1 | MULTISPECIES: hypothetical protein | taxID used:135614 OG05206|WP\_003486678.1 | MULTISPECIES: 50S ribosomal protein | taxID used:135614 OG00520|WP\_010366864.1 | ABC transporter ATP-binding protein | taxID used:135614 OG05215|WP\_003486703.1 | MULTISPECIES: 50S ribosomal protein | taxID used:135614 OG05219|WP\_080764490.1 | MULTISPECIES: hypothetical protein | taxID used:135614 OG05223|WP\_010366726.1 | MULTISPECIES: hypothetical protein | taxID used:135614 OG05229|WP\_010366034.1 | MULTISPECIES: hypothetical protein | taxID used:135614 OG05239|WP\_026112677.1 | MULTISPECIES: hypothetical protein | taxID used:135614 OG05241|WP\_010367781.1 | MULTISPECIES: hypothetical protein | taxID used:135614 OG05253|WP\_003487537.1 | MULTISPECIES: 30S ribosomal protein | taxID used:135614 OG05254|WP\_010372635.1 | MULTISPECIES: DUF3606 domain-contai | taxID used:135614 OG05260|WP\_002809462.1 | MULTISPECIES: 50S ribosomal protein | taxID used:135614 OG05263|WP\_010366023.1 | MULTISPECIES: DUF1328 domain-contai | taxID used:135614 OG05269|WP\_010372903.1 | MULTISPECIES: heme exporter protein | taxID used:135614 OG05274|WP\_010363717.1 | MULTISPECIES: hypothetical protein | taxID used:135614 OG00527|WP\_010370923.1 | MULTISPECIES: assimilatory sulfite | taxID used:135614 OG05283|WP\_010374875.1 | MULTISPECIES: entericidin A/B famil | taxID used:135614 OG00528|WP\_010368633.1 | MULTISPECIES: translational GTPase | taxID used:135614 OG00530|WP\_010371960.1 | MULTISPECIES: glutamine--fructose-6 | taxID used:135614 OG00536|WP\_010366861.1 | MULTISPECIES: DUF885 domain-contain | taxID used:135614 OG00537|WP\_010364730.1 | MULTISPECIES: M20/M25/M40 family me | taxID used:135614 OG00538|WP\_010364562.1 | MULTISPECIES: VWA domain-containing | taxID used:135614 OG00541|WP\_010368210.1 | MULTISPECIES: PAS domain S-box prot | taxID used:135614 OG00543|WP\_010373310.1 | MULTISPECIES: succinate dehydrogena | taxID used:135614 OG00545|WP\_010363333.1 | MULTISPECIES: glycoside hydrolase f | taxID used:135614 OG00546|WP\_010374453.1 | MULTISPECIES: type II/IV secretion | taxID used:135614 OG00547|WP\_010365963.1 | MULTISPECIES: elongation factor 4 [ | taxID used:135614 OG00554|WP\_026112789.1 | MULTISPECIES: outer membrane protei | taxID used:135614 OG00556|WP\_010372021.1 | MULTISPECIES: dihydrolipoyl dehydro | taxID used:135614 OG00559|WP\_010367676.1 | MULTISPECIES: potassium-transportin | taxID used:135614 OG00562|WP\_026112759.1 | MULTISPECIES: glycoside hydrolase [ | taxID used:135614 OG00565|WP\_010372344.1 | MULTISPECIES: cellulase [Xanthomona | taxID used:135614 OG00572|WP\_054394000.1 | gamma-glutamyltransferase, partial | taxID used:135614 OG00573|WP\_010367421.1 | MULTISPECIES: glutamine--tRNA ligas | taxID used:135614 OG00574|WP\_010373329.1 | MULTISPECIES: lipid A export permea | taxID used:135614 OG00578|WP\_010374654.1 | DNA primase, partial [Xanthomonas v | taxID used:135614 OG00579|WP\_010364840.1 | membrane protein insertase YidC, pa | taxID used:135614 OG00581|WP\_010366385.1 | MULTISPECIES: ATP-binding cassette | taxID used:135614 OG00587|WP\_010364688.1 | hypothetical protein, partial [Xant | taxID used:135614 OG00589|WP\_010364564.1 | membrane protein, partial [Xanthomo | taxID used:135614 OG00595|WP\_010365833.1 | MULTISPECIES: M61 family peptidase | taxID used:135614 OG00596|WP\_010363784.1 | MULTISPECIES: type IV-A pilus assem | taxID used:135614 OG00597|WP\_010367784.1 | MULTISPECIES: type II secretion sys | taxID used:135614 OG00600|WP\_010372497.1 | MULTISPECIES: MFS transporter [Xant | taxID used:135614 OG00601|WP\_010367100.1 | MULTISPECIES: single-stranded-DNA-s | taxID used:135614 OG00602|WP\_010364393.1 | MULTISPECIES: acetolactate synthase | taxID used:135614 OG00607|WP\_010366760.1 | MULTISPECIES: alkaline phosphatase | taxID used:135614 OG00613|WP\_010374365.1 | MULTISPECIES: cation acetate sympor | taxID used:135614 OG00617|WP\_026112397.1 | peptidase S8, partial [Xanthomonas | taxID used:135614 OG00619|WP\_010370919.1 | NADPH-dependent assimilatory sulfit | taxID used:135614 OG00622|WP\_017116392.1 | MULTISPECIES: serine hydrolase [Xan | taxID used:135614 OG00623|WP\_010374749.1 | arginine--tRNA ligase, partial [Xan | taxID used:135614 OG00630|WP\_010365592.1 | MULTISPECIES: proline--tRNA ligase | taxID used:135614 OG00632|WP\_010371576.1 | MULTISPECIES: phosphoethanolamine t | taxID used:135614 OG00639|WP\_010367344.1 | MULTISPECIES: peptidase S8 [Xanthom | taxID used:135614 OG00640|WP\_010371299.1 | MULTISPECIES: hypothetical protein | taxID used:135614 OG00642|WP\_010365614.1 | MULTISPECIES: electron transfer fla | taxID used:135614 OG00644|WP\_010372882.1 | MULTISPECIES: thiol reductant ABC e | taxID used:135614 OG00648|WP\_010365666.1 | MULTISPECIES: CTP synthase [Xanthom | taxID used:135614 OG00651|WP\_010365712.1 | MULTISPECIES: 2-polyprenylphenol 6- | taxID used:135614 OG00658|WP\_010374555.1 | MULTISPECIES: glucuronate isomerase | taxID used:135614 OG00660|WP\_010371728.1 | MULTISPECIES: malonate decarboxylas | taxID used:135614 OG00662|WP\_026112192.1 | MULTISPECIES: NAD+ synthase [Xantho | taxID used:135614 OG00066|WP\_010374682.1 | MULTISPECIES: bifunctional proline | taxID used:135614 OG00671|WP\_054393987.1 | MULTISPECIES: PepSY domain-containi | taxID used:135614 OG00679|WP\_054393894.1 | MULTISPECIES: flavin monoamine oxid | taxID used:135614 OG00681|WP\_010374606.1 | MULTISPECIES: glucan biosynthesis p | taxID used:135614 OG00683|WP\_010363407.1 | MULTISPECIES: glycosyl hydrolase fa | taxID used:135614 OG00686|WP\_010363479.1 | MULTISPECIES: PAS domain-containing | taxID used:135614 OG00689|WP\_010363469.1 | signal recognition particle-docking | taxID used:135614 OG00691|WP\_017115697.1 | MULTISPECIES: MFS transporter [Xant | taxID used:135614 OG00693|WP\_010367234.1 | MULTISPECIES: Na+/H+ antiporter [Xa | taxID used:135614 OG00699|WP\_010368157.1 | MULTISPECIES: chaperonin GroEL [Xan | taxID used:135614 OG00706|WP\_010365999.1 | MULTISPECIES: ATP-dependent DNA lig | taxID used:135614 OG00708|WP\_010366203.1 | MULTISPECIES: HAMP domain-containin | taxID used:135614 OG00709|WP\_010367156.1 | MULTISPECIES: calcineurin phosphoes | taxID used:135614 OG00716|WP\_010365813.1 | MULTISPECIES: murein biosynthesis i | taxID used:135614 OG00717|WP\_010366698.1 | MULTISPECIES: LysM peptidoglycan-bi | taxID used:135614 OG00718|WP\_010374446.1 | MULTISPECIES: sulfotransferase fami | taxID used:135614 OG00719|WP\_010368418.1 | propionate catabolism operon regula | taxID used:135614 OG00724|WP\_010363436.1 | MULTISPECIES: APC family permease [ | taxID used:135614 OG00725|WP\_010366226.1 | MULTISPECIES: DHA2 family efflux MF | taxID used:135614 OG00726|WP\_010367394.1 | MULTISPECIES: alkyl hydroperoxide r | taxID used:135614 OG00730|WP\_010372538.1 | MULTISPECIES: DHA2 family efflux MF | taxID used:135614 OG00733|WP\_010373041.1 | MULTISPECIES: glutamine-hydrolyzing | taxID used:135614 OG00734|WP\_010371787.1 | MULTISPECIES: hypothetical protein | taxID used:135614 OG00736|WP\_026112115.1 | MULTISPECIES: DUF255 domain-contain | taxID used:135614 OG00739|WP\_010366246.1 | MULTISPECIES: MFS transporter [Xant | taxID used:135614 OG00741|WP\_010368122.1 | MULTISPECIES: bifunctional phosphor | taxID used:135614 OG00743|WP\_010365961.1 | PDZ domain-containing protein, part | taxID used:135614 OG00746|WP\_082345066.1 | MULTISPECIES: type III effector Hop | taxID used:135614 OG00747|WP\_010364388.1 | MULTISPECIES: 2-isopropylmalate syn | taxID used:135614 OG00749|WP\_010364889.1 | MULTISPECIES: DNA recombination pro | taxID used:135614 OG00754|WP\_010366272.1 | MULTISPECIES: fumarate hydratase [X | taxID used:135614 OG00756|WP\_010371414.1 | MULTISPECIES: hypothetical protein | taxID used:135614 OG00761|WP\_017115050.1 | MULTISPECIES: SulP family inorganic | taxID used:135614 OG00763|WP\_010373159.1 | oxidoreductase, partial [Xanthomona | taxID used:135614 OG00765|WP\_017116051.1 | MULTISPECIES: type VI secretion sys | taxID used:135614 OG00766|WP\_010371997.1 | MULTISPECIES: F0F1 ATP synthase sub | taxID used:135614 OG00767|WP\_010367085.1 | MULTISPECIES: lysine--tRNA ligase [ | taxID used:135614 OG00776|WP\_033009681.1 | exopolyphosphatase, partial [Xantho | taxID used:135614 OG00778|WP\_017116512.1 | PDZ domain-containing protein [Xant | taxID used:135614 OG00077|WP\_010365881.1 | chemotaxis protein CheY [Xanthomona | taxID used:135614 OG00780|WP\_010366407.1 | MULTISPECIES: transcriptional regul | taxID used:135614 OG00781|WP\_010370197.1 | MULTISPECIES: xylulokinase [Xanthom | taxID used:135614 OG00783|WP\_010370026.1 | MULTISPECIES: membrane protein [Xan | taxID used:135614 OG00791|WP\_026112694.1 | MULTISPECIES: NADH-quinone oxidored | taxID used:135614 OG00792|WP\_010365937.1 | MULTISPECIES: methylmalonate-semial | taxID used:135614 OG00796|WP\_010371209.1 | transcription termination/antitermi | taxID used:135614 OG00798|WP\_010363528.1 | MULTISPECIES: lipopolysaccharide bi | taxID used:135614 OG00799|WP\_054393925.1 | deoxyribodipyrimidine photo-lyase, | taxID used:135614 OG00801|WP\_010364635.1 | MULTISPECIES: proline/glycine betai | taxID used:135614 OG00802|WP\_010372327.1 | MULTISPECIES: aminotransferase clas | taxID used:135614 OG00804|WP\_010368207.1 | MULTISPECIES: hybrid sensor histidi | taxID used:135614 OG00805|WP\_026112157.1 | MULTISPECIES: MATE family efflux tr | taxID used:135614 OG00808|WP\_010364705.1 | glutamate synthase subunit beta, pa | taxID used:135614 OG00811|WP\_010374563.1 | MULTISPECIES: MFS transporter [Xant | taxID used:135614 OG00813|WP\_010365450.1 | MULTISPECIES: DUF4130 domain-contai | taxID used:135614 OG00815|WP\_010367639.1 | MULTISPECIES: UDP-N-acetylmuramoyla | taxID used:135614 OG00818|WP\_010373648.1 | MULTISPECIES: sigma-54-dependent Fi | taxID used:135614 OG00820|WP\_010367641.1 | MULTISPECIES: UDP-N-acetylmuramoyl- | taxID used:135614 OG00821|WP\_010374516.1 | MULTISPECIES: mannitol dehydrogenas | taxID used:135614 OG00823|WP\_010368055.1 | MULTISPECIES: anthranilate synthase | taxID used:135614 OG00824|WP\_010364759.1 | MULTISPECIES: cardiolipin synthase | taxID used:135614 OG00827|WP\_010363539.1 | MULTISPECIES: undecaprenyl-phosphat | taxID used:135614 OG00082|WP\_010363734.1 | MULTISPECIES: efflux RND transporte | taxID used:135614 OG00830|WP\_010364273.1 | MULTISPECIES: DUF3375 domain-contai | taxID used:135614 OG00831|WP\_038894766.1 | MULTISPECIES: acetylhydrolase [Xant | taxID used:135614 OG00838|WP\_010366550.1 | MULTISPECIES: exodeoxyribonuclease | taxID used:135614 OG00839|WP\_010366878.1 | MULTISPECIES: efflux transporter ou | taxID used:135614 OG00083|WP\_010373274.1 | MULTISPECIES: efflux RND transporte | taxID used:135614 OG00840|WP\_010372574.1 | MULTISPECIES: tRNA (N6-isopentenyl | taxID used:135614 OG00842|WP\_010370866.1 | MULTISPECIES: pyruvate kinase [Xant | taxID used:135614 OG00846|WP\_010364379.1 | MULTISPECIES: 3-isopropylmalate deh | taxID used:135614 OG00847|WP\_017116803.1 | MULTISPECIES: NADH-quinone oxidored | taxID used:135614 OG00848|WP\_033006705.1 | MULTISPECIES: phosphonoacetaldehyde | taxID used:135614 OG00852|WP\_010367146.1 | amino acid permease, partial [Xanth | taxID used:135614 OG00855|WP\_010373039.1 | MULTISPECIES: IMP dehydrogenase [Xa | taxID used:135614 OG00857|WP\_017116206.1 | MULTISPECIES: cysteine--tRNA ligase | taxID used:135614 OG00858|WP\_017115271.1 | MULTISPECIES: DHA2 family efflux MF | taxID used:135614 OG00861|WP\_010365843.1 | MULTISPECIES: AraC family transcrip | taxID used:135614 OG00862|WP\_010373293.1 | MULTISPECIES: glucose-6-phosphate d | taxID used:135614 OG00866|WP\_017116438.1 | polysaccharide biosynthesis protein | taxID used:135614 OG00867|WP\_010374695.1 | membrane protein, partial [Xanthomo | taxID used:135614 OG00869|WP\_010366393.1 | MULTISPECIES: dihydrolipoyl dehydro | taxID used:135614 OG00874|WP\_010366304.1 | MULTISPECIES: replicative DNA helic | taxID used:135614 OG00876|WP\_010365538.1 | MULTISPECIES: amidohydrolase [Xanth | taxID used:135614 OG00087|WP\_010374577.1 | MULTISPECIES: TonB-dependent recept | taxID used:135614 OG00880|WP\_010373699.1 | MULTISPECIES: cardiolipin synthase | taxID used:135614 OG00884|WP\_010367138.1 | MULTISPECIES: FAD-binding oxidoredu | taxID used:135614 OG00887|WP\_010374912.1 | MULTISPECIES: two-component sensor | taxID used:135614 OG00891|WP\_010374819.1 | MULTISPECIES: Do family serine endo | taxID used:135614 OG00895|WP\_010366572.1 | asparagine--tRNA ligase, partial [X | taxID used:135614 OG00897|WP\_010371991.1 | MULTISPECIES: F0F1 ATP synthase sub | taxID used:135614 OG00898|WP\_010368224.1 | MULTISPECIES: nicotinate phosphorib | taxID used:135614 OG00900|WP\_010365742.1 | MULTISPECIES: PhoH family protein [ | taxID used:135614 OG00901|WP\_010366322.1 | MULTISPECIES: efflux transporter ou | taxID used:135614 OG00904|WP\_010370974.1 | M20/M25/M40 family metallo-hydrolas | taxID used:135614 OG00911|WP\_010364880.1 | MULTISPECIES: amino acid permease [ | taxID used:135614 OG00915|WP\_010366111.1 | MULTISPECIES: S-methylmethionine pe | taxID used:135614 OG00916|WP\_010367604.1 | hypothetical protein, partial [Xant | taxID used:135614 OG00917|WP\_010374368.1 | MULTISPECIES: hypothetical protein | taxID used:135614 OG00918|WP\_010373697.1 | pyridine nucleotide-disulfide oxido | taxID used:135614 OG00921|WP\_026112471.1 | MULTISPECIES: ABC transporter subst | taxID used:135614 OG00924|WP\_010374413.1 | MULTISPECIES: MFS transporter [Xant | taxID used:135614 OG00927|WP\_054393932.1 | MULTISPECIES: glutamate--cysteine l | taxID used:135614 OG00929|WP\_054393887.1 | MULTISPECIES: nucleoside hydrolase | taxID used:135614 OG00932|WP\_010366421.1 | MULTISPECIES: class II fumarate hyd | taxID used:135614 OG00934|WP\_017113389.1 | MULTISPECIES: FAD-dependent monooxy | taxID used:135614 OG00937|WP\_010363332.1 | alpha,alpha-trehalose-phosphate syn | taxID used:135614 OG00938|WP\_010367465.1 | MULTISPECIES: molecular chaperone S | taxID used:135614 OG00939|WP\_010368143.1 | MULTISPECIES: acetyl-CoA carboxylas | taxID used:135614 OG00943|WP\_010366037.1 | MULTISPECIES: heat-shock protein Hs | taxID used:135614 OG00947|WP\_010372522.1 | MULTISPECIES: glutamine synthetase | taxID used:135614 OG00953|WP\_010374831.1 | MULTISPECIES: leucyl aminopeptidase | taxID used:135614 OG00956|WP\_010366061.1 | MULTISPECIES: virulence factor fami | taxID used:135614 OG00958|WP\_010366711.1 | 30S ribosomal protein S12 methylthi | taxID used:135614 OG00959|WP\_010365499.1 | MULTISPECIES: sensor histidine kina | taxID used:135614 OG00961|WP\_010365835.1 | DNA repair protein RadA, partial [X | taxID used:135614 OG00962|WP\_010364289.1 | MULTISPECIES: MFS transporter [Xant | taxID used:135614 OG00963|WP\_017117413.1 | MULTISPECIES: chloride channel prot | taxID used:135614 OG00966|WP\_010364366.1 | MULTISPECIES: membrane protein [Xan | taxID used:135614 OG00967|WP\_026112498.1 | replication-associated recombinatio | taxID used:135614 OG00096|WP\_010365794.1 | excinuclease ABC subunit A, partial | taxID used:135614 OG00971|WP\_010367636.1 | MULTISPECIES: putative lipid II fli | taxID used:135614 OG00974|WP\_010372553.1 | MULTISPECIES: NAD-dependent succina | taxID used:135614 OG00975|WP\_010371376.1 | polynucleotide adenylyltransferase | taxID used:135614 OG00976|WP\_010371860.1 | MULTISPECIES: pyridoxal-phosphate d | taxID used:135614 OG00977|WP\_010367668.1 | dimethylmenaquinone methyltransfera | taxID used:135614 OG00978|WP\_010370090.1 | MULTISPECIES: MATE family efflux tr | taxID used:135614 OG00981|WP\_010363737.1 | MULTISPECIES: DUF1338 domain-contai | taxID used:135614 OG00982|WP\_010371980.1 | MULTISPECIES: HAMP domain-containin | taxID used:135614 OG00984|WP\_010371179.1 | MULTISPECIES: NADH oxidoreductase ( | taxID used:135614 OG00985|WP\_010372811.1 | MULTISPECIES: dihydroorotase [Xanth | taxID used:135614 OG00987|WP\_026112718.1 | MULTISPECIES: ATP-dependent proteas | taxID used:135614 OG00989|WP\_010368217.1 | MULTISPECIES: hypothetical protein | taxID used:135614 OG00994|WP\_010366447.1 | MULTISPECIES: UDP-glucose/GDP-manno | taxID used:135614 OG00995|WP\_010371982.1 | MULTISPECIES: UDP-N-acetylglucosami | taxID used:135614 OG00996|WP\_010366415.1 | adenylosuccinate lyase, partial [Xa | taxID used:135614 OG00997|WP\_010364426.1 | MULTISPECIES: UDP-N-acetylmuramate: | taxID used:135614 
OG01102|WP\_039437140.1 | hypothetical protein [Xanthomonas v | taxID used:135614 OG01179|WP\_010373043.1 | MULTISPECIES: site-specific integra | taxID used:135614 OG00126|WP\_017121663.1 | MULTISPECIES: class I SAM-dependent | taxID used:135614 OG00177|WP\_039431354.1 | MULTISPECIES: ATPase [Xanthomonas] | taxID used:135614 OG01915|WP\_039445628.1 | TIGR03756 family integrating conjug | taxID used:135614 OG02062|WP\_017170370.1 | hypothetical protein, partial [Xant | taxID used:135614 OG02608|WP\_033011972.1 | hypothetical protein [Xanthomonas v | taxID used:135614 OG02717|WP\_017117671.1 | MULTISPECIES: hypothetical protein | taxID used:135614 OG02748|WP\_010365326.1 | YopT-type cysteine protease domain- | taxID used:135614 OG02805|WP\_017117948.1 | hypothetical protein [Xanthomonas v | taxID used:135614 OG03074|WP\_017112227.1 | MULTISPECIES: ABC transporter ATP-b | taxID used:135614 OG03161|WP\_010371649.1 | MULTISPECIES: 3'-5' exonuclease [Xa | taxID used:135614 OG03197|WP\_082320276.1 | MULTISPECIES: GGDEF domain-containi | taxID used:135614 OG03227|WP\_017122811.1 | MULTISPECIES: recombinase family pr | taxID used:135614 OG03382|WP\_039445941.1 | MULTISPECIES: hypothetical protein | taxID used:135614 OG03532|WP\_039443032.1 | MULTISPECIES: GNAT family N-acetylt | taxID used:135614 OG03614|WP\_116896642.1 | hypothetical protein, partial [Xant | taxID used:135614 OG03943|WP\_010367198.1 | MULTISPECIES: hypothetical protein | taxID used:135614 OG03971|WP\_010366350.1 | MULTISPECIES: outer membrane protei | taxID used:135614 OG04076|WP\_039433053.1 | MULTISPECIES: DUF3742 domain-contai | taxID used:135614 OG04092|WP\_010364702.1 | Elastase inhibitor AFLEI Flags: Pre | taxID used:135614 OG04190|WP\_011269728.1 | MULTISPECIES: DUF3085 domain-contai | taxID used:135614 OG04205|WP\_010368851.1 | hypothetical protein, partial [Xant | taxID used:135614 OG04292|WP\_033009084.1 | MULTISPECIES: hypothetical protein | taxID used:135614 OG04410|WP\_010368816.1 | MULTISPECIES: hypothetical protein | taxID used:135614 OG04838|WP\_015471948.1 | MULTISPECIES: AlpA family transcrip | taxID used:135614 OG04866|WP\_010373154.1 | MULTISPECIES: hypothetical protein | taxID used:135614 OG04868|WP\_116896639.1 | antitoxin, partial [Xanthomonas vas | taxID used:135614 OG04897|WP\_010373138.1 | MULTISPECIES: type I toxin-antitoxi | taxID used:135614 OG04934|WP\_015471975.1 | MULTISPECIES: TIGR03745 family inte | taxID used:135614 OG01000|WP\_003486674.1 | MULTISPECIES: preprotein translocas | taxID used:135614 OG01001|WP\_010372160.1 | MULTISPECIES: two-component sensor | taxID used:135614 OG01002|WP\_017116329.1 | amino acid permease, partial [Xanth | taxID used:135614 OG01003|WP\_010369090.1 | MULTISPECIES: MFS transporter [Xant | taxID used:135614 OG01004|WP\_010371500.1 | MULTISPECIES: endonuclease/exonucle | taxID used:135614 OG01005|WP\_010369813.1 | MULTISPECIES: magnesium transporter | taxID used:135614 OG01006|WP\_010366774.1 | MULTISPECIES: metalloprotease PmbA | taxID used:135614 OG01007|WP\_010370858.1 | DUF3999 domain-containing protein, | taxID used:135614 OG01008|WP\_010373683.1 | MULTISPECIES: flagellar protein exp | taxID used:135614 OG01009|WP\_026112558.1 | MULTISPECIES: MFS transporter [Xant | taxID used:135614 OG00100|WP\_010374562.1 | MULTISPECIES: TonB-dependent recept | taxID used:135614 OG01010|WP\_017116753.1 | MULTISPECIES: RIP metalloprotease R | taxID used:135614 OG01011|WP\_010367680.1 | MULTISPECIES: type III secretion sy | taxID used:135614 OG01012|WP\_010368411.1 | MULTISPECIES: aminodeoxychorismate | taxID used:135614 OG01013|WP\_039445328.1 | VirB4 family type IV secretion/conj | taxID used:135614 OG01014|WP\_010365480.1 | MULTISPECIES: dicarboxylate/amino a | taxID used:135614 OG01015|WP\_010365621.1 | MULTISPECIES: phosphomannomutase/ph | taxID used:135614 OG01016|WP\_010364488.1 | MULTISPECIES: M24 family metallopep | taxID used:135614 OG01017|WP\_026112311.1 | MULTISPECIES: MFS transporter [Xant | taxID used:135614 OG01018|WP\_010374761.1 | glycosyltransferase, partial [Xanth | taxID used:135614 OG01019|WP\_010364492.1 | MULTISPECIES: Xaa-Pro dipeptidase [ | taxID used:135614 OG00101|WP\_010371599.1 | MULTISPECIES: glycine dehydrogenase | taxID used:135614 OG01020|WP\_010365977.1 | MULTISPECIES: 23S rRNA (uracil(1939 | taxID used:135614 OG01021|WP\_010376388.1 | MULTISPECIES: membrane protein [Xan | taxID used:135614 OG01022|WP\_054393858.1 | MULTISPECIES: DUF763 domain-contain | taxID used:135614 OG01023|WP\_010371171.1 | MULTISPECIES: NADH-quinone oxidored | taxID used:135614 OG01024|WP\_116896624.1 | hypothetical protein, partial [Xant | taxID used:135614 OG01025|WP\_010368545.1 | MULTISPECIES: phosphate regulon sen | taxID used:135614 OG01026|WP\_010369132.1 | MULTISPECIES: MFS transporter [Xant | taxID used:135614 OG01027|WP\_039438729.1 | filamentous phage phiLf protein I [ | taxID used:135614 OG01028|WP\_010369204.1 | MULTISPECIES: magnesium transporter | taxID used:135614 OG01029|WP\_026112300.1 | MULTISPECIES: NAD(P)/FAD-dependent | taxID used:135614 OG00102|WP\_086019493.1 | MULTISPECIES: membrane protein [Xan | taxID used:135614 OG01030|WP\_010372847.1 | MULTISPECIES: acetylglutamate kinas | taxID used:135614 OG01031|WP\_053012856.1 | hypothetical protein [Xanthomonas v | taxID used:135614 OG01032|WP\_010368970.1 | MULTISPECIES: 3-carboxy-cis,cis-muc | taxID used:135614 OG01033|WP\_010365507.1 | MULTISPECIES: MFS transporter [Xant | taxID used:135614 OG01034|WP\_017116650.1 | MULTISPECIES: sorbosone dehydrogena | taxID used:135614 OG01035|WP\_010371673.1 | MULTISPECIES: lipase [Xanthomonas] | taxID used:135614 OG01036|WP\_010364810.1 | MULTISPECIES: chromosomal replicati | taxID used:135614 OG01037|WP\_010364996.1 | nucleotidyltransferase [Xanthomonas | taxID used:135614 OG01038|WP\_010373410.1 | type VI secretion system baseplate | taxID used:135614 OG01039|WP\_010372519.1 | FAD-binding oxidoreductase, partial | taxID used:135614 OG00103|WP\_017115563.1 | DeoR family transcriptional regulat | taxID used:135614 OG01040|WP\_010363803.1 | MULTISPECIES: HAMP domain-containin | taxID used:135614 OG01041|WP\_010363627.1 | HlyC/CorC family transporter, parti | taxID used:135614 OG01042|WP\_017112958.1 | MULTISPECIES: O-antigen ligase fami | taxID used:135614 OG01043|WP\_010363357.1 | MULTISPECIES: LLM class flavin-depe | taxID used:135614 OG01044|WP\_126922518.1 | IS5/IS1182 family transposase, part | taxID used:135614 OG01045|WP\_080764533.1 | MFS transporter [Xanthomonas vasico | taxID used:135614 OG01046|WP\_010371708.1 | DeoR family transcriptional regulat | taxID used:135614 OG01047|WP\_010366758.1 | dicarboxylate/amino acid:cation sym | taxID used:135614 OG01048|WP\_010373438.1 | MULTISPECIES: sorbosone dehydrogena | taxID used:135614 OG01049|WP\_010366656.1 | MULTISPECIES: phosphoglucosamine mu | taxID used:135614 OG00104|WP\_010368712.1 | MULTISPECIES: TonB-dependent recept | taxID used:135614 OG01050|WP\_017115858.1 | MULTISPECIES: O-acetylhomoserine am | taxID used:135614 OG01051|WP\_017115275.1 | ergothioneine biosynthesis protein | taxID used:135614 OG01052|WP\_010365417.1 | MULTISPECIES: sensor histidine kina | taxID used:135614 OG01053|WP\_010370579.1 | MULTISPECIES: sigma-54-dependent Fi | taxID used:135614 OG01054|WP\_010364364.1 | MULTISPECIES: 3-deoxy-D-manno-octul | taxID used:135614 OG01055|WP\_010371977.1 | MULTISPECIES: sigma-54-dependent Fi | taxID used:135614 OG01056|WP\_033009794.1 | NAD(P)/FAD-dependent oxidoreductase | taxID used:135614 OG01057|WP\_010371842.1 | MULTISPECIES: methyltransferase dom | taxID used:135614 OG01058|WP\_010363992.1 | MULTISPECIES: Tol-Pal system beta p | taxID used:135614 OG01059|WP\_010365503.1 | MULTISPECIES: TRAP transporter larg | taxID used:135614 OG00105|WP\_010364405.1 | MULTISPECIES: TonB-dependent recept | taxID used:135614 OG01060|WP\_010374394.1 | MULTISPECIES: L-fuconate dehydratas | taxID used:135614 OG01061|WP\_017170371.1 | MULTISPECIES: hypothetical protein | taxID used:135614 OG01062|WP\_026113071.1 | rod shape-determining protein MreC, | taxID used:135614 OG01063|WP\_010372660.1 | MULTISPECIES: exodeoxyribonuclease | taxID used:135614 OG01064|WP\_010368451.1 | MULTISPECIES: hypothetical protein | taxID used:135614 OG01065|WP\_010366923.1 | MULTISPECIES: alkaline phosphatase | taxID used:135614 OG01066|WP\_026112809.1 | MULTISPECIES: MFS transporter [Xant | taxID used:135614 OG01067|WP\_010373211.1 | MULTISPECIES: divalent metal cation | taxID used:135614 OG01068|WP\_010363686.1 | MULTISPECIES: serine protease [Xant | taxID used:135614 OG01069|WP\_010363731.1 | amidohydrolase, partial [Xanthomona | taxID used:135614 OG00106|WP\_017116464.1 | MULTISPECIES: TonB-dependent recept | taxID used:135614 OG01070|WP\_054393923.1 | lipid-A-disaccharide synthase, part | taxID used:135614 OG01071|WP\_017117349.1 | MULTISPECIES: hypothetical protein | taxID used:135614 OG01072|WP\_010365643.1 | MULTISPECIES: FAD-binding oxidoredu | taxID used:135614 OG01073|WP\_010366419.1 | MULTISPECIES: HlyC/CorC family tran | taxID used:135614 OG01074|WP\_026112865.1 | MULTISPECIES: tRNA lysidine(34) syn | taxID used:135614 OG01075|WP\_010372473.1 | MULTISPECIES: DUF445 domain-contain | taxID used:135614 OG01076|WP\_010364866.1 | MULTISPECIES: MFS transporter [Xant | taxID used:135614 OG01077|WP\_010366981.1 | siderophore biosynthesis PLP-depend | taxID used:135614 OG01078|WP\_010367925.1 | MULTISPECIES: EscN/YscN/HrcN family | taxID used:135614 OG01079|WP\_010364590.1 | cell envelope biogenesis protein To | taxID used:135614 OG00107|WP\_010368173.1 | MULTISPECIES: TonB-dependent recept | taxID used:135614 OG01080|WP\_017115386.1 | ABC transporter permease, partial [ | taxID used:135614 OG01081|WP\_010364845.1 | MULTISPECIES: tRNA uridine-5-carbox | taxID used:135614 OG01082|WP\_017116370.1 | MULTISPECIES: cell envelope integri | taxID used:135614 OG01083|WP\_010371969.1 | MULTISPECIES: FtsX-like permease fa | taxID used:135614 OG01084|WP\_010369716.1 | MULTISPECIES: MFS transporter [Xant | taxID used:135614 OG01085|WP\_010370487.1 | MULTISPECIES: isocitrate lyase [Xan | taxID used:135614 OG01086|WP\_017115281.1 | MULTISPECIES: 3-phosphoshikimate 1- | taxID used:135614 OG01087|WP\_010369601.1 | hypothetical protein [Xanthomonas v | taxID used:135614 OG01088|WP\_010371477.1 | hydroxylase [Xanthomonas vasicola] | taxID used:135614 OG01089|WP\_010370038.1 | MULTISPECIES: 16S rRNA (cytosine(96 | taxID used:135614 OG00108|WP\_017116576.1 | MULTISPECIES: DUF2339 domain-contai | taxID used:135614 OG01090|WP\_010369005.1 | MULTISPECIES: MFS transporter [Xant | taxID used:135614 OG01091|WP\_010375609.1 | MULTISPECIES: peptidoglycan-binding | taxID used:135614 OG01092|WP\_010364535.1 | MULTISPECIES: citrate synthase [Xan | taxID used:135614 OG01093|WP\_039445437.1 | filamentous phage phiLf protein I, | taxID used:135614 OG01094|WP\_039442440.1 | hypothetical protein [Xanthomonas v | taxID used:135614 OG01095|WP\_010365705.1 | MULTISPECIES: GTPase HflX [Xanthomo | taxID used:135614 OG01096|WP\_010366079.1 | MULTISPECIES: class I SAM-dependent | taxID used:135614 OG01097|WP\_010366457.1 | MULTISPECIES: L-fucose:H+ symporter | taxID used:135614 OG01098|WP\_010373409.1 | MULTISPECIES: type VI secretion sys | taxID used:135614 OG01099|WP\_017112670.1 | efflux RND transporter periplasmic | taxID used:135614 OG00109|WP\_039431225.1 | MULTISPECIES: adhesin [Xanthomonas] | taxID used:135614 OG00010|WP\_010363272.1 | DUF3320 domain-containing protein [ | taxID used:135614 OG01100|WP\_010368616.1 | MULTISPECIES: membrane protein [Xan | taxID used:135614 OG01101|WP\_010377068.1 | MULTISPECIES: dicarboxylate/amino a | taxID used:135614 OG01103|WP\_010369736.1 | MULTISPECIES: acyl-CoA synthetase [ | taxID used:135614 OG01104|WP\_010374398.1 | MULTISPECIES: L-fucose:H+ symporter | taxID used:135614 OG01105|WP\_010373340.1 | nucleotide sugar dehydrogenase, par | taxID used:135614 OG01106|WP\_010372559.1 | MULTISPECIES: DUF4105 domain-contai | taxID used:135614 OG01107|WP\_010364452.1 | glutamate-1-semialdehyde 2,1-aminom | taxID used:135614 OG01108|WP\_010368527.1 | MULTISPECIES: trigger factor [Xanth | taxID used:135614 OG01109|WP\_054393967.1 | MULTISPECIES: NAD(P)/FAD-dependent | taxID used:135614 OG00110|WP\_017116047.1 | serine/threonine protein kinase, pa | taxID used:135614 OG01110|WP\_010363761.1 | MULTISPECIES: amino acid permease [ | taxID used:135614 OG01111|WP\_010372281.1 | MULTISPECIES: carbohydrate porin [X | taxID used:135614 OG01112|WP\_010372121.1 | MULTISPECIES: D-amino acid dehydrog | taxID used:135614 OG01113|WP\_010368184.1 | MULTISPECIES: poly-beta-1,6 N-acety | taxID used:135614 OG01114|WP\_010366554.1 | kynureninase, partial [Xanthomonas | taxID used:135614 OG01115|WP\_116896648.1 | hypothetical protein [Xanthomonas v | taxID used:135614 OG01116|WP\_010368449.1 | MULTISPECIES: adenylosuccinate synt | taxID used:135614 OG01117|WP\_026112896.1 | diguanylate cyclase response regula | taxID used:135614 OG01118|WP\_010373633.1 | MULTISPECIES: flagellar protein [Xa | taxID used:135614 OG01119|WP\_010365207.1 | MULTISPECIES: serine--tRNA ligase [ | taxID used:135614 OG00111|WP\_010367825.1 | MULTISPECIES: valine--tRNA ligase [ | taxID used:135614 OG01120|WP\_010372592.1 | MULTISPECIES: cytochrome bc complex | taxID used:135614 OG01121|WP\_010367182.1 | threonine synthase, partial [Xantho | taxID used:135614 OG01122|WP\_010365994.1 | MULTISPECIES: FAD-dependent monooxy | taxID used:135614 OG01123|WP\_010370621.1 | MULTISPECIES: acetyl-CoA C-acetyltr | taxID used:135614 OG01124|WP\_010374868.1 | MULTISPECIES: tryptophan--tRNA liga | taxID used:135614 OG01125|WP\_039432516.1 | multidrug transporter, partial [Xan | taxID used:135614 OG01126|WP\_010370034.1 | MULTISPECIES: O-antigen ligase fami | taxID used:135614 OG01127|WP\_116645396.1 | HlyD family efflux transporter peri | taxID used:135614 OG01128|WP\_010368119.1 | phosphoribosylamine--glycine ligase | taxID used:135614 OG01129|WP\_010374708.1 | MULTISPECIES: MFS transporter [Xant | taxID used:135614 OG00112|WP\_017116633.1 | MULTISPECIES: DUF4135 domain-contai | taxID used:135614 OG01130|WP\_010363907.1 | MULTISPECIES: NCS2 family permease | taxID used:135614 OG01131|WP\_010368578.1 | bifunctional tetrahydrofolate synth | taxID used:135614 OG01132|WP\_080764528.1 | acyl-CoA dehydrogenase [Xanthomonas | taxID used:135614 OG01133|WP\_080765164.1 | MULTISPECIES: efflux RND transporte | taxID used:135614 OG01134|WP\_010374510.1 | MULTISPECIES: D-galactonate dehydra | taxID used:135614 OG01135|WP\_010371564.1 | MULTISPECIES: HAMP domain-containin | taxID used:135614 OG01136|WP\_010367329.1 | MULTISPECIES: YihY family inner mem | taxID used:135614 OG01137|WP\_010370752.1 | MULTISPECIES: lytic murein transgly | taxID used:135614 OG01138|WP\_039432410.1 | MULTISPECIES: flagellar hook-length | taxID used:135614 OG01139|WP\_010367540.1 | Na+ dependent nucleoside transporte | taxID used:135614 OG00113|WP\_010368326.1 | MULTISPECIES: insulinase family pro | taxID used:135614 OG01140|WP\_010372855.1 | argininosuccinate lyase, partial [X | taxID used:135614 OG01141|WP\_033485492.1 | MULTISPECIES: hypothetical protein | taxID used:135614 OG01142|WP\_010367319.1 | MULTISPECIES: glutamyl-tRNA reducta | taxID used:135614 OG01143|WP\_039437452.1 | aminotransferase class V-fold PLP-d | taxID used:135614 OG01144|WP\_010366082.1 | MULTISPECIES: FAD-dependent oxidore | taxID used:135614 OG01145|WP\_010371742.1 | MULTISPECIES: histidine-type phosph | taxID used:135614 OG01146|WP\_010365670.1 | phosphopyruvate hydratase [Xanthomo | taxID used:135614 OG01147|WP\_039441795.1 | MULTISPECIES: cytochrome P450 [Xant | taxID used:135614 OG01148|WP\_003483788.1 | MULTISPECIES: ATP-dependent Clp pro | taxID used:135614 OG01149|WP\_017116880.1 | allantoate amidohydrolase, partial | taxID used:135614 OG00114|WP\_010365817.1 | MULTISPECIES: isoleucine--tRNA liga | taxID used:135614 OG01150|WP\_010367171.1 | MULTISPECIES: histidinol dehydrogen | taxID used:135614 OG01151|WP\_010364293.1 | MULTISPECIES: cardiolipin synthase | taxID used:135614 OG01152|WP\_010370063.1 | DUF4339 domain-containing protein [ | taxID used:135614 OG01153|WP\_010365392.1 | MULTISPECIES: sigma-54-dependent Fi | taxID used:135614 OG01154|WP\_010369503.1 | MULTISPECIES: diguanylate phosphodi | taxID used:135614 OG01155|WP\_010365518.1 | MULTISPECIES: pyridoxal phosphate-d | taxID used:135614 OG01156|WP\_010372931.1 | MULTISPECIES: SMC-Scp complex subun | taxID used:135614 OG01157|WP\_010363503.1 | hypothetical protein, partial [Xant | taxID used:135614 OG01158|WP\_010366744.1 | MULTISPECIES: FAD-dependent oxidore | taxID used:135614 OG01159|WP\_010372653.1 | MULTISPECIES: virulence factor [Xan | taxID used:135614 OG00115|WP\_017115454.1 | MULTISPECIES: glycoside hydrolase f | taxID used:135614 OG01160|WP\_010367488.1 | MULTISPECIES: HAMP domain-containin | taxID used:135614 OG01161|WP\_017116967.1 | MULTISPECIES: type II secretion sys | taxID used:135614 OG01162|WP\_010371421.1 | flavodoxin-dependent (E)-4-hydroxy- | taxID used:135614 OG01163|WP\_010372514.1 | MULTISPECIES: MFS transporter [Xant | taxID used:135614 OG01164|WP\_010365349.1 | MULTISPECIES: DUF3526 domain-contai | taxID used:135614 OG01165|WP\_039435374.1 | ABC transporter permease [Xanthomon | taxID used:135614 OG01166|WP\_010368190.1 | MULTISPECIES: glucose/galactose MFS | taxID used:135614 OG01167|WP\_010371431.1 | MULTISPECIES: MFS transporter [Xant | taxID used:135614 OG01168|WP\_010366884.1 | MULTISPECIES: potassium transporter | taxID used:135614 OG01169|WP\_010366703.1 | MULTISPECIES: membrane protein [Xan | taxID used:135614 OG00116|WP\_017116155.1 | MULTISPECIES: 2-oxoglutarate dehydr | taxID used:135614 OG01170|WP\_010368102.1 | MULTISPECIES: lipase [Xanthomonas] | taxID used:135614 OG01171|WP\_010365514.1 | MULTISPECIES: flavohemoglobin expre | taxID used:135614 OG01172|WP\_010369003.1 | MULTISPECIES: DUF3391 domain-contai | taxID used:135614 OG01173|WP\_010367986.1 | MULTISPECIES: ATP-dependent helicas | taxID used:135614 OG01174|WP\_010370615.1 | MULTISPECIES: porphyrin biosynthesi | taxID used:135614 OG01175|WP\_026112776.1 | MULTISPECIES: undecaprenyldiphospho | taxID used:135614 OG01176|WP\_010373342.1 | MULTISPECIES: aminoacetone oxidase | taxID used:135614 OG01177|WP\_010371845.1 | ATP-binding cassette domain-contain | taxID used:135614 OG01178|WP\_010370793.1 | MULTISPECIES: TRAP transporter larg | taxID used:135614 OG00117|WP\_026112084.1 | hybrid sensor histidine kinase/resp | taxID used:135614 OG01180|WP\_010368529.1 | MULTISPECIES: PAS domain-containing | taxID used:135614 OG01181|WP\_010366492.1 | porin, partial [Xanthomonas vasicol | taxID used:135614 OG01182|WP\_010367709.1 | MULTISPECIES: serine hydroxymethylt | taxID used:135614 OG01183|WP\_010365541.1 | amidohydrolase [Xanthomonas vasicol | taxID used:135614 OG01184|WP\_010368565.1 | MULTISPECIES: peptidase [Xanthomona | taxID used:135614 OG01185|WP\_123180759.1 | exodeoxyribonuclease V subunit alph | taxID used:135614 OG01186|WP\_026113095.1 | ATP-dependent helicase HrpB, partia | taxID used:135614 OG01187|WP\_010374342.1 | MULTISPECIES: HlyD family efflux tr | taxID used:135614 OG01188|WP\_017116056.1 | MULTISPECIES: molybdopterin molybde | taxID used:135614 OG01189|WP\_010366917.1 | MULTISPECIES: hypothetical protein | taxID used:135614 OG00118|WP\_010370347.1 | type IV secretion protein Rhs [Xant | taxID used:135614 OG01190|WP\_039434483.1 | membrane protein [Xanthomonas vasic | taxID used:135614 OG01191|WP\_010370360.1 | MULTISPECIES: alanine--glyoxylate a | taxID used:135614 OG01192|WP\_010366459.1 | MULTISPECIES: AGE family epimerase/ | taxID used:135614 OG01193|WP\_054393993.1 | MULTISPECIES: Bcr/CflA family efflu | taxID used:135614 OG01194|WP\_010363454.1 | MULTISPECIES: FAD-binding oxidoredu | taxID used:135614 OG01195|WP\_017116735.1 | 2OG-Fe(II) oxygenase [Xanthomonas v | taxID used:135614 OG01196|WP\_010364250.1 | MULTISPECIES: tetracycline resistan | taxID used:135614 OG01197|WP\_010371963.1 | MULTISPECIES: efflux RND transporte | taxID used:135614 OG01198|WP\_026112746.1 | membrane dipeptidase, partial [Xant | taxID used:135614 OG01199|WP\_010369231.1 | MULTISPECIES: UDP-N-acetylglucosami | taxID used:135614 OG00119|WP\_026112100.1 | MULTISPECIES: TonB-dependent recept | taxID used:135614 OG00011|WP\_010372216.1 | NAD-glutamate dehydrogenase, partia | taxID used:135614 OG01200|WP\_010364418.1 | MULTISPECIES: 6-phosphofructokinase | taxID used:135614 OG01201|WP\_010366747.1 | MULTISPECIES: DUF418 domain-contain | taxID used:135614 OG01202|WP\_010374741.1 | MULTISPECIES: bifunctional phosphop | taxID used:135614 OG01203|WP\_010374604.1 | MULTISPECIES: GAF domain-containing | taxID used:135614 OG01204|WP\_010368409.1 | MULTISPECIES: beta-ketoacyl-[acyl-c | taxID used:135614 OG01205|WP\_017112838.1 | MULTISPECIES: putative DNA modifica | taxID used:135614 OG01206|WP\_010369296.1 | MULTISPECIES: cysteine desulfurase | taxID used:135614 OG01207|WP\_086019581.1 | MULTISPECIES: MFS transporter [Xant | taxID used:135614 OG01208|WP\_086019477.1 | MULTISPECIES: RtcB family protein [ | taxID used:135614 OG01209|WP\_010366591.1 | MULTISPECIES: pyridoxal phosphate-d | taxID used:135614 OG00120|WP\_017118940.1 | phage tail tape measure protein [Xa | taxID used:135614 OG01210|WP\_054393941.1 | MULTISPECIES: siderophore biosynthe | taxID used:135614 OG01212|WP\_010369297.1 | MULTISPECIES: Fe-S cluster assembly | taxID used:135614 OG01213|WP\_017113128.1 | MULTISPECIES: membrane protein [Xan | taxID used:135614 OG01214|WP\_010363659.1 | MULTISPECIES: O-antigen translocase | taxID used:135614 OG01215|WP\_039445626.1 | MULTISPECIES: integrating conjugati | taxID used:135614 OG01216|WP\_010373234.1 | MULTISPECIES: class III poly(R)-hyd | taxID used:135614 OG01217|WP\_116896635.1 | hypothetical protein, partial [Xant | taxID used:135614 OG01218|WP\_010374543.1 | MULTISPECIES: endo-1,4-beta-xylanas | taxID used:135614 OG01219|WP\_010374344.1 | MULTISPECIES: TolC family protein [ | taxID used:135614 OG00121|WP\_039443906.1 | helicase IV [Xanthomonas vasicola] | taxID used:135614 OG01220|WP\_010378794.1 | MULTISPECIES: lipoprotein-releasing | taxID used:135614 OG01221|WP\_017116291.1 | MULTISPECIES: HlyD family efflux tr | taxID used:135614 OG01222|WP\_010363732.1 | efflux RND transporter periplasmic | taxID used:135614 OG01223|WP\_010367324.1 | hypothetical protein, partial [Xant | taxID used:135614 OG01224|WP\_017112234.1 | MULTISPECIES: DUF819 domain-contain | taxID used:135614 OG01225|WP\_003485272.1 | MULTISPECIES: cell division protein | taxID used:135614 OG01226|WP\_010365595.1 | MULTISPECIES: threonine/serine expo | taxID used:135614 OG01227|WP\_010365008.1 | MULTISPECIES: chemotaxis protein Ch | taxID used:135614 OG01228|WP\_010379468.1 | MULTISPECIES: ATPase [Xanthomonas] | taxID used:135614 OG01229|WP\_010363645.1 | hypothetical protein, partial [Xant | taxID used:135614 OG00122|WP\_039442350.1 | autotransporter domain-containing p | taxID used:135614 OG01230|WP\_010370498.1 | MULTISPECIES: cation:proton antipor | taxID used:135614 OG01231|WP\_010365932.1 | MULTISPECIES: arabinogalactan endo- | taxID used:135614 OG01232|WP\_087911144.1 | peptidase [Xanthomonas vasicola] [G | taxID used:135614 OG01233|WP\_017117441.1 | MULTISPECIES: argininosuccinate syn | taxID used:135614 OG01234|WP\_010372825.1 | MULTISPECIES: MFS transporter [Xant | taxID used:135614 OG01235|WP\_010367620.1 | MULTISPECIES: cell division protein | taxID used:135614 OG01236|WP\_026112828.1 | MULTISPECIES: amidohydrolase [Xanth | taxID used:135614 OG01237|WP\_010373598.1 | sensor histidine kinase, partial [X | taxID used:135614 OG01238|WP\_010371974.1 | MULTISPECIES: FtsX-like permease fa | taxID used:135614 OG01239|WP\_010363943.1 | MULTISPECIES: MFS transporter [Xant | taxID used:135614 OG00123|WP\_017115948.1 | MULTISPECIES: monovalent cation/H+ | taxID used:135614 OG01240|WP\_010379894.1 | MULTISPECIES: hypothetical protein | taxID used:135614 OG01241|WP\_010370732.1 | MULTISPECIES: aminotransferase [Xan | taxID used:135614 OG01242|WP\_010367140.1 | MULTISPECIES: phosphoglycerate dehy | taxID used:135614 OG01243|WP\_010368176.1 | MULTISPECIES: multifunctional CCA a | taxID used:135614 OG01244|WP\_039431361.1 | hypothetical protein [Xanthomonas v | taxID used:135614 OG01245|WP\_010373610.1 | MULTISPECIES: flagellar hook protei | taxID used:135614 OG01247|WP\_010379565.1 | MULTISPECIES: IS4 family transposas | taxID used:135614 OG01248|WP\_010368265.1 | MULTISPECIES: phosphonopyruvate dec | taxID used:135614 OG01249|WP\_086019498.1 | MULTISPECIES: HAMP domain-containin | taxID used:135614 OG00124|WP\_010369643.1 | DNA polymerase I, partial [Xanthomo | taxID used:135614 OG01250|WP\_010373246.1 | MULTISPECIES: FAD-binding oxidoredu | taxID used:135614 OG01251|WP\_010368298.1 | MULTISPECIES: pyridoxal phosphate-d | taxID used:135614 OG01252|WP\_010373542.1 | MULTISPECIES: 23S rRNA (adenine(250 | taxID used:135614 OG01253|WP\_010376381.1 | MULTISPECIES: peptidase M23 [Xantho | taxID used:135614 OG01254|WP\_010363526.1 | MULTISPECIES: glycosyltransferase f | taxID used:135614 OG01255|WP\_010368235.1 | MULTISPECIES: D-alanyl-D-alanine ca | taxID used:135614 OG01256|WP\_039439306.1 | SGNH/GDSL hydrolase family protein | taxID used:135614 OG01257|WP\_010374848.1 | MULTISPECIES: efflux RND transporte | taxID used:135614 OG01258|WP\_017116375.1 | MULTISPECIES: aspartate aminotransf | taxID used:135614 OG01259|WP\_010372869.1 | MULTISPECIES: glutamate-5-semialdeh | taxID used:135614 OG00125|WP\_010367562.1 | TonB-dependent receptor, partial [X | taxID used:135614 OG01260|WP\_010365414.1 | FAD-binding oxidoreductase, partial | taxID used:135614 OG01261|WP\_017118714.1 | zonular occludens toxin, partial [X | taxID used:135614 OG01262|WP\_010367557.1 | MULTISPECIES: methionine adenosyltr | taxID used:135614 OG01263|WP\_010372969.1 | MULTISPECIES: TraB/GumN family prot | taxID used:135614 OG01264|WP\_010366666.1 | tryptophan synthase subunit beta, p | taxID used:135614 OG01265|WP\_010363442.1 | MULTISPECIES: aminopeptidase P fami | taxID used:135614 OG01266|WP\_039437058.1 | sensor histidine kinase [Xanthomona | taxID used:135614 OG01267|WP\_084818983.1 | hypothetical protein [Xanthomonas v | taxID used:135614 OG01268|WP\_010364434.1 | MULTISPECIES: aspartate aminotransf | taxID used:135614 OG01269|WP\_010366617.1 | MULTISPECIES: imidazolonepropionase | taxID used:135614 OG01270|WP\_010373528.1 | MULTISPECIES: molybdopterin molybde | taxID used:135614 OG01271|WP\_010367044.1 | enoyl-[acyl-carrier-protein] reduct | taxID used:135614 OG01272|WP\_010372890.1 | MULTISPECIES: homoserine O-acetyltr | taxID used:135614 OG01273|WP\_010365377.1 | MULTISPECIES: UDP-galactopyranose m | taxID used:135614 OG01274|WP\_010365197.1 | MULTISPECIES: prephenate dehydratas | taxID used:135614 OG01275|WP\_017170374.1 | MULTISPECIES: hypothetical protein | taxID used:135614 OG01276|WP\_017115227.1 | LytTR family transcriptional regula | taxID used:135614 OG01277|WP\_010365501.1 | MULTISPECIES: porin [Xanthomonas] [ | taxID used:135614 OG01278|WP\_010365378.1 | MULTISPECIES: glycosyltransferase f | taxID used:135614 OG01279|WP\_010373534.1 | MULTISPECIES: outer membrane protei | taxID used:135614 OG00127|WP\_010374443.1 | MULTISPECIES: TonB-dependent recept | taxID used:135614 OG01280|WP\_010374689.1 | MULTISPECIES: tyrosine--tRNA ligase | taxID used:135614 OG01281|WP\_010368601.1 | MULTISPECIES: glycine C-acetyltrans | taxID used:135614 OG01282|WP\_010368457.1 | DUF4172 domain-containing protein [ | taxID used:135614 OG01283|WP\_026112856.1 | MULTISPECIES: DUF2974 domain-contai | taxID used:135614 OG01284|WP\_026112729.1 | beta-ketoacyl-[acyl-carrier-protein | taxID used:135614 OG01285|WP\_010366395.1 | dihydrolipoyllysine-residue succiny | taxID used:135614 OG01286|WP\_017118858.1 | MULTISPECIES: pyridoxal phosphate-d | taxID used:135614 OG01287|WP\_010366882.1 | MULTISPECIES: HAMP domain-containin | taxID used:135614 OG01288|WP\_010364119.1 | hypothetical protein [Xanthomonas v | taxID used:135614 OG01289|WP\_010366048.1 | MULTISPECIES: MFS transporter [Xant | taxID used:135614 OG00128|WP\_026112731.1 | MULTISPECIES: bifunctional [glutama | taxID used:135614 OG01290|WP\_054393994.1 | MULTISPECIES: HlyD family efflux tr | taxID used:135614 OG01291|WP\_017121665.1 | MULTISPECIES: GIY-YIG nuclease fami | taxID used:135614 OG01292|WP\_010366187.1 | MULTISPECIES: 2,3,4,5-tetrahydropyr | taxID used:135614 OG01293|WP\_010379849.1 | MULTISPECIES: MFS transporter [Xant | taxID used:135614 OG01294|WP\_010373550.1 | acetyl-CoA C-acyltransferase, parti | taxID used:135614 OG01295|WP\_080764660.1 | histidine kinase [Xanthomonas vasic | taxID used:135614 OG01296|WP\_010363346.1 | MULTISPECIES: DUF1311 domain-contai | taxID used:135614 OG01297|WP\_010369571.1 | MULTISPECIES: MexE family multidrug | taxID used:135614 OG01298|WP\_039441382.1 | MULTISPECIES: chromosome segregatio | taxID used:135614 OG01299|WP\_087979042.1 | MULTISPECIES: hypothetical protein | taxID used:135614 OG00129|WP\_010373588.1 | MULTISPECIES: GGDEF domain-containi | taxID used:135614 OG00012|WP\_054393954.1 | MULTISPECIES: alpha-2-macroglobulin | taxID used:135614 OG01300|WP\_010367736.1 | MULTISPECIES: nicotinate phosphorib | taxID used:135614 OG01302|WP\_010373627.1 | MULTISPECIES: flagellar hook-associ | taxID used:135614 OG01303|WP\_010368737.1 | MULTISPECIES: O-succinylhomoserine | taxID used:135614 OG01304|WP\_017115492.1 | MULTISPECIES: nucleoside hydrolase | taxID used:135614 OG01305|WP\_010367780.1 | MULTISPECIES: type II secretion sys | taxID used:135614 OG01306|WP\_010371857.1 | MULTISPECIES: cystathionine gamma-s | taxID used:135614 OG01307|WP\_010369789.1 | MULTISPECIES: beta-ketoacyl-[acyl-c | taxID used:135614 OG01308|WP\_010368931.1 | MULTISPECIES: 8-amino-7-oxononanoat | taxID used:135614 OG01309|WP\_010367294.1 | elongation factor Tu, partial [Xant | taxID used:135614 OG00130|WP\_010367038.1 | MULTISPECIES: TonB-dependent recept | taxID used:135614 OG01310|WP\_010376339.1 | elongation factor Tu, partial [Xant | taxID used:135614 OG01311|WP\_017115234.1 | MULTISPECIES: PLP-dependent aminotr | taxID used:135614 OG01312|WP\_039442467.1 | hypothetical protein [Xanthomonas v | taxID used:135614 OG01313|WP\_010371506.1 | MULTISPECIES: formate-dependent pho | taxID used:135614 OG01314|WP\_010371541.1 | MULTISPECIES: sensor histidine kina | taxID used:135614 OG01315|WP\_010368426.1 | 2-methylaconitate cis-trans isomera | taxID used:135614 OG01316|WP\_017116174.1 | MULTISPECIES: HlyD family secretion | taxID used:135614 OG01317|WP\_010365376.1 | MULTISPECIES: beta-glucosidase [Xan | taxID used:135614 OG01318|WP\_010367069.1 | MULTISPECIES: aspartate/tyrosine/ar | taxID used:135614 OG01319|WP\_010370890.1 | uroporphyrinogen-III C-methyltransf | taxID used:135614 OG00131|WP\_086020502.1 | methyl-accepting chemotaxis protein | taxID used:135614 OG01320|WP\_010369130.1 | MULTISPECIES: glutathionylspermidin | taxID used:135614 OG01321|WP\_017112489.1 | MULTISPECIES: hypothetical protein | taxID used:135614 OG01322|WP\_010367455.1 | MULTISPECIES: 2-octaprenyl-6-methox | taxID used:135614 OG01323|WP\_010370903.1 | MULTISPECIES: sensor histidine kina | taxID used:135614 OG01324|WP\_033004672.1 | MULTISPECIES: chemotaxis protein [X | taxID used:135614 OG01325|WP\_010368975.1 | MULTISPECIES: 3-oxoadipyl-CoA thiol | taxID used:135614 OG01326|WP\_010373507.1 | MULTISPECIES: glutathione-dependent | taxID used:135614 OG01327|WP\_010372702.1 | NAD(P)/FAD-dependent oxidoreductase | taxID used:135614 OG01328|WP\_010365939.1 | MULTISPECIES: acyl-CoA dehydrogenas | taxID used:135614 OG01329|WP\_010369992.1 | DUF4102 domain-containing protein [ | taxID used:135614 OG00132|WP\_010366460.1 | methionine synthase [Xanthomonas va | taxID used:135614 OG01330|WP\_017116966.1 | membrane protein [Xanthomonas vasic | taxID used:135614 OG01331|WP\_010368991.1 | MULTISPECIES: 4-hydroxybenzoate 3-m | taxID used:135614 OG01332|WP\_010364859.1 | MULTISPECIES: class I SAM-dependent | taxID used:135614 OG01333|WP\_010365482.1 | MULTISPECIES: porin [Xanthomonas] [ | taxID used:135614 OG01334|WP\_010374907.1 | MULTISPECIES: DUF1501 domain-contai | taxID used:135614 OG01335|WP\_010364796.1 | MULTISPECIES: tetratricopeptide rep | taxID used:135614 OG01336|WP\_010370719.1 | MULTISPECIES: aminotransferase clas | taxID used:135614 OG01337|WP\_010373020.1 | MULTISPECIES: lipopolysaccharide as | taxID used:135614 OG01338|WP\_010366225.1 | MULTISPECIES: HlyD family efflux tr | taxID used:135614 OG01339|WP\_010368421.1 | MULTISPECIES: 2-methylcitrate synth | taxID used:135614 OG00133|WP\_010365292.1 | MULTISPECIES: aconitate hydratase A | taxID used:135614 OG01340|WP\_010364873.1 | MULTISPECIES: CdaR family transcrip | taxID used:135614 OG01341|WP\_026112535.1 | MULTISPECIES: hypothetical protein | taxID used:135614 OG01342|WP\_010364700.1 | MULTISPECIES: DNA topoisomerase IB | taxID used:135614 OG01343|WP\_010371534.1 | MULTISPECIES: polyketide cyclase [X | taxID used:135614 OG01344|WP\_106888028.1 | ergothioneine biosynthesis protein | taxID used:135614 OG01345|WP\_010366317.1 | MexE family multidrug efflux RND tr | taxID used:135614 OG01346|WP\_010373665.1 | aromatic ring-hydroxylating dioxyge | taxID used:135614 OG01347|WP\_010368204.1 | MULTISPECIES: class I SAM-dependent | taxID used:135614 OG01348|WP\_010363769.1 | MULTISPECIES: PQQ-dependent sugar d | taxID used:135614 OG01349|WP\_010366985.1 | MULTISPECIES: MFS transporter [Xant | taxID used:135614 OG00134|WP\_010367615.1 | MULTISPECIES: preprotein translocas | taxID used:135614 OG01350|WP\_010365379.1 | MULTISPECIES: UDP-glucose 4-epimera | taxID used:135614 OG01351|WP\_010369878.1 | MULTISPECIES: ABC transporter perme | taxID used:135614 OG01352|WP\_010365943.1 | MULTISPECIES: enoyl-CoA hydratase/i | taxID used:135614 OG01353|WP\_087911094.1 | zonular occludens toxin [Xanthomona | taxID used:135614 OG01354|WP\_026112862.1 | MFS transporter, partial [Xanthomon | taxID used:135614 OG01355|WP\_010370560.1 | MULTISPECIES: Fic family protein [X | taxID used:135614 OG01356|WP\_050557299.1 | MULTISPECIES: ROK family protein [X | taxID used:135614 OG01357|WP\_017112297.1 | MULTISPECIES: sensor histidine kina | taxID used:135614 OG01358|WP\_010367750.1 | MULTISPECIES: aminoglycoside phosph | taxID used:135614 OG01359|WP\_010366294.1 | MULTISPECIES: acyl-CoA dehydrogenas | taxID used:135614 OG00135|WP\_010364448.1 | MULTISPECIES: helix-turn-helix tran | taxID used:135614 OG01360|WP\_010365368.1 | MULTISPECIES: glutathione-dependent | taxID used:135614 OG01361|WP\_010364501.1 | MULTISPECIES: radical SAM family he | taxID used:135614 OG01362|WP\_017115662.1 | MULTISPECIES: LysM peptidoglycan-bi | taxID used:135614 OG01363|WP\_010373629.1 | MULTISPECIES: flagellin [Xanthomona | taxID used:135614 OG01364|WP\_010373152.1 | MULTISPECIES: ATP-dependent DNA hel | taxID used:135614 OG01365|WP\_010368193.1 | MULTISPECIES: DUF1624 domain-contai | taxID used:135614 OG01366|WP\_010366046.1 | MULTISPECIES: alkene reductase [Xan | taxID used:135614 OG01367|WP\_010374662.1 | MULTISPECIES: heme A synthase [Xant | taxID used:135614 OG01368|WP\_086019485.1 | IS3 family transposase, partial [Xa | taxID used:135614 OG01369|WP\_100243791.1 | MULTISPECIES: IS3 family transposas | taxID used:135614 OG00136|WP\_026112760.1 | TonB-dependent receptor, partial [X | taxID used:135614 OG01370|WP\_010371312.1 | MULTISPECIES: TrbI/VirB10 family pr | taxID used:135614 OG01371|WP\_080762927.1 | 2-aminoethylphosphonate--pyruvate t | taxID used:135614 OG01372|WP\_026112737.1 | MULTISPECIES: LacI family DNA-bindi | taxID used:135614 OG01373|WP\_010367453.1 | 2-octaprenyl-3-methyl-6-methoxy-1,4 | taxID used:135614 OG01374|WP\_017115509.1 | hypothetical protein [Xanthomonas v | taxID used:135614 OG01376|WP\_010368113.1 | MULTISPECIES: MFS transporter [Xant | taxID used:135614 OG01377|WP\_010364227.1 | MULTISPECIES: hypothetical protein | taxID used:135614 OG01378|WP\_010366014.1 | MULTISPECIES: acetyl-CoA C-acyltran | taxID used:135614 OG01379|WP\_010366159.1 | 1-deoxy-D-xylulose-5-phosphate redu | taxID used:135614 OG00137|WP\_017117099.1 | MULTISPECIES: polysaccharide biosyn | taxID used:135614 OG01380|WP\_010373622.1 | MULTISPECIES: flagellar assembly pe | taxID used:135614 OG01381|WP\_054393979.1 | MULTISPECIES: isovaleryl-CoA dehydr | taxID used:135614 OG01382|WP\_010372314.1 | MULTISPECIES: tRNA guanosine(34) tr | taxID used:135614 OG01383|WP\_054393931.1 | MULTISPECIES: acyl-CoA dehydrogenas | taxID used:135614 OG01384|WP\_054393991.1 | pilus assembly protein PilW [Xantho | taxID used:135614 OG01385|WP\_017116212.1 | MULTISPECIES: pyridoxal phosphate-d | taxID used:135614 OG01386|WP\_017115986.1 | MULTISPECIES: D-galactonate dehydra | taxID used:135614 OG01387|WP\_017170413.1 | MULTISPECIES: hypothetical protein | taxID used:135614 OG01388|WP\_010367057.1 | MULTISPECIES: alpha-hydroxy-acid ox | taxID used:135614 OG01389|WP\_010366204.1 | MULTISPECIES: ADP-forming succinate | taxID used:135614 OG00138|WP\_017113834.1 | MULTISPECIES: glycoside hydrolase f | taxID used:135614 OG01390|WP\_017115290.1 | DUF4236 domain-containing protein, | taxID used:135614 OG01391|WP\_010365884.1 | MULTISPECIES: PAS domain-containing | taxID used:135614 OG01392|WP\_010374852.1 | MULTISPECIES: ABC transporter perme | taxID used:135614 OG01393|WP\_017116067.1 | MULTISPECIES: N-acetyltransferase [ | taxID used:135614 OG01394|WP\_010363508.1 | ceramide glucosyltransferase [Xanth | taxID used:135614 OG01395|WP\_039432029.1 | benzoate transporter BenE [Xanthomo | taxID used:135614 OG01396|WP\_010370809.1 | MULTISPECIES: aldo/keto reductase [ | taxID used:135614 OG01397|WP\_010364056.1 | MULTISPECIES: phage tail sheath pro | taxID used:135614 OG01398|WP\_010369244.1 | MULTISPECIES: DUF2066 domain-contai | taxID used:135614 OG01399|WP\_038894056.1 | MULTISPECIES: hypothetical protein | taxID used:135614 OG00139|WP\_010364582.1 | MULTISPECIES: TonB-dependent recept | taxID used:135614 OG00013|WP\_116896653.1 | hypothetical protein, partial [Xant | taxID used:135614 OG01400|WP\_010380300.1 | MULTISPECIES: phospholipase [Xantho | taxID used:135614 OG01401|WP\_010370862.1 | MULTISPECIES: phosphoglycerate kina | taxID used:135614 OG01402|WP\_010369246.1 | MULTISPECIES: AI-2E family transpor | taxID used:135614 OG01403|WP\_010363532.1 | MULTISPECIES: glycosyltransferase f | taxID used:135614 OG01404|WP\_010368238.1 | MULTISPECIES: lytic murein transgly | taxID used:135614 OG01405|WP\_082337890.1 | molybdopterin-synthase adenylyltran | taxID used:135614 OG01406|WP\_010373581.1 | tRNA 2-thiouridine(34) synthase Mnm | taxID used:135614 OG01407|WP\_010372148.1 | MULTISPECIES: motility protein MotB | taxID used:135614 OG01408|WP\_010374787.1 | MULTISPECIES: acyl-CoA desaturase [ | taxID used:135614 OG01409|WP\_010369335.1 | MULTISPECIES: type IV pilus twitchi | taxID used:135614 OG00140|WP\_010368648.1 | glycoside hydrolase family 2 protei | taxID used:135614 OG01410|WP\_010364869.1 | MULTISPECIES: glycerate kinase [Xan | taxID used:135614 OG01411|WP\_010372544.1 | MULTISPECIES: polyamine ABC transpo | taxID used:135614 OG01412|WP\_010371893.1 | type IV pilus twitching motility pr | taxID used:135614 OG01413|WP\_116894708.1 | M23 family peptidase [Xanthomonas v | taxID used:135614 OG01414|WP\_010367083.1 | MULTISPECIES: two-component system | taxID used:135614 OG01415|WP\_010371956.1 | hypothetical protein [Xanthomonas v | taxID used:135614 OG01416|WP\_010367166.1 | MULTISPECIES: bifunctional histidin | taxID used:135614 OG01417|WP\_017116962.1 | MULTISPECIES: type VI secretion pro | taxID used:135614 OG01418|WP\_010368187.1 | MULTISPECIES: N-acetylglucosamine-6 | taxID used:135614 OG01419|WP\_087910813.1 | type IV secretion system protein Vi | taxID used:135614 OG00141|WP\_086020514.1 | bifunctional DNA primase/helicase [ | taxID used:135614 OG01420|WP\_010370048.1 | MULTISPECIES: LysM peptidoglycan-bi | taxID used:135614 OG01421|WP\_010366461.1 | MULTISPECIES: 5-methyltetrahydrofol | taxID used:135614 OG01422|WP\_010366364.1 | MULTISPECIES: molecular chaperone D | taxID used:135614 OG01423|WP\_010368556.1 | MULTISPECIES: glycosyltransferase f | taxID used:135614 OG01424|WP\_017116523.1 | alpha/beta hydrolase [Xanthomonas v | taxID used:135614 OG01425|WP\_010370264.1 | hypothetical protein [Xanthomonas v | taxID used:135614 OG01426|WP\_053012821.1 | IS3 family transposase, partial [Xa | taxID used:135614 OG01427|WP\_106888039.1 | IS3 family transposase, partial [Xa | taxID used:135614 OG01428|WP\_017170377.1 | MULTISPECIES: site-specific integra | taxID used:135614 OG01429|WP\_026112090.1 | MULTISPECIES: hypothetical protein | taxID used:135614 OG00142|WP\_010371657.1 | MULTISPECIES: pyruvate dehydrogenas | taxID used:135614 OG01430|WP\_017112910.1 | MULTISPECIES: 4-hydroxyphenylpyruva | taxID used:135614 OG01431|WP\_010367112.1 | MULTISPECIES: carbamoyl-phosphate s | taxID used:135614 OG01432|WP\_058958769.1 | MULTISPECIES: M23 family peptidase | taxID used:135614 OG01433|WP\_010373765.1 | MULTISPECIES: flagellar biosynthesi | taxID used:135614 OG01434|WP\_017116901.1 | MULTISPECIES: DNA-protecting protei | taxID used:135614 OG01435|WP\_106888060.1 | type IV secretion system protein Vi | taxID used:135614 OG01436|WP\_017116805.1 | MULTISPECIES: 5-(carboxyamino)imida | taxID used:135614 OG01437|WP\_010364169.1 | pyrroloquinoline quinone biosynthes | taxID used:135614 OG01438|WP\_017117353.1 | MULTISPECIES: glycosyltransferase [ | taxID used:135614 OG01439|WP\_010367699.1 | 3,4-dihydroxy-2-butanone-4-phosphat | taxID used:135614 OG00143|WP\_010368199.1 | MULTISPECIES: TonB-dependent recept | taxID used:135614 OG01440|WP\_039443838.1 | DUF4236 domain-containing protein [ | taxID used:135614 OG01441|WP\_058958771.1 | MULTISPECIES: hypothetical protein | taxID used:135614 OG01442|WP\_010364299.1 | MULTISPECIES: type II toxin-antitox | taxID used:135614 OG01443|WP\_010363622.1 | MULTISPECIES: inorganic phosphate t | taxID used:135614 OG01444|WP\_039438513.1 | Type IV secretion system protein vi | taxID used:135614 OG01445|WP\_026112530.1 | type IV secretion system protein Vi | taxID used:135614 OG01446|WP\_026112750.1 | MULTISPECIES: glycoside hydrolase f | taxID used:135614 OG01447|WP\_010374356.1 | ROK family transcriptional regulato | taxID used:135614 OG01448|WP\_010365874.1 | MULTISPECIES: hybrid sensor histidi | taxID used:135614 OG01449|WP\_010363281.1 | DUF4062 domain-containing protein [ | taxID used:135614 OG00144|WP\_010373420.1 | MULTISPECIES: type VI secretion sys | taxID used:135614 OG01450|WP\_010366193.1 | MULTISPECIES: succinyl-diaminopimel | taxID used:135614 OG01451|WP\_010368253.1 | MULTISPECIES: rod shape-determining | taxID used:135614 OG01452|WP\_026112542.1 | MULTISPECIES: HDOD domain-containin | taxID used:135614 OG01453|WP\_010367431.1 | MULTISPECIES: gfo/Idh/MocA family o | taxID used:135614 OG01454|WP\_010373187.1 | MULTISPECIES: HAMP domain-containin | taxID used:135614 OG01455|WP\_097370619.1 | MULTISPECIES: peptide chain release | taxID used:135614 OG01456|WP\_010372866.1 | MULTISPECIES: glutamate 5-kinase [X | taxID used:135614 OG01457|WP\_017113073.1 | MULTISPECIES: chemotaxis response r | taxID used:135614 OG01458|WP\_026112985.1 | type IV secretion protein VirB10 [X | taxID used:135614 OG01459|WP\_054393955.1 | MULTISPECIES: FtsH protease activit | taxID used:135614 OG00145|WP\_010374514.1 | glucan 1,4-alpha-glucosidase [Xanth | taxID used:135614 OG01460|WP\_017113090.1 | MULTISPECIES: sensor domain-contain | taxID used:135614 OG01461|WP\_010366368.1 | MULTISPECIES: prephenate dehydrogen | taxID used:135614 OG01462|WP\_010371145.1 | mechanosensitive ion channel family | taxID used:135614 OG01463|WP\_010374856.1 | MULTISPECIES: alpha/beta fold hydro | taxID used:135614 OG01464|WP\_010365569.1 | MULTISPECIES: S-(hydroxymethyl)glut | taxID used:135614 OG01465|WP\_010372337.1 | MULTISPECIES: phytase [Xanthomonas] | taxID used:135614 OG01466|WP\_026112773.1 | MULTISPECIES: general secretion pat | taxID used:135614 OG01467|WP\_010372531.1 | MULTISPECIES: polyamine ABC transpo | taxID used:135614 OG01468|WP\_010373650.1 | MULTISPECIES: DegT/DnrJ/EryC1/StrS | taxID used:135614 OG01469|WP\_010369080.1 | MULTISPECIES: NADH:flavin oxidoredu | taxID used:135614 OG00146|WP\_010368657.1 | MULTISPECIES: TonB-dependent recept | taxID used:135614 OG01470|WP\_039436485.1 | replication protein, partial [Xanth | taxID used:135614 OG01471|WP\_017116285.1 | exported exo-alpha-sialidase [Xanth | taxID used:135614 OG01472|WP\_087910794.1 | hypothetical protein [Xanthomonas v | taxID used:135614 OG01473|WP\_010371935.1 | MULTISPECIES: PLP-dependent cystein | taxID used:135614 OG01474|WP\_024420139.1 | MULTISPECIES: SAM-dependent methylt | taxID used:135614 OG01475|WP\_010374699.1 | MULTISPECIES: anhydro-N-acetylmuram | taxID used:135614 OG01476|WP\_017115450.1 | MULTISPECIES: hypothetical protein | taxID used:135614 OG01477|WP\_017170353.1 | MULTISPECIES: hypothetical protein | taxID used:135614 OG01478|WP\_017119485.1 | MULTISPECIES: glycosyl hydrolase [X | taxID used:135614 OG01479|WP\_010363166.1 | IS3 family transposase, partial [Xa | taxID used:135614 OG00147|WP\_054393990.1 | translation initiation factor IF-2, | taxID used:135614 OG01480|WP\_017121766.1 | IS3 family transposase, partial [Xa | taxID used:135614 OG01481|WP\_010367553.1 | metal-dependent hydrolase, partial | taxID used:135614 OG01482|WP\_026113240.1 | MULTISPECIES: DNA cytosine methyltr | taxID used:135614 OG01483|WP\_017121808.1 | MULTISPECIES: cupin domain-containi | taxID used:135614 OG01484|WP\_010370020.1 | GTP cyclohydrolase II RibA, partial | taxID used:135614 OG01485|WP\_017112451.1 | MULTISPECIES: DUF4380 domain-contai | taxID used:135614 OG01486|WP\_010363661.1 | MULTISPECIES: aminotransferase clas | taxID used:135614 OG01487|WP\_010371184.1 | MULTISPECIES: NADH-quinone oxidored | taxID used:135614 OG01488|WP\_054393985.1 | hypothetical protein, partial [Xant | taxID used:135614 OG01489|WP\_010365573.1 | MULTISPECIES: glycosyl transferase | taxID used:135614 OG00148|WP\_010365933.1 | TonB-dependent receptor, partial [X | taxID used:135614 OG01490|WP\_010373173.1 | MULTISPECIES: YeiH family putative | taxID used:135614 OG01491|WP\_125523535.1 | IS3 family transposase [Xanthomonas | taxID used:135614 OG01492|WP\_026112477.1 | MexH family multidrug efflux RND tr | taxID used:135614 OG01493|WP\_080765185.1 | DUF2213 domain-containing protein [ | taxID used:135614 OG01494|WP\_087911202.1 | hypothetical protein, partial [Xant | taxID used:135614 OG01495|WP\_010367803.1 | MULTISPECIES: LPS export ABC transp | taxID used:135614 OG01496|WP\_087911215.1 | MULTISPECIES: type IV secretion sys | taxID used:135614 OG01497|WP\_010368684.1 | MULTISPECIES: glycine cleavage syst | taxID used:135614 OG01498|WP\_010365678.1 | MULTISPECIES: tRNA pseudouridine(13 | taxID used:135614 OG01499|WP\_017113679.1 | MULTISPECIES: hypothetical protein | taxID used:135614 OG00149|WP\_017113152.1 | autotransporter domain-containing p | taxID used:135614 OG00014|WP\_010364927.1 | MULTISPECIES: autotransporter domai | taxID used:135614 OG01500|WP\_010366687.1 | chorismate synthase, partial [Xanth | taxID used:135614 OG01501|WP\_033009783.1 | restriction endonuclease or methyla | taxID used:135614 OG01502|WP\_010370424.1 | MULTISPECIES: cell division protein | taxID used:135614 OG01503|WP\_010368597.1 | MULTISPECIES: sulfate ABC transport | taxID used:135614 OG01504|WP\_010371349.1 | type IV secretion system protein [X | taxID used:135614 OG01505|WP\_010366769.1 | MULTISPECIES: protease [Xanthomonas | taxID used:135614 OG01506|WP\_010374836.1 | MULTISPECIES: catalase family perox | taxID used:135614 OG01508|WP\_010364390.1 | MULTISPECIES: threonine/serine dehy | taxID used:135614 OG01509|WP\_052239604.1 | site-specific integrase [Xanthomona | taxID used:135614 OG00150|WP\_010367889.1 | serine kinase, partial [Xanthomonas | taxID used:135614 OG01510|WP\_010368610.1 | MULTISPECIES: OmpA family protein [ | taxID used:135614 OG01511|WP\_084819000.1 | hypothetical protein [Xanthomonas v | taxID used:135614 OG01512|WP\_010364805.1 | MULTISPECIES: DNA replication/repai | taxID used:135614 OG01513|WP\_010370540.1 | MULTISPECIES: D-alanine--D-alanine | taxID used:135614 OG01514|WP\_010373460.1 | MULTISPECIES: efflux RND transporte | taxID used:135614 OG01515|WP\_010372118.1 | MULTISPECIES: alanine racemase [Xan | taxID used:135614 OG01516|WP\_010375294.1 | MULTISPECIES: aliphatic sulfonate A | taxID used:135614 OG01517|WP\_010374805.1 | MULTISPECIES: DUF3182 domain-contai | taxID used:135614 OG01518|WP\_010364757.1 | MULTISPECIES: PA0069 family radical | taxID used:135614 OG01519|WP\_010367098.1 | MULTISPECIES: DUF1176 domain-contai | taxID used:135614 OG00151|WP\_010367566.1 | MULTISPECIES: phosphoenolpyruvate c | taxID used:135614 OG01520|WP\_010366071.1 | MULTISPECIES: class I SAM-dependent | taxID used:135614 OG01521|WP\_054393907.1 | hypothetical protein, partial [Xant | taxID used:135614 OG01522|WP\_010367998.1 | MULTISPECIES: pyruvate dehydrogenas | taxID used:135614 OG01523|WP\_010363536.1 | MULTISPECIES: exopolysaccharide xan | taxID used:135614 OG01524|WP\_010376411.1 | MULTISPECIES: glycerophosphoryl die | taxID used:135614 OG01525|WP\_010365597.1 | MULTISPECIES: ABC transporter perme | taxID used:135614 OG01526|WP\_010372844.1 | MULTISPECIES: acetylornithine deace | taxID used:135614 OG01527|WP\_010364355.1 | hypothetical protein, partial [Xant | taxID used:135614 OG01528|WP\_054394006.1 | MULTISPECIES: AI-2E family transpor | taxID used:135614 OG01529|WP\_017116186.1 | MULTISPECIES: ribonuclease D [Xanth | taxID used:135614 OG00152|WP\_017115700.1 | MULTISPECIES: histidinol-phosphatas | taxID used:135614 OG01530|WP\_010363021.1 | conjugal transfer protein TraH [Xan | taxID used:135614 OG01531|WP\_010366017.1 | MULTISPECIES: Glu/Leu/Phe/Val dehyd | taxID used:135614 OG01532|WP\_082347513.1 | RES domain-containing protein [Xant | taxID used:135614 OG01533|WP\_010367705.1 | MULTISPECIES: bifunctional diaminoh | taxID used:135614 OG01534|WP\_010373300.1 | MULTISPECIES: sn-glycerol-3-phospha | taxID used:135614 OG01535|WP\_010369115.1 | 3-dehydroquinate synthase, partial | taxID used:135614 OG01536|WP\_010374790.1 | MULTISPECIES: ferredoxin reductase | taxID used:135614 OG01537|WP\_010365516.1 | MULTISPECIES: ribosome small subuni | taxID used:135614 OG01538|WP\_017112473.1 | MULTISPECIES: flagellar basal body | taxID used:135614 OG01539|WP\_010363474.1 | MULTISPECIES: A/G-specific adenine | taxID used:135614 OG00153|WP\_010377530.1 | leucine--tRNA ligase, partial [Xant | taxID used:135614 OG01540|WP\_078563106.1 | MULTISPECIES: type III secretion sy | taxID used:135614 OG01541|WP\_010367350.1 | MULTISPECIES: branched-chain amino | taxID used:135614 OG01542|WP\_010368353.1 | MULTISPECIES: DUF1615 domain-contai | taxID used:135614 OG01543|WP\_010363755.1 | MULTISPECIES: dipeptide epimerase [ | taxID used:135614 OG01544|WP\_010372663.1 | MULTISPECIES: tRNA epoxyqueuosine(3 | taxID used:135614 OG01545|WP\_010369667.1 | ATP synthase subunit alpha [Xanthom | taxID used:135614 OG01546|WP\_010367638.1 | MULTISPECIES: phospho-N-acetylmuram | taxID used:135614 OG01547|WP\_010366489.1 | MULTISPECIES: phosphate ABC transpo | taxID used:135614 OG01548|WP\_010371473.1 | hypothetical protein [Xanthomonas v | taxID used:135614 OG01549|WP\_039442970.1 | LuxR family transcriptional regulat | taxID used:135614 OG00154|WP\_010373519.1 | MULTISPECIES: TonB-dependent recept | taxID used:135614 OG01550|WP\_010373324.1 | MULTISPECIES: NAD-dependent epimera | taxID used:135614 OG01551|WP\_017115279.1 | MULTISPECIES: 3-phosphoserine/phosp | taxID used:135614 OG01552|WP\_039439186.1 | hypothetical protein [Xanthomonas v | taxID used:135614 OG01553|WP\_010366378.1 | MULTISPECIES: HlyD family efflux tr | taxID used:135614 OG01554|WP\_017115586.1 | NAD(P)(+) transhydrogenase (Re/Si-s | taxID used:135614 OG01555|WP\_017118717.1 | replication protein [Xanthomonas va | taxID used:135614 OG01556|WP\_010368221.1 | restriction endonuclease subunit M, | taxID used:135614 OG01557|WP\_010364809.1 | MULTISPECIES: DNA polymerase III su | taxID used:135614 OG01558|WP\_010373238.1 | MULTISPECIES: class III poly(R)-hyd | taxID used:135614 OG01559|WP\_017112646.1 | MULTISPECIES: xanthine dehydrogenas | taxID used:135614 OG00155|WP\_010368450.1 | MULTISPECIES: M1 family peptidase [ | taxID used:135614 OG01560|WP\_010364715.1 | MULTISPECIES: glycoside hydrolase f | taxID used:135614 OG01562|WP\_010363646.1 | MULTISPECIES: glycosyltransferase [ | taxID used:135614 OG01563|WP\_010369554.1 | DUF3616 domain-containing protein, | taxID used:135614 OG01564|WP\_010368283.1 | MULTISPECIES: HlyD family secretion | taxID used:135614 OG01565|WP\_010371754.1 | DUF4880 domain-containing protein, | taxID used:135614 OG01566|WP\_017116394.1 | MULTISPECIES: efflux RND transporte | taxID used:135614 OG01567|WP\_010369121.1 | MULTISPECIES: potassium channel pro | taxID used:135614 OG01568|WP\_010363342.1 | MULTISPECIES: DUF4432 domain-contai | taxID used:135614 OG01569|WP\_010368234.1 | MULTISPECIES: lipid A deacylase Lpx | taxID used:135614 OG00156|WP\_010374409.1 | MULTISPECIES: hypothetical protein | taxID used:135614 OG01570|WP\_010371921.1 | DNA polymerase IV, partial [Xanthom | taxID used:135614 OG01571|WP\_010368189.1 | MULTISPECIES: LacI family DNA-bindi | taxID used:135614 OG01572|WP\_039440129.1 | MULTISPECIES: integron integrase [X | taxID used:135614 OG01573|WP\_010370803.1 | MULTISPECIES: alpha/beta hydrolase | taxID used:135614 OG01574|WP\_010370485.1 | MULTISPECIES: GGDEF domain-containi | taxID used:135614 OG01575|WP\_010367805.1 | MULTISPECIES: LPS export ABC transp | taxID used:135614 OG01576|WP\_010367320.1 | MULTISPECIES: peptide chain release | taxID used:135614 OG01577|WP\_010375808.1 | MULTISPECIES: P-type DNA transfer A | taxID used:135614 OG01578|WP\_010367169.1 | MULTISPECIES: histidinol-phosphate | taxID used:135614 OG01579|WP\_010364711.1 | MULTISPECIES: NAD(P)-dependent alco | taxID used:135614 OG00157|WP\_010366603.1 | MULTISPECIES: DNA gyrase subunit A | taxID used:135614 OG01580|WP\_010371942.1 | MULTISPECIES: copper resistance pro | taxID used:135614 OG01581|WP\_017121566.1 | M4 family peptidase [Xanthomonas va | taxID used:135614 OG01582|WP\_010363534.1 | polysaccharide biosynthesis protein | taxID used:135614 OG01583|WP\_017116184.1 | MULTISPECIES: sensor domain-contain | taxID used:135614 OG01584|WP\_026112077.1 | MULTISPECIES: hypothetical protein | taxID used:135614 OG01585|WP\_010363900.1 | MULTISPECIES: aromatic ring-hydroxy | taxID used:135614 OG01586|WP\_123180688.1 | DUF3363 domain-containing protein, | taxID used:135614 OG01587|WP\_010371818.1 | MULTISPECIES: 3-deoxy-7-phosphohept | taxID used:135614 OG01588|WP\_017112141.1 | MULTISPECIES: DUF2272 domain-contai | taxID used:135614 OG01589|WP\_010367154.1 | MULTISPECIES: hypothetical protein | taxID used:135614 OG00158|WP\_039436272.1 | M1 family peptidase [Xanthomonas va | taxID used:135614 OG01590|WP\_010367296.1 | MULTISPECIES: redox-regulated ATPas | taxID used:135614 OG01591|WP\_010367912.1 | MULTISPECIES: EscU/YscU/HrcU family | taxID used:135614 OG01592|WP\_039445230.1 | hypothetical protein [Xanthomonas v | taxID used:135614 OG01593|WP\_010364723.1 | MULTISPECIES: glycoside hydrolase f | taxID used:135614 OG01594|WP\_010367996.1 | MULTISPECIES: alpha-ketoacid dehydr | taxID used:135614 OG01595|WP\_010368739.1 | MULTISPECIES: homoserine dehydrogen | taxID used:135614 OG01596|WP\_010372088.1 | MULTISPECIES: 16S rRNA methyltransf | taxID used:135614 OG01597|WP\_010370507.1 | MULTISPECIES: hydrolase [Xanthomona | taxID used:135614 OG01598|WP\_010366196.1 | MULTISPECIES: right-handed parallel | taxID used:135614 OG01599|WP\_082320240.1 | MULTISPECIES: chemotaxis protein [X | taxID used:135614 OG00159|WP\_010374571.1 | glycerol-3-phosphate 1-O-acyltransf | taxID used:135614 OG00015|WP\_010364703.1 | glutamate synthase large subunit, p | taxID used:135614 OG01600|WP\_010369826.1 | MULTISPECIES: ribonucleotide-diphos | taxID used:135614 OG01601|WP\_010365494.1 | MULTISPECIES: aldo/keto reductase [ | taxID used:135614 OG01602|WP\_017116284.1 | MULTISPECIES: N(4)-(beta-N-acetylgl | taxID used:135614 OG01603|WP\_005989679.1 | MULTISPECIES: LacI family DNA-bindi | taxID used:135614 OG01604|WP\_010372318.1 | MULTISPECIES: tRNA preQ1(34) S-aden | taxID used:135614 OG01605|WP\_106888051.1 | type IV secretion system protein [X | taxID used:135614 OG01606|WP\_017116122.1 | MULTISPECIES: hypothetical protein | taxID used:135614 OG01607|WP\_010368225.1 | MULTISPECIES: bifunctional nicotina | taxID used:135614 OG01608|WP\_010368214.1 | MULTISPECIES: oxidoreductase [Xanth | taxID used:135614 OG01609|WP\_017116707.1 | nitronate monooxygenase, partial [X | taxID used:135614 OG00160|WP\_010363032.1 | conjugal transfer protein TraG [Xan | taxID used:135614 OG01610|WP\_010363239.1 | dihydrorhizobitoxine desaturase [Xa | taxID used:135614 OG01611|WP\_010368412.1 | endolytic transglycosylase MltG, pa | taxID used:135614 OG01612|WP\_082320307.1 | MULTISPECIES: DNA cytosine methyltr | taxID used:135614 OG01613|WP\_010374564.1 | MULTISPECIES: alpha-N-arabinofurano | taxID used:135614 OG01614|WP\_106888067.1 | IS3 family transposase [Xanthomonas | taxID used:135614 OG01615|WP\_010365270.1 | chemotaxis response regulator prote | taxID used:135614 OG01616|WP\_010364905.1 | MULTISPECIES: VacJ family lipoprote | taxID used:135614 OG01617|WP\_010364384.1 | MULTISPECIES: 3-isopropylmalate deh | taxID used:135614 OG01618|WP\_010369111.1 | MULTISPECIES: uroporphyrinogen deca | taxID used:135614 OG01619|WP\_010366601.1 | MULTISPECIES: S-methyl-5-thioribose | taxID used:135614 OG00161|WP\_010370251.1 | MULTISPECIES: glycoside hydrolase f | taxID used:135614 OG01620|WP\_010363260.1 | hypothetical protein [Xanthomonas v | taxID used:135614 OG01621|WP\_054393996.1 | MULTISPECIES: arabinogalactan endo- | taxID used:135614 OG01622|WP\_010365631.1 | MULTISPECIES: dTDP-glucose 4,6-dehy | taxID used:135614 OG01623|WP\_010370028.1 | MULTISPECIES: CDP-glycerol glycerop | taxID used:135614 OG01624|WP\_010369140.1 | MULTISPECIES: cupin-like domain-con | taxID used:135614 OG01625|WP\_017116727.1 | NAD/FAD-dependent oxidoreductase [X | taxID used:135614 OG01626|WP\_123180788.1 | lytic murein transglycosylase, part | taxID used:135614 OG01627|WP\_080765167.1 | N-acetylmuramoyl-L-alanine amidase | taxID used:135614 OG01628|WP\_010366458.1 | MULTISPECIES: carbohydrate kinase [ | taxID used:135614 OG01629|WP\_010366927.1 | MULTISPECIES: alcohol dehydrogenase | taxID used:135614 OG00162|WP\_010369816.1 | GNAT family N-acetyltransferase, pa | taxID used:135614 OG01630|WP\_017115772.1 | hypothetical protein [Xanthomonas v | taxID used:135614 OG01631|WP\_010363292.1 | MULTISPECIES: lipopolysaccharide he | taxID used:135614 OG01632|WP\_010377901.1 | MULTISPECIES: LacI family DNA-bindi | taxID used:135614 OG01633|WP\_010371434.1 | MULTISPECIES: UDP-N-acetylmuramate | taxID used:135614 OG01634|WP\_017116793.1 | MULTISPECIES: D-glycerate dehydroge | taxID used:135614 OG01635|WP\_010363639.1 | MULTISPECIES: Hpr(Ser) kinase/phosp | taxID used:135614 OG01636|WP\_010363530.1 | MULTISPECIES: GDP-mannose--glycolip | taxID used:135614 OG01637|WP\_010369593.1 | MULTISPECIES: sulfotransferase [Xan | taxID used:135614 OG01638|WP\_017116281.1 | MULTISPECIES: type IV secretion sys | taxID used:135614 OG01639|WP\_010370932.1 | MULTISPECIES: MexH family multidrug | taxID used:135614 OG00163|WP\_010364161.1 | MULTISPECIES: TonB-dependent recept | taxID used:135614 OG01640|WP\_010370968.1 | cytochrome P450 [Xanthomonas vasico | taxID used:135614 OG01641|WP\_017115849.1 | hypothetical protein, partial [Xant | taxID used:135614 OG01642|WP\_010372979.1 | MULTISPECIES: agmatine deiminase fa | taxID used:135614 OG01643|WP\_080764640.1 | hypothetical protein [Xanthomonas v | taxID used:135614 OG01644|WP\_010368057.1 | MULTISPECIES: aminotransferase clas | taxID used:135614 OG01645|WP\_033011908.1 | MULTISPECIES: IS21 family transposa | taxID used:135614 OG01646|WP\_010365815.1 | bifunctional riboflavin kinase/FAD | taxID used:135614 OG01647|WP\_010367428.1 | MULTISPECIES: 23S rRNA (cytidine(24 | taxID used:135614 OG01648|WP\_010374576.1 | MULTISPECIES: LacI family transcrip | taxID used:135614 OG01649|WP\_010369242.1 | MULTISPECIES: phosphoribosylformylg | taxID used:135614 OG00164|WP\_039437109.1 | MULTISPECIES: phage tail tape measu | taxID used:135614 OG01650|WP\_010372838.1 | MULTISPECIES: N-acetylornithine car | taxID used:135614 OG01651|WP\_010368929.1 | MULTISPECIES: biotin synthase BioB | taxID used:135614 OG01652|WP\_010365894.1 | MULTISPECIES: galactose mutarotase | taxID used:135614 OG01653|WP\_010369416.1 | MULTISPECIES: xanthine dehydrogenas | taxID used:135614 OG01654|WP\_054394003.1 | MULTISPECIES: tRNA (adenosine(37)-N | taxID used:135614 OG01655|WP\_116645459.1 | DUF4917 family protein [Xanthomonas | taxID used:135614 OG01656|WP\_010367649.1 | MULTISPECIES: 16S rRNA (cytosine(14 | taxID used:135614 OG01657|WP\_026112784.1 | MULTISPECIES: phenol degradation pr | taxID used:135614 OG01658|WP\_010370971.1 | MULTISPECIES: LacI family DNA-bindi | taxID used:135614 OG01659|WP\_010365498.1 | ABC transporter substrate-binding p | taxID used:135614 OG00165|WP\_039437097.1 | hypothetical protein [Xanthomonas v | taxID used:135614 OG01660|WP\_010368742.1 | MULTISPECIES: alpha/beta hydrolase | taxID used:135614 OG01661|WP\_010367517.1 | MULTISPECIES: ABC transporter subst | taxID used:135614 OG01662|WP\_010368587.1 | MULTISPECIES: L-threonine 3-dehydro | taxID used:135614 OG01663|WP\_017116078.1 | MULTISPECIES: ketoacyl-ACP synthase | taxID used:135614 OG01664|WP\_010374656.1 | MULTISPECIES: bile acid:sodium symp | taxID used:135614 OG01665|WP\_082345047.1 | abortive phage resistance protein [ | taxID used:135614 OG01666|WP\_010365805.1 | MULTISPECIES: GTPase ObgE [Xanthomo | taxID used:135614 OG01667|WP\_010366003.1 | MULTISPECIES: ligase-associated DNA | taxID used:135614 OG01668|WP\_017117477.1 | phenol degradation protein [Xanthom | taxID used:135614 OG01669|WP\_010364541.1 | type IV pilus assembly protein PilM | taxID used:135614 OG00166|WP\_026112758.1 | MULTISPECIES: DUF802 domain-contain | taxID used:135614 OG01670|WP\_010363988.1 | MULTISPECIES: cell envelope integri | taxID used:135614 OG01671|WP\_003489919.1 | MULTISPECIES: type IV pilus twitchi | taxID used:135614 OG01672|WP\_010373330.1 | MULTISPECIES: tetraacyldisaccharide | taxID used:135614 OG01673|WP\_017112277.1 | MULTISPECIES: DUF475 domain-contain | taxID used:135614 OG01674|WP\_010363360.1 | MULTISPECIES: L-glyceraldehyde 3-ph | taxID used:135614 OG01675|WP\_080762850.1 | hypothetical protein [Xanthomonas v | taxID used:135614 OG01676|WP\_010366041.1 | MULTISPECIES: cation transporter [X | taxID used:135614 OG01677|WP\_017115809.1 | MULTISPECIES: two-component sensor | taxID used:135614 OG01678|WP\_010368259.1 | MULTISPECIES: rod shape-determining | taxID used:135614 OG01679|WP\_010374592.1 | DUF4272 domain-containing protein [ | taxID used:135614 OG00167|WP\_010365719.1 | MULTISPECIES: alanine--tRNA ligase | taxID used:135614 OG01680|WP\_010366462.1 | MULTISPECIES: methyltransferase dom | taxID used:135614 OG01681|WP\_010368575.1 | SPOR domain-containing protein, par | taxID used:135614 OG01682|WP\_010366358.1 | MULTISPECIES: heat-inducible transc | taxID used:135614 OG01683|WP\_010368310.1 | MULTISPECIES: histidine kinase [Xan | taxID used:135614 OG01684|WP\_010369054.1 | 5-methyltetrahydropteroyltriglutama | taxID used:135614 OG01685|WP\_010371435.1 | MULTISPECIES: quinone-dependent dih | taxID used:135614 OG01686|WP\_010365228.1 | rhomboid family intramembrane serin | taxID used:135614 OG01687|WP\_010363299.1 | MULTISPECIES: GTP 3',8-cyclase MoaA | taxID used:135614 OG01688|WP\_010368720.1 | MULTISPECIES: glycosyltransferase [ | taxID used:135614 OG01689|WP\_026112774.1 | MULTISPECIES: hypothetical protein | taxID used:135614 OG00168|WP\_033012134.1 | TonB-dependent receptor, partial [X | taxID used:135614 OG01690|WP\_054393971.1 | MULTISPECIES: hypothetical protein | taxID used:135614 OG01691|WP\_087911193.1 | GGDEF domain-containing protein [Xa | taxID used:135614 OG01692|WP\_026112596.1 | MULTISPECIES: type I-C CRISPR-assoc | taxID used:135614 OG01693|WP\_017113416.1 | MULTISPECIES: hypothetical protein | taxID used:135614 OG01694|WP\_082345061.1 | DUF4238 domain-containing protein [ | taxID used:135614 OG01695|WP\_010368064.1 | MULTISPECIES: anthranilate phosphor | taxID used:135614 OG01696|WP\_010368229.1 | MULTISPECIES: lipoyl synthase [Xant | taxID used:135614 OG01697|WP\_010366310.1 | MULTISPECIES: LysR family transcrip | taxID used:135614 OG01698|WP\_010365384.1 | MULTISPECIES: cytochrome d ubiquino | taxID used:135614 OG01699|WP\_026112620.1 | MULTISPECIES: hypothetical protein | taxID used:135614 OG00169|WP\_010365263.1 | MULTISPECIES: HAMP domain-containin | taxID used:135614 OG00016|WP\_054393866.1 | hypothetical protein, partial [Xant | taxID used:135614 OG01700|WP\_010366173.1 | MULTISPECIES: spore coat U domain-c | taxID used:135614 OG01701|WP\_010366942.1 | MULTISPECIES: aldo/keto reductase [ | taxID used:135614 OG01702|WP\_010363979.1 | MULTISPECIES: Holliday junction bra | taxID used:135614 OG01703|WP\_010364162.1 | MULTISPECIES: ROK family protein [X | taxID used:135614 OG01704|WP\_010368612.1 | MULTISPECIES: zinc-binding alcohol | taxID used:135614 OG01705|WP\_017117064.1 | gamma-glutamyltransferase 2 [Xantho | taxID used:135614 OG01706|WP\_039441063.1 | ImmA/IrrE family metallo-endopeptid | taxID used:135614 OG01707|WP\_010370400.1 | MULTISPECIES: NADP-dependent oxidor | taxID used:135614 OG01708|WP\_010374540.1 | MULTISPECIES: gluconolactonase [Xan | taxID used:135614 OG01709|WP\_010369255.1 | MULTISPECIES: aminoglycoside phosph | taxID used:135614 OG00170|WP\_010372638.1 | MULTISPECIES: DNA ligase D [Xanthom | taxID used:135614 OG01710|WP\_026112811.1 | kelch repeat-containing protein [Xa | taxID used:135614 OG01711|WP\_017117506.1 | acyl-CoA desaturase, partial [Xanth | taxID used:135614 OG01712|WP\_017117276.1 | hypothetical protein, partial [Xant | taxID used:135614 OG01713|WP\_033009525.1 | MULTISPECIES: patatin-like phosphol | taxID used:135614 OG01714|WP\_017117656.1 | toll/interleukin-1 receptor domain- | taxID used:135614 OG01715|WP\_010368591.1 | MULTISPECIES: sulfate/molybdate ABC | taxID used:135614 OG01716|WP\_017118524.1 | MULTISPECIES: glycerophosphodiester | taxID used:135614 OG01717|WP\_010369849.1 | MULTISPECIES: hypothetical protein | taxID used:135614 OG01718|WP\_010365715.1 | MULTISPECIES: recombinase RecA [Xan | taxID used:135614 OG01719|WP\_010365505.1 | MULTISPECIES: LacI family transcrip | taxID used:135614 OG00171|WP\_010367670.1 | MULTISPECIES: sensor histidine kina | taxID used:135614 OG01720|WP\_010366680.1 | MULTISPECIES: aspartate-semialdehyd | taxID used:135614 OG01721|WP\_010364017.1 | phage portal protein, partial [Xant | taxID used:135614 OG01722|WP\_010364560.1 | MULTISPECIES: VWA domain-containing | taxID used:135614 OG01723|WP\_017112363.1 | KamA family radical SAM protein, pa | taxID used:135614 OG01724|WP\_010369201.1 | MULTISPECIES: cytochrome d ubiquino | taxID used:135614 OG01725|WP\_010368675.1 | MULTISPECIES: nucleoside triphospha | taxID used:135614 OG01726|WP\_039449000.1 | hypothetical protein [Xanthomonas v | taxID used:135614 OG01727|WP\_010367040.1 | MULTISPECIES: sugar kinase [Xanthom | taxID used:135614 OG01728|WP\_010364021.1 | phage major capsid protein, P2 fami | taxID used:135614 OG01729|WP\_010373396.1 | MULTISPECIES: type VI secretion sys | taxID used:135614 OG00172|WP\_010370482.1 | MULTISPECIES: putative peptide modi | taxID used:135614 OG01730|WP\_010363515.1 | MULTISPECIES: 3-oxoacyl-ACP synthas | taxID used:135614 OG01731|WP\_010364623.1 | MULTISPECIES: class 1 fructose-bisp | taxID used:135614 OG01732|WP\_010366490.1 | MULTISPECIES: phosphate ABC transpo | taxID used:135614 OG01733|WP\_010369891.1 | MULTISPECIES: cupin-like domain-con | taxID used:135614 OG01734|WP\_010374916.1 | MULTISPECIES: tRNA dihydrouridine(2 | taxID used:135614 OG01735|WP\_010373744.1 | MULTISPECIES: flagellar motor switc | taxID used:135614 OG01736|WP\_054393873.1 | MULTISPECIES: PAS domain S-box prot | taxID used:135614 OG01737|WP\_017115167.1 | MULTISPECIES: LysR family transcrip | taxID used:135614 OG01738|WP\_087910975.1 | hypothetical protein [Xanthomonas v | taxID used:135614 OG01739|WP\_017114610.1 | MULTISPECIES: TraB/GumN family prot | taxID used:135614 OG00173|WP\_039431473.1 | MULTISPECIES: DUF3363 domain-contai | taxID used:135614 OG01740|WP\_010366823.1 | MULTISPECIES: DNA polymerase III su | taxID used:135614 OG01741|WP\_017170372.1 | MULTISPECIES: ParM/StbA family prot | taxID used:135614 OG01742|WP\_010365475.1 | hypothetical protein [Xanthomonas v | taxID used:135614 OG01743|WP\_123184409.1 | type II/IV secretion system family | taxID used:135614 OG01744|WP\_010372556.1 | MULTISPECIES: magnesium and cobalt | taxID used:135614 OG01745|WP\_010368736.1 | MULTISPECIES: homoserine O-succinyl | taxID used:135614 OG01746|WP\_039442447.1 | hypothetical protein [Xanthomonas v | taxID used:135614 OG01747|WP\_017112367.1 | MULTISPECIES: RNA polymerase-bindin | taxID used:135614 OG01748|WP\_010372942.1 | LacI family transcriptional regulat | taxID used:135614 OG01749|WP\_010370562.1 | MULTISPECIES: 3-oxoacyl-ACP synthas | taxID used:135614 OG00174|WP\_017119930.1 | sensor domain-containing diguanylat | taxID used:135614 OG01750|WP\_010368331.1 | MULTISPECIES: hypothetical protein | taxID used:135614 OG01751|WP\_010370589.1 | MULTISPECIES: NAD(P)-dependent glyc | taxID used:135614 OG01752|WP\_017116050.1 | MULTISPECIES: type VI secretion sys | taxID used:135614 OG01753|WP\_010368188.1 | MULTISPECIES: SIS domain-containing | taxID used:135614 OG01754|WP\_010364554.1 | MULTISPECIES: MoxR family ATPase [X | taxID used:135614 OG01755|WP\_010369742.1 | MULTISPECIES: xanthomonadin biosynt | taxID used:135614 OG01756|WP\_010370797.1 | DctP family TRAP transporter solute | taxID used:135614 OG01757|WP\_082347461.1 | N-acetylmuramoyl-L-alanine amidase | taxID used:135614 OG01758|WP\_010370550.1 | MULTISPECIES: NAD-dependent epimera | taxID used:135614 OG01759|WP\_010370738.1 | MULTISPECIES: TIGR00266 family prot | taxID used:135614 OG00175|WP\_010365486.1 | MULTISPECIES: hybrid sensor histidi | taxID used:135614 OG01760|WP\_010370517.1 | MULTISPECIES: isoaspartyl peptidase | taxID used:135614 OG01761|WP\_010363633.1 | MULTISPECIES: LLM class flavin-depe | taxID used:135614 OG01762|WP\_010370873.1 | MULTISPECIES: fructose-bisphosphate | taxID used:135614 OG01763|WP\_017113182.1 | hypothetical protein, partial [Xant | taxID used:135614 OG01764|WP\_017119821.1 | hypothetical protein [Xanthomonas v | taxID used:135614 OG01765|WP\_010367549.1 | MULTISPECIES: tRNA dihydrouridine s | taxID used:135614 OG01766|WP\_010372078.1 | MULTISPECIES: alpha/beta fold hydro | taxID used:135614 OG01767|WP\_010365859.1 | MULTISPECIES: ATP-binding protein [ | taxID used:135614 OG01768|WP\_026112196.1 | hypothetical protein [Xanthomonas v | taxID used:135614 OG01769|WP\_010365180.1 | MULTISPECIES: membrane protein [Xan | taxID used:135614 OG00176|WP\_017116096.1 | 1,4-beta-D-glucan glucohydrolase, p | taxID used:135614 OG01770|WP\_010369142.1 | MULTISPECIES: MBL fold metallo-hydr | taxID used:135614 OG01771|WP\_026112879.1 | MULTISPECIES: HAMP domain-containin | taxID used:135614 OG01772|WP\_010369055.1 | MULTISPECIES: DUF1852 domain-contai | taxID used:135614 OG01773|WP\_010366151.1 | UDP-3-O-(3-hydroxymyristoyl)glucosa | taxID used:135614 OG01774|WP\_010368664.1 | MULTISPECIES: glucokinase [Xanthomo | taxID used:135614 OG01775|WP\_010374556.1 | MULTISPECIES: endo-1,4-beta-xylanas | taxID used:135614 OG01776|WP\_010379005.1 | MULTISPECIES: inosine-uridine prefe | taxID used:135614 OG01777|WP\_010365988.1 | MULTISPECIES: beta-N-acetylhexosami | taxID used:135614 OG01778|WP\_010372891.1 | tetratricopeptide repeat protein, p | taxID used:135614 OG01779|WP\_010364395.1 | MULTISPECIES: ketol-acid reductoiso | taxID used:135614 OG01780|WP\_010363591.1 | MULTISPECIES: phenylalanine--tRNA l | taxID used:135614 OG01781|WP\_003490161.1 | MULTISPECIES: type I glyceraldehyde | taxID used:135614 OG01782|WP\_010368360.1 | MULTISPECIES: glutathione S-transfe | taxID used:135614 OG01783|WP\_010369920.1 | MULTISPECIES: porphobilinogen synth | taxID used:135614 OG01785|WP\_017117813.1 | MULTISPECIES: NAD-dependent epimera | taxID used:135614 OG01786|WP\_010369399.1 | MULTISPECIES: NAD(P)-dependent alco | taxID used:135614 OG01787|WP\_010373500.1 | MULTISPECIES: exodeoxyribonuclease | taxID used:135614 OG01788|WP\_010370669.1 | MULTISPECIES: alpha/beta fold hydro | taxID used:135614 OG01789|WP\_010364887.1 | zinc-binding alcohol dehydrogenase | taxID used:135614 OG00178|WP\_010367964.1 | MULTISPECIES: malto-oligosyltrehalo | taxID used:135614 OG01790|WP\_017121641.1 | P-type conjugative transfer protein | taxID used:135614 OG01791|WP\_017116495.1 | hypothetical protein, partial [Xant | taxID used:135614 OG01792|WP\_010372060.1 | MULTISPECIES: methionine ABC transp | taxID used:135614 OG01793|WP\_113622535.1 | IS5 family transposase, partial [Xa | taxID used:135614 OG01794|WP\_106888043.1 | IS5/IS1182 family transposase, part | taxID used:135614 OG01795|WP\_010373388.1 | MULTISPECIES: cytochrome-c peroxida | taxID used:135614 OG01796|WP\_113622305.1 | MULTISPECIES: IS5 family transposas | taxID used:135614 OG01797|WP\_010368534.1 | MULTISPECIES: NAD-dependent isocitr | taxID used:135614 OG01798|WP\_010365435.1 | MULTISPECIES: aldo/keto reductase [ | taxID used:135614 OG01799|WP\_126922540.1 | IS5 family transposase [Xanthomonas | taxID used:135614 OG00179|WP\_097370594.1 | DUF853 domain-containing protein [X | taxID used:135614 OG00017|WP\_017116463.1 | MULTISPECIES: carbohydrate-binding | taxID used:135614 OG01800|WP\_054393964.1 | MULTISPECIES: ABC transporter ATP-b | taxID used:135614 OG01801|WP\_017170416.1 | MULTISPECIES: hypothetical protein | taxID used:135614 OG01803|WP\_106888037.1 | IS5/IS1182 family transposase, part | taxID used:135614 OG01804|WP\_106888034.1 | IS5 family transposase, partial [Xa | taxID used:135614 OG01805|WP\_106888025.1 | IS5 family transposase, partial [Xa | taxID used:135614 OG01806|WP\_087910750.1 | IS5 family transposase, partial [Xa | taxID used:135614 OG01807|WP\_057417709.1 | MULTISPECIES: LysR family transcrip | taxID used:135614 OG01809|WP\_087910750.1 | IS5 family transposase, partial [Xa | taxID used:135614 OG00180|WP\_010369376.1 | MULTISPECIES: bifunctional aspartat | taxID used:135614 OG01810|WP\_010369865.1 | MULTISPECIES: cation transporter [X | taxID used:135614 OG01811|WP\_010366544.1 | MULTISPECIES: aldo/keto reductase [ | taxID used:135614 OG01812|WP\_010366081.1 | DUF1365 domain-containing protein, | taxID used:135614 OG01813|WP\_026112793.1 | aldo/keto reductase, partial [Xanth | taxID used:135614 OG01814|WP\_010367373.1 | MULTISPECIES: exodeoxyribonuclease | taxID used:135614 OG01815|WP\_033012133.1 | MULTISPECIES: glyoxalase/bleomycin | taxID used:135614 OG01816|WP\_010372468.1 | MULTISPECIES: two-component system | taxID used:135614 OG01817|WP\_010363902.1 | MULTISPECIES: oxidoreductase [Xanth | taxID used:135614 OG01818|WP\_010381603.1 | MULTISPECIES: LysR family transcrip | taxID used:135614 OG01819|WP\_010366210.1 | MULTISPECIES: 23S rRNA pseudouridin | taxID used:135614 OG00181|WP\_010366185.1 | MULTISPECIES: bifunctional uridylyl | taxID used:135614 OG01820|WP\_010378578.1 | MULTISPECIES: glycoside hydrolase f | taxID used:135614 OG01821|WP\_010371899.1 | fumarylacetoacetate hydrolase, part | taxID used:135614 OG01822|WP\_010366840.1 | MULTISPECIES: peptidylprolyl isomer | taxID used:135614 OG01823|WP\_010372270.1 | MULTISPECIES: LacI family DNA-bindi | taxID used:135614 OG01824|WP\_017116082.1 | flagellar M-ring protein FliF, part | taxID used:135614 OG01825|WP\_017116570.1 | murein L,D-transpeptidase, partial | taxID used:135614 OG01826|WP\_017118871.1 | MULTISPECIES: IS1595 family transpo | taxID used:135614 OG01827|WP\_002811635.1 | MULTISPECIES: DNA-directed RNA poly | taxID used:135614 OG01828|WP\_010366429.1 | MULTISPECIES: hypothetical protein | taxID used:135614 OG01829|WP\_010369386.1 | MULTISPECIES: octaprenyl-diphosphat | taxID used:135614 OG00182|WP\_010365931.1 | MULTISPECIES: glycoside hydrolase f | taxID used:135614 OG01830|WP\_010364075.1 | phage late control D family protein | taxID used:135614 OG01831|WP\_010367564.1 | hypothetical protein, partial [Xant | taxID used:135614 OG01832|WP\_010369755.1 | MULTISPECIES: acyltransferase [Xant | taxID used:135614 OG01833|WP\_010370709.1 | L-histidine N(alpha)-methyltransfer | taxID used:135614 OG01834|WP\_010366417.1 | CAP domain-containing protein, part | taxID used:135614 OG01835|WP\_017113361.1 | MULTISPECIES: YscQ/HrcQ family type | taxID used:135614 OG01836|WP\_026113028.1 | MULTISPECIES: lytic transglycosylas | taxID used:135614 OG01837|WP\_042505923.1 | DUF692 domain-containing protein [X | taxID used:135614 OG01838|WP\_010364737.1 | MULTISPECIES: D-2-hydroxyacid dehyd | taxID used:135614 OG01839|WP\_054394004.1 | MULTISPECIES: cytochrome c oxidase | taxID used:135614 OG00183|WP\_010368424.1 | MULTISPECIES: Fe/S-dependent 2-meth | taxID used:135614 OG01840|WP\_010363199.1 | MULTISPECIES: helix-turn-helix doma | taxID used:135614 OG01841|WP\_017118062.1 | L-histidine N(alpha)-methyltransfer | taxID used:135614 OG01842|WP\_010369225.1 | KpsF/GutQ family sugar-phosphate is | taxID used:135614 OG01843|WP\_010372686.1 | MULTISPECIES: TraB/GumN family prot | taxID used:135614 OG01844|WP\_017170391.1 | MULTISPECIES: hypothetical protein | taxID used:135614 OG01845|WP\_010372572.1 | MULTISPECIES: PhoH family protein [ | taxID used:135614 OG01846|WP\_010364573.1 | MULTISPECIES: LysR family transcrip | taxID used:135614 OG01847|WP\_010365731.1 | MULTISPECIES: membrane protein [Xan | taxID used:135614 OG01848|WP\_126720827.1 | MULTISPECIES: hypothetical protein | taxID used:135614 OG01849|WP\_010365191.1 | MULTISPECIES: protein-methionine-su | taxID used:135614 OG00184|WP\_010365276.1 | MULTISPECIES: bifunctional aconitat | taxID used:135614 OG01850|WP\_017122816.1 | MULTISPECIES: malate dehydrogenase | taxID used:135614 OG01851|WP\_017112581.1 | MULTISPECIES: FAD:protein FMN trans | taxID used:135614 OG01852|WP\_010372761.1 | IS110 family transposase, partial [ | taxID used:135614 OG01853|WP\_010373023.1 | MULTISPECIES: glycosyltransferase f | taxID used:135614 OG01854|WP\_010374727.1 | MULTISPECIES: NAD-dependent epimera | taxID used:135614 OG01855|WP\_010371836.1 | MULTISPECIES: GDP-mannose 4,6-dehyd | taxID used:135614 OG01856|WP\_010373215.1 | MULTISPECIES: mechanosensitive ion | taxID used:135614 OG01857|WP\_010365699.1 | MULTISPECIES: tRNA (adenosine(37)-N | taxID used:135614 OG01858|WP\_017115996.1 | MULTISPECIES: ferrochelatase [Xanth | taxID used:135614 OG01859|WP\_010367690.1 | MULTISPECIES: thiamine-phosphate ki | taxID used:135614 OG00185|WP\_017116712.1 | DNA mismatch repair protein MutS, p | taxID used:135614 OG01860|WP\_010381478.1 | MULTISPECIES: 5'-nucleotidase [Xant | taxID used:135614 OG01861|WP\_010363163.1 | MULTISPECIES: KR domain-containing | taxID used:135614 OG01862|WP\_017114424.1 | MULTISPECIES: FtsX-like permease fa | taxID used:135614 OG01863|WP\_010370892.1 | MULTISPECIES: LysR family transcrip | taxID used:135614 OG01864|WP\_010373361.1 | 23S rRNA pseudouridine(955/2504/258 | taxID used:135614 OG01865|WP\_026112883.1 | MULTISPECIES: LysR family transcrip | taxID used:135614 OG01866|WP\_010363666.1 | MULTISPECIES: hypothetical protein | taxID used:135614 OG01867|WP\_010373490.1 | MULTISPECIES: siroheme synthase [Xa | taxID used:135614 OG01868|WP\_010367537.1 | MULTISPECIES: aldo/keto reductase [ | taxID used:135614 OG01869|WP\_010367738.1 | MULTISPECIES: hypothetical protein | taxID used:135614 OG01870|WP\_010368826.1 | cell filamentation protein Fic [Xan | taxID used:135614 OG01871|WP\_003482910.1 | MULTISPECIES: thioredoxin-disulfide | taxID used:135614 OG01872|WP\_017115905.1 | lysylphosphatidylglycerol synthetas | taxID used:135614 OG01873|WP\_017112148.1 | uroporphyrin-III methyltransferase | taxID used:135614 OG01874|WP\_010365004.1 | MULTISPECIES: flagellar motor prote | taxID used:135614 OG01875|WP\_010367471.1 | MULTISPECIES: symmetrical bis(5'-nu | taxID used:135614 OG01876|WP\_026112517.1 | MULTISPECIES: FKBP-type peptidyl-pr | taxID used:135614 OG01877|WP\_082320305.1 | MULTISPECIES: restriction endonucle | taxID used:135614 OG01878|WP\_026112080.1 | MULTISPECIES: alcohol dehydrogenase | taxID used:135614 OG01879|WP\_010368305.1 | MULTISPECIES: YafY family transcrip | taxID used:135614 OG00187|WP\_039446694.1 | hypothetical protein [Xanthomonas v | taxID used:135614 OG01880|WP\_017116725.1 | MULTISPECIES: cation transporter [X | taxID used:135614 OG01881|WP\_010368402.1 | MULTISPECIES: ketoacyl-ACP synthase | taxID used:135614 OG01882|WP\_010373680.1 | MULTISPECIES: flagellar motor switc | taxID used:135614 OG01883|WP\_010366113.1 | MULTISPECIES: homocysteine S-methyl | taxID used:135614 OG01884|WP\_010375834.1 | MULTISPECIES: type IV pilus assembl | taxID used:135614 OG01885|WP\_017116113.1 | MULTISPECIES: hypothetical protein | taxID used:135614 OG01886|WP\_017116614.1 | MULTISPECIES: phosphoenolpyruvate m | taxID used:135614 OG01887|WP\_080762925.1 | MULTISPECIES: homoserine O-succinyl | taxID used:135614 OG01888|WP\_010367603.1 | MULTISPECIES: NAD(P)H quinone oxido | taxID used:135614 OG01889|WP\_010368266.1 | MULTISPECIES: hypothetical protein | taxID used:135614 OG00188|WP\_010366466.1 | MULTISPECIES: ribonuclease R [Xanth | taxID used:135614 OG01890|WP\_010367378.1 | MULTISPECIES: prolyl aminopeptidase | taxID used:135614 OG01891|WP\_010366487.1 | MULTISPECIES: phosphate ABC transpo | taxID used:135614 OG01892|WP\_017170379.1 | MULTISPECIES: hypothetical protein | taxID used:135614 OG01893|WP\_010363510.1 | MULTISPECIES: NAD(P)-dependent oxid | taxID used:135614 OG01894|WP\_010367796.1 | MULTISPECIES: site-specific tyrosin | taxID used:135614 OG01895|WP\_010366325.1 | MULTISPECIES: GGDEF domain-containi | taxID used:135614 OG01896|WP\_054393860.1 | MULTISPECIES: alpha/beta hydrolase | taxID used:135614 OG01897|WP\_010364432.1 | MULTISPECIES: DUF3829 domain-contai | taxID used:135614 OG01898|WP\_010368960.1 | MULTISPECIES: alpha/beta hydrolase | taxID used:135614 OG01899|WP\_010365771.1 | MULTISPECIES: LysR family transcrip | taxID used:135614 OG00189|WP\_017116259.1 | TonB-dependent receptor, partial [X | taxID used:135614 OG00018|WP\_017116183.1 | MULTISPECIES: DEAD/DEAH box helicas | taxID used:135614 OG01900|WP\_010375972.1 | MULTISPECIES: SPFH/Band 7/PHB domai | taxID used:135614 OG01901|WP\_010364857.1 | TerC/Alx family metal homeostasis m | taxID used:135614 OG01902|WP\_010372305.1 | MULTISPECIES: protein translocase s | taxID used:135614 OG01903|WP\_010367467.1 | 4-hydroxythreonine-4-phosphate dehy | taxID used:135614 OG01904|WP\_053012894.1 | IS110 family transposase, partial [ | taxID used:135614 OG01905|WP\_010363657.1 | MULTISPECIES: glycosyl transferase | taxID used:135614 OG01906|WP\_010369186.1 | MULTISPECIES: alpha/beta fold hydro | taxID used:135614 OG01907|WP\_010365825.1 | MULTISPECIES: ubiquinol oxidase sub | taxID used:135614 OG01908|WP\_010370397.1 | MULTISPECIES: aldo/keto reductase [ | taxID used:135614 OG01909|WP\_010365646.1 | anchor NAD-dependent epimerase/dehy | taxID used:135614 OG00190|WP\_010363370.1 | MULTISPECIES: ATP-dependent chapero | taxID used:135614 OG01910|WP\_039436023.1 | hypothetical protein [Xanthomonas v | taxID used:135614 OG01911|WP\_033009780.1 | MULTISPECIES: 5'-nucleotidase, lipo | taxID used:135614 OG01912|WP\_010366595.1 | MULTISPECIES: EF-P lysine aminoacyl | taxID used:135614 OG01913|WP\_010367059.1 | MULTISPECIES: Ku protein [Xanthomon | taxID used:135614 OG01914|WP\_026113455.1 | MULTISPECIES: replication initiatio | taxID used:135614 OG01916|WP\_010366512.1 | MULTISPECIES: DUF1684 domain-contai | taxID used:135614 OG01917|WP\_010367399.1 | MULTISPECIES: transaldolase [Xantho | taxID used:135614 OG01918|WP\_010372851.1 | MULTISPECIES: N-acetyl-gamma-glutam | taxID used:135614 OG01919|WP\_039439198.1 | DUF2184 domain-containing protein [ | taxID used:135614 OG00191|WP\_026113014.1 | DUF3857 domain-containing protein, | taxID used:135614 OG01920|WP\_010366538.1 | MULTISPECIES: 5'-nucleotidase [Xant | taxID used:135614 OG01921|WP\_003482735.1 | MULTISPECIES: carbohydrate kinase f | taxID used:135614 OG01922|WP\_017115626.1 | MULTISPECIES: sulfotransferase doma | taxID used:135614 OG01923|WP\_010366140.1 | MULTISPECIES: acetyl-CoA carboxylas | taxID used:135614 OG01924|WP\_010374865.1 | MULTISPECIES: MBL fold metallo-hydr | taxID used:135614 OG01925|WP\_010366654.1 | MULTISPECIES: isopenicillin N synth | taxID used:135614 OG01926|WP\_010367613.1 | MULTISPECIES: 8-oxo-dGTP diphosphat | taxID used:135614 OG01927|WP\_010365147.1 | MULTISPECIES: DUF58 domain-containi | taxID used:135614 OG01928|WP\_010367308.1 | MULTISPECIES: ribose-phosphate pyro | taxID used:135614 OG01929|WP\_010368593.1 | MULTISPECIES: sulfate ABC transport | taxID used:135614 OG00192|WP\_010374941.1 | iron-uptake factor, partial [Xantho | taxID used:135614 OG01930|WP\_054393852.1 | P-type DNA transfer ATPase VirB11, | taxID used:135614 OG01931|WP\_010368413.1 | MULTISPECIES: DNA polymerase III su | taxID used:135614 OG01932|WP\_010370244.1 | MULTISPECIES: hypothetical protein | taxID used:135614 OG01933|WP\_026112662.1 | MULTISPECIES: DUF58 domain-containi | taxID used:135614 OG01934|WP\_010372425.1 | MULTISPECIES: LysR family transcrip | taxID used:135614 OG01935|WP\_017118495.1 | MULTISPECIES: hypothetical protein | taxID used:135614 OG01936|WP\_010370887.1 | MULTISPECIES: cysteine synthase A [ | taxID used:135614 OG01937|WP\_017116178.1 | MULTISPECIES: WYL domain-containing | taxID used:135614 OG01938|WP\_010365823.1 | 4-hydroxy-3-methylbut-2-enyl diphos | taxID used:135614 OG01939|WP\_010370492.1 | MULTISPECIES: LysR family transcrip | taxID used:135614 OG00193|WP\_010366063.1 | MULTISPECIES: bifunctional lysylpho | taxID used:135614 OG01940|WP\_010364362.1 | MULTISPECIES: LpxL/LpxP family Kdo( | taxID used:135614 OG01941|WP\_010369216.1 | MULTISPECIES: HPr kinase/phosphoryl | taxID used:135614 OG01942|WP\_010374652.1 | MULTISPECIES: YihY/virulence factor | taxID used:135614 OG01943|WP\_010367629.1 | MULTISPECIES: D-alanine--D-alanine | taxID used:135614 OG01944|WP\_017116766.1 | hypothetical protein [Xanthomonas v | taxID used:135614 OG01945|WP\_010368354.1 | MULTISPECIES: hypothetical protein | taxID used:135614 OG01946|WP\_010372683.1 | MULTISPECIES: DUF1684 domain-contai | taxID used:135614 OG01947|WP\_010364206.1 | MULTISPECIES: glutathione synthetas | taxID used:135614 OG01948|WP\_010368951.1 | MULTISPECIES: pyridoxal-phosphate d | taxID used:135614 OG01949|WP\_010372277.1 | MULTISPECIES: 1-phosphofructokinase | taxID used:135614 OG00194|WP\_082337920.1 | MULTISPECIES: S9 family peptidase [ | taxID used:135614 OG01950|WP\_039443526.1 | MULTISPECIES: nucleoside-diphosphat | taxID used:135614 OG01951|WP\_017115932.1 | MULTISPECIES: glycerophosphodiester | taxID used:135614 OG01952|WP\_010368216.1 | MULTISPECIES: 6-phosphogluconate de | taxID used:135614 OG01953|WP\_010363456.1 | MULTISPECIES: hydroxyproline-2-epim | taxID used:135614 OG01954|WP\_010378548.1 | MULTISPECIES: homoserine kinase [Xa | taxID used:135614 OG01955|WP\_087910792.1 | hypothetical protein [Xanthomonas v | taxID used:135614 OG01956|WP\_017122563.1 | MULTISPECIES: phosphoribosylaminoim | taxID used:135614 OG01957|WP\_010367593.1 | MULTISPECIES: gfo/Idh/MocA family o | taxID used:135614 OG01958|WP\_052239558.1 | MULTISPECIES: hypothetical protein | taxID used:135614 OG01959|WP\_010369410.1 | MULTISPECIES: xanthine dehydrogenas | taxID used:135614 OG00195|WP\_026112277.1 | MULTISPECIES: glycoside hydrolase f | taxID used:135614 OG01960|WP\_017117312.1 | MULTISPECIES: arginyltransferase [X | taxID used:135614 OG01961|WP\_010368134.1 | MULTISPECIES: hypothetical protein | taxID used:135614 OG01962|WP\_010371804.1 | SMP-30/gluconolactonase/LRE family | taxID used:135614 OG01963|WP\_026112340.1 | MULTISPECIES: M23 family metallopep | taxID used:135614 OG01964|WP\_010364529.1 | MULTISPECIES: inosine-uridine prefe | taxID used:135614 OG01965|WP\_010367233.1 | MULTISPECIES: ABC transporter ATP-b | taxID used:135614 OG01966|WP\_010367395.1 | MULTISPECIES: LysR family transcrip | taxID used:135614 OG01968|WP\_017112290.1 | MULTISPECIES: bifunctional biotin-- | taxID used:135614 OG01969|WP\_010368404.1 | MULTISPECIES: [acyl-carrier-protein | taxID used:135614 OG00196|WP\_017118055.1 | MULTISPECIES: type III secretion sy | taxID used:135614 OG01970|WP\_010369356.1 | MULTISPECIES: aspartate carbamoyltr | taxID used:135614 OG01971|WP\_017116049.1 | MULTISPECIES: hypothetical protein | taxID used:135614 OG01972|WP\_010369665.1 | MULTISPECIES: hypothetical protein | taxID used:135614 OG01973|WP\_017116156.1 | MULTISPECIES: GNAT family N-acetylt | taxID used:135614 OG01974|WP\_017112362.1 | MULTISPECIES: phosphate/phosphite/p | taxID used:135614 OG01975|WP\_010373602.1 | MULTISPECIES: chemotaxis protein Ch | taxID used:135614 OG01976|WP\_010373479.1 | DUF2817 domain-containing protein, | taxID used:135614 OG01977|WP\_087911191.1 | nucleotidyl transferase AbiEii/AbiG | taxID used:135614 OG01978|WP\_010365148.1 | MULTISPECIES: MoxR family ATPase [X | taxID used:135614 OG01979|WP\_010367514.1 | MULTISPECIES: TauD/TfdA family diox | taxID used:135614 OG00197|WP\_010364539.1 | MULTISPECIES: penicillin-binding pr | taxID used:135614 OG01980|WP\_010370558.1 | MULTISPECIES: alpha/beta fold hydro | taxID used:135614 OG01981|WP\_010369852.1 | MULTISPECIES: 2-dehydropantoate 2-r | taxID used:135614 OG01982|WP\_010379236.1 | MULTISPECIES: arginase [Xanthomonas | taxID used:135614 OG01983|WP\_017117502.1 | MULTISPECIES: hypothetical protein | taxID used:135614 OG01984|WP\_010365430.1 | MULTISPECIES: DUF2236 domain-contai | taxID used:135614 OG01985|WP\_010368132.1 | MULTISPECIES: 50S ribosomal protein | taxID used:135614 OG01986|WP\_126922594.1 | IS5/IS1182 family transposase [Xant | taxID used:135614 OG01987|WP\_033009644.1 | SPOR domain-containing protein, par | taxID used:135614 OG01988|WP\_010374452.1 | MULTISPECIES: glycine--tRNA ligase | taxID used:135614 OG01989|WP\_026112911.1 | MULTISPECIES: 2-keto-3-deoxyglucona | taxID used:135614 OG00198|WP\_010369823.1 | MULTISPECIES: ribonucleoside-diphos | taxID used:135614 OG01990|WP\_010367542.1 | MULTISPECIES: ribokinase [Xanthomon | taxID used:135614 OG01991|WP\_054393921.1 | MULTISPECIES: SMP-30/gluconolactona | taxID used:135614 OG01992|WP\_010370132.1 | MULTISPECIES: ABC transporter perme | taxID used:135614 OG01993|WP\_010374500.1 | MULTISPECIES: tRNA 2-thiocytidine(3 | taxID used:135614 OG01994|WP\_097370603.1 | hypothetical protein, partial [Xant | taxID used:135614 OG01995|WP\_010368942.1 | MULTISPECIES: lipid A hydroxylase L | taxID used:135614 OG01996|WP\_039431401.1 | DUF3577 domain-containing protein [ | taxID used:135614 OG01997|WP\_010366896.1 | MULTISPECIES: EamA/RhaT family tran | taxID used:135614 OG01998|WP\_010367902.1 | MULTISPECIES: hypothetical protein | taxID used:135614 OG01999|WP\_054393917.1 | MULTISPECIES: hypothetical protein | taxID used:135614 OG00199|WP\_017117128.1 | ATP-binding cassette domain-contain | taxID used:135614 OG00019|WP\_010367275.1 | DNA-directed RNA polymerase subunit | taxID used:135614 OG00001|WP\_116652141.1 | filamentous hemagglutinin N-termina | taxID used:135614 OG02000|WP\_010364283.1 | MULTISPECIES: efflux RND transporte | taxID used:135614 OG02001|WP\_010367425.1 | MULTISPECIES: EamA/RhaT family tran | taxID used:135614 OG02002|WP\_010368707.1 | MULTISPECIES: alpha/beta hydrolase | taxID used:135614 OG02003|WP\_017115980.1 | MULTISPECIES: LysR family transcrip | taxID used:135614 OG02004|WP\_017112922.1 | MULTISPECIES: GAF domain-containing | taxID used:135614 OG02005|WP\_010369684.1 | MULTISPECIES: oxygen-dependent copr | taxID used:135614 OG02006|WP\_010365637.1 | electron transfer flavoprotein subu | taxID used:135614 OG02007|WP\_017115886.1 | MULTISPECIES: monothiol glutaredoxi | taxID used:135614 OG02008|WP\_010366690.1 | MULTISPECIES: 50S ribosomal protein | taxID used:135614 OG02009|WP\_010370926.1 | MULTISPECIES: sulfate adenylyltrans | taxID used:135614 OG00200|WP\_010364663.1 | DUF1998 domain-containing protein [ | taxID used:135614 OG02010|WP\_010366667.1 | MULTISPECIES: LysR family transcrip | taxID used:135614 OG02011|WP\_010364885.1 | MULTISPECIES: LysR family transcrip | taxID used:135614 OG02012|WP\_010368289.1 | MULTISPECIES: tyrosine recombinase | taxID used:135614 OG02013|WP\_010371840.1 | MULTISPECIES: NAD-dependent epimera | taxID used:135614 OG02014|WP\_010367102.1 | MULTISPECIES: phosphoglycerate muta | taxID used:135614 OG02015|WP\_010364201.1 | MULTISPECIES: ADP-ribosylglycohydro | taxID used:135614 OG02016|WP\_010372966.1 | endonuclease/exonuclease/phosphatas | taxID used:135614 OG02017|WP\_010367950.1 | MULTISPECIES: DUF808 domain-contain | taxID used:135614 OG02018|WP\_010369059.1 | MULTISPECIES: LysR family transcrip | taxID used:135614 OG02019|WP\_010374883.1 | MULTISPECIES: hypothetical protein | taxID used:135614 OG00201|WP\_010370355.1 | MULTISPECIES: S9 family peptidase [ | taxID used:135614 OG02020|WP\_010369342.1 | MULTISPECIES: DUF72 domain-containi | taxID used:135614 OG02021|WP\_010370010.1 | lipid A biosynthesis lauroyl acyltr | taxID used:135614 OG02022|WP\_010368340.1 | MULTISPECIES: magnesium transporter | taxID used:135614 OG02023|WP\_017116104.1 | hypothetical protein [Xanthomonas v | taxID used:135614 OG02024|WP\_017112303.1 | MULTISPECIES: hypothetical protein | taxID used:135614 OG02025|WP\_010368054.1 | MULTISPECIES: lipid kinase YegS [Xa | taxID used:135614 OG02026|WP\_010367461.1 | MULTISPECIES: histone deacetylase f | taxID used:135614 OG02027|WP\_010370042.1 | methionyl-tRNA formyltransferase, p | taxID used:135614 OG02028|WP\_010374402.1 | MULTISPECIES: hypothetical protein | taxID used:135614 OG02029|WP\_010365789.1 | MULTISPECIES: acyl-CoA thioesterase | taxID used:135614 OG00202|WP\_033011892.1 | MULTISPECIES: membrane-bound PQQ-de | taxID used:135614 OG02030|WP\_010382061.1 | MULTISPECIES: tryptophan 2,3-dioxyg | taxID used:135614 OG02031|WP\_010367619.1 | MULTISPECIES: UDP-3-O-[3-hydroxymyr | taxID used:135614 OG02032|WP\_010369533.1 | MULTISPECIES: 23S rRNA (adenine(203 | taxID used:135614 OG02033|WP\_010363663.1 | MULTISPECIES: GNAT family N-acetylt | taxID used:135614 OG02034|WP\_017115756.1 | MULTISPECIES: 4-hydroxybenzoate oct | taxID used:135614 OG02035|WP\_010371371.1 | GTP cyclohydrolase I FolE2, partial | taxID used:135614 OG02036|WP\_116652163.1 | hypothetical protein [Xanthomonas v | taxID used:135614 OG02037|WP\_082346465.1 | MULTISPECIES: rRNA methyltransferas | taxID used:135614 OG02038|WP\_010370218.1 | replication protein C, partial [Xan | taxID used:135614 OG02039|WP\_017119001.1 | MULTISPECIES: AraC family transcrip | taxID used:135614 OG00203|WP\_017121487.1 | conjugal transfer protein TrbI [Xan | taxID used:135614 OG02040|WP\_010371433.1 | EamA/RhaT family transporter [Xanth | taxID used:135614 OG02041|WP\_010366129.1 | MULTISPECIES: membrane protein [Xan | taxID used:135614 OG02042|WP\_010365603.1 | MULTISPECIES: MCE family protein [X | taxID used:135614 OG02043|WP\_010372547.1 | spermidine/putrescine ABC transport | taxID used:135614 OG02044|WP\_043089965.1 | LysR family transcriptional regulat | taxID used:135614 OG02046|WP\_017112150.1 | MULTISPECIES: hypothetical protein | taxID used:135614 OG02047|WP\_010371711.1 | MULTISPECIES: malonate decarboxylas | taxID used:135614 OG02048|WP\_010364177.1 | MULTISPECIES: pyrroloquinoline quin | taxID used:135614 OG02049|WP\_010374720.1 | MULTISPECIES: ParB/RepB/Spo0J famil | taxID used:135614 OG00204|WP\_010366593.1 | MULTISPECIES: NAD-dependent DNA lig | taxID used:135614 OG02050|WP\_010374858.1 | MULTISPECIES: EamA family transport | taxID used:135614 OG02051|WP\_010370761.1 | MULTISPECIES: phenylalanine 4-monoo | taxID used:135614 OG02052|WP\_010363898.1 | MULTISPECIES: LysR family transcrip | taxID used:135614 OG02053|WP\_010363279.1 | plasmid mobilization protein, parti | taxID used:135614 OG02054|WP\_010370693.1 | MULTISPECIES: AEC family transporte | taxID used:135614 OG02056|WP\_010371722.1 | MULTISPECIES: biotin-independent ma | taxID used:135614 OG02057|WP\_010371225.1 | MULTISPECIES: tRNA pseudouridine(55 | taxID used:135614 OG02058|WP\_010378912.1 | MULTISPECIES: LysR family transcrip | taxID used:135614 OG02059|WP\_010369063.1 | MULTISPECIES: methylenetetrahydrofo | taxID used:135614 OG00205|WP\_017113395.1 | MULTISPECIES: membrane-bound PQQ-de | taxID used:135614 OG02060|WP\_010370363.1 | MULTISPECIES: allantoinase PuuE [Xa | taxID used:135614 OG02061|WP\_010372694.1 | MULTISPECIES: tRNA glutamyl-Q(34) s | taxID used:135614 OG02063|WP\_010365405.1 | MULTISPECIES: manganese catalase fa | taxID used:135614 OG02064|WP\_010369930.1 | MULTISPECIES: LysR family transcrip | taxID used:135614 OG02065|WP\_010368476.1 | SDR family NAD(P)-dependent oxidore | taxID used:135614 OG02066|WP\_010374660.1 | MULTISPECIES: protoheme IX farnesyl | taxID used:135614 OG02067|WP\_106888038.1 | IS5 family transposase, partial [Xa | taxID used:135614 OG02068|WP\_010367433.1 | MULTISPECIES: gluconolactonase [Xan | taxID used:135614 OG02069|WP\_010368280.1 | MULTISPECIES: GGDEF domain-containi | taxID used:135614 OG00206|WP\_084818999.1 | MULTISPECIES: LPS-assembly protein | taxID used:135614 OG02070|WP\_010366209.1 | MULTISPECIES: outer membrane protei | taxID used:135614 OG02071|WP\_010368734.1 | MULTISPECIES: M23 family metallopep | taxID used:135614 OG02072|WP\_126720824.1 | hypothetical protein [Xanthomonas v | taxID used:135614 OG02073|WP\_017113342.1 | MULTISPECIES: helix-turn-helix doma | taxID used:135614 OG02074|WP\_010367173.1 | MULTISPECIES: ATP phosphoribosyltra | taxID used:135614 OG02075|WP\_010365625.1 | MULTISPECIES: dTDP-4-dehydrorhamnos | taxID used:135614 OG02076|WP\_010374670.1 | MULTISPECIES: cytochrome c oxidase | taxID used:135614 OG02077|WP\_010369467.1 | MULTISPECIES: pirin family protein | taxID used:135614 OG02078|WP\_010365492.1 | LysR family transcriptional regulat | taxID used:135614 OG02079|WP\_054393879.1 | pseudouridylate synthase, partial [ | taxID used:135614 OG00207|WP\_017116686.1 | TonB-dependent receptor, partial [X | taxID used:135614 OG02080|WP\_054393937.1 | MULTISPECIES: nicotinate-nucleotide | taxID used:135614 OG02081|WP\_010367474.1 | MULTISPECIES: prolipoprotein diacyl | taxID used:135614 OG02082|WP\_010372808.1 | MULTISPECIES: M23 family metallopep | taxID used:135614 OG02083|WP\_010368308.1 | MULTISPECIES: hydroxymethylbilane s | taxID used:135614 OG02084|WP\_010372932.1 | MULTISPECIES: segregation/condensat | taxID used:135614 OG02085|WP\_017115513.1 | hypothetical protein [Xanthomonas v | taxID used:135614 OG02086|WP\_010368105.1 | MULTISPECIES: membrane protein [Xan | taxID used:135614 OG02087|WP\_026112916.1 | MULTISPECIES: hypothetical protein | taxID used:135614 OG02088|WP\_017170429.1 | hypothetical protein, partial [Xant | taxID used:135614 OG02089|WP\_010373037.1 | MULTISPECIES: bifunctional methylen | taxID used:135614 OG00208|WP\_080764511.1 | MULTISPECIES: helicase SNF2 [Xantho | taxID used:135614 OG02090|WP\_010364403.1 | MULTISPECIES: helix-turn-helix doma | taxID used:135614 OG02091|WP\_010365629.1 | MULTISPECIES: glucose-1-phosphate t | taxID used:135614 OG02092|WP\_010368103.1 | NAD(+) diphosphatase, partial [Xant | taxID used:135614 OG02093|WP\_010366658.1 | acetyl-CoA carboxylase carboxyltran | taxID used:135614 OG02094|WP\_017115997.1 | MULTISPECIES: hypothetical protein | taxID used:135614 OG02095|WP\_010372975.1 | MULTISPECIES: apolipoprotein acyltr | taxID used:135614 OG02096|WP\_010372063.1 | EamA/RhaT family transporter, parti | taxID used:135614 OG02097|WP\_010366366.1 | pyridoxal kinase, partial [Xanthomo | taxID used:135614 OG02098|WP\_010365697.1 | MULTISPECIES: dihydropteroate synth | taxID used:135614 OG02099|WP\_010363448.1 | MULTISPECIES: dihydrodipicolinate s | taxID used:135614 OG00209|WP\_010365997.1 | MULTISPECIES: ligase-associated DNA | taxID used:135614 OG00020|WP\_010373557.1 | MULTISPECIES: hypothetical protein | taxID used:135614 OG02100|WP\_106888041.1 | HlyD family efflux transporter peri | taxID used:135614 OG02101|WP\_039435042.1 | site-specific DNA-methyltransferase | taxID used:135614 OG02103|WP\_010372957.1 | MULTISPECIES: sugar ABC transporter | taxID used:135614 OG02104|WP\_010367023.1 | MULTISPECIES: class A beta-lactamas | taxID used:135614 OG02105|WP\_039442469.1 | WYL domain-containing protein [Xant | taxID used:135614 OG02106|WP\_010374498.1 | MULTISPECIES: recombination-associa | taxID used:135614 OG02107|WP\_026112734.1 | MULTISPECIES: hypothetical protein | taxID used:135614 OG02108|WP\_010365733.1 | MULTISPECIES: alpha/beta fold hydro | taxID used:135614 OG02109|WP\_017117887.1 | hypothetical protein [Xanthomonas v | taxID used:135614 OG00210|WP\_010370069.1 | MULTISPECIES: DNA topoisomerase I [ | taxID used:135614 OG02110|WP\_017116192.1 | alpha/beta hydrolase, partial [Xant | taxID used:135614 OG02111|WP\_010368112.1 | MULTISPECIES: LysR family transcrip | taxID used:135614 OG02112|WP\_010374476.1 | MULTISPECIES: hypothetical protein | taxID used:135614 OG02113|WP\_010369082.1 | MULTISPECIES: NAD-dependent protein | taxID used:135614 OG02114|WP\_010368347.1 | MULTISPECIES: EamA/RhaT family tran | taxID used:135614 OG02115|WP\_010365440.1 | MULTISPECIES: LysR family transcrip | taxID used:135614 OG02116|WP\_033009642.1 | drug/metabolite exporter YedA, part | taxID used:135614 OG02117|WP\_010372412.1 | type I-C CRISPR-associated protein | taxID used:135614 OG02118|WP\_017113063.1 | MULTISPECIES: cytochrome c biogenes | taxID used:135614 OG02119|WP\_026113080.1 | MULTISPECIES: GTPase Era [Xanthomon | taxID used:135614 OG00211|WP\_017116677.1 | glycosyltransferase family 1 protei | taxID used:135614 OG02120|WP\_033012136.1 | MULTISPECIES: 3-hydroxyisobutyrate | taxID used:135614 OG02121|WP\_010375549.1 | MULTISPECIES: clavaminate synthase | taxID used:135614 OG02122|WP\_010365750.1 | MULTISPECIES: 4-hydroxy-tetrahydrod | taxID used:135614 OG02123|WP\_010368939.1 | MULTISPECIES: malonyl-[acyl-carrier | taxID used:135614 OG02124|WP\_010368419.1 | MULTISPECIES: methylisocitrate lyas | taxID used:135614 OG02125|WP\_010374815.1 | MULTISPECIES: TIGR01777 family prot | taxID used:135614 OG02126|WP\_082320287.1 | MULTISPECIES: ferredoxin--NADP redu | taxID used:135614 OG02127|WP\_010364428.1 | MULTISPECIES: membrane protein [Xan | taxID used:135614 OG02128|WP\_039431545.1 | MULTISPECIES: SPOR domain-containin | taxID used:135614 OG02129|WP\_010373029.1 | MULTISPECIES: UTP--glucose-1-phosph | taxID used:135614 OG00212|WP\_026113225.1 | MULTISPECIES: ATP-dependent helicas | taxID used:135614 OG02130|WP\_010367130.1 | MULTISPECIES: hydroxymethylglutaryl | taxID used:135614 OG02131|WP\_080763728.1 | MULTISPECIES: hypothetical protein | taxID used:135614 OG02132|WP\_010363027.1 | P-type conjugative transfer protein | taxID used:135614 OG02133|WP\_026112722.1 | MULTISPECIES: SAM-dependent methylt | taxID used:135614 OG02134|WP\_017170390.1 | MULTISPECIES: hypothetical protein | taxID used:135614 OG02135|WP\_010366382.1 | hydrogen peroxide-inducible genes a | taxID used:135614 OG02136|WP\_017117939.1 | LysR family transcriptional regulat | taxID used:135614 OG02137|WP\_010378449.1 | MULTISPECIES: enoyl-CoA hydratase [ | taxID used:135614 OG02138|WP\_010371230.1 | MULTISPECIES: nicotinate-nucleotide | taxID used:135614 OG02139|WP\_010368226.1 | MULTISPECIES: endonuclease/exonucle | taxID used:135614 OG00213|WP\_017115241.1 | MULTISPECIES: bifunctional aspartat | taxID used:135614 OG02140|WP\_010364433.1 | MULTISPECIES: ion transporter [Xant | taxID used:135614 OG02141|WP\_017116347.1 | MULTISPECIES: PhzF family phenazine | taxID used:135614 OG02142|WP\_010368716.1 | MULTISPECIES: Hsp33 family molecula | taxID used:135614 OG02143|WP\_017115731.1 | MULTISPECIES: N-formylglutamate def | taxID used:135614 OG02144|WP\_087911186.1 | HlyD family efflux transporter peri | taxID used:135614 OG02145|WP\_010369373.1 | MULTISPECIES: PhzF family phenazine | taxID used:135614 OG02146|WP\_017115150.1 | DUF1173 domain-containing protein, | taxID used:135614 OG02147|WP\_010374763.1 | MULTISPECIES: polyamine aminopropyl | taxID used:135614 OG02148|WP\_010381919.1 | MULTISPECIES: hypothetical protein | taxID used:135614 OG02149|WP\_010364044.1 | MULTISPECIES: baseplate assembly pr | taxID used:135614 OG00214|WP\_010372274.1 | phosphoenolpyruvate--protein phosph | taxID used:135614 OG02150|WP\_010370120.1 | MULTISPECIES: RNA polymerase sigma | taxID used:135614 OG02151|WP\_010369552.1 | MULTISPECIES: nitrilase [Xanthomona | taxID used:135614 OG02152|WP\_033009735.1 | MULTISPECIES: hypothetical protein | taxID used:135614 OG02153|WP\_033006651.1 | MULTISPECIES: formyltetrahydrofolat | taxID used:135614 OG02154|WP\_010369655.1 | MULTISPECIES: alpha/beta fold hydro | taxID used:135614 OG02155|WP\_026112187.1 | MULTISPECIES: 30S ribosomal protein | taxID used:135614 OG02156|WP\_010365759.1 | MULTISPECIES: 2-dehydro-3-deoxygala | taxID used:135614 OG02157|WP\_010369215.1 | MULTISPECIES: RNase adapter RapZ [X | taxID used:135614 OG02158|WP\_010374294.1 | MinD/ParA family protein, partial [ | taxID used:135614 OG02159|WP\_010363513.1 | MULTISPECIES: MBL fold metallo-hydr | taxID used:135614 OG00215|WP\_017113320.1 | acyl-CoA dehydrogenase, partial [Xa | taxID used:135614 OG02160|WP\_010363798.1 | MULTISPECIES: prepilin peptidase [X | taxID used:135614 OG02161|WP\_010377758.1 | MULTISPECIES: phytanoyl-CoA dioxyge | taxID used:135614 OG02162|WP\_010373162.1 | MULTISPECIES: LysR family transcrip | taxID used:135614 OG02163|WP\_010372697.1 | MULTISPECIES: protease HtpX [Xantho | taxID used:135614 OG02164|WP\_010368977.1 | MULTISPECIES: CoA transferase subun | taxID used:135614 OG02165|WP\_010367313.1 | MULTISPECIES: 4-(cytidine 5'-diphos | taxID used:135614 OG02166|WP\_010374939.1 | hypothetical protein, partial [Xant | taxID used:135614 OG02167|WP\_010370216.1 | MULTISPECIES: replication protein A | taxID used:135614 OG02168|WP\_084819002.1 | type III secretion system effector | taxID used:135614 OG02169|WP\_010374386.1 | MULTISPECIES: amidohydrolase [Xanth | taxID used:135614 OG00216|WP\_010363013.1 | VirB4 family type IV secretion/conj | taxID used:135614 OG02170|WP\_010365268.1 | MULTISPECIES: chemotaxis protein Ch | taxID used:135614 OG02171|WP\_010364204.1 | MULTISPECIES: energy transducer Ton | taxID used:135614 OG02172|WP\_017115720.1 | rhodanese-like domain-containing pr | taxID used:135614 OG02173|WP\_082345087.1 | gamma-glutamyl-gamma-aminobutyrate | taxID used:135614 OG02174|WP\_026112781.1 | hypothetical protein, partial [Xant | taxID used:135614 OG02175|WP\_017116030.1 | hypothetical protein, partial [Xant | taxID used:135614 OG02176|WP\_010371995.1 | MULTISPECIES: F0F1 ATP synthase sub | taxID used:135614 OG02177|WP\_010374360.1 | MULTISPECIES: peptidylprolyl isomer | taxID used:135614 OG02178|WP\_026112837.1 | MULTISPECIES: folate-binding protei | taxID used:135614 OG02179|WP\_008571326.1 | MULTISPECIES: CBS domain-containing | taxID used:135614 OG00217|WP\_010365509.1 | MULTISPECIES: TonB-dependent recept | taxID used:135614 OG02180|WP\_010366431.1 | MULTISPECIES: ABC transporter ATP-b | taxID used:135614 OG02181|WP\_010367021.1 | MULTISPECIES: LysR family transcrip | taxID used:135614 OG02182|WP\_010371510.1 | MULTISPECIES: SPFH domain-containin | taxID used:135614 OG02183|WP\_010367522.1 | MULTISPECIES: ABC transporter perme | taxID used:135614 OG02184|WP\_017170366.1 | MULTISPECIES: hypothetical protein | taxID used:135614 OG02185|WP\_010366205.1 | MULTISPECIES: succinate--CoA ligase | taxID used:135614 OG02186|WP\_010365381.1 | MULTISPECIES: SDR family NAD(P)-dep | taxID used:135614 OG02187|WP\_026112215.1 | MULTISPECIES: hypothetical protein | taxID used:135614 OG02188|WP\_010374804.1 | MULTISPECIES: alpha/beta fold hydro | taxID used:135614 OG02189|WP\_010365861.1 | MULTISPECIES: histidine kinase [Xan | taxID used:135614 OG00218|WP\_010366154.1 | MULTISPECIES: outer membrane protei | taxID used:135614 OG02190|WP\_010369091.1 | MULTISPECIES: LysR family transcrip | taxID used:135614 OG02191|WP\_010369455.1 | MULTISPECIES: pirin family protein | taxID used:135614 OG02192|WP\_010370412.1 | MULTISPECIES: DUF2884 domain-contai | taxID used:135614 OG02193|WP\_038897223.1 | DNA methyltransferase, partial [Xan | taxID used:135614 OG02194|WP\_010364377.1 | MULTISPECIES: LysR family transcrip | taxID used:135614 OG02195|WP\_010369078.1 | MULTISPECIES: NAD(P)-dependent oxid | taxID used:135614 OG02196|WP\_010371714.1 | triphosphoribosyl-dephospho-CoA syn | taxID used:135614 OG02197|WP\_080764478.1 | MULTISPECIES: hypothetical protein | taxID used:135614 OG02198|WP\_039435790.1 | DNA methyltransferase [Xanthomonas | taxID used:135614 OG02199|WP\_010368714.1 | MULTISPECIES: hypothetical protein | taxID used:135614 OG00219|WP\_087911081.1 | MULTISPECIES: HAMP domain-containin | taxID used:135614 OG00021|WP\_010367276.1 | MULTISPECIES: DNA-directed RNA poly | taxID used:135614 OG02200|WP\_097370604.1 | hypothetical protein, partial [Xant | taxID used:135614 OG02201|WP\_086019511.1 | EamA/RhaT family transporter [Xanth | taxID used:135614 OG02202|WP\_010370780.1 | MULTISPECIES: 5-dehydro-4-deoxy-D-g | taxID used:135614 OG02203|WP\_054393986.1 | MULTISPECIES: methyltransferase dom | taxID used:135614 OG02204|WP\_017117278.1 | MULTISPECIES: cell division protein | taxID used:135614 OG02205|WP\_010366766.1 | MULTISPECIES: polyprenyl synthetase | taxID used:135614 OG02206|WP\_087920681.1 | relaxase, partial [Xanthomonas vasi | taxID used:135614 OG02207|WP\_039431356.1 | ParA family protein [Xanthomonas va | taxID used:135614 OG02208|WP\_010368964.1 | MULTISPECIES: serine protein kinase | taxID used:135614 OG02209|WP\_010365533.1 | MULTISPECIES: hypothetical protein | taxID used:135614 OG00220|WP\_010366341.1 | MULTISPECIES: serine peptidase [Xan | taxID used:135614 OG02210|WP\_010368294.1 | MULTISPECIES: diaminopimelate epime | taxID used:135614 OG02211|WP\_010367745.1 | MULTISPECIES: glycosyltransferase f | taxID used:135614 OG02212|WP\_010366485.1 | MULTISPECIES: phosphate ABC transpo | taxID used:135614 OG02213|WP\_010374389.1 | MULTISPECIES: FAA hydrolase family | taxID used:135614 OG02214|WP\_010366169.1 | MULTISPECIES: elongation factor Ts | taxID used:135614 OG02215|WP\_039440121.1 | MULTISPECIES: SDR family NAD(P)-dep | taxID used:135614 OG02216|WP\_010364847.1 | MULTISPECIES: phosphatase PAP2 fami | taxID used:135614 OG02217|WP\_010372922.1 | MULTISPECIES: glutaminyl-peptide cy | taxID used:135614 OG02218|WP\_010369706.1 | MULTISPECIES: hypothetical protein | taxID used:135614 OG02219|WP\_010363048.1 | MULTISPECIES: hypothetical protein | taxID used:135614 OG00221|WP\_054394007.1 | MULTISPECIES: NdvB protein [Xanthom | taxID used:135614 OG02220|WP\_010366219.1 | MULTISPECIES: AraC family transcrip | taxID used:135614 OG02221|WP\_010374490.1 | MULTISPECIES: alpha/beta hydrolase | taxID used:135614 OG02222|WP\_010371886.1 | MULTISPECIES: membrane protein [Xan | taxID used:135614 OG02223|WP\_010375100.1 | MULTISPECIES: hypothetical protein | taxID used:135614 OG02224|WP\_010373538.1 | MULTISPECIES: helix-turn-helix doma | taxID used:135614 OG02225|WP\_010366646.1 | MULTISPECIES: DUF3034 domain-contai | taxID used:135614 OG02226|WP\_010368448.1 | MULTISPECIES: protease modulator Hf | taxID used:135614 OG02227|WP\_010371601.1 | MULTISPECIES: aldo/keto reductase [ | taxID used:135614 OG02228|WP\_010370938.1 | aminopeptidase N [Xanthomonas vasic | taxID used:135614 OG02229|WP\_052252030.1 | hypothetical protein [Xanthomonas v | taxID used:135614 OG00222|WP\_039433168.1 | beta-hexosaminidase [Xanthomonas va | taxID used:135614 OG02230|WP\_010363652.1 | MULTISPECIES: class I SAM-dependent | taxID used:135614 OG02231|WP\_010374841.1 | MULTISPECIES: ThuA domain-containin | taxID used:135614 OG02232|WP\_010368606.1 | rRNA pseudouridine synthase, partia | taxID used:135614 OG02233|WP\_010367759.1 | MULTISPECIES: hypothetical protein | taxID used:135614 OG02234|WP\_010372480.1 | plasmid replication/partition relat | taxID used:135614 OG02235|WP\_010368595.1 | MULTISPECIES: sulfate ABC transport | taxID used:135614 OG02236|WP\_017117070.1 | MULTISPECIES: tetratricopeptide rep | taxID used:135614 OG02237|WP\_039434103.1 | hypothetical protein [Xanthomonas v | taxID used:135614 OG02238|WP\_010369027.1 | MULTISPECIES: DUF4238 domain-contai | taxID used:135614 OG02239|WP\_017119589.1 | hypothetical protein [Xanthomonas v | taxID used:135614 OG00223|WP\_017116515.1 | MULTISPECIES: penicillin acylase fa | taxID used:135614 OG02240|WP\_010372144.1 | MULTISPECIES: flagellar motor stato | taxID used:135614 OG02241|WP\_010365273.1 | 2-succinyl-6-hydroxy-2,4-cyclohexad | taxID used:135614 OG02242|WP\_026112938.1 | MULTISPECIES: restriction endonucle | taxID used:135614 OG02243|WP\_017112814.1 | MULTISPECIES: CoA pyrophosphatase [ | taxID used:135614 OG02244|WP\_043096298.1 | MULTISPECIES: hypothetical protein | taxID used:135614 OG02245|WP\_010373429.1 | MULTISPECIES: ImpE protein [Xanthom | taxID used:135614 OG02246|WP\_017116390.1 | MULTISPECIES: DUF72 domain-containi | taxID used:135614 OG02247|WP\_017116631.1 | MULTISPECIES: DUF692 domain-contain | taxID used:135614 OG02248|WP\_097370626.1 | MULTISPECIES: IS3 family transposas | taxID used:135614 OG02249|WP\_010368966.1 | MULTISPECIES: IclR family transcrip | taxID used:135614 OG00224|WP\_116894707.1 | type IV secretion system protein Do | taxID used:135614 OG02250|WP\_080762970.1 | MULTISPECIES: carboxylesterase [Xan | taxID used:135614 OG02251|WP\_039441054.1 | hypothetical protein [Xanthomonas v | taxID used:135614 OG02252|WP\_010366694.1 | MULTISPECIES: phosphatidylserine de | taxID used:135614 OG02253|WP\_010366832.1 | MULTISPECIES: thioredoxin [Xanthomo | taxID used:135614 OG02254|WP\_010373751.1 | MULTISPECIES: flagellar biosyntheti | taxID used:135614 OG02256|WP\_017117122.1 | MULTISPECIES: transporter [Xanthomo | taxID used:135614 OG02257|WP\_010365566.1 | MULTISPECIES: S-formylglutathione h | taxID used:135614 OG02258|WP\_017115369.1 | hypothetical protein [Xanthomonas v | taxID used:135614 OG02259|WP\_010369236.1 | MULTISPECIES: DUF3108 domain-contai | taxID used:135614 OG00225|WP\_010368195.1 | MULTISPECIES: alpha-N-acetylglucosa | taxID used:135614 OG02260|WP\_033479974.1 | MULTISPECIES: DUF2063 domain-contai | taxID used:135614 OG02261|WP\_026112506.1 | MULTISPECIES: DUF3298 domain-contai | taxID used:135614 OG02262|WP\_010364020.1 | phage capsid scaffolding protein, p | taxID used:135614 OG02263|WP\_010370030.1 | glycosyltransferase family 2 protei | taxID used:135614 OG02264|WP\_010369318.1 | MULTISPECIES: pyrroline-5-carboxyla | taxID used:135614 OG02265|WP\_010371383.1 | MULTISPECIES: pantoate--beta-alanin | taxID used:135614 OG02266|WP\_010366087.1 | MULTISPECIES: hypothetical protein | taxID used:135614 OG02267|WP\_010373209.1 | MULTISPECIES: alpha/beta hydrolase | taxID used:135614 OG02268|WP\_075286079.1 | MULTISPECIES: NYN domain-containing | taxID used:135614 OG02269|WP\_026112321.1 | MULTISPECIES: class I SAM-dependent | taxID used:135614 OG00226|WP\_017117205.1 | MULTISPECIES: hypothetical protein | taxID used:135614 OG02270|WP\_010365476.1 | hypothetical protein, partial [Xant | taxID used:135614 OG02271|WP\_010366728.1 | MULTISPECIES: iron-sulfur cluster c | taxID used:135614 OG02272|WP\_010368470.1 | NAD(P)H-hydrate dehydratase, partia | taxID used:135614 OG02273|WP\_087910829.1 | MULTISPECIES: rhomboid family intra | taxID used:135614 OG02274|WP\_010364600.1 | MULTISPECIES: MBL fold metallo-hydr | taxID used:135614 OG02275|WP\_010372500.1 | MULTISPECIES: formate dehydrogenase | taxID used:135614 OG02276|WP\_010364509.1 | hypothetical protein, partial [Xant | taxID used:135614 OG02277|WP\_010366483.1 | MULTISPECIES: phosphate ABC transpo | taxID used:135614 OG02278|WP\_010367388.1 | MULTISPECIES: peptide chain release | taxID used:135614 OG02279|WP\_010367438.1 | MULTISPECIES: p-hydroxycinnamoyl Co | taxID used:135614 OG00227|WP\_010364194.1 | penicillin-binding protein 1B, part | taxID used:135614 OG02280|WP\_010367606.1 | MULTISPECIES: methylenetetrahydrofo | taxID used:135614 OG02281|WP\_082337879.1 | MULTISPECIES: hypothetical protein | taxID used:135614 OG02282|WP\_010372959.1 | MULTISPECIES: carbohydrate ABC tran | taxID used:135614 OG02283|WP\_026112894.1 | MULTISPECIES: spermidine/putrescine | taxID used:135614 OG02284|WP\_010367770.1 | MULTISPECIES: general secretion pat | taxID used:135614 OG02285|WP\_010366875.1 | MULTISPECIES: MipA/OmpV family prot | taxID used:135614 OG02286|WP\_010365879.1 | MULTISPECIES: chemotaxis protein Ch | taxID used:135614 OG02287|WP\_010369969.1 | toprim domain-containing protein [X | taxID used:135614 OG02288|WP\_010365668.1 | MULTISPECIES: 2-dehydro-3-deoxyphos | taxID used:135614 OG02289|WP\_010365601.1 | MULTISPECIES: ABC transporter ATP-b | taxID used:135614 OG00228|WP\_010368585.1 | MULTISPECIES: TonB-dependent recept | taxID used:135614 OG02290|WP\_010374937.1 | MULTISPECIES: shikimate dehydrogena | taxID used:135614 OG02291|WP\_039437120.1 | hypothetical protein [Xanthomonas v | taxID used:135614 OG02292|WP\_010371059.1 | MULTISPECIES: 50S ribosomal protein | taxID used:135614 OG02293|WP\_017123085.1 | DUF3037 domain-containing protein, | taxID used:135614 OG02294|WP\_010369782.1 | MULTISPECIES: glycosyltransferase f | taxID used:135614 OG02295|WP\_010370735.1 | MULTISPECIES: ATP-binding cassette | taxID used:135614 OG02296|WP\_010367551.1 | MULTISPECIES: methyltransferase dom | taxID used:135614 OG02297|WP\_017115654.1 | MULTISPECIES: ferritin-like domain- | taxID used:135614 OG02298|WP\_010363654.1 | MULTISPECIES: chain-length determin | taxID used:135614 OG02299|WP\_010372749.1 | MULTISPECIES: inositol monophosphat | taxID used:135614 OG00229|WP\_010368518.1 | MULTISPECIES: endopeptidase La [Xan | taxID used:135614 OG00022|WP\_010364159.1 | ATP-dependent RNA helicase HrpA, pa | taxID used:135614 OG02300|WP\_026112982.1 | MULTISPECIES: hypothetical protein | taxID used:135614 OG02301|WP\_080762825.1 | MULTISPECIES: pseudouridylate synth | taxID used:135614 OG02302|WP\_010373220.1 | MULTISPECIES: phosphoenolpyruvate s | taxID used:135614 OG02303|WP\_010370107.1 | MULTISPECIES: NYN domain-containing | taxID used:135614 OG02304|WP\_010368986.1 | aquaporin family protein [Xanthomon | taxID used:135614 OG02305|WP\_123184335.1 | hypothetical protein, partial [Xant | taxID used:135614 OG02306|WP\_010367520.1 | MULTISPECIES: ABC transporter ATP-b | taxID used:135614 OG02307|WP\_017117497.1 | MULTISPECIES: STAS domain-containin | taxID used:135614 OG02308|WP\_010368271.1 | MULTISPECIES: sulfite exporter TauE | taxID used:135614 OG02309|WP\_010374712.1 | MULTISPECIES: exodeoxyribonuclease | taxID used:135614 OG00230|WP\_010364803.1 | MULTISPECIES: DNA topoisomerase (AT | taxID used:135614 OG02310|WP\_086019434.1 | MULTISPECIES: response regulator [X | taxID used:135614 OG02311|WP\_010364801.1 | MULTISPECIES: CPBP family intramemb | taxID used:135614 OG02312|WP\_010366286.1 | MULTISPECIES: tRNA threonylcarbamoy | taxID used:135614 OG02313|WP\_010365445.1 | MULTISPECIES: dioxygenase [Xanthomo | taxID used:135614 OG02314|WP\_010368980.1 | MULTISPECIES: DeoR family transcrip | taxID used:135614 OG02315|WP\_010372920.1 | amidohydrolase, partial [Xanthomona | taxID used:135614 OG02316|WP\_053012819.1 | restriction endonuclease subunit M | taxID used:135614 OG02317|WP\_100243350.1 | MULTISPECIES: FRG domain-containing | taxID used:135614 OG02318|WP\_010363896.1 | MULTISPECIES: preprotein translocas | taxID used:135614 OG02319|WP\_010370605.1 | MULTISPECIES: glycosyltransferase f | taxID used:135614 OG00231|WP\_039439212.1 | toprim domain-containing protein [X | taxID used:135614 OG02320|WP\_010365584.1 | thiol:disulfide interchange protein | taxID used:135614 OG02321|WP\_010374905.1 | MULTISPECIES: zinc-dependent peptid | taxID used:135614 OG02322|WP\_026112162.1 | MULTISPECIES: hypothetical protein | taxID used:135614 OG02323|WP\_010367928.1 | MULTISPECIES: EscT/YscT/HrcT family | taxID used:135614 OG02324|WP\_026112460.1 | MULTISPECIES: hypothetical protein | taxID used:135614 OG02325|WP\_082345046.1 | 3'-5' exonuclease [Xanthomonas vasi | taxID used:135614 OG02327|WP\_010374427.1 | MULTISPECIES: hypothetical protein | taxID used:135614 OG02328|WP\_010370076.1 | hypothetical protein, partial [Xant | taxID used:135614 OG02329|WP\_010369701.1 | MULTISPECIES: hypothetical protein | taxID used:135614 OG00232|WP\_017117366.1 | MULTISPECIES: DUF2309 domain-contai | taxID used:135614 OG02330|WP\_024420136.1 | MULTISPECIES: hypothetical protein | taxID used:135614 OG02331|WP\_010375526.1 | MULTISPECIES: peptidase C1 [Xanthom | taxID used:135614 OG02332|WP\_010365736.1 | MULTISPECIES: bifunctional hydroxym | taxID used:135614 OG02333|WP\_017116011.1 | HDOD domain-containing protein, par | taxID used:135614 OG02334|WP\_010369184.1 | MULTISPECIES: LuxR family transcrip | taxID used:135614 OG02335|WP\_010363730.1 | MULTISPECIES: NADPH-dependent 7-cya | taxID used:135614 OG02336|WP\_010372010.1 | MULTISPECIES: F0F1 ATP synthase sub | taxID used:135614 OG02337|WP\_086020548.1 | IS5/IS1182 family transposase [Xant | taxID used:135614 OG02338|WP\_010363348.1 | MULTISPECIES: DUF3014 domain-contai | taxID used:135614 OG02339|WP\_010374685.1 | MULTISPECIES: bifunctional DNA-form | taxID used:135614 OG00233|WP\_010366900.1 | MULTISPECIES: GGDEF domain-containi | taxID used:135614 OG02340|WP\_010365187.1 | MULTISPECIES: FHA domain-containing | taxID used:135614 OG02341|WP\_017170357.1 | MULTISPECIES: DUF2974 domain-contai | taxID used:135614 OG02342|WP\_010370653.1 | MULTISPECIES: META domain-containin | taxID used:135614 OG02343|WP\_039441081.1 | hypothetical protein [Xanthomonas v | taxID used:135614 OG02344|WP\_010365965.1 | MULTISPECIES: signal peptidase I [X | taxID used:135614 OG02345|WP\_010372015.1 | MULTISPECIES: hypothetical protein | taxID used:135614 OG02346|WP\_086019516.1 | sulfite exporter TauE/SafE family p | taxID used:135614 OG02347|WP\_010370800.1 | MULTISPECIES: 2-keto-4-pentenoate h | taxID used:135614 OG02348|WP\_026112613.1 | P-type conjugative transfer protein | taxID used:135614 OG02349|WP\_010376920.1 | MULTISPECIES: phospholipase [Xantho | taxID used:135614 OG00234|WP\_010363339.1 | MULTISPECIES: TonB-dependent recept | taxID used:135614 OG02350|WP\_053012819.1 | restriction endonuclease subunit M | taxID used:135614 OG02351|WP\_010374818.1 | histidine biosynthesis protein HisI | taxID used:135614 OG02352|WP\_010367473.1 | MULTISPECIES: thymidylate synthase | taxID used:135614 OG02353|WP\_010369436.1 | MULTISPECIES: ankyrin repeat domain | taxID used:135614 OG02354|WP\_053012854.1 | hypothetical protein [Xanthomonas v | taxID used:135614 OG02355|WP\_017115556.1 | MULTISPECIES: N-acetylmuramoyl-L-al | taxID used:135614 OG02356|WP\_039443844.1 | MULTISPECIES: protein phosphatase 2 | taxID used:135614 OG02357|WP\_010369497.1 | MULTISPECIES: protein-glutamate O-m | taxID used:135614 OG02358|WP\_010374802.1 | MULTISPECIES: hypothetical protein | taxID used:135614 OG02359|WP\_010363996.1 | MULTISPECIES: tol-pal system protei | taxID used:135614 OG00235|WP\_010366199.1 | MULTISPECIES: penicillin acylase fa | taxID used:135614 OG02360|WP\_010367903.1 | MULTISPECIES: hypothetical protein | taxID used:135614 OG02361|WP\_054393998.1 | MULTISPECIES: sulfotransferase [Xan | taxID used:135614 OG02362|WP\_010374358.1 | MULTISPECIES: hypothetical protein | taxID used:135614 OG02363|WP\_017115423.1 | MULTISPECIES: multi-copper polyphen | taxID used:135614 OG02364|WP\_010373311.1 | MULTISPECIES: succinate dehydrogena | taxID used:135614 OG02365|WP\_010372082.1 | SDR family NAD(P)-dependent oxidore | taxID used:135614 OG02366|WP\_010367663.1 | MULTISPECIES: 16S rRNA (cytidine(14 | taxID used:135614 OG02367|WP\_010366162.1 | MULTISPECIES: phosphatidate cytidyl | taxID used:135614 OG02368|WP\_017116411.1 | MULTISPECIES: hypothetical protein | taxID used:135614 OG02369|WP\_010364431.1 | MULTISPECIES: NAD(P)-dependent oxid | taxID used:135614 OG00236|WP\_033009909.1 | MULTISPECIES: penicillin-binding pr | taxID used:135614 OG02370|WP\_026112683.1 | MULTISPECIES: siderophore-interacti | taxID used:135614 OG02371|WP\_017117920.1 | hypothetical protein [Xanthomonas v | taxID used:135614 OG02372|WP\_010363184.1 | SDR family NAD(P)-dependent oxidore | taxID used:135614 OG02373|WP\_039438732.1 | hypothetical protein, partial [Xant | taxID used:135614 OG02374|WP\_017113519.1 | MULTISPECIES: M48 family peptidase | taxID used:135614 OG02375|WP\_010363366.1 | MULTISPECIES: aliphatic sulfonate A | taxID used:135614 OG02376|WP\_010379825.1 | MULTISPECIES: 1-acyl-sn-glycerol-3- | taxID used:135614 OG02377|WP\_010368069.1 | MULTISPECIES: S-adenosylmethionine | taxID used:135614 OG02378|WP\_010371380.1 | MULTISPECIES: 3-methyl-2-oxobutanoa | taxID used:135614 OG02379|WP\_087911172.1 | hypothetical protein [Xanthomonas v | taxID used:135614 OG00237|WP\_039438753.1 | TonB-dependent receptor [Xanthomona | taxID used:135614 OG02380|WP\_010378754.1 | MULTISPECIES: zinc transporter ZupT | taxID used:135614 OG02381|WP\_010380616.1 | MULTISPECIES: response regulator tr | taxID used:135614 OG02382|WP\_082345093.1 | MULTISPECIES: DUF3426 domain-contai | taxID used:135614 OG02383|WP\_017115867.1 | MULTISPECIES: flagellar brake prote | taxID used:135614 OG02384|WP\_054393927.1 | MULTISPECIES: SDR family NAD(P)-dep | taxID used:135614 OG02385|WP\_116645410.1 | NYN domain-containing protein [Xant | taxID used:135614 OG02386|WP\_010368673.1 | MULTISPECIES: 3'(2'),5'-bisphosphat | taxID used:135614 OG02387|WP\_010363524.1 | MULTISPECIES: polysaccharide pyruvy | taxID used:135614 OG02388|WP\_017115589.1 | MULTISPECIES: N-formylglutamate ami | taxID used:135614 OG02389|WP\_017116796.1 | MULTISPECIES: tryptophan synthase s | taxID used:135614 OG00238|WP\_017115345.1 | MULTISPECIES: two-component system | taxID used:135614 OG02390|WP\_010363214.1 | hypothetical protein [Xanthomonas v | taxID used:135614 OG02391|WP\_010366170.1 | MULTISPECIES: 30S ribosomal protein | taxID used:135614 OG02392|WP\_010363680.1 | MULTISPECIES: hypothetical protein | taxID used:135614 OG02393|WP\_017116088.1 | MULTISPECIES: hypothetical protein | taxID used:135614 OG02394|WP\_010368307.1 | MULTISPECIES: DUF481 domain-contain | taxID used:135614 OG02395|WP\_010369691.1 | MULTISPECIES: taurine catabolism di | taxID used:135614 OG02396|WP\_080765162.1 | hypothetical protein [Xanthomonas v | taxID used:135614 OG02397|WP\_010371608.1 | MULTISPECIES: hypothetical protein | taxID used:135614 OG02398|WP\_017117598.1 | 2,5-didehydrogluconate reductase Dk | taxID used:135614 OG02399|WP\_010365582.1 | cytochrome c4, partial [Xanthomonas | taxID used:135614 OG00239|WP\_017115610.1 | MULTISPECIES: FtsX-like permease fa | taxID used:135614 OG00023|WP\_010372470.1 | hybrid sensor histidine kinase/resp | taxID used:135614 OG02400|WP\_010368220.1 | MULTISPECIES: DUF2242 domain-contai | taxID used:135614 OG02401|WP\_010371150.1 | SDR family NAD(P)-dependent oxidore | taxID used:135614 OG02402|WP\_010362980.1 | MULTISPECIES: AAA family ATPase [Xa | taxID used:135614 OG02403|WP\_010366073.1 | MULTISPECIES: DUF1295 domain-contai | taxID used:135614 OG02404|WP\_010379135.1 | MULTISPECIES: D-alanyl-D-alanine ca | taxID used:135614 OG02405|WP\_010369272.1 | MULTISPECIES: DUF4198 domain-contai | taxID used:135614 OG02406|WP\_080762822.1 | MULTISPECIES: uroporphyrinogen-III | taxID used:135614 OG02407|WP\_010369261.1 | MULTISPECIES: hypothetical protein | taxID used:135614 OG02408|WP\_010367323.1 | MULTISPECIES: polyphosphate kinase | taxID used:135614 OG02409|WP\_010366855.1 | MULTISPECIES: DUF4349 domain-contai | taxID used:135614 OG00240|WP\_010367005.1 | MULTISPECIES: TonB-dependent recept | taxID used:135614 OG02410|WP\_010369858.1 | MULTISPECIES: ABC transporter perme | taxID used:135614 OG02411|WP\_086019552.1 | MULTISPECIES: glycosyltransferase [ | taxID used:135614 OG02412|WP\_010369384.1 | MULTISPECIES: dienelactone hydrolas | taxID used:135614 OG02413|WP\_010367795.1 | MULTISPECIES: hypothetical protein | taxID used:135614 OG02414|WP\_010366428.1 | MULTISPECIES: hypothetical protein | taxID used:135614 OG02415|WP\_010371553.1 | MULTISPECIES: septum site-determini | taxID used:135614 OG02416|WP\_017116708.1 | MULTISPECIES: hypothetical protein | taxID used:135614 OG02417|WP\_010368969.1 | MULTISPECIES: 3-oxoadipate enol-lac | taxID used:135614 OG02418|WP\_010365674.1 | MULTISPECIES: 2-C-methyl-D-erythrit | taxID used:135614 OG02419|WP\_100214363.1 | MULTISPECIES: hypothetical protein | taxID used:135614 OG00241|WP\_017116465.1 | MULTISPECIES: glycoside hydrolase f | taxID used:135614 OG02420|WP\_010367764.1 | general secretion pathway protein G | taxID used:135614 OG02421|WP\_010366148.1 | MULTISPECIES: acyl-ACP--UDP-N-acety | taxID used:135614 OG02422|WP\_010381151.1 | MULTISPECIES: avirulence protein [X | taxID used:135614 OG02423|WP\_010365527.1 | MULTISPECIES: polysaccharide deacet | taxID used:135614 OG02424|WP\_010366184.1 | MULTISPECIES: type I methionyl amin | taxID used:135614 OG02425|WP\_010365941.1 | MULTISPECIES: enoyl-CoA hydratase [ | taxID used:135614 OG02427|WP\_010366292.1 | MULTISPECIES: glycine zipper 2TM do | taxID used:135614 OG02428|WP\_010374496.1 | DUF45 domain-containing protein [Xa | taxID used:135614 OG02429|WP\_010363758.1 | hypothetical protein [Xanthomonas v | taxID used:135614 OG00242|WP\_080763716.1 | RHS repeat protein [Xanthomonas vas | taxID used:135614 OG02430|WP\_039439245.1 | hypothetical protein [Xanthomonas v | taxID used:135614 OG02431|WP\_010364675.1 | MULTISPECIES: DUF1868 domain-contai | taxID used:135614 OG02432|WP\_100229188.1 | hypothetical protein [Xanthomonas v | taxID used:135614 OG02433|WP\_010365688.1 | MULTISPECIES: LysM peptidoglycan-bi | taxID used:135614 OG02434|WP\_039431434.1 | conjugal transfer protein, partial | taxID used:135614 OG02435|WP\_010372053.1 | MULTISPECIES: MetQ/NlpA family ABC | taxID used:135614 OG02436|WP\_026112294.1 | hypothetical protein, partial [Xant | taxID used:135614 OG02437|WP\_010364383.1 | MULTISPECIES: class I SAM-dependent | taxID used:135614 OG02438|WP\_017112625.1 | MULTISPECIES: hypothetical protein | taxID used:135614 OG02439|WP\_010374354.1 | MULTISPECIES: exodeoxyribonuclease | taxID used:135614 OG00243|WP\_010369731.1 | S9 family peptidase, partial [Xanth | taxID used:135614 OG02440|WP\_010370651.1 | MULTISPECIES: undecaprenyl-diphosph | taxID used:135614 OG02441|WP\_010368065.1 | MULTISPECIES: indole-3-glycerol pho | taxID used:135614 OG02442|WP\_010363952.1 | MULTISPECIES: DUF3011 domain-contai | taxID used:135614 OG02443|WP\_010366672.1 | MULTISPECIES: tRNA pseudouridine(38 | taxID used:135614 OG02444|WP\_010363185.1 | MULTISPECIES: SDR family NAD(P)-dep | taxID used:135614 OG02445|WP\_010374718.1 | MULTISPECIES: ParA family protein [ | taxID used:135614 OG02446|WP\_010367469.1 | MULTISPECIES: 16S rRNA (adenine(151 | taxID used:135614 OG02447|WP\_010366270.1 | MULTISPECIES: ferredoxin--NADP redu | taxID used:135614 OG02448|WP\_010373332.1 | MULTISPECIES: 3-deoxy-manno-octulos | taxID used:135614 OG02449|WP\_010371848.1 | MULTISPECIES: ABC transporter perme | taxID used:135614 OG00244|WP\_010364499.1 | MULTISPECIES: DUF1631 domain-contai | taxID used:135614 OG02451|WP\_010363003.1 | lytic transglycosylase [Xanthomonas | taxID used:135614 OG02452|WP\_017112923.1 | MULTISPECIES: DUF72 domain-containi | taxID used:135614 OG02453|WP\_010367572.1 | MULTISPECIES: class I SAM-dependent | taxID used:135614 OG02454|WP\_087911031.1 | IS5 family transposase, partial [Xa | taxID used:135614 OG02455|WP\_087911031.1 | IS5 family transposase [Xanthomonas | taxID used:135614 OG02456|WP\_084819016.1 | IS5 family transposase, partial [Xa | taxID used:135614 OG02457|WP\_017115411.1 | MULTISPECIES: aliphatic sulfonate A | taxID used:135614 OG02458|WP\_017118559.1 | type I methionyl aminopeptidase, pa | taxID used:135614 OG02459|WP\_017112429.1 | MULTISPECIES: rhomboid family intra | taxID used:135614 OG00245|WP\_039440908.1 | restriction endonuclease [Xanthomon | taxID used:135614 OG02460|WP\_010373615.1 | MULTISPECIES: flagellar basal-body | taxID used:135614 OG02461|WP\_010364602.1 | MULTISPECIES: endonuclease [Xanthom | taxID used:135614 OG02462|WP\_010371000.1 | MULTISPECIES: thiazole synthase [Xa | taxID used:135614 OG02463|WP\_026112740.1 | MULTISPECIES: class I SAM-dependent | taxID used:135614 OG02464|WP\_039443026.1 | MULTISPECIES: P-type conjugative tr | taxID used:135614 OG02465|WP\_010364916.1 | MULTISPECIES: ATP-binding cassette | taxID used:135614 OG02466|WP\_010368346.1 | MULTISPECIES: transporter substrate | taxID used:135614 OG02467|WP\_010365006.1 | MULTISPECIES: ParA family protein [ | taxID used:135614 OG02468|WP\_010367160.1 | MULTISPECIES: imidazole glycerol ph | taxID used:135614 OG02469|WP\_010368976.1 | MULTISPECIES: CoA-transferase subun | taxID used:135614 OG00246|WP\_010373325.1 | MULTISPECIES: DNA internalization-r | taxID used:135614 OG02471|WP\_026112801.1 | enoyl-CoA hydratase, partial [Xanth | taxID used:135614 OG02472|WP\_010373757.1 | MULTISPECIES: flagellar biosyntheti | taxID used:135614 OG02473|WP\_087911117.1 | hypothetical protein [Xanthomonas v | taxID used:135614 OG02474|WP\_026112304.1 | MULTISPECIES: response regulator tr | taxID used:135614 OG02475|WP\_116652145.1 | IS5 family transposase, partial [Xa | taxID used:135614 OG02476|WP\_017117237.1 | EcsC family protein [Xanthomonas va | taxID used:135614 OG02477|WP\_010365682.1 | MULTISPECIES: 5'/3'-nucleotidase Su | taxID used:135614 OG02478|WP\_010365897.1 | MULTISPECIES: membrane protein [Xan | taxID used:135614 OG02479|WP\_087910765.1 | MULTISPECIES: type III secretion sy | taxID used:135614 OG00247|WP\_026112689.1 | MULTISPECIES: glycoside hydrolase f | taxID used:135614 OG02480|WP\_017170424.1 | MULTISPECIES: hypothetical protein | taxID used:135614 OG02481|WP\_010370081.1 | MULTISPECIES: KR domain-containing | taxID used:135614 OG02482|WP\_010371574.1 | MULTISPECIES: phosphatase PAP2 fami | taxID used:135614 OG02483|WP\_010364543.1 | MULTISPECIES: fimbrial protein [Xan | taxID used:135614 OG02484|WP\_010368092.1 | hypothetical protein, partial [Xant | taxID used:135614 OG02485|WP\_010380112.1 | MULTISPECIES: NAD kinase [Xanthomon | taxID used:135614 OG02486|WP\_010366119.1 | MULTISPECIES: VUT family protein [X | taxID used:135614 OG02487|WP\_026112323.1 | hypothetical protein, partial [Xant | taxID used:135614 OG02488|WP\_010371311.1 | type IV secretion pathway protein V | taxID used:135614 OG02489|WP\_116645480.1 | hypothetical protein [Xanthomonas v | taxID used:135614 OG00248|WP\_026112819.1 | Tat pathway signal protein [Xanthom | taxID used:135614 OG02490|WP\_010365590.1 | MULTISPECIES: CDP-diacylglycerol--s | taxID used:135614 OG02491|WP\_017112413.1 | MULTISPECIES: hypothetical protein | taxID used:135614 OG02492|WP\_010368574.1 | MULTISPECIES: colicin V biosynthesi | taxID used:135614 OG02493|WP\_010368469.1 | MULTISPECIES: histidine phosphatase | taxID used:135614 OG02494|WP\_010366288.1 | MULTISPECIES: TatD family deoxyribo | taxID used:135614 OG02495|WP\_010366164.1 | MULTISPECIES: di-trans,poly-cis-dec | taxID used:135614 OG02496|WP\_010373505.1 | MULTISPECIES: DUF1264 domain-contai | taxID used:135614 OG02497|WP\_010365490.1 | molybdenum ABC transporter substrat | taxID used:135614 OG02498|WP\_010364622.1 | MULTISPECIES: hypothetical protein | taxID used:135614 OG02499|WP\_010368463.1 | MULTISPECIES: hypothetical protein | taxID used:135614 OG00249|WP\_026112817.1 | MULTISPECIES: S9 family peptidase [ | taxID used:135614 OG00024|WP\_010367790.1 | MULTISPECIES: phosphoribosylformylg | taxID used:135614 OG02500|WP\_010373540.1 | MULTISPECIES: type IV pilus biogene | taxID used:135614 OG02501|WP\_010368937.1 | SDR family NAD(P)-dependent oxidore | taxID used:135614 OG02502|WP\_010363983.1 | MULTISPECIES: protein TolQ [Xanthom | taxID used:135614 OG02503|WP\_080765176.1 | KilA-N domain-containing protein [X | taxID used:135614 OG02504|WP\_010365419.1 | MULTISPECIES: response regulator tr | taxID used:135614 OG02505|WP\_010371951.1 | MULTISPECIES: hypothetical protein | taxID used:135614 OG02506|WP\_010374387.1 | MULTISPECIES: SDR family NAD(P)-dep | taxID used:135614 OG02507|WP\_010368508.1 | MULTISPECIES: hydroxyacylglutathion | taxID used:135614 OG02508|WP\_010370997.1 | tRNA (guanosine(46)-N7)-methyltrans | taxID used:135614 OG02509|WP\_010365202.1 | MULTISPECIES: energy transducer Ton | taxID used:135614 OG00250|WP\_017117247.1 | MULTISPECIES: glycoside hydrolase f | taxID used:135614 OG02510|WP\_010363620.1 | MULTISPECIES: YdcF family protein [ | taxID used:135614 OG02511|WP\_100229205.1 | MULTISPECIES: type III secretion sy | taxID used:135614 OG02512|WP\_017113079.1 | MULTISPECIES: hypothetical protein | taxID used:135614 OG02513|WP\_026112139.1 | MULTISPECIES: molecular chaperone [ | taxID used:135614 OG02514|WP\_010363684.1 | MULTISPECIES: zf-HC2 domain-contain | taxID used:135614 OG02515|WP\_010363688.1 | MULTISPECIES: heme ABC transporter | taxID used:135614 OG02516|WP\_010367653.1 | MULTISPECIES: hypothetical protein | taxID used:135614 OG02517|WP\_010374473.1 | MULTISPECIES: glutamine amidotransf | taxID used:135614 OG02518|WP\_010373170.1 | MULTISPECIES: SDR family NAD(P)-dep | taxID used:135614 OG02519|WP\_026112891.1 | ubiquinol cytochrome C oxidoreducta | taxID used:135614 OG00251|WP\_010366934.1 | MULTISPECIES: TonB-dependent recept | taxID used:135614 OG02520|WP\_017116596.1 | MULTISPECIES: endonuclease [Xanthom | taxID used:135614 OG02521|WP\_010366504.1 | MULTISPECIES: enoyl-CoA hydratase [ | taxID used:135614 OG02522|WP\_010364597.1 | MULTISPECIES: molybdate ABC transpo | taxID used:135614 OG02523|WP\_010372905.1 | MULTISPECIES: heme ABC transporter | taxID used:135614 OG02524|WP\_017115456.1 | MULTISPECIES: GntR family transcrip | taxID used:135614 OG02525|WP\_010368994.1 | MULTISPECIES: SDR family NAD(P)-dep | taxID used:135614 OG02526|WP\_017170369.1 | MULTISPECIES: hypothetical protein | taxID used:135614 OG02527|WP\_010363025.1 | virB8 family protein [Xanthomonas v | taxID used:135614 OG02528|WP\_010367232.1 | sugar ABC transporter permease [Xan | taxID used:135614 OG02529|WP\_010368281.1 | bifunctional demethylmenaquinone me | taxID used:135614 OG00252|WP\_010363005.1 | type I DNA topoisomerase [Xanthomon | taxID used:135614 OG02530|WP\_010374297.1 | FliA/WhiG family RNA polymerase sig | taxID used:135614 OG02531|WP\_010367919.1 | MULTISPECIES: EscJ/YscJ/HrcJ family | taxID used:135614 OG02532|WP\_082347462.1 | restriction endonuclease subunit M, | taxID used:135614 OG02533|WP\_126720773.1 | hypothetical protein [Xanthomonas v | taxID used:135614 OG02534|WP\_010365910.1 | MULTISPECIES: tRNA (guanosine(37)-N | taxID used:135614 OG02535|WP\_010368390.1 | MULTISPECIES: transporter [Xanthomo | taxID used:135614 OG02537|WP\_017170361.1 | MULTISPECIES: hypothetical protein | taxID used:135614 OG02538|WP\_080762937.1 | hypothetical protein, partial [Xant | taxID used:135614 OG02539|WP\_010369277.1 | MULTISPECIES: sel1 repeat family pr | taxID used:135614 OG00253|WP\_010367008.1 | MULTISPECIES: TonB-dependent recept | taxID used:135614 OG02540|WP\_010369785.1 | MULTISPECIES: hypothetical protein | taxID used:135614 OG02541|WP\_010372609.1 | MULTISPECIES: SDR family NAD(P)-dep | taxID used:135614 OG02542|WP\_010368059.1 | MULTISPECIES: DUF541 domain-contain | taxID used:135614 OG02543|WP\_010373348.1 | MULTISPECIES: sulfurtransferase [Xa | taxID used:135614 OG02544|WP\_010363296.1 | MULTISPECIES: MBL fold metallo-hydr | taxID used:135614 OG02546|WP\_017115239.1 | MULTISPECIES: cyclic nucleotide-bin | taxID used:135614 OG02547|WP\_010371168.1 | MULTISPECIES: NADH-quinone oxidored | taxID used:135614 OG02548|WP\_010380640.1 | MULTISPECIES: enoyl-CoA hydratase/i | taxID used:135614 OG02549|WP\_017116068.1 | MULTISPECIES: leucyl/phenylalanyl-t | taxID used:135614 OG00254|WP\_010363432.1 | MULTISPECIES: S9 family peptidase [ | taxID used:135614 OG02550|WP\_087911190.1 | hypothetical protein [Xanthomonas v | taxID used:135614 OG02551|WP\_010369120.1 | MULTISPECIES: PspA/IM30 family prot | taxID used:135614 OG02552|WP\_005912445.1 | MULTISPECIES: MotA/TolQ/ExbB proton | taxID used:135614 OG02553|WP\_010379207.1 | MULTISPECIES: DNA/RNA non-specific | taxID used:135614 OG02554|WP\_010372525.1 | gamma-glutamyl-gamma-aminobutyrate | taxID used:135614 OG02555|WP\_010373611.1 | MULTISPECIES: flagellar basal-body | taxID used:135614 OG02556|WP\_010364175.1 | MULTISPECIES: pyrroloquinoline-quin | taxID used:135614 OG02557|WP\_010367131.1 | MULTISPECIES: 3-hydroxy-2-methylbut | taxID used:135614 OG02558|WP\_010363152.1 | MULTISPECIES: EAL domain-containing | taxID used:135614 OG02559|WP\_039431363.1 | TIGR03761 family integrating conjug | taxID used:135614 OG00255|WP\_010372503.1 | MULTISPECIES: FdhF/YdeP family oxid | taxID used:135614 OG02560|WP\_017116728.1 | MULTISPECIES: S-methyl-5'-thioinosi | taxID used:135614 OG02561|WP\_010363294.1 | MULTISPECIES: 3-deoxy-D-manno-octul | taxID used:135614 OG02562|WP\_010364763.1 | MULTISPECIES: pyridoxine 5'-phospha | taxID used:135614 OG02563|WP\_029218439.1 | hypothetical protein, partial [Xant | taxID used:135614 OG02564|WP\_010366995.1 | MULTISPECIES: 4-hydroxy-2-oxovalera | taxID used:135614 OG02565|WP\_010368532.1 | MULTISPECIES: Bax inhibitor-1/YccA | taxID used:135614 OG02566|WP\_010368935.1 | MULTISPECIES: pimeloyl-[acyl-carrie | taxID used:135614 OG02567|WP\_010366138.1 | tetratricopeptide repeat protein [X | taxID used:135614 OG02568|WP\_010368267.1 | MULTISPECIES: phosphocholine cytidy | taxID used:135614 OG02569|WP\_010369472.1 | MULTISPECIES: 2,3-bisphosphoglycera | taxID used:135614 OG00256|WP\_017115747.1 | MULTISPECIES: mechanosensitive ion | taxID used:135614 OG02570|WP\_017115205.1 | MULTISPECIES: HEPN domain-containin | taxID used:135614 OG02571|WP\_010363335.1 | trehalose-phosphatase, partial [Xan | taxID used:135614 OG02572|WP\_010367976.1 | SDR family NAD(P)-dependent oxidore | taxID used:135614 OG02573|WP\_010364574.1 | MULTISPECIES: SDR family NAD(P)-dep | taxID used:135614 OG02574|WP\_080764639.1 | hypothetical protein [Xanthomonas v | taxID used:135614 OG02575|WP\_010372752.1 | MULTISPECIES: RNA methyltransferase | taxID used:135614 OG02576|WP\_010369138.1 | MULTISPECIES: peptide ABC transport | taxID used:135614 OG02577|WP\_010369299.1 | MULTISPECIES: Fe-S cluster assembly | taxID used:135614 OG02578|WP\_010363742.1 | MULTISPECIES: haloacid dehalogenase | taxID used:135614 OG02579|WP\_087911174.1 | hypothetical protein [Xanthomonas v | taxID used:135614 OG00257|WP\_010370278.1 | MULTISPECIES: hypothetical protein | taxID used:135614 OG02580|WP\_010374400.1 | MULTISPECIES: transcriptional regul | taxID used:135614 OG02581|WP\_010374833.1 | MULTISPECIES: membrane protein [Xan | taxID used:135614 OG02582|WP\_010368718.1 | monofunctional biosynthetic peptido | taxID used:135614 OG02583|WP\_017113349.1 | MULTISPECIES: protocatechuate 3,4-d | taxID used:135614 OG02584|WP\_026112794.1 | MULTISPECIES: hypothetical protein | taxID used:135614 OG02585|WP\_010373659.1 | MULTISPECIES: SDR family NAD(P)-dep | taxID used:135614 OG02586|WP\_010373229.1 | MULTISPECIES: 3-hydroxybutyrate deh | taxID used:135614 OG02587|WP\_010374829.1 | MULTISPECIES: HAD family hydrolase | taxID used:135614 OG02588|WP\_010365555.1 | MULTISPECIES: cellulase [Xanthomona | taxID used:135614 OG02589|WP\_039437114.1 | hypothetical protein [Xanthomonas v | taxID used:135614 OG00258|WP\_026112779.1 | carbohydrate-binding protein [Xanth | taxID used:135614 OG02590|WP\_010369751.1 | MULTISPECIES: 1-acyl-sn-glycerol-3- | taxID used:135614 OG02591|WP\_010371764.1 | MULTISPECIES: polyisoprenoid-bindin | taxID used:135614 OG02592|WP\_010382354.1 | MULTISPECIES: beta-ketoacyl-ACP red | taxID used:135614 OG02593|WP\_010374757.1 | MULTISPECIES: NADP-dependent 3-hydr | taxID used:135614 OG02594|WP\_082345100.1 | MULTISPECIES: hypothetical protein | taxID used:135614 OG02595|WP\_010374478.1 | MULTISPECIES: twin-arginine translo | taxID used:135614 OG02596|WP\_017116128.1 | MULTISPECIES: TetR/AcrR family tran | taxID used:135614 OG02597|WP\_010366451.1 | MULTISPECIES: DUF2058 domain-contai | taxID used:135614 OG02598|WP\_010369750.1 | MULTISPECIES: ketosynthase [Xanthom | taxID used:135614 OG02599|WP\_080762854.1 | hypothetical protein [Xanthomonas v | taxID used:135614 OG00259|WP\_010367014.1 | TonB-dependent receptor, partial [X | taxID used:135614 OG00025|WP\_039432078.1 | MULTISPECIES: exodeoxyribonuclease | taxID used:135614 OG02600|WP\_086020492.1 | peptidoglycan-binding protein [Xant | taxID used:135614 OG02601|WP\_017117484.1 | MULTISPECIES: hypothetical protein, | taxID used:135614 OG02602|WP\_106888061.1 | IS5 family transposase [Xanthomonas | taxID used:135614 OG02603|WP\_010377815.1 | EEP domain-containing protein [Xant | taxID used:135614 OG02604|WP\_010371152.1 | MULTISPECIES: triose-phosphate isom | taxID used:135614 OG02605|WP\_017121924.1 | DNA/RNA non-specific endonuclease [ | taxID used:135614 OG02606|WP\_010370777.1 | MULTISPECIES: 2-dehydro-3-deoxy-D-g | taxID used:135614 OG02607|WP\_010368561.1 | MULTISPECIES: UDP-2,3-diacylglucosa | taxID used:135614 OG02609|WP\_042506527.1 | histidine utilization repressor, pa | taxID used:135614 OG00260|WP\_010373216.1 | phosphoenolpyruvate synthase, parti | taxID used:135614 OG02610|WP\_026112428.1 | MULTISPECIES: DUF3348 domain-contai | taxID used:135614 OG02611|WP\_010365504.1 | MULTISPECIES: KR domain-containing | taxID used:135614 OG02612|WP\_002811889.1 | MULTISPECIES: response regulator tr | taxID used:135614 OG02613|WP\_010370576.1 | hypothetical protein, partial [Xant | taxID used:135614 OG02614|WP\_017112764.1 | MULTISPECIES: TetR/AcrR family tran | taxID used:135614 OG02615|WP\_010368928.1 | MULTISPECIES: amidophosphoribosyltr | taxID used:135614 OG02616|WP\_080764600.1 | helix-turn-helix transcriptional re | taxID used:135614 OG02617|WP\_017113113.1 | MULTISPECIES: murein L,D-transpepti | taxID used:135614 OG02618|WP\_017117375.1 | MULTISPECIES: hypothetical protein | taxID used:135614 OG02619|WP\_010369874.1 | MULTISPECIES: ATP-binding cassette | taxID used:135614 OG00261|WP\_010373548.1 | MULTISPECIES: 3-hydroxyacyl-CoA deh | taxID used:135614 OG02620|WP\_010370916.1 | MULTISPECIES: phosphoadenylyl-sulfa | taxID used:135614 OG02621|WP\_010366752.1 | MULTISPECIES: NlpC/P60 family prote | taxID used:135614 OG02622|WP\_010371422.1 | MULTISPECIES: phosphatase PAP2 fami | taxID used:135614 OG02623|WP\_010370124.1 | MULTISPECIES: uracil-DNA glycosylas | taxID used:135614 OG02624|WP\_010371550.1 | MULTISPECIES: septum site-determini | taxID used:135614 OG02625|WP\_005993372.1 | MULTISPECIES: 30S ribosomal protein | taxID used:135614 OG02626|WP\_039433051.1 | MULTISPECIES: KR domain-containing | taxID used:135614 OG02627|WP\_054393944.1 | MULTISPECIES: hypothetical protein | taxID used:135614 OG02628|WP\_010366472.1 | MULTISPECIES: 23S rRNA (guanosine(2 | taxID used:135614 OG02629|WP\_010372330.1 | hypothetical protein [Xanthomonas v | taxID used:135614 OG00262|WP\_010364401.1 | MULTISPECIES: biopolymer transporte | taxID used:135614 OG02630|WP\_010371254.1 | MULTISPECIES: SDR family NAD(P)-dep | taxID used:135614 OG02631|WP\_082345111.1 | DUF839 domain-containing protein [X | taxID used:135614 OG02632|WP\_010366586.1 | MULTISPECIES: cell division protein | taxID used:135614 OG02633|WP\_010368309.1 | MULTISPECIES: response regulator tr | taxID used:135614 OG02634|WP\_010365002.1 | MULTISPECIES: flagellar motor prote | taxID used:135614 OG02636|WP\_010368405.1 | 3-oxoacyl-ACP reductase FabG, parti | taxID used:135614 OG02637|WP\_052259139.1 | hypothetical protein [Xanthomonas v | taxID used:135614 OG02638|WP\_010373661.1 | MULTISPECIES: SDR family NAD(P)-dep | taxID used:135614 OG02639|WP\_010367757.1 | MULTISPECIES: GntR family transcrip | taxID used:135614 OG00263|WP\_054393949.1 | MULTISPECIES: S9 family peptidase [ | taxID used:135614 OG02640|WP\_010364914.1 | MULTISPECIES: ABC transporter perme | taxID used:135614 OG02641|WP\_010366605.1 | hypothetical protein, partial [Xant | taxID used:135614 OG02642|WP\_010368205.1 | MULTISPECIES: KR domain-containing | taxID used:135614 OG02643|WP\_039431392.1 | TIGR03759 family integrating conjug | taxID used:135614 OG02644|WP\_010369767.1 | MULTISPECIES: 3-oxoacyl-ACP reducta | taxID used:135614 OG02645|WP\_010366145.1 | MULTISPECIES: ribonuclease HII [Xan | taxID used:135614 OG02646|WP\_010371630.1 | MULTISPECIES: AraC family transcrip | taxID used:135614 OG02647|WP\_010373375.1 | hypothetical protein [Xanthomonas v | taxID used:135614 OG02648|WP\_017115142.1 | MULTISPECIES: DUF4339 domain-contai | taxID used:135614 OG02649|WP\_003489074.1 | MULTISPECIES: glycosyltransferase f | taxID used:135614 OG00264|WP\_017117328.1 | methyl-accepting chemotaxis protein | taxID used:135614 OG02650|WP\_010374583.1 | orotidine-5'-phosphate decarboxylas | taxID used:135614 OG02651|WP\_010368278.1 | MULTISPECIES: hypothetical protein | taxID used:135614 OG02652|WP\_010368502.1 | MULTISPECIES: DNA polymerase III su | taxID used:135614 OG02653|WP\_010365633.1 | MULTISPECIES: electron transfer fla | taxID used:135614 OG02654|WP\_010366320.1 | MULTISPECIES: SDR family NAD(P)-dep | taxID used:135614 OG02655|WP\_039435793.1 | hypothetical protein [Xanthomonas v | taxID used:135614 OG02656|WP\_017116144.1 | MULTISPECIES: hypothetical protein | taxID used:135614 OG02657|WP\_010370726.1 | MULTISPECIES: ATP-binding cassette | taxID used:135614 OG02658|WP\_010366789.1 | MULTISPECIES: SIMPL domain-containi | taxID used:135614 OG02659|WP\_033009660.1 | MULTISPECIES: hypothetical protein | taxID used:135614 OG00265|WP\_010368389.1 | methyl-accepting chemotaxis protein | taxID used:135614 OG02660|WP\_010367163.1 | MULTISPECIES: 1-(5-phosphoribosyl)- | taxID used:135614 OG02661|WP\_026112136.1 | hypothetical protein [Xanthomonas v | taxID used:135614 OG02662|WP\_017113101.1 | MULTISPECIES: copper homeostasis pr | taxID used:135614 OG02663|WP\_007965956.1 | MULTISPECIES: AraC family transcrip | taxID used:135614 OG02664|WP\_010368667.1 | MULTISPECIES: 16S rRNA (uracil(1498 | taxID used:135614 OG02665|WP\_039443891.1 | hypothetical protein [Xanthomonas v | taxID used:135614 OG02666|WP\_017115690.1 | MULTISPECIES: 6-phosphogluconolacto | taxID used:135614 OG02667|WP\_010366898.1 | MULTISPECIES: 23S rRNA pseudouridin | taxID used:135614 OG02668|WP\_017121642.1 | MULTISPECIES: type IV secretory pat | taxID used:135614 OG02669|WP\_010364576.1 | MULTISPECIES: M23 family metallopep | taxID used:135614 OG00266|WP\_010365847.1 | MULTISPECIES: hypothetical protein | taxID used:135614 OG02670|WP\_039440913.1 | hypothetical protein [Xanthomonas v | taxID used:135614 OG02671|WP\_010366453.1 | transporter, partial [Xanthomonas v | taxID used:135614 OG02672|WP\_010365620.1 | MULTISPECIES: CoA transferase subun | taxID used:135614 OG02673|WP\_005922326.1 | hypothetical protein [Xanthomonas v | taxID used:135614 OG02674|WP\_010367117.1 | MULTISPECIES: MOSC domain-containin | taxID used:135614 OG02675|WP\_010373322.1 | MULTISPECIES: lipoprotein-releasing | taxID used:135614 OG02676|WP\_010363965.1 | MULTISPECIES: YebC/PmpR family DNA- | taxID used:135614 OG02677|WP\_008576608.1 | MULTISPECIES: GntR family transcrip | taxID used:135614 OG02678|WP\_088124071.1 | MULTISPECIES: YitT family protein [ | taxID used:135614 OG02679|WP\_010372777.1 | MULTISPECIES: bifunctional 2-polypr | taxID used:135614 OG00267|WP\_010363589.1 | MULTISPECIES: phenylalanine--tRNA l | taxID used:135614 OG02680|WP\_084818994.1 | MULTISPECIES: DUF998 domain-contain | taxID used:135614 OG02681|WP\_017117707.1 | MULTISPECIES: hypothetical protein | taxID used:135614 OG02682|WP\_010369955.1 | MULTISPECIES: DUF1629 domain-contai | taxID used:135614 OG02683|WP\_010363017.1 | P-type DNA transfer protein VirB5 [ | taxID used:135614 OG02684|WP\_010374666.1 | MULTISPECIES: SURF1 family protein | taxID used:135614 OG02685|WP\_010366308.1 | MULTISPECIES: cell envelope biogene | taxID used:135614 OG02686|WP\_010368589.1 | MULTISPECIES: hypothetical protein | taxID used:135614 OG02687|WP\_010365645.1 | MULTISPECIES: short-chain dehydroge | taxID used:135614 OG02688|WP\_010368066.1 | MULTISPECIES: haloacid dehalogenase | taxID used:135614 OG02689|WP\_017118135.1 | MULTISPECIES: hypothetical protein | taxID used:135614 OG00268|WP\_026112782.1 | MULTISPECIES: DUF1631 domain-contai | taxID used:135614 OG02690|WP\_010364013.1 | site-specific DNA-methyltransferase | taxID used:135614 OG02691|WP\_010364507.1 | MULTISPECIES: ribonuclease PH [Xant | taxID used:135614 OG02692|WP\_017115105.1 | hypothetical protein [Xanthomonas v | taxID used:135614 OG02693|WP\_017118334.1 | hypothetical protein [Xanthomonas v | taxID used:135614 OG02694|WP\_010365972.1 | MULTISPECIES: DNA repair protein Re | taxID used:135614 OG02695|WP\_010374897.1 | MULTISPECIES: type III pantothenate | taxID used:135614 OG02696|WP\_010373404.1 | serine/threonine-protein phosphatas | taxID used:135614 OG02697|WP\_017113437.1 | MULTISPECIES: glutathione S-transfe | taxID used:135614 OG02698|WP\_010366166.1 | MULTISPECIES: UMP kinase [Xanthomon | taxID used:135614 OG02699|WP\_010366501.1 | MULTISPECIES: endonuclease III [Xan | taxID used:135614 OG00269|WP\_026112693.1 | MULTISPECIES: sensor domain-contain | taxID used:135614 OG00026|WP\_039437571.1 | DUF1983 domain-containing protein [ | taxID used:135614 OG02700|WP\_010364022.1 | terminase endonuclease subunit, par | taxID used:135614 OG02701|WP\_010379111.1 | MULTISPECIES: response regulator tr | taxID used:135614 OG02702|WP\_017118050.1 | hypothetical protein [Xanthomonas v | taxID used:135614 OG02703|WP\_010366481.1 | MULTISPECIES: phosphate transport s | taxID used:135614 OG02704|WP\_010365975.1 | MULTISPECIES: hybrid sensor histidi | taxID used:135614 OG02705|WP\_010370980.1 | FAA hydrolase family protein, parti | taxID used:135614 OG02706|WP\_026112491.1 | MULTISPECIES: DUF1349 domain-contai | taxID used:135614 OG02707|WP\_010372714.1 | MULTISPECIES: glutathione S-transfe | taxID used:135614 OG02708|WP\_010371966.1 | MULTISPECIES: ABC transporter ATP-b | taxID used:135614 OG02709|WP\_010367435.1 | MULTISPECIES: hypothetical protein | taxID used:135614 OG00270|WP\_010367412.1 | HAMP domain-containing protein [Xan | taxID used:135614 OG02710|WP\_017115332.1 | MULTISPECIES: molecular chaperone [ | taxID used:135614 OG02711|WP\_010366739.1 | MULTISPECIES: rhomboid family intra | taxID used:135614 OG02712|WP\_010369674.1 | MULTISPECIES: phosphatase [Xanthomo | taxID used:135614 OG02713|WP\_010365725.1 | MULTISPECIES: pirin family protein | taxID used:135614 OG02714|WP\_010366597.1 | MULTISPECIES: DUF3011 domain-contai | taxID used:135614 OG02715|WP\_010369829.1 | DUF3800 domain-containing protein [ | taxID used:135614 OG02716|WP\_010368200.1 | MULTISPECIES: type II secretion sys | taxID used:135614 OG02718|WP\_017115727.1 | MULTISPECIES: DUF2461 domain-contai | taxID used:135614 OG02719|WP\_010363648.1 | MULTISPECIES: class I SAM-dependent | taxID used:135614 OG00271|WP\_010367710.1 | MULTISPECIES: TonB-dependent recept | taxID used:135614 OG02720|WP\_010369254.1 | MULTISPECIES: nucleotidyltransferas | taxID used:135614 OG02721|WP\_010367669.1 | MULTISPECIES: response regulator tr | taxID used:135614 OG02722|WP\_010367485.1 | MULTISPECIES: hypothetical protein | taxID used:135614 OG02723|WP\_010371432.1 | MULTISPECIES: aspartate/glutamate r | taxID used:135614 OG02724|WP\_017112664.1 | MULTISPECIES: transposase [Xanthomo | taxID used:135614 OG02725|WP\_008572666.1 | MULTISPECIES: LPS export ABC transp | taxID used:135614 OG02726|WP\_010364133.1 | MULTISPECIES: RNA-binding protein S | taxID used:135614 OG02727|WP\_010379721.1 | MULTISPECIES: ATP-binding cassette | taxID used:135614 OG02728|WP\_010367152.1 | MULTISPECIES: acireductone synthase | taxID used:135614 OG02729|WP\_010369469.1 | deoxyribonuclease V, partial [Xanth | taxID used:135614 OG00272|WP\_010366175.1 | fimbrial biogenesis outer membrane | taxID used:135614 OG02730|WP\_010369086.1 | MULTISPECIES: SDR family NAD(P)-dep | taxID used:135614 OG02731|WP\_076605000.1 | MULTISPECIES: P-type DNA transfer p | taxID used:135614 OG02732|WP\_010369892.1 | MULTISPECIES: peptidase [Xanthomona | taxID used:135614 OG02733|WP\_080762857.1 | MULTISPECIES: hypothetical protein | taxID used:135614 OG02734|WP\_010367114.1 | MULTISPECIES: 4-hydroxy-tetrahydrod | taxID used:135614 OG02735|WP\_010373387.1 | DNA-binding response regulator, par | taxID used:135614 OG02736|WP\_010368498.1 | MULTISPECIES: serine/threonine-prot | taxID used:135614 OG02737|WP\_010372086.1 | MULTISPECIES: rRNA pseudouridine sy | taxID used:135614 OG02738|WP\_010372785.1 | phytoene/squalene synthase family p | taxID used:135614 OG02739|WP\_010367580.1 | MULTISPECIES: hypothetical protein | taxID used:135614 OG00273|WP\_010373566.1 | MULTISPECIES: DNA translocase FtsK | taxID used:135614 OG02740|WP\_010371677.1 | MULTISPECIES: hypothetical protein | taxID used:135614 OG02741|WP\_010369897.1 | MULTISPECIES: thiol:disulfide oxido | taxID used:135614 OG02742|WP\_010366213.1 | MULTISPECIES: thiopurine S-methyltr | taxID used:135614 OG02743|WP\_005914015.1 | polysaccharide biosynthesis protein | taxID used:135614 OG02744|WP\_033009707.1 | MULTISPECIES: hypothetical protein | taxID used:135614 OG02745|WP\_010367924.1 | MULTISPECIES: HrpE/YscL family type | taxID used:135614 OG02746|WP\_010367411.1 | MULTISPECIES: response regulator tr | taxID used:135614 OG02747|WP\_010368230.1 | MULTISPECIES: lipoyl(octanoyl) tran | taxID used:135614 OG02749|WP\_017170383.1 | MULTISPECIES: HNH endonuclease [Xan | taxID used:135614 OG00274|WP\_026112854.1 | MULTISPECIES: TonB-dependent sidero | taxID used:135614 OG02751|WP\_010365433.1 | MULTISPECIES: glycosyl transferase | taxID used:135614 OG02752|WP\_010368273.1 | MULTISPECIES: class I SAM-dependent | taxID used:135614 OG02753|WP\_010369060.1 | MULTISPECIES: epoxyqueuosine reduct | taxID used:135614 OG02754|WP\_017118732.1 | MULTISPECIES: hypothetical protein | taxID used:135614 OG02755|WP\_010364199.1 | tRNA (adenosine(37)-N6)-threonylcar | taxID used:135614 OG02756|WP\_017117402.1 | MULTISPECIES: HAD family hydrolase | taxID used:135614 OG02757|WP\_010363998.1 | MULTISPECIES: 7-carboxy-7-deazaguan | taxID used:135614 OG02758|WP\_123180887.1 | MULTISPECIES: DNA-binding protein [ | taxID used:135614 OG02759|WP\_017118422.1 | hypothetical protein [Xanthomonas v | taxID used:135614 OG00275|WP\_010373251.1 | RNA-binding transcriptional accesso | taxID used:135614 OG02760|WP\_017113759.1 | 2OG-Fe(II) oxygenase superfamily pr | taxID used:135614 OG02761|WP\_010363700.1 | MULTISPECIES: hypothetical protein | taxID used:135614 OG02762|WP\_010366505.1 | MULTISPECIES: peptidylprolyl isomer | taxID used:135614 OG02763|WP\_002806631.1 | MULTISPECIES: phosphate regulon tra | taxID used:135614 OG02764|WP\_026112198.1 | heteromeric transposase endonucleas | taxID used:135614 OG02765|WP\_010365500.1 | MULTISPECIES: response regulator tr | taxID used:135614 OG02766|WP\_010364271.1 | MULTISPECIES: DUF4194 domain-contai | taxID used:135614 OG02767|WP\_010372780.1 | MULTISPECIES: phosphoglycolate phos | taxID used:135614 OG02768|WP\_017113330.1 | MULTISPECIES: DUF2491 domain-contai | taxID used:135614 OG02769|WP\_010369321.1 | MULTISPECIES: YggS family pyridoxal | taxID used:135614 OG00276|WP\_026112563.1 | MULTISPECIES: bifunctional diguanyl | taxID used:135614 OG02770|WP\_010366497.1 | carbonic anhydrase, partial [Xantho | taxID used:135614 OG02771|WP\_010368339.1 | MULTISPECIES: hypothetical protein | taxID used:135614 OG02772|WP\_010365058.1 | MULTISPECIES: hypothetical protein | taxID used:135614 OG02773|WP\_010371719.1 | biotin-independent malonate decarbo | taxID used:135614 OG02774|WP\_039439086.1 | hypothetical protein [Xanthomonas v | taxID used:135614 OG02775|WP\_010370078.1 | MULTISPECIES: hypothetical protein | taxID used:135614 OG02776|WP\_010371823.1 | IS3 family transposase [Xanthomonas | taxID used:135614 OG02777|WP\_052259138.1 | hypothetical protein [Xanthomonas v | taxID used:135614 OG02778|WP\_010372034.1 | MULTISPECIES: membrane protein [Xan | taxID used:135614 OG02779|WP\_017115557.1 | MULTISPECIES: D-alanyl-D-alanine di | taxID used:135614 OG00277|WP\_010364849.1 | MULTISPECIES: TonB-dependent recept | taxID used:135614 OG02780|WP\_010374850.1 | MULTISPECIES: ABC transporter ATP-b | taxID used:135614 OG02781|WP\_080765175.1 | DUF2290 domain-containing protein [ | taxID used:135614 OG02782|WP\_010369281.1 | MULTISPECIES: Fe2+-dependent dioxyg | taxID used:135614 OG02783|WP\_010369234.1 | MULTISPECIES: DUF3108 domain-contai | taxID used:135614 OG02784|WP\_010368067.1 | MULTISPECIES: cAMP-activated global | taxID used:135614 OG02785|WP\_010364746.1 | MULTISPECIES: rhomboid family intra | taxID used:135614 OG02786|WP\_017113468.1 | MULTISPECIES: NYN domain-containing | taxID used:135614 OG02787|WP\_017117317.1 | hypothetical protein, partial [Xant | taxID used:135614 OG02788|WP\_010364596.1 | MULTISPECIES: molybdate ABC transpo | taxID used:135614 OG02789|WP\_039439219.1 | helix-turn-helix transcriptional re | taxID used:135614 OG00278|WP\_010374736.1 | MULTISPECIES: phosphomannomutase/ph | taxID used:135614 OG02790|WP\_010369713.1 | MULTISPECIES: ABC transporter ATP-b | taxID used:135614 OG02791|WP\_010366652.1 | MULTISPECIES: methylamine utilizati | taxID used:135614 OG02792|WP\_010374716.1 | MULTISPECIES: hypothetical protein | taxID used:135614 OG02793|WP\_010369247.1 | MULTISPECIES: DnaA regulatory inact | taxID used:135614 OG02794|WP\_039439688.1 | MULTISPECIES: hypothetical protein | taxID used:135614 OG02795|WP\_010363063.1 | MULTISPECIES: chromosome partitioni | taxID used:135614 OG02796|WP\_010367280.1 | MULTISPECIES: 50S ribosomal protein | taxID used:135614 OG02797|WP\_010373617.1 | MULTISPECIES: flagellar basal body | taxID used:135614 OG02798|WP\_010372057.1 | MULTISPECIES: ABC transporter perme | taxID used:135614 OG02799|WP\_039436454.1 | MULTISPECIES: two-component system | taxID used:135614 OG00279|WP\_017118300.1 | MULTISPECIES: xanthine dehydrogenas | taxID used:135614 OG00027|WP\_017115405.1 | MULTISPECIES: non-ribosomal peptide | taxID used:135614 OG02800|WP\_010363668.1 | MULTISPECIES: sugar O-acyltransfera | taxID used:135614 OG02801|WP\_017115728.1 | MULTISPECIES: carbonate dehydratase | taxID used:135614 OG02802|WP\_039435738.1 | MULTISPECIES: DsbA family oxidoredu | taxID used:135614 OG02803|WP\_080765187.1 | PDZ domain-containing protein [Xant | taxID used:135614 OG02804|WP\_010369588.1 | MULTISPECIES: hypothetical protein | taxID used:135614 OG02806|WP\_017115630.1 | MULTISPECIES: hypothetical protein | taxID used:135614 OG02807|WP\_010365426.1 | MULTISPECIES: hypothetical protein | taxID used:135614 OG02808|WP\_026112932.1 | MULTISPECIES: type I-C CRISPR-assoc | taxID used:135614 OG02809|WP\_017112818.1 | MULTISPECIES: response regulator tr | taxID used:135614 OG00280|WP\_033012264.1 | MULTISPECIES: TonB-dependent recept | taxID used:135614 OG02810|WP\_033836774.1 | MULTISPECIES: VWA domain-containing | taxID used:135614 OG02811|WP\_038897333.1 | hypothetical protein [Xanthomonas v | taxID used:135614 OG02812|WP\_017113985.1 | MULTISPECIES: hemolysin III [Xantho | taxID used:135614 OG02813|WP\_010363483.1 | MULTISPECIES: methyltransferase dom | taxID used:135614 OG02814|WP\_010366880.1 | MULTISPECIES: response regulator tr | taxID used:135614 OG02815|WP\_010369649.1 | MULTISPECIES: response regulator tr | taxID used:135614 OG02816|WP\_039441461.1 | MULTISPECIES: energy transducer Ton | taxID used:135614 OG02817|WP\_010368312.1 | hypothetical protein, partial [Xant | taxID used:135614 OG02818|WP\_039441060.1 | uracil-DNA glycosylase [Xanthomonas | taxID used:135614 OG02819|WP\_002808458.1 | MULTISPECIES: response regulator tr | taxID used:135614 OG00281|WP\_010368151.1 | MULTISPECIES: cytochrome c biogenes | taxID used:135614 OG02820|WP\_010371528.1 | MULTISPECIES: DNA-3-methyladenine g | taxID used:135614 OG02821|WP\_039431377.1 | MULTISPECIES: DUF3275 domain-contai | taxID used:135614 OG02822|WP\_017116871.1 | MULTISPECIES: nuclease-related prot | taxID used:135614 OG02823|WP\_010372084.1 | MULTISPECIES: HAD family phosphatas | taxID used:135614 OG02824|WP\_010371912.1 | MULTISPECIES: dethiobiotin synthase | taxID used:135614 OG02825|WP\_026113273.1 | MULTISPECIES: aldehyde dehydrogenas | taxID used:135614 OG02826|WP\_010376017.1 | MULTISPECIES: DUF484 domain-contain | taxID used:135614 OG02827|WP\_010380413.1 | MULTISPECIES: DUF502 domain-contain | taxID used:135614 OG02828|WP\_010365969.1 | MULTISPECIES: ribonuclease III [Xan | taxID used:135614 OG02829|WP\_010365512.1 | MULTISPECIES: type 1 glutamine amid | taxID used:135614 OG00282|WP\_010364287.1 | MULTISPECIES: FUSC family protein [ | taxID used:135614 OG02830|WP\_039436278.1 | hypothetical protein [Xanthomonas v | taxID used:135614 OG02831|WP\_082347509.1 | hypothetical protein [Xanthomonas v | taxID used:135614 OG02832|WP\_039436278.1 | hypothetical protein [Xanthomonas v | taxID used:135614 OG02833|WP\_017122787.1 | MULTISPECIES: hypothetical protein | taxID used:135614 OG02834|WP\_017116218.1 | MULTISPECIES: energy transducer Ton | taxID used:135614 OG02835|WP\_010370134.1 | MULTISPECIES: cell division ATP-bin | taxID used:135614 OG02836|WP\_017112868.1 | MULTISPECIES: outer membrane lipopr | taxID used:135614 OG02837|WP\_010368049.1 | MULTISPECIES: ribulose-phosphate 3- | taxID used:135614 OG02838|WP\_039435454.1 | hypothetical protein [Xanthomonas v | taxID used:135614 OG02839|WP\_017115508.1 | DUF3800 domain-containing protein [ | taxID used:135614 OG00283|WP\_054393965.1 | MULTISPECIES: membrane protein [Xan | taxID used:135614 OG02840|WP\_010369238.1 | MULTISPECIES: phosphoribosylglycina | taxID used:135614 OG02841|WP\_010371896.1 | MULTISPECIES: maleylacetoacetate is | taxID used:135614 OG02842|WP\_017116757.1 | MULTISPECIES: alpha/beta fold hydro | taxID used:135614 OG02843|WP\_010365525.1 | methyltransferase domain-containing | taxID used:135614 OG02844|WP\_017115278.1 | MULTISPECIES: protein-methionine-su | taxID used:135614 OG02845|WP\_010370426.1 | MULTISPECIES: alpha/beta hydrolase | taxID used:135614 OG02846|WP\_010363254.1 | MULTISPECIES: hypothetical protein | taxID used:135614 OG02847|WP\_010366669.1 | MULTISPECIES: N-(5'-phosphoribosyl) | taxID used:135614 OG02848|WP\_017112196.1 | MULTISPECIES: HNH endonuclease [Xan | taxID used:135614 OG02849|WP\_003490678.1 | MULTISPECIES: response regulator tr | taxID used:135614 OG00284|WP\_010365478.1 | NADP-dependent malic enzyme, partia | taxID used:135614 OG02850|WP\_087911196.1 | DUF1629 domain-containing protein [ | taxID used:135614 OG02851|WP\_039442531.1 | MULTISPECIES: NYN domain-containing | taxID used:135614 OG02852|WP\_010381948.1 | MULTISPECIES: JAB domain-containing | taxID used:135614 OG02853|WP\_010371043.1 | secreted signal peptide protein [Xa | taxID used:135614 OG02854|WP\_026112444.1 | TetR/AcrR family transcriptional re | taxID used:135614 OG02855|WP\_010365684.1 | MULTISPECIES: protein-L-isoaspartat | taxID used:135614 OG02856|WP\_116896646.1 | glycosyl transferase, partial [Xant | taxID used:135614 OG02857|WP\_010364430.1 | MULTISPECIES: serine/threonine prot | taxID used:135614 OG02858|WP\_010373008.1 | MULTISPECIES: (d)CMP kinase [Xantho | taxID used:135614 OG02859|WP\_010367363.1 | MULTISPECIES: RNA polymerase sigma | taxID used:135614 OG00285|WP\_039440920.1 | MULTISPECIES: type I restriction en | taxID used:135614 OG02860|WP\_010364546.1 | MULTISPECIES: fimbrial protein [Xan | taxID used:135614 OG02861|WP\_010364121.1 | MULTISPECIES: 7-cyano-7-deazaguanin | taxID used:135614 OG02862|WP\_017170356.1 | MULTISPECIES: hypothetical protein | taxID used:135614 OG02863|WP\_010365120.1 | MULTISPECIES: DNA polymerase III su | taxID used:135614 OG02864|WP\_010374890.1 | MULTISPECIES: dTMP kinase [Xanthomo | taxID used:135614 OG02865|WP\_010372906.1 | MULTISPECIES: heme exporter protein | taxID used:135614 OG02866|WP\_010364371.1 | MULTISPECIES: TetR/AcrR family tran | taxID used:135614 OG02867|WP\_010363630.1 | MULTISPECIES: exopolysaccharide bio | taxID used:135614 OG02868|WP\_010370261.1 | hypothetical protein [Xanthomonas v | taxID used:135614 OG02869|WP\_010373406.1 | MULTISPECIES: type VI secretion sys | taxID used:135614 OG00286|WP\_054394001.1 | MULTISPECIES: CRISPR-associated end | taxID used:135614 OG02870|WP\_010367374.1 | MULTISPECIES: NUDIX hydrolase [Xant | taxID used:135614 OG02871|WP\_010365355.1 | MULTISPECIES: DUF2894 domain-contai | taxID used:135614 OG02872|WP\_010367403.1 | MULTISPECIES: peptide-methionine (S | taxID used:135614 OG02873|WP\_010370104.1 | MULTISPECIES: glutathione S-transfe | taxID used:135614 OG02874|WP\_010369035.1 | MULTISPECIES: hypothetical protein | taxID used:135614 OG02875|WP\_010374372.1 | MULTISPECIES: response regulator tr | taxID used:135614 OG02876|WP\_010366714.1 | MULTISPECIES: dienelactone hydrolas | taxID used:135614 OG02877|WP\_026112296.1 | MULTISPECIES: class I SAM-dependent | taxID used:135614 OG02878|WP\_080764497.1 | hypothetical protein [Xanthomonas v | taxID used:135614 OG02879|WP\_017121390.1 | MULTISPECIES: DUF1275 domain-contai | taxID used:135614 OG00287|WP\_010370183.1 | hypothetical protein, partial [Xant | taxID used:135614 OG02880|WP\_010365357.1 | MULTISPECIES: membrane protein [Xan | taxID used:135614 OG02881|WP\_010369846.1 | MULTISPECIES: hypothetical protein | taxID used:135614 OG02882|WP\_010374767.1 | MULTISPECIES: kinase [Xanthomonas] | taxID used:135614 OG02883|WP\_010369972.1 | hypothetical protein [Xanthomonas v | taxID used:135614 OG02884|WP\_010368507.1 | MULTISPECIES: hypothetical protein | taxID used:135614 OG02885|WP\_010370772.1 | MULTISPECIES: hypothetical protein | taxID used:135614 OG02886|WP\_010363741.1 | MULTISPECIES: hypothetical protein | taxID used:135614 OG02887|WP\_017112139.1 | MULTISPECIES: NADH-quinone oxidored | taxID used:135614 OG02888|WP\_011038242.1 | MULTISPECIES: hypothetical protein | taxID used:135614 OG02889|WP\_080764530.1 | DUF1629 domain-containing protein [ | taxID used:135614 OG00288|WP\_010367762.1 | type II secretion system protein Gs | taxID used:135614 OG02890|WP\_086020499.1 | transcriptional regulator [Xanthomo | taxID used:135614 OG02891|WP\_010364369.1 | protein-L-isoaspartate O-methyltran | taxID used:135614 OG02892|WP\_010368073.1 | MULTISPECIES: 2-polyprenyl-3-methyl | taxID used:135614 OG02893|WP\_010373200.1 | MULTISPECIES: hydrolase [Xanthomona | taxID used:135614 OG02894|WP\_010373663.1 | MULTISPECIES: acetyltransferase [Xa | taxID used:135614 OG02895|WP\_010364381.1 | MULTISPECIES: 3-isopropylmalate deh | taxID used:135614 OG02896|WP\_087910981.1 | conjugal transfer protein TrbP, par | taxID used:135614 OG02897|WP\_010364628.1 | MULTISPECIES: DUF938 domain-contain | taxID used:135614 OG02898|WP\_010363104.1 | MULTISPECIES: translesion DNA synth | taxID used:135614 OG02899|WP\_100243546.1 | avirulence protein AvrXv3 [Xanthomo | taxID used:135614 OG00289|WP\_010368087.1 | hybrid sensor histidine kinase/resp | taxID used:135614 OG00028|WP\_010364265.1 | MULTISPECIES: ATP-binding protein [ | taxID used:135614 OG02900|WP\_010367148.1 | MULTISPECIES: methylthioribulose 1- | taxID used:135614 OG02901|WP\_010367315.1 | MULTISPECIES: lipoprotein localizat | taxID used:135614 OG02902|WP\_082345095.1 | hypothetical protein [Xanthomonas v | taxID used:135614 OG02903|WP\_010378718.1 | MULTISPECIES: conjugal transfer pro | taxID used:135614 OG02904|WP\_010364909.1 | MULTISPECIES: organic solvent ABC t | taxID used:135614 OG02905|WP\_017112380.1 | MULTISPECIES: hypothetical protein | taxID used:135614 OG02906|WP\_017113228.1 | MULTISPECIES: glutathione S-transfe | taxID used:135614 OG02907|WP\_010365583.1 | MULTISPECIES: thiol:disulfide inter | taxID used:135614 OG02908|WP\_010373608.1 | MULTISPECIES: flagellar basal body | taxID used:135614 OG02909|WP\_010366692.1 | MULTISPECIES: SCO family protein [X | taxID used:135614 OG00290|WP\_010369901.1 | MULTISPECIES: S9 family peptidase [ | taxID used:135614 OG02910|WP\_026112892.1 | ubiquinol-cytochrome c reductase ir | taxID used:135614 OG02911|WP\_010374589.1 | DUF480 domain-containing protein [X | taxID used:135614 OG02912|WP\_010365382.1 | MULTISPECIES: hypothetical protein | taxID used:135614 OG02913|WP\_033836512.1 | MULTISPECIES: hypothetical protein | taxID used:135614 OG02914|WP\_010373327.1 | MULTISPECIES: MotA/TolQ/ExbB proton | taxID used:135614 OG02915|WP\_010368757.1 | MULTISPECIES: polyisoprenoid-bindin | taxID used:135614 OG02916|WP\_080763713.1 | ATP-binding protein [Xanthomonas va | taxID used:135614 OG02917|WP\_010366273.1 | MULTISPECIES: glutathione S-transfe | taxID used:135614 OG02918|WP\_017115600.1 | MULTISPECIES: glutathione S-transfe | taxID used:135614 OG02919|WP\_010371919.1 | MULTISPECIES: phosphoglycolate phos | taxID used:135614 OG00291|WP\_039431387.1 | DEAD/DEAH box helicase [Xanthomonas | taxID used:135614 OG02920|WP\_017113604.1 | MULTISPECIES: hypothetical protein | taxID used:135614 OG02921|WP\_010366476.1 | MULTISPECIES: ribonuclease T [Xanth | taxID used:135614 OG02922|WP\_010369276.1 | MULTISPECIES: membrane protein [Xan | taxID used:135614 OG02923|WP\_026112442.1 | carboxypeptidase [Xanthomonas vasic | taxID used:135614 OG02924|WP\_017113413.1 | MULTISPECIES: hypothetical protein | taxID used:135614 OG02925|WP\_010364537.1 | MULTISPECIES: hypothetical protein | taxID used:135614 OG02926|WP\_017115270.1 | peptidoglycan-binding protein [Xant | taxID used:135614 OG02927|WP\_010368729.1 | MULTISPECIES: hemolysin III family | taxID used:135614 OG02928|WP\_010368878.1 | muramidase [Xanthomonas vasicola] [ | taxID used:135614 OG02929|WP\_010371717.1 | malonate decarboxylase holo-[acyl-c | taxID used:135614 OG00292|WP\_116896643.1 | type IV secretion system protein Do | taxID used:135614 OG02930|WP\_010368847.1 | hypothetical protein, partial [Xant | taxID used:135614 OG02931|WP\_010366564.1 | MULTISPECIES: FMN-binding negative | taxID used:135614 OG02932|WP\_038894643.1 | hypothetical protein [Xanthomonas v | taxID used:135614 OG02933|WP\_010370632.1 | MULTISPECIES: superoxide dismutase | taxID used:135614 OG02934|WP\_010367766.1 | MULTISPECIES: general secretion pat | taxID used:135614 OG02935|WP\_010379506.1 | hypothetical protein [Xanthomonas v | taxID used:135614 OG02936|WP\_010371052.1 | MULTISPECIES: 50S ribosomal protein | taxID used:135614 OG02937|WP\_010367481.1 | MULTISPECIES: LemA family protein [ | taxID used:135614 OG02938|WP\_038893805.1 | hypothetical protein [Xanthomonas v | taxID used:135614 OG02939|WP\_010365995.1 | MULTISPECIES: ligase-associated DNA | taxID used:135614 OG00293|WP\_010367961.1 | MULTISPECIES: 1,4-alpha-glucan bran | taxID used:135614 OG02940|WP\_010365521.1 | MULTISPECIES: SGNH/GDSL hydrolase f | taxID used:135614 OG02941|WP\_010370518.1 | MULTISPECIES: lysophosphatidylcholi | taxID used:135614 OG02942|WP\_017116342.1 | MULTISPECIES: M23 family metallopep | taxID used:135614 OG02943|WP\_010364732.1 | MULTISPECIES: hypothetical protein | taxID used:135614 OG02944|WP\_010370864.1 | MULTISPECIES: haloacid dehalogenase | taxID used:135614 OG02945|WP\_017115691.1 | MULTISPECIES: bifunctional 4-hydrox | taxID used:135614 OG02946|WP\_017116541.1 | gamma-glutamyltranspeptidase [Xanth | taxID used:135614 OG02947|WP\_010371359.1 | peptidoglycan-binding protein [Xant | taxID used:135614 OG02948|WP\_010374438.1 | MULTISPECIES: hypothetical protein | taxID used:135614 OG02949|WP\_010364275.1 | MULTISPECIES: GTP cyclohydrolase I | taxID used:135614 OG00294|WP\_010365923.1 | MULTISPECIES: catalase/peroxidase H | taxID used:135614 OG02950|WP\_104607792.1 | glycoside hydrolase family 19 prote | taxID used:135614 OG02951|WP\_010365606.1 | MULTISPECIES: ABC transporter [Xant | taxID used:135614 OG02952|WP\_054393896.1 | MULTISPECIES: ribose-5-phosphate is | taxID used:135614 OG02953|WP\_010367506.1 | MULTISPECIES: arylesterase [Xanthom | taxID used:135614 OG02954|WP\_010364032.1 | MULTISPECIES: glycoside hydrolase f | taxID used:135614 OG02955|WP\_010367908.1 | type III secretion protein [Xanthom | taxID used:135614 OG02956|WP\_054393958.1 | MULTISPECIES: type III secretion sy | taxID used:135614 OG02957|WP\_039431390.1 | membrane protein [Xanthomonas vasic | taxID used:135614 OG02958|WP\_010374714.1 | MULTISPECIES: orotate phosphoribosy | taxID used:135614 OG02959|WP\_010367905.1 | MULTISPECIES: EscR/YscR/HrcR family | taxID used:135614 OG00295|WP\_010373572.1 | ATP-dependent Clp protease ATP-bind | taxID used:135614 OG02960|WP\_010369172.1 | resolvase, partial [Xanthomonas vas | taxID used:135614 OG02961|WP\_010370451.1 | MULTISPECIES: DUF4198 domain-contai | taxID used:135614 OG02962|WP\_010373338.1 | MULTISPECIES: CDP-diacylglycerol--g | taxID used:135614 OG02963|WP\_017113212.1 | tetratricopeptide repeat protein [X | taxID used:135614 OG02964|WP\_010369757.1 | hypothetical protein, partial [Xant | taxID used:135614 OG02965|WP\_010372410.1 | CRISPR-associated protein Cas4, par | taxID used:135614 OG02966|WP\_010370763.1 | MULTISPECIES: DUF4375 domain-contai | taxID used:135614 OG02967|WP\_033009881.1 | glutathione S-transferase family pr | taxID used:135614 OG02968|WP\_010368581.1 | MULTISPECIES: histidine phosphatase | taxID used:135614 OG02969|WP\_054393863.1 | hypothetical protein, partial [Xant | taxID used:135614 OG00296|WP\_039439021.1 | HAMP domain-containing protein, par | taxID used:135614 OG02970|WP\_010372025.1 | MULTISPECIES: hypothetical protein | taxID used:135614 OG02971|WP\_010365295.1 | hypothetical protein [Xanthomonas v | taxID used:135614 OG02972|WP\_010368062.1 | MULTISPECIES: flavin reductase fami | taxID used:135614 OG02973|WP\_017112913.1 | MULTISPECIES: HAD family hydrolase | taxID used:135614 OG02974|WP\_010374793.1 | MULTISPECIES: HTH-type transcriptio | taxID used:135614 OG02975|WP\_010372598.1 | MULTISPECIES: stringent starvation | taxID used:135614 OG02976|WP\_010380996.1 | MULTISPECIES: hypothetical protein | taxID used:135614 OG02978|WP\_039433055.1 | hypothetical protein [Xanthomonas v | taxID used:135614 OG02979|WP\_010373667.1 | MULTISPECIES: UDP-3-O-(3-hydroxymyr | taxID used:135614 OG00297|WP\_017115865.1 | MULTISPECIES: methyl-accepting chem | taxID used:135614 OG02980|WP\_010370338.1 | hypothetical protein, partial [Xant | taxID used:135614 OG02982|WP\_010363150.1 | MULTISPECIES: HD domain-containing | taxID used:135614 OG02983|WP\_039430885.1 | hypothetical protein [Xanthomonas v | taxID used:135614 OG02984|WP\_010365694.1 | MULTISPECIES: 23S rRNA (uridine(255 | taxID used:135614 OG02985|WP\_010374599.1 | MULTISPECIES: thymidine kinase [Xan | taxID used:135614 OG02986|WP\_010365618.1 | MULTISPECIES: CoA transferase subun | taxID used:135614 OG02987|WP\_010363800.1 | MULTISPECIES: dephospho-CoA kinase | taxID used:135614 OG02988|WP\_010366825.1 | MULTISPECIES: hypothetical protein | taxID used:135614 OG02989|WP\_010372350.1 | MULTISPECIES: catalase [Xanthomonas | taxID used:135614 OG00298|WP\_039442464.1 | hypothetical protein [Xanthomonas v | taxID used:135614 OG02990|WP\_008573812.1 | MULTISPECIES: cytochrome c biogenes | taxID used:135614 OG02991|WP\_010367528.1 | MULTISPECIES: hypothetical protein | taxID used:135614 OG02992|WP\_005927202.1 | MULTISPECIES: hypothetical protein | taxID used:135614 OG02993|WP\_010367772.1 | MULTISPECIES: general secretion pat | taxID used:135614 OG02994|WP\_123184247.1 | DUF3363 domain-containing protein, | taxID used:135614 OG02995|WP\_010368393.1 | MULTISPECIES: methyl-accepting chem | taxID used:135614 OG02996|WP\_017115699.1 | MULTISPECIES: response regulator tr | taxID used:135614 OG02997|WP\_010367304.1 | MULTISPECIES: 50S ribosomal protein | taxID used:135614 OG02998|WP\_010370504.1 | MULTISPECIES: TetR/AcrR family tran | taxID used:135614 OG02999|WP\_010363034.1 | hypothetical protein [Xanthomonas v | taxID used:135614 OG00299|WP\_039438892.1 | hypothetical protein [Xanthomonas v | taxID used:135614 OG00029|WP\_010367032.1 | LamG domain-containing protein [Xan | taxID used:135614 OG00002|WP\_039437917.1 | hypothetical protein, partial [Xant | taxID used:135614 OG03000|WP\_010373546.1 | MULTISPECIES: TetR/AcrR family tran | taxID used:135614 OG03001|WP\_017170387.1 | MULTISPECIES: hypothetical protein | taxID used:135614 OG03002|WP\_010365714.1 | MULTISPECIES: transcriptional repre | taxID used:135614 OG03003|WP\_010365497.1 | MULTISPECIES: response regulator tr | taxID used:135614 OG03004|WP\_010370691.1 | MULTISPECIES: ParA family protein [ | taxID used:135614 OG03005|WP\_033009934.1 | MULTISPECIES: cytochrome o ubiquino | taxID used:135614 OG03006|WP\_010363158.1 | MULTISPECIES: alpha-ketoglutarate-d | taxID used:135614 OG03007|WP\_003489223.1 | MULTISPECIES: DNA-binding response | taxID used:135614 OG03008|WP\_010371538.1 | MULTISPECIES: response regulator tr | taxID used:135614 OG03009|WP\_010364229.1 | MULTISPECIES: GNAT family N-acetylt | taxID used:135614 OG00300|WP\_010381548.1 | MULTISPECIES: hypothetical protein | taxID used:135614 OG03010|WP\_033006739.1 | MULTISPECIES: glycine cleavage syst | taxID used:135614 OG03011|WP\_033836508.1 | MULTISPECIES: hypothetical protein | taxID used:135614 OG03012|WP\_017116372.1 | TetR/AcrR family transcriptional re | taxID used:135614 OG03013|WP\_003482986.1 | MULTISPECIES: response regulator tr | taxID used:135614 OG03014|WP\_010367920.1 | MULTISPECIES: type III secretion pr | taxID used:135614 OG03015|WP\_010366701.1 | MULTISPECIES: hypothetical protein | taxID used:135614 OG03016|WP\_010371323.1 | hypothetical protein [Xanthomonas v | taxID used:135614 OG03017|WP\_010371403.1 | MULTISPECIES: hypothetical protein | taxID used:135614 OG03018|WP\_017112904.1 | MULTISPECIES: NAD-dependent epimera | taxID used:135614 OG03019|WP\_017116272.1 | hypothetical protein [Xanthomonas v | taxID used:135614 OG00301|WP\_038894058.1 | MULTISPECIES: hypothetical protein | taxID used:135614 OG03020|WP\_010374347.1 | 16S rRNA (guanine(527)-N(7))-methyl | taxID used:135614 OG03021|WP\_010373601.1 | MULTISPECIES: flagellar basal body | taxID used:135614 OG03022|WP\_039435153.1 | hypothetical protein [Xanthomonas v | taxID used:135614 OG03023|WP\_010363624.1 | MULTISPECIES: DUF47 domain-containi | taxID used:135614 OG03024|WP\_010370537.1 | MULTISPECIES: sterol-binding protei | taxID used:135614 OG03025|WP\_039435045.1 | restriction endonuclease BglII [Xan | taxID used:135614 OG03026|WP\_010373536.1 | MULTISPECIES: tetratricopeptide rep | taxID used:135614 OG03027|WP\_017112508.1 | hypothetical protein, partial [Xant | taxID used:135614 OG03028|WP\_007962205.1 | MULTISPECIES: RNA polymerase sigma | taxID used:135614 OG03029|WP\_002806026.1 | MULTISPECIES: ATP-dependent Clp pro | taxID used:135614 OG00302|WP\_010368388.1 | methyl-accepting chemotaxis protein | taxID used:135614 OG03030|WP\_010368949.1 | MULTISPECIES: YdcF family protein [ | taxID used:135614 OG03031|WP\_010368467.1 | MULTISPECIES: TetR/AcrR family tran | taxID used:135614 OG03032|WP\_039440911.1 | hypothetical protein [Xanthomonas v | taxID used:135614 OG03033|WP\_017116408.1 | hypothetical protein, partial [Xant | taxID used:135614 OG03034|WP\_010371121.1 | MULTISPECIES: 30S ribosomal protein | taxID used:135614 OG03035|WP\_010372037.1 | MULTISPECIES: hypothetical protein | taxID used:135614 OG03036|WP\_033483078.1 | MULTISPECIES: hypothetical protein | taxID used:135614 OG03037|WP\_054393919.1 | MULTISPECIES: YihA family ribosome | taxID used:135614 OG03038|WP\_017118172.1 | MULTISPECIES: diguanylate cyclase [ | taxID used:135614 OG03039|WP\_010367703.1 | MULTISPECIES: hypothetical protein | taxID used:135614 OG00303|WP\_050556469.1 | MULTISPECIES: alpha-glucuronidase [ | taxID used:135614 OG03040|WP\_010370572.1 | MULTISPECIES: hypothetical protein | taxID used:135614 OG03041|WP\_015471990.1 | MULTISPECIES: hypothetical protein | taxID used:135614 OG03042|WP\_010365768.1 | MULTISPECIES: 2-dehydro-3-deoxy-6-p | taxID used:135614 OG03043|WP\_010374480.1 | MULTISPECIES: twin-arginine translo | taxID used:135614 OG03044|WP\_010372746.1 | MULTISPECIES: superoxide dismutase | taxID used:135614 OG03045|WP\_010373231.1 | MULTISPECIES: CDP-diacylglycerol--s | taxID used:135614 OG03046|WP\_010372887.1 | MULTISPECIES: DUF1294 domain-contai | taxID used:135614 OG03047|WP\_080990582.1 | MULTISPECIES: hypothetical protein | taxID used:135614 OG03048|WP\_010370659.1 | MULTISPECIES: YbhB/YbcL family Raf | taxID used:135614 OG03049|WP\_010379465.1 | MULTISPECIES: DUF4276 domain-contai | taxID used:135614 OG00304|WP\_010370756.1 | TonB-dependent siderophore receptor | taxID used:135614 OG03050|WP\_010364511.1 | MULTISPECIES: guanylate kinase [Xan | taxID used:135614 OG03051|WP\_010368084.1 | MULTISPECIES: RNA pyrophosphohydrol | taxID used:135614 OG03052|WP\_017115773.1 | hypothetical protein [Xanthomonas v | taxID used:135614 OG03053|WP\_010377214.1 | MULTISPECIES: TetR/AcrR family tran | taxID used:135614 OG03054|WP\_010369548.1 | MULTISPECIES: GNAT family acetyltra | taxID used:135614 OG03055|WP\_010365370.1 | MULTISPECIES: DUF4142 domain-contai | taxID used:135614 OG03056|WP\_010372342.1 | MULTISPECIES: uracil phosphoribosyl | taxID used:135614 OG03057|WP\_039437184.1 | hypothetical protein [Xanthomonas v | taxID used:135614 OG03058|WP\_010365612.1 | MULTISPECIES: alpha-ketoglutarate-d | taxID used:135614 OG03059|WP\_010365686.1 | MULTISPECIES: DedA family protein [ | taxID used:135614 OG00305|WP\_054393984.1 | MULTISPECIES: 1,4-alpha-glucan bran | taxID used:135614 OG03060|WP\_010372373.1 | MULTISPECIES: 16S rRNA (guanine(966 | taxID used:135614 OG03061|WP\_010366057.1 | MULTISPECIES: hypothetical protein | taxID used:135614 OG03062|WP\_097370608.1 | hypothetical protein, partial [Xant | taxID used:135614 OG03063|WP\_010366248.1 | MULTISPECIES: rhomboid family intra | taxID used:135614 OG03064|WP\_039443094.1 | hypothetical protein [Xanthomonas v | taxID used:135614 OG03065|WP\_010374309.1 | MULTISPECIES: chemotaxis protein [X | taxID used:135614 OG03066|WP\_010369029.1 | MULTISPECIES: hypothetical protein | taxID used:135614 OG03067|WP\_010382263.1 | MULTISPECIES: N-acetyltransferase [ | taxID used:135614 OG03068|WP\_010364604.1 | MULTISPECIES: OmpW family protein [ | taxID used:135614 OG03069|WP\_039437061.1 | response regulator transcription fa | taxID used:135614 OG00306|WP\_033009773.1 | methyl-accepting chemotaxis protein | taxID used:135614 OG03070|WP\_017115643.1 | MULTISPECIES: hypothetical protein | taxID used:135614 OG03071|WP\_010364594.1 | MULTISPECIES: molybdenum ABC transp | taxID used:135614 OG03072|WP\_010372615.1 | MULTISPECIES: DNA-3-methyladenine g | taxID used:135614 OG03073|WP\_010368090.1 | MULTISPECIES: DUF4126 domain-contai | taxID used:135614 OG03075|WP\_010371696.1 | MULTISPECIES: haloacid dehalogenase | taxID used:135614 OG03076|WP\_010368185.1 | MULTISPECIES: poly-beta-1,6-N-acety | taxID used:135614 OG03077|WP\_010367327.1 | MULTISPECIES: TlpA family protein d | taxID used:135614 OG03078|WP\_010367158.1 | MULTISPECIES: bifunctional phosphor | taxID used:135614 OG03079|WP\_080764534.1 | hypothetical protein [Xanthomonas v | taxID used:135614 OG00307|WP\_017116638.1 | methyl-accepting chemotaxis protein | taxID used:135614 OG03080|WP\_017118338.1 | MULTISPECIES: potassium-transportin | taxID used:135614 OG03081|WP\_010364459.1 | MULTISPECIES: thiamine phosphate sy | taxID used:135614 OG03082|WP\_010363040.1 | conjugal transfer protein TrbM, par | taxID used:135614 OG03083|WP\_017170360.1 | MULTISPECIES: hypothetical protein | taxID used:135614 OG03084|WP\_017116904.1 | hypothetical protein [Xanthomonas v | taxID used:135614 OG03085|WP\_010373467.1 | MULTISPECIES: response regulator tr | taxID used:135614 OG03086|WP\_010370467.1 | MULTISPECIES: TetR/AcrR family tran | taxID used:135614 OG03087|WP\_010368625.1 | MULTISPECIES: glutathione S-transfe | taxID used:135614 OG03088|WP\_087920697.1 | hypothetical protein, partial [Xant | taxID used:135614 OG03089|WP\_010368710.1 | MULTISPECIES: TetR/AcrR family tran | taxID used:135614 OG00308|WP\_010366217.1 | DNA topoisomerase IV subunit A, par | taxID used:135614 OG03090|WP\_010373184.1 | MULTISPECIES: hypothetical protein | taxID used:135614 OG03091|WP\_010371547.1 | MULTISPECIES: GNAT family N-acetylt | taxID used:135614 OG03092|WP\_017116551.1 | MULTISPECIES: hydrolase [Xanthomona | taxID used:135614 OG03093|WP\_026112871.1 | MULTISPECIES: lysogenization regula | taxID used:135614 OG03094|WP\_039438596.1 | hypothetical protein [Xanthomonas v | taxID used:135614 OG03095|WP\_010364503.1 | MULTISPECIES: non-canonical purine | taxID used:135614 OG03096|WP\_010373682.1 | MULTISPECIES: flagellar assembly pr | taxID used:135614 OG03097|WP\_010374764.1 | MULTISPECIES: hypothetical protein | taxID used:135614 OG03098|WP\_017116017.1 | MULTISPECIES: GIY-YIG nuclease fami | taxID used:135614 OG03099|WP\_010363506.1 | MULTISPECIES: hypothetical protein | taxID used:135614 OG00309|WP\_017118093.1 | methyl-accepting chemotaxis protein | taxID used:135614 OG00030|WP\_010366781.1 | TIGR02099 family protein, partial [ | taxID used:135614 OG03100|WP\_010366391.1 | MULTISPECIES: TIGR00730 family Ross | taxID used:135614 OG03101|WP\_010373369.1 | MULTISPECIES: NfuA family Fe-S biog | taxID used:135614 OG03102|WP\_010369118.1 | MULTISPECIES: pyridoxamine 5'-phosp | taxID used:135614 OG03103|WP\_010371523.1 | MULTISPECIES: membrane protein [Xan | taxID used:135614 OG03104|WP\_010374349.1 | MULTISPECIES: 4'-phosphopantetheiny | taxID used:135614 OG03105|WP\_010366055.1 | MULTISPECIES: RNA 2',3'-cyclic phos | taxID used:135614 OG03106|WP\_010370585.1 | MULTISPECIES: Ax21 family protein [ | taxID used:135614 OG03107|WP\_010368420.1 | MULTISPECIES: DUF1003 domain-contai | taxID used:135614 OG03108|WP\_082347483.1 | MULTISPECIES: prepilin-type N-termi | taxID used:135614 OG03109|WP\_010373511.1 | MULTISPECIES: DUF4142 domain-contai | taxID used:135614 OG00310|WP\_010372423.1 | MULTISPECIES: NADP-dependent isocit | taxID used:135614 OG03110|WP\_010366101.1 | MULTISPECIES: HAD-IB family hydrola | taxID used:135614 OG03111|WP\_017115910.1 | TlpA family protein disulfide reduc | taxID used:135614 OG03112|WP\_010369419.1 | MULTISPECIES: nucleotidyltransferas | taxID used:135614 OG03113|WP\_039437449.1 | hypothetical protein [Xanthomonas v | taxID used:135614 OG03114|WP\_010372046.1 | DUF1453 domain-containing protein [ | taxID used:135614 OG03115|WP\_010366869.1 | MULTISPECIES: plasmid pRiA4b ORF-3 | taxID used:135614 OG03116|WP\_026112604.1 | DUF3106 domain-containing protein, | taxID used:135614 OG03117|WP\_017112239.1 | MULTISPECIES: glycine zipper 2TM do | taxID used:135614 OG03118|WP\_042504619.1 | hypothetical protein [Xanthomonas v | taxID used:135614 OG03119|WP\_010367164.1 | MULTISPECIES: imidazole glycerol ph | taxID used:135614 OG00311|WP\_054393913.1 | methyl-accepting chemotaxis protein | taxID used:135614 OG03120|WP\_017116028.1 | hypothetical protein, partial [Xant | taxID used:135614 OG03121|WP\_010369855.1 | MULTISPECIES: hypothetical protein | taxID used:135614 OG03122|WP\_010372897.1 | DsbE family thiol:disulfide interch | taxID used:135614 OG03123|WP\_017123173.1 | hypothetical protein, partial [Xant | taxID used:135614 OG03124|WP\_054393968.1 | MULTISPECIES: trimeric intracellula | taxID used:135614 OG03125|WP\_017112795.1 | MULTISPECIES: DUF1949 domain-contai | taxID used:135614 OG03126|WP\_078516405.1 | MULTISPECIES: hypothetical protein | taxID used:135614 OG03127|WP\_017117417.1 | MULTISPECIES: DUF998 domain-contain | taxID used:135614 OG03128|WP\_017116381.1 | MULTISPECIES: hypothetical protein | taxID used:135614 OG03129|WP\_010371494.1 | MULTISPECIES: hypothetical protein | taxID used:135614 OG00312|WP\_010371182.1 | MULTISPECIES: NADH-quinone oxidored | taxID used:135614 OG03130|WP\_054393950.1 | MULTISPECIES: hypothetical protein | taxID used:135614 OG03131|WP\_010369585.1 | AAA family ATPase [Xanthomonas vasi | taxID used:135614 OG03132|WP\_010370635.1 | MULTISPECIES: superoxide dismutase | taxID used:135614 OG03133|WP\_039431394.1 | lytic transglycosylase [Xanthomonas | taxID used:135614 OG03134|WP\_039431389.1 | integrating conjugative element pro | taxID used:135614 OG03135|WP\_106888066.1 | MULTISPECIES: hypothetical protein | taxID used:135614 OG03136|WP\_017115856.1 | LysE family translocator [Xanthomon | taxID used:135614 OG03137|WP\_010365269.1 | MULTISPECIES: chemoreceptor glutami | taxID used:135614 OG03138|WP\_123184322.1 | hypothetical protein, partial [Xant | taxID used:135614 OG03139|WP\_010374671.1 | MULTISPECIES: cytochrome c oxidase | taxID used:135614 OG00313|WP\_010367530.1 | MULTISPECIES: TonB-dependent hemogl | taxID used:135614 OG03141|WP\_010364248.1 | MULTISPECIES: biliverdin-producing | taxID used:135614 OG03142|WP\_010371054.1 | MULTISPECIES: 50S ribosomal protein | taxID used:135614 OG03143|WP\_010364471.1 | MULTISPECIES: 5-formyltetrahydrofol | taxID used:135614 OG03145|WP\_010363108.1 | MULTISPECIES: repressor LexA [Xanth | taxID used:135614 OG03146|WP\_017117296.1 | hypothetical protein, partial [Xant | taxID used:135614 OG03147|WP\_039431452.1 | transglycosylase, partial [Xanthomo | taxID used:135614 OG03148|WP\_010365925.1 | MULTISPECIES: DUF937 domain-contain | taxID used:135614 OG03149|WP\_010381254.1 | MULTISPECIES: DUF4276 domain-contai | taxID used:135614 OG00314|WP\_010368428.1 | MULTISPECIES: TonB-dependent recept | taxID used:135614 OG03150|WP\_017122844.1 | hypothetical protein, partial [Xant | taxID used:135614 OG03151|WP\_010369308.1 | MULTISPECIES: DUF1439 domain-contai | taxID used:135614 OG03152|WP\_017119549.1 | MULTISPECIES: ATP-dependent proteas | taxID used:135614 OG03153|WP\_010363682.1 | MULTISPECIES: DNA-directed RNA poly | taxID used:135614 OG03154|WP\_010371261.1 | MULTISPECIES: YhgN family NAAT tran | taxID used:135614 OG03155|WP\_010373214.1 | MULTISPECIES: oligoribonuclease [Xa | taxID used:135614 OG03156|WP\_017115257.1 | hypothetical protein, partial [Xant | taxID used:135614 OG03157|WP\_022557751.1 | hypothetical protein, partial [Xant | taxID used:135614 OG03158|WP\_010364592.1 | MULTISPECIES: hypothetical protein | taxID used:135614 OG03159|WP\_010366514.1 | N-acetylmuramoyl-L-alanine amidase, | taxID used:135614 OG00315|WP\_010370101.1 | MULTISPECIES: primosomal protein N' | taxID used:135614 OG03160|WP\_026112909.1 | MULTISPECIES: hypothetical protein | taxID used:135614 OG03162|WP\_053012998.1 | hypothetical protein, partial [Xant | taxID used:135614 OG03163|WP\_010367457.1 | MULTISPECIES: hypothetical protein | taxID used:135614 OG03164|WP\_010370172.1 | hypothetical protein, partial [Xant | taxID used:135614 OG03165|WP\_010371246.1 | MULTISPECIES: superoxide dismutase | taxID used:135614 OG03166|WP\_010367701.1 | MULTISPECIES: riboflavin synthase [ | taxID used:135614 OG03167|WP\_010366029.1 | MULTISPECIES: mechanosensitive ion | taxID used:135614 OG03168|WP\_010369190.1 | pectate lyase, partial [Xanthomonas | taxID used:135614 OG03169|WP\_010367335.1 | MULTISPECIES: NAD(P)H:quinone oxido | taxID used:135614 OG00316|WP\_082345103.1 | peptidase domain-containing ABC tra | taxID used:135614 OG03170|WP\_039436264.1 | MULTISPECIES: DUF239 domain-contain | taxID used:135614 OG03171|WP\_010365139.1 | MULTISPECIES: recombination protein | taxID used:135614 OG03172|WP\_010374843.1 | MULTISPECIES: response regulator tr | taxID used:135614 OG03173|WP\_010371930.1 | MULTISPECIES: hypothetical protein | taxID used:135614 OG03174|WP\_010376425.1 | MULTISPECIES: hypothetical protein | taxID used:135614 OG03175|WP\_010367755.1 | MULTISPECIES: nicotinamide riboside | taxID used:135614 OG03176|WP\_026112910.1 | MULTISPECIES: FMN reductase [Xantho | taxID used:135614 OG03177|WP\_010365543.1 | MULTISPECIES: hypothetical protein | taxID used:135614 OG03178|WP\_010369343.1 | MULTISPECIES: DNA-3-methyladenine g | taxID used:135614 OG03179|WP\_008571534.1 | polyisoprenoid-binding protein [Xan | taxID used:135614 OG00317|WP\_010364571.1 | MULTISPECIES: ferric-rhodotorulic a | taxID used:135614 OG03180|WP\_010368061.1 | MULTISPECIES: aminodeoxychorismate/ | taxID used:135614 OG03181|WP\_026112674.1 | MULTISPECIES: S-(hydroxymethyl)glut | taxID used:135614 OG03182|WP\_010371766.1 | MULTISPECIES: malonic semialdehyde | taxID used:135614 OG03183|WP\_017112356.1 | polyhydroxyalkanoate synthesis repr | taxID used:135614 OG03184|WP\_010367508.1 | MULTISPECIES: ATP-binding cassette | taxID used:135614 OG03185|WP\_039431825.1 | hypothetical protein [Xanthomonas v | taxID used:135614 OG03186|WP\_010366559.1 | MULTISPECIES: 3-hydroxyanthranilate | taxID used:135614 OG03187|WP\_010366360.1 | MULTISPECIES: nucleotide exchange f | taxID used:135614 OG03188|WP\_010363129.1 | MULTISPECIES: 2Fe-2S iron-sulfur cl | taxID used:135614 OG03189|WP\_010368007.1 | MULTISPECIES: thioredoxin family pr | taxID used:135614 OG00318|WP\_010364937.1 | exodeoxyribonuclease V subunit alph | taxID used:135614 OG03190|WP\_039443846.1 | MULTISPECIES: hypothetical protein | taxID used:135614 OG03191|WP\_010366727.1 | MULTISPECIES: deoxycytidine triphos | taxID used:135614 OG03192|WP\_010369221.1 | MULTISPECIES: LPS export ABC transp | taxID used:135614 OG03193|WP\_017120073.1 | MULTISPECIES: hypothetical protein | taxID used:135614 OG03194|WP\_010374814.1 | MULTISPECIES: DUF4142 domain-contai | taxID used:135614 OG03195|WP\_010366940.1 | MULTISPECIES: hypothetical protein | taxID used:135614 OG03196|WP\_010368814.1 | hypothetical protein [Xanthomonas v | taxID used:135614 OG03198|WP\_017113148.1 | MULTISPECIES: sugar O-acetyltransfe | taxID used:135614 OG03199|WP\_017115839.1 | MULTISPECIES: hypothetical protein | taxID used:135614 OG00319|WP\_010368115.1 | MULTISPECIES: TIGR01666 family memb | taxID used:135614 OG00031|WP\_010374431.1 | MULTISPECIES: translocation/assembl | taxID used:135614 OG03200|WP\_010371208.1 | MULTISPECIES: ribosome maturation f | taxID used:135614 OG03201|WP\_039440905.1 | TetR/AcrR family transcriptional re | taxID used:135614 OG03202|WP\_039437145.1 | hypothetical protein [Xanthomonas v | taxID used:135614 OG03203|WP\_010364006.1 | MULTISPECIES: hypothetical protein | taxID used:135614 OG03204|WP\_039433034.1 | hypothetical protein [Xanthomonas v | taxID used:135614 OG03205|WP\_010364050.1 | hypothetical protein, partial [Xant | taxID used:135614 OG03206|WP\_033009547.1 | MULTISPECIES: DUF924 domain-contain | taxID used:135614 OG03207|WP\_017115166.1 | MULTISPECIES: FMN-dependent NADH-az | taxID used:135614 OG03209|WP\_010369129.1 | MULTISPECIES: DUF1190 domain-contai | taxID used:135614 OG00320|WP\_010369413.1 | MULTISPECIES: xanthine dehydrogenas | taxID used:135614 OG03210|WP\_010374415.1 | MULTISPECIES: gluconokinase [Xantho | taxID used:135614 OG03211|WP\_010365400.1 | MULTISPECIES: hypothetical protein | taxID used:135614 OG03212|WP\_010367298.1 | MULTISPECIES: aminoacyl-tRNA hydrol | taxID used:135614 OG03213|WP\_010365222.1 | DUF2813 domain-containing protein, | taxID used:135614 OG03214|WP\_010367150.1 | MULTISPECIES: acireductone dioxygen | taxID used:135614 OG03215|WP\_010382083.1 | MULTISPECIES: Ax21 family protein [ | taxID used:135614 OG03216|WP\_010372112.1 | MULTISPECIES: DUF3016 domain-contai | taxID used:135614 OG03217|WP\_010368398.1 | MULTISPECIES: septum formation inhi | taxID used:135614 OG03218|WP\_010368302.1 | membrane protein, partial [Xanthomo | taxID used:135614 OG03219|WP\_080764573.1 | hypothetical protein [Xanthomonas v | taxID used:135614 OG00321|WP\_082346491.1 | methyl-accepting chemotaxis protein | taxID used:135614 OG03220|WP\_100243560.1 | hypothetical protein [Xanthomonas v | taxID used:135614 OG03221|WP\_026112797.1 | MULTISPECIES: hypothetical protein | taxID used:135614 OG03222|WP\_010374664.1 | hypothetical protein, partial [Xant | taxID used:135614 OG03223|WP\_026112765.1 | MULTISPECIES: DUF2059 domain-contai | taxID used:135614 OG03224|WP\_010374535.1 | MULTISPECIES: aldehyde dehydrogenas | taxID used:135614 OG03225|WP\_017116946.1 | chromosome partitioning protein Par | taxID used:135614 OG03226|WP\_010377396.1 | MULTISPECIES: lytic transglycosylas | taxID used:135614 OG03228|WP\_010372152.1 | MULTISPECIES: hypothetical protein | taxID used:135614 OG03229|WP\_017116116.1 | MULTISPECIES: hypothetical protein | taxID used:135614 OG00322|WP\_010367067.1 | TonB-dependent receptor, partial [X | taxID used:135614 OG03230|WP\_010372769.1 | MULTISPECIES: elongation factor P [ | taxID used:135614 OG03231|WP\_010368756.1 | MULTISPECIES: cytochrome b [Xanthom | taxID used:135614 OG03232|WP\_010367372.1 | MULTISPECIES: nitroreductase [Xanth | taxID used:135614 OG03233|WP\_010374404.1 | MULTISPECIES: manganese efflux pump | taxID used:135614 OG03234|WP\_010370531.1 | methyltransferase domain-containing | taxID used:135614 OG03235|WP\_010369220.1 | MULTISPECIES: lipopolysaccharide tr | taxID used:135614 OG03236|WP\_010366776.1 | MULTISPECIES: DUF615 domain-contain | taxID used:135614 OG03237|WP\_010367987.1 | hypothetical protein [Xanthomonas v | taxID used:135614 OG03238|WP\_010365437.1 | MULTISPECIES: flavodoxin family pro | taxID used:135614 OG03239|WP\_010369724.1 | MULTISPECIES: hypothetical protein | taxID used:135614 OG00323|WP\_026112753.1 | MULTISPECIES: glycerophosphodiester | taxID used:135614 OG03240|WP\_017170425.1 | MULTISPECIES: hypothetical protein | taxID used:135614 OG03241|WP\_010363969.1 | MULTISPECIES: Holliday junction bra | taxID used:135614 OG03242|WP\_039441482.1 | hypothetical protein [Xanthomonas v | taxID used:135614 OG03243|WP\_010365877.1 | chemotaxis protein CheB, partial [X | taxID used:135614 OG03244|WP\_082345104.1 | MULTISPECIES: hypothetical protein | taxID used:135614 OG03245|WP\_010370391.1 | MULTISPECIES: ribosomal large subun | taxID used:135614 OG03246|WP\_017115370.1 | membrane protein [Xanthomonas vasic | taxID used:135614 OG03247|WP\_017116406.1 | DNA-binding response regulator [Xan | taxID used:135614 OG03248|WP\_010365557.1 | hypothetical protein [Xanthomonas v | taxID used:135614 OG03249|WP\_010372241.1 | hypothetical protein, partial [Xant | taxID used:135614 OG00324|WP\_010374449.1 | MULTISPECIES: glycine--tRNA ligase | taxID used:135614 OG03250|WP\_010374921.1 | ankyrin repeat domain-containing pr | taxID used:135614 OG03252|WP\_010371165.1 | MULTISPECIES: NADH-quinone oxidored | taxID used:135614 OG03253|WP\_017118740.1 | MULTISPECIES: molybdenum cofactor g | taxID used:135614 OG03254|WP\_039437055.1 | protein-S-isoprenylcysteine methylt | taxID used:135614 OG03255|WP\_097370633.1 | VWA domain-containing protein, part | taxID used:135614 OG03256|WP\_010364920.1 | MULTISPECIES: N-acetyltransferase [ | taxID used:135614 OG03257|WP\_113629558.1 | hypothetical protein, partial [Xant | taxID used:135614 OG03258|WP\_017113579.1 | MULTISPECIES: hypothetical protein | taxID used:135614 OG03259|WP\_010367427.1 | MULTISPECIES: nucleoside deaminase | taxID used:135614 OG00325|WP\_010371811.1 | MULTISPECIES: hypothetical protein | taxID used:135614 OG03260|WP\_010367401.1 | MULTISPECIES: glutamine amidotransf | taxID used:135614 OG03261|WP\_010367393.1 | MULTISPECIES: peroxiredoxin [Xantho | taxID used:135614 OG03262|WP\_010367585.1 | MULTISPECIES: DUF3228 domain-contai | taxID used:135614 OG03263|WP\_010366787.1 | MULTISPECIES: Maf-like protein [Xan | taxID used:135614 OG03264|WP\_010374348.1 | MULTISPECIES: hypothetical protein | taxID used:135614 OG03266|WP\_010368972.1 | MULTISPECIES: protocatechuate 3,4-d | taxID used:135614 OG03267|WP\_005993551.1 | MULTISPECIES: elongation factor P-l | taxID used:135614 OG03268|WP\_026112282.1 | MULTISPECIES: glutathione peroxidas | taxID used:135614 OG03269|WP\_010365627.1 | MULTISPECIES: dTDP-4-dehydrorhamnos | taxID used:135614 OG00326|WP\_010366242.1 | MULTISPECIES: glycosyl hydrolase [X | taxID used:135614 OG03270|WP\_010371987.1 | chorismate mutase AroQ, gamma subcl | taxID used:135614 OG03271|WP\_097370632.1 | hypothetical protein [Xanthomonas v | taxID used:135614 OG03272|WP\_010369349.1 | MULTISPECIES: YqgE/AlgH family prot | taxID used:135614 OG03273|WP\_039432686.1 | hypothetical protein [Xanthomonas v | taxID used:135614 OG03274|WP\_010372031.1 | MULTISPECIES: DNA-deoxyinosine glyc | taxID used:135614 OG03275|WP\_010368425.1 | MULTISPECIES: hypothetical protein | taxID used:135614 OG03276|WP\_010368439.1 | MULTISPECIES: bacterioferritin [Xan | taxID used:135614 OG03277|WP\_087910996.1 | hypothetical protein [Xanthomonas v | taxID used:135614 OG03278|WP\_010364679.1 | MULTISPECIES: hypothetical protein | taxID used:135614 OG03279|WP\_010366088.1 | MULTISPECIES: sigma-70 family RNA p | taxID used:135614 OG00327|WP\_039437134.1 | Clp protease ClpP [Xanthomonas vasi | taxID used:135614 OG03280|WP\_116894722.1 | hypothetical protein [Xanthomonas v | taxID used:135614 OG03281|WP\_010367286.1 | MULTISPECIES: transcription termina | taxID used:135614 OG03282|WP\_082347473.1 | MULTISPECIES: RND transporter [Xant | taxID used:135614 OG03283|WP\_010373639.1 | MULTISPECIES: PilZ domain-containin | taxID used:135614 OG03284|WP\_010364052.1 | phage baseplate assembly protein V, | taxID used:135614 OG03285|WP\_010379309.1 | MULTISPECIES: hypothetical protein | taxID used:135614 OG03286|WP\_010364925.1 | MULTISPECIES: microcystin dependent | taxID used:135614 OG03287|WP\_010366542.1 | MULTISPECIES: DUF2939 domain-contai | taxID used:135614 OG03288|WP\_010367352.1 | MULTISPECIES: putative Fe-S cluster | taxID used:135614 OG03289|WP\_011269741.1 | MULTISPECIES: DUF2857 domain-contai | taxID used:135614 OG00328|WP\_026112419.1 | MULTISPECIES: DNA helicase II [Xant | taxID used:135614 OG03290|WP\_010368671.1 | ADP compounds hydrolase NudE, parti | taxID used:135614 OG03291|WP\_010370072.1 | MULTISPECIES: tRNA threonylcarbamoy | taxID used:135614 OG03292|WP\_010368754.1 | MULTISPECIES: polyisoprenoid-bindin | taxID used:135614 OG03293|WP\_010371417.1 | MULTISPECIES: response regulator tr | taxID used:135614 OG03294|WP\_080762887.1 | MULTISPECIES: hypothetical protein | taxID used:135614 OG03295|WP\_010366443.1 | MULTISPECIES: glutathione peroxidas | taxID used:135614 OG03296|WP\_100243605.1 | hypothetical protein [Xanthomonas c | taxID used:135614 OG03297|WP\_010368178.1 | MULTISPECIES: TonB-dependent recept | taxID used:135614 OG03298|WP\_017115256.1 | hypothetical protein [Xanthomonas v | taxID used:135614 OG03299|WP\_017122460.1 | ATP-binding protein, partial [Xanth | taxID used:135614 OG00329|WP\_010371201.1 | MULTISPECIES: NADH-quinone oxidored | taxID used:135614 OG00032|WP\_010370700.1 | MULTISPECIES: indolepyruvate ferred | taxID used:135614 OG03300|WP\_010372080.1 | MULTISPECIES: DUF1415 domain-contai | taxID used:135614 OG03301|WP\_010372706.1 | MULTISPECIES: YaeQ family protein [ | taxID used:135614 OG03302|WP\_010364158.1 | MULTISPECIES: DNA starvation/statio | taxID used:135614 OG03303|WP\_010372241.1 | hypothetical protein [Xanthomonas v | taxID used:135614 OG03304|WP\_039436027.1 | MULTISPECIES: phage tail protein I | taxID used:135614 OG03305|WP\_026113097.1 | MULTISPECIES: plasmid pRiA4b ORF-3 | taxID used:135614 OG03306|WP\_010374593.1 | MULTISPECIES: N-acetyltransferase [ | taxID used:135614 OG03307|WP\_010365182.1 | MULTISPECIES: poly(hydroxyalcanoate | taxID used:135614 OG03308|WP\_010374549.1 | MULTISPECIES: hypothetical protein | taxID used:135614 OG03309|WP\_126720872.1 | hypothetical protein [Xanthomonas c | taxID used:135614 OG00330|WP\_010367969.1 | MULTISPECIES: glycogen debranching | taxID used:135614 OG03310|WP\_010365991.1 | MULTISPECIES: hypoxanthine-guanine | taxID used:135614 OG03311|WP\_010372726.1 | MULTISPECIES: adenine phosphoribosy | taxID used:135614 OG03312|WP\_010372571.1 | MULTISPECIES: hypothetical protein | taxID used:135614 OG03313|WP\_010366302.1 | MULTISPECIES: NAD(P)H-dependent oxi | taxID used:135614 OG03314|WP\_010364046.1 | phage tail protein I [Xanthomonas v | taxID used:135614 OG03315|WP\_010374840.1 | MULTISPECIES: cytochrome b [Xanthom | taxID used:135614 OG03316|WP\_010370878.1 | MULTISPECIES: O-acetyl-ADP-ribose d | taxID used:135614 OG03317|WP\_010364291.1 | MULTISPECIES: DUF1905 domain-contai | taxID used:135614 OG03318|WP\_010369388.1 | single-stranded DNA-binding protein | taxID used:135614 OG03319|WP\_010366007.1 | polymer-forming cytoskeletal family | taxID used:135614 OG00331|WP\_010370246.1 | MULTISPECIES: hypothetical protein | taxID used:135614 OG03320|WP\_087910787.1 | DUF4189 domain-containing protein [ | taxID used:135614 OG03321|WP\_010369009.1 | MULTISPECIES: gamma carbonic anhydr | taxID used:135614 OG03322|WP\_080762940.1 | MULTISPECIES: translation initiatio | taxID used:135614 OG03323|WP\_010363095.1 | MULTISPECIES: glyoxalase/bleomycin | taxID used:135614 OG03324|WP\_010370300.1 | MULTISPECIES: CPBP family intramemb | taxID used:135614 OG03325|WP\_010366165.1 | MULTISPECIES: ribosome recycling fa | taxID used:135614 OG03326|WP\_010370420.1 | MULTISPECIES: hypothetical protein | taxID used:135614 OG03327|WP\_017113538.1 | MULTISPECIES: sensor histidine kina | taxID used:135614 OG03328|WP\_010380360.1 | MULTISPECIES: SCPU domain-containin | taxID used:135614 OG03329|WP\_017113121.1 | MULTISPECIES: NUDIX domain-containi | taxID used:135614 OG00332|WP\_010367026.1 | MULTISPECIES: phospholipase C, phos | taxID used:135614 OG03330|WP\_010366075.1 | MULTISPECIES: DUF2878 domain-contai | taxID used:135614 OG03331|WP\_010365495.1 | MULTISPECIES: nuclear transport fac | taxID used:135614 OG03332|WP\_010370954.1 | MULTISPECIES: N-acetyltransferase [ | taxID used:135614 OG03333|WP\_026112601.1 | plasmid mobilization relaxosome pro | taxID used:135614 OG03334|WP\_010367459.1 | MULTISPECIES: cob(I)yrinic acid a,c | taxID used:135614 OG03335|WP\_010381550.1 | MULTISPECIES: hypothetical protein | taxID used:135614 OG03336|WP\_010369223.1 | 3-deoxy-D-manno-octulosonate 8-phos | taxID used:135614 OG03337|WP\_017117875.1 | MULTISPECIES: hypothetical protein | taxID used:135614 OG03338|WP\_010364422.1 | MULTISPECIES: adenylate kinase [Xan | taxID used:135614 OG03339|WP\_010371290.1 | MULTISPECIES: prepilin-type N-termi | taxID used:135614 OG00333|WP\_010364523.1 | MULTISPECIES: bifunctional (p)ppGpp | taxID used:135614 OG03340|WP\_026112219.1 | hypothetical protein [Xanthomonas v | taxID used:135614 OG03341|WP\_010379039.1 | Slp family lipoprotein, partial [Xa | taxID used:135614 OG03342|WP\_010374565.1 | MULTIS
[truncated: 209,809 more chars]
