# Supplementary material for: Orthology-Based Estimate of the Contribution of Horizontal Gene Transfer from Distantly Related Bacteria to the Intraspecific Diversity and Differentiation of Xylella fastidiosa
Source: Pathogens. 2021 Jan 7;10(1):46. doi: 10.3390/pathogens10010046 (PMC7828034; doi:10.3390/pathogens10010046)
Supplement: Supplementary file 1 [file pathogens-10-00046-s001.zip › pathogens-1031631-supplementary-final/pathogens-1031631-Figure S3.html]

Javascript must be enabled to view this page.

members
magnitude
magnitudeUnassigned
count
unassigned
taxon
rank

Busco
Core
Pangenome

44423863369

2
superkingdom
44423863369

201174
12
phylum

1760
class
12

1
order
85010

2070
1
family

695999
1
genus

485602 
1
species


OG03284|WP\_045760688.1 | hypothetical protein [Xanthomonas a | taxID used:485602 

85011
11
order

2062
11
family

1883
11
genus

11
1890286 


OG01290|WP\_080929712.1 | cytochrome P450 [Xanthomonas albili | taxID used:1890286 
OG01290|WP\_080929712.1 | cytochrome P450 [Xanthomonas albili | taxID used:1890286 
species

429581 
1
species


OG03152|WP\_080931604.1 | transcriptional regulator [Xanthomo | taxID used:429581 

142182
phylum
11

11
class
219685

11
order
219686

219687
11
family

11
genus
173479

11
1379270 


OG02147|WP\_045761476.1 | alpha/beta hydrolase [Xanthomonas a | taxID used:1379270 
OG02147|WP\_045761476.1 | alpha/beta hydrolase [Xanthomonas a | taxID used:1379270 
species

phylum
11
1930617

class
11
1962850

1962852
11
order

11
family
1962854

11
genus
187144

11
187145 
species


OG00138|WP\_012916339.1 | truncated non-ribosomal peptide syn | taxID used:187145 
OG00138|WP\_012916339.1 | truncated non-ribosomal peptide syn | taxID used:187145 

40117
2
phylum

2
class
203693

order
2
189778

189779
2
family

1234
2
genus

330214 
2
species


OG02884|WP\_045757014.1 | hypothetical protein [Xanthomonas a | taxID used:330214 OG03431|WP\_045758571.1 | hypothetical protein [Xanthomonas a | taxID used:330214 

1224
phylum
44423833357

class
44423723228
1236

135614 
44423713198

OG03154|WP\_012917381.1 | MULTISPECIES: F0F1 ATP synthase sub | taxID used:135614 OG00101|WP\_012916222.1 | DNA gyrase subunit A [Xanthomonas a | taxID used:135614 OG00104|WP\_012917275.1 | preprotein translocase subunit SecA | taxID used:135614 OG01056|WP\_080929791.1 | lipid-A-disaccharide synthase [Xant | taxID used:135614 OG01076|WP\_012916516.1 | 1-deoxy-D-xylulose-5-phosphate redu | taxID used:135614 OG01084|WP\_045756912.1 | tRNA-guanine(34) transglycosylase [ | taxID used:135614 OG00108|WP\_012914896.1 | pyruvate dehydrogenase (acetyl-tran | taxID used:135614 OG01090|WP\_012916784.1 | phosphoglycerate kinase [Xanthomona | taxID used:135614 OG01093|WP\_012915198.1 | MULTISPECIES: ADP-forming succinate | taxID used:135614 OG01098|WP\_012917330.1 | DNA-protecting protein DprA [Xantho | taxID used:135614 OG01101|WP\_012916174.1 | molecular chaperone DnaJ [Xanthomon | taxID used:135614 OG01102|WP\_012915786.1 | 5-(carboxyamino)imidazole ribonucle | taxID used:135614 OG01106|WP\_012915334.1 | rod shape-determining protein MreC | taxID used:135614 OG01117|WP\_012916500.1 | succinyl-diaminopimelate desuccinyl | taxID used:135614 OG01120|WP\_012915849.1 | carbamoyl-phosphate synthase small | taxID used:135614 OG00112|WP\_080931561.1 | bifunctional uridylyltransferase/ur | taxID used:135614 OG01133|WP\_080929716.1 | RNA polymerase-binding protein DksA | taxID used:135614 OG01139|WP\_012914939.1 | anhydro-N-acetylmuramic acid kinase | taxID used:135614 OG01161|WP\_012917141.1 | LPS export ABC transporter permease | taxID used:135614 OG01163|WP\_012915506.1 | chorismate synthase [Xanthomonas al | taxID used:135614 OG01168|WP\_012914568.1 | DNA replication and repair protein | taxID used:135614 OG00116|WP\_012916954.1 | leucine--tRNA ligase [Xanthomonas a | taxID used:135614 OG01199|WP\_012916649.1 | 3-dehydroquinate synthase [Xanthomo | taxID used:135614 OG00119|WP\_012915540.1 | translation initiation factor IF-2 | taxID used:135614 OG01200|WP\_012916201.1 | 3-phosphoserine/phosphohydroxythreo | taxID used:135614 OG01202|WP\_012917310.1 | bifunctional diaminohydroxyphosphor | taxID used:135614 OG01205|WP\_012914567.1 | DNA polymerase III subunit beta [Xa | taxID used:135614 OG01207|WP\_012917286.1 | phospho-N-acetylmuramoyl-pentapepti | taxID used:135614 OG01214|WP\_012915769.1 | bifunctional histidinol-phosphatase | taxID used:135614 OG01216|WP\_012915037.1 | peptide chain release factor 1 [Xan | taxID used:135614 OG01219|WP\_012915345.1 | lipoyl synthase [Xanthomonas albili | taxID used:135614 OG01220|WP\_045757336.1 | A/G-specific adenine glycosylase [X | taxID used:135614 OG01226|WP\_012915045.1 | redox-regulated ATPase YchF [Xantho | taxID used:135614 OG01227|WP\_045756718.1 | endolytic transglycosylase MltG [Xa | taxID used:135614 OG01228|WP\_012917142.1 | LPS export ABC transporter permease | taxID used:135614 OG01236|WP\_012915279.1 | 3-isopropylmalate dehydrogenase [Xa | taxID used:135614 OG01241|WP\_012915446.1 | GTPase ObgE [Xanthomonas albilinean | taxID used:135614 OG01249|WP\_012916654.1 | uroporphyrinogen decarboxylase [Xan | taxID used:135614 OG00125|WP\_012915692.1 | alanine--tRNA ligase [Xanthomonas a | taxID used:135614 OG01260|WP\_012914913.1 | biotin synthase BioB [Xanthomonas a | taxID used:135614 OG01266|WP\_045757315.1 | S-adenosylmethionine:tRNA ribosyltr | taxID used:135614 OG01269|WP\_012914843.1 | anthranilate phosphoribosyltransfer | taxID used:135614 OG01281|WP\_045756933.1 | phosphoribosylformylglycinamidine c | taxID used:135614 OG01285|WP\_012915333.1 | rod shape-determining protein [Xant | taxID used:135614 OG01288|WP\_012916305.1 | tetraacyldisaccharide 4'-kinase [Xa | taxID used:135614 OG01292|WP\_012915731.1 | UDP-N-acetylmuramate dehydrogenase | taxID used:135614 OG01295|WP\_012915732.1 | quinone-dependent dihydroorotate de | taxID used:135614 OG01319|WP\_012915690.1 | recombinase RecA [Xanthomonas albil | taxID used:135614 OG01328|WP\_012914806.1 | tRNA dihydrouridine(20/20a) synthas | taxID used:135614 OG01331|WP\_045756985.1 | Holliday junction branch migration | taxID used:135614 OG01335|WP\_012916952.1 | DNA polymerase III subunit delta [X | taxID used:135614 OG01340|WP\_012916519.1 | UDP-3-O-(3-hydroxymyristoyl)glucosa | taxID used:135614 OG01352|WP\_012917548.1 | glycerol-3-phosphate dehydrogenase | taxID used:135614 OG01357|WP\_012917255.1 | tRNA dihydrouridine synthase DusB [ | taxID used:135614 OG01361|WP\_012915597.1 | phenylalanine--tRNA ligase subunit | taxID used:135614 OG01371|WP\_012915223.1 | 23S rRNA pseudouridine(1911/1915/19 | taxID used:135614 OG01373|WP\_012917291.1 | 16S rRNA (cytosine(1402)-N(4))-meth | taxID used:135614 OG01386|WP\_012915564.1 | octaprenyl-diphosphate synthase [Xa | taxID used:135614 OG01387|WP\_012915078.1 | DNA-directed RNA polymerase subunit | taxID used:135614 OG01388|WP\_012915449.1 | bifunctional riboflavin kinase/FAD | taxID used:135614 OG01398|WP\_012916117.1 | RluA family pseudouridine synthase | taxID used:135614 OG00139|WP\_012916712.1 | DNA mismatch repair protein MutS [X | taxID used:135614 OG00140|WP\_012915240.1 | ATP-dependent chaperone ClpB [Xanth | taxID used:135614 OG01410|WP\_012914634.1 | ferrochelatase [Xanthomonas albilin | taxID used:135614 OG01416|WP\_045756822.1 | thioredoxin-disulfide reductase [Xa | taxID used:135614 OG01435|WP\_012917305.1 | thiamine-phosphate kinase [Xanthomo | taxID used:135614 OG01451|WP\_012917233.1 | 4-hydroxythreonine-4-phosphate dehy | taxID used:135614 OG01457|WP\_012916525.1 | acetyl-CoA carboxylase carboxyltran | taxID used:135614 OG01465|WP\_012915042.1 | ribose-phosphate pyrophosphokinase | taxID used:135614 OG01468|WP\_012915452.1 | 4-hydroxy-3-methylbut-2-enyl diphos | taxID used:135614 OG01479|WP\_012915685.1 | tRNA (adenosine(37)-N6)-dimethylall | taxID used:135614 OG01504|WP\_045757152.1 | bifunctional biotin--[acetyl-CoA-ca | taxID used:135614 OG01513|WP\_012914643.1 | glycine--tRNA ligase subunit alpha | taxID used:135614 OG01527|WP\_012917236.1 | symmetrical bis(5'-nucleosyl)-tetra | taxID used:135614 OG01536|WP\_045756762.1 | 50S ribosomal protein L3 N(5)-gluta | taxID used:135614 OG01539|WP\_012917278.1 | UDP-3-O-acyl-N-acetylglucosamine de | taxID used:135614 OG01542|WP\_012917327.1 | methionyl-tRNA formyltransferase [X | taxID used:135614 OG01548|WP\_012914915.1 | 4-hydroxybenzoate octaprenyltransfe | taxID used:135614 OG01554|WP\_012914870.1 | 50S ribosomal protein L11 methyltra | taxID used:135614 OG00155|WP\_012917334.1 | DNA topoisomerase I [Xanthomonas al | taxID used:135614 OG01561|WP\_012915542.1 | tRNA pseudouridine(55) synthase Tru | taxID used:135614 OG01563|WP\_012915766.1 | ATP phosphoribosyltransferase [Xant | taxID used:135614 OG00156|WP\_080931571.1 | LPS-assembly protein LptD [Xanthomo | taxID used:135614 OG01581|WP\_012915222.1 | outer membrane protein assembly fac | taxID used:135614 OG01593|WP\_012917060.1 | hydroxymethylbilane synthase [Xanth | taxID used:135614 OG01596|WP\_012917239.1 | prolipoprotein diacylglyceryl trans | taxID used:135614 OG00159|WP\_012916226.1 | NAD-dependent DNA ligase LigA [Xant | taxID used:135614 OG01605|WP\_012915515.1 | acetyl-CoA carboxylase carboxyltran | taxID used:135614 OG01606|WP\_012917014.1 | GTPase Era [Xanthomonas albilineans | taxID used:135614 OG01612|WP\_012916676.1 | Hsp33 family molecular chaperone Hs | taxID used:135614 OG01615|WP\_012916626.1 | RNase adapter RapZ [Xanthomonas alb | taxID used:135614 OG01627|WP\_012917345.1 | RNA polymerase sigma factor RpoH [X | taxID used:135614 OG01629|WP\_012915619.1 | protease HtpX [Xanthomonas albiline | taxID used:135614 OG00163|WP\_012914569.1 | DNA topoisomerase (ATP-hydrolyzing) | taxID used:135614 OG01645|WP\_012914630.1 | tRNA 2-thiocytidine(32) synthetase | taxID used:135614 OG01646|WP\_012916569.1 | CBS domain-containing protein [Xant | taxID used:135614 OG01655|WP\_012916091.1 | DUF4115 domain-containing protein [ | taxID used:135614 OG01657|WP\_012917377.1 | F0F1 ATP synthase subunit gamma [Xa | taxID used:135614 OG01663|WP\_012915199.1 | succinate--CoA ligase subunit alpha | taxID used:135614 OG01664|WP\_012916928.1 | farnesyl-diphosphate synthase [Xant | taxID used:135614 OG01667|WP\_012916511.1 | elongation factor Ts [Xanthomonas a | taxID used:135614 OG01669|WP\_012917051.1 | diaminopimelate epimerase [Xanthomo | taxID used:135614 OG01670|WP\_012917013.1 | DNA repair protein RecO [Xanthomona | taxID used:135614 OG01673|WP\_012917281.1 | cell division protein FtsQ [Xanthom | taxID used:135614 OG01692|WP\_012916319.1 | folate-binding protein [Xanthomonas | taxID used:135614 OG00169|WP\_012916832.1 | penicillin-binding protein 1A [Xant | taxID used:135614 OG01703|WP\_012915503.1 | phosphatidylserine decarboxylase [X | taxID used:135614 OG01708|WP\_012915495.1 | iron-sulfur cluster carrier protein | taxID used:135614 OG01709|WP\_045756709.1 | 4-(cytidine 5'-diphospho)-2-C-methy | taxID used:135614 OG01719|WP\_012915720.1 | pantoate--beta-alanine ligase [Xant | taxID used:135614 OG00171|WP\_012915124.1 | endopeptidase La [Xanthomonas albil | taxID used:135614 OG01729|WP\_012915669.1 | 3-deoxy-8-phosphooctulonate synthas | taxID used:135614 OG00172|WP\_012916486.1 | ribonuclease R [Xanthomonas albilin | taxID used:135614 OG01736|WP\_012917463.1 | shikimate dehydrogenase [Xanthomona | taxID used:135614 OG01738|WP\_012915060.1 | MULTISPECIES: 50S ribosomal protein | taxID used:135614 OG01752|WP\_012917202.1 | NADPH-dependent 7-cyano-7-deazaguan | taxID used:135614 OG01753|WP\_012916646.1 | pyrroline-5-carboxylate reductase [ | taxID used:135614 OG01762|WP\_012915012.1 | peptide chain release factor N(5)-g | taxID used:135614 OG00176|WP\_012916518.1 | outer membrane protein assembly fac | taxID used:135614 OG01773|WP\_012917632.1 | bifunctional DNA-formamidopyrimidin | taxID used:135614 OG01781|WP\_012914928.1 | SPOR domain-containing protein [Xan | taxID used:135614 OG01788|WP\_012917238.1 | thymidylate synthase [Xanthomonas a | taxID used:135614 OG01790|WP\_012917382.1 | F0F1 ATP synthase subunit A [Xantho | taxID used:135614 OG01797|WP\_012917294.1 | 16S rRNA (cytidine(1402)-2'-O)-meth | taxID used:135614 OG01812|WP\_012916510.1 | 30S ribosomal protein S2 [Xanthomon | taxID used:135614 OG01815|WP\_012916256.1 | NAD kinase [Xanthomonas albilineans | taxID used:135614 OG01828|WP\_012915481.1 | tRNA (guanosine(37)-N1)-methyltrans | taxID used:135614 OG01832|WP\_012916521.1 | acyl-ACP--UDP-N-acetylglucosamine O | taxID used:135614 OG01833|WP\_012917234.1 | 16S rRNA (adenine(1518)-N(6)/adenin | taxID used:135614 OG01837|WP\_012915127.1 | hydroxyacylglutathione hydrolase [X | taxID used:135614 OG01838|WP\_045756941.1 | nucleoside triphosphate pyrophospho | taxID used:135614 OG01840|WP\_012914664.1 | exodeoxyribonuclease III [Xanthomon | taxID used:135614 OG01843|WP\_012915510.1 | tRNA pseudouridine(38-40) synthase | taxID used:135614 OG01848|WP\_045757215.1 | tryptophan synthase subunit alpha [ | taxID used:135614 OG01852|WP\_012914844.1 | indole-3-glycerol phosphate synthas | taxID used:135614 OG01856|WP\_045756758.1 | membrane protein [Xanthomonas albil | taxID used:135614 OG01863|WP\_012916304.1 | 3-deoxy-manno-octulosonate cytidyly | taxID used:135614 OG01868|WP\_012915772.1 | imidazole glycerol phosphate syntha | taxID used:135614 OG01873|WP\_012914592.1 | ABC transporter ATP-binding protein | taxID used:135614 OG01874|WP\_012916744.1 | tRNA (guanosine(46)-N7)-methyltrans | taxID used:135614 OG01882|WP\_045757068.1 | uroporphyrinogen III methyltransfer | taxID used:135614 OG01908|WP\_012915106.1 | colicin V biosynthesis protein [Xan | taxID used:135614 OG01927|WP\_012917346.1 | uracil-DNA glycosylase [Xanthomonas | taxID used:135614 OG01941|WP\_012916411.1 | RNA methyltransferase [Xanthomonas | taxID used:135614 OG01943|WP\_045756925.1 | di-trans,poly-cis-decaprenylcistran | taxID used:135614 OG01946|WP\_012915109.1 | UDP-2,3-diacylglucosamine diphospha | taxID used:135614 OG01947|WP\_012915523.1 | triose-phosphate isomerase [Xanthom | taxID used:135614 OG01948|WP\_045756687.1 | amidophosphoribosyltransferase [Xan | taxID used:135614 OG01958|WP\_012916406.1 | bifunctional 2-polyprenyl-6-hydroxy | taxID used:135614 OG01968|WP\_012916485.1 | 23S rRNA (guanosine(2251)-2'-O)-met | taxID used:135614 OG01969|WP\_012915155.1 | 3-oxoacyl-ACP reductase FabG [Xanth | taxID used:135614 OG00196|WP\_012915598.1 | phenylalanine--tRNA ligase subunit | taxID used:135614 OG01976|WP\_012915130.1 | DNA polymerase III subunit epsilon | taxID used:135614 OG01978|WP\_012914612.1 | orotidine-5'-phosphate decarboxylas | taxID used:135614 OG01981|WP\_012914593.1 | ABC transporter permease [Xanthomon | taxID used:135614 OG01986|WP\_012915062.1 | 30S ribosomal protein S3 [Xanthomon | taxID used:135614 OG01992|WP\_012916228.1 | cell division protein ZipA [Xanthom | taxID used:135614 OG01999|WP\_012915771.1 | 1-(5-phosphoribosyl)-5-[(5-phosphor | taxID used:135614 OG02006|WP\_012916695.1 | 16S rRNA (uracil(1498)-N(3))-methyl | taxID used:135614 OG02015|WP\_012915323.1 | ribonuclease PH [Xanthomonas albili | taxID used:135614 OG02020|WP\_012916512.1 | UMP kinase [Xanthomonas albilineans | taxID used:135614 OG02033|WP\_012915344.1 | lipoyl(octanoyl) transferase LipB [ | taxID used:135614 OG02036|WP\_012916622.1 | LPS export ABC transporter ATP-bind | taxID used:135614 OG02045|WP\_012915848.1 | 4-hydroxy-tetrahydrodipicolinate re | taxID used:135614 OG02060|WP\_012916728.1 | tRNA (adenosine(37)-N6)-threonylcar | taxID used:135614 OG02071|WP\_012915672.1 | 2-C-methyl-D-erythritol 4-phosphate | taxID used:135614 OG02072|WP\_012916473.1 | endonuclease III [Xanthomonas albil | taxID used:135614 OG02081|WP\_012915050.1 | 50S ribosomal protein L1 [Xanthomon | taxID used:135614 OG02098|WP\_012915160.1 | dTMP kinase [Xanthomonas albilinean | taxID used:135614 OG00020|WP\_012915054.1 | DNA-directed RNA polymerase subunit | taxID used:135614 OG02115|WP\_045757388.1 | ribonuclease III [Xanthomonas albil | taxID used:135614 OG02116|WP\_080928215.1 | YggS family pyridoxal phosphate-dep | taxID used:135614 OG02118|WP\_012914837.1 | ribulose-phosphate 3-epimerase [Xan | taxID used:135614 OG02128|WP\_012916105.1 | (d)CMP kinase [Xanthomonas albiline | taxID used:135614 OG02136|WP\_012916893.1 | 7-cyano-7-deazaguanine synthase Que | taxID used:135614 OG02137|WP\_012916484.1 | ribonuclease T [Xanthomonas albilin | taxID used:135614 OG02143|WP\_012915040.1 | lipoprotein localization protein Lo | taxID used:135614 OG02155|WP\_080928209.1 | ribonuclease HII [Xanthomonas albil | taxID used:135614 OG02166|WP\_012916611.1 | phosphoribosylglycinamide formyltra | taxID used:135614 OG02169|WP\_012915058.1 | 50S ribosomal protein L3 [Xanthomon | taxID used:135614 OG02173|WP\_012916300.1 | CDP-diacylglycerol--glycerol-3-phos | taxID used:135614 OG02174|WP\_012915043.1 | 50S ribosomal protein L25 [Xanthomo | taxID used:135614 OG02178|WP\_012915310.1 | ribose-5-phosphate isomerase RpiA [ | taxID used:135614 OG02192|WP\_012915780.1 | stringent starvation protein A [Xan | taxID used:135614 OG02195|WP\_012914935.1 | orotate phosphoribosyltransferase [ | taxID used:135614 OG02199|WP\_045756790.1 | 23S rRNA (uridine(2552)-2'-O)-methy | taxID used:135614 OG00021|WP\_012915053.1 | DNA-directed RNA polymerase subunit | taxID used:135614 OG02207|WP\_012915325.1 | guanylate kinase [Xanthomonas albil | taxID used:135614 OG02208|WP\_012914853.1 | RNA pyrophosphohydrolase [Xanthomon | taxID used:135614 OG02232|WP\_012914668.1 | 16S rRNA (guanine(527)-N(7))-methyl | taxID used:135614 OG02233|WP\_012915077.1 | 30S ribosomal protein S4 [Xanthomon | taxID used:135614 OG02234|WP\_012915122.1 | ATP-dependent Clp endopeptidase, pr | taxID used:135614 OG02235|WP\_012915962.1 | outer membrane lipoprotein carrier | taxID used:135614 OG02236|WP\_012916090.1 | membrane protein [Xanthomonas albil | taxID used:135614 OG02238|WP\_012915382.1 | YihA family ribosome biogenesis GTP | taxID used:135614 OG00223|WP\_012916496.1 | DNA topoisomerase IV subunit A [Xan | taxID used:135614 OG02270|WP\_012915191.1 | dephospho-CoA kinase [Xanthomonas a | taxID used:135614 OG02282|WP\_012915952.1 | lysogenization regulator HflD [Xant | taxID used:135614 OG02284|WP\_012915143.1 | recombination protein RecR [Xanthom | taxID used:135614 OG02304|WP\_045757240.1 | imidazole glycerol phosphate syntha | taxID used:135614 OG02308|WP\_012916430.1 | 16S rRNA (guanine(966)-N(2))-methyl | taxID used:135614 OG02309|WP\_012916621.1 | lipopolysaccharide transport peripl | taxID used:135614 OG02310|WP\_012915312.1 | 5-formyltetrahydrofolate cyclo-liga | taxID used:135614 OG02316|WP\_012915059.1 | 50S ribosomal protein L4 [Xanthomon | taxID used:135614 OG02318|WP\_012915538.1 | ribosome maturation factor RimP [Xa | taxID used:135614 OG02319|WP\_012915322.1 | non-canonical purine NTP pyrophosph | taxID used:135614 OG02323|WP\_012916113.1 | Fe-S biogenesis protein NfuA [Xanth | taxID used:135614 OG02339|WP\_012916146.1 | oligoribonuclease [Xanthomonas albi | taxID used:135614 OG02345|WP\_012915044.1 | aminoacyl-tRNA hydrolase [Xanthomon | taxID used:135614 OG02353|WP\_012916931.1 | DUF615 domain-containing protein [X | taxID used:135614 OG02369|WP\_012916904.1 | Holliday junction branch migration | taxID used:135614 OG02393|WP\_012915048.1 | transcription termination/antitermi | taxID used:135614 OG02398|WP\_012916640.1 | YqgE/AlgH family protein [Xanthomon | taxID used:135614 OG00239|WP\_012917343.1 | primosomal protein N' [Xanthomonas | taxID used:135614 OG02418|WP\_012917335.1 | tRNA threonylcarbamoyladenosine bio | taxID used:135614 OG02422|WP\_080928065.1 | translation initiation factor IF-3 | taxID used:135614 OG02428|WP\_012916513.1 | ribosome recycling factor [Xanthomo | taxID used:135614 OG00243|WP\_012915327.1 | bifunctional (p)ppGpp synthetase/gu | taxID used:135614 OG02443|WP\_012916619.1 | 3-deoxy-D-manno-octulosonate 8-phos | taxID used:135614 OG02449|WP\_012915068.1 | 50S ribosomal protein L5 [Xanthomon | taxID used:135614 OG02450|WP\_012915296.1 | adenylate kinase [Xanthomonas albil | taxID used:135614 OG02456|WP\_012915073.1 | 30S ribosomal protein S5 [Xanthomon | taxID used:135614 OG02460|WP\_012916648.1 | shikimate kinase [Xanthomonas albil | taxID used:135614 OG00246|WP\_012916462.1 | bifunctional 23S rRNA (guanine(2069 | taxID used:135614 OG02476|WP\_012915316.1 | hypothetical protein [Xanthomonas a | taxID used:135614 OG02479|WP\_012917312.1 | transcriptional repressor NrdR [Xan | taxID used:135614 OG02499|WP\_012915051.1 | 50S ribosomal protein L10 [Xanthomo | taxID used:135614 OG02510|WP\_012915071.1 | 50S ribosomal protein L6 [Xanthomon | taxID used:135614 OG02522|WP\_012917549.1 | protein-export chaperone SecB [Xant | taxID used:135614 OG02527|WP\_012917379.1 | F0F1 ATP synthase subunit delta [Xa | taxID used:135614 OG02528|WP\_012916176.1 | MULTISPECIES: nucleotide exchange f | taxID used:135614 OG02531|WP\_012915480.1 | ribosome maturation factor RimM [Xa | taxID used:135614 OG02537|WP\_012916183.1 | MULTISPECIES: SsrA-binding protein | taxID used:135614 OG02540|WP\_045757374.1 | crossover junction endodeoxyribonuc | taxID used:135614 OG02549|WP\_045756717.1 | characterized ACR protein [Xanthomo | taxID used:135614 OG02559|WP\_012916429.1 | pantetheine-phosphate adenylyltrans | taxID used:135614 OG02560|WP\_012915673.1 | 2-C-methyl-D-erythritol 2,4-cyclodi | taxID used:135614 OG02563|WP\_012915785.1 | 5-(carboxyamino)imidazole ribonucle | taxID used:135614 OG02566|WP\_049887217.1 | dihydrofolate reductase [Xanthomona | taxID used:135614 OG02570|WP\_012916147.1 | tRNA adenosine(34) deaminase TadA [ | taxID used:135614 OG02577|WP\_012917146.1 | ribosomal-protein-alanine N-acetylt | taxID used:135614 OG02581|WP\_012916639.1 | Holliday junction resolvase RuvX [X | taxID used:135614 OG02584|WP\_012915691.1 | recombination regulator RecX [Xanth | taxID used:135614 OG02609|WP\_012915335.1 | rod shape-determining protein MreD | taxID used:135614 OG02610|WP\_012916567.1 | rRNA maturation RNase YbeY [Xanthom | taxID used:135614 OG02611|WP\_012915611.1 | tRNA (adenosine(37)-N6)-threonylcar | taxID used:135614 OG02612|WP\_012914872.1 | acetyl-CoA carboxylase biotin carbo | taxID used:135614 OG00261|WP\_045756947.1 | bifunctional (p)ppGpp synthetase/gu | taxID used:135614 OG02624|WP\_012917546.1 | tRNA (uridine(34)/cytosine(34)/5-ca | taxID used:135614 OG02647|WP\_012915056.1 | 30S ribosomal protein S7 [Xanthomon | taxID used:135614 OG02650|WP\_012916948.1 | 23S rRNA (pseudouridine(1915)-N(3)) | taxID used:135614 OG02653|WP\_012915851.1 | transcription elongation factor Gre | taxID used:135614 OG02656|WP\_012917306.1 | transcription antitermination facto | taxID used:135614 OG02667|WP\_012916520.1 | 3-hydroxyacyl-[acyl-carrier-protein | taxID used:135614 OG02668|WP\_012917380.1 | F0F1 ATP synthase subunit B [Xantho | taxID used:135614 OG00266|WP\_012914644.1 | glycine--tRNA ligase subunit beta [ | taxID used:135614 OG00271|WP\_012915329.1 | DNA helicase RecG [Xanthomonas albi | taxID used:135614 OG02740|WP\_012917644.1 | ribonuclease P protein component [X | taxID used:135614 OG02741|WP\_012916230.1 | 50S ribosomal protein L9 [Xanthomon | taxID used:135614 OG02742|WP\_012916231.1 | 30S ribosomal protein S6 [Xanthomon | taxID used:135614 OG02746|WP\_012915781.1 | ClpXP protease specificity-enhancin | taxID used:135614 OG02748|WP\_012917317.1 | D-tyrosyl-tRNA(Tyr) deacylase [Xant | taxID used:135614 OG02757|WP\_012915075.1 | 50S ribosomal protein L15 [Xanthomo | taxID used:135614 OG02761|WP\_012914851.1 | MULTISPECIES: 50S ribosomal protein | taxID used:135614 OG02771|WP\_045757397.1 | DNA polymerase III subunit chi [Xan | taxID used:135614 OG02777|WP\_012916179.1 | ferric iron uptake transcriptional | taxID used:135614 OG02783|WP\_012916094.1 | nucleoside-diphosphate kinase [Xant | taxID used:135614 OG02787|WP\_012916964.1 | MULTISPECIES: RnfABCDGE type electr | taxID used:135614 OG02788|WP\_012915049.1 | MULTISPECIES: 50S ribosomal protein | taxID used:135614 OG02792|WP\_012915063.1 | MULTISPECIES: 50S ribosomal protein | taxID used:135614 OG02805|WP\_012915524.1 | preprotein translocase subunit SecG | taxID used:135614 OG02807|WP\_012916950.1 | ribosome silencing factor [Xanthomo | taxID used:135614 OG02809|WP\_012917375.1 | F0F1 ATP synthase subunit epsilon [ | taxID used:135614 OG02811|WP\_012915541.1 | 30S ribosome-binding factor RbfA [X | taxID used:135614 OG02813|WP\_012915047.1 | preprotein translocase subunit SecE | taxID used:135614 OG02815|WP\_012915482.1 | 50S ribosomal protein L19 [Xanthomo | taxID used:135614 OG02826|WP\_012915485.1 | RNA-binding S4 domain-containing pr | taxID used:135614 OG02832|WP\_012916180.1 | outer membrane protein assembly fac | taxID used:135614 OG02871|WP\_012915070.1 | MULTISPECIES: 30S ribosomal protein | taxID used:135614 OG02874|WP\_003470632.1 | MULTISPECIES: 30S ribosomal protein | taxID used:135614 OG02883|WP\_012914852.1 | MULTISPECIES: 30S ribosomal protein | taxID used:135614 OG00289|WP\_012915543.1 | polyribonucleotide nucleotidyltrans | taxID used:135614 OG00028|WP\_012917137.1 | phosphoribosylformylglycinamidine s | taxID used:135614 OG02906|WP\_012915079.1 | MULTISPECIES: 50S ribosomal protein | taxID used:135614 OG02932|WP\_012915055.1 | 30S ribosomal protein S12 [Xanthomo | taxID used:135614 OG02949|WP\_012915066.1 | 50S ribosomal protein L14 [Xanthomo | taxID used:135614 OG02959|WP\_012917296.1 | YraN family protein [Xanthomonas al | taxID used:135614 OG00295|WP\_012916962.1 | methionine--tRNA ligase [Xanthomona | taxID used:135614 OG02962|WP\_012915428.1 | DUF423 domain-containing protein [X | taxID used:135614 OG02969|WP\_012916501.1 | arsenate reductase (arsc) family pr | taxID used:135614 OG02981|WP\_010342859.1 | MULTISPECIES: 50S ribosomal protein | taxID used:135614 OG02985|WP\_012915052.1 | 50S ribosomal protein L7/L12 [Xanth | taxID used:135614 OG02990|WP\_010340453.1 | MULTISPECIES: 30S ribosomal protein | taxID used:135614 OG02992|WP\_012915671.1 | cell division protein FtsB [Xanthom | taxID used:135614 OG03005|WP\_012915072.1 | MULTISPECIES: 50S ribosomal protein | taxID used:135614 OG03015|WP\_012916103.1 | integration host factor subunit bet | taxID used:135614 OG03028|WP\_012917351.1 | thioredoxin TrxA, partial [Xanthomo | taxID used:135614 OG03029|WP\_012916416.1 | preprotein translocase subunit YajC | taxID used:135614 OG03067|WP\_003486710.1 | MULTISPECIES: 50S ribosomal protein | taxID used:135614 OG03072|WP\_012915788.1 | MULTISPECIES: monothiol glutaredoxi | taxID used:135614 OG03085|WP\_012915142.1 | nucleoid-associated protein, YbaB/E | taxID used:135614 OG03097|WP\_012915444.1 | MULTISPECIES: 50S ribosomal protein | taxID used:135614 OG03104|WP\_012915067.1 | MULTISPECIES: 50S ribosomal protein | taxID used:135614 OG03111|WP\_010341589.1 | MULTISPECIES: 30S ribosomal protein | taxID used:135614 OG03121|WP\_012915069.1 | 30S ribosomal protein S14 [Xanthomo | taxID used:135614 OG03132|WP\_012915681.1 | ribosome assembly RNA-binding prote | taxID used:135614 OG03140|WP\_012915326.1 | DNA-directed RNA polymerase subunit | taxID used:135614 OG03141|WP\_012915599.1 | integration host factor subunit alp | taxID used:135614 OG03142|WP\_003470672.1 | MULTISPECIES: 50S ribosomal protein | taxID used:135614 OG03172|WP\_012916562.1 | oxidative damage protection protein | taxID used:135614 OG00317|WP\_012916360.1 | excinuclease ABC subunit UvrB [Xant | taxID used:135614 OG03184|WP\_012914877.1 | co-chaperone GroES [Xanthomonas alb | taxID used:135614 OG03187|WP\_012915343.1 | hypothetical protein [Xanthomonas a | taxID used:135614 OG03203|WP\_012915686.1 | RNA chaperone Hfq [Xanthomonas albi | taxID used:135614 OG03212|WP\_012915061.1 | MULTISPECIES: 30S ribosomal protein | taxID used:135614 OG03219|WP\_012914868.1 | DNA-binding transcriptional regulat | taxID used:135614 OG03226|WP\_012915065.1 | 30S ribosomal protein S17 [Xanthomo | taxID used:135614 OG03239|WP\_012915447.1 | 30S ribosomal protein S20 [Xanthomo | taxID used:135614 OG03245|WP\_012917290.1 | cell division protein FtsL [Xanthom | taxID used:135614 OG03258|WP\_012914918.1 | hypothetical protein [Xanthomonas a | taxID used:135614 OG03263|WP\_012915479.1 | 30S ribosomal protein S16 [Xanthomo | taxID used:135614 OG03276|WP\_003467392.1 | MULTISPECIES: 30S ribosomal protein | taxID used:135614 OG03277|WP\_012915445.1 | 50S ribosomal protein L27 [Xanthomo | taxID used:135614 OG03286|WP\_012916313.1 | succinate dehydrogenase assembly fa | taxID used:135614 OG00328|WP\_012917636.1 | ATP-dependent DNA helicase Rep [Xan | taxID used:135614 OG03301|WP\_012916181.1 | RnfH family protein [Xanthomonas al | taxID used:135614 OG03304|WP\_012916835.1 | type B 50S ribosomal protein L31 [X | taxID used:135614 OG03335|WP\_010342733.1 | MULTISPECIES: 50S ribosomal protein | taxID used:135614 OG03342|WP\_012915156.1 | MULTISPECIES: acyl carrier protein | taxID used:135614 OG03359|WP\_002804494.1 | MULTISPECIES: 30S ribosomal protein | taxID used:135614 OG03382|WP\_004425677.1 | MULTISPECIES: translation initiatio | taxID used:135614 OG00338|WP\_012916536.1 | RNA helicase [Xanthomonas albilinea | taxID used:135614 OG03399|WP\_003465342.1 | MULTISPECIES: 30S ribosomal protein | taxID used:135614 OG00345|WP\_012915595.1 | threonine--tRNA ligase [Xanthomonas | taxID used:135614 OG03465|WP\_012915152.1 | 50S ribosomal protein L32 [Xanthomo | taxID used:135614 OG03487|WP\_012915074.1 | 50S ribosomal protein L30 [Xanthomo | taxID used:135614 OG03491|WP\_012915064.1 | MULTISPECIES: 50S ribosomal protein | taxID used:135614 OG00034|WP\_012914590.1 | exodeoxyribonuclease V subunit beta | taxID used:135614 OG03518|WP\_005411637.1 | MULTISPECIES: 50S ribosomal protein | taxID used:135614 OG00366|WP\_012915603.1 | 1-deoxy-D-xylulose-5-phosphate synt | taxID used:135614 OG00368|WP\_012916431.1 | molecular chaperone HtpG [Xanthomon | taxID used:135614 OG00374|WP\_012916175.1 | molecular chaperone DnaK [Xanthomon | taxID used:135614 OG00377|WP\_012914906.1 | tRNA uridine-5-carboxymethylaminome | taxID used:135614 OG00379|WP\_012915666.1 | DNA topoisomerase IV subunit B [Xan | taxID used:135614 OG00384|WP\_045757228.1 | ABC transporter ATPase [Xanthomonas | taxID used:135614 OG00038|WP\_012916524.1 | DNA polymerase III subunit alpha [X | taxID used:135614 OG00391|WP\_012917316.1 | RNA polymerase sigma factor RpoD [X | taxID used:135614 OG00393|WP\_012915613.1 | DNA mismatch repair endonuclease Mu | taxID used:135614 OG00398|WP\_012916915.1 | glutathione ABC transporter ATP-bin | taxID used:135614 OG00399|WP\_012916301.1 | excinuclease ABC subunit UvrC [Xant | taxID used:135614 OG00403|WP\_012917455.1 | dihydroxy-acid dehydratase [Xanthom | taxID used:135614 OG00409|WP\_012915084.1 | translational GTPase TypA [Xanthomo | taxID used:135614 OG00411|WP\_012917363.1 | glutamine--fructose-6-phosphate tra | taxID used:135614 OG00426|WP\_012917607.1 | outer membrane protein assembly fac | taxID used:135614 OG00428|WP\_012917018.1 | elongation factor 4 [Xanthomonas al | taxID used:135614 OG00042|WP\_012916550.1 | transcription-repair coupling facto | taxID used:135614 OG00438|WP\_012915004.1 | glutamine--tRNA ligase/YqeY domain | taxID used:135614 OG00448|WP\_012916909.1 | aspartate--tRNA ligase [Xanthomonas | taxID used:135614 OG00449|WP\_012917643.1 | membrane protein insertase YidC [Xa | taxID used:135614 OG00453|WP\_045757038.1 | transcription termination factor Rh | taxID used:135614 OG00466|WP\_012914966.1 | DNA primase [Xanthomonas albilinean | taxID used:135614 OG00473|WP\_012915853.1 | single-stranded-DNA-specific exonuc | taxID used:135614 OG00047|WP\_045757122.1 | exodeoxyribonuclease V subunit gamm | taxID used:135614 OG00048|WP\_045770128.1 | ribonuclease E [Xanthomonas albilin | taxID used:135614 OG00490|WP\_012917150.1 | proline--tRNA ligase [Xanthomonas a | taxID used:135614 OG00496|WP\_012917350.1 | ATP-dependent RNA helicase RhlB [Xa | taxID used:135614 OG00506|WP\_012915668.1 | CTP synthase [Xanthomonas albilinea | taxID used:135614 OG00507|WP\_012916104.1 | 30S ribosomal protein S1 [Xanthomon | taxID used:135614 OG00508|WP\_045757033.1 | energy-dependent translational thro | taxID used:135614 OG00511|WP\_012917536.1 | ubiquinone biosynthesis regulatory | taxID used:135614 OG00520|WP\_012916178.1 | DNA repair protein RecN [Xanthomona | taxID used:135614 OG00534|WP\_012914878.1 | chaperonin GroEL [Xanthomonas albil | taxID used:135614 OG00538|WP\_012916671.1 | peptide chain release factor 3 [Xan | taxID used:135614 OG00053|WP\_012915850.1 | carbamoyl-phosphate synthase large | taxID used:135614 OG00552|WP\_012915448.1 | murein biosynthesis integral membra | taxID used:135614 OG00560|WP\_012916081.1 | glutamine-hydrolyzing GMP synthase | taxID used:135614 OG00561|WP\_012914866.1 | bifunctional phosphoribosylaminoimi | taxID used:135614 OG00582|WP\_012915820.1 | rRNA pseudouridine synthase [Xantho | taxID used:135614 OG00584|WP\_012917378.1 | F0F1 ATP synthase subunit alpha [Xa | taxID used:135614 OG00590|WP\_012915855.1 | lysine--tRNA ligase [Xanthomonas al | taxID used:135614 OG00609|WP\_012915722.1 | glucose-6-phosphate isomerase [Xant | taxID used:135614 OG00613|WP\_012915539.1 | transcription termination/antitermi | taxID used:135614 OG00620|WP\_012917143.1 | leucyl aminopeptidase [Xanthomonas | taxID used:135614 OG00623|WP\_012916934.1 | ribonuclease G [Xanthomonas albilin | taxID used:135614 OG00625|WP\_012917287.1 | UDP-N-acetylmuramoylalanyl-D-glutam | taxID used:135614 OG00629|WP\_012915107.1 | amidophosphoribosyltransferase [Xan | taxID used:135614 OG00635|WP\_012917288.1 | UDP-N-acetylmuramoyl-L-alanyl-D-glu | taxID used:135614 OG00659|WP\_012916082.1 | IMP dehydrogenase [Xanthomonas albi | taxID used:135614 OG00683|WP\_080928195.1 | polynucleotide adenylyltransferase | taxID used:135614 OG00686|WP\_012917283.1 | UDP-N-acetylmuramate--L-alanine lig | taxID used:135614 OG00692|WP\_012915775.1 | tRNA (N6-isopentenyl adenosine(37)- | taxID used:135614 OG00694|WP\_012916546.1 | glutamate--tRNA ligase [Xanthomonas | taxID used:135614 OG00707|WP\_045756906.1 | cysteine--tRNA ligase [Xanthomonas | taxID used:135614 OG00715|WP\_012917376.1 | F0F1 ATP synthase subunit beta [Xan | taxID used:135614 OG00071|WP\_012917145.1 | valine--tRNA ligase [Xanthomonas al | taxID used:135614 OG00721|WP\_012916694.1 | adenosylmethionine--8-amino-7-oxono | taxID used:135614 OG00733|WP\_012915763.1 | histidine--tRNA ligase [Xanthomonas | taxID used:135614 OG00735|WP\_012916088.1 | ribosome biogenesis GTPase Der [Xan | taxID used:135614 OG00748|WP\_012915462.1 | DNA repair protein RadA [Xanthomona | taxID used:135614 OG00755|WP\_012914937.1 | MFS transporter [Xanthomonas albili | taxID used:135614 OG00761|WP\_012915297.1 | UDP-N-acetylmuramate:L-alanyl-gamma | taxID used:135614 OG00763|WP\_012915076.1 | preprotein translocase subunit SecY | taxID used:135614 OG00767|WP\_012915964.1 | replication-associated recombinatio | taxID used:135614 OG00768|WP\_012915476.1 | signal recognition particle protein | taxID used:135614 OG00769|WP\_012915609.1 | exodeoxyribonuclease VII large subu | taxID used:135614 OG00771|WP\_012916163.1 | adenylosuccinate lyase [Xanthomonas | taxID used:135614 OG00778|WP\_012916517.1 | RIP metalloprotease RseP [Xanthomon | taxID used:135614 OG00077|WP\_012915443.1 | excinuclease ABC subunit UvrA [Xant | taxID used:135614 OG00790|WP\_012917372.1 | UDP-N-acetylglucosamine diphosphory | taxID used:135614 OG00798|WP\_012914566.1 | chromosomal replication initiator p | taxID used:135614 OG00799|WP\_012915274.1 | 3-deoxy-D-manno-octulosonic acid tr | taxID used:135614 OG00822|WP\_045757376.1 | tRNA lysidine(34) synthetase TilS [ | taxID used:135614 OG00836|WP\_012915516.1 | phosphoglucosamine mutase [Xanthomo | taxID used:135614 OG00843|WP\_012916897.1 | Tol-Pal system beta propeller repea | taxID used:135614 OG00850|WP\_012917642.1 | tRNA uridine-5-carboxymethylaminome | taxID used:135614 OG00855|WP\_012915687.1 | GTPase HflX [Xanthomonas albilinean | taxID used:135614 OG00857|WP\_012917285.1 | putative lipid II flippase FtsW [Xa | taxID used:135614 OG00865|WP\_045757036.1 | 16S rRNA (cytosine(967)-C(5))-methy | taxID used:135614 OG00870|WP\_045757286.1 | serine--tRNA ligase [Xanthomonas al | taxID used:135614 OG00874|WP\_012915121.1 | trigger factor [Xanthomonas albilin | taxID used:135614 OG00877|WP\_012915305.1 | glutamate-1-semialdehyde-2,1-aminom | taxID used:135614 OG00881|WP\_012915185.1 | adenylosuccinate synthase [Xanthomo | taxID used:135614 OG00897|WP\_012915123.1 | MULTISPECIES: ATP-dependent Clp pro | taxID used:135614 OG00089|WP\_012915450.1 | isoleucine--tRNA ligase [Xanthomona | taxID used:135614 OG00905|WP\_012914865.1 | phosphoribosylamine--glycine ligase | taxID used:135614 OG00911|WP\_012915670.1 | phosphopyruvate hydratase [Xanthomo | taxID used:135614 OG00915|WP\_012915038.1 | glutamyl-tRNA reductase [Xanthomona | taxID used:135614 OG00920|WP\_012915728.1 | flavodoxin-dependent (E)-4-hydroxy- | taxID used:135614 OG00931|WP\_012915104.1 | bifunctional tetrahydrofolate synth | taxID used:135614 OG00934|WP\_012916616.1 | UDP-N-acetylglucosamine 1-carboxyvi | taxID used:135614 OG00936|WP\_012915370.1 | multifunctional CCA addition/repair | taxID used:135614 OG00093|WP\_012916166.1 | 2-oxoglutarate dehydrogenase E1 com | taxID used:135614 OG00940|WP\_045756739.1 | YggW family oxidoreductase [Xanthom | taxID used:135614 OG00944|WP\_012916560.1 | signal recognition particle-docking | taxID used:135614 OG00948|WP\_012917284.1 | undecaprenyldiphospho-muramoylpenta | taxID used:135614 OG00953|WP\_012915157.1 | beta-ketoacyl-[acyl-carrier-protein | taxID used:135614 OG00955|WP\_012917280.1 | MULTISPECIES: cell division protein | taxID used:135614 OG00965|WP\_045756690.1 | bifunctional phosphopantothenoylcys | taxID used:135614 OG00968|WP\_045756813.1 | phosphoglycerate dehydrogenase [Xan | taxID used:135614 OG00981|WP\_012916093.1 | 23S rRNA (adenine(2503)-C(2))-methy | taxID used:135614 OG00986|WP\_012917257.1 | methionine adenosyltransferase [Xan | taxID used:135614 OG00990|WP\_012917279.1 | cell division protein FtsZ [Xanthom | taxID used:135614 OG00994|WP\_012915953.1 | tRNA 2-thiouridine(34) synthase Mnm | taxID used:135614 OG00099|WP\_049887161.1 | bifunctional [glutamate--ammonia li | taxID used:135614 
OG01024|WP\_012916655.1 | serine hydrolase [Xanthomonas albil | taxID used:135614 OG02598|WP\_012914820.1 | hypothetical protein [Xanthomonas a | taxID used:135614 OG03154|WP\_012917381.1 | MULTISPECIES: F0F1 ATP synthase sub | taxID used:135614 OG00675|WP\_012917627.1 | histidine kinase [Xanthomonas albil | taxID used:135614 OG00791|WP\_012915965.1 | voltage-gated chloride channel prot | taxID used:135614 OG01001|WP\_045757163.1 | peptidase M23 [Xanthomonas albiline | taxID used:135614 OG01004|WP\_045757045.1 | GGDEF domain-containing protein [Xa | taxID used:135614 OG01005|WP\_045756897.1 | hypothetical protein [Xanthomonas a | taxID used:135614 OG01006|WP\_012916668.1 | O-succinylhomoserine (thiol)-lyase | taxID used:135614 OG01007|WP\_012915100.1 | glycine C-acetyltransferase [Xantho | taxID used:135614 OG01008|WP\_012916097.1 | acetyl-CoA C-acyltransferase [Xanth | taxID used:135614 OG01009|WP\_012915931.1 | flagellar rod assembly protein FlgJ | taxID used:135614 OG00100|WP\_012914652.1 | TonB-dependent receptor [Xanthomona | taxID used:135614 OG01010|WP\_012915621.1 | NAD(P)/FAD-dependent oxidoreductase | taxID used:135614 OG01011|WP\_012916167.1 | dihydrolipoyllysine-residue succiny | taxID used:135614 OG01012|WP\_012915425.1 | sensor histidine kinase [Xanthomona | taxID used:135614 OG01013|WP\_012915929.1 | flagellar hook-associated protein F | taxID used:135614 OG01015|WP\_012917295.1 | penicillin-binding protein activato | taxID used:135614 OG01016|WP\_045756859.1 | two-component sensor histidine kina | taxID used:135614 OG01017|WP\_012915091.1 | glycerophosphodiester phosphodieste | taxID used:135614 OG01018|WP\_012917508.1 | 4-hydroxybenzoate 3-monooxygenase [ | taxID used:135614 OG01019|WP\_012914572.1 | tetratricopeptide repeat protein [X | taxID used:135614 OG00101|WP\_012916222.1 | DNA gyrase subunit A [Xanthomonas a | taxID used:135614 OG01021|WP\_012916213.1 | imidazolonepropionase [Xanthomonas | taxID used:135614 OG01022|WP\_012914705.1 | beta-ketoacyl-[acyl-carrier-protein | taxID used:135614 OG01023|WP\_012914583.1 | aspartate/tyrosine/aromatic aminotr | taxID used:135614 OG01025|WP\_012917182.1 | cystathionine gamma-synthase [Xanth | taxID used:135614 OG01027|WP\_012917618.1 | class I SAM-dependent rRNA methyltr | taxID used:135614 OG01028|WP\_012914810.1 | DUF1501 domain-containing protein [ | taxID used:135614 OG01030|WP\_012916994.1 | sensor histidine kinase [Xanthomona | taxID used:135614 OG01031|WP\_012917133.1 | type II secretion system F family p | taxID used:135614 OG01032|WP\_012915951.1 | chemotaxis protein [Xanthomonas alb | taxID used:135614 OG01034|WP\_012916798.1 | hypothetical protein [Xanthomonas a | taxID used:135614 OG01035|WP\_012915715.1 | N-acetylmuramoyl-L-alanine amidase | taxID used:135614 OG01036|WP\_012917501.1 | 3-oxoadipyl-CoA thiolase [Xanthomon | taxID used:135614 OG01037|WP\_012915434.1 | formate-dependent phosphoribosylgly | taxID used:135614 OG01039|WP\_012917224.1 | 2-octaprenyl-6-methoxyphenyl hydrox | taxID used:135614 OG00103|WP\_012915860.1 | aconitate hydratase AcnA [Xanthomon | taxID used:135614 OG01040|WP\_045756686.1 | 8-amino-7-oxononanoate synthase [Xa | taxID used:135614 OG01041|WP\_012915433.1 | aromatic ring-hydroxylating dioxyge | taxID used:135614 OG01042|WP\_012916425.1 | flavoprotein dehydrogenase [Xanthom | taxID used:135614 OG01043|WP\_012915265.1 | porin [Xanthomonas albilineans] [GC | taxID used:135614 OG01044|WP\_012916101.1 | lipopolysaccharide assembly protein | taxID used:135614 OG01047|WP\_045756684.1 | efflux RND transporter periplasmic | taxID used:135614 OG01048|WP\_012915170.1 | 2-methylaconitate cis-trans isomera | taxID used:135614 OG01049|WP\_012915168.1 | 2-methylcitrate synthase [Xanthomon | taxID used:135614 OG00104|WP\_012917275.1 | preprotein translocase subunit SecA | taxID used:135614 OG01050|WP\_012915648.1 | phospholipase [Xanthomonas albiline | taxID used:135614 OG01052|WP\_012915427.1 | polyketide cyclase [Xanthomonas alb | taxID used:135614 OG01056|WP\_080929791.1 | lipid-A-disaccharide synthase [Xant | taxID used:135614 OG01057|WP\_012916492.1 | multidrug transporter [Xanthomonas | taxID used:135614 OG00105|WP\_012914623.1 | glucan 1,4-alpha-glucosidase [Xanth | taxID used:135614 OG01061|WP\_012915362.1 | SAM-dependent methyltransferase [Xa | taxID used:135614 OG01066|WP\_012917474.1 | sodium ABC transporter permease [Xa | taxID used:135614 OG01067|WP\_012916949.1 | molecular chaperone DnaJ [Xanthomon | taxID used:135614 OG01068|WP\_045756698.1 | hypothetical protein [Xanthomonas a | taxID used:135614 OG00106|WP\_012914682.1 | DNA polymerase I [Xanthomonas albil | taxID used:135614 OG01070|WP\_012915958.1 | N-acetyltransferase [Xanthomonas al | taxID used:135614 OG01071|WP\_012914961.1 | heme A synthase [Xanthomonas albili | taxID used:135614 OG01073|WP\_012917223.1 | 2-octaprenyl-3-methyl-6-methoxy-1,4 | taxID used:135614 OG01074|WP\_012915928.1 | flagellin [Xanthomonas albilineans] | taxID used:135614 OG01075|WP\_012914714.1 | isovaleryl-CoA dehydrogenase [Xanth | taxID used:135614 OG01076|WP\_012916516.1 | 1-deoxy-D-xylulose-5-phosphate redu | taxID used:135614 OG01077|WP\_012916200.1 | prephenate dehydratase [Xanthomonas | taxID used:135614 OG01078|WP\_012915810.1 | pyridoxal phosphate-dependent amino | taxID used:135614 OG01079|WP\_012916999.1 | acetyl-CoA C-acyltransferase [Xanth | taxID used:135614 OG00107|WP\_012916799.1 | TonB-dependent receptor [Xanthomona | taxID used:135614 OG01080|WP\_012914614.1 | GGDEF domain-containing protein [Xa | taxID used:135614 OG01082|WP\_012915706.1 | galactonate dehydratase [Xanthomona | taxID used:135614 OG01083|WP\_012916575.1 | polyamine ABC transporter ATP-bindi | taxID used:135614 OG01084|WP\_045756912.1 | tRNA-guanine(34) transglycosylase [ | taxID used:135614 OG01085|WP\_012917590.1 | membrane protein [Xanthomonas albil | taxID used:135614 OG01086|WP\_012915248.1 | MFS transporter [Xanthomonas albili | taxID used:135614 OG01087|WP\_012916260.1 | acyl-CoA dehydrogenase [Xanthomonas | taxID used:135614 OG01088|WP\_045757121.1 | alpha-hydroxy-acid oxidizing protei | taxID used:135614 OG01089|WP\_012915235.1 | membrane protein [Xanthomonas albil | taxID used:135614 OG00108|WP\_012914896.1 | pyruvate dehydrogenase (acetyl-tran | taxID used:135614 OG01090|WP\_012916784.1 | phosphoglycerate kinase [Xanthomona | taxID used:135614 OG01091|WP\_012914822.1 | alpha/beta hydrolase [Xanthomonas a | taxID used:135614 OG01092|WP\_012917219.1 | gfo/Idh/MocA family oxidoreductase | taxID used:135614 OG01093|WP\_012915198.1 | MULTISPECIES: ADP-forming succinate | taxID used:135614 OG01095|WP\_012917413.1 | motility protein MotB [Xanthomonas | taxID used:135614 OG01096|WP\_012916642.1 | twitching motility protein PilT [Xa | taxID used:135614 OG01097|WP\_012917187.1 | twitching motility protein PilT [Xa | taxID used:135614 OG01099|WP\_012915628.1 | efflux RND transporter periplasmic | taxID used:135614 OG00109|WP\_012916702.1 | glycoside hydrolase family 2 protei | taxID used:135614 OG01100|WP\_012916576.1 | polyamine ABC transporter substrate | taxID used:135614 OG01101|WP\_012916174.1 | molecular chaperone DnaJ [Xanthomon | taxID used:135614 OG01102|WP\_012915786.1 | 5-(carboxyamino)imidazole ribonucle | taxID used:135614 OG01103|WP\_012916262.1 | 5-methyltetrahydrofolate--homocyste | taxID used:135614 OG01104|WP\_012917074.1 | acyl-CoA desaturase [Xanthomonas al | taxID used:135614 OG01105|WP\_045757350.1 | efflux RND transporter periplasmic | taxID used:135614 OG01106|WP\_012915334.1 | rod shape-determining protein MreC | taxID used:135614 OG01108|WP\_012916375.1 | glutamate 5-kinase [Xanthomonas alb | taxID used:135614 OG00110|WP\_012916703.1 | glycoside hydrolase family 3 [Xanth | taxID used:135614 OG01110|WP\_012915340.1 | lytic murein transglycosylase B [Xa | taxID used:135614 OG01112|WP\_012915183.1 | FtsH protease activity modulator Hf | taxID used:135614 OG01113|WP\_012917329.1 | LysM peptidoglycan-binding domain-c | taxID used:135614 OG01115|WP\_012917208.1 | 4-hydroxyphenylpyruvate dioxygenase | taxID used:135614 OG01116|WP\_012915894.1 | flagellar biosynthesis protein FlhB | taxID used:135614 OG01117|WP\_012916500.1 | succinyl-diaminopimelate desuccinyl | taxID used:135614 OG01119|WP\_012916773.1 | glycosyltransferase family 1 protei | taxID used:135614 OG01120|WP\_012915849.1 | carbamoyl-phosphate synthase small | taxID used:135614 OG01121|WP\_012915568.1 | NAD(P) transhydrogenase subunit alp | taxID used:135614 OG01122|WP\_012915111.1 | glycosyltransferase family 1 protei | taxID used:135614 OG01123|WP\_012916085.1 | molybdopterin-synthase adenylyltran | taxID used:135614 OG01125|WP\_012915369.1 | N-acetylglucosamine-6-phosphate dea | taxID used:135614 OG01126|WP\_080929656.1 | alpha/beta hydrolase [Xanthomonas a | taxID used:135614 OG01127|WP\_045757411.1 | 3,4-dihydroxy-2-butanone-4-phosphat | taxID used:135614 OG00112|WP\_080931561.1 | bifunctional uridylyltransferase/ur | taxID used:135614 OG01130|WP\_012915245.1 | sterol desaturase family protein [X | taxID used:135614 OG01131|WP\_012915800.1 | homoserine O-acetyltransferase [Xan | taxID used:135614 OG01133|WP\_080929716.1 | RNA polymerase-binding protein DksA | taxID used:135614 OG01135|WP\_012915390.1 | S-(hydroxymethyl)glutathione dehydr | taxID used:135614 OG01136|WP\_012914661.1 | glycerophosphodiester phosphodieste | taxID used:135614 OG01137|WP\_012917320.1 | GTP cyclohydrolase II RibA [Xanthom | taxID used:135614 OG01139|WP\_012914939.1 | anhydro-N-acetylmuramic acid kinase | taxID used:135614 OG00113|WP\_012916263.1 | methionine synthase [Xanthomonas al | taxID used:135614 OG01141|WP\_012916267.1 | ABC transporter permease [Xanthomon | taxID used:135614 OG01142|WP\_012917127.1 | general secretion pathway protein G | taxID used:135614 OG01143|WP\_012916389.1 | alpha/beta hydrolase [Xanthomonas a | taxID used:135614 OG01145|WP\_012917200.1 | PLP-dependent cysteine synthase fam | taxID used:135614 OG01148|WP\_012915582.1 | type III polyketide synthase [Xanth | taxID used:135614 OG01149|WP\_012914826.1 | catalase [Xanthomonas albilineans] | taxID used:135614 OG00114|WP\_012917259.1 | phosphoenolpyruvate carboxylase [Xa | taxID used:135614 OG01150|WP\_012915337.1 | rod shape-determining protein RodA | taxID used:135614 OG01151|WP\_012917504.1 | aromatic ring-hydroxylating dioxyge | taxID used:135614 OG01153|WP\_012915094.1 | ROK family protein [Xanthomonas alb | taxID used:135614 OG01154|WP\_012915531.1 | NADH-quinone oxidoreductase subunit | taxID used:135614 OG01155|WP\_012916975.1 | class I SAM-dependent methyltransfe | taxID used:135614 OG01157|WP\_012916138.1 | class III poly(R)-hydroxyalkanoic a | taxID used:135614 OG01158|WP\_045757284.1 | prephenate dehydrogenase/arogenate | taxID used:135614 OG01159|WP\_012915097.1 | membrane protein [Xanthomonas albil | taxID used:135614 OG01160|WP\_045756703.1 | AI-2E family transporter [Xanthomon | taxID used:135614 OG01161|WP\_012917141.1 | LPS export ABC transporter permease | taxID used:135614 OG01163|WP\_012915506.1 | chorismate synthase [Xanthomonas al | taxID used:135614 OG01164|WP\_012915400.1 | ribosome small subunit-dependent GT | taxID used:135614 OG01165|WP\_012915474.1 | galactose-1-epimerase [Xanthomonas | taxID used:135614 OG01166|WP\_012916688.1 | glycine cleavage system protein T [ | taxID used:135614 OG01167|WP\_012914721.1 | cell division protein ZapE [Xanthom | taxID used:135614 OG01168|WP\_012914568.1 | DNA replication and repair protein | taxID used:135614 OG01169|WP\_012916063.1 | efflux RND transporter periplasmic | taxID used:135614 OG00116|WP\_012916954.1 | leucine--tRNA ligase [Xanthomonas a | taxID used:135614 OG01172|WP\_012916709.1 | exported exo-alpha-sialidase [Xanth | taxID used:135614 OG01173|WP\_045757339.1 | DUF4105 domain-containing protein [ | taxID used:135614 OG01174|WP\_012916458.1 | alanine dehydrogenase [Xanthomonas | taxID used:135614 OG01175|WP\_012916476.1 | phosphate ABC transporter substrate | taxID used:135614 OG01176|WP\_012915271.1 | glycosyl transferase [Xanthomonas a | taxID used:135614 OG01177|WP\_045757019.1 | ABC transporter permease [Xanthomon | taxID used:135614 OG01179|WP\_012915290.1 | HDOD domain-containing protein [Xan | taxID used:135614 OG00117|WP\_012916698.1 | TonB-dependent receptor [Xanthomona | taxID used:135614 OG01184|WP\_012914973.1 | dipeptide epimerase [Xanthomonas al | taxID used:135614 OG01185|WP\_012917629.1 | efflux RND transporter periplasmic | taxID used:135614 OG01186|WP\_012914581.1 | radical SAM protein [Xanthomonas al | taxID used:135614 OG01188|WP\_012916997.1 | Glu/Leu/Phe/Val dehydrogenase [Xant | taxID used:135614 OG01189|WP\_012915284.1 | threonine/serine dehydratase [Xanth | taxID used:135614 OG01191|WP\_012915932.1 | flagellar basal body P-ring protein | taxID used:135614 OG01192|WP\_012916381.1 | acetylornithine deacetylase [Xantho | taxID used:135614 OG01193|WP\_012916667.1 | homoserine dehydrogenase [Xanthomon | taxID used:135614 OG01194|WP\_012916608.1 | DUF2066 domain-containing protein [ | taxID used:135614 OG01195|WP\_012916565.1 | ribonuclease D [Xanthomonas albilin | taxID used:135614 OG01198|WP\_045757364.1 | hypothetical protein [Xanthomonas a | taxID used:135614 OG01199|WP\_012916649.1 | 3-dehydroquinate synthase [Xanthomo | taxID used:135614 OG00119|WP\_012915540.1 | translation initiation factor IF-2 | taxID used:135614 OG01200|WP\_012916201.1 | 3-phosphoserine/phosphohydroxythreo | taxID used:135614 OG01201|WP\_012916700.1 | glycosyl hydrolase [Xanthomonas alb | taxID used:135614 OG01202|WP\_012917310.1 | bifunctional diaminohydroxyphosphor | taxID used:135614 OG01203|WP\_012915105.1 | SPOR domain-containing protein [Xan | taxID used:135614 OG01204|WP\_012915768.1 | histidinol-phosphate transaminase [ | taxID used:135614 OG01205|WP\_012914567.1 | DNA polymerase III subunit beta [Xa | taxID used:135614 OG01206|WP\_012916451.1 | FAD-binding oxidoreductase [Xanthom | taxID used:135614 OG01207|WP\_012917286.1 | phospho-N-acetylmuramoyl-pentapepti | taxID used:135614 OG01208|WP\_012916137.1 | class III poly(R)-hydroxyalkanoic a | taxID used:135614 OG01209|WP\_012915027.1 | branched-chain amino acid aminotran | taxID used:135614 OG01212|WP\_012916320.1 | sn-glycerol-3-phosphate ABC transpo | taxID used:135614 OG01213|WP\_012916981.1 | alkene reductase [Xanthomonas albil | taxID used:135614 OG01214|WP\_012915769.1 | bifunctional histidinol-phosphatase | taxID used:135614 OG01215|WP\_012917075.1 | ferredoxin reductase [Xanthomonas a | taxID used:135614 OG01216|WP\_012915037.1 | peptide chain release factor 1 [Xan | taxID used:135614 OG01217|WP\_045756890.1 | GNAT family N-acetyltransferase [Xa | taxID used:135614 OG01219|WP\_012915345.1 | lipoyl synthase [Xanthomonas albili | taxID used:135614 OG00121|WP\_012914607.1 | glycerol-3-phosphate 1-O-acyltransf | taxID used:135614 OG01221|WP\_012916598.1 | methylamine utilization protein [Xa | taxID used:135614 OG01223|WP\_012917396.1 | 16S rRNA methyltransferase [Xanthom | taxID used:135614 OG01224|WP\_012914880.1 | 3-deoxy-7-phosphoheptulonate syntha | taxID used:135614 OG01225|WP\_012916330.1 | P-type DNA transfer ATPase VirB11 [ | taxID used:135614 OG01226|WP\_012915045.1 | redox-regulated ATPase YchF [Xantho | taxID used:135614 OG01227|WP\_045756718.1 | endolytic transglycosylase MltG [Xa | taxID used:135614 OG01228|WP\_012917142.1 | LPS export ABC transporter permease | taxID used:135614 OG00122|WP\_012917299.1 | sensor histidine kinase KdpD [Xanth | taxID used:135614 OG01230|WP\_012915367.1 | LacI family DNA-binding transcripti | taxID used:135614 OG01232|WP\_012914597.1 | lipoprotein [Xanthomonas albilinean | taxID used:135614 OG01233|WP\_012915019.1 | NAD(P) transhydrogenase subunit alp | taxID used:135614 OG01234|WP\_012917404.1 | alanine racemase [Xanthomonas albil | taxID used:135614 OG01237|WP\_012917522.1 | type I-F CRISPR-associated protein | taxID used:135614 OG01238|WP\_012915674.1 | tRNA pseudouridine(13) synthase Tru | taxID used:135614 OG01239|WP\_012916498.1 | right-handed parallel beta-helix re | taxID used:135614 OG00123|WP\_012915264.1 | hybrid sensor histidine kinase/resp | taxID used:135614 OG01241|WP\_012915446.1 | GTPase ObgE [Xanthomonas albilinean | taxID used:135614 OG01242|WP\_012917167.1 | dTDP-glucose 4,6-dehydratase [Xanth | taxID used:135614 OG01243|WP\_012914609.1 | LacI family transcriptional regulat | taxID used:135614 OG01244|WP\_045756812.1 | peptidase C13 [Xanthomonas albiline | taxID used:135614 OG01245|WP\_012914628.1 | LacI family DNA-binding transcripti | taxID used:135614 OG01246|WP\_012917218.1 | 23S rRNA (cytidine(2498)-2'-O)-meth | taxID used:135614 OG01247|WP\_045757331.1 | sensor domain-containing diguanylat | taxID used:135614 OG01248|WP\_045757211.1 | nitronate monooxygenase [Xanthomona | taxID used:135614 OG01249|WP\_012916654.1 | uroporphyrinogen decarboxylase [Xan | taxID used:135614 OG00124|WP\_012914710.1 | GCN5 family N-acetyltransferase [Xa | taxID used:135614 OG01250|WP\_012917625.1 | two-component sensor histidine kina | taxID used:135614 OG01251|WP\_012917198.1 | DNA polymerase IV [Xanthomonas albi | taxID used:135614 OG01252|WP\_012914725.1 | ribonucleotide-diphosphate reductas | taxID used:135614 OG01253|WP\_012916711.1 | N(4)-(beta-N-acetylglucosaminyl)-L- | taxID used:135614 OG01254|WP\_012915133.1 | lipopolysaccharide heptosyltransfer | taxID used:135614 OG01255|WP\_012916714.1 | WD40 repeat domain-containing prote | taxID used:135614 OG01257|WP\_012915865.1 | chemotaxis response regulator prote | taxID used:135614 OG01259|WP\_012916461.1 | two-component system response regul | taxID used:135614 OG00125|WP\_012915692.1 | alanine--tRNA ligase [Xanthomonas a | taxID used:135614 OG01260|WP\_012914913.1 | biotin synthase BioB [Xanthomonas a | taxID used:135614 OG01261|WP\_012916223.1 | S-methyl-5-thioribose-1-phosphate i | taxID used:135614 OG01263|WP\_012917321.1 | CDP-glycerol glycerophosphotransfer | taxID used:135614 OG01265|WP\_012916750.1 | LacI family DNA-binding transcripti | taxID used:135614 OG01266|WP\_045757315.1 | S-adenosylmethionine:tRNA ribosyltr | taxID used:135614 OG01267|WP\_012916031.1 | syrp, albicidin biosynthesis regula | taxID used:135614 OG01269|WP\_012914843.1 | anthranilate phosphoribosyltransfer | taxID used:135614 OG00126|WP\_012917068.1 | malto-oligosyltrehalose synthase [X | taxID used:135614 OG01270|WP\_012916107.1 | agmatine deiminase family protein [ | taxID used:135614 OG01271|WP\_012914885.1 | glycerophosphoryl diester phosphodi | taxID used:135614 OG01272|WP\_080931547.1 | sulfotransferase family protein [Xa | taxID used:135614 OG01273|WP\_012915101.1 | L-threonine 3-dehydrogenase [Xantho | taxID used:135614 OG01274|WP\_012915917.1 | ketoacyl-ACP synthase III [Xanthomo | taxID used:135614 OG01275|WP\_012917621.1 | cupin-like domain-containing protei | taxID used:135614 OG01276|WP\_045757412.1 | glycosyl transferase [Xanthomonas a | taxID used:135614 OG01277|WP\_012917517.1 | GGDEF domain-containing protein [Xa | taxID used:135614 OG01278|WP\_012916384.1 | acetylornithine carbamoyltransferas | taxID used:135614 OG00127|WP\_012915723.1 | 1,4-beta-D-glucan glucohydrolase [X | taxID used:135614 OG01281|WP\_045756933.1 | phosphoribosylformylglycinamidine c | taxID used:135614 OG01282|WP\_012915963.1 | cation transporter [Xanthomonas alb | taxID used:135614 OG01283|WP\_012914605.1 | alpha-N-arabinofuranosidase [Xantho | taxID used:135614 OG01284|WP\_045756705.1 | aldo/keto reductase [Xanthomonas al | taxID used:135614 OG01285|WP\_012915333.1 | rod shape-determining protein [Xant | taxID used:135614 OG01286|WP\_045757348.1 | hypothetical protein [Xanthomonas a | taxID used:135614 OG01287|WP\_012916831.1 | pilus assembly protein PilM [Xantho | taxID used:135614 OG01288|WP\_012916305.1 | tetraacyldisaccharide 4'-kinase [Xa | taxID used:135614 OG01289|WP\_012916177.1 | heat-inducible transcriptional repr | taxID used:135614 OG00128|WP\_012915864.1 | bifunctional diguanylate cyclase/ph | taxID used:135614 OG01291|WP\_012916643.1 | type IV pili twitching motility pro | taxID used:135614 OG01292|WP\_012915731.1 | UDP-N-acetylmuramate dehydrogenase | taxID used:135614 OG01293|WP\_012915470.1 | ABC transporter permease [Xanthomon | taxID used:135614 OG01295|WP\_012915732.1 | quinone-dependent dihydroorotate de | taxID used:135614 OG01296|WP\_012916604.1 | aminoglycoside phosphotransferase [ | taxID used:135614 OG01297|WP\_012917490.1 | NADP-dependent oxidoreductase [Xant | taxID used:135614 OG01298|WP\_012916036.1 | class I SAM-dependent methyltransfe | taxID used:135614 OG00129|WP\_012915169.1 | Fe/S-dependent 2-methylisocitrate d | taxID used:135614 OG01302|WP\_012915793.1 | sensor histidine kinase [Xanthomona | taxID used:135614 OG01309|WP\_012916669.1 | homoserine O-acetyltransferase [Xan | taxID used:135614 OG01310|WP\_049887219.1 | protein involved in meta-pathway of | taxID used:135614 OG01311|WP\_012915261.1 | LacI family transcriptional regulat | taxID used:135614 OG01312|WP\_045756767.1 | alcohol dehydrogenase AdhP [Xanthom | taxID used:135614 OG01313|WP\_045756910.1 | EF-P beta-lysylation protein EpmB [ | taxID used:135614 OG01314|WP\_012915136.1 | GTP 3',8-cyclase MoaA [Xanthomonas | taxID used:135614 OG01315|WP\_012917454.1 | AI-2E family transporter [Xanthomon | taxID used:135614 OG01317|WP\_012916507.1 | SCPU domain-containing protein [Xan | taxID used:135614 OG01318|WP\_012915096.1 | zinc-binding alcohol dehydrogenase | taxID used:135614 OG01319|WP\_012915690.1 | recombinase RecA [Xanthomonas albil | taxID used:135614 OG01322|WP\_012917543.1 | 3-oxoacyl-ACP synthase III [Xanthom | taxID used:135614 OG01324|WP\_012915508.1 | aspartate-semialdehyde dehydrogenas | taxID used:135614 OG01325|WP\_012915507.1 | D-glycerate dehydrogenase [Xanthomo | taxID used:135614 OG01326|WP\_012915801.1 | tetratricopeptide repeat protein [X | taxID used:135614 OG01327|WP\_012916239.1 | hypothetical protein [Xanthomonas a | taxID used:135614 OG01328|WP\_012914806.1 | tRNA dihydrouridine(20/20a) synthas | taxID used:135614 OG01329|WP\_012914687.1 | cytochrome d ubiquinol oxidase subu | taxID used:135614 OG00132|WP\_045756766.1 | diaminopimelate decarboxylase [Xant | taxID used:135614 OG01330|WP\_012917614.1 | patatin [Xanthomonas albilineans] [ | taxID used:135614 OG01331|WP\_045756985.1 | Holliday junction branch migration | taxID used:135614 OG01332|WP\_045756637.1 | sugar kinase [Xanthomonas albilinea | taxID used:135614 OG01333|WP\_012914953.1 | cytochrome c oxidase subunit II [Xa | taxID used:135614 OG01334|WP\_012917616.1 | TIGR00266 family protein [Xanthomon | taxID used:135614 OG01335|WP\_012916952.1 | DNA polymerase III subunit delta [X | taxID used:135614 OG01337|WP\_012917538.1 | 3-beta hydroxysteroid dehydrogenase | taxID used:135614 OG01338|WP\_012915900.1 | flagellar motor switch protein FliM | taxID used:135614 OG01339|WP\_012917560.1 | PAS domain-containing sensor histid | taxID used:135614 OG00133|WP\_012915863.1 | bifunctional aconitate hydratase 2/ | taxID used:135614 OG01340|WP\_012916519.1 | UDP-3-O-(3-hydroxymyristoyl)glucosa | taxID used:135614 OG01342|WP\_012917473.1 | energy transducer TonB [Xanthomonas | taxID used:135614 OG01343|WP\_012915471.1 | sugar ABC transporter permease YjfF | taxID used:135614 OG01344|WP\_045756938.1 | glycosyltransferase [Xanthomonas al | taxID used:135614 OG01345|WP\_012916475.1 | phosphate ABC transporter substrate | taxID used:135614 OG01347|WP\_012915368.1 | SIS domain-containing protein [Xant | taxID used:135614 OG01348|WP\_012916697.1 | glucokinase [Xanthomonas albilinean | taxID used:135614 OG01349|WP\_012916898.1 | cell envelope integrity protein Tol | taxID used:135614 OG01351|WP\_012914601.1 | beta-1,4-xylanase [Xanthomonas albi | taxID used:135614 OG01352|WP\_012917548.1 | glycerol-3-phosphate dehydrogenase | taxID used:135614 OG01353|WP\_012916780.1 | fructose-bisphosphate aldolase clas | taxID used:135614 OG01354|WP\_012916826.1 | MoxR family ATPase [Xanthomonas alb | taxID used:135614 OG01355|WP\_012917567.1 | alpha/beta hydrolase [Xanthomonas a | taxID used:135614 OG01356|WP\_012916322.1 | glucokinase [Xanthomonas albilinean | taxID used:135614 OG01357|WP\_012917255.1 | tRNA dihydrouridine synthase DusB [ | taxID used:135614 OG00135|WP\_012916696.1 | TonB-dependent receptor [Xanthomona | taxID used:135614 OG01360|WP\_012915287.1 | ketol-acid reductoisomerase [Xantho | taxID used:135614 OG01361|WP\_012915597.1 | phenylalanine--tRNA ligase subunit | taxID used:135614 OG01362|WP\_045757209.1 | fumarylacetoacetate hydrolase [Xant | taxID used:135614 OG01364|WP\_012917008.1 | beta-N-acetylhexosaminidase [Xantho | taxID used:135614 OG01365|WP\_012917521.1 | type I-F CRISPR-associated protein | taxID used:135614 OG01366|WP\_012916785.1 | type I glyceraldehyde-3-phosphate d | taxID used:135614 OG01367|WP\_012917478.1 | ABC transporter ATP-binding protein | taxID used:135614 OG01368|WP\_012915172.1 | inosine-uridine preferring nucleosi | taxID used:135614 OG01369|WP\_012917467.1 | porphobilinogen synthase [Xanthomon | taxID used:135614 OG01370|WP\_045756913.1 | hypothetical protein [Xanthomonas a | taxID used:135614 OG01371|WP\_012915223.1 | 23S rRNA pseudouridine(1911/1915/19 | taxID used:135614 OG01372|WP\_012916571.1 | magnesium and cobalt transport prot | taxID used:135614 OG01374|WP\_012917506.1 | oxidoreductase [Xanthomonas albilin | taxID used:135614 OG01375|WP\_012916158.1 | hypothetical protein [Xanthomonas a | taxID used:135614 OG01376|WP\_012917427.1 | aldo/keto reductase [Xanthomonas al | taxID used:135614 OG01377|WP\_012915119.1 | NAD-dependent isocitrate dehydrogen | taxID used:135614 OG01379|WP\_045757416.1 | methionine ABC transporter ATP-bind | taxID used:135614 OG00137|WP\_045757056.1 | ATP-dependent helicase HrpB [Xantho | taxID used:135614 OG01380|WP\_045756720.1 | quinone oxidoreductase [Xanthomonas | taxID used:135614 OG01381|WP\_012916225.1 | EF-P lysine aminoacylase GenX [Xant | taxID used:135614 OG01384|WP\_080931583.1 | GGDEF domain-containing protein [Xa | taxID used:135614 OG01385|WP\_012917040.1 | alpha/beta hydrolase [Xanthomonas a | taxID used:135614 OG01386|WP\_012915564.1 | octaprenyl-diphosphate synthase [Xa | taxID used:135614 OG01387|WP\_012915078.1 | DNA-directed RNA polymerase subunit | taxID used:135614 OG01388|WP\_012915449.1 | bifunctional riboflavin kinase/FAD | taxID used:135614 OG01389|WP\_012915694.1 | membrane protein [Xanthomonas albil | taxID used:135614 OG01390|WP\_045757241.1 | PhoH family protein [Xanthomonas al | taxID used:135614 OG01391|WP\_012916333.1 | conjugative transfer protein [Xanth | taxID used:135614 OG01392|WP\_012916100.1 | LPS biosynthesis protein [Xanthomon | taxID used:135614 OG01393|WP\_012917518.1 | type I-F CRISPR-associated endonucl | taxID used:135614 OG01394|WP\_049887211.1 | hypothetical protein [Xanthomonas a | taxID used:135614 OG01395|WP\_045756922.1 | 2,3,4,5-tetrahydropyridine-2,6-dica | taxID used:135614 OG01396|WP\_012915217.1 | L-histidine N(alpha)-methyltransfer | taxID used:135614 OG01397|WP\_012917189.1 | fumarylacetoacetate hydrolase [Xant | taxID used:135614 OG01398|WP\_012916117.1 | RluA family pseudouridine synthase | taxID used:135614 OG01399|WP\_012916202.1 | protein-methionine-sulfoxide reduct | taxID used:135614 OG00139|WP\_012916712.1 | DNA mismatch repair protein MutS [X | taxID used:135614 OG00013|WP\_045756888.1 | glutamate dehydrogenase [Xanthomona | taxID used:135614 OG01401|WP\_012915086.1 | malate dehydrogenase [Xanthomonas a | taxID used:135614 OG01402|WP\_012917024.1 | putative sulfate exporter family tr | taxID used:135614 OG01403|WP\_012914696.1 | acyltransferase [Xanthomonas albili | taxID used:135614 OG01404|WP\_012916618.1 | KpsF/GutQ family sugar-phosphate is | taxID used:135614 OG01408|WP\_045756697.1 | bile acid:sodium symporter [Xanthom | taxID used:135614 OG01409|WP\_012914671.1 | nucleoside-diphosphate sugar epimer | taxID used:135614 OG00140|WP\_012915240.1 | ATP-dependent chaperone ClpB [Xanth | taxID used:135614 OG01410|WP\_012914634.1 | ferrochelatase [Xanthomonas albilin | taxID used:135614 OG01411|WP\_012916378.1 | N-acetyl-gamma-glutamyl-phosphate r | taxID used:135614 OG01412|WP\_012916995.1 | LysR family transcriptional regulat | taxID used:135614 OG01413|WP\_012917641.1 | hypothetical protein [Xanthomonas a | taxID used:135614 OG01414|WP\_012917058.1 | YafY family transcriptional regulat | taxID used:135614 OG01415|WP\_012915906.1 | flagellar motor switch protein FliG | taxID used:135614 OG01416|WP\_045756822.1 | thioredoxin-disulfide reductase [Xa | taxID used:135614 OG01417|WP\_012915153.1 | ketoacyl-ACP synthase III [Xanthomo | taxID used:135614 OG01418|WP\_012915466.1 | LysR family transcriptional regulat | taxID used:135614 OG01419|WP\_045756756.1 | LacI family transcriptional regulat | taxID used:135614 OG01420|WP\_045756680.1 | nucleoside-diphosphate sugar epimer | taxID used:135614 OG01421|WP\_012917139.1 | site-specific tyrosine recombinase | taxID used:135614 OG01422|WP\_012914947.1 | 3-phosphoglycerate dehydrogenase [X | taxID used:135614 OG01423|WP\_012917020.1 | hypothetical protein [Xanthomonas a | taxID used:135614 OG01424|WP\_012916574.1 | putrescine/spermidine ABC transport | taxID used:135614 OG01425|WP\_012917049.1 | tyrosine recombinase XerC [Xanthomo | taxID used:135614 OG01426|WP\_045757433.1 | uroporphyrin-III methyltransferase | taxID used:135614 OG01431|WP\_012916690.1 | SPFH/Band 7/PHB domain protein [Xan | taxID used:135614 OG01433|WP\_012915273.1 | lipid A biosynthesis lauroyl acyltr | taxID used:135614 OG01434|WP\_012917247.1 | hypothetical protein [Xanthomonas a | taxID used:135614 OG01435|WP\_012917305.1 | thiamine-phosphate kinase [Xanthomo | taxID used:135614 OG01436|WP\_012916477.1 | phosphate ABC transporter permease | taxID used:135614 OG01437|WP\_012916713.1 | GTP-binding protein [Xanthomonas al | taxID used:135614 OG01439|WP\_012916023.1 | hypothetical protein [Xanthomonas a | taxID used:135614 OG00143|WP\_012916977.1 | bifunctional lysylphosphatidylglyce | taxID used:135614 OG01441|WP\_012917273.1 | DNA mismatch repair protein MutT [X | taxID used:135614 OG01444|WP\_012915456.1 | ubiquinol oxidase subunit II [Xanth | taxID used:135614 OG01445|WP\_012915008.1 | transaldolase [Xanthomonas albiline | taxID used:135614 OG01446|WP\_012916414.1 | protein translocase subunit SecF [X | taxID used:135614 OG01447|WP\_012915013.1 | prolyl aminopeptidase [Xanthomonas | taxID used:135614 OG01450|WP\_012917262.1 | sulfotransferase [Xanthomonas albil | taxID used:135614 OG01451|WP\_012917233.1 | 4-hydroxythreonine-4-phosphate dehy | taxID used:135614 OG01452|WP\_012915332.1 | carbohydrate kinase family protein | taxID used:135614 OG01455|WP\_012916255.1 | 5'-nucleotidase [Xanthomonas albili | taxID used:135614 OG01457|WP\_012916525.1 | acetyl-CoA carboxylase carboxyltran | taxID used:135614 OG01458|WP\_012915161.1 | DNA polymerase III subunit delta' [ | taxID used:135614 OG01459|WP\_045756918.1 | DUF1684 domain-containing protein [ | taxID used:135614 OG00145|WP\_045757054.1 | iron-uptake factor [Xanthomonas alb | taxID used:135614 OG01460|WP\_012915885.1 | flagellar motor protein MotD [Xanth | taxID used:135614 OG01461|WP\_012917617.1 | TerC family protein [Xanthomonas al | taxID used:135614 OG01462|WP\_012916996.1 | cysteine synthase A [Xanthomonas al | taxID used:135614 OG01463|WP\_012915517.1 | isopenicillin N synthase family oxy | taxID used:135614 OG01464|WP\_012916532.1 | 2-keto-3-deoxygluconate transporter | taxID used:135614 OG01465|WP\_012915042.1 | ribose-phosphate pyrophosphokinase | taxID used:135614 OG01467|WP\_012915708.1 | galactose-binding protein [Xanthomo | taxID used:135614 OG01468|WP\_012915452.1 | 4-hydroxy-3-methylbut-2-enyl diphos | taxID used:135614 OG01469|WP\_012915776.1 | DUF4124 domain-containing protein [ | taxID used:135614 OG01470|WP\_045757383.1 | stearoyl-CoA 9-desaturase [Xanthomo | taxID used:135614 OG01471|WP\_012916625.1 | HPr kinase/phosphorylase [Xanthomon | taxID used:135614 OG01473|WP\_012917282.1 | D-alanine--D-alanine ligase [Xantho | taxID used:135614 OG01476|WP\_012917489.1 | aldo/keto reductase [Xanthomonas al | taxID used:135614 OG01478|WP\_012916725.1 | glutathione synthase [Xanthomonas a | taxID used:135614 OG01479|WP\_012915685.1 | tRNA (adenosine(37)-N6)-dimethylall | taxID used:135614 OG01480|WP\_012915009.1 | LysR family transcriptional regulat | taxID used:135614 OG01481|WP\_012917276.1 | membrane protein [Xanthomonas albil | taxID used:135614 OG01483|WP\_012916154.1 | peptidyl-prolyl cis-trans isomerase | taxID used:135614 OG01485|WP\_045757381.1 | homocysteine S-methyltransferase [X | taxID used:135614 OG01486|WP\_012916145.1 | mechanosensitive ion channel family | taxID used:135614 OG01487|WP\_045757049.1 | GAF domain-containing protein [Xant | taxID used:135614 OG01488|WP\_012916452.1 | hydroxyproline-2-epimerase [Xanthom | taxID used:135614 OG01489|WP\_012914819.1 | MBL fold metallo-hydrolase [Xanthom | taxID used:135614 OG00148|WP\_012916701.1 | beta-hexosaminidase [Xanthomonas al | taxID used:135614 OG01490|WP\_012917333.1 | RDD family protein [Xanthomonas alb | taxID used:135614 OG01491|WP\_045756715.1 | MoxR family ATPase [Xanthomonas alb | taxID used:135614 OG01492|WP\_012915705.1 | SMP-30/gluconolactonase/LRE family | taxID used:135614 OG01493|WP\_012915175.1 | glycerophosphodiester phosphodieste | taxID used:135614 OG01494|WP\_045757028.1 | ribokinase [Xanthomonas albilineans | taxID used:135614 OG01495|WP\_012916583.1 | diguanylate cyclase response regula | taxID used:135614 OG01497|WP\_012917480.1 | 2-dehydropantoate 2-reductase [Xant | taxID used:135614 OG01498|WP\_012916196.1 | LysR family transcriptional regulat | taxID used:135614 OG01499|WP\_012914861.1 | NAD(+) diphosphatase [Xanthomonas a | taxID used:135614 OG00149|WP\_012916326.1 | VirB4 family type IV secretion/conj | taxID used:135614 OG01500|WP\_045768259.1 | pilus assembly protein PilW [Xantho | taxID used:135614 OG01501|WP\_012915822.1 | segregation/condensation protein A | taxID used:135614 OG01502|WP\_012915154.1 | [acyl-carrier-protein] S-malonyltra | taxID used:135614 OG01504|WP\_045757152.1 | bifunctional biotin--[acetyl-CoA-ca | taxID used:135614 OG01505|WP\_012917271.1 | peptidase [Xanthomonas albilineans] | taxID used:135614 OG01506|WP\_045757250.1 | chemotaxis protein CheV [Xanthomona | taxID used:135614 OG01507|WP\_012915614.1 | DUF1684 domain-containing protein [ | taxID used:135614 OG01508|WP\_012917542.1 | alpha/beta hydrolase [Xanthomonas a | taxID used:135614 OG01509|WP\_045756747.1 | hypothetical protein [Xanthomonas a | taxID used:135614 OG00150|WP\_012914856.1 | hybrid sensor histidine kinase/resp | taxID used:135614 OG01510|WP\_012916638.1 | aspartate carbamoyltransferase cata | taxID used:135614 OG01511|WP\_012915716.1 | GTP cyclohydrolase I FolE2 [Xanthom | taxID used:135614 OG01512|WP\_045757050.1 | magnesium transporter [Xanthomonas | taxID used:135614 OG01513|WP\_012914643.1 | glycine--tRNA ligase subunit alpha | taxID used:135614 OG01514|WP\_012916529.1 | peptidylprolyl isomerase [Xanthomon | taxID used:135614 OG01515|WP\_045756816.1 | phosphoglycerate mutase [Xanthomona | taxID used:135614 OG01516|WP\_012917348.1 | ABC transporter permease [Xanthomon | taxID used:135614 OG01518|WP\_012915016.1 | exodeoxyribonuclease IX [Xanthomona | taxID used:135614 OG01519|WP\_045757155.1 | nucleoside-diphosphate sugar epimer | taxID used:135614 OG00151|WP\_045756711.1 | S9 family peptidase [Xanthomonas al | taxID used:135614 OG01520|WP\_012914835.1 | phosphoribosylaminoimidazolesuccino | taxID used:135614 OG01522|WP\_012914608.1 | hypothetical protein [Xanthomonas a | taxID used:135614 OG01523|WP\_045757425.1 | AraC family transcriptional regulat | taxID used:135614 OG01524|WP\_012915439.1 | acyl-CoA thioesterase II [Xanthomon | taxID used:135614 OG01526|WP\_012916776.1 | sulfate adenylyltransferase subunit | taxID used:135614 OG01527|WP\_012917236.1 | symmetrical bis(5'-nucleosyl)-tetra | taxID used:135614 OG01528|WP\_012917169.1 | electron transfer flavoprotein subu | taxID used:135614 OG01529|WP\_012915396.1 | monothiol glutaredoxin, Grx4 family | taxID used:135614 OG00152|WP\_012917584.1 | DUF1998 domain-containing protein [ | taxID used:135614 OG01530|WP\_012917318.1 | lipid A biosynthesis lauroyl acyltr | taxID used:135614 OG01533|WP\_012916533.1 | EamA/RhaT family transporter [Xanth | taxID used:135614 OG01535|WP\_012914683.1 | oxygen-dependent coproporphyrinogen | taxID used:135614 OG01536|WP\_045756762.1 | 50S ribosomal protein L3 N(5)-gluta | taxID used:135614 OG01538|WP\_012917214.1 | carbohydrate kinase [Xanthomonas al | taxID used:135614 OG01539|WP\_012917278.1 | UDP-3-O-acyl-N-acetylglucosamine de | taxID used:135614 OG01542|WP\_012917327.1 | methionyl-tRNA formyltransferase [X | taxID used:135614 OG01544|WP\_045757153.1 | EamA family transporter RarD [Xanth | taxID used:135614 OG01545|WP\_012917230.1 | acetoin utilization protein [Xantho | taxID used:135614 OG01546|WP\_012917164.1 | dTDP-4-dehydrorhamnose reductase [X | taxID used:135614 OG01547|WP\_012914635.1 | SH3 domain-containing-like protein | taxID used:135614 OG01548|WP\_012914915.1 | 4-hydroxybenzoate octaprenyltransfe | taxID used:135614 OG00154|WP\_012915093.1 | TonB-dependent receptor [Xanthomona | taxID used:135614 OG01550|WP\_012914962.1 | protoheme IX farnesyltransferase [X | taxID used:135614 OG01551|WP\_012917155.1 | MCE family protein [Xanthomonas alb | taxID used:135614 OG01553|WP\_012915354.1 | 6-phosphogluconate dehydrogenase (d | taxID used:135614 OG01554|WP\_012914870.1 | 50S ribosomal protein L11 methyltra | taxID used:135614 OG01555|WP\_045757218.1 | transcriptional activator feaR [Xan | taxID used:135614 OG01556|WP\_012915843.1 | hydroxymethylglutaryl-CoA lyase [Xa | taxID used:135614 OG01557|WP\_012917425.1 | LysR family transcriptional regulat | taxID used:135614 OG01559|WP\_012915618.1 | tRNA glutamyl-Q(34) synthetase GluQ | taxID used:135614 OG00155|WP\_012917334.1 | DNA topoisomerase I [Xanthomonas al | taxID used:135614 OG01560|WP\_080931584.1 | homoserine kinase [Xanthomonas albi | taxID used:135614 OG01561|WP\_012915542.1 | tRNA pseudouridine(55) synthase Tru | taxID used:135614 OG01563|WP\_012915766.1 | ATP phosphoribosyltransferase [Xant | taxID used:135614 OG01564|WP\_045757370.1 | DUF58 domain-containing protein [Xa | taxID used:135614 OG01565|WP\_012915625.1 | alpha/beta hydrolase [Xanthomonas a | taxID used:135614 OG01566|WP\_012917640.1 | SDR family NAD(P)-dependent oxidore | taxID used:135614 OG01567|WP\_012917220.1 | gluconolactonase [Xanthomonas albil | taxID used:135614 OG01568|WP\_012914957.1 | cytochrome c oxidase subunit 3 [Xan | taxID used:135614 OG01569|WP\_012916215.1 | N-formylglutamate deformylase [Xant | taxID used:135614 OG00156|WP\_080931571.1 | LPS-assembly protein LptD [Xanthomo | taxID used:135614 OG01570|WP\_012914909.1 | malonyl-[acyl-carrier protein] O-me | taxID used:135614 OG01572|WP\_045756699.1 | YihY/virulence factor BrkB family p | taxID used:135614 OG01574|WP\_012916274.1 | hypothetical protein [Xanthomonas a | taxID used:135614 OG01575|WP\_012915821.1 | SMC-Scp complex subunit ScpB [Xanth | taxID used:135614 OG01579|WP\_012914670.1 | ParB/RepB/Spo0J family partition pr | taxID used:135614 OG00157|WP\_012916680.1 | acyl-CoA dehydrogenase [Xanthomonas | taxID used:135614 OG01580|WP\_012916422.1 | MBL fold metallo-hydrolase [Xanthom | taxID used:135614 OG01581|WP\_012915222.1 | outer membrane protein assembly fac | taxID used:135614 OG01582|WP\_012914639.1 | membrane protein [Xanthomonas albil | taxID used:135614 OG01583|WP\_012914631.1 | recombination-associated protein Rd | taxID used:135614 OG01584|WP\_012915704.1 | MFS transporter [Xanthomonas albili | taxID used:135614 OG01586|WP\_012916448.1 | dihydrodipicolinate synthase family | taxID used:135614 OG01587|WP\_045757172.1 | glycerophosphodiester phosphodieste | taxID used:135614 OG01588|WP\_045756741.1 | endonuclease [Xanthomonas albilinea | taxID used:135614 OG01589|WP\_012917464.1 | hypothetical protein [Xanthomonas a | taxID used:135614 OG01590|WP\_045756872.1 | acyltransferase [Xanthomonas albili | taxID used:135614 OG01591|WP\_012917392.1 | EamA/RhaT family transporter [Xanth | taxID used:135614 OG01592|WP\_012917166.1 | glucose-1-phosphate thymidylyltrans | taxID used:135614 OG01593|WP\_012917060.1 | hydroxymethylbilane synthase [Xanth | taxID used:135614 OG01595|WP\_012915701.1 | 4-hydroxy-tetrahydrodipicolinate sy | taxID used:135614 OG01596|WP\_012917239.1 | prolipoprotein diacylglyceryl trans | taxID used:135614 OG01597|WP\_012914613.1 | 5'-nucleotidase, lipoprotein e(P4) | taxID used:135614 OG01598|WP\_012915512.1 | LysR family transcriptional regulat | taxID used:135614 OG01599|WP\_012915684.1 | dihydropteroate synthase [Xanthomon | taxID used:135614 OG00159|WP\_012916226.1 | NAD-dependent DNA ligase LigA [Xant | taxID used:135614 OG01600|WP\_012916971.1 | DUF1365 domain-containing protein [ | taxID used:135614 OG01601|WP\_012916269.1 | HlyD family secretion protein [Xant | taxID used:135614 OG01602|WP\_012916329.1 | lytic transglycosylase domain-conta | taxID used:135614 OG01603|WP\_012917574.1 | AEC family transporter [Xanthomonas | taxID used:135614 OG01605|WP\_012915515.1 | acetyl-CoA carboxylase carboxyltran | taxID used:135614 OG01606|WP\_012917014.1 | GTPase Era [Xanthomonas albilineans | taxID used:135614 OG01608|WP\_045757070.1 | hypothetical protein [Xanthomonas a | taxID used:135614 OG00160|WP\_045756804.1 | bifunctional aspartate kinase/homos | taxID used:135614 OG01610|WP\_012915352.1 | TIGR01777 family protein [Xanthomon | taxID used:135614 OG01611|WP\_012915827.1 | sugar ABC transporter permease [Xan | taxID used:135614 OG01612|WP\_012916676.1 | Hsp33 family molecular chaperone Hs | taxID used:135614 OG01613|WP\_080931549.1 | polysaccharide deacetylase family p | taxID used:135614 OG01614|WP\_012917589.1 | methylglyoxal synthase [Xanthomonas | taxID used:135614 OG01615|WP\_012916626.1 | RNase adapter RapZ [Xanthomonas alb | taxID used:135614 OG01616|WP\_012916913.1 | class A beta-lactamase [Xanthomonas | taxID used:135614 OG01617|WP\_012914862.1 | membrane protein [Xanthomonas albil | taxID used:135614 OG01619|WP\_012914996.1 | amidohydrolase [Xanthomonas albilin | taxID used:135614 OG01620|WP\_012916083.1 | bifunctional methylenetetrahydrofol | taxID used:135614 OG01621|WP\_012916468.1 | sulfurtransferase [Xanthomonas albi | taxID used:135614 OG01623|WP\_012917212.1 | tryptophan 2,3-dioxygenase [Xanthom | taxID used:135614 OG01625|WP\_045756818.1 | enoyl-CoA hydratase [Xanthomonas al | taxID used:135614 OG01626|WP\_012915891.1 | MinD/ParA family protein [Xanthomon | taxID used:135614 OG01627|WP\_012917345.1 | RNA polymerase sigma factor RpoH [X | taxID used:135614 OG01628|WP\_012916726.1 | energy transducer TonB [Xanthomonas | taxID used:135614 OG01629|WP\_012915619.1 | protease HtpX [Xanthomonas albiline | taxID used:135614 OG00162|WP\_012916434.1 | glycoside hydrolase family 127 prot | taxID used:135614 OG01631|WP\_012915192.1 | prepilin peptidase [Xanthomonas alb | taxID used:135614 OG01634|WP\_012915560.1 | PhzF family phenazine biosynthesis | taxID used:135614 OG01636|WP\_012915180.1 | cytochrome c biogenesis protein [Xa | taxID used:135614 OG01638|WP\_012917446.1 | EamA/RhaT family transporter [Xanth | taxID used:135614 OG01639|WP\_012915635.1 | endonuclease/exonuclease/phosphatas | taxID used:135614 OG00163|WP\_012914569.1 | DNA topoisomerase (ATP-hydrolyzing) | taxID used:135614 OG01640|WP\_012916914.1 | LysR family transcriptional regulat | taxID used:135614 OG01642|WP\_012915431.1 | SPFH domain-containing protein [Xan | taxID used:135614 OG01643|WP\_012917593.1 | D-hexose-6-phosphate mutarotase [Xa | taxID used:135614 OG01644|WP\_012914920.1 | polyamine aminopropyltransferase [X | taxID used:135614 OG01645|WP\_012914630.1 | tRNA 2-thiocytidine(32) synthetase | taxID used:135614 OG01646|WP\_012916569.1 | CBS domain-containing protein [Xant | taxID used:135614 OG01647|WP\_012914728.1 | NAD-dependent protein deacetylase [ | taxID used:135614 OG01648|WP\_012915867.1 | chemotaxis protein CheR [Xanthomona | taxID used:135614 OG01649|WP\_012916157.1 | ABC transporter ATP-binding protein | taxID used:135614 OG00164|WP\_012915259.1 | TonB-dependent receptor [Xanthomona | taxID used:135614 OG01650|WP\_012916098.1 | UTP--glucose-1-phosphate uridylyltr | taxID used:135614 OG01651|WP\_012915166.1 | methylisocitrate lyase [Xanthomonas | taxID used:135614 OG01652|WP\_080931560.1 | M23 family peptidase [Xanthomonas a | taxID used:135614 OG01653|WP\_012917185.1 | membrane protein [Xanthomonas albil | taxID used:135614 OG01655|WP\_012916091.1 | DUF4115 domain-containing protein [ | taxID used:135614 OG01656|WP\_012915188.1 | 30S ribosomal protein S6--L-glutama | taxID used:135614 OG01657|WP\_012917377.1 | F0F1 ATP synthase subunit gamma [Xa | taxID used:135614 OG01658|WP\_045756920.1 | DUF692 domain-containing protein [X | taxID used:135614 OG00165|WP\_012914648.1 | glycosyl transferase family 36 [Xan | taxID used:135614 OG01660|WP\_012915783.1 | nicotinate-nucleotide diphosphoryla | taxID used:135614 OG01662|WP\_012914662.1 | peptidylprolyl isomerase [Xanthomon | taxID used:135614 OG01663|WP\_012915199.1 | succinate--CoA ligase subunit alpha | taxID used:135614 OG01664|WP\_012916928.1 | farnesyl-diphosphate synthase [Xant | taxID used:135614 OG01665|WP\_012916478.1 | phosphate ABC transporter, permease | taxID used:135614 OG01666|WP\_012916554.1 | pirin family protein [Xanthomonas a | taxID used:135614 OG01667|WP\_012916511.1 | elongation factor Ts [Xanthomonas a | taxID used:135614 OG01669|WP\_012917051.1 | diaminopimelate epimerase [Xanthomo | taxID used:135614 OG01670|WP\_012917013.1 | DNA repair protein RecO [Xanthomona | taxID used:135614 OG01671|WP\_012914998.1 | FAA hydrolase family protein [Xanth | taxID used:135614 OG01673|WP\_012917281.1 | cell division protein FtsQ [Xanthom | taxID used:135614 OG01675|WP\_012916677.1 | hypothetical protein [Xanthomonas a | taxID used:135614 OG01676|WP\_045756797.1 | EamA/RhaT family transporter [Xanth | taxID used:135614 OG01678|WP\_080931572.1 | IclR family transcriptional regulat | taxID used:135614 OG01679|WP\_012917488.1 | methyltransferase domain-containing | taxID used:135614 OG00167|WP\_012914724.1 | ribonucleoside-diphosphate reductas | taxID used:135614 OG01681|WP\_012915184.1 | protease modulator HflC [Xanthomona | taxID used:135614 OG01683|WP\_012914904.1 | serine protein kinase RIO [Xanthomo | taxID used:135614 OG01684|WP\_012916221.1 | hypothetical protein [Xanthomonas a | taxID used:135614 OG01685|WP\_012916548.1 | 23S rRNA (adenine(2030)-N(6))-methy | taxID used:135614 OG01686|WP\_012916956.1 | thioredoxin [Xanthomonas albilinean | taxID used:135614 OG01688|WP\_049887186.1 | M23 family peptidase [Xanthomonas a | taxID used:135614 OG01689|WP\_012917272.1 | methylenetetrahydrofolate reductase | taxID used:135614 OG00168|WP\_012916731.1 | penicillin-binding protein 1B [Xant | taxID used:135614 OG01690|WP\_012917180.1 | ABC transporter permease [Xanthomon | taxID used:135614 OG01691|WP\_012915653.1 | tRNA threonylcarbamoyladenosine deh | taxID used:135614 OG01692|WP\_012916319.1 | folate-binding protein [Xanthomonas | taxID used:135614 OG01693|WP\_012915520.1 | DUF3034 domain-containing protein [ | taxID used:135614 OG01694|WP\_012915811.1 | amidohydrolase [Xanthomonas albilin | taxID used:135614 OG01695|WP\_045756688.1 | hydrogenase expression protein [Xan | taxID used:135614 OG01696|WP\_012917322.1 | glycosyltransferase family 2 protei | taxID used:135614 OG01698|WP\_012915391.1 | S-formylglutathione hydrolase [Xant | taxID used:135614 OG00169|WP\_012916832.1 | penicillin-binding protein 1A [Xant | taxID used:135614 OG01701|WP\_080928224.1 | glycosyltransferase family 2 protei | taxID used:135614 OG01702|WP\_045756736.1 | hypothetical protein [Xanthomonas a | taxID used:135614 OG01703|WP\_012915503.1 | phosphatidylserine decarboxylase [X | taxID used:135614 OG01704|WP\_012916922.1 | peptidoglycan endopeptidase [Xantho | taxID used:135614 OG01705|WP\_012917412.1 | flagellar motor stator protein MotA | taxID used:135614 OG01706|WP\_012914570.1 | CPBP family intramembrane metallopr | taxID used:135614 OG01707|WP\_012916789.1 | MBL fold metallo-hydrolase [Xanthom | taxID used:135614 OG01708|WP\_012915495.1 | iron-sulfur cluster carrier protein | taxID used:135614 OG01709|WP\_045756709.1 | 4-(cytidine 5'-diphospho)-2-C-methy | taxID used:135614 OG01710|WP\_080929799.1 | class I SAM-dependent methyltransfe | taxID used:135614 OG01711|WP\_012917515.1 | p-hydroxycinnamoyl CoA hydratase/ly | taxID used:135614 OG01712|WP\_012915376.1 | clavaminate synthase [Xanthomonas a | taxID used:135614 OG01713|WP\_080929728.1 | DUF3108 domain-containing protein [ | taxID used:135614 OG01717|WP\_012915324.1 | YicC family protein [Xanthomonas al | taxID used:135614 OG01719|WP\_012915720.1 | pantoate--beta-alanine ligase [Xant | taxID used:135614 OG00171|WP\_012915124.1 | endopeptidase La [Xanthomonas albil | taxID used:135614 OG01720|WP\_080931599.1 | rhomboid family intramembrane serin | taxID used:135614 OG01721|WP\_012915601.1 | peptidase C1 [Xanthomonas albilinea | taxID used:135614 OG01722|WP\_045757051.1 | SAM-dependent methyltransferase [Xa | taxID used:135614 OG01723|WP\_012916573.1 | putrescine/spermidine ABC transport | taxID used:135614 OG01724|WP\_012917128.1 | general secretion pathway protein G | taxID used:135614 OG01726|WP\_045757018.1 | glycosyl transferase [Xanthomonas a | taxID used:135614 OG01727|WP\_045757184.1 | DUF3014 domain-containing protein [ | taxID used:135614 OG01729|WP\_012915669.1 | 3-deoxy-8-phosphooctulonate synthas | taxID used:135614 OG00172|WP\_012916486.1 | ribonuclease R [Xanthomonas albilin | taxID used:135614 OG01730|WP\_012914633.1 | alpha/beta hydrolase [Xanthomonas a | taxID used:135614 OG01732|WP\_045757323.1 | phosphate ABC transporter ATP-bindi | taxID used:135614 OG01733|WP\_012916062.1 | MipA/OmpV family protein [Xanthomon | taxID used:135614 OG01734|WP\_012916631.1 | phospholipase [Xanthomonas albiline | taxID used:135614 OG01736|WP\_012917463.1 | shikimate dehydrogenase [Xanthomona | taxID used:135614 OG01738|WP\_012915060.1 | MULTISPECIES: 50S ribosomal protein | taxID used:135614 OG01739|WP\_045757245.1 | carbohydrate ABC transporter permea | taxID used:135614 OG01741|WP\_012914663.1 | hypothetical protein [Xanthomonas a | taxID used:135614 OG01742|WP\_045757174.1 | DUF455 domain-containing protein [X | taxID used:135614 OG01743|WP\_012916232.1 | phosphopantetheinyl transferase [Xa | taxID used:135614 OG01744|WP\_012916587.1 | formate dehydrogenase accessory sul | taxID used:135614 OG01745|WP\_012915463.1 | lysophospholipase [Xanthomonas albi | taxID used:135614 OG01746|WP\_012915972.1 | heme ABC transporter permease [Xant | taxID used:135614 OG01747|WP\_012915381.1 | cytochrome c4 [Xanthomonas albiline | taxID used:135614 OG01748|WP\_045757280.1 | kinase/pyrophosphorylase [Xanthomon | taxID used:135614 OG01749|WP\_012916270.1 | alpha/beta hydrolase [Xanthomonas a | taxID used:135614 OG00174|WP\_012915228.1 | TonB-dependent receptor [Xanthomona | taxID used:135614 OG01750|WP\_012914975.1 | N-acetylmuramoyl-L-alanine amidase | taxID used:135614 OG01751|WP\_012915379.1 | thiol:disulfide interchange protein | taxID used:135614 OG01752|WP\_012917202.1 | NADPH-dependent 7-cyano-7-deazaguan | taxID used:135614 OG01753|WP\_012916646.1 | pyrroline-5-carboxylate reductase [ | taxID used:135614 OG01754|WP\_012914703.1 | glycosyltransferase family 2 protei | taxID used:135614 OG01755|WP\_012914936.1 | exodeoxyribonuclease III [Xanthomon | taxID used:135614 OG01757|WP\_012914993.1 | aquaporin family protein [Xanthomon | taxID used:135614 OG01758|WP\_012916412.1 | inositol monophosphatase [Xanthomon | taxID used:135614 OG00175|WP\_045756645.1 | S9 family peptidase [Xanthomonas al | taxID used:135614 OG01760|WP\_012917479.1 | ABC transporter permease [Xanthomon | taxID used:135614 OG01762|WP\_012915012.1 | peptide chain release factor N(5)-g | taxID used:135614 OG01763|WP\_012917443.1 | cyclohexadienyl dehydratase precurs | taxID used:135614 OG01764|WP\_012916159.1 | hypothetical protein [Xanthomonas a | taxID used:135614 OG01767|WP\_045757366.1 | endonuclease [Xanthomonas albilinea | taxID used:135614 OG01769|WP\_045756752.1 | D-alanyl-D-alanine carboxypeptidase | taxID used:135614 OG00176|WP\_012916518.1 | outer membrane protein assembly fac | taxID used:135614 OG01770|WP\_012915655.1 | glycine zipper 2TM domain-containin | taxID used:135614 OG01771|WP\_045756789.1 | LysM peptidoglycan-binding domain-c | taxID used:135614 OG01773|WP\_012917632.1 | bifunctional DNA-formamidopyrimidin | taxID used:135614 OG01774|WP\_080929686.1 | DUF3298 domain-containing protein [ | taxID used:135614 OG01775|WP\_012914571.1 | M48 family peptidase [Xanthomonas a | taxID used:135614 OG01777|WP\_012915897.1 | flagellar biosynthetic protein FliP | taxID used:135614 OG01778|WP\_012917256.1 | UDP-2,3-diacylglucosamine diphospha | taxID used:135614 OG01779|WP\_012915099.1 | rRNA pseudouridine synthase [Xantho | taxID used:135614 OG00177|WP\_012914649.1 | ATPase [Xanthomonas albilineans] [G | taxID used:135614 OG01780|WP\_045757168.1 | HDOD domain-containing protein [Xan | taxID used:135614 OG01781|WP\_012914928.1 | SPOR domain-containing protein [Xan | taxID used:135614 OG01782|WP\_012916092.1 | type IV pilus biogenesis/stability | taxID used:135614 OG01783|WP\_012916515.1 | phosphatidate cytidylyltransferase | taxID used:135614 OG01784|WP\_012917017.1 | signal peptidase I [Xanthomonas alb | taxID used:135614 OG01785|WP\_012915695.1 | bifunctional hydroxymethylpyrimidin | taxID used:135614 OG01786|WP\_012917347.1 | response regulator [Xanthomonas alb | taxID used:135614 OG01787|WP\_012917568.1 | 1-acyl-sn-glycerol-3-phosphate acyl | taxID used:135614 OG01788|WP\_012917238.1 | thymidylate synthase [Xanthomonas a | taxID used:135614 OG01789|WP\_012916314.1 | succinate dehydrogenase iron-sulfur | taxID used:135614 OG01790|WP\_012917382.1 | F0F1 ATP synthase subunit A [Xantho | taxID used:135614 OG01791|WP\_012914848.1 | adenosylmethionine decarboxylase [X | taxID used:135614 OG01792|WP\_012916469.1 | CoA pyrophosphatase [Xanthomonas al | taxID used:135614 OG01794|WP\_045756880.1 | hypothetical protein [Xanthomonas a | taxID used:135614 OG01795|WP\_012915719.1 | 3-methyl-2-oxobutanoate hydroxymeth | taxID used:135614 OG01797|WP\_012917294.1 | 16S rRNA (cytidine(1402)-2'-O)-meth | taxID used:135614 OG01798|WP\_080931598.1 | tol-pal system protein YbgF [Xantho | taxID used:135614 OG01799|WP\_012916692.1 | 3'(2'),5'-bisphosphate nucleotidase | taxID used:135614 OG00179|WP\_012917246.1 | oxidoreductase [Xanthomonas albilin | taxID used:135614 OG01800|WP\_012916974.1 | DUF1295 domain-containing protein [ | taxID used:135614 OG01801|WP\_012917138.1 | hypothetical protein [Xanthomonas a | taxID used:135614 OG01802|WP\_045756671.1 | hypothetical protein [Xanthomonas a | taxID used:135614 OG01803|WP\_012917481.1 | preprotein translocase subunit TatD | taxID used:135614 OG01804|WP\_012916965.1 | hypothetical protein [Xanthomonas a | taxID used:135614 OG01805|WP\_012916058.1 | IS5/IS1182 family transposase [Xant | taxID used:135614 OG01806|WP\_045757030.1 | hypothetical protein [Xanthomonas a | taxID used:135614 OG01807|WP\_045756883.1 | hypothetical protein [Xanthomonas a | taxID used:135614 OG01808|WP\_012915417.1 | phosphatase PAP2 family protein [Xa | taxID used:135614 OG00180|WP\_012916916.1 | TonB-dependent receptor [Xanthomona | taxID used:135614 OG01810|WP\_045757400.1 | ABC transporter ATP-binding protein | taxID used:135614 OG01811|WP\_012917126.1 | general secretion pathway protein G | taxID used:135614 OG01812|WP\_012916510.1 | 30S ribosomal protein S2 [Xanthomon | taxID used:135614 OG01813|WP\_080931568.1 | nicotinate-nucleotide adenylyltrans | taxID used:135614 OG01814|WP\_012916186.1 | KR domain-containing protein [Xanth | taxID used:135614 OG01815|WP\_012916256.1 | NAD kinase [Xanthomonas albilineans | taxID used:135614 OG01816|WP\_012915478.1 | hypothetical protein [Xanthomonas a | taxID used:135614 OG01817|WP\_012917389.1 | MetQ/NlpA family ABC transporter su | taxID used:135614 OG01819|WP\_012915014.1 | N-formylglutamate amidohydrolase [X | taxID used:135614 OG00181|WP\_012917254.1 | carbohydrate-binding protein [Xanth | taxID used:135614 OG01820|WP\_012916985.1 | EAL domain-containing protein [Xant | taxID used:135614 OG01822|WP\_012915422.1 | septum site-determining protein Min | taxID used:135614 OG01823|WP\_012917424.1 | dioxygenase [Xanthomonas albilinean | taxID used:135614 OG01825|WP\_012916968.1 | hypothetical protein [Xanthomonas a | taxID used:135614 OG01827|WP\_012915522.1 | SDR family NAD(P)-dependent oxidore | taxID used:135614 OG01828|WP\_012915481.1 | tRNA (guanosine(37)-N1)-methyltrans | taxID used:135614 OG01829|WP\_012915871.1 | pilus assembly protein PilZ [Xantho | taxID used:135614 OG01830|WP\_045756755.1 | SDR family NAD(P)-dependent oxidore | taxID used:135614 OG01831|WP\_012917497.1 | 3-oxoadipate enol-lactonase [Xantho | taxID used:135614 OG01832|WP\_012916521.1 | acyl-ACP--UDP-N-acetylglucosamine O | taxID used:135614 OG01833|WP\_012917234.1 | 16S rRNA (adenine(1518)-N(6)/adenin | taxID used:135614 OG01834|WP\_012916504.1 | type I methionyl aminopeptidase [Xa | taxID used:135614 OG01835|WP\_012914815.1 | hypothetical protein [Xanthomonas a | taxID used:135614 OG01837|WP\_012915127.1 | hydroxyacylglutathione hydrolase [X | taxID used:135614 OG01838|WP\_045756941.1 | nucleoside triphosphate pyrophospho | taxID used:135614 OG01839|WP\_012915733.1 | methyltransferase domain-containing | taxID used:135614 OG00183|WP\_045756738.1 | DUF1631 domain-containing protein [ | taxID used:135614 OG01840|WP\_012914664.1 | exodeoxyribonuclease III [Xanthomon | taxID used:135614 OG01841|WP\_045756782.1 | ferredoxin--NADP reductase [Xanthom | taxID used:135614 OG01842|WP\_012914669.1 | ParA family protein [Xanthomonas al | taxID used:135614 OG01843|WP\_012915510.1 | tRNA pseudouridine(38-40) synthase | taxID used:135614 OG01844|WP\_012915895.1 | flagellar biosynthetic protein FliR | taxID used:135614 OG01845|WP\_012915436.1 | arginyltransferase [Xanthomonas alb | taxID used:135614 OG01846|WP\_012915654.1 | TatD family deoxyribonuclease [Xant | taxID used:135614 OG01847|WP\_080931579.1 | 1-acyl-sn-glycerol-3-phosphate acyl | taxID used:135614 OG01848|WP\_045757215.1 | tryptophan synthase subunit alpha [ | taxID used:135614 OG00184|WP\_012916096.1 | 3-hydroxyacyl-CoA dehydrogenase [Xa | taxID used:135614 OG01850|WP\_012917059.1 | DUF481 domain-containing protein [X | taxID used:135614 OG01851|WP\_012917564.1 | undecaprenyl-diphosphate phosphatas | taxID used:135614 OG01852|WP\_012914844.1 | indole-3-glycerol phosphate synthas | taxID used:135614 OG01853|WP\_045757222.1 | enoyl-CoA hydratase [Xanthomonas al | taxID used:135614 OG01854|WP\_012915934.1 | flagellar basal-body rod protein Fl | taxID used:135614 OG01855|WP\_080929807.1 | ABC transporter ATP-binding protein | taxID used:135614 OG01856|WP\_045756758.1 | membrane protein [Xanthomonas albil | taxID used:135614 OG01857|WP\_012916743.1 | thiazole synthase [Xanthomonas albi | taxID used:135614 OG01859|WP\_012915884.1 | ParA family protein [Xanthomonas al | taxID used:135614 OG00185|WP\_012916586.1 | formate dehydrogenase a chain prote | taxID used:135614 OG01860|WP\_012914641.1 | molybdenum ABC transporter substrat | taxID used:135614 OG01861|WP\_012914632.1 | M48 family peptidase [Xanthomonas a | taxID used:135614 OG01862|WP\_012915676.1 | 5'/3'-nucleotidase SurE [Xanthomona | taxID used:135614 OG01863|WP\_012916304.1 | 3-deoxy-manno-octulosonate cytidyly | taxID used:135614 OG01864|WP\_012916240.1 | hypothetical protein [Xanthomonas a | taxID used:135614 OG01868|WP\_012915772.1 | imidazole glycerol phosphate syntha | taxID used:135614 OG00186|WP\_012916705.1 | alpha-1,2-mannosidase [Xanthomonas | taxID used:135614 OG01870|WP\_012917148.1 | CDP-diacylglycerol--serine O-phosph | taxID used:135614 OG01871|WP\_012916958.1 | membrane protein [Xanthomonas albil | taxID used:135614 OG01872|WP\_012914711.1 | hypothetical protein [Xanthomonas a | taxID used:135614 OG01873|WP\_012914592.1 | ABC transporter ATP-binding protein | taxID used:135614 OG01874|WP\_012916744.1 | tRNA (guanosine(46)-N7)-methyltrans | taxID used:135614 OG01875|WP\_012916332.1 | hypothetical protein [Xanthomonas a | taxID used:135614 OG01876|WP\_012915135.1 | MBL fold metallo-hydrolase [Xanthom | taxID used:135614 OG01877|WP\_012915874.1 | transporter [Xanthomonas albilinean | taxID used:135614 OG01878|WP\_012915440.1 | enoyl-CoA hydratase/isomerase famil | taxID used:135614 OG01879|WP\_012915844.1 | enoyl-CoA hydratase [Xanthomonas al | taxID used:135614 OG00187|WP\_012916144.1 | phosphoenolpyruvate synthase [Xanth | taxID used:135614 OG01880|WP\_012915224.1 | peptidoglycan editing factor PgeF [ | taxID used:135614 OG01882|WP\_045757068.1 | uroporphyrinogen III methyltransfer | taxID used:135614 OG01883|WP\_012914910.1 | 3-oxoacyl-ACP reductase [Xanthomona | taxID used:135614 OG01884|WP\_012915378.1 | endonuclease [Xanthomonas albilinea | taxID used:135614 OG01885|WP\_012915915.1 | SDR family NAD(P)-dependent oxidore | taxID used:135614 OG01886|WP\_012917261.1 | class I SAM-dependent methyltransfe | taxID used:135614 OG01887|WP\_012917338.1 | short-chain dehydrogenase/reductase | taxID used:135614 OG01889|WP\_012915807.1 | heme ABC transporter permease [Xant | taxID used:135614 OG00188|WP\_012916440.1 | hypothetical protein [Xanthomonas a | taxID used:135614 OG01891|WP\_012916900.1 | protein TolQ [Xanthomonas albilinea | taxID used:135614 OG01893|WP\_012914704.1 | hypothetical protein [Xanthomonas a | taxID used:135614 OG01894|WP\_012916790.1 | molybdate ABC transporter substrate | taxID used:135614 OG01896|WP\_012916471.1 | enoyl-CoA hydratase [Xanthomonas al | taxID used:135614 OG01897|WP\_012916578.1 | gamma-glutamyl-gamma-aminobutyrate | taxID used:135614 OG01898|WP\_045757392.1 | bifunctional demethylmenaquinone me | taxID used:135614 OG01899|WP\_012917634.1 | sel1 repeat family protein [Xanthom | taxID used:135614 OG00189|WP\_045756917.1 | S9 family peptidase [Xanthomonas al | taxID used:135614 OG00018|WP\_012917587.1 | glutamate synthase large subunit [X | taxID used:135614 OG01900|WP\_012917466.1 | DNA/RNA non-specific endonuclease [ | taxID used:135614 OG01901|WP\_012915842.1 | 3-hydroxy-2-methylbutyryl-CoA dehyd | taxID used:135614 OG01903|WP\_012914574.1 | MotA/TolQ/ExbB proton channel famil | taxID used:135614 OG01904|WP\_045756889.1 | ABC transporter ATP-binding protein | taxID used:135614 OG01905|WP\_045756763.1 | NADH-quinone oxidoreductase subunit | taxID used:135614 OG01906|WP\_045757175.1 | membrane protein [Xanthomonas albil | taxID used:135614 OG01907|WP\_045757456.1 | membrane protein [Xanthomonas albil | taxID used:135614 OG01908|WP\_012915106.1 | colicin V biosynthesis protein [Xan | taxID used:135614 OG01909|WP\_012916830.1 | fimbrial protein [Xanthomonas albil | taxID used:135614 OG00190|WP\_012916436.1 | S9 family peptidase [Xanthomonas al | taxID used:135614 OG01910|WP\_012915002.1 | IclR family transcriptional regulat | taxID used:135614 OG01912|WP\_045756743.1 | Expansin-YoaJ [Xanthomonas albiline | taxID used:135614 OG01913|WP\_012916772.1 | polysaccharide deacetylase family p | taxID used:135614 OG01914|WP\_012915779.1 | cytochrome c1 [Xanthomonas albiline | taxID used:135614 OG01916|WP\_012914911.1 | pimeloyl-[acyl-carrier protein] met | taxID used:135614 OG01917|WP\_012916551.1 | 2,3-diphosphoglycerate-dependent ph | taxID used:135614 OG01918|WP\_012914927.1 | malonic semialdehyde reductase [Xan | taxID used:135614 OG01919|WP\_012915552.1 | Fe-S cluster assembly ATPase SufC [ | taxID used:135614 OG01920|WP\_012914578.1 | pyridoxine 5'-phosphate synthase [X | taxID used:135614 OG01921|WP\_012917620.1 | peptide ABC transporter permease [X | taxID used:135614 OG01922|WP\_012914959.1 | SURF1 family protein [Xanthomonas a | taxID used:135614 OG01924|WP\_012915365.1 | GntR family transcriptional regulat | taxID used:135614 OG01925|WP\_012915583.1 | enoyl-CoA hydratase [Xanthomonas al | taxID used:135614 OG01926|WP\_012915134.1 | 3-deoxy-D-manno-octulosonic acid ki | taxID used:135614 OG01927|WP\_012917346.1 | uracil-DNA glycosylase [Xanthomonas | taxID used:135614 OG01928|WP\_012916675.1 | monofunctional biosynthetic peptido | taxID used:135614 OG00192|WP\_012915868.1 | HAMP domain-containing protein, par | taxID used:135614 OG01930|WP\_012915890.1 | MULTISPECIES: RNA polymerase sigma | taxID used:135614 OG01931|WP\_012917071.1 | hypothetical protein [Xanthomonas a | taxID used:135614 OG01932|WP\_012915227.1 | trehalose-phosphatase [Xanthomonas | taxID used:135614 OG01933|WP\_012915916.1 | SDR family NAD(P)-dependent oxidore | taxID used:135614 OG01935|WP\_012915935.1 | flagellar basal body rod protein Fl | taxID used:135614 OG01936|WP\_012915617.1 | beta-ketoacyl-ACP reductase [Xantho | taxID used:135614 OG01937|WP\_012914997.1 | KR domain-containing protein [Xanth | taxID used:135614 OG01938|WP\_012916140.1 | 3-hydroxybutyrate dehydrogenase [Xa | taxID used:135614 OG01939|WP\_012914858.1 | hypothetical protein [Xanthomonas a | taxID used:135614 OG01940|WP\_045756702.1 | haloacid dehalogenase [Xanthomonas | taxID used:135614 OG01941|WP\_012916411.1 | RNA methyltransferase [Xanthomonas | taxID used:135614 OG01942|WP\_012915957.1 | leucyl/phenylalanyl-tRNA--protein t | taxID used:135614 OG01943|WP\_045756925.1 | di-trans,poly-cis-decaprenylcistran | taxID used:135614 OG01944|WP\_012916483.1 | DUF2063 domain-containing protein [ | taxID used:135614 OG01946|WP\_012915109.1 | UDP-2,3-diacylglucosamine diphospha | taxID used:135614 OG01947|WP\_012915523.1 | triose-phosphate isomerase [Xanthom | taxID used:135614 OG01948|WP\_045756687.1 | amidophosphoribosyltransferase [Xan | taxID used:135614 OG01949|WP\_012915423.1 | septum site-determining protein Min | taxID used:135614 OG00194|WP\_045757404.1 | DUF3772 domain-containing protein [ | taxID used:135614 OG01950|WP\_012916224.1 | DUF3011 domain-containing protein [ | taxID used:135614 OG01952|WP\_049887182.1 | molecular chaperone [Xanthomonas al | taxID used:135614 OG01953|WP\_012917500.1 | protocatechuate 3,4-dioxygenase sub | taxID used:135614 OG01954|WP\_012917505.1 | GntR family transcriptional regulat | taxID used:135614 OG01955|WP\_012917006.1 | S-methyl-5'-thioinosine phosphoryla | taxID used:135614 OG01957|WP\_080929804.1 | ferredoxin--NADP(+) reductase [Xant | taxID used:135614 OG01958|WP\_012916406.1 | bifunctional 2-polyprenyl-6-hydroxy | taxID used:135614 OG01959|WP\_012916710.1 | copper homeostasis protein CutC [Xa | taxID used:135614 OG00195|WP\_012915519.1 | GGDEF domain-containing protein [Xa | taxID used:135614 OG01960|WP\_012917240.1 | TerC family protein [Xanthomonas al | taxID used:135614 OG01961|WP\_045757244.1 | CDP-diacylglycerol diphosphatase [X | taxID used:135614 OG01963|WP\_012917475.1 | ABC transporter ATP-binding protein | taxID used:135614 OG01964|WP\_080928154.1 | general secretion pathway protein G | taxID used:135614 OG01965|WP\_045756728.1 | 4-hydroxy-2-oxovalerate aldolase [X | taxID used:135614 OG01966|WP\_012914638.1 | twin-arginine translocase subunit T | taxID used:135614 OG01967|WP\_045756642.1 | glutamine amidotransferase [Xanthom | taxID used:135614 OG01968|WP\_012916485.1 | 23S rRNA (guanosine(2251)-2'-O)-met | taxID used:135614 OG01969|WP\_012915155.1 | 3-oxoacyl-ACP reductase FabG [Xanth | taxID used:135614 OG00196|WP\_012915598.1 | phenylalanine--tRNA ligase subunit | taxID used:135614 OG01970|WP\_012915886.1 | flagellar motor protein [Xanthomona | taxID used:135614 OG01971|WP\_045756646.1 | 1-acyl-sn-glycerol-3-phosphate acyl | taxID used:135614 OG01972|WP\_012917168.1 | electron transfer flavoprotein subu | taxID used:135614 OG01973|WP\_045757021.1 | CoA transferase subunit A [Xanthomo | taxID used:135614 OG01976|WP\_012915130.1 | DNA polymerase III subunit epsilon | taxID used:135614 OG01977|WP\_012916187.1 | sulfurtransferase [Xanthomonas albi | taxID used:135614 OG01978|WP\_012914612.1 | orotidine-5'-phosphate decarboxylas | taxID used:135614 OG01979|WP\_045757415.1 | hypothetical protein [Xanthomonas a | taxID used:135614 OG00197|WP\_012916506.1 | fimbrial biogenesis outer membrane | taxID used:135614 OG01980|WP\_012917332.1 | DUF4339 domain-containing protein [ | taxID used:135614 OG01981|WP\_012914593.1 | ABC transporter permease [Xanthomon | taxID used:135614 OG01982|WP\_012914814.1 | type III pantothenate kinase [Xanth | taxID used:135614 OG01984|WP\_012916936.1 | DUF541 domain-containing protein [X | taxID used:135614 OG01985|WP\_012916811.1 | DUF3348 domain-containing protein [ | taxID used:135614 OG01986|WP\_012915062.1 | 30S ribosomal protein S3 [Xanthomon | taxID used:135614 OG01987|WP\_012915789.1 | KR domain-containing protein [Xanth | taxID used:135614 OG01988|WP\_012914672.1 | glycosyltransferase family 2 protei | taxID used:135614 OG01989|WP\_012914700.1 | 3-oxoacyl-ACP reductase FabG [Xanth | taxID used:135614 OG00198|WP\_045756691.1 | phosphomannomutase/phosphoglucomuta | taxID used:135614 OG01990|WP\_012917043.1 | isopentenyl transferase [Xanthomona | taxID used:135614 OG01991|WP\_012916906.1 | YebC/PmpR family DNA-binding transc | taxID used:135614 OG01992|WP\_012916228.1 | cell division protein ZipA [Xanthom | taxID used:135614 OG01994|WP\_045756927.1 | 23S rRNA pseudouridine synthase F [ | taxID used:135614 OG01995|WP\_012915361.1 | KR domain-containing protein [Xanth | taxID used:135614 OG01996|WP\_012917494.1 | ABC transporter ATP-binding protein | taxID used:135614 OG01997|WP\_012917298.1 | DNA-binding response regulator [Xan | taxID used:135614 OG01998|WP\_012915221.1 | ABC transporter ATP-binding protein | taxID used:135614 OG01999|WP\_012915771.1 | 1-(5-phosphoribosyl)-5-[(5-phosphor | taxID used:135614 OG02000|WP\_012917039.1 | hypothetical protein [Xanthomonas a | taxID used:135614 OG02001|WP\_012916605.1 | nucleotidyltransferase family prote | taxID used:135614 OG02002|WP\_012917061.1 | DNA-binding response regulator [Xan | taxID used:135614 OG02003|WP\_012914845.1 | haloacid dehalogenase-like hydrolas | taxID used:135614 OG02004|WP\_012916323.1 | 6-phosphogluconolactonase [Xanthomo | taxID used:135614 OG02005|WP\_045756881.1 | hypothetical protein [Xanthomonas a | taxID used:135614 OG02006|WP\_012916695.1 | 16S rRNA (uracil(1498)-N(3))-methyl | taxID used:135614 OG02007|WP\_012916606.1 | DnaA regulatory inactivator Hda [Xa | taxID used:135614 OG02009|WP\_012916150.1 | superoxide dismutase [Xanthomonas a | taxID used:135614 OG00200|WP\_012915960.1 | DNA translocase FtsK [Xanthomonas a | taxID used:135614 OG02010|WP\_012916453.1 | AraC family transcriptional regulat | taxID used:135614 OG02011|WP\_012914582.1 | hypothetical protein [Xanthomonas a | taxID used:135614 OG02012|WP\_012917626.1 | MgtC/SapB family protein [Xanthomon | taxID used:135614 OG02013|WP\_012916480.1 | phosphate transport system regulato | taxID used:135614 OG02015|WP\_012915323.1 | ribonuclease PH [Xanthomonas albili | taxID used:135614 OG02017|WP\_045757200.1 | DUF2242 domain-containing protein [ | taxID used:135614 OG02018|WP\_080929679.1 | DNA-binding response regulator [Xan | taxID used:135614 OG00201|WP\_012915187.1 | TonB-dependent receptor [Xanthomona | taxID used:135614 OG02020|WP\_012916512.1 | UMP kinase [Xanthomonas albilineans | taxID used:135614 OG02021|WP\_012916987.1 | RNA-binding protein S4 [Xanthomonas | taxID used:135614 OG02022|WP\_012916552.1 | deoxyribonuclease V [Xanthomonas al | taxID used:135614 OG02023|WP\_045757301.1 | lipoprotein-releasing ABC transport | taxID used:135614 OG02027|WP\_012915131.1 | serine/threonine-protein phosphatas | taxID used:135614 OG02028|WP\_012917221.1 | hypothetical protein [Xanthomonas a | taxID used:135614 OG02029|WP\_012917243.1 | MULTISPECIES: DNA-binding response | taxID used:135614 OG00202|WP\_012916135.1 | RNA-binding transcriptional accesso | taxID used:135614 OG02030|WP\_012915831.1 | acireductone synthase [Xanthomonas | taxID used:135614 OG02032|WP\_012916404.1 | phytoene synthase [Xanthomonas albi | taxID used:135614 OG02033|WP\_012915344.1 | lipoyl(octanoyl) transferase LipB [ | taxID used:135614 OG02034|WP\_012916748.1 | FAA hydrolase family protein [Xanth | taxID used:135614 OG02035|WP\_012916238.1 | DNA-binding response regulator [Xan | taxID used:135614 OG02036|WP\_012916622.1 | LPS export ABC transporter ATP-bind | taxID used:135614 OG02037|WP\_012917471.1 | glutathione S-transferase [Xanthomo | taxID used:135614 OG02039|WP\_012915624.1 | glutathione S-transferase [Xanthomo | taxID used:135614 OG00203|WP\_012914732.1 | 5-methyltetrahydropteroyltriglutama | taxID used:135614 OG02040|WP\_012916253.1 | DUF2461 domain-containing protein [ | taxID used:135614 OG02042|WP\_012916405.1 | phosphoglycolate phosphatase [Xanth | taxID used:135614 OG02045|WP\_012915848.1 | 4-hydroxy-tetrahydrodipicolinate re | taxID used:135614 OG02048|WP\_012914679.1 | hypothetical protein [Xanthomonas a | taxID used:135614 OG02049|WP\_012916470.1 | FKBP-type peptidyl-prolyl cis-trans | taxID used:135614 OG02050|WP\_012916279.1 | phosphatase [Xanthomonas albilinean | taxID used:135614 OG02051|WP\_080931558.1 | MotA/TolQ/ExbB proton channel famil | taxID used:135614 OG02052|WP\_012917263.1 | hypothetical protein [Xanthomonas a | taxID used:135614 OG02053|WP\_012916814.1 | DUF2894 domain-containing protein [ | taxID used:135614 OG02054|WP\_012916264.1 | DNA-binding response regulator [Xan | taxID used:135614 OG02056|WP\_012915418.1 | DNA-binding response regulator [Xan | taxID used:135614 OG02057|WP\_012917599.1 | DUF4194 domain-containing protein [ | taxID used:135614 OG02058|WP\_012917193.1 | dethiobiotin synthase [Xanthomonas | taxID used:135614 OG02059|WP\_012915933.1 | flagellar basal body L-ring protein | taxID used:135614 OG02060|WP\_012916728.1 | tRNA (adenosine(37)-N6)-threonylcar | taxID used:135614 OG02061|WP\_012915115.1 | phosphate regulon transcriptional r | taxID used:135614 OG02062|WP\_012916894.1 | 7-carboxy-7-deazaguanine synthase [ | taxID used:135614 OG02063|WP\_012914934.1 | hypothetical protein [Xanthomonas a | taxID used:135614 OG02064|WP\_012917395.1 | rRNA pseudouridine synthase [Xantho | taxID used:135614 OG02068|WP\_012914645.1 | MULTISPECIES: hypothetical protein | taxID used:135614 OG02069|WP\_012917012.1 | response regulator [Xanthomonas alb | taxID used:135614 OG00206|WP\_045756647.1 | membrane protein [Xanthomonas albil | taxID used:135614 OG02070|WP\_049887210.1 | hypothetical protein [Xanthomonas a | taxID used:135614 OG02071|WP\_012915672.1 | 2-C-methyl-D-erythritol 4-phosphate | taxID used:135614 OG02072|WP\_012916473.1 | endonuclease III [Xanthomonas albil | taxID used:135614 OG02073|WP\_012917503.1 | 3-oxoadipate CoA-transferase subuni | taxID used:135614 OG02074|WP\_045757439.1 | hypothetical protein [Xanthomonas a | taxID used:135614 OG02075|WP\_012915234.1 | ABC transporter ATP-binding protein | taxID used:135614 OG02076|WP\_012916791.1 | molybdate ABC transporter permease | taxID used:135614 OG02077|WP\_012915660.1 | cell envelope biogenesis protein Om | taxID used:135614 OG02078|WP\_012916613.1 | DUF3108 domain-containing protein [ | taxID used:135614 OG00207|WP\_012917124.1 | type II secretion system protein Gs | taxID used:135614 OG02081|WP\_012915050.1 | 50S ribosomal protein L1 [Xanthomon | taxID used:135614 OG02082|WP\_012916060.1 | DNA-binding response regulator [Xan | taxID used:135614 OG02083|WP\_012916614.1 | DUF3108 domain-containing protein [ | taxID used:135614 OG02085|WP\_012917390.1 | ABC transporter permease [Xanthomon | taxID used:135614 OG02087|WP\_012915383.1 | VIT family protein [Xanthomonas alb | taxID used:135614 OG02088|WP\_012914573.1 | energy transducer TonB [Xanthomonas | taxID used:135614 OG02089|WP\_012916454.1 | haloacid dehalogenase [Xanthomonas | taxID used:135614 OG00208|WP\_080931582.1 | glycosyl hydrolase [Xanthomonas alb | taxID used:135614 OG02090|WP\_012917009.1 | DsbA family oxidoreductase [Xanthom | taxID used:135614 OG02091|WP\_012917423.1 | class I SAM-dependent methyltransfe | taxID used:135614 OG02092|WP\_012916247.1 | carbonate dehydratase [Xanthomonas | taxID used:135614 OG02093|WP\_012917349.1 | cell division ATP-binding protein F | taxID used:135614 OG02095|WP\_003469280.1 | MULTISPECIES: DNA-binding response | taxID used:135614 OG02096|WP\_012916957.1 | DUF502 domain-containing protein [X | taxID used:135614 OG02097|WP\_012917188.1 | maleylacetoacetate isomerase [Xanth | taxID used:135614 OG02098|WP\_012915160.1 | dTMP kinase [Xanthomonas albilinean | taxID used:135614 OG02099|WP\_012917062.1 | carboxylesterase [Xanthomonas albil | taxID used:135614 OG00209|WP\_012915267.1 | NADP-dependent malic enzyme [Xantho | taxID used:135614 OG02102|WP\_012916672.1 | hemolysin III [Xanthomonas albiline | taxID used:135614 OG02103|WP\_012914930.1 | JAB domain-containing protein [Xant | taxID used:135614 OG02104|WP\_012915604.1 | HNH endonuclease [Xanthomonas albil | taxID used:135614 OG02105|WP\_012916829.1 | fimbrial protein [Xanthomonas albil | taxID used:135614 OG02106|WP\_012915373.1 | hypothetical protein [Xanthomonas a | taxID used:135614 OG02107|WP\_012916917.1 | DUF1751 domain-containing protein [ | taxID used:135614 OG02109|WP\_012915937.1 | flagellar basal body rod modificati | taxID used:135614 OG02111|WP\_045728928.1 | MULTISPECIES: DNA-binding response | taxID used:135614 OG02113|WP\_012914720.1 | alpha/beta hydrolase [Xanthomonas a | taxID used:135614 OG02114|WP\_012914949.1 | rhomboid family intramembrane serin | taxID used:135614 OG02115|WP\_045757388.1 | ribonuclease III [Xanthomonas albil | taxID used:135614 OG02116|WP\_080928215.1 | YggS family pyridoxal phosphate-dep | taxID used:135614 OG02117|WP\_012915677.1 | protein-L-isoaspartate(D-aspartate) | taxID used:135614 OG02118|WP\_012914837.1 | ribulose-phosphate 3-epimerase [Xan | taxID used:135614 OG02119|WP\_012915499.1 | dienelactone hydrolase family prote | taxID used:135614 OG00211|WP\_012914875.1 | cytochrome c biogenesis protein [Xa | taxID used:135614 OG02121|WP\_012915302.1 | serine/threonine protein kinase [Xa | taxID used:135614 OG02123|WP\_012917583.1 | hemolysin III [Xanthomonas albiline | taxID used:135614 OG02124|WP\_080931593.1 | molecular chaperone [Xanthomonas al | taxID used:135614 OG02126|WP\_012915007.1 | peptide-methionine (S)-S-oxide redu | taxID used:135614 OG02127|WP\_012916151.1 | DUF2058 domain-containing protein [ | taxID used:135614 OG02128|WP\_012916105.1 | (d)CMP kinase [Xanthomonas albiline | taxID used:135614 OG02129|WP\_012915511.1 | phosphoribosylanthranilate isomeras | taxID used:135614 OG00212|WP\_012915018.1 | DUF1631 domain-containing protein [ | taxID used:135614 OG02132|WP\_012916198.1 | energy transducer TonB [Xanthomonas | taxID used:135614 OG02134|WP\_012915276.1 | protein-L-isoaspartate O-methyltran | taxID used:135614 OG02135|WP\_012915808.1 | heme exporter protein CcmB [Xanthom | taxID used:135614 OG02136|WP\_012916893.1 | 7-cyano-7-deazaguanine synthase Que | taxID used:135614 OG02137|WP\_012916484.1 | ribonuclease T [Xanthomonas albilin | taxID used:135614 OG02139|WP\_012917050.1 | DUF484 domain-containing protein [X | taxID used:135614 OG00213|WP\_012915956.1 | ATP-dependent Clp protease ATP-bind | taxID used:135614 OG02141|WP\_012914944.1 | hypothetical protein [Xanthomonas a | taxID used:135614 OG02143|WP\_012915040.1 | lipoprotein localization protein Lo | taxID used:135614 OG02145|WP\_045757000.1 | HAD family hydrolase [Xanthomonas a | taxID used:135614 OG02146|WP\_012916658.1 | polyisoprenoid-binding protein [Xan | taxID used:135614 OG02149|WP\_012914656.1 | DNA-binding response regulator [Xan | taxID used:135614 OG02150|WP\_012915696.1 | DUF1275 domain-containing protein [ | taxID used:135614 OG02151|WP\_012915914.1 | acetyltransferase [Xanthomonas albi | taxID used:135614 OG02152|WP\_045757406.1 | arylesterase [Xanthomonas albilinea | taxID used:135614 OG02153|WP\_012917502.1 | 3-oxoadipate CoA-transferase subuni | taxID used:135614 OG02154|WP\_080929709.1 | protein-methionine-sulfoxide reduct | taxID used:135614 OG02155|WP\_080928209.1 | ribonuclease HII [Xanthomonas albil | taxID used:135614 OG02157|WP\_012914595.1 | organic solvent ABC transporter [Xa | taxID used:135614 OG02158|WP\_045756958.1 | hypothetical protein [Xanthomonas a | taxID used:135614 OG02159|WP\_012915504.1 | SCO family protein [Xanthomonas alb | taxID used:135614 OG02160|WP\_012916833.1 | hypothetical protein [Xanthomonas a | taxID used:135614 OG02162|WP\_012916783.1 | haloacid dehalogenase [Xanthomonas | taxID used:135614 OG02163|WP\_012917129.1 | general secretion pathway protein G | taxID used:135614 OG02164|WP\_012915974.1 | hypothetical protein [Xanthomonas a | taxID used:135614 OG02165|WP\_012917197.1 | phosphoglycolate phosphatase [Xanth | taxID used:135614 OG02166|WP\_012916611.1 | phosphoribosylglycinamide formyltra | taxID used:135614 OG02167|WP\_012915533.1 | NADH-quinone oxidoreductase subunit | taxID used:135614 OG02169|WP\_012915058.1 | 50S ribosomal protein L3 [Xanthomon | taxID used:135614 OG02170|WP\_012915833.1 | methylthioribulose 1-phosphate dehy | taxID used:135614 OG02171|WP\_012914850.1 | 2-nonaprenyl-3-methyl-6-methoxy-1,4 | taxID used:135614 OG02173|WP\_012916300.1 | CDP-diacylglycerol--glycerol-3-phos | taxID used:135614 OG02174|WP\_012915043.1 | 50S ribosomal protein L25 [Xanthomo | taxID used:135614 OG02176|WP\_012915637.1 | rhomboid family intramembrane serin | taxID used:135614 OG02177|WP\_012917156.1 | ABC transporter [Xanthomonas albili | taxID used:135614 OG02178|WP\_012915310.1 | ribose-5-phosphate isomerase RpiA [ | taxID used:135614 OG02179|WP\_012915103.1 | histidine phosphatase family protei | taxID used:135614 OG00217|WP\_012917119.1 | 1,4-alpha-glucan branching protein | taxID used:135614 OG02181|WP\_045757413.1 | DUF4124 domain-containing protein [ | taxID used:135614 OG02183|WP\_012916813.1 | hypothetical protein [Xanthomonas a | taxID used:135614 OG02184|WP\_012916325.1 | 2-dehydro-3-deoxy-phosphogluconate | taxID used:135614 OG02185|WP\_012917451.1 | DUF2076 domain-containing protein [ | taxID used:135614 OG02187|WP\_045757216.1 | methylamine utilization protein [Xa | taxID used:135614 OG02188|WP\_012916219.1 | TetR family transcriptional regulat | taxID used:135614 OG00218|WP\_012917470.1 | S9 family peptidase [Xanthomonas al | taxID used:135614 OG02190|WP\_012915777.1 | ubiquinol-cytochrome c reductase ir | taxID used:135614 OG02192|WP\_012915780.1 | stringent starvation protein A [Xan | taxID used:135614 OG02193|WP\_012915815.1 | DNA-binding response regulator [Xan | taxID used:135614 OG02194|WP\_012914842.1 | flavin reductase family protein [Xa | taxID used:135614 OG02195|WP\_012914935.1 | orotate phosphoribosyltransferase [ | taxID used:135614 OG02196|WP\_012916119.1 | DNA-binding response regulator [Xan | taxID used:135614 OG02199|WP\_045756790.1 | 23S rRNA (uridine(2552)-2'-O)-methy | taxID used:135614 OG00219|WP\_012915530.1 | NADH dehydrogenase (quinone) subuni | taxID used:135614 OG00021|WP\_012915053.1 | DNA-directed RNA polymerase subunit | taxID used:135614 OG02200|WP\_012916095.1 | TetR/AcrR family transcriptional re | taxID used:135614 OG02201|WP\_012916244.1 | DUF2589 domain-containing protein [ | taxID used:135614 OG02202|WP\_012917388.1 | membrane protein [Xanthomonas albil | taxID used:135614 OG02204|WP\_012915809.1 | heme ABC exporter ATP-binding prote | taxID used:135614 OG02205|WP\_080931580.1 | HAD family phosphatase [Xanthomonas | taxID used:135614 OG02206|WP\_012917076.1 | HTH-type transcriptional repressor | taxID used:135614 OG02207|WP\_012915325.1 | guanylate kinase [Xanthomonas albil | taxID used:135614 OG02208|WP\_012914853.1 | RNA pyrophosphohydrolase [Xanthomon | taxID used:135614 OG02209|WP\_012914666.1 | 4'-phosphopantetheinyl transferase | taxID used:135614 OG00220|WP\_012915873.1 | methyl-accepting chemotaxis protein | taxID used:135614 OG02210|WP\_080928228.1 | muramidase [Xanthomonas albilineans | taxID used:135614 OG02212|WP\_012917160.1 | CoA transferase subunit B [Xanthomo | taxID used:135614 OG02214|WP\_080929805.1 | tetratricopeptide repeat-containing | taxID used:135614 OG02216|WP\_012917537.1 | sterol-binding protein [Xanthomonas | taxID used:135614 OG02217|WP\_012917300.1 | potassium-transporting ATPase subun | taxID used:135614 OG02218|WP\_045756820.1 | flagella basal body P-ring formatio | taxID used:135614 OG02219|WP\_012914987.1 | hypothetical protein [Xanthomonas a | taxID used:135614 OG02220|WP\_012915432.1 | hypothetical protein [Xanthomonas a | taxID used:135614 OG02221|WP\_012915398.1 | SGNH/GDSL hydrolase family protein | taxID used:135614 OG02222|WP\_012917573.1 | ParA family protein [Xanthomonas al | taxID used:135614 OG02224|WP\_012917635.1 | thymidine kinase [Xanthomonas albil | taxID used:135614 OG02225|WP\_012915923.1 | DNA-binding response regulator [Xan | taxID used:135614 OG02226|WP\_012915426.1 | DNA-binding response regulator [Xan | taxID used:135614 OG02227|WP\_012915263.1 | DNA-binding response regulator [Xan | taxID used:135614 OG02228|WP\_012917431.1 | hypothetical protein [Xanthomonas a | taxID used:135614 OG02229|WP\_012915689.1 | transcriptional repressor LexA [Xan | taxID used:135614 OG00222|WP\_012917353.1 | NADP-dependent isocitrate dehydroge | taxID used:135614 OG02230|WP\_012917021.1 | RNA polymerase sigma factor RpoE [X | taxID used:135614 OG02231|WP\_045757072.1 | hypothetical protein [Xanthomonas a | taxID used:135614 OG02232|WP\_012914668.1 | 16S rRNA (guanine(527)-N(7))-methyl | taxID used:135614 OG02233|WP\_012915077.1 | 30S ribosomal protein S4 [Xanthomon | taxID used:135614 OG02234|WP\_012915122.1 | ATP-dependent Clp endopeptidase, pr | taxID used:135614 OG02235|WP\_012915962.1 | outer membrane lipoprotein carrier | taxID used:135614 OG02236|WP\_012916090.1 | membrane protein [Xanthomonas albil | taxID used:135614 OG02238|WP\_012915382.1 | YihA family ribosome biogenesis GTP | taxID used:135614 OG02239|WP\_012915458.1 | cytochrome o ubiquinol oxidase subu | taxID used:135614 OG00223|WP\_012916496.1 | DNA topoisomerase IV subunit A [Xan | taxID used:135614 OG02240|WP\_045756876.1 | CDP-diacylglycerol--serine O-phosph | taxID used:135614 OG02243|WP\_012915634.1 | superoxide dismutase [Xanthomonas a | taxID used:135614 OG02244|WP\_012916379.1 | N-acetyltransferase [Xanthomonas al | taxID used:135614 OG02245|WP\_012914908.1 | YdcF family protein [Xanthomonas al | taxID used:135614 OG02247|WP\_012915912.1 | acetyltransferase [Xanthomonas albi | taxID used:135614 OG02248|WP\_012915773.1 | bifunctional phosphoribosyl-AMP cyc | taxID used:135614 OG02249|WP\_049887209.1 | cytochrome c biogenesis protein [Xa | taxID used:135614 OG02250|WP\_012916028.1 | pentapeptide repeat-containing prot | taxID used:135614 OG02252|WP\_012914890.1 | polyisoprenoid-binding protein [Xan | taxID used:135614 OG02254|WP\_012917624.1 | DNA-binding response regulator [Xan | taxID used:135614 OG02257|WP\_012915678.1 | DedA family protein [Xanthomonas al | taxID used:135614 OG02258|WP\_012917565.1 | YbhB/YbcL family Raf kinase inhibit | taxID used:135614 OG02259|WP\_012916679.1 | TetR/AcrR family transcriptional re | taxID used:135614 OG02262|WP\_045756926.1 | class I SAM-dependent methyltransfe | taxID used:135614 OG02264|WP\_012916792.1 | molybdenum ABC transporter ATP-bind | taxID used:135614 OG02265|WP\_012917601.1 | GTP cyclohydrolase I FolE [Xanthomo | taxID used:135614 OG02266|WP\_012916955.1 | DUF998 domain-containing protein [X | taxID used:135614 OG02267|WP\_012917577.1 | DUF938 domain-containing protein [X | taxID used:135614 OG02268|WP\_012916208.1 | poly(hydroxyalkanoate) granule-asso | taxID used:135614 OG02269|WP\_045757135.1 | hypothetical protein [Xanthomonas a | taxID used:135614 OG00226|WP\_045756737.1 | GGDEF domain-containing protein [Xa | taxID used:135614 OG02270|WP\_012915191.1 | dephospho-CoA kinase [Xanthomonas a | taxID used:135614 OG02271|WP\_012915088.1 | glutathione S-transferase [Xanthomo | taxID used:135614 OG02272|WP\_012915707.1 | 2-dehydro-3-deoxy-6-phosphogalacton | taxID used:135614 OG02274|WP\_012916963.1 | HAD-IB family hydrolase [Xanthomona | taxID used:135614 OG02275|WP\_012917210.1 | thioredoxin family protein [Xanthom | taxID used:135614 OG02277|WP\_012917472.1 | TIGR00645 family protein [Xanthomon | taxID used:135614 OG02278|WP\_012915306.1 | thiamine phosphate synthase [Xantho | taxID used:135614 OG02279|WP\_012916907.1 | alpha/beta hydrolase [Xanthomonas a | taxID used:135614 OG00227|WP\_012915486.1 | catalase/peroxidase HPI [Xanthomona | taxID used:135614 OG02280|WP\_012915032.1 | TlpA family protein disulfide reduc | taxID used:135614 OG02281|WP\_045757343.1 | hypothetical protein [Xanthomonas a | taxID used:135614 OG02282|WP\_012915952.1 | lysogenization regulator HflD [Xant | taxID used:135614 OG02283|WP\_012916647.1 | pyridoxamine 5'-phosphate oxidase [ | taxID used:135614 OG02284|WP\_012915143.1 | recombination protein RecR [Xanthom | taxID used:135614 OG02285|WP\_012915167.1 | DUF1003 domain-containing protein [ | taxID used:135614 OG00228|WP\_045757130.1 | alpha-glucuronidase [Xanthomonas al | taxID used:135614 OG02290|WP\_045756809.1 | DsbE family thiol:disulfide interch | taxID used:135614 OG02293|WP\_045757115.1 | heme oxygenase [Xanthomonas albilin | taxID used:135614 OG02295|WP\_012915905.1 | flagellar assembly protein FliH [Xa | taxID used:135614 OG02296|WP\_045756903.1 | hypothetical protein [Xanthomonas a | taxID used:135614 OG02297|WP\_012915866.1 | chemoreceptor glutamine deamidase C | taxID used:135614 OG02299|WP\_012917044.1 | hypothetical protein [Xanthomonas a | taxID used:135614 OG00022|WP\_012916460.1 | PAS domain S-box protein [Xanthomon | taxID used:135614 OG02301|WP\_012915424.1 | GNAT family N-acetyltransferase [Xa | taxID used:135614 OG02304|WP\_045757240.1 | imidazole glycerol phosphate syntha | taxID used:135614 OG02305|WP\_045756732.1 | TetR/AcrR family transcriptional re | taxID used:135614 OG02306|WP\_012914653.1 | hypothetical protein [Xanthomonas a | taxID used:135614 OG02308|WP\_012916430.1 | 16S rRNA (guanine(966)-N(2))-methyl | taxID used:135614 OG02309|WP\_012916621.1 | lipopolysaccharide transport peripl | taxID used:135614 OG00230|WP\_012917070.1 | glycogen debranching enzyme GlgX [X | taxID used:135614 OG02310|WP\_012915312.1 | 5-formyltetrahydrofolate cyclo-liga | taxID used:135614 OG02312|WP\_012915888.1 | chemotaxis protein [Xanthomonas alb | taxID used:135614 OG02314|WP\_012917547.1 | Ax21 family protein [Xanthomonas al | taxID used:135614 OG02315|WP\_012917056.1 | membrane protein [Xanthomonas albil | taxID used:135614 OG02316|WP\_012915059.1 | 50S ribosomal protein L4 [Xanthomon | taxID used:135614 OG02318|WP\_012915538.1 | ribosome maturation factor RimP [Xa | taxID used:135614 OG02319|WP\_012915322.1 | non-canonical purine NTP pyrophosph | taxID used:135614 OG00231|WP\_012915171.1 | TonB-dependent receptor [Xanthomona | taxID used:135614 OG02320|WP\_012917228.1 | hypothetical protein [Xanthomonas a | taxID used:135614 OG02321|WP\_012915557.1 | DUF1439 domain-containing protein [ | taxID used:135614 OG02323|WP\_012916113.1 | Fe-S biogenesis protein NfuA [Xanth | taxID used:135614 OG02326|WP\_012914956.1 | cytochrome c oxidase assembly prote | taxID used:135614 OG02327|WP\_012915030.1 | NAD(P)H:quinone oxidoreductase [Xan | taxID used:135614 OG02328|WP\_012916087.1 | molybdopterin-guanine dinucleotide | taxID used:135614 OG02329|WP\_012917309.1 | riboflavin synthase [Xanthomonas al | taxID used:135614 OG00232|WP\_012915876.1 | HAMP domain-containing protein [Xan | taxID used:135614 OG02330|WP\_080928039.1 | hypothetical protein [Xanthomonas a | taxID used:135614 OG02331|WP\_012915975.1 | DNA-directed RNA polymerase sigma-7 | taxID used:135614 OG02334|WP\_012916466.1 | N-acetylmuramoyl-L-alanine amidase | taxID used:135614 OG02335|WP\_012914585.1 | protein-S-isoprenylcysteine methylt | taxID used:135614 OG02338|WP\_012917157.1 | alpha-ketoglutarate-dependent dioxy | taxID used:135614 OG02339|WP\_012916146.1 | oligoribonuclease [Xanthomonas albi | taxID used:135614 OG02340|WP\_012915787.1 | superoxide dismutase [Xanthomonas a | taxID used:135614 OG02341|WP\_012914841.1 | type 1 glutamine amidotransferase [ | taxID used:135614 OG02342|WP\_012914889.1 | malonic semialdehyde reductase [Xan | taxID used:135614 OG02343|WP\_012915559.1 | MULTISPECIES: glycine zipper 2TM do | taxID used:135614 OG02344|WP\_045757407.1 | ABC transporter ATP-binding protein | taxID used:135614 OG02345|WP\_012915044.1 | aminoacyl-tRNA hydrolase [Xanthomon | taxID used:135614 OG02346|WP\_012914825.1 | cytochrome b [Xanthomonas albilinea | taxID used:135614 OG02347|WP\_012916568.1 | hypothetical protein [Xanthomonas a | taxID used:135614 OG02348|WP\_045756735.1 | ATP-dependent protease [Xanthomonas | taxID used:135614 OG02349|WP\_045757434.1 | superoxide dismutase family protein | taxID used:135614 OG00234|WP\_012914864.1 | TIGR01666 family membrane protein [ | taxID used:135614 OG02350|WP\_012915496.1 | dCTP deaminase [Xanthomonas albilin | taxID used:135614 OG02351|WP\_012915280.1 | 3-isopropylmalate dehydratase small | taxID used:135614 OG02352|WP\_012916169.1 | TIGR00730 family Rossman fold prote | taxID used:135614 OG02353|WP\_012916931.1 | DUF615 domain-containing protein [X | taxID used:135614 OG02354|WP\_012915022.1 | RNA polymerase sigma factor [Xantho | taxID used:135614 OG02355|WP\_012915832.1 | acireductone dioxygenase [Xanthomon | taxID used:135614 OG02358|WP\_012914874.1 | TlpA family protein disulfide reduc | taxID used:135614 OG02359|WP\_012917270.1 | DUF3228 domain-containing protein [ | taxID used:135614 OG00235|WP\_012914676.1 | DNA helicase II [Xanthomonas albili | taxID used:135614 OG02360|WP\_012915017.1 | nitroreductase [Xanthomonas albilin | taxID used:135614 OG02361|WP\_012915797.1 | type IV pilus protein signal pilx [ | taxID used:135614 OG02362|WP\_012915924.1 | PilZ domain-containing protein [Xan | taxID used:135614 OG02363|WP\_012916659.1 | cytochrome b [Xanthomonas albilinea | taxID used:135614 OG02364|WP\_012917426.1 | flavodoxin family protein [Xanthomo | taxID used:135614 OG02365|WP\_012914960.1 | hypothetical protein [Xanthomonas a | taxID used:135614 OG02368|WP\_012916738.1 | DNA starvation/stationary phase pro | taxID used:135614 OG02369|WP\_012916904.1 | Holliday junction branch migration | taxID used:135614 OG00236|WP\_012917248.1 | TonB-dependent hemoglobin/transferr | taxID used:135614 OG02370|WP\_012916408.1 | elongation factor P [Xanthomonas al | taxID used:135614 OG02371|WP\_012915011.1 | MULTISPECIES: peroxiredoxin [Xantho | taxID used:135614 OG02372|WP\_045756992.1 | lipoprotein [Xanthomonas albilinean | taxID used:135614 OG02373|WP\_012917523.1 | type I-F CRISPR-associated endoribo | taxID used:135614 OG02374|WP\_045757043.1 | DNA-deoxyinosine glycosylase [Xanth | taxID used:135614 OG02376|WP\_012916620.1 | LPS export ABC transporter periplas | taxID used:135614 OG02377|WP\_012917374.1 | gamma subclass chorismate mutase Ar | taxID used:135614 OG02378|WP\_012916155.1 | glutathione peroxidase [Xanthomonas | taxID used:135614 OG02379|WP\_012914654.1 | manganese efflux pump MntP [Xanthom | taxID used:135614 OG00237|WP\_012916191.1 | sensor domain-containing diguanylat | taxID used:135614 OG02380|WP\_010343006.1 | MULTISPECIES: NADH-quinone oxidored | taxID used:135614 OG02382|WP\_012917165.1 | dTDP-4-dehydrorhamnose 3,5-epimeras | taxID used:135614 OG02383|WP\_045757430.1 | acyltransferase [Xanthomonas albili | taxID used:135614 OG02385|WP\_012916779.1 | O-acetyl-ADP-ribose deacetylase [Xa | taxID used:135614 OG02386|WP\_012915150.1 | septum formation inhibitor Maf [Xan | taxID used:135614 OG02387|WP\_012915841.1 | elongation factor P-like protein Ye | taxID used:135614 OG02388|WP\_012916160.1 | DUF2884 domain-containing protein [ | taxID used:135614 OG02389|WP\_045757202.1 | AAA family ATPase [Xanthomonas albi | taxID used:135614 OG02390|WP\_045756791.1 | glycine cleavage system regulatory | taxID used:135614 OG02393|WP\_012915048.1 | transcription termination/antitermi | taxID used:135614 OG02394|WP\_012915794.1 | prepilin-type N-terminal cleavage/m | taxID used:135614 OG02398|WP\_012916640.1 | YqgE/AlgH family protein [Xanthomon | taxID used:135614 OG02399|WP\_012917499.1 | protocatechuate 3,4-dioxygenase sub | taxID used:135614 OG00239|WP\_012917343.1 | primosomal protein N' [Xanthomonas | taxID used:135614 OG00023|WP\_012916737.1 | ATP-dependent RNA helicase HrpA [Xa | taxID used:135614 OG02400|WP\_012917217.1 | nucleoside deaminase [Xanthomonas a | taxID used:135614 OG02401|WP\_052689715.1 | DUF3426 domain-containing protein [ | taxID used:135614 OG02406|WP\_012914684.1 | hypothetical protein [Xanthomonas a | taxID used:135614 OG02407|WP\_045757292.1 | DUF2589 domain-containing protein [ | taxID used:135614 OG02408|WP\_012916660.1 | polyisoprenoid-binding protein [Xan | taxID used:135614 OG00240|WP\_012916026.1 | aminodeoxychorismate synthase, comp | taxID used:135614 OG02411|WP\_012915026.1 | putative Fe-S cluster assembly prot | taxID used:135614 OG02413|WP\_012916967.1 | RNA polymerase sigma factor [Xantho | taxID used:135614 OG02415|WP\_045757169.1 | NUDIX hydrolase [Xanthomonas albili | taxID used:135614 OG02416|WP\_012917417.1 | hypothetical protein [Xanthomonas a | taxID used:135614 OG02418|WP\_012917335.1 | tRNA threonylcarbamoyladenosine bio | taxID used:135614 OG02419|WP\_012917460.1 | ankyrin repeat domain-containing pr | taxID used:135614 OG02420|WP\_012917007.1 | hypoxanthine-guanine phosphoribosyl | taxID used:135614 OG02421|WP\_012915395.1 | DUF924 domain-containing protein [X | taxID used:135614 OG02422|WP\_080928065.1 | translation initiation factor IF-3 | taxID used:135614 OG02425|WP\_012915726.1 | DNA-binding response regulator [Xan | taxID used:135614 OG02427|WP\_012916693.1 | ADP compounds hydrolase NudE [Xanth | taxID used:135614 OG02428|WP\_012916513.1 | ribosome recycling factor [Xanthomo | taxID used:135614 OG02429|WP\_012917394.1 | DUF1415 domain-containing protein [ | taxID used:135614 OG00242|WP\_012915535.1 | NADH-quinone oxidoreductase subunit | taxID used:135614 OG02431|WP\_012916935.1 | septum formation inhibitor Maf [Xan | taxID used:135614 OG02437|WP\_012917229.1 | cob(I)yrinic acid a,c-diamide adeno | taxID used:135614 OG00243|WP\_012915327.1 | bifunctional (p)ppGpp synthetase/gu | taxID used:135614 OG02440|WP\_012915836.1 | NUDIX hydrolase [Xanthomonas albili | taxID used:135614 OG02441|WP\_012916641.1 | DNA-3-methyladenine glycosylase I [ | taxID used:135614 OG02442|WP\_012916973.1 | DUF2878 domain-containing protein [ | taxID used:135614 OG02443|WP\_012916619.1 | 3-deoxy-D-manno-octulosonate 8-phos | taxID used:135614 OG02444|WP\_012916248.1 | 3-hydroxyanthranilate 3,4-dioxygena | taxID used:135614 OG02445|WP\_012917534.1 | DUF2059 domain-containing protein [ | taxID used:135614 OG02446|WP\_012917003.1 | polymer-forming cytoskeletal protei | taxID used:135614 OG02448|WP\_012914899.1 | gamma carbonic anhydrase family pro | taxID used:135614 OG02449|WP\_012915068.1 | 50S ribosomal protein L5 [Xanthomon | taxID used:135614 OG02450|WP\_012915296.1 | adenylate kinase [Xanthomonas albil | taxID used:135614 OG02451|WP\_012916921.1 | peptidoglycan endopeptidase [Xantho | taxID used:135614 OG02452|WP\_012915622.1 | hypothetical protein [Xanthomonas a | taxID used:135614 OG02453|WP\_012914598.1 | glutathione peroxidase [Xanthomonas | taxID used:135614 OG02454|WP\_012916549.1 | N-acetyltransferase [Xanthomonas al | taxID used:135614 OG02455|WP\_012915437.1 | hypothetical protein [Xanthomonas a | taxID used:135614 OG02456|WP\_012915073.1 | 30S ribosomal protein S5 [Xanthomon | taxID used:135614 OG02457|WP\_012916541.1 | methylated-DNA--[protein]-cysteine | taxID used:135614 OG02458|WP\_012916361.1 | prepilin-type N-terminal cleavage/m | taxID used:135614 OG02459|WP\_012915403.1 | GNAT family N-acetyltransferase [Xa | taxID used:135614 OG00245|WP\_045757061.1 | ligand-gated channel [Xanthomonas a | taxID used:135614 OG02460|WP\_012916648.1 | shikimate kinase [Xanthomonas albil | taxID used:135614 OG02461|WP\_012916828.1 | fimbrial protein [Xanthomonas albil | taxID used:135614 OG02462|WP\_012915968.1 | DsbE family thiol:disulfide interch | taxID used:135614 OG02463|WP\_012915294.1 | hypothetical protein [Xanthomonas a | taxID used:135614 OG02464|WP\_012915291.1 | inorganic diphosphatase [Xanthomona | taxID used:135614 OG02465|WP\_012917048.1 | ATP-dependent protease subunit HslV | taxID used:135614 OG02466|WP\_012915528.1 | NADH-quinone oxidoreductase subunit | taxID used:135614 OG02467|WP\_012916246.1 | N-acetylmuramidase [Xanthomonas alb | taxID used:135614 OG02468|WP\_012917487.1 | ribosomal large subunit pseudouridi | taxID used:135614 OG02469|WP\_012915110.1 | phosphatase PAP2 family protein [Xa | taxID used:135614 OG00246|WP\_012916462.1 | bifunctional 23S rRNA (guanine(2069 | taxID used:135614 OG02470|WP\_080929772.1 | hypothetical protein [Xanthomonas a | taxID used:135614 OG02473|WP\_012915549.1 | N-acetyltransferase [Xanthomonas al | taxID used:135614 OG02475|WP\_012916385.1 | hypothetical protein [Xanthomonas a | taxID used:135614 OG02476|WP\_012915316.1 | hypothetical protein [Xanthomonas a | taxID used:135614 OG02477|WP\_012914637.1 | twin-arginine translocase subunit T | taxID used:135614 OG02478|WP\_012916986.1 | DUF2058 domain-containing protein [ | taxID used:135614 OG02479|WP\_012917312.1 | transcriptional repressor NrdR [Xan | taxID used:135614 OG00247|WP\_012915857.1 | hybrid sensor histidine kinase/resp | taxID used:135614 OG02481|WP\_012915145.1 | membrane protein [Xanthomonas albil | taxID used:135614 OG02483|WP\_045757232.1 | SMR domain protein [Xanthomonas alb | taxID used:135614 OG02484|WP\_012916722.1 | chemotaxis protein CheW [Xanthomona | taxID used:135614 OG02487|WP\_012914594.1 | outer membrane lipid asymmetry main | taxID used:135614 OG02488|WP\_012914726.1 | flavodoxin [Xanthomonas albilineans | taxID used:135614 OG02489|WP\_012916254.1 | DUF2939 domain-containing protein [ | taxID used:135614 OG00248|WP\_012914971.1 | beta-glucosidase [Xanthomonas albil | taxID used:135614 OG02490|WP\_012917319.1 | N-acetyltransferase [Xanthomonas al | taxID used:135614 OG02497|WP\_012915616.1 | polyhydroxyalkanoate synthesis repr | taxID used:135614 OG02498|WP\_012916464.1 | hypothetical protein [Xanthomonas a | taxID used:135614 OG02499|WP\_012915051.1 | 50S ribosomal protein L10 [Xanthomo | taxID used:135614 OG02501|WP\_012916896.1 | peptidoglycan-associated lipoprotei | taxID used:135614 OG02502|WP\_012915565.1 | single-stranded DNA-binding protein | taxID used:135614 OG02504|WP\_012916908.1 | N-acetyltransferase [Xanthomonas al | taxID used:135614 OG02505|WP\_012915901.1 | flagellar basal body protein FliL [ | taxID used:135614 OG02508|WP\_012915080.1 | disulfide bond formation protein B | taxID used:135614 OG02509|WP\_012915360.1 | 2-amino-4-hydroxy-6-hydroxymethyldi | taxID used:135614 OG02510|WP\_012915071.1 | 50S ribosomal protein L6 [Xanthomon | taxID used:135614 OG02511|WP\_012914715.1 | cytochrome c5 family protein [Xanth | taxID used:135614 OG02513|WP\_012916142.1 | DUF1249 domain-containing protein [ | taxID used:135614 OG02515|WP\_012917328.1 | peptide deformylase [Xanthomonas al | taxID used:135614 OG02518|WP\_045757419.1 | hypothetical protein [Xanthomonas a | taxID used:135614 OG02519|WP\_012916730.1 | hypothetical protein [Xanthomonas a | taxID used:135614 OG00251|WP\_012915102.1 | dipeptidyl-peptidase 7 [Xanthomonas | taxID used:135614 OG02520|WP\_012916463.1 | N-acetyltransferase [Xanthomonas al | taxID used:135614 OG02521|WP\_012915138.1 | cyclic pyranopterin monophosphate s | taxID used:135614 OG02522|WP\_012917549.1 | protein-export chaperone SecB [Xant | taxID used:135614 OG02523|WP\_012915034.1 | molybdenum cofactor biosynthesis pr | taxID used:135614 OG02524|WP\_012915641.1 | MarR family transcriptional regulat | taxID used:135614 OG02526|WP\_012916735.1 | hypothetical protein [Xanthomonas a | taxID used:135614 OG02527|WP\_012917379.1 | F0F1 ATP synthase subunit delta [Xa | taxID used:135614 OG02528|WP\_012916176.1 | MULTISPECIES: nucleotide exchange f | taxID used:135614 OG02529|WP\_012915451.1 | lipoprotein signal peptidase [Xanth | taxID used:135614 OG00252|WP\_012915346.1 | tail-specific protease [Xanthomonas | taxID used:135614 OG02530|WP\_012915702.1 | hypothetical protein [Xanthomonas a | taxID used:135614 OG02536|WP\_045756734.1 | hypothetical protein [Xanthomonas a | taxID used:135614 OG02537|WP\_012916183.1 | MULTISPECIES: SsrA-binding protein | taxID used:135614 OG02538|WP\_012915020.1 | DUF3106 domain-containing protein [ | taxID used:135614 OG02539|WP\_012914849.1 | hypothetical protein [Xanthomonas a | taxID used:135614 OG02540|WP\_045757374.1 | crossover junction endodeoxyribonuc | taxID used:135614 OG02541|WP\_012916505.1 | hypothetical protein [Xanthomonas a | taxID used:135614 OG02542|WP\_080929792.1 | DUF4019 domain-containing protein [ | taxID used:135614 OG02545|WP\_012914829.1 | Na+/H+ antiporter subunit E [Xantho | taxID used:135614 OG02547|WP\_012916740.1 | hypothetical protein [Xanthomonas a | taxID used:135614 OG02548|WP\_012917354.1 | hypothetical protein [Xanthomonas a | taxID used:135614 OG02549|WP\_045756717.1 | characterized ACR protein [Xanthomo | taxID used:135614 OG02550|WP\_012914834.1 | DUF962 domain-containing protein [X | taxID used:135614 OG02551|WP\_012915643.1 | glutathione peroxidase [Xanthomonas | taxID used:135614 OG02552|WP\_012915420.1 | hypothetical protein [Xanthomonas a | taxID used:135614 OG02554|WP\_012917010.1 | CYTH domain-containing protein [Xan | taxID used:135614 OG02555|WP\_045756793.1 | 2-amino-4-hydroxy-6-hydroxymethyldi | taxID used:135614 OG02556|WP\_012916545.1 | Fur family transcriptional regulato | taxID used:135614 OG02557|WP\_012915501.1 | transcription elongation factor Gre | taxID used:135614 OG02558|WP\_045757062.1 | hypothetical protein [Xanthomonas a | taxID used:135614 OG02559|WP\_012916429.1 | pantetheine-phosphate adenylyltrans | taxID used:135614 OG00255|WP\_012916715.1 | TonB-dependent siderophore receptor | taxID used:135614 OG02560|WP\_012915673.1 | 2-C-methyl-D-erythritol 2,4-cyclodi | taxID used:135614 OG02561|WP\_012917140.1 | RDD family protein [Xanthomonas alb | taxID used:135614 OG02563|WP\_012915785.1 | 5-(carboxyamino)imidazole ribonucle | taxID used:135614 OG02564|WP\_012915795.1 | type IV pilus modification protein | taxID used:135614 OG02565|WP\_012916141.1 | 8-oxo-dGTP diphosphatase [Xanthomon | taxID used:135614 OG02566|WP\_049887217.1 | dihydrofolate reductase [Xanthomona | taxID used:135614 OG02567|WP\_012915532.1 | MULTISPECIES: NADH-quinone oxidored | taxID used:135614 OG02568|WP\_080929663.1 | DUF4440 domain-containing protein [ | taxID used:135614 OG02569|WP\_012916303.1 | low molecular weight phosphotyrosin | taxID used:135614 OG00256|WP\_045757064.1 | peptidyl-dipeptidase Dcp [Xanthomon | taxID used:135614 OG02570|WP\_012916147.1 | tRNA adenosine(34) deaminase TadA [ | taxID used:135614 OG02571|WP\_012916497.1 | bacterioferritin [Xanthomonas albil | taxID used:135614 OG02573|WP\_012915438.1 | pathogenicity-like protein [Xanthom | taxID used:135614 OG02574|WP\_012915085.1 | peptidylprolyl isomerase [Xanthomon | taxID used:135614 OG02575|WP\_080931576.1 | gluconokinase [Xanthomonas albiline | taxID used:135614 OG02576|WP\_045757066.1 | hypothetical protein [Xanthomonas a | taxID used:135614 OG02577|WP\_012917146.1 | ribosomal-protein-alanine N-acetylt | taxID used:135614 OG00257|WP\_045757004.1 | oligopeptidase B [Xanthomonas albil | taxID used:135614 OG02580|WP\_012916923.1 | acyl-CoA thioesterase [Xanthomonas | taxID used:135614 OG02581|WP\_012916639.1 | Holliday junction resolvase RuvX [X | taxID used:135614 OG02582|WP\_012916428.1 | hypothetical protein [Xanthomonas a | taxID used:135614 OG02584|WP\_012915691.1 | recombination regulator RecX [Xanth | taxID used:135614 OG02585|WP\_012915098.1 | hypothetical protein [Xanthomonas a | taxID used:135614 OG02586|WP\_012915688.1 | CinA family protein [Xanthomonas al | taxID used:135614 OG02587|WP\_012915178.1 | peroxiredoxin [Xanthomonas albiline | taxID used:135614 OG02588|WP\_012916920.1 | peptidylprolyl isomerase [Xanthomon | taxID used:135614 OG00258|WP\_012917054.1 | S9 family peptidase [Xanthomonas al | taxID used:135614 OG02590|WP\_012915870.1 | chemotaxis protein CheW [Xanthomona | taxID used:135614 OG02591|WP\_012917209.1 | MarR family transcriptional regulat | taxID used:135614 OG02592|WP\_012915028.1 | asparaginase [Xanthomonas albilinea | taxID used:135614 OG02594|WP\_049887152.1 | hypothetical protein [Xanthomonas a | taxID used:135614 OG02595|WP\_012917311.1 | N-acetyltransferase [Xanthomonas al | taxID used:135614 OG02596|WP\_012916465.1 | MOSC domain-containing protein [Xan | taxID used:135614 OG02597|WP\_012915163.1 | hypothetical protein [Xanthomonas a | taxID used:135614 OG02599|WP\_012914838.1 | N-acetyltransferase [Xanthomonas al | taxID used:135614 OG00259|WP\_012915492.1 | peptidase [Xanthomonas albilineans] | taxID used:135614 OG02600|WP\_045756753.1 | copper chaperone PCu(A)C [Xanthomon | taxID used:135614 OG02602|WP\_012915237.1 | DUF3574 domain-containing protein [ | taxID used:135614 OG02603|WP\_012915385.1 | hypothetical protein [Xanthomonas a | taxID used:135614 OG02605|WP\_012914984.1 | hypothetical protein [Xanthomonas a | taxID used:135614 OG02607|WP\_012915649.1 | DUF456 domain-containing protein [X | taxID used:135614 OG02608|WP\_012915699.1 | peroxiredoxin [Xanthomonas albiline | taxID used:135614 OG02609|WP\_012915335.1 | rod shape-determining protein MreD | taxID used:135614 OG00260|WP\_012915251.1 | TonB-dependent siderophore receptor | taxID used:135614 OG02610|WP\_012916567.1 | rRNA maturation RNase YbeY [Xanthom | taxID used:135614 OG02611|WP\_012915611.1 | tRNA (adenosine(37)-N6)-threonylcar | taxID used:135614 OG02612|WP\_012914872.1 | acetyl-CoA carboxylase biotin carbo | taxID used:135614 OG02614|WP\_012917194.1 | GAF domain-containing protein [Xant | taxID used:135614 OG02615|WP\_012915311.1 | EVE domain-containing protein [Xant | taxID used:135614 OG02616|WP\_012917331.1 | DUF494 domain-containing protein [X | taxID used:135614 OG02617|WP\_012915967.1 | cytochrome c-type biogenesis protei | taxID used:135614 OG02618|WP\_012917582.1 | hypothetical protein [Xanthomonas a | taxID used:135614 OG02619|WP\_012914991.1 | DUF1456 domain-containing protein [ | taxID used:135614 OG00261|WP\_045756947.1 | bifunctional (p)ppGpp synthetase/gu | taxID used:135614 OG02620|WP\_012915633.1 | ribonuclease [Xanthomonas albilinea | taxID used:135614 OG02622|WP\_012914620.1 | Lrp/AsnC family transcriptional reg | taxID used:135614 OG02623|WP\_012915083.1 | DUF2127 domain-containing protein [ | taxID used:135614 OG02624|WP\_012917546.1 | tRNA (uridine(34)/cytosine(34)/5-ca | taxID used:135614 OG02625|WP\_045757435.1 | histidine phosphatase family protei | taxID used:135614 OG02626|WP\_012916718.1 | chemotaxis protein CheW [Xanthomona | taxID used:135614 OG02627|WP\_012917393.1 | YajQ family cyclic di-GMP-binding p | taxID used:135614 OG02628|WP\_012916599.1 | hypothetical protein [Xanthomonas a | taxID used:135614 OG02629|WP\_012915179.1 | MULTISPECIES: Hsp20/alpha crystalli | taxID used:135614 OG02635|WP\_012915651.1 | glycine zipper 2TM domain-containin | taxID used:135614 OG02636|WP\_012915556.1 | SET domain-containing protein-lysin | taxID used:135614 OG02637|WP\_012916553.1 | hypothetical protein [Xanthomonas a | taxID used:135614 OG02638|WP\_012917410.1 | peptide-methionine (R)-S-oxide redu | taxID used:135614 OG02639|WP\_012914873.1 | type II 3-dehydroquinate dehydratas | taxID used:135614 OG00263|WP\_012915081.1 | TonB-dependent receptor [Xanthomona | taxID used:135614 OG02641|WP\_012915602.1 | hypothetical protein [Xanthomonas a | taxID used:135614 OG02642|WP\_012916901.1 | tol-pal system-associated acyl-CoA | taxID used:135614 OG02644|WP\_012917277.1 | DUF721 domain-containing protein [X | taxID used:135614 OG02645|WP\_012915129.1 | ribonuclease HI [Xanthomonas albili | taxID used:135614 OG02646|WP\_012915606.1 | hypothetical protein [Xanthomonas a | taxID used:135614 OG02647|WP\_012915056.1 | 30S ribosomal protein S7 [Xanthomon | taxID used:135614 OG02648|WP\_045757398.1 | hypothetical protein [Xanthomonas a | taxID used:135614 OG00264|WP\_045757052.1 | beta-galactosidase [Xanthomonas alb | taxID used:135614 OG02650|WP\_012916948.1 | 23S rRNA (pseudouridine(1915)-N(3)) | taxID used:135614 OG02651|WP\_012917201.1 | hypothetical protein [Xanthomonas a | taxID used:135614 OG02652|WP\_012914855.1 | MULTISPECIES: bacterioferritin [Xan | taxID used:135614 OG02653|WP\_012915851.1 | transcription elongation factor Gre | taxID used:135614 OG02655|WP\_012915903.1 | flagellar export protein FliJ [Xant | taxID used:135614 OG02656|WP\_012917306.1 | transcription antitermination facto | taxID used:135614 OG02657|WP\_045757432.1 | thioesterase [Xanthomonas albilinea | taxID used:135614 OG02659|WP\_012915089.1 | cell wall hydrolase [Xanthomonas al | taxID used:135614 OG02660|WP\_012914932.1 | dUTP diphosphatase [Xanthomonas alb | taxID used:135614 OG02662|WP\_012915970.1 | cytochrome c biogenesis protein Ccm | taxID used:135614 OG02663|WP\_012915954.1 | NUDIX hydrolase [Xanthomonas albili | taxID used:135614 OG02664|WP\_012915802.1 | cytochrome c-type biogenesis protei | taxID used:135614 OG02666|WP\_045757291.1 | hypothetical protein [Xanthomonas a | taxID used:135614 OG02667|WP\_012916520.1 | 3-hydroxyacyl-[acyl-carrier-protein | taxID used:135614 OG02668|WP\_012917380.1 | F0F1 ATP synthase subunit B [Xantho | taxID used:135614 OG02669|WP\_012916111.1 | cytochrome c biogenesis protein Ccs | taxID used:135614 OG00266|WP\_012914644.1 | glycine--tRNA ligase subunit beta [ | taxID used:135614 OG02672|WP\_045757427.1 | MarR family transcriptional regulat | taxID used:135614 OG02674|WP\_012915309.1 | DUF192 domain-containing protein [X | taxID used:135614 OG02675|WP\_080931544.1 | glutaredoxin 3 [Xanthomonas albilin | taxID used:135614 OG02677|WP\_012917307.1 | 6,7-dimethyl-8-ribityllumazine synt | taxID used:135614 OG02678|WP\_012917314.1 | hypothetical protein [Xanthomonas a | taxID used:135614 OG00267|WP\_012915186.1 | phospholipase C, phosphocholine-spe | taxID used:135614 OG02680|WP\_012916148.1 | transcriptional regulator MntR [Xan | taxID used:135614 OG02684|WP\_045757233.1 | hypothetical protein [Xanthomonas a | taxID used:135614 OG02686|WP\_012916961.1 | DUF2147 domain-containing protein [ | taxID used:135614 OG02687|WP\_012915299.1 | hypothetical protein [Xanthomonas a | taxID used:135614 OG02689|WP\_012916824.1 | DUF4381 domain-containing protein [ | taxID used:135614 OG00268|WP\_012917462.1 | ATP-dependent DNA helicase DinG [Xa | taxID used:135614 OG02692|WP\_012916526.1 | membrane protein [Xanthomonas albil | taxID used:135614 OG02693|WP\_012915555.1 | SUF system Fe-S cluster assembly re | taxID used:135614 OG02696|WP\_012917151.1 | H-NS histone family protein [Xantho | taxID used:135614 OG02697|WP\_012914859.1 | cell shape determination protein Cc | taxID used:135614 OG02699|WP\_012916610.1 | hypothetical protein [Xanthomonas a | taxID used:135614 OG02700|WP\_012916312.1 | hypothetical protein [Xanthomonas a | taxID used:135614 OG02701|WP\_080928225.1 | type II secretion system protein Gs | taxID used:135614 OG02704|WP\_045756649.1 | MarR family transcriptional regulat | taxID used:135614 OG02706|WP\_012915521.1 | group 1 truncated hemoglobin [Xanth | taxID used:135614 OG02708|WP\_012916717.1 | DUF4440 domain-containing protein [ | taxID used:135614 OG02709|WP\_045757136.1 | phosphotransferase [Xanthomonas alb | taxID used:135614 OG00270|WP\_045757020.1 | hypothetical protein [Xanthomonas a | taxID used:135614 OG02711|WP\_012916161.1 | hypothetical protein [Xanthomonas a | taxID used:135614 OG02713|WP\_012917434.1 | type II 3-dehydroquinate dehydratas | taxID used:135614 OG02716|WP\_012917292.1 | transcriptional regulator MraZ [Xan | taxID used:135614 OG02718|WP\_012916494.1 | MarR family transcriptional regulat | taxID used:135614 OG02719|WP\_012915632.1 | hypothetical protein [Xanthomonas a | taxID used:135614 OG00271|WP\_012915329.1 | DNA helicase RecG [Xanthomonas albi | taxID used:135614 OG02720|WP\_012914678.1 | universal stress protein [Xanthomon | taxID used:135614 OG02721|WP\_045756907.1 | Fe-S cluster assembly protein SufE | taxID used:135614 OG02724|WP\_045756700.1 | GatB/YqeY domain-containing protein | taxID used:135614 OG02726|WP\_012916487.1 | hypothetical protein [Xanthomonas a | taxID used:135614 OG02728|WP\_045757119.1 | transcriptional regulator [Xanthomo | taxID used:135614 OG02729|WP\_045757285.1 | FKBP-type peptidyl-prolyl cis-trans | taxID used:135614 OG00272|WP\_012915592.1 | S9 family peptidase [Xanthomonas al | taxID used:135614 OG02730|WP\_045756682.1 | ATP-binding protein [Xanthomonas al | taxID used:135614 OG02738|WP\_012917469.1 | hypothetical protein [Xanthomonas a | taxID used:135614 OG02739|WP\_012916182.1 | ubiquinone-binding protein [Xanthom | taxID used:135614 OG00273|WP\_012917037.1 | peptidase domain-containing ABC tra | taxID used:135614 OG02740|WP\_012917644.1 | ribonuclease P protein component [X | taxID used:135614 OG02741|WP\_012916230.1 | 50S ribosomal protein L9 [Xanthomon | taxID used:135614 OG02742|WP\_012916231.1 | 30S ribosomal protein S6 [Xanthomon | taxID used:135614 OG02744|WP\_012915358.1 | MULTISPECIES: response regulator [X | taxID used:135614 OG02746|WP\_012915781.1 | ClpXP protease specificity-enhancin | taxID used:135614 OG02747|WP\_012916115.1 | energy transducer TonB [Xanthomonas | taxID used:135614 OG02749|WP\_012915255.1 | hypothetical protein [Xanthomonas a | taxID used:135614 OG00274|WP\_012915057.1 | elongation factor G [Xanthomonas al | taxID used:135614 OG02750|WP\_012916307.1 | biopolymer transporter ExbD [Xantho | taxID used:135614 OG02751|WP\_012916899.1 | protein TolR [Xanthomonas albilinea | taxID used:135614 OG02756|WP\_045756784.1 | hypothetical protein [Xanthomonas a | taxID used:135614 OG02757|WP\_012915075.1 | 50S ribosomal protein L15 [Xanthomo | taxID used:135614 OG02759|WP\_012914680.1 | hypothetical protein [Xanthomonas a | taxID used:135614 OG00275|WP\_045757047.1 | TonB-dependent siderophore receptor | taxID used:135614 OG02761|WP\_012914851.1 | MULTISPECIES: 50S ribosomal protein | taxID used:135614 OG02762|WP\_012915303.1 | lactoylglutathione lyase [Xanthomon | taxID used:135614 OG02763|WP\_012916689.1 | NfeD family protein [Xanthomonas al | taxID used:135614 OG02764|WP\_012917132.1 | type II secretion system protein Gs | taxID used:135614 OG02765|WP\_045757303.1 | hypothetical protein [Xanthomonas a | taxID used:135614 OG02766|WP\_012914575.1 | biopolymer transporter ExbD [Xantho | taxID used:135614 OG02767|WP\_012915140.1 | molybdopterin-converting factor sub | taxID used:135614 OG02768|WP\_012916673.1 | CBS domain-containing protein [Xant | taxID used:135614 OG00276|WP\_012917416.1 | tail fiber protein [Xanthomonas alb | taxID used:135614 OG02770|WP\_012915812.1 | hypothetical protein [Xanthomonas a | taxID used:135614 OG02771|WP\_045757397.1 | DNA polymerase III subunit chi [Xan | taxID used:135614 OG02772|WP\_012914577.1 | response regulator [Xanthomonas alb | taxID used:135614 OG02773|WP\_012917550.1 | rhodanese-like domain-containing pr | taxID used:135614 OG02774|WP\_012915799.1 | type IV pilin protein [Xanthomonas | taxID used:135614 OG02776|WP\_012914723.1 | organic hydroperoxide resistance pr | taxID used:135614 OG02777|WP\_012916179.1 | ferric iron uptake transcriptional | taxID used:135614 OG02778|WP\_045757065.1 | aminoacyl-tRNA hydrolase [Xanthomon | taxID used:135614 OG02779|WP\_012915926.1 | flagellar export chaperone FliS [Xa | taxID used:135614 OG02780|WP\_080928177.1 | DUF2244 domain-containing protein [ | taxID used:135614 OG02782|WP\_012916419.1 | Lrp/AsnC family transcriptional reg | taxID used:135614 OG02783|WP\_012916094.1 | nucleoside-diphosphate kinase [Xant | taxID used:135614 OG02785|WP\_012914576.1 | biopolymer transporter ExbD [Xantho | taxID used:135614 OG02787|WP\_012916964.1 | MULTISPECIES: RnfABCDGE type electr | taxID used:135614 OG02788|WP\_012915049.1 | MULTISPECIES: 50S ribosomal protein | taxID used:135614 OG02789|WP\_012915025.1 | hypothetical protein [Xanthomonas a | taxID used:135614 OG02790|WP\_012915021.1 | hypothetical protein [Xanthomonas a | taxID used:135614 OG02792|WP\_012915063.1 | MULTISPECIES: 50S ribosomal protein | taxID used:135614 OG02793|WP\_012917569.1 | thioesterase [Xanthomonas albilinea | taxID used:135614 OG02794|WP\_012917195.1 | transcriptional regulator [Xanthomo | taxID used:135614 OG02795|WP\_080931573.1 | hypothetical protein [Xanthomonas a | taxID used:135614 OG02799|WP\_012915404.1 | hypothetical protein [Xanthomonas a | taxID used:135614 OG02800|WP\_012916749.1 | large-conductance mechanosensitive | taxID used:135614 OG02804|WP\_012916566.1 | histone-like nucleoid-structuring p | taxID used:135614 OG02805|WP\_012915524.1 | preprotein translocase subunit SecG | taxID used:135614 OG02806|WP\_012917435.1 | hypothetical protein [Xanthomonas a | taxID used:135614 OG02807|WP\_012916950.1 | ribosome silencing factor [Xanthomo | taxID used:135614 OG02808|WP\_012917130.1 | prepilin-type N-terminal cleavage/m | taxID used:135614 OG02809|WP\_012917375.1 | F0F1 ATP synthase subunit epsilon [ | taxID used:135614 OG02811|WP\_012915541.1 | 30S ribosome-binding factor RbfA [X | taxID used:135614 OG02813|WP\_012915047.1 | preprotein translocase subunit SecE | taxID used:135614 OG02814|WP\_080931569.1 | hypothetical protein [Xanthomonas a | taxID used:135614 OG02815|WP\_012915482.1 | 50S ribosomal protein L19 [Xanthomo | taxID used:135614 OG02816|WP\_012915498.1 | HIT domain-containing protein [Xant | taxID used:135614 OG02817|WP\_012916544.1 | MerC domain-containing protein [Xan | taxID used:135614 OG02818|WP\_080931600.1 | hypothetical protein [Xanthomonas a | taxID used:135614 OG02820|WP\_045757057.1 | hypothetical protein [Xanthomonas a | taxID used:135614 OG02822|WP\_012915938.1 | MULTISPECIES: flagellar basal body | taxID used:135614 OG02824|WP\_080929670.1 | hypothetical protein [Xanthomonas a | taxID used:135614 OG02825|WP\_012915862.1 | PIN domain-containing protein [Xant | taxID used:135614 OG02826|WP\_012915485.1 | RNA-binding S4 domain-containing pr | taxID used:135614 OG02829|WP\_049887195.1 | hypothetical protein [Xanthomonas a | taxID used:135614 OG00282|WP\_012917603.1 | methyl-accepting chemotaxis protein | taxID used:135614 OG02830|WP\_045757386.1 | DUF2946 domain-containing protein [ | taxID used:135614 OG02832|WP\_012916180.1 | outer membrane protein assembly fac | taxID used:135614 OG02835|WP\_012915553.1 | DUF1311 domain-containing protein [ | taxID used:135614 OG02837|WP\_012916317.1 | succinate dehydrogenase, cytochrome | taxID used:135614 OG02838|WP\_012915132.1 | hypothetical protein [Xanthomonas a | taxID used:135614 OG02839|WP\_012917595.1 | hypothetical protein [Xanthomonas a | taxID used:135614 OG02841|WP\_012915898.1 | flagellar biosynthetic protein FliO | taxID used:135614 OG02842|WP\_012916272.1 | membrane protein [Xanthomonas albil | taxID used:135614 OG02844|WP\_012914989.1 | hypothetical protein [Xanthomonas a | taxID used:135614 OG02845|WP\_045728072.1 | MULTISPECIES: response regulator [X | taxID used:135614 OG02846|WP\_012915939.1 | flagellar basal body rod protein Fl | taxID used:135614 OG02847|WP\_049887213.1 | type VI secretion protein [Xanthomo | taxID used:135614 OG02848|WP\_012915118.1 | carboxymuconolactone decarboxylase | taxID used:135614 OG02849|WP\_012917637.1 | calcium-dependent protein kinase 21 | taxID used:135614 OG02850|WP\_045757025.1 | DUF4398 domain-containing protein [ | taxID used:135614 OG02852|WP\_080929788.1 | hypothetical protein [Xanthomonas a | taxID used:135614 OG02853|WP\_045757399.1 | DUF4124 domain-containing protein [ | taxID used:135614 OG00285|WP\_045757316.1 | hypothetical protein [Xanthomonas a | taxID used:135614 OG02862|WP\_012917586.1 | Elastase inhibitor AFLEI flags: Pre | taxID used:135614 OG02864|WP\_012917496.1 | 4-carboxymuconolactone decarboxylas | taxID used:135614 OG00286|WP\_012915113.1 | polyphosphate kinase 1 [Xanthomonas | taxID used:135614 OG02870|WP\_012915889.1 | chemotaxis protein CheY [Xanthomona | taxID used:135614 OG02871|WP\_012915070.1 | MULTISPECIES: 30S ribosomal protein | taxID used:135614 OG02874|WP\_003470632.1 | MULTISPECIES: 30S ribosomal protein | taxID used:135614 OG02875|WP\_012916687.1 | MULTISPECIES: glycine cleavage syst | taxID used:135614 OG02877|WP\_012917235.1 | Co2+/Mg2+ efflux protein ApaG [Xant | taxID used:135614 OG02878|WP\_012915608.1 | hypothetical protein [Xanthomonas a | taxID used:135614 OG00287|WP\_012917419.1 | tail fiber protein [Xanthomonas alb | taxID used:135614 OG02880|WP\_012915558.1 | DNA-binding protein [Xanthomonas al | taxID used:135614 OG02881|WP\_012915921.1 | DNA-binding response regulator [Xan | taxID used:135614 OG02882|WP\_045757332.1 | membrane protein [Xanthomonas albil | taxID used:135614 OG02883|WP\_012914852.1 | MULTISPECIES: 30S ribosomal protein | taxID used:135614 OG02886|WP\_012917540.1 | YkgJ family cysteine cluster protei | taxID used:135614 OG02887|WP\_012915181.1 | response regulator [Xanthomonas alb | taxID used:135614 OG00288|WP\_012916249.1 | FUSC family protein [Xanthomonas al | taxID used:135614 OG02890|WP\_045757041.1 | membrane protein [Xanthomonas albil | taxID used:135614 OG02895|WP\_012914860.1 | MULTISPECIES: iron-sulfur cluster i | taxID used:135614 OG02896|WP\_045756648.1 | membrane protein [Xanthomonas albil | taxID used:135614 OG00289|WP\_012915543.1 | polyribonucleotide nucleotidyltrans | taxID used:135614 OG00028|WP\_012917137.1 | phosphoribosylformylglycinamidine s | taxID used:135614 OG02900|WP\_012917241.1 | diacylglycerol kinase [Xanthomonas | taxID used:135614 OG02904|WP\_012915869.1 | hypothetical protein [Xanthomonas a | taxID used:135614 OG02906|WP\_012915079.1 | MULTISPECIES: 50S ribosomal protein | taxID used:135614 OG02910|WP\_012915328.1 | RidA family protein [Xanthomonas al | taxID used:135614 OG02914|WP\_012916627.1 | phosphotransferase system (pts), ma | taxID used:135614 OG02916|WP\_045757067.1 | hypothetical protein [Xanthomonas a | taxID used:135614 OG02917|WP\_012915000.1 | L-rhamnose mutarotase [Xanthomonas | taxID used:135614 OG02918|WP\_012914827.1 | Na+/H+ antiporter subunit G [Xantho | taxID used:135614 OG00291|WP\_012916992.1 | TonB-dependent receptor [Xanthomona | taxID used:135614 OG02921|WP\_012915721.1 | aspartate 1-decarboxylase [Xanthomo | taxID used:135614 OG02922|WP\_012914893.1 | anti-sigma factor antagonist [Xanth | taxID used:135614 OG02923|WP\_012916929.1 | DUF4870 domain-containing protein [ | taxID used:135614 OG02925|WP\_012917016.1 | DUF4845 domain-containing protein [ | taxID used:135614 OG02926|WP\_012916316.1 | succinate dehydrogenase, hydrophobi | taxID used:135614 OG00292|WP\_012916555.1 | carbon starvation protein A [Xantho | taxID used:135614 OG02930|WP\_012916472.1 | hypothetical protein [Xanthomonas a | taxID used:135614 OG02932|WP\_012915055.1 | 30S ribosomal protein S12 [Xanthomo | taxID used:135614 OG02934|WP\_012914977.1 | hypothetical protein [Xanthomonas a | taxID used:135614 OG02938|WP\_012915003.1 | hypothetical protein [Xanthomonas a | taxID used:135614 OG02939|WP\_045757006.1 | response regulator [Xanthomonas alb | taxID used:135614 OG00293|WP\_012915620.1 | PAS domain S-box protein [Xanthomon | taxID used:135614 OG02940|WP\_045756873.1 | cytochrome c [Xanthomonas albilinea | taxID used:135614 OG02941|WP\_045757401.1 | phasin family protein [Xanthomonas | taxID used:135614 OG02942|WP\_012915680.1 | hypothetical protein [Xanthomonas a | taxID used:135614 OG02943|WP\_012917191.1 | MULTISPECIES: 6-carboxytetrahydropt | taxID used:135614 OG02945|WP\_012916966.1 | hypothetical protein [Xanthomonas a | taxID used:135614 OG02946|WP\_012916787.1 | rhodanese-like domain-containing pr | taxID used:135614 OG02949|WP\_012915066.1 | 50S ribosomal protein L14 [Xanthomo | taxID used:135614 OG02952|WP\_012916796.1 | transcriptional regulator [Xanthomo | taxID used:135614 OG02954|WP\_012916133.1 | response regulator [Xanthomonas alb | taxID used:135614 OG02957|WP\_012917004.1 | hypothetical protein [Xanthomonas a | taxID used:135614 OG02958|WP\_012915351.1 | glycine zipper 2TM domain-containin | taxID used:135614 OG02959|WP\_012917296.1 | YraN family protein [Xanthomonas al | taxID used:135614 OG00295|WP\_012916962.1 | methionine--tRNA ligase [Xanthomona | taxID used:135614 OG02960|WP\_012915881.1 | MULTISPECIES: response regulator [X | taxID used:135614 OG02961|WP\_012916723.1 | response regulator [Xanthomonas alb | taxID used:135614 OG02962|WP\_012915428.1 | DUF423 domain-containing protein [X | taxID used:135614 OG02964|WP\_012916156.1 | GntR family transcriptional regulat | taxID used:135614 OG02965|WP\_012915029.1 | DUF2069 domain-containing protein [ | taxID used:135614 OG02967|WP\_045757005.1 | transcriptional regulator [Xanthomo | taxID used:135614 OG02969|WP\_012916501.1 | arsenate reductase (arsc) family pr | taxID used:135614 OG00296|WP\_012916812.1 | DUF802 domain-containing protein [X | taxID used:135614 OG02970|WP\_012916271.1 | membrane protein [Xanthomonas albil | taxID used:135614 OG02972|WP\_012915908.1 | flagellar hook-basal body complex p | taxID used:135614 OG02973|WP\_012914970.1 | dihydroneopterin aldolase [Xanthomo | taxID used:135614 OG02974|WP\_012915355.1 | hypothetical protein [Xanthomonas a | taxID used:135614 OG02976|WP\_012915525.1 | MULTISPECIES: NADH-quinone oxidored | taxID used:135614 OG02977|WP\_009597860.1 | MULTISPECIES: MerR family transcrip | taxID used:135614 OG02978|WP\_012914985.1 | hypothetical protein [Xanthomonas a | taxID used:135614 OG02979|WP\_045757159.1 | hypothetical protein [Xanthomonas a | taxID used:135614 OG00297|WP\_012917566.1 | hypothetical protein [Xanthomonas a | taxID used:135614 OG02980|WP\_012917041.1 | cupin domain-containing protein [Xa | taxID used:135614 OG02981|WP\_010342859.1 | MULTISPECIES: 50S ribosomal protein | taxID used:135614 OG02985|WP\_012915052.1 | 50S ribosomal protein L7/L12 [Xanth | taxID used:135614 OG02988|WP\_012916188.1 | hypothetical protein [Xanthomonas a | taxID used:135614 OG00298|WP\_045757162.1 | TonB-dependent receptor [Xanthomona | taxID used:135614 OG02990|WP\_010340453.1 | MULTISPECIES: 30S ribosomal protein | taxID used:135614 OG02992|WP\_012915671.1 | cell division protein FtsB [Xanthom | taxID used:135614 OG02993|WP\_045756987.1 | cysteine methyltransferase [Xanthom | taxID used:135614 OG02995|WP\_012915319.1 | PilZ domain-containing protein [Xan | taxID used:135614 OG02997|WP\_012914673.1 | membrane protein [Xanthomonas albil | taxID used:135614 OG00299|WP\_012915893.1 | flagellar biosynthesis protein FlhA | taxID used:135614 OG00029|WP\_045758799.1 | PAS domain S-box protein [Xanthomon | taxID used:135614 OG03000|WP\_012915407.1 | nucleotide pyrophosphohydrolase [Xa | taxID used:135614 OG03005|WP\_012915072.1 | MULTISPECIES: 50S ribosomal protein | taxID used:135614 OG03006|WP\_012916983.1 | YkgJ family cysteine cluster protei | taxID used:135614 OG03007|WP\_012914831.1 | Na+/H+ antiporter subunit C [Xantho | taxID used:135614 OG03008|WP\_012916681.1 | DUF1304 domain-containing protein [ | taxID used:135614 OG03009|WP\_012915162.1 | tfp pilus assembly protein [Xanthom | taxID used:135614 OG00300|WP\_012915336.1 | penicillin-binding protein 2 [Xanth | taxID used:135614 OG03010|WP\_045756959.1 | transcriptional regulator [Xanthomo | taxID used:135614 OG03011|WP\_045757027.1 | hypothetical protein [Xanthomonas a | taxID used:135614 OG03014|WP\_012917001.1 | hypothetical protein [Xanthomonas a | taxID used:135614 OG03015|WP\_012916103.1 | integration host factor subunit bet | taxID used:135614 OG03017|WP\_012916114.1 | 4a-hydroxytetrahydrobiopterin dehyd | taxID used:135614 OG03019|WP\_012915144.1 | histidine triad nucleotide-binding | taxID used:135614 OG00301|WP\_045756754.1 | cytochrome o ubiquinol oxidase subu | taxID used:135614 OG03020|WP\_012916489.1 | XRE family transcriptional regulato | taxID used:135614 OG03021|WP\_012916976.1 | RidA family protein [Xanthomonas al | taxID used:135614 OG03022|WP\_012915782.1 | MULTISPECIES: DUF3301 domain-contai | taxID used:135614 OG03023|WP\_045757290.1 | iron-sulfur cluster assembly access | taxID used:135614 OG03024|WP\_012915459.1 | cytochrome o ubiquinol oxidase subu | taxID used:135614 OG03028|WP\_012917351.1 | thioredoxin TrxA, partial [Xanthomo | taxID used:135614 OG03029|WP\_012916416.1 | preprotein translocase subunit YajC | taxID used:135614 OG00302|WP\_012917022.1 | 3-hydroxyacyl-CoA dehydrogenase [Xa | taxID used:135614 OG03030|WP\_012915548.1 | non-heme iron oxygenase ferredoxin | taxID used:135614 OG03031|WP\_012915392.1 | hypothetical protein [Xanthomonas a | taxID used:135614 OG03033|WP\_012916481.1 | hypothetical protein [Xanthomonas a | taxID used:135614 OG03036|WP\_012914876.1 | divalent-cation tolerance protein C | taxID used:135614 OG03038|WP\_012916746.1 | Rieske (2Fe-2S) protein [Xanthomona | taxID used:135614 OG00303|WP\_012914626.1 | 9-O-acetylesterase [Xanthomonas alb | taxID used:135614 OG03040|WP\_012916615.1 | hypothetical protein [Xanthomonas a | taxID used:135614 OG03042|WP\_012915033.1 | transcriptional regulator [Xanthomo | taxID used:135614 OG03043|WP\_012914836.1 | J domain-containing protein [Xantho | taxID used:135614 OG03045|WP\_012915943.1 | flagellar protein FlgN [Xanthomonas | taxID used:135614 OG03048|WP\_012915703.1 | ferredoxin family protein [Xanthomo | taxID used:135614 OG03049|WP\_012914919.1 | DUF3240 domain-containing protein [ | taxID used:135614 OG00304|WP\_012915095.1 | NADPH-dependent 2,4-dienoyl-CoA red | taxID used:135614 OG03051|WP\_012914727.1 | thioredoxin [Xanthomonas albilinean | taxID used:135614 OG03052|WP\_012915899.1 | flagellar motor switch protein FliN | taxID used:135614 OG03053|WP\_012916106.1 | hypothetical protein [Xanthomonas a | taxID used:135614 OG03056|WP\_012917562.1 | MULTISPECIES: nitrogen regulatory p | taxID used:135614 OG03058|WP\_045756821.1 | ATP-dependent Clp protease adapter | taxID used:135614 OG00305|WP\_080929784.1 | PAS domain S-box protein [Xanthomon | taxID used:135614 OG03060|WP\_012917213.1 | hypothetical protein [Xanthomonas a | taxID used:135614 OG03061|WP\_012916910.1 | zinc ribbon domain-containing prote | taxID used:135614 OG03065|WP\_012916327.1 | hypothetical protein [Xanthomonas a | taxID used:135614 OG03066|WP\_045756765.1 | DUF3175 domain-containing protein [ | taxID used:135614 OG03067|WP\_003486710.1 | MULTISPECIES: 50S ribosomal protein | taxID used:135614 OG03068|WP\_012915639.1 | GNAT family N-acetyltransferase [Xa | taxID used:135614 OG00306|WP\_045757347.1 | peptidase M1 [Xanthomonas albilinea | taxID used:135614 OG03072|WP\_012915788.1 | MULTISPECIES: monothiol glutaredoxi | taxID used:135614 OG03074|WP\_045757042.1 | hypothetical protein [Xanthomonas a | taxID used:135614 OG03076|WP\_012916601.1 | comea-related DNA uptake protein [X | taxID used:135614 OG03078|WP\_045756693.1 | DUF1820 domain-containing protein [ | taxID used:135614 OG00307|WP\_012914712.1 | acetyl/propionyl/methylcrotonyl-CoA | taxID used:135614 OG03080|WP\_045756924.1 | hypothetical protein [Xanthomonas a | taxID used:135614 OG03083|WP\_080928105.1 | hypothetical protein [Xanthomonas a | taxID used:135614 OG03085|WP\_012915142.1 | nucleoid-associated protein, YbaB/E | taxID used:135614 OG03089|WP\_012916624.1 | ribosome-associated translation inh | taxID used:135614 OG00308|WP\_012915301.1 | phosphatase PAP2 family protein [Xa | taxID used:135614 OG03091|WP\_012917544.1 | DUF4156 domain-containing protein [ | taxID used:135614 OG03092|WP\_012915569.1 | NAD(P) transhydrogenase subunit alp | taxID used:135614 OG03096|WP\_012914658.1 | DUF485 domain-containing protein [X | taxID used:135614 OG03097|WP\_012915444.1 | MULTISPECIES: 50S ribosomal protein | taxID used:135614 OG00030|WP\_012916933.1 | TIGR02099 family protein [Xanthomon | taxID used:135614 OG03102|WP\_012915942.1 | flagellar biosynthesis anti-sigma f | taxID used:135614 OG03104|WP\_012915067.1 | MULTISPECIES: 50S ribosomal protein | taxID used:135614 OG03105|WP\_045756707.1 | NAD(P) transhydrogenase subunit alp | taxID used:135614 OG03106|WP\_012916236.1 | hypothetical protein [Xanthomonas a | taxID used:135614 OG03107|WP\_012915925.1 | hypothetical protein [Xanthomonas a | taxID used:135614 OG00310|WP\_012917199.1 | oligopeptidase A [Xanthomonas albil | taxID used:135614 OG03110|WP\_012915839.1 | DUF2388 domain-containing protein [ | taxID used:135614 OG03111|WP\_010341589.1 | MULTISPECIES: 30S ribosomal protein | taxID used:135614 OG03118|WP\_045756914.1 | ferredoxin [Xanthomonas albilineans | taxID used:135614 OG03119|WP\_012915950.1 | hypothetical protein [Xanthomonas a | taxID used:135614 OG00311|WP\_012915491.1 | M13 family peptidase [Xanthomonas a | taxID used:135614 OG03121|WP\_012915069.1 | 30S ribosomal protein S14 [Xanthomo | taxID used:135614 OG03122|WP\_045756986.1 | hypothetical protein [Xanthomonas a | taxID used:135614 OG03123|WP\_012916235.1 | hypothetical protein [Xanthomonas a | taxID used:135614 OG03124|WP\_012914596.1 | STAS domain-containing protein [Xan | taxID used:135614 OG03125|WP\_012916084.1 | DUF1244 domain-containing protein [ | taxID used:135614 OG03126|WP\_012917610.1 | transcriptional regulator [Xanthomo | taxID used:135614 OG03127|WP\_012917055.1 | hypothetical protein [Xanthomonas a | taxID used:135614 OG03128|WP\_012916441.1 | hypothetical protein [Xanthomonas a | taxID used:135614 OG00312|WP\_012916815.1 | transketolase [Xanthomonas albiline | taxID used:135614 OG03132|WP\_012915681.1 | ribosome assembly RNA-binding prote | taxID used:135614 OG03137|WP\_012915823.1 | hypothetical protein [Xanthomonas a | taxID used:135614 OG03139|WP\_012917486.1 | hypothetical protein [Xanthomonas a | taxID used:135614 OG03140|WP\_012915326.1 | DNA-directed RNA polymerase subunit | taxID used:135614 OG03141|WP\_012915599.1 | integration host factor subunit alp | taxID used:135614 OG03142|WP\_003470672.1 | MULTISPECIES: 50S ribosomal protein | taxID used:135614 OG03143|WP\_012915534.1 | MULTISPECIES: NADH-quinone oxidored | taxID used:135614 OG03145|WP\_012915765.1 | Trp operon repressor [Xanthomonas a | taxID used:135614 OG03147|WP\_012914681.1 | DUF2782 domain-containing protein [ | taxID used:135614 OG03155|WP\_012917415.1 | DUF4031 domain-containing protein [ | taxID used:135614 OG03158|WP\_012915313.1 | cell division protein ZapA [Xanthom | taxID used:135614 OG00315|WP\_045757410.1 | K(+)-transporting ATPase subunit B | taxID used:135614 OG03161|WP\_012914695.1 | dehydratase [Xanthomonas albilinean | taxID used:135614 OG03164|WP\_012914881.1 | GIY-YIG nuclease family protein [Xa | taxID used:135614 OG03166|WP\_012915734.1 | DUF4190 domain-containing protein [ | taxID used:135614 OG03167|WP\_012914828.1 | K+/H+ antiporter subunit F [Xanthom | taxID used:135614 OG00316|WP\_012916721.1 | methyl-accepting chemotaxis protein | taxID used:135614 OG03172|WP\_012916562.1 | oxidative damage protection protein | taxID used:135614 OG03175|WP\_012915824.1 | BolA family transcriptional regulat | taxID used:135614 OG03176|WP\_045756672.1 | hypothetical protein [Xanthomonas a | taxID used:135614 OG00317|WP\_012916360.1 | excinuclease ABC subunit UvrB [Xant | taxID used:135614 OG03180|WP\_080929717.1 | membrane protein insertion efficien | taxID used:135614 OG03184|WP\_012914877.1 | co-chaperone GroES [Xanthomonas alb | taxID used:135614 OG03187|WP\_012915343.1 | hypothetical protein [Xanthomonas a | taxID used:135614 OG03189|WP\_080931578.1 | hypothetical protein [Xanthomonas a | taxID used:135614 OG03195|WP\_012916102.1 | DUF1049 domain-containing protein [ | taxID used:135614 OG00319|WP\_012917399.1 | hybrid sensor histidine kinase/resp | taxID used:135614 OG00031|WP\_045756808.1 | hypothetical protein [Xanthomonas a | taxID used:135614 OG03203|WP\_012915686.1 | RNA chaperone Hfq [Xanthomonas albi | taxID used:135614 OG03209|WP\_045761604.1 | hypothetical protein [Xanthomonas a | taxID used:135614 OG00320|WP\_012916210.1 | short chain dehydrogenase [Xanthomo | taxID used:135614 OG03212|WP\_012915061.1 | MULTISPECIES: 30S ribosomal protein | taxID used:135614 OG03215|WP\_012916205.1 | MULTISPECIES: polyhydroxyalkanoic a | taxID used:135614 OG03219|WP\_012914868.1 | DNA-binding transcriptional regulat | taxID used:135614 OG00321|WP\_012917570.1 | phospholipase D family protein [Xan | taxID used:135614 OG03226|WP\_012915065.1 | 30S ribosomal protein S17 [Xanthomo | taxID used:135614 OG03231|WP\_012916747.1 | hypothetical protein [Xanthomonas a | taxID used:135614 OG03235|WP\_012916243.1 | hypothetical protein [Xanthomonas a | taxID used:135614 OG03236|WP\_012916585.1 | hypothetical protein [Xanthomonas a | taxID used:135614 OG03237|WP\_045756635.1 | hypothetical protein [Xanthomonas a | taxID used:135614 OG03239|WP\_012915447.1 | 30S ribosomal protein S20 [Xanthomo | taxID used:135614 OG00323|WP\_012915125.1 | peptidylprolyl isomerase [Xanthomon | taxID used:135614 OG03240|WP\_012916927.1 | exodeoxyribonuclease VII small subu | taxID used:135614 OG03243|WP\_012916603.1 | GlsB/YeaQ/YmgE family stress respon | taxID used:135614 OG03245|WP\_012917290.1 | cell division protein FtsL [Xanthom | taxID used:135614 OG03248|WP\_009607753.1 | MULTISPECIES: HU family DNA-binding | taxID used:135614 OG03249|WP\_012917397.1 | hypothetical protein [Xanthomonas a | taxID used:135614 OG03250|WP\_012914691.1 | acyl carrier protein [Xanthomonas a | taxID used:135614 OG03252|WP\_012916540.1 | hypothetical protein [Xanthomonas a | taxID used:135614 OG03253|WP\_012916628.1 | HPr family phosphocarrier protein [ | taxID used:135614 OG03258|WP\_012914918.1 | hypothetical protein [Xanthomonas a | taxID used:135614 OG03259|WP\_012915896.1 | flagellar biosynthetic protein FliQ | taxID used:135614 OG03260|WP\_045757459.1 | hypothetical protein [Xanthomonas a | taxID used:135614 OG03263|WP\_012915479.1 | 30S ribosomal protein S16 [Xanthomo | taxID used:135614 OG03266|WP\_012915784.1 | hypothetical protein [Xanthomonas a | taxID used:135614 OG03267|WP\_012914706.1 | YcgL domain-containing protein [Xan | taxID used:135614 OG03269|WP\_012916653.1 | WGR domain-containing protein [Xant | taxID used:135614 OG00326|WP\_045756683.1 | HAMP domain-containing protein [Xan | taxID used:135614 OG03272|WP\_012916276.1 | acyl carrier protein [Xanthomonas a | taxID used:135614 OG03276|WP\_003467392.1 | MULTISPECIES: 30S ribosomal protein | taxID used:135614 OG03277|WP\_012915445.1 | 50S ribosomal protein L27 [Xanthomo | taxID used:135614 OG00327|WP\_012914731.1 | hypothetical protein [Xanthomonas a | taxID used:135614 OG03281|WP\_012916376.1 | hypothetical protein [Xanthomonas a | taxID used:135614 OG03286|WP\_012916313.1 | succinate dehydrogenase assembly fa | taxID used:135614 OG00328|WP\_012917636.1 | ATP-dependent DNA helicase Rep [Xan | taxID used:135614 OG03293|WP\_012916450.1 | (2Fe-2S)-binding protein [Xanthomon | taxID used:135614 OG03294|WP\_012916218.1 | acyl-CoA-binding protein [Xanthomon | taxID used:135614 OG03297|WP\_012915847.1 | hypothetical protein [Xanthomonas a | taxID used:135614 OG03299|WP\_012915421.1 | cell division topological specifici | taxID used:135614 OG00329|WP\_012917067.1 | 4-alpha-glucanotransferase [Xanthom | taxID used:135614 OG00032|WP\_045757458.1 | translocation/assembly module TamB | taxID used:135614 OG03301|WP\_012916181.1 | RnfH family protein [Xanthomonas al | taxID used:135614 OG03302|WP\_012914665.1 | GlsB/YeaQ/YmgE family stress respon | taxID used:135614 OG03303|WP\_012915861.1 | AbrB/MazE/SpoVT family DNA-binding | taxID used:135614 OG03304|WP\_012916835.1 | type B 50S ribosomal protein L31 [X | taxID used:135614 OG03307|WP\_012915285.1 | acetolactate synthase [Xanthomonas | taxID used:135614 OG03308|WP\_045757044.1 | DUF3297 domain-containing protein [ | taxID used:135614 OG00330|WP\_045756744.1 | murein transglycosylase [Xanthomona | taxID used:135614 OG03312|WP\_012915623.1 | cold-shock protein [Xanthomonas alb | taxID used:135614 OG03319|WP\_080928191.1 | hypothetical protein [Xanthomonas a | taxID used:135614 OG03324|WP\_012915139.1 | MoaD/ThiS family protein [Xanthomon | taxID used:135614 OG00332|WP\_012914677.1 | peptidase M61 [Xanthomonas albiline | taxID used:135614 OG03334|WP\_012916617.1 | BolA family transcriptional regulat | taxID used:135614 OG03335|WP\_010342733.1 | MULTISPECIES: 50S ribosomal protein | taxID used:135614 OG03337|WP\_012916556.1 | putative selenoprotein [Xanthomonas | taxID used:135614 OG03338|WP\_045757003.1 | hypothetical protein [Xanthomonas a | taxID used:135614 OG03340|WP\_012915269.1 | XRE family transcriptional regulato | taxID used:135614 OG03342|WP\_012915156.1 | MULTISPECIES: acyl carrier protein | taxID used:135614 OG00334|WP\_012915509.1 | hypothetical protein [Xanthomonas a | taxID used:135614 OG03355|WP\_012916152.1 | hypothetical protein [Xanthomonas a | taxID used:135614 OG03357|WP\_045756937.1 | glutaredoxin family protein [Xantho | taxID used:135614 OG03359|WP\_002804494.1 | MULTISPECIES: 30S ribosomal protein | taxID used:135614 OG03360|WP\_012914636.1 | twin-arginine translocase subunit T | taxID used:135614 OG03364|WP\_012915588.1 | antibiotic synthesis protein MbtH [ | taxID used:135614 OG03369|WP\_045757438.1 | hypothetical protein [Xanthomonas a | taxID used:135614 OG00336|WP\_012916373.1 | glutaryl-7-ACA acylase [Xanthomonas | taxID used:135614 OG03373|WP\_080928076.1 | ferrous iron transport protein A [X | taxID used:135614 OG03375|WP\_012915650.1 | cold-shock protein [Xanthomonas alb | taxID used:135614 OG03377|WP\_012915918.1 | acyl carrier protein [Xanthomonas a | taxID used:135614 OG03378|WP\_012917433.1 | hypothetical protein [Xanthomonas a | taxID used:135614 OG00337|WP\_012914657.1 | acetate--CoA ligase [Xanthomonas al | taxID used:135614 OG03380|WP\_012915005.1 | DUF2007 domain-containing protein [ | taxID used:135614 OG03382|WP\_004425677.1 | MULTISPECIES: translation initiatio | taxID used:135614 OG03383|WP\_045757024.1 | DUF465 domain-containing protein [X | taxID used:135614 OG03387|WP\_012914938.1 | hypothetical protein [Xanthomonas a | taxID used:135614 OG00338|WP\_012916536.1 | RNA helicase [Xanthomonas albilinea | taxID used:135614 OG03391|WP\_012915971.1 | heme exporter protein CcmD [Xanthom | taxID used:135614 OG03392|WP\_012915314.1 | TIGR02449 family protein [Xanthomon | taxID used:135614 OG03395|WP\_012916537.1 | RNA-binding S4 domain-containing pr | taxID used:135614 OG03397|WP\_012915693.1 | carbon storage regulator [Xanthomon | taxID used:135614 OG03399|WP\_003465342.1 | MULTISPECIES: 30S ribosomal protein | taxID used:135614 OG00339|WP\_012915880.1 | chemotaxis protein CheA [Xanthomona | taxID used:135614 OG03406|WP\_012914958.1 | twin transmembrane helix small prot | taxID used:135614 OG00340|WP\_080928131.1 | acetyl-CoA hydrolase [Xanthomonas a | taxID used:135614 OG03410|WP\_012914854.1 | bacterioferritin [Xanthomonas albil | taxID used:135614 OG00341|WP\_012915146.1 | DUF3488 domain-containing protein [ | taxID used:135614 OG03428|WP\_012915547.1 | hypothetical protein [Xanthomonas a | taxID used:135614 OG03430|WP\_012915270.1 | zinc-finger domain-containing prote | taxID used:135614 OG03438|WP\_012915638.1 | hypothetical protein [Xanthomonas a | taxID used:135614 OG00343|WP\_012916116.1 | DUF3300 domain-containing protein [ | taxID used:135614 OG03441|WP\_045757037.1 | hypothetical protein [Xanthomonas a | taxID used:135614 OG03442|WP\_012915307.1 | rubredoxin [Xanthomonas albilineans | taxID used:135614 OG03443|WP\_045757125.1 | DUF465 domain-containing protein [X | taxID used:135614 OG03445|WP\_012917069.1 | DUF2934 domain-containing protein [ | taxID used:135614 OG00344|WP\_012917063.1 | glucans biosynthesis glucosyltransf | taxID used:135614 OG03454|WP\_012917249.1 | hemin uptake protein HemP [Xanthomo | taxID used:135614 OG03456|WP\_010342861.1 | MULTISPECIES: 50S ribosomal protein | taxID used:135614 OG03459|WP\_012916742.1 | sulfur carrier protein ThiS [Xantho | taxID used:135614 OG00345|WP\_012915595.1 | threonine--tRNA ligase [Xanthomonas | taxID used:135614 OG03460|WP\_012916488.1 | hypothetical protein [Xanthomonas a | taxID used:135614 OG03464|WP\_012916979.1 | oxidoreductase-like protein [Xantho | taxID used:135614 OG03465|WP\_012915152.1 | 50S ribosomal protein L32 [Xanthomo | taxID used:135614 OG03466|WP\_012916241.1 | hypothetical protein [Xanthomonas a | taxID used:135614 OG03468|WP\_012916984.1 | hypothetical protein [Xanthomonas a | taxID used:135614 OG00346|WP\_012915710.1 | 9-O-acetylesterase [Xanthomonas alb | taxID used:135614 OG03478|WP\_012916185.1 | hypothetical protein [Xanthomonas a | taxID used:135614 OG00347|WP\_045757001.1 | M1 family peptidase [Xanthomonas al | taxID used:135614 OG03487|WP\_012915074.1 | 50S ribosomal protein L30 [Xanthomo | taxID used:135614 OG03491|WP\_012915064.1 | MULTISPECIES: 50S ribosomal protein | taxID used:135614 OG00034|WP\_012914590.1 | exodeoxyribonuclease V subunit beta | taxID used:135614 OG03502|WP\_012916543.1 | 30S ribosomal protein THX [Xanthomo | taxID used:135614 OG00350|WP\_012915683.1 | ATP-dependent metallopeptidase FtsH | taxID used:135614 OG03512|WP\_012916318.1 | DUF1674 domain-containing protein [ | taxID used:135614 OG03514|WP\_012915806.1 | heme exporter protein CcmD [Xanthom | taxID used:135614 OG03518|WP\_005411637.1 | MULTISPECIES: 50S ribosomal protein | taxID used:135614 OG03520|WP\_080929708.1 | DUF3060 domain-containing protein [ | taxID used:135614 OG03526|WP\_012914816.1 | MULTISPECIES: entericidin, EcnA/B f | taxID used:135614 OG00352|WP\_012915258.1 | amylosucrase [Xanthomonas albilinea | taxID used:135614 OG00353|WP\_012915591.1 | oligopeptide transporter, OPT famil | taxID used:135614 OG00354|WP\_012916959.1 | DUF885 domain-containing protein [X | taxID used:135614 OG00355|WP\_012916903.1 | potassium transporter Kup [Xanthomo | taxID used:135614 OG00356|WP\_012917591.1 | PAS domain-containing protein [Xant | taxID used:135614 OG00357|WP\_012915804.1 | heme lyase CcmF/NrfE family subunit | taxID used:135614 OG00359|WP\_012914863.1 | MFS transporter [Xanthomonas albili | taxID used:135614 OG00360|WP\_012915460.1 | peptidase M61 [Xanthomonas albiline | taxID used:135614 OG00361|WP\_012914857.1 | GGDEF domain-containing response re | taxID used:135614 OG00362|WP\_045757192.1 | phosphomethylpyrimidine synthase Th | taxID used:135614 OG00363|WP\_012914589.1 | exodeoxyribonuclease V subunit alph | taxID used:135614 OG00365|WP\_012916324.1 | phosphogluconate dehydratase [Xanth | taxID used:135614 OG00366|WP\_012915603.1 | 1-deoxy-D-xylulose-5-phosphate synt | taxID used:135614 OG00367|WP\_012916099.1 | polysaccharide biosynthesis protein | taxID used:135614 OG00368|WP\_012916431.1 | molecular chaperone HtpG [Xanthomon | taxID used:135614 OG00369|WP\_045757365.1 | adenylyl-sulfate kinase [Xanthomona | taxID used:135614 OG00036|WP\_045757436.1 | indolepyruvate ferredoxin oxidoredu | taxID used:135614 OG00371|WP\_012914887.1 | peptidase S53 [Xanthomonas albiline | taxID used:135614 OG00372|WP\_045757246.1 | DUF4105 domain-containing protein [ | taxID used:135614 OG00374|WP\_012916175.1 | molecular chaperone DnaK [Xanthomon | taxID used:135614 OG00375|WP\_012914921.1 | arginine decarboxylase [Xanthomonas | taxID used:135614 OG00377|WP\_012914906.1 | tRNA uridine-5-carboxymethylaminome | taxID used:135614 OG00379|WP\_012915666.1 | DNA topoisomerase IV subunit B [Xan | taxID used:135614 OG00380|WP\_012917340.1 | signal peptide peptidase SppA [Xant | taxID used:135614 OG00381|WP\_012917135.1 | protease [Xanthomonas albilineans] | taxID used:135614 OG00382|WP\_012916706.1 | beta-galactosidase [Xanthomonas alb | taxID used:135614 OG00383|WP\_012915646.1 | ABC transporter ATP-binding protein | taxID used:135614 OG00384|WP\_045757228.1 | ABC transporter ATPase [Xanthomonas | taxID used:135614 OG00385|WP\_012914651.1 | sulfotransferase family protein [Xa | taxID used:135614 OG00386|WP\_012917514.1 | feruloyl-CoA synthase [Xanthomonas | taxID used:135614 OG00387|WP\_045756968.1 | type IV pilus secretin PilQ [Xantho | taxID used:135614 OG00388|WP\_012917196.1 | TonB-dependent vitamin B12 receptor | taxID used:135614 OG00389|WP\_012916445.1 | DUF885 domain-containing protein [X | taxID used:135614 OG00038|WP\_012916524.1 | DNA polymerase III subunit alpha [X | taxID used:135614 OG00391|WP\_012917316.1 | RNA polymerase sigma factor RpoD [X | taxID used:135614 OG00392|WP\_012917289.1 | cell division protein [Xanthomonas | taxID used:135614 OG00393|WP\_012915613.1 | DNA mismatch repair endonuclease Mu | taxID used:135614 OG00394|WP\_012915930.1 | flagellar hook-associated protein F | taxID used:135614 OG00395|WP\_012916745.1 | SLC13 family permease [Xanthomonas | taxID used:135614 OG00396|WP\_045757338.1 | formylglycine-generating enzyme fam | taxID used:135614 OG00398|WP\_012916915.1 | glutathione ABC transporter ATP-bin | taxID used:135614 OG00399|WP\_012916301.1 | excinuclease ABC subunit UvrC [Xant | taxID used:135614 OG00402|WP\_012915846.1 | ferrous iron transport protein B [X | taxID used:135614 OG00403|WP\_012917455.1 | dihydroxy-acid dehydratase [Xanthom | taxID used:135614 OG00404|WP\_012915149.1 | membrane protein [Xanthomonas albil | taxID used:135614 OG00406|WP\_012916822.1 | tetratricopeptide repeat protein [X | taxID used:135614 OG00407|WP\_012916741.1 | autotransporter domain-containing e | taxID used:135614 OG00408|WP\_012917066.1 | malto-oligosyltrehalose trehalohydr | taxID used:135614 OG00409|WP\_012915084.1 | translational GTPase TypA [Xanthomo | taxID used:135614 OG00410|WP\_012915141.1 | DNA polymerase III subunit gamma/ta | taxID used:135614 OG00411|WP\_012917363.1 | glutamine--fructose-6-phosphate tra | taxID used:135614 OG00412|WP\_045756769.1 | hypothetical protein [Xanthomonas a | taxID used:135614 OG00413|WP\_012915288.1 | methyl-accepting chemotaxis protein | taxID used:135614 OG00416|WP\_012917453.1 | acid phosphatase [Xanthomonas albil | taxID used:135614 OG00417|WP\_012915357.1 | PAS domain S-box protein [Xanthomon | taxID used:135614 OG00418|WP\_012916315.1 | succinate dehydrogenase flavoprotei | taxID used:135614 OG00419|WP\_012916739.1 | DNA helicase RecQ [Xanthomonas albi | taxID used:135614 OG00041|WP\_080931585.1 | O-antigen biosynthesis protein [Xan | taxID used:135614 OG00420|WP\_012914642.1 | type II/IV secretion system protein | taxID used:135614 OG00421|WP\_012915249.1 | siderophore biosynthesis protein, I | taxID used:135614 OG00423|WP\_012916415.1 | protein translocase subunit SecD [X | taxID used:135614 OG00424|WP\_012915226.1 | glycoside hydrolase family 15 prote | taxID used:135614 OG00425|WP\_012914709.1 | potassium transporter KefB [Xanthom | taxID used:135614 OG00426|WP\_012917607.1 | outer membrane protein assembly fac | taxID used:135614 OG00428|WP\_012917018.1 | elongation factor 4 [Xanthomonas al | taxID used:135614 OG00432|WP\_012916021.1 | carbamoyltransferase [Xanthomonas a | taxID used:135614 OG00433|WP\_012915605.1 | acyl-CoA dehydrogenase [Xanthomonas | taxID used:135614 OG00436|WP\_012915714.1 | hypothetical protein [Xanthomonas a | taxID used:135614 OG00438|WP\_012915004.1 | glutamine--tRNA ligase/YqeY domain | taxID used:135614 OG00439|WP\_080928196.1 | sensor domain-containing phosphodie | taxID used:135614 OG00043|WP\_012916229.1 | chromosome segregation protein SMC | taxID used:135614 OG00440|WP\_012917384.1 | dihydrolipoyl dehydrogenase [Xantho | taxID used:135614 OG00441|WP\_012915247.1 | iron transporter [Xanthomonas albil | taxID used:135614 OG00446|WP\_012915948.1 | sulfonate ABC transporter permease | taxID used:135614 OG00447|WP\_012916421.1 | cellulase [Xanthomonas albilineans] | taxID used:135614 OG00448|WP\_012916909.1 | aspartate--tRNA ligase [Xanthomonas | taxID used:135614 OG00449|WP\_012917643.1 | membrane protein insertase YidC [Xa | taxID used:135614 OG00044|WP\_012917519.1 | type I-F CRISPR-associated helicase | taxID used:135614 OG00450|WP\_012915590.1 | MFS transporter [Xanthomonas albili | taxID used:135614 OG00451|WP\_012916426.1 | gamma-glutamyltransferase [Xanthomo | taxID used:135614 OG00453|WP\_045757038.1 | transcription termination factor Rh | taxID used:135614 OG00458|WP\_012916736.1 | chloride channel protein [Xanthomon | taxID used:135614 OG00045|WP\_012917598.1 | ATP-binding protein [Xanthomonas al | taxID used:135614 OG00460|WP\_012916821.1 | membrane protein [Xanthomonas albil | taxID used:135614 OG00461|WP\_012916172.1 | ABC transporter ATP-binding protein | taxID used:135614 OG00462|WP\_012915464.1 | dihydroxy-acid dehydratase [Xanthom | taxID used:135614 OG00463|WP\_012917302.1 | potassium-transporting ATPase subun | taxID used:135614 OG00464|WP\_012916699.1 | alpha-L-fucosidase [Xanthomonas alb | taxID used:135614 OG00465|WP\_012915286.1 | acetolactate synthase 2 catalytic s | taxID used:135614 OG00466|WP\_012914966.1 | DNA primase [Xanthomonas albilinean | taxID used:135614 OG00468|WP\_012915036.1 | UDP-N-acetylglucosamine--peptide N- | taxID used:135614 OG00469|WP\_012915945.1 | c-di-GMP phosphodiesterase A [Xanth | taxID used:135614 OG00046|WP\_012914650.1 | hybrid sensor histidine kinase/resp | taxID used:135614 OG00470|WP\_012917325.1 | dolichyl-phosphate-mannose--protein | taxID used:135614 OG00471|WP\_045756675.1 | hypothetical protein [Xanthomonas a | taxID used:135614 OG00473|WP\_012915853.1 | single-stranded-DNA-specific exonuc | taxID used:135614 OG00474|WP\_012915195.1 | type IV-A pilus assembly ATPase Pil | taxID used:135614 OG00476|WP\_012915887.1 | chemotaxis protein CheA [Xanthomona | taxID used:135614 OG00479|WP\_045756909.1 | polygalacturonase [Xanthomonas albi | taxID used:135614 OG00047|WP\_045757122.1 | exodeoxyribonuclease V subunit gamm | taxID used:135614 OG00480|WP\_012915587.1 | cyclic peptide export ABC transport | taxID used:135614 OG00481|WP\_012917442.1 | alpha,alpha-trehalase TreA [Xanthom | taxID used:135614 OG00483|WP\_012914659.1 | cation acetate symporter [Xanthomon | taxID used:135614 OG00484|WP\_012917134.1 | type II secretion system protein Gs | taxID used:135614 OG00485|WP\_012916499.1 | asparagine synthase B [Xanthomonas | taxID used:135614 OG00486|WP\_012914624.1 | glycoside hydrolase family 43 prote | taxID used:135614 OG00487|WP\_012914701.1 | tannase/feruloyl esterase family al | taxID used:135614 OG00488|WP\_012915907.1 | flagellar M-ring protein FliF [Xant | taxID used:135614 OG00489|WP\_012914929.1 | arginine--tRNA ligase [Xanthomonas | taxID used:135614 OG00490|WP\_012917150.1 | proline--tRNA ligase [Xanthomonas a | taxID used:135614 OG00491|WP\_012917002.1 | GGDEF domain-containing protein [Xa | taxID used:135614 OG00492|WP\_012915859.1 | long-chain fatty acid--CoA ligase [ | taxID used:135614 OG00496|WP\_012917350.1 | ATP-dependent RNA helicase RhlB [Xa | taxID used:135614 OG00497|WP\_045756882.1 | urocanate hydratase [Xanthomonas al | taxID used:135614 OG00498|WP\_012915416.1 | phosphoethanolamine transferase [Xa | taxID used:135614 OG00499|WP\_080931588.1 | hypothetical protein [Xanthomonas a | taxID used:135614 OG00501|WP\_012916629.1 | phosphoenolpyruvate--protein phosph | taxID used:135614 OG00502|WP\_012917385.1 | dihydrolipoyllysine-residue acetylt | taxID used:135614 OG00503|WP\_012917158.1 | electron transfer flavoprotein-ubiq | taxID used:135614 OG00504|WP\_012915825.1 | hypothetical protein [Xanthomonas a | taxID used:135614 OG00505|WP\_012914954.1 | cytochrome c oxidase subunit I [Xan | taxID used:135614 OG00506|WP\_012915668.1 | CTP synthase [Xanthomonas albilinea | taxID used:135614 OG00507|WP\_012916104.1 | 30S ribosomal protein S1 [Xanthomon | taxID used:135614 OG00508|WP\_045757033.1 | energy-dependent translational thro | taxID used:135614 OG00050|WP\_012915814.1 | hybrid sensor histidine kinase/resp | taxID used:135614 OG00510|WP\_012914702.1 | NAD(P)/FAD-dependent oxidoreductase | taxID used:135614 OG00511|WP\_012917536.1 | ubiquinone biosynthesis regulatory | taxID used:135614 OG00512|WP\_045757133.1 | alkaline phosphatase [Xanthomonas a | taxID used:135614 OG00514|WP\_012917539.1 | peptide synthase [Xanthomonas albil | taxID used:135614 OG00516|WP\_012915216.1 | NAD+ synthase [Xanthomonas albiline | taxID used:135614 OG00517|WP\_012915253.1 | Kef family K(+) transporter [Xantho | taxID used:135614 OG00518|WP\_012915039.1 | tetratricopeptide repeat protein [X | taxID used:135614 OG00519|WP\_012917633.1 | glucan biosynthesis protein D [Xant | taxID used:135614 OG00051|WP\_045757242.1 | Oar protein [Xanthomonas albilinean | taxID used:135614 OG00520|WP\_012916178.1 | DNA repair protein RecN [Xanthomona | taxID used:135614 OG00521|WP\_012916437.1 | glycosyl hydrolase family 43 [Xanth | taxID used:135614 OG00524|WP\_012915116.1 | M48 family peptidase [Xanthomonas a | taxID used:135614 OG00525|WP\_012916136.1 | hybrid sensor histidine kinase/resp | taxID used:135614 OG00527|WP\_045756748.1 | rhamnogalacturonase B [Xanthomonas | taxID used:135614 OG00529|WP\_012915473.1 | alpha-L-arabinofuranosidase [Xantho | taxID used:135614 OG00052|WP\_012914707.1 | hypothetical protein [Xanthomonas a | taxID used:135614 OG00530|WP\_012916433.1 | glycoside hydrolase family 27 prote | taxID used:135614 OG00531|WP\_012916682.1 | choline dehydrogenase [Xanthomonas | taxID used:135614 OG00532|WP\_012915502.1 | lytic transglycosylase [Xanthomonas | taxID used:135614 OG00533|WP\_045757007.1 | glycogen synthase GlgA [Xanthomonas | taxID used:135614 OG00534|WP\_012914878.1 | chaperonin GroEL [Xanthomonas albil | taxID used:135614 OG00535|WP\_012914713.1 | methylcrotonoyl-CoA carboxylase [Xa | taxID used:135614 OG00536|WP\_012917422.1 | Na+/H+ antiporter [Xanthomonas albi | taxID used:135614 OG00537|WP\_012915197.1 | two-component sensor histidine kina | taxID used:135614 OG00538|WP\_012916671.1 | peptide chain release factor 3 [Xan | taxID used:135614 OG00053|WP\_012915850.1 | carbamoyl-phosphate synthase large | taxID used:135614 OG00540|WP\_012917436.1 | APC family permease [Xanthomonas al | taxID used:135614 OG00541|WP\_045757224.1 | MBL fold metallo-hydrolase [Xanthom | taxID used:135614 OG00542|WP\_012917215.1 | GGDEF domain-containing protein [Xa | taxID used:135614 OG00543|WP\_012917516.1 | SulP family inorganic anion transpo | taxID used:135614 OG00544|WP\_012914809.1 | DUF1800 domain-containing protein [ | taxID used:135614 OG00546|WP\_045757330.1 | serine hydrolase [Xanthomonas albil | taxID used:135614 OG00547|WP\_012917459.1 | oxidoreductase [Xanthomonas albilin | taxID used:135614 OG00548|WP\_012915626.1 | YdiU family protein [Xanthomonas al | taxID used:135614 OG00549|WP\_012917407.1 | pentalenene synthase [Xanthomonas a | taxID used:135614 OG00054|WP\_080931594.1 | GGDEF domain-containing protein [Xa | taxID used:135614 OG00551|WP\_045756761.1 | NAD(P)/FAD-dependent oxidoreductase | taxID used:135614 OG00552|WP\_012915448.1 | murein biosynthesis integral membra | taxID used:135614 OG00555|WP\_012916491.1 | MFS transporter [Xanthomonas albili | taxID used:135614 OG00559|WP\_012915010.1 | alkyl hydroperoxide reductase subun | taxID used:135614 OG00055|WP\_045756695.1 | bifunctional proline dehydrogenase/ | taxID used:135614 OG00560|WP\_012916081.1 | glutamine-hydrolyzing GMP synthase | taxID used:135614 OG00561|WP\_012914866.1 | bifunctional phosphoribosylaminoimi | taxID used:135614 OG00562|WP\_045756696.1 | thioredoxin family protein [Xanthom | taxID used:135614 OG00563|WP\_012915238.1 | hypothetical protein [Xanthomonas a | taxID used:135614 OG00564|WP\_045757123.1 | DNA recombination protein RmuC [Xan | taxID used:135614 OG00565|WP\_012916447.1 | ketoglutarate semialdehyde dehydrog | taxID used:135614 OG00566|WP\_012915892.1 | flagellar biosynthesis regulator Fl | taxID used:135614 OG00567|WP\_012916444.1 | APC family permease [Xanthomonas al | taxID used:135614 OG00056|WP\_012917204.1 | acriflavine resistance protein B [X | taxID used:135614 OG00570|WP\_045756777.1 | N-acetylmuramoyl-L-alanine amidase | taxID used:135614 OG00571|WP\_012915283.1 | 2-isopropylmalate synthase [Xanthom | taxID used:135614 OG00572|WP\_045757918.1 | peptidase S10 [Xanthomonas albiline | taxID used:135614 OG00573|WP\_012917304.1 | hypothetical protein [Xanthomonas a | taxID used:135614 OG00574|WP\_012917622.1 | tryptophan 7-halogenase [Xanthomona | taxID used:135614 OG00576|WP\_012915724.1 | sodium transporter [Xanthomonas alb | taxID used:135614 OG00577|WP\_012914830.1 | monovalent cation/H+ antiporter sub | taxID used:135614 OG00579|WP\_012914917.1 | ATP-dependent protease [Xanthomonas | taxID used:135614 OG00057|WP\_012917619.1 | TonB-dependent receptor [Xanthomona | taxID used:135614 OG00580|WP\_012914622.1 | mannitol dehydrogenase family prote | taxID used:135614 OG00581|WP\_012917461.1 | catalase [Xanthomonas albilineans] | taxID used:135614 OG00582|WP\_012915820.1 | rRNA pseudouridine synthase [Xantho | taxID used:135614 OG00583|WP\_012914611.1 | tryptophan 7-halogenase [Xanthomona | taxID used:135614 OG00584|WP\_012917378.1 | F0F1 ATP synthase subunit alpha [Xa | taxID used:135614 OG00587|WP\_012915647.1 | fumarate hydratase [Xanthomonas alb | taxID used:135614 OG00589|WP\_012915735.1 | aldehyde dehydrogenase family prote | taxID used:135614 OG00590|WP\_012915855.1 | lysine--tRNA ligase [Xanthomonas al | taxID used:135614 OG00591|WP\_012917019.1 | DegQ family serine endoprotease [Xa | taxID used:135614 OG00592|WP\_045757055.1 | alpha/beta hydrolase [Xanthomonas a | taxID used:135614 OG00596|WP\_012915112.1 | exopolyphosphatase [Xanthomonas alb | taxID used:135614 OG00059|WP\_012916259.1 | multidrug efflux RND transporter pe | taxID used:135614 OG00600|WP\_012916490.1 | acetylhydrolase [Xanthomonas albili | taxID used:135614 OG00602|WP\_012914992.1 | glycerol-3-phosphate dehydrogenase | taxID used:135614 OG00603|WP\_012915254.1 | histidine kinase [Xanthomonas albil | taxID used:135614 OG00604|WP\_012917211.1 | MFS transporter [Xanthomonas albili | taxID used:135614 OG00605|WP\_012915359.1 | hybrid sensor histidine kinase/resp | taxID used:135614 OG00606|WP\_012915712.1 | xylulokinase [Xanthomonas albilinea | taxID used:135614 OG00609|WP\_012915722.1 | glucose-6-phosphate isomerase [Xant | taxID used:135614 OG00060|WP\_045756811.1 | coagulation factor 5/8 type domain- | taxID used:135614 OG00611|WP\_045756757.1 | sugar ABC transporter ATP-binding p | taxID used:135614 OG00612|WP\_012915536.1 | NADH-quinone oxidoreductase subunit | taxID used:135614 OG00613|WP\_012915539.1 | transcription termination/antitermi | taxID used:135614 OG00615|WP\_012916420.1 | lysine 6-aminotransferase [Xanthomo | taxID used:135614 OG00616|WP\_012917609.1 | NADP-dependent isocitrate dehydroge | taxID used:135614 OG00617|WP\_080929710.1 | FAD-dependent monooxygenase [Xantho | taxID used:135614 OG00618|WP\_012916733.1 | hypothetical protein [Xanthomonas a | taxID used:135614 OG00619|WP\_012916704.1 | glycoside hydrolase family 125 prot | taxID used:135614 OG00061|WP\_012916651.1 | AcrB/AcrD/AcrF family protein [Xant | taxID used:135614 OG00620|WP\_012917143.1 | leucyl aminopeptidase [Xanthomonas | taxID used:135614 OG00621|WP\_012915387.1 | two-component sensor histidine kina | taxID used:135614 OG00622|WP\_012916022.1 | MFS transporter [Xanthomonas albili | taxID used:135614 OG00623|WP\_012916934.1 | ribonuclease G [Xanthomonas albilin | taxID used:135614 OG00624|WP\_012917588.1 | glutamate synthase subunit beta [Xa | taxID used:135614 OG00625|WP\_012917287.1 | UDP-N-acetylmuramoylalanyl-D-glutam | taxID used:135614 OG00626|WP\_012915920.1 | sigma-54-dependent Fis family trans | taxID used:135614 OG00628|WP\_012916795.1 | biotin transporter BioY [Xanthomona | taxID used:135614 OG00629|WP\_012915107.1 | amidophosphoribosyltransferase [Xan | taxID used:135614 OG00062|WP\_080931546.1 | GGDEF domain-containing protein [Xa | taxID used:135614 OG00631|WP\_012914940.1 | membrane protein [Xanthomonas albil | taxID used:135614 OG00632|WP\_012915835.1 | amino acid permease [Xanthomonas al | taxID used:135614 OG00633|WP\_012914604.1 | MFS transporter [Xanthomonas albili | taxID used:135614 OG00634|WP\_012915537.1 | NADH-quinone oxidoreductase subunit | taxID used:135614 OG00635|WP\_012917288.1 | UDP-N-acetylmuramoyl-L-alanyl-D-glu | taxID used:135614 OG00636|WP\_012914840.1 | anthranilate synthase component I [ | taxID used:135614 OG00637|WP\_012915483.1 | MATE family efflux transporter [Xan | taxID used:135614 OG00638|WP\_012915554.1 | Fe-S cluster assembly protein SufB | taxID used:135614 OG00639|WP\_045756786.1 | deoxyribodipyrimidine photo-lyase [ | taxID used:135614 OG00063|WP\_012914902.1 | multidrug efflux RND transporter pe | taxID used:135614 OG00640|WP\_012917600.1 | DUF3375 domain-containing protein [ | taxID used:135614 OG00642|WP\_012916061.1 | outer membrane channel protein [Xan | taxID used:135614 OG00643|WP\_012915659.1 | wax ester/triacylglycerol synthase | taxID used:135614 OG00644|WP\_012917260.1 | adenosylhomocysteinase [Xanthomonas | taxID used:135614 OG00646|WP\_012915610.1 | bifunctional ADP-dependent NAD(P)H- | taxID used:135614 OG00648|WP\_012916278.1 | aspartate aminotransferase family p | taxID used:135614 OG00649|WP\_012915220.1 | amino acid ABC transporter permease | taxID used:135614 OG00064|WP\_045756916.1 | hypothetical protein [Xanthomonas a | taxID used:135614 OG00650|WP\_045756887.1 | exodeoxyribonuclease I [Xanthomonas | taxID used:135614 OG00651|WP\_080931595.1 | hypothetical protein [Xanthomonas a | taxID used:135614 OG00654|WP\_012916782.1 | pyruvate kinase [Xanthomonas albili | taxID used:135614 OG00655|WP\_012914946.1 | S41 family peptidase [Xanthomonas a | taxID used:135614 OG00656|WP\_012916623.1 | RNA polymerase sigma-54 factor [Xan | taxID used:135614 OG00659|WP\_012916082.1 | IMP dehydrogenase [Xanthomonas albi | taxID used:135614 OG00065|WP\_012917205.1 | AcrB/AcrD/AcrF family protein [Xant | taxID used:135614 OG00660|WP\_012915834.1 | amino acid permease [Xanthomonas al | taxID used:135614 OG00662|WP\_045757302.1 | glucose-6-phosphate dehydrogenase [ | taxID used:135614 OG00663|WP\_080929729.1 | chitinase [Xanthomonas albilineans] | taxID used:135614 OG00665|WP\_012916719.1 | chemotaxis-related protein [Xanthom | taxID used:135614 OG00666|WP\_012916990.1 | endoproteinase ArgC [Xanthomonas al | taxID used:135614 OG00667|WP\_012915281.1 | 3-isopropylmalate dehydratase large | taxID used:135614 OG00668|WP\_012914600.1 | glucuronate isomerase [Xanthomonas | taxID used:135614 OG00669|WP\_012916978.1 | virulence factor family protein [Xa | taxID used:135614 OG00066|WP\_012915629.1 | multidrug efflux RND transporter pe | taxID used:135614 OG00670|WP\_012916932.1 | metalloprotease TldD [Xanthomonas a | taxID used:135614 OG00671|WP\_045757349.1 | transporter [Xanthomonas albilinean | taxID used:135614 OG00672|WP\_012916656.1 | MFS transporter [Xanthomonas albili | taxID used:135614 OG00673|WP\_012916168.1 | dihydrolipoyl dehydrogenase [Xantho | taxID used:135614 OG00674|WP\_012915349.1 | nicotinate phosphoribosyltransferas | taxID used:135614 OG00676|WP\_012917563.1 | type I glutamate--ammonia ligase [X | taxID used:135614 OG00677|WP\_012914686.1 | cytochrome ubiquinol oxidase subuni | taxID used:135614 OG00678|WP\_045756644.1 | pyridine nucleotide-disulfide oxido | taxID used:135614 OG00679|WP\_012915024.1 | NAD(P)(+) transhydrogenase (Re/Si-s | taxID used:135614 OG00067|WP\_012914610.1 | membrane protein [Xanthomonas albil | taxID used:135614 OG00680|WP\_012917623.1 | amino acid permease [Xanthomonas al | taxID used:135614 OG00682|WP\_012917073.1 | sensor histidine kinase [Xanthomona | taxID used:135614 OG00683|WP\_080928195.1 | polynucleotide adenylyltransferase | taxID used:135614 OG00684|WP\_012915275.1 | membrane protein [Xanthomonas albil | taxID used:135614 OG00685|WP\_012915341.1 | septal ring lytic transglycosylase | taxID used:135614 OG00686|WP\_012917283.1 | UDP-N-acetylmuramate--L-alanine lig | taxID used:135614 OG00687|WP\_012916302.1 | hypothetical protein [Xanthomonas a | taxID used:135614 OG00688|WP\_012914660.1 | coniferyl aldehyde dehydrogenase [X | taxID used:135614 OG00068|WP\_012916947.1 | TonB-dependent receptor [Xanthomona | taxID used:135614 OG00690|WP\_012916991.1 | PepSY domain-containing protein [Xa | taxID used:135614 OG00691|WP\_012916234.1 | asparagine--tRNA ligase [Xanthomona | taxID used:135614 OG00692|WP\_012915775.1 | tRNA (N6-isopentenyl adenosine(37)- | taxID used:135614 OG00693|WP\_012915713.1 | MFS transporter [Xanthomonas albili | taxID used:135614 OG00694|WP\_012916546.1 | glutamate--tRNA ligase [Xanthomonas | taxID used:135614 OG00695|WP\_012914674.1 | cardiolipin synthase [Xanthomonas a | taxID used:135614 OG00696|WP\_012915082.1 | 3-deoxy-7-phosphoheptulonate syntha | taxID used:135614 OG00697|WP\_045756643.1 | MFS transporter [Xanthomonas albili | taxID used:135614 OG00698|WP\_012914903.1 | multidrug transporter [Xanthomonas | taxID used:135614 OG00699|WP\_012915657.1 | replicative DNA helicase [Xanthomon | taxID used:135614 OG00069|WP\_012917628.1 | CusA/CzcA family heavy metal efflux | taxID used:135614 OG00006|WP\_012916720.1 | hybrid sensor histidine kinase/resp | taxID used:135614 OG00700|WP\_012914808.1 | two-component sensor histidine kina | taxID used:135614 OG00701|WP\_012917493.1 | DUF3526 domain-containing protein [ | taxID used:135614 OG00702|WP\_012917561.1 | ammonia channel protein [Xanthomona | taxID used:135614 OG00703|WP\_012915698.1 | PhoH family protein [Xanthomonas al | taxID used:135614 OG00704|WP\_012917163.1 | mannose-1-phosphate guanylyltransfe | taxID used:135614 OG00705|WP\_012914986.1 | PDZ domain-containing protein [Xant | taxID used:135614 OG00706|WP\_012916266.1 | hypothetical protein [Xanthomonas a | taxID used:135614 OG00707|WP\_045756906.1 | cysteine--tRNA ligase [Xanthomonas | taxID used:135614 OG00708|WP\_012916212.1 | formimidoylglutamate deiminase [Xan | taxID used:135614 OG00709|WP\_012916577.1 | glutamine synthetase [Xanthomonas a | taxID used:135614 OG00710|WP\_012917559.1 | nitrogen regulation protein NR(I) [ | taxID used:135614 OG00711|WP\_012915570.1 | NAD(P)(+) transhydrogenase (Re/Si-s | taxID used:135614 OG00715|WP\_012917376.1 | F0F1 ATP synthase subunit beta [Xan | taxID used:135614 OG00716|WP\_012917580.1 | MFS transporter [Xanthomonas albili | taxID used:135614 OG00718|WP\_012916708.1 | alpha-L-fucosidase [Xanthomonas alb | taxID used:135614 OG00719|WP\_012916162.1 | class II fumarate hydratase [Xantho | taxID used:135614 OG00071|WP\_012917145.1 | valine--tRNA ligase [Xanthomonas al | taxID used:135614 OG00721|WP\_012916694.1 | adenosylmethionine--8-amino-7-oxono | taxID used:135614 OG00722|WP\_045757166.1 | NlpC-P60 family protein [Xanthomona | taxID used:135614 OG00723|WP\_012916683.1 | serine hydrolase [Xanthomonas albil | taxID used:135614 OG00725|WP\_045756807.1 | LOG family protein [Xanthomonas alb | taxID used:135614 OG00727|WP\_012915922.1 | RNA polymerase sigma-54 factor [Xan | taxID used:135614 OG00728|WP\_012917520.1 | type I-F CRISPR-associated protein | taxID used:135614 OG00729|WP\_012916662.1 | L-serine ammonia-lyase [Xanthomonas | taxID used:135614 OG00072|WP\_012916064.1 | AcrB/AcrD/AcrF family protein [Xant | taxID used:135614 OG00732|WP\_012917046.1 | DUF2252 domain-containing protein [ | taxID used:135614 OG00733|WP\_012915763.1 | histidine--tRNA ligase [Xanthomonas | taxID used:135614 OG00734|WP\_012915838.1 | FAD-binding oxidoreductase [Xanthom | taxID used:135614 OG00735|WP\_012916088.1 | ribosome biogenesis GTPase Der [Xan | taxID used:135614 OG00736|WP\_012917594.1 | lytic murein transglycosylase [Xant | taxID used:135614 OG00739|WP\_012916542.1 | chloride channel protein [Xanthomon | taxID used:135614 OG00741|WP\_012915902.1 | flagellar hook-length control prote | taxID used:135614 OG00742|WP\_045757203.1 | glutamate--cysteine ligase [Xanthom | taxID used:135614 OG00743|WP\_012917445.1 | D-serine/D-alanine/glycine transpor | taxID used:135614 OG00744|WP\_012914871.1 | MULTISPECIES: acetyl-CoA carboxylas | taxID used:135614 OG00746|WP\_012917179.1 | ABC transporter ATP-binding protein | taxID used:135614 OG00748|WP\_012915462.1 | DNA repair protein RadA [Xanthomona | taxID used:135614 OG00749|WP\_012917602.1 | MFS transporter [Xanthomonas albili | taxID used:135614 OG00074|WP\_012916727.1 | TonB-dependent receptor [Xanthomona | taxID used:135614 OG00750|WP\_012914980.1 | cytochrome c oxidase subunit II [Xa | taxID used:135614 OG00752|WP\_012915225.1 | alpha,alpha-trehalose-phosphate syn | taxID used:135614 OG00754|WP\_012916581.1 | diguanylate cyclase response regula | taxID used:135614 OG00755|WP\_012914937.1 | MFS transporter [Xanthomonas albili | taxID used:135614 OG00756|WP\_012916457.1 | aspartate aminotransferase family p | taxID used:135614 OG00757|WP\_012915529.1 | NADH oxidoreductase (quinone) subun | taxID used:135614 OG00758|WP\_012915461.1 | transferase [Xanthomonas albilinean | taxID used:135614 OG00759|WP\_012917371.1 | ATP-binding protein [Xanthomonas al | taxID used:135614 OG00761|WP\_012915297.1 | UDP-N-acetylmuramate:L-alanyl-gamma | taxID used:135614 OG00762|WP\_012915196.1 | sigma-54-dependent Fis family trans | taxID used:135614 OG00763|WP\_012915076.1 | preprotein translocase subunit SecY | taxID used:135614 OG00764|WP\_012917047.1 | ATP-dependent protease ATPase subun | taxID used:135614 OG00765|WP\_012917190.1 | RNA helicase [Xanthomonas albilinea | taxID used:135614 OG00767|WP\_012915964.1 | replication-associated recombinatio | taxID used:135614 OG00768|WP\_012915476.1 | signal recognition particle protein | taxID used:135614 OG00769|WP\_012915609.1 | exodeoxyribonuclease VII large subu | taxID used:135614 OG00771|WP\_012916163.1 | adenylosuccinate lyase [Xanthomonas | taxID used:135614 OG00772|WP\_012917232.1 | molecular chaperone SurA [Xanthomon | taxID used:135614 OG00773|WP\_012917183.1 | CBS domain-containing protein [Xant | taxID used:135614 OG00774|WP\_012917297.1 | dimethylmenaquinone methyltransfera | taxID used:135614 OG00775|WP\_012915562.1 | hypothetical protein [Xanthomonas a | taxID used:135614 OG00776|WP\_012916982.1 | heat-shock protein Hsp70 [Xanthomon | taxID used:135614 OG00777|WP\_012915663.1 | hypothetical protein [Xanthomonas a | taxID used:135614 OG00778|WP\_012916517.1 | RIP metalloprotease RseP [Xanthomon | taxID used:135614 OG00779|WP\_012915904.1 | FliI/YscN family ATPase [Xanthomona | taxID used:135614 OG00077|WP\_012915443.1 | excinuclease ABC subunit UvrA [Xant | taxID used:135614 OG00780|WP\_012916930.1 | metalloprotease PmbA [Xanthomonas a | taxID used:135614 OG00781|WP\_012916153.1 | UDP-glucose/GDP-mannose dehydrogena | taxID used:135614 OG00782|WP\_012915630.1 | ATP-dependent RNA helicase DbpA [Xa | taxID used:135614 OG00785|WP\_080931586.1 | serine protease [Xanthomonas albili | taxID used:135614 OG00788|WP\_012917162.1 | phosphomannomutase/phosphoglucomuta | taxID used:135614 OG00789|WP\_012916449.1 | NAD(P)/FAD-dependent oxidoreductase | taxID used:135614 OG00078|WP\_012915709.1 | TonB-dependent receptor [Xanthomona | taxID used:135614 OG00790|WP\_012917372.1 | UDP-N-acetylglucosamine diphosphory | taxID used:135614 OG00793|WP\_012915318.1 | Xaa-Pro dipeptidase [Xanthomonas al | taxID used:135614 OG00794|WP\_012915272.1 | O-antigen ligase family protein [Xa | taxID used:135614 OG00796|WP\_012914923.1 | glycosyltransferase [Xanthomonas al | taxID used:135614 OG00797|WP\_045756871.1 | outer membrane protein assembly fac | taxID used:135614 OG00798|WP\_012914566.1 | chromosomal replication initiator p | taxID used:135614 OG00799|WP\_012915274.1 | 3-deoxy-D-manno-octulosonic acid tr | taxID used:135614 OG00079|WP\_080929779.1 | DUF5110 domain-containing protein [ | taxID used:135614 OG00800|WP\_080931552.1 | flagellar protein [Xanthomonas albi | taxID used:135614 OG00801|WP\_012915826.1 | ABC transporter substrate-binding p | taxID used:135614 OG00802|WP\_012915527.1 | NADH-quinone oxidoreductase subunit | taxID used:135614 OG00803|WP\_012914979.1 | DUF1338 domain-containing protein [ | taxID used:135614 OG00804|WP\_012917173.1 | FAD-dependent oxidoreductase [Xanth | taxID used:135614 OG00805|WP\_012917341.1 | MATE family efflux transporter [Xan | taxID used:135614 OG00806|WP\_012916633.1 | amino acid permease [Xanthomonas al | taxID used:135614 OG00807|WP\_012916407.1 | N-ethylammeline chlorohydrolase [Xa | taxID used:135614 OG00808|WP\_012915114.1 | phosphate regulon sensor histidine | taxID used:135614 OG00809|WP\_012917398.1 | sorbosone dehydrogenase family prot | taxID used:135614 OG00080|WP\_012916678.1 | TonB-dependent receptor [Xanthomona | taxID used:135614 OG00810|WP\_012915585.1 | PLP-dependent aminotransferase fami | taxID used:135614 OG00811|WP\_012916400.1 | dihydroorotase [Xanthomonas albilin | taxID used:135614 OG00812|WP\_012916149.1 | divalent metal cation transporter [ | taxID used:135614 OG00813|WP\_012915190.1 | sensor histidine kinase [Xanthomona | taxID used:135614 OG00815|WP\_012916580.1 | FAD-dependent oxidoreductase [Xanth | taxID used:135614 OG00817|WP\_012916925.1 | dicarboxylate/amino acid:cation sym | taxID used:135614 OG00819|WP\_012914688.1 | AMP-ligase [Xanthomonas albilineans | taxID used:135614 OG00081|WP\_012914603.1 | TonB-dependent receptor [Xanthomona | taxID used:135614 OG00820|WP\_012917615.1 | amidohydrolase [Xanthomonas albilin | taxID used:135614 OG00821|WP\_012915317.1 | peptidase M24 family protein [Xanth | taxID used:135614 OG00822|WP\_045757376.1 | tRNA lysidine(34) synthetase TilS [ | taxID used:135614 OG00823|WP\_045757329.1 | polyhydroxyalkanoate depolymerase [ | taxID used:135614 OG00824|WP\_045757060.1 | DUF3526 domain-containing protein [ | taxID used:135614 OG00825|WP\_012916184.1 | HlyC/CorC family transporter [Xanth | taxID used:135614 OG00826|WP\_045757387.1 | 23S rRNA (uracil(1939)-C(5))-methyl | taxID used:135614 OG00828|WP\_012915266.1 | dicarboxylate/amino acid:cation sym | taxID used:135614 OG00082|WP\_012916170.1 | bifunctional diguanylate cyclase/ph | taxID used:135614 OG00831|WP\_012916630.1 | magnesium transporter [Xanthomonas | taxID used:135614 OG00832|WP\_012916972.1 | class I SAM-dependent methyltransfe | taxID used:135614 OG00833|WP\_045757137.1 | GTP-binding protein [Xanthomonas al | taxID used:135614 OG00835|WP\_012917498.1 | 3-carboxy-cis,cis-muconate cycloiso | taxID used:135614 OG00836|WP\_012915516.1 | phosphoglucosamine mutase [Xanthomo | taxID used:135614 OG00838|WP\_012915218.1 | ergothioneine biosynthesis protein | taxID used:135614 OG00839|WP\_012917207.1 | homogentisate 1,2-dioxygenase [Xant | taxID used:135614 OG00083|WP\_012915406.1 | glycine dehydrogenase (aminomethyl- | taxID used:135614 OG00841|WP\_012914999.1 | L-fuconate dehydratase [Xanthomonas | taxID used:135614 OG00842|WP\_012917072.1 | sigma-54-dependent Fis family trans | taxID used:135614 OG00843|WP\_012916897.1 | Tol-Pal system beta propeller repea | taxID used:135614 OG00844|WP\_012917121.1 | AGE family epimerase/isomerase [Xan | taxID used:135614 OG00845|WP\_012915260.1 | MFS transporter [Xanthomonas albili | taxID used:135614 OG00846|WP\_012917120.1 | glucose/galactose MFS transporter [ | taxID used:135614 OG00848|WP\_012917370.1 | sigma-54-dependent Fis family trans | taxID used:135614 OG00084|WP\_012914879.1 | membrane protein [Xanthomonas albil | taxID used:135614 OG00850|WP\_012917642.1 | tRNA uridine-5-carboxymethylaminome | taxID used:135614 OG00851|WP\_012916380.1 | acetylglutamate kinase [Xanthomonas | taxID used:135614 OG00852|WP\_080929789.1 | sensor histidine kinase [Xanthomona | taxID used:135614 OG00854|WP\_012915949.1 | nitrate ABC transporter ATP-binding | taxID used:135614 OG00855|WP\_012915687.1 | GTPase HflX [Xanthomonas albilinean | taxID used:135614 OG00857|WP\_012917285.1 | putative lipid II flippase FtsW [Xa | taxID used:135614 OG00859|WP\_012917064.1 | PAS domain-containing sensor histid | taxID used:135614 OG00085|WP\_012915377.1 | TonB-dependent receptor [Xanthomona | taxID used:135614 OG00860|WP\_045757134.1 | NAD(P)/FAD-dependent oxidoreductase | taxID used:135614 OG00861|WP\_012917513.1 | MFS transporter, partial [Xanthomon | taxID used:135614 OG00862|WP\_012914900.1 | MFS transporter [Xanthomonas albili | taxID used:135614 OG00864|WP\_045757124.1 | MFS transporter [Xanthomonas albili | taxID used:135614 OG00865|WP\_045757036.1 | 16S rRNA (cytosine(967)-C(5))-methy | taxID used:135614 OG00867|WP\_012916834.1 | citrate synthase [Xanthomonas albil | taxID used:135614 OG00869|WP\_049887205.1 | hypothetical protein [Xanthomonas a | taxID used:135614 OG00086|WP\_012915174.1 | TonB-dependent receptor [Xanthomona | taxID used:135614 OG00870|WP\_045757286.1 | serine--tRNA ligase [Xanthomonas al | taxID used:135614 OG00871|WP\_012917405.1 | D-amino acid dehydrogenase small su | taxID used:135614 OG00873|WP\_045757183.1 | ABC transporter permease [Xanthomon | taxID used:135614 OG00874|WP\_012915121.1 | trigger factor [Xanthomonas albilin | taxID used:135614 OG00876|WP\_045757171.1 | hypothetical protein [Xanthomonas a | taxID used:135614 OG00877|WP\_012915305.1 | glutamate-1-semialdehyde-2,1-aminom | taxID used:135614 OG00878|WP\_012915778.1 | cytochrome b [Xanthomonas albilinea | taxID used:135614 OG00879|WP\_012914818.1 | tryptophan--tRNA ligase [Xanthomona | taxID used:135614 OG00087|WP\_012916432.1 | TonB-dependent receptor [Xanthomona | taxID used:135614 OG00880|WP\_012915584.1 | enoyl-CoA hydratase/isomerase famil | taxID used:135614 OG00881|WP\_012915185.1 | adenylosuccinate synthase [Xanthomo | taxID used:135614 OG00882|WP\_012915001.1 | L-fucose:H+ symporter permease [Xan | taxID used:135614 OG00883|WP\_045756879.1 | 3-phosphoshikimate 1-carboxyvinyltr | taxID used:135614 OG00884|WP\_012916250.1 | kynureninase [Xanthomonas albilinea | taxID used:135614 OG00885|WP\_012915759.1 | threonine synthase [Xanthomonas alb | taxID used:135614 OG00886|WP\_012917251.1 | Na+ dependent nucleoside transporte | taxID used:135614 OG00887|WP\_012915575.1 | diaminobutyrate--2-oxoglutarate tra | taxID used:135614 OG00888|WP\_012916193.1 | nucleotide sugar dehydrogenase [Xan | taxID used:135614 OG00889|WP\_045757405.1 | sensor histidine kinase [Xanthomona | taxID used:135614 OG00891|WP\_012916377.1 | argininosuccinate lyase [Xanthomona | taxID used:135614 OG00892|WP\_012915193.1 | type II secretion system F family p | taxID used:135614 OG00893|WP\_012915366.1 | glucose/galactose MFS transporter [ | taxID used:135614 OG00894|WP\_012916410.1 | lytic murein transglycosylase [Xant | taxID used:135614 OG00896|WP\_012914625.1 | D-galactonate dehydratase family pr | taxID used:135614 OG00897|WP\_012915123.1 | MULTISPECIES: ATP-dependent Clp pro | taxID used:135614 OG00898|WP\_012916970.1 | FAD-dependent oxidoreductase [Xanth | taxID used:135614 OG00899|WP\_045756751.1 | sensor histidine kinase [Xanthomona | taxID used:135614 OG00900|WP\_045757035.1 | O-antigen ligase family protein [Xa | taxID used:135614 OG00902|WP\_012915399.1 | pyridoxal phosphate-dependent amino | taxID used:135614 OG00903|WP\_012916207.1 | lectin subunit beta [Xanthomonas al | taxID used:135614 OG00905|WP\_012914865.1 | phosphoribosylamine--glycine ligase | taxID used:135614 OG00906|WP\_012915031.1 | hypothetical protein [Xanthomonas a | taxID used:135614 OG00908|WP\_012917605.1 | lipase [Xanthomonas albilineans] [G | taxID used:135614 OG00910|WP\_012915767.1 | histidinol dehydrogenase [Xanthomon | taxID used:135614 OG00911|WP\_012915670.1 | phosphopyruvate hydratase [Xanthomo | taxID used:135614 OG00913|WP\_045757069.1 | acetyl-CoA C-acyltransferase [Xanth | taxID used:135614 OG00915|WP\_012915038.1 | glutamyl-tRNA reductase [Xanthomona | taxID used:135614 OG00917|WP\_012915813.1 | histidine kinase [Xanthomonas albil | taxID used:135614 OG00091|WP\_012916439.1 | TonB-denpendent receptor [Xanthomon | taxID used:135614 OG00920|WP\_012915728.1 | flavodoxin-dependent (E)-4-hydroxy- | taxID used:135614 OG00921|WP\_012916273.1 | nucleotide sugar aminotransferase [ | taxID used:135614 OG00922|WP\_045757165.1 | amino acid permease [Xanthomonas al | taxID used:135614 OG00924|WP\_012917555.1 | porphyrin biosynthesis protein [Xan | taxID used:135614 OG00925|WP\_012917313.1 | serine hydroxymethyltransferase [Xa | taxID used:135614 OG00928|WP\_012915472.1 | potassium transporter [Xanthomonas | taxID used:135614 OG00929|WP\_012916190.1 | aminoacetone oxidase family FAD-bin | taxID used:135614 OG00930|WP\_012915405.1 | Bcr/CflA family drug resistance eff | taxID used:135614 OG00931|WP\_012915104.1 | bifunctional tetrahydrofolate synth | taxID used:135614 OG00932|WP\_045756919.1 | DUF3138 domain-containing protein [ | taxID used:135614 OG00933|WP\_012915295.1 | 6-phosphofructokinase [Xanthomonas | taxID used:135614 OG00934|WP\_012916616.1 | UDP-N-acetylglucosamine 1-carboxyvi | taxID used:135614 OG00936|WP\_012915370.1 | multifunctional CCA addition/repair | taxID used:135614 OG00937|WP\_012915550.1 | cysteine desulfurase [Xanthomonas a | taxID used:135614 OG00938|WP\_012915250.1 | carboxylate--amine ligase [Xanthomo | taxID used:135614 OG00093|WP\_012916166.1 | 2-oxoglutarate dehydrogenase E1 com | taxID used:135614 OG00940|WP\_045756739.1 | YggW family oxidoreductase [Xanthom | taxID used:135614 OG00941|WP\_045756877.1 | cupin domain-containing protein [Xa | taxID used:135614 OG00942|WP\_012917152.1 | membrane protein [Xanthomonas albil | taxID used:135614 OG00943|WP\_012916632.1 | arsenic transporter [Xanthomonas al | taxID used:135614 OG00944|WP\_012916560.1 | signal recognition particle-docking | taxID used:135614 OG00945|WP\_012915551.1 | Fe-S cluster assembly protein SufD | taxID used:135614 OG00946|WP\_012916911.1 | cation:proton antiporter [Xanthomon | taxID used:135614 OG00947|WP\_012916388.1 | MFS transporter [Xanthomonas albili | taxID used:135614 OG00948|WP\_012917284.1 | undecaprenyldiphospho-muramoylpenta | taxID used:135614 OG00094|WP\_045757251.1 | bifunctional diguanylate cyclase/ph | taxID used:135614 OG00950|WP\_012916311.1 | lipoprotein-releasing system transm | taxID used:135614 OG00951|WP\_080931550.1 | endonuclease/exonuclease/phosphatas | taxID used:135614 OG00952|WP\_012917029.1 | hemolysin D, partial [Xanthomonas a | taxID used:135614 OG00953|WP\_012915157.1 | beta-ketoacyl-[acyl-carrier-protein | taxID used:135614 OG00954|WP\_012916459.1 | DUF445 domain-containing protein [X | taxID used:135614 OG00955|WP\_012917280.1 | MULTISPECIES: cell division protein | taxID used:135614 OG00956|WP\_012915401.1 | flavohemoglobin expression-modulati | taxID used:135614 OG00095|WP\_012916998.1 | autotransporter domain-containing p | taxID used:135614 OG00960|WP\_012915233.1 | efflux RND transporter periplasmic | taxID used:135614 OG00964|WP\_012917203.1 | efflux RND transporter periplasmic | taxID used:135614 OG00965|WP\_045756690.1 | bifunctional phosphopantothenoylcys | taxID used:135614 OG00966|WP\_012916374.1 | glutamate-5-semialdehyde dehydrogen | taxID used:135614 OG00967|WP\_012914941.1 | tyrosine--tRNA ligase [Xanthomonas | taxID used:135614 OG00968|WP\_045756813.1 | phosphoglycerate dehydrogenase [Xan | taxID used:135614 OG00969|WP\_045757375.1 | DUF418 domain-containing protein [X | taxID used:135614 OG00096|WP\_012914832.1 | monovalent cation/H+ antiporter sub | taxID used:135614 OG00970|WP\_012916277.1 | beta-ketoacyl-[acyl-carrier-protein | taxID used:135614 OG00971|WP\_012916258.1 | efflux RND transporter periplasmic | taxID used:135614 OG00972|WP\_012915944.1 | sensor histidine kinase [Xanthomona | taxID used:135614 OG00974|WP\_012915246.1 | type III PLP-dependent enzyme [Xant | taxID used:135614 OG00975|WP\_045756745.1 | aspartate aminotransferase family p | taxID used:135614 OG00976|WP\_012916383.1 | argininosuccinate synthase [Xanthom | taxID used:135614 OG00977|WP\_012915936.1 | flagellar hook protein FlgE [Xantho | taxID used:135614 OG00978|WP\_045757409.1 | phosphoanhydride phosphohydrolase [ | taxID used:135614 OG00979|WP\_012916446.1 | aminopeptidase P family protein [Xa | taxID used:135614 OG00097|WP\_045756727.1 | TonB-dependent receptor [Xanthomona | taxID used:135614 OG00981|WP\_012916093.1 | 23S rRNA (adenine(2503)-C(2))-methy | taxID used:135614 OG00982|WP\_012915304.1 | aspartate aminotransferase family p | taxID used:135614 OG00983|WP\_012915219.1 | aminotransferase class V-fold PLP-d | taxID used:135614 OG00986|WP\_012917257.1 | methionine adenosyltransferase [Xan | taxID used:135614 OG00987|WP\_012915342.1 | D-alanyl-D-alanine carboxypeptidase | taxID used:135614 OG00988|WP\_045756713.1 | lytic transglycosylase [Xanthomonas | taxID used:135614 OG00989|WP\_012916109.1 | TraB family protein [Xanthomonas al | taxID used:135614 OG00098|WP\_045757213.1 | TonB-dependent receptor [Xanthomona | taxID used:135614 OG00990|WP\_012917279.1 | cell division protein FtsZ [Xanthom | taxID used:135614 OG00992|WP\_012917053.1 | pyridoxal phosphate-dependent amino | taxID used:135614 OG00994|WP\_012915953.1 | tRNA 2-thiouridine(34) synthase Mnm | taxID used:135614 OG00995|WP\_045756795.1 | sensor histidine kinase [Xanthomona | taxID used:135614 OG00996|WP\_012916086.1 | molybdopterin molybdenumtransferase | taxID used:135614 OG00997|WP\_012915513.1 | tryptophan synthase subunit beta [X | taxID used:135614 OG00998|WP\_012914586.1 | enoyl-[acyl-carrier-protein] reduct | taxID used:135614 OG00099|WP\_049887161.1 | bifunctional [glutamate--ammonia li | taxID used:135614
[truncated: 279,811 more chars]
