# Supplementary material for: Orthology-Based Estimate of the Contribution of Horizontal Gene Transfer from Distantly Related Bacteria to the Intraspecific Diversity and Differentiation of Xylella fastidiosa
Source: Pathogens. 2021 Jan 7;10(1):46. doi: 10.3390/pathogens10010046 (PMC7828034; doi:10.3390/pathogens10010046)
Supplement: Supplementary file 1 [file pathogens-10-00046-s001.zip › pathogens-1031631-supplementary-final/pathogens-1031631-Figure S4.html]

Javascript must be enabled to view this page.

members
magnitude
magnitudeUnassigned
count
unassigned
taxon
rank

Busco
Core
Pangenome

43710453049

superkingdom
43710453049
2

phylum
43710453011
1224

28216
2375
class

1311
order


OG02220|WP\_011098226.1 | hypothetical protein | taxID used:80840 OG02306|WP\_023906603.1 | cupin domain-containing protein | taxID used:80840 OG02484|WP\_060870378.1 | hypothetical protein | taxID used:80840 OG00976|WP\_004083632.1 | DNA cytosine methyltransferase | taxID used:80840 
80840
4

93681
7
genus


OG01154|WP\_126709124.1 | hypothetical protein | taxID used:1855305 OG01163|WP\_046418682.1 | hypothetical protein | taxID used:1855305 OG01276|WP\_010893417.1 | hypothetical protein | taxID used:1855305 OG01328|WP\_076613608.1 | hypothetical protein | taxID used:1855305 OG01486|WP\_076613272.1 | hypothetical protein, partial | taxID used:1855305 OG02690|WP\_038274292.1 | membrane protein | taxID used:1855305 OG00714|WP\_011097887.1 | hemagglutinin, partial | taxID used:1855305 
species
7
1855305 

10
75682
family

2
29580
genus

1075768 
species


OG02232|WP\_020850971.1 | ATP-dependent helicase | taxID used:1075768 
1


OG02761|WP\_088577924.1 | hypothetical protein | taxID used:1537274 
species
1
1537274 

963
7
genus


OG02311|WP\_004087820.1 | hypothetical protein | taxID used:213312 
species
1
213312 

4


OG00532|WP\_060871695.1 | hemagglutinin | taxID used:211589 OG00540|WP\_023908183.1 | hemagglutinin | taxID used:211589 OG00645|WP\_031337194.1 | hemagglutinin | taxID used:211589 OG00069|WP\_081089895.1 | hemagglutinin-related protein | taxID used:211589 
species
211589 

species


OG01798|WP\_023908172.1 | hypothetical protein | taxID used:964 
1
964 

346179 
species


OG02127|WP\_060870149.1 | hypothetical protein | taxID used:346179 
1

1
149698
genus


OG01304|WP\_004090261.1 | hypothetical protein | taxID used:1678028 
species
1
1678028 

1158
family


OG01543|WP\_081046826.1 | DUF3800 domain-containing protein | taxID used:119060 OG02461|WP\_042464090.1 | type II toxin-antitoxin system RelE/ParE family toxin | taxID used:119060 
119060
2

genus
1822464
11

28094 


OG02304|WP\_004086769.1 | cupin domain-containing protein | taxID used:28094 
species
1

species


OG02391|WP\_024748528.1 | PIN domain-containing protein | taxID used:36873 
1
36873 

4


OG02231|WP\_004085897.1 | hypothetical protein | taxID used:412963 OG02372|WP\_004090697.1 | type II toxin-antitoxin system MqsA family antitoxin | taxID used:412963 OG02397|WP\_004085029.1 | hypothetical protein | taxID used:412963 OG03663|WP\_081044460.1 | DUF1640 domain-containing protein | taxID used:412963 
species
412963 

312026 
1
species


OG02673|WP\_080507177.1 | hypothetical protein | taxID used:312026 

417203 


OG01957|WP\_060871998.1 | DUF1640 domain-containing protein | taxID used:417203 OG01984|WP\_031337817.1 | DUF1640 domain-containing protein | taxID used:417203 OG02878|WP\_038274391.1 | hypothetical protein | taxID used:417203 
species
3

1441714 
species


OG03134|WP\_080729512.1 | hypothetical protein | taxID used:1441714 
1

genus
1827195
6

species


OG02644|WP\_060871680.1 | hypothetical protein | taxID used:1777143 
1
1777143 

1777134 
2


OG02686|WP\_010893744.1 | hypothetical protein | taxID used:1777134 OG02718|WP\_021358615.1 | hypothetical protein | taxID used:1777134 
species

1
species


OG00464|WP\_004091515.1 | TrbI/VirB10 family protein | taxID used:326474 
326474 

1


OG01488|WP\_023906482.1 | membrane protein | taxID used:1777136 
species
1777136 

196367 
species


OG03096|WP\_020852902.1 | transcriptional regulator AbrB | taxID used:196367 
1

240411
1
genus

1559339 
species


OG02709|WP\_060870072.1 | XRE family transcriptional regulator | taxID used:1559339 
1

1
106589
genus


OG02646|WP\_023908247.1 | XRE family transcriptional regulator | taxID used:106589 
10

119219 
3
species


OG01693|WP\_118853414.1 | hypothetical protein | taxID used:119219 OG01820|WP\_080679734.1 | hypothetical protein | taxID used:119219 OG01911|WP\_038230623.1 | hypothetical protein | taxID used:119219 

1312922 
2


OG02176|WP\_081364533.1 | HNH endonuclease | taxID used:1312922 OG02312|WP\_031337162.1 | hypothetical protein | taxID used:1312922 
species


OG02937|WP\_004091355.1 | XRE family transcriptional regulator | taxID used:68895 OG03068|WP\_080513031.1 | hypothetical protein | taxID used:68895 
species
2
68895 

367825 


OG02403|WP\_060872197.1 | hypothetical protein | taxID used:367825 
species
1

876364 
1
species


OG02453|WP\_010894193.1 | LysR family transcriptional regulator | taxID used:876364 

32008
5
44
genus


OG01355|WP\_023906375.1 | DUF2815 domain-containing protein | taxID used:32008 OG00168|WP\_060872410.1 | DNA-directed DNA polymerase | taxID used:32008 OG01851|WP\_024748663.1 | DUF2815 domain-containing protein | taxID used:32008 OG03045|WP\_088572643.1 | hypothetical protein | taxID used:32008 OG00483|WP\_081046896.1 | DNA polymerase | taxID used:32008 

5


OG01263|WP\_081095316.1 | conjugal transfer protein TrbI | taxID used:1926878 OG02524|WP\_040123200.1 | conjugal transfer protein, partial | taxID used:1926878 OG02551|WP\_088577939.1 | conjugal transfer protein | taxID used:1926878 OG03179|WP\_010894503.1 | hypothetical protein | taxID used:1926878 OG00465|WP\_042464052.1 | conjugal transfer protein TrbI | taxID used:1926878 
species
1926878 

1761774 


OG02298|WP\_040123340.1 | DUF1640 domain-containing protein | taxID used:1761774 OG02346|WP\_020851668.1 | DUF1640 domain-containing protein, partial | taxID used:1761774 OG02531|WP\_010894740.1 | DUF1640 domain-containing protein | taxID used:1761774 OG02726|WP\_023907477.1 | DUF1640 domain-containing protein | taxID used:1761774 OG02856|WP\_046420589.1 | DUF1640 domain-containing protein | taxID used:1761774 OG02887|WP\_058570029.1 | hypothetical protein | taxID used:1761774 OG03026|WP\_020851619.1 | DUF1640 domain-containing protein | taxID used:1761774 
species
7

1836045 


OG02625|WP\_046419237.1 | hypothetical protein | taxID used:1836045 
species
1

species group
3
111527

28450 
species


OG01221|WP\_020852944.1 | hypothetical protein | taxID used:28450 
1

57975 
2


OG02965|WP\_014607494.1 | hypothetical protein | taxID used:57975 OG03302|WP\_004086582.1 | lipase chaperone | taxID used:57975 
species


OG02701|WP\_038230332.1 | type II toxin-antitoxin system PemK/MazF family toxin | taxID used:242163 
species
1
242163 


OG03564|WP\_020850948.1 | hypothetical protein | taxID used:28095 OG00372|WP\_060870359.1 | hypothetical protein | taxID used:28095 
species
2
28095 

species group
20
87882

4


OG02806|WP\_023907240.1 | colicin V synthesis protein | taxID used:292 OG03386|WP\_081089772.1 | hypothetical protein | taxID used:292 OG03445|WP\_076613211.1 | hypothetical protein | taxID used:292 OG03453|WP\_088569661.1 | hypothetical protein | taxID used:292 
species
292 


OG02448|WP\_060872042.1 | PIN domain-containing protein | taxID used:101571 OG03133|WP\_088577927.1 | plasmid stability protein stbC | taxID used:101571 
species
2
101571 

species


OG01516|WP\_080679735.1 | hypothetical protein | taxID used:87883 OG02703|WP\_024749127.1 | hypothetical protein | taxID used:87883 OG03012|WP\_046417513.1 | type II toxin-antitoxin system RelE/ParE family toxin | taxID used:87883 OG03561|WP\_012382581.1 | hypothetical protein | taxID used:87883 OG03569|WP\_020852716.1 | hypothetical protein | taxID used:87883 OG03634|WP\_042464043.1 | plasmid stabilization protein | taxID used:87883 
6
87883 

152480
species


OG03048|WP\_060870051.1 | CopG family transcriptional regulator | taxID used:339670 
1

60552 
1


OG03035|WP\_031337170.1 | hypothetical protein | taxID used:60552 
species


OG02040|WP\_046417897.1 | hypothetical protein | taxID used:1637875 OG02467|WP\_004087957.1 | restriction endonuclease subunit S | taxID used:1637875 
species
2
1637875 

488732 
3
species


OG00151|WP\_010895214.1 | hypothetical protein | taxID used:488732 OG03077|WP\_060871751.1 | hypothetical protein | taxID used:488732 OG03540|WP\_010895257.1 | conjugal transfer protein | taxID used:488732 


OG00373|WP\_088572612.1 | hypothetical protein | taxID used:216591 
species
1
95486

44013
4
genus

1743157 
species


OG02000|WP\_004572886.1 | glutamine amidotransferase | taxID used:1743157 OG02913|WP\_118853459.1 | hypothetical protein | taxID used:1743157 OG02953|WP\_004572878.1 | trp operon repressor | taxID used:1743157 OG00396|WP\_023907608.1 | anthranilate synthase component I family protein | taxID used:1743157 
4

2
1810868
genus

1553431 


OG01976|WP\_058565160.1 | hypothetical protein | taxID used:1553431 OG00358|WP\_004085027.1 | DNA-directed DNA polymerase | taxID used:1553431 
species
2

1
48736
genus


OG01121|WP\_023907138.1 | hypothetical protein | taxID used:48736 
165

105219 
species


OG02103|WP\_020851019.1 | M48 family peptidase | taxID used:105219 
1

1944648 
4
species


OG01512|WP\_126715048.1 | DUF1376 domain-containing protein, partial | taxID used:1944648 OG02743|WP\_023906549.1 | DUF1376 domain-containing protein | taxID used:1944648 OG03138|WP\_081044379.1 | hypothetical protein | taxID used:1944648 OG00789|WP\_063618861.1 | DUF1376 domain-containing protein | taxID used:1944648 


OG02658|WP\_080513037.1 | hypothetical protein | taxID used:795666 OG00832|WP\_027700397.1 | restriction endonuclease subunit S | taxID used:795666 
species
2
795666 

305 
157
species


OG02385|WP\_027700119.1 | DUF596 domain-containing protein | taxID used:305 
OG01104|WP\_004085602.1 | hypothetical protein | taxID used:305 OG01130|WP\_004090263.1 | hypothetical protein | taxID used:305 OG01131|WP\_004090262.1 | hypothetical protein | taxID used:305 OG01174|WP\_010895247.1 | DUF4942 domain-containing protein | taxID used:305 OG01180|WP\_004089585.1 | hypothetical protein | taxID used:305 OG01183|WP\_060871977.1 | hypothetical protein | taxID used:305 OG01184|WP\_023908036.1 | hypothetical protein | taxID used:305 OG01187|WP\_060871925.1 | hypothetical protein | taxID used:305 OG01188|WP\_088569695.1 | hypothetical protein | taxID used:305 OG01195|WP\_023908038.1 | DUF769 domain-containing protein | taxID used:305 OG01196|WP\_023908035.1 | hypothetical protein | taxID used:305 OG01198|WP\_011097888.1 | hypothetical protein | taxID used:305 OG01202|WP\_046418678.1 | DUF769 domain-containing protein | taxID used:305 OG01322|WP\_004086157.1 | hemagglutinin | taxID used:305 OG01705|WP\_063618863.1 | hemagglutinin | taxID used:305 OG01874|WP\_042836442.1 | hypothetical protein | taxID used:305 OG01924|WP\_080507221.1 | hypothetical protein | taxID used:305 OG00001|WP\_071869970.1 | filamentous hemagglutinin N-terminal domain-containing protein | taxID used:305 OG02190|WP\_118853430.1 | hemagglutinin | taxID used:305 OG02357|WP\_004083391.1 | DUF596 domain-containing protein | taxID used:305 OG02375|WP\_004088338.1 | DUF596 domain-containing protein | taxID used:305 OG02382|WP\_031337198.1 | DUF596 domain-containing protein | taxID used:305 OG02384|WP\_010894641.1 | DUF596 domain-containing protein | taxID used:305 OG02385|WP\_027700119.1 | DUF596 domain-containing protein | taxID used:305 OG02404|WP\_060872241.1 | DUF596 domain-containing protein | taxID used:305 OG02411|WP\_031336471.1 | DUF596 domain-containing protein | taxID used:305 OG02412|WP\_004087821.1 | DUF596 domain-containing protein | taxID used:305 OG02415|WP\_088578194.1 | DUF596 domain-containing protein | taxID used:305 OG02419|WP\_004090790.1 | DUF596 domain-containing protein | taxID used:305 OG02420|WP\_004090788.1 | DUF596 domain-containing protein | taxID used:305 OG02422|WP\_031337201.1 | DUF596 domain-containing protein | taxID used:305 OG02425|WP\_081044445.1 | hemagglutinin | taxID used:305 OG02427|WP\_004088339.1 | DUF596 domain-containing protein | taxID used:305 OG02430|WP\_126715036.1 | hemagglutinin | taxID used:305 OG02437|WP\_057682466.1 | DUF596 domain-containing protein | taxID used:305 OG02440|WP\_011098380.1 | DUF596 domain-containing protein | taxID used:305 OG02446|WP\_038230102.1 | DUF596 domain-containing protein | taxID used:305 OG02452|WP\_004089371.1 | DUF596 domain-containing protein | taxID used:305 OG02454|WP\_012337933.1 | DUF596 domain-containing protein | taxID used:305 OG02459|WP\_038228427.1 | DUF596 domain-containing protein | taxID used:305 OG02460|WP\_012382790.1 | DUF596 domain-containing protein | taxID used:305 OG02463|WP\_004086155.1 | DUF596 domain-containing protein | taxID used:305 OG02468|WP\_004090973.1 | hypothetical protein | taxID used:305 OG02470|WP\_023908182.1 | DUF596 domain-containing protein | taxID used:305 OG02513|WP\_011098383.1 | hemagglutinin | taxID used:305 OG02559|WP\_011098381.1 | hemagglutinin | taxID used:305 OG02562|WP\_004090753.1 | DUF596 domain-containing protein | taxID used:305 OG03327|WP\_058570058.1 | DUF596 domain-containing protein | taxID used:305 OG03347|WP\_081378026.1 | hemagglutinin | taxID used:305 OG03665|WP\_072866420.1 | DUF596 domain-containing protein | taxID used:305 OG00056|WP\_061278124.1 | hemagglutinin | taxID used:305 OG00801|WP\_012382788.1 | hypothetical protein | taxID used:305 OG00805|WP\_046420959.1 | hypothetical protein | taxID used:305 OG00850|WP\_060872191.1 | hypothetical protein | taxID used:305 OG00939|WP\_081392196.1 | hemagglutinin | taxID used:305 OG02450|WP\_038211469.1 | DUF596 domain-containing protein | taxID used:305 OG02455|WP\_004086848.1 | DUF596 domain-containing protein | taxID used:305 

genus
224135
9

132144 
species


OG02007|WP\_004085881.1 | hypothetical protein | taxID used:132144 
1

8
species


OG02050|WP\_076613243.1 | hypothetical protein | taxID used:1286373 OG02066|WP\_023907042.1 | hypothetical protein | taxID used:1286373 OG02248|WP\_060871775.1 | hypothetical protein | taxID used:1286373 OG02872|WP\_058570001.1 | hypothetical protein | taxID used:1286373 OG00575|WP\_071869949.1 | DUF2800 domain-containing protein | taxID used:1286373 OG00594|WP\_012382637.1 | DUF2800 domain-containing protein | taxID used:1286373 OG00795|WP\_040123273.1 | DUF2800 domain-containing protein | taxID used:1286373 OG00969|WP\_060872115.1 | DUF2800 domain-containing protein | taxID used:1286373 
1286373 

genus


OG02388|WP\_038230267.1 | hypothetical protein, partial | taxID used:93217 
4
1
93217

573737 
species


OG01333|WP\_058564969.1 | recombinase family protein | taxID used:573737 OG02107|WP\_004086742.1 | recombinase family protein | taxID used:573737 OG03637|WP\_080544275.1 | hypothetical protein | taxID used:573737 
3

genus
318147
1


OG01595|WP\_038211241.1 | hypothetical protein | taxID used:1768242 
species
1
1768242 

1
species


OG03165|WP\_023908086.1 | hypothetical protein | taxID used:1891238 
1891238 


OG02894|WP\_004085592.1 | putative addiction module antidote protein | taxID used:1469502 
species
1
1469502 

5
80864
family


OG01414|WP\_058565014.1 | hypothetical protein | taxID used:83494 OG02899|WP\_004090909.1 | type II toxin-antitoxin system YafQ family toxin | taxID used:80864 OG02939|WP\_060870083.1 | type II toxin-antitoxin system YafQ family toxin | taxID used:80864 OG03072|WP\_060870273.1 | type II toxin-antitoxin system antitoxin, RelB/DinJ family | taxID used:80864 OG00615|WP\_038231351.1 | hypothetical protein, partial | taxID used:80864 
79

364316
40
genus

35


OG01015|WP\_060871694.1 | conjugal transfer protein TrbL | taxID used:364317 OG01021|WP\_069107187.1 | conjugal transfer protein TrbL | taxID used:364317 OG00109|WP\_081046866.1 | CagE, TrbE, VirB component of type IV transporter system | taxID used:364317 OG01246|WP\_013087966.1 | hypothetical protein | taxID used:364317 OG01343|WP\_060870084.1 | conjugal transfer protein TrbL | taxID used:364317 OG01371|WP\_038211583.1 | conjugal transfer protein TrbG | taxID used:364317 OG01409|WP\_060870054.1 | conjugal transfer protein TraL | taxID used:364317 OG00148|WP\_020852264.1 | conjugal transfer protein TraG | taxID used:364317 OG01502|WP\_058564986.1 | VirB8 | taxID used:364317 OG01586|WP\_013087957.1 | glutamate-1-semialdehyde 2,1-aminomutase | taxID used:364317 OG01645|WP\_020852241.1 | plasmid mobilization relaxosome protein MobC | taxID used:364317 OG01764|WP\_060871683.1 | recombinase family protein | taxID used:364317 OG01832|WP\_004089198.1 | hypothetical protein | taxID used:364317 OG02105|WP\_081046867.1 | hypothetical protein | taxID used:364317 OG02252|WP\_014607318.1 | hypothetical protein | taxID used:364317 OG02434|WP\_013087959.1 | hypothetical protein | taxID used:364317 OG02510|WP\_010894485.1 | hypothetical protein | taxID used:364317 OG02573|WP\_020852242.1 | hypothetical protein | taxID used:364317 OG02684|WP\_010895215.1 | hypothetical protein | taxID used:364317 OG02694|WP\_031336788.1 | DUF1640 domain-containing protein | taxID used:364317 OG02730|WP\_013087960.1 | hypothetical protein | taxID used:364317 OG02785|WP\_082355556.1 | hypothetical protein | taxID used:364317 OG02800|WP\_081090393.1 | hypothetical protein | taxID used:364317 OG02822|WP\_080679722.1 | hypothetical protein | taxID used:364317 OG02974|WP\_080702548.1 | hypothetical protein | taxID used:364317 OG03127|WP\_014607322.1 | hypothetical protein | taxID used:364317 OG03154|WP\_014607311.1 | hypothetical protein | taxID used:364317 OG03437|WP\_060872367.1 | hypothetical protein | taxID used:364317 OG03554|WP\_027700471.1 | hypothetical protein | taxID used:364317 OG03617|WP\_081089724.1 | hypothetical protein | taxID used:364317 OG00567|WP\_020852262.1 | conjugal transfer protein TrbI | taxID used:364317 OG00811|WP\_060870250.1 | hypothetical protein | taxID used:364317 OG00819|WP\_038211569.1 | toxin | taxID used:364317 OG00897|WP\_058564984.1 | P-type DNA transfer ATPase VirB11 | taxID used:364317 OG00962|WP\_058564998.1 | relaxase | taxID used:364317 
species
364317 

1110389 
5


OG01836|WP\_010895199.1 | conjugal transfer protein | taxID used:1110389 OG02767|WP\_058565082.1 | TraY domain-containing protein | taxID used:1110389 OG02909|WP\_081089756.1 | hypothetical protein | taxID used:1110389 OG02934|WP\_010895249.1 | transcriptional regulator | taxID used:1110389 OG03148|WP\_023908207.1 | hypothetical protein | taxID used:1110389 
species

352449
3
genus

180197 
3
species


OG02695|WP\_010895223.1 | type II toxin-antitoxin system PemK/MazF family toxin | taxID used:180197 OG02710|WP\_060872373.1 | type II toxin-antitoxin system PemK/MazF family toxin | taxID used:180197 OG02737|WP\_020851663.1 | transposase | taxID used:180197 

genus
13
665874

2
species


OG02344|WP\_081089723.1 | type II toxin-antitoxin system YafQ family toxin | taxID used:1100724 OG03071|WP\_080702547.1 | type II toxin-antitoxin system antitoxin, RelB/DinJ family | taxID used:1100724 
1100724 

species


OG01813|WP\_004087956.1 | restriction endonuclease subunit S | taxID used:1597955 
1
1597955 

1


OG02941|WP\_011097988.1 | type II toxin-antitoxin system RelE/ParE family toxin | taxID used:1100711 
species
1100711 


OG01157|WP\_081089793.1 | phage tail tape measure protein, partial | taxID used:1835769 OG00166|WP\_004085183.1 | phage tail tape measure protein, partial | taxID used:1835769 OG01706|WP\_126715046.1 | hypothetical protein | taxID used:1835769 OG01709|WP\_023907028.1 | hypothetical protein | taxID used:1835769 OG01861|WP\_004086890.1 | hypothetical protein | taxID used:1835769 OG01883|WP\_081089813.1 | phage tail tape measure protein, partial | taxID used:1835769 OG01956|WP\_004085154.1 | hypothetical protein | taxID used:1835769 OG02238|WP\_088569667.1 | hypothetical protein | taxID used:1835769 OG03309|WP\_076613297.1 | phage major tail tube protein, partial | taxID used:1835769 
species
9
1835769 

genus
12916
8

1276756 
species


OG03584|WP\_080702442.1 | hypothetical protein | taxID used:1276756 
1


OG02602|WP\_012337734.1 | XRE family transcriptional regulator | taxID used:1768795 OG02620|WP\_011098178.1 | DUF5131 family protein | taxID used:1768795 OG03094|WP\_082355533.1 | type II toxin-antitoxin system RelE/ParE family toxin | taxID used:1768795 
species
3
1768795 

species


OG00022|WP\_088572696.1 | cell surface protein | taxID used:1177982 
1
1177982 

1


OG00954|WP\_080654457.1 | AcrB/AcrD/AcrF family protein | taxID used:32040 
species
32040 

1


OG03117|WP\_058564326.1 | hypothetical protein | taxID used:721785 
species
721785 

187868 
1
species


OG02174|WP\_042464096.1 | virulence factor | taxID used:187868 

2
species


OG03075|WP\_060872141.1 | type II toxin-antitoxin system antitoxin, RelB/DinJ family | taxID used:1458426 OG00701|WP\_038211240.1 | ImmA/IrrE family metallo-endopeptidase | taxID used:1458426 
1458426 

genus
34072
1

1034889 
1
species


OG03248|WP\_004091411.1 | DUF433 domain-containing protein | taxID used:1034889 

genus
28065
1

81479 
species


OG00149|WP\_023906984.1 | acyltransferase | taxID used:81479 
1

genus
1
1436289

1436290 
species


OG00696|WP\_012337730.1 | transposase | taxID used:1436290 
1

genus
1
219181

219182 
1
species


OG00581|WP\_023907129.1 | restriction endonuclease subunit S | taxID used:219182 

genus
283
2

species


OG01270|WP\_046419774.1 | phage antirepressor | taxID used:225992 OG01744|WP\_023907749.1 | phage antirepressor | taxID used:225992 
2
225992 

1
281915
genus

1
species


OG02159|WP\_012382560.1 | integral membrane protein | taxID used:80879 
80879 

genus
1
151754

1
species


OG02046|WP\_109160983.1 | hypothetical protein | taxID used:151755 
151755 

family
506
50

1
90243
genus


OG01466|WP\_081089822.1 | phage repressor protein C | taxID used:1581112 
species
1
1581112 


OG01887|WP\_012382680.1 | hypothetical protein | taxID used:222 OG02396|WP\_031336635.1 | DUF2255 domain-containing protein | taxID used:222 OG02849|WP\_020851108.1 | addiction module antidote protein, HigA family | taxID used:222 OG02935|WP\_020851109.1 | plasmid maintenance system killer | taxID used:222 OG03557|WP\_060871946.1 | hypothetical protein | taxID used:222 
genus
9
5
222

32002 
species


OG02389|WP\_080702471.1 | hypothetical protein | taxID used:32002 
1

85698 
2
species


OG01159|WP\_031336630.1 | LysR family transcriptional regulator | taxID used:85698 OG02435|WP\_010894192.1 | LysR family transcriptional regulator | taxID used:85698 

1


OG03022|WP\_060870082.1 | hypothetical protein | taxID used:1389932 
species
1389932 

40


OG01614|WP\_038200659.1 | carboxymuconolactone decarboxylase | taxID used:517 
genus
517
1


OG02041|WP\_023906604.1 | cupin domain-containing protein | taxID used:288768 
species
1
288768 

103855 
2


OG02614|WP\_080502610.1 | hypothetical protein | taxID used:103855 OG00547|WP\_088569647.1 | restriction endonuclease subunit S | taxID used:103855 
species

1


OG02859|WP\_004085590.1 | type II toxin-antitoxin system RelE/ParE family toxin | taxID used:520 
species
520 

518 
35


OG01139|WP\_069107145.1 | ATP-dependent helicase | taxID used:518 OG01601|WP\_031336647.1 | LysR family transcriptional regulator | taxID used:518 OG00167|WP\_088569725.1 | DNA polymerase | taxID used:518 OG01697|WP\_082352344.1 | ATP-dependent helicase | taxID used:518 OG00169|WP\_060871912.1 | DNA polymerase | taxID used:518 OG01772|WP\_020851954.1 | phage antirepressor protein | taxID used:518 OG01774|WP\_020851636.1 | hypothetical protein | taxID used:518 OG01795|WP\_058565061.1 | phage antirepressor protein | taxID used:518 OG01810|WP\_011097944.1 | hypothetical protein | taxID used:518 OG01846|WP\_081090398.1 | DUF2815 domain-containing protein | taxID used:518 OG01847|WP\_020851996.1 | DUF2815 domain-containing protein | taxID used:518 OG01849|WP\_060872417.1 | DUF2815 domain-containing protein | taxID used:518 OG01935|WP\_080679672.1 | ATP-dependent helicase | taxID used:518 OG02692|WP\_081089826.1 | hypothetical protein | taxID used:518 OG02735|WP\_060871766.1 | DUF2815 domain-containing protein, partial | taxID used:518 OG00286|WP\_042463400.1 | hypothetical protein | taxID used:518 OG02923|WP\_020851999.1 | VRR-NUC domain-containing protein | taxID used:518 OG02930|WP\_038230726.1 | VRR-NUC domain-containing protein | taxID used:518 OG02975|WP\_023906378.1 | VRR-NUC domain-containing protein | taxID used:518 OG00430|WP\_058565016.1 | ATP-dependent helicase | taxID used:518 OG00434|WP\_126709119.1 | ATP-dependent helicase | taxID used:518 OG00435|WP\_057682583.1 | ATP-dependent helicase | taxID used:518 OG00438|WP\_060870122.1 | ATP-dependent helicase | taxID used:518 OG00439|WP\_021358707.1 | ATP-dependent helicase | taxID used:518 OG00443|WP\_060872275.1 | ATP-dependent helicase | taxID used:518 OG00445|WP\_060871926.1 | ATP-dependent helicase | taxID used:518 OG00515|WP\_040123263.1 | ATP-dependent helicase | taxID used:518 OG00531|WP\_040123077.1 | ATP-dependent helicase | taxID used:518 OG00533|WP\_060871997.1 | ATP-dependent helicase | taxID used:518 OG00597|WP\_069107164.1 | DUF2800 domain-containing protein | taxID used:518 OG00604|WP\_046419156.1 | DUF2800 domain-containing protein | taxID used:518 OG00685|WP\_060870422.1 | DUF2800 domain-containing protein | taxID used:518 OG00750|WP\_088371691.1 | ATP-dependent helicase | taxID used:518 OG02924|WP\_020850968.1 | VRR-NUC domain-containing protein | taxID used:518 OG00440|WP\_011097622.1 | ATP-dependent helicase | taxID used:518 
species

206389
23
order

family
8
75787

1898103 
2
species


OG01640|WP\_004085312.1 | restriction endonuclease subunit R | taxID used:1898103 OG00860|WP\_004087958.1 | restriction endonuclease subunit R | taxID used:1898103 

genus
857334
6

857335 


OG01144|WP\_088372266.1 | hypothetical protein | taxID used:857335 OG01676|WP\_038211652.1 | antirepressor | taxID used:857335 OG02085|WP\_020852945.1 | DUF488 domain-containing protein | taxID used:857335 OG03160|WP\_081089810.1 | hypothetical protein | taxID used:857335 OG00355|WP\_023907727.1 | antirepressor | taxID used:857335 OG00421|WP\_060872215.1 | DUF2163 domain-containing protein | taxID used:857335 
species
6

1
2008795
family

genus
1
73029

259537 
species


OG00215|WP\_046419976.1 | ATP-binding protein | taxID used:259537 
1

family
2008794
14

genus
1
392735

392736 
1


OG02780|WP\_004091409.1 | hypothetical protein | taxID used:392736 
species

12
33057
genus

species


OG01321|WP\_058565145.1 | DUF3560 domain-containing protein | taxID used:76113 OG01830|WP\_010895240.1 | plasmid mobilization relaxosome protein MobC | taxID used:76113 OG01913|WP\_010895250.1 | hypothetical protein | taxID used:76113 OG02172|WP\_010895243.1 | hypothetical protein | taxID used:76113 OG00219|WP\_010895200.1 | DNA topoisomerase I, plasmid | taxID used:76113 OG02477|WP\_010895241.1 | hypothetical protein | taxID used:76113 OG02586|WP\_069106970.1 | conjugal transfer protein | taxID used:76113 OG03161|WP\_042464024.1 | hypothetical protein | taxID used:76113 OG03166|WP\_010895207.1 | hypothetical protein | taxID used:76113 OG00467|WP\_010895239.1 | nickase | taxID used:76113 OG00703|WP\_010895251.1 | chromosome partitioning protein ParB | taxID used:76113 
11
76113 

1
species


OG02067|WP\_126715051.1 | DUF1376 domain-containing protein, partial | taxID used:1134435 
1134435 

1
12960
genus

1637998 
1
species


OG02672|WP\_004091353.1 | hypothetical protein | taxID used:1637998 

1442155
1
order

family
1
1442156

genus
416212
1

416213 


OG03305|WP\_004091377.1 | CopG family transcriptional regulator | taxID used:416213 
species
1

order
206351
33

family
11
481

genus
1
1193515

1196083 


OG02532|WP\_010893152.1 | hypothetical protein | taxID used:1196083 
species
1

6
422440
genus

422441 


OG01961|WP\_004087886.1 | hypothetical protein | taxID used:422441 OG01969|WP\_010893529.1 | hypothetical protein | taxID used:422441 OG01973|WP\_038210551.1 | hypothetical protein | taxID used:422441 OG01977|WP\_004087884.1 | hypothetical protein | taxID used:422441 OG01990|WP\_071869915.1 | hypothetical protein | taxID used:422441 OG02020|WP\_088578328.1 | hypothetical protein | taxID used:422441 
species
6

482
2
genus

2
species


OG03463|WP\_042836328.1 | hypothetical protein | taxID used:485 OG03608|WP\_075584692.1 | hypothetical protein | taxID used:485 
485 

1654931
2
genus

2
species


OG01853|WP\_004085357.1 | N-6 DNA methylase | taxID used:1452487 OG02488|WP\_004085311.1 | hypothetical protein | taxID used:1452487 
1452487 

family
1499392
22

genus
9
535

8
species


OG02039|WP\_021358700.1 | peptidase | taxID used:238241 OG02044|WP\_020852985.1 | peptidase | taxID used:238241 OG02054|WP\_023907027.1 | peptidase | taxID used:238241 OG02059|WP\_023907985.1 | peptidase | taxID used:238241 OG02089|WP\_004086917.1 | peptidase | taxID used:238241 OG02125|WP\_004086882.1 | peptidase | taxID used:238241 OG02226|WP\_088371695.1 | peptidase | taxID used:238241 OG02236|WP\_060870514.1 | peptidase | taxID used:238241 
238241 

1


OG02205|WP\_014607313.1 | restriction endonuclease | taxID used:1382803 
species
1382803 

genus
187
1

1938604 
1
species


OG03251|WP\_060870075.1 | addiction module antitoxin | taxID used:1938604 

2
57479
genus


OG00400|WP\_004085007.1 | DUF3383 domain-containing protein | taxID used:57480 OG00468|WP\_088371573.1 | DUF3383 domain-containing protein | taxID used:57480 
species
2
57480 

genus


OG02486|WP\_057682544.1 | DUF4224 domain-containing protein | taxID used:407217 OG02811|WP\_010894737.1 | DUF4224 domain-containing protein | taxID used:407217 OG02826|WP\_023906705.1 | DUF4224 domain-containing protein | taxID used:407217 OG03209|WP\_004090683.1 | DUF4224 domain-containing protein | taxID used:407217 OG00831|WP\_023906382.1 | integrase | taxID used:407217 OG00874|WP\_020852652.1 | phage integrase | taxID used:407217 OG00883|WP\_023907725.1 | integrase | taxID used:407217 
7
407217 

genus
3
168470

168471 
3


OG02664|WP\_053014147.1 | cytosolic protein | taxID used:168471 OG00043|WP\_081089912.1 | hypothetical protein | taxID used:168471 OG00909|WP\_004087776.1 | hypothetical protein | taxID used:168471 
species

17
32003
order

family
2
90627

1443590
2
genus


OG01239|WP\_004087203.1 | site-specific integrase | taxID used:1485544 OG02063|WP\_080702484.1 | site-specific integrase | taxID used:1485544 
species
2
1485544 

3
32011
family

genus
3
404

405 


OG01307|WP\_004085309.1 | SAM-dependent DNA methyltransferase | taxID used:405 
species
1

2


OG02809|WP\_046420237.1 | type II toxin-antitoxin system RelE/ParE family toxin | taxID used:551994 OG02866|WP\_004085905.1 | type II toxin-antitoxin system RelE/ParE family toxin | taxID used:551994 
species
551994 

family
206379
12

genus
11
35798


OG00639|WP\_031336128.1 | membrane protein | taxID used:35799 
OG00639|WP\_031336128.1 | membrane protein | taxID used:35799 
species
11
35799 

1232
1
genus

1
species


OG02716|WP\_004084245.1 | hypothetical protein | taxID used:1855339 
1855339 

subphylum
10
68525

class
1
29547

265570
1
genus


OG02112|WP\_023907735.1 | DUF1566 domain-containing protein | taxID used:206403 
species
1
206403 

28221
9
class

2
29
order

80812
2
suborder

2
49
family

2
39643
genus


OG01206|WP\_010894495.1 | aspartyl/asparaginyl beta-hydroxylase domain-containing protein | taxID used:56 OG00824|WP\_010894496.1 | DUF3419 domain-containing protein | taxID used:56 
species
2
56 

order
213118
1

1
213121
family

genus
1
893

53332 
1
species


OG03116|WP\_058565086.1 | type II toxin-antitoxin system Phd/YefM family antitoxin | taxID used:53332 

order
1
69541

family
1
213422

1
392332
genus

483547 
1


OG02478|WP\_088572676.1 | hypothetical protein | taxID used:483547 
species

order
213115
3

family
3
194924

genus
3
872

293256 
3


OG02006|WP\_004085308.1 | XRE family transcriptional regulator | taxID used:293256 OG02117|WP\_081089864.1 | XRE family transcriptional regulator | taxID used:293256 OG00517|WP\_071869901.1 | helicase | taxID used:293256 
species

88274 
1
species


OG02308|WP\_004088056.1 | DUF1566 domain-containing protein | taxID used:88274 

262489 
1
species


OG03183|WP\_050812602.1 | hypothetical protein | taxID used:262489 

3
1236


OG02042|WP\_081392197.1 | peptidase | taxID used:1236 OG02294|WP\_024748685.1 | IS200/IS605 family transposase | taxID used:1236 OG00752|WP\_072866413.1 | phage tail tape measure protein, partial | taxID used:1236 
class
43710422576

order
118969
12

12
444
family

1
465
genus

451 
1
species


OG03143|WP\_004089823.1 | site-specific DNA-methyltransferase | taxID used:451 

11
445
genus

168933 
species


OG00758|WP\_057682994.1 | acyltransferase | taxID used:168933 
OG00758|WP\_057682994.1 | acyltransferase | taxID used:168933 
11

order
1
72273

2212439
1
family

genus
2212691
1

species


OG02587|WP\_012382719.1 | hypothetical protein | taxID used:125614 
1
125614 

9
135618
order

family
2
403

genus
1
413

414 
species


OG03560|WP\_038229438.1 | hypothetical protein | taxID used:414 
1

1
429
genus

1
species


OG03039|WP\_014607314.1 | type II toxin-antitoxin system RelE/ParE family toxin | taxID used:173365 
173365 

7
1486721
family

genus
7
244364

244365 
7


OG01258|WP\_046419912.1 | phage antirepressor | taxID used:244365 OG01637|WP\_060871922.1 | hypothetical protein | taxID used:244365 OG01695|WP\_023906568.1 | phage antirepressor | taxID used:244365 OG01707|WP\_060871942.1 | phage antirepressor protein | taxID used:244365 OG01734|WP\_020852982.1 | phage-related protein | taxID used:244365 OG01760|WP\_020852204.1 | phage-related protein | taxID used:244365 OG02442|WP\_011097946.1 | phage-related protein | taxID used:244365 
species

91347
7
161
order


OG01357|WP\_020851904.1 | hypothetical protein | taxID used:91347 OG02493|WP\_004083562.1 | hypothetical protein | taxID used:91347 OG02580|WP\_004086850.1 | hemagglutinin | taxID used:91347 OG02645|WP\_011098018.1 | hemagglutinin | taxID used:91347 OG02815|WP\_060870177.1 | colicin V synthesis protein | taxID used:91347 OG03390|WP\_071869662.1 | hypothetical protein | taxID used:91347 OG02832|WP\_046417857.1 | colicin V synthesis protein | taxID used:91347 

family


OG00203|WP\_060870093.1 | DNA-directed DNA polymerase | taxID used:543 OG02283|WP\_004086786.1 | transcriptional regulator | taxID used:543 OG02808|WP\_020852063.1 | hypothetical protein | taxID used:543 OG03322|WP\_057683513.1 | hypothetical protein | taxID used:543 
135
4
543

115
561
genus

species


OG02803|WP\_080939611.1 | hemagglutinin | taxID used:208962 
1
208962 

562 
114
species


OG02966|WP\_046420467.1 | hemagglutinin | taxID used:562 
OG02010|WP\_031337790.1 | hypothetical protein | taxID used:562 OG02263|WP\_004086862.1 | DUF596 domain-containing protein | taxID used:562 OG02456|WP\_058570023.1 | DUF596 domain-containing protein | taxID used:562 OG02612|WP\_080507214.1 | hypothetical protein | taxID used:562 OG02669|WP\_004086204.1 | hemagglutinin | taxID used:562 OG02683|WP\_081033548.1 | hemagglutinin | taxID used:562 OG02724|WP\_020851903.1 | hypothetical protein | taxID used:562 OG02741|WP\_031338087.1 | hemagglutinin | taxID used:562 OG02825|WP\_075584702.1 | hemagglutinin | taxID used:562 OG02966|WP\_046420467.1 | hemagglutinin | taxID used:562 OG03058|WP\_012382580.1 | transposase | taxID used:562 OG03153|WP\_060872313.1 | hemagglutinin, partial | taxID used:562 OG03669|WP\_081090400.1 | hemagglutinin, partial | taxID used:562 OG00995|WP\_004086162.1 | hemagglutinin | taxID used:562 

genus
547
2

2


OG01124|WP\_081364548.1 | arginine deaminase | taxID used:354276 
species group
354276 
1

species


OG01379|WP\_088372212.1 | hypothetical protein | taxID used:208224 
1
208224 

568987
1
genus

138072 
1


OG01319|WP\_088372258.1 | hypothetical protein | taxID used:138072 
species

570
3
genus

573 
3


OG02295|WP\_038232460.1 | IS200/IS605 family transposase | taxID used:573 OG00641|WP\_060871686.1 | transposase | taxID used:573 OG00792|WP\_031337009.1 | restriction endonuclease, SacI family | taxID used:573 
species

544
1
genus

species group
1
1344959

546 
1


OG02595|WP\_023908235.1 | hypothetical protein | taxID used:546 
species

genus
1330546
3

1334193 


OG00161|WP\_011098080.1 | autotransporter domain-containing protein | taxID used:1334193 OG00171|WP\_071869863.1 | outer membrane autotransporter barrel | taxID used:1334193 OG00640|WP\_023908193.1 | hemagglutinin | taxID used:701347 
species
3

1
1780190
genus

species


OG01050|WP\_081090411.1 | DNA-directed DNA polymerase, partial | taxID used:1367852 
1
1367852 

genus
5
590

1


OG00636|WP\_042836805.1 | transposase | taxID used:54736 
species
54736 

4
species


OG02633|WP\_060870136.1 | hypothetical protein | taxID used:28901 OG02962|WP\_060870505.1 | hypothetical protein | taxID used:28901 OG03007|WP\_081046868.1 | XRE family transcriptional regulator | taxID used:28901 OG03510|WP\_118853433.1 | hypothetical protein | taxID used:28901 
28901 

1903410
1
family

1
122277
genus

2108399 
1
species


OG01216|WP\_051404210.1 | alpha/beta hydrolase | taxID used:2108399 

family
1903414
4

626
1
4


OG02194|WP\_058565085.1 | PIN domain-containing protein | taxID used:626 
genus


OG00678|WP\_004083651.1 | hypothetical protein | taxID used:1873484 
species
1
1873484 


OG01943|WP\_071869758.1 | hypothetical protein | taxID used:351676 OG02925|WP\_088371422.1 | hypothetical protein | taxID used:351676 
species
2
351676 

1903409
7
family

genus
551
4

796334 


OG02804|WP\_058564432.1 | colicin V synthesis protein | taxID used:796334 OG02820|WP\_004572927.1 | colicin V synthesis protein | taxID used:796334 
species
2

65700 
1
species


OG00164|WP\_058565015.1 | DNA polymerase | taxID used:65700 

182337 
1


OG00917|WP\_060870179.1 | peptidase M4 family protein | taxID used:182337 
species

82986
1
genus

1408192 
1
species


OG01251|WP\_071869653.1 | DUF3289 domain-containing protein | taxID used:1408192 

2
53335
genus

1267600 
1
species


OG02565|WP\_081089730.1 | hypothetical protein | taxID used:1267600 

1
species


OG03029|WP\_046419259.1 | hypothetical protein | taxID used:1465635 
1465635 


OG02489|WP\_004089535.1 | DUF2913 domain-containing protein | taxID used:2025587 
species
1
2025587 

family
1903411
6

genus
1927833
2

2
species


OG01843|WP\_004087326.1 | D-alanyl-D-alanine carboxypeptidase/D-alanyl-D-alanine-endopeptidase | taxID used:1878942 OG00408|WP\_081044406.1 | D-alanyl-D-alanine carboxypeptidase/D-alanyl-D-alanine-endopeptidase | taxID used:1878942 
1878942 

genus
1
1964366

species


OG03249|WP\_024749199.1 | hypothetical protein | taxID used:1917880 
1
1917880 

613
3
genus

615 


OG02846|WP\_004086788.1 | transcriptional regulator | taxID used:615 
species
1

61652 
species


OG02728|WP\_118853466.1 | hypothetical protein | taxID used:61652 OG03532|WP\_081392193.1 | hypothetical protein | taxID used:61652 
2

6
135622
order

family
72275
2

2742
2
genus

2


OG02505|WP\_004088346.1 | hypothetical protein | taxID used:2055143 OG02823|WP\_023906563.1 | hypothetical protein | taxID used:2055143 
species
2055143 

family
4
267889

genus
1518149
4

4
species


OG02978|WP\_023906406.1 | hypothetical protein | taxID used:349064 OG03009|WP\_031337929.1 | hypothetical protein | taxID used:349064 OG03212|WP\_027700451.1 | membrane protein | taxID used:349064 OG03219|WP\_040123227.1 | hypothetical protein | taxID used:349064 
349064 

135619
6
order

family
28256
1

genus
1
2745

1
species


OG02116|WP\_004088357.1 | hypothetical protein | taxID used:176290 
176290 

224372
5
family

5
genus


OG01033|WP\_010892625.1 | fimbrial protein | taxID used:59753 OG00753|WP\_081089841.1 | type 1 fimbrial protein | taxID used:59753 
59753 
2


OG00887|WP\_004087689.1 | type 1 fimbrial protein | taxID used:64988 
species
1
64988 

1
species


OG00868|WP\_031336134.1 | type 1 fimbrial protein | taxID used:1798238 
1798238 

1817793 
1


OG02345|WP\_080939631.1 | hypothetical protein | taxID used:1817793 
species

order
1706369
11

1706371
11
family

10
11
genus

11
species


OG01848|WP\_038210460.1 | hypothetical protein | taxID used:155077 
OG01848|WP\_038210460.1 | hypothetical protein | taxID used:155077 
155077 

order
3
135613

1
1046
family

61593
1
genus

631362 


OG02426|WP\_004089975.1 | XRE family transcriptional regulator | taxID used:631362 
species
1

family
2
72276

2
1051
genus

1396821 


OG02955|WP\_023907014.1 | XRE family transcriptional regulator | taxID used:1396821 
species
1

1


OG02740|WP\_058564631.1 | hypothetical protein | taxID used:421628 
species
421628 

order
72274
173

family
163
135621

subfamily
3
351


OG02944|WP\_060872372.1 | type II toxin-antitoxin system YafQ family toxin | taxID used:352 
genus
3
1
352

species


OG01182|WP\_010894212.1 | daunorubicin C-13 ketoreductase | taxID used:170623 OG03101|WP\_004089517.1 | type II toxin-antitoxin system HicA family toxin | taxID used:170623 
2
170623 

286
4
158
genus


OG02588|WP\_038232956.1 | hypothetical protein | taxID used:286 OG02749|WP\_071869944.1 | DUF4224 domain-containing protein | taxID used:286 OG02904|WP\_031336646.1 | XRE family transcriptional regulator | taxID used:286 OG03555|WP\_023907115.1 | hypothetical protein | taxID used:286 


OG00100|WP\_023907052.1 | DNA primase | taxID used:2075551 OG00101|WP\_023907750.1 | DNA primase | taxID used:2075551 OG00110|WP\_058565007.1 | DNA primase | taxID used:2075551 OG00112|WP\_072866400.1 | phage/plasmid primase P4 | taxID used:2075551 OG00097|WP\_012382624.1 | DNA primase | taxID used:2075551 OG00098|WP\_060872310.1 | DNA primase | taxID used:2075551 OG00099|WP\_042836502.1 | phage/plasmid primase P4 | taxID used:2075551 
species
7
2075551 

2
136849
species group


OG03158|WP\_088569754.1 | type II toxin-antitoxin system RelE/ParE family toxin | taxID used:136849 OG03515|WP\_046420276.1 | hypothetical protein | taxID used:136849 
8

50340 
1
species


OG00842|WP\_004088418.1 | hypothetical protein | taxID used:50340 

2
species subgroup


OG02750|WP\_004089386.1 | type II toxin-antitoxin system RelE/ParE family toxin | taxID used:251698 
251698
1

1
species


OG01142|WP\_010894232.1 | DUF3577 domain-containing protein | taxID used:47877 
47877 

species subgroup
251695
3


OG01440|WP\_104993211.1 | restriction endonuclease subunit S | taxID used:317 OG02948|WP\_057683733.1 | hypothetical protein | taxID used:317 OG00093|WP\_010893407.1 | glycosyltransferase family 1 protein | taxID used:317 
species
3
317 

1
species


OG03513|WP\_081089897.1 | lysozyme | taxID used:216142 
216142 


OG01235|WP\_004083633.1 | type II restriction endonuclease NgoMIV | taxID used:915471 
species
1
915471 

797277 


OG02632|WP\_004085025.1 | toxin-antitoxin system HicB family antitoxin | taxID used:797277 
species
1

1144323 
species


OG03312|WP\_081089815.1 | hypothetical protein | taxID used:1144323 
1

2075548 
species


OG01453|WP\_023906444.1 | hypothetical protein | taxID used:2075548 
1

2


OG01285|WP\_027700598.1 | Restriction endonuclease BglII | taxID used:1582493 OG01510|WP\_027700599.1 | S-adenosylmethionine-binding protein | taxID used:1582493 
species
1582493 

1960828 


OG02230|WP\_031338090.1 | hemagglutinin | taxID used:1960828 
species
1

1


OG02888|WP\_060872041.1 | hypothetical protein | taxID used:321846 
species
321846 


OG02175|WP\_004085106.1 | penicillin-binding protein | taxID used:2009038 
species
1
2009038 

species group
136841
117

53406 


OG01752|WP\_057683790.1 | hypothetical protein | taxID used:53406 
species
1

287 


OG03107|WP\_004084157.1 | hypothetical protein | taxID used:287 
OG01214|WP\_023906215.1 | hypothetical protein | taxID used:287 OG01428|WP\_023906600.1 | ABC transporter substrate-binding protein | taxID used:287 OG01477|WP\_042462852.1 | hypothetical protein | taxID used:287 OG01531|WP\_038232427.1 | hypothetical protein | taxID used:287 OG02142|WP\_010893160.1 | hypothetical protein | taxID used:287 OG02533|WP\_004086687.1 | hypothetical protein | taxID used:287 OG02592|WP\_080939594.1 | DUF262 domain-containing protein | taxID used:287 OG03107|WP\_004084157.1 | hypothetical protein | taxID used:287 OG03197|WP\_031336329.1 | DNA-binding protein | taxID used:287 OG03457|WP\_050765471.1 | restriction endonuclease subunit S | taxID used:287 OG03565|WP\_004090969.1 | hypothetical protein | taxID used:287 OG03626|WP\_120279373.1 | hypothetical protein | taxID used:287 OG00638|WP\_031337835.1 | hypothetical protein | taxID used:287 OG00650|WP\_060871744.1 | restriction endonuclease subunit S | taxID used:287 OG00802|WP\_042463693.1 | antirepressor | taxID used:287 OG00986|WP\_004088917.1 | hypothetical protein | taxID used:287 
species
116

1
136845
species group

1
species


OG00656|WP\_088572588.1 | hemagglutinin | taxID used:303 
303 

7


OG01125|WP\_023906614.1 | LysR family transcriptional regulator | taxID used:1826671 OG01461|WP\_023906610.1 | 5-amino-6-(5-phosphoribosylamino)uracil reductase | taxID used:1826671 OG01665|WP\_010894218.1 | NmrA-like family protein | taxID used:1826671 OG03157|WP\_010894196.1 | 4-oxalocrotonate tautomerase | taxID used:1826671 OG00747|WP\_010894199.1 | MFS transporter | taxID used:1826671 OG00846|WP\_010894194.1 | aldo/keto reductase | taxID used:1826671 OG00890|WP\_010894213.1 | SMP-30/gluconolactonase/LRE family protein | taxID used:1826671 
species
1826671 

1985343 
1


OG00728|WP\_081089837.1 | hemagglutinin | taxID used:1985343 
species

95619 


OG00797|WP\_023906608.1 | alpha/beta hydrolase | taxID used:95619 
species
1

1487922 
1


OG01296|WP\_020852217.1 | DUF2303 domain-containing protein | taxID used:1487922 
species

1
species


OG01600|WP\_057683746.1 | hypothetical protein | taxID used:1736221 
1736221 

2
1849530
genus

1697053 
2


OG02518|WP\_038211603.1 | hypothetical protein | taxID used:1697053 OG03332|WP\_004091106.1 | hypothetical protein | taxID used:1697053 
species

family
10
468

9
genus


OG01945|WP\_010894383.1 | GNAT family N-acetyltransferase | taxID used:469 OG00740|WP\_020850953.1 | phage tail sheath family protein | taxID used:469 
469
2

1217714 
species


OG01968|WP\_004085234.1 | hypothetical protein | taxID used:1217714 OG01983|WP\_004087880.1 | hypothetical protein | taxID used:1217714 OG01992|WP\_060872146.1 | hypothetical protein | taxID used:1217714 OG01994|WP\_012337616.1 | hypothetical protein | taxID used:1217714 OG02137|WP\_058564402.1 | hypothetical protein | taxID used:1217714 
5

species


OG01197|WP\_060872395.1 | phage tail sheath family protein | taxID used:52133 OG01561|WP\_004572885.1 | anthranilate synthase component I family protein | taxID used:52133 
2
52133 

genus
1
475

1775913 
1
species


OG03512|WP\_080507223.1 | hypothetical protein | taxID used:1775913 

order
135624
5

family
5
84642

genus
642
4

3
species


OG03099|WP\_060870188.1 | hypothetical protein | taxID used:1588629 OG03103|WP\_024748592.1 | hypothetical protein | taxID used:1588629 OG03113|WP\_012382619.1 | hypothetical protein | taxID used:1588629 
1588629 

2033032 
species


OG01064|WP\_004085954.1 | hypothetical protein | taxID used:2033032 
1

347533
1
genus

347534 
1
species


OG00172|WP\_004084729.1 | helicase | taxID used:347534 

order
135623
6

6
641
family

657
1
genus


OG03407|WP\_038211656.1 | hypothetical protein | taxID used:1295392 
species
1
1295392 

662
5
genus

species group
717610
2

species


OG02212|WP\_080715113.1 | hypothetical protein | taxID used:670 
1
670 

species


OG03649|WP\_080679660.1 | hypothetical protein | taxID used:696485 
1
696485 

3
species


OG01422|WP\_060870428.1 | site-specific DNA-methyltransferase | taxID used:1307414 OG01696|WP\_082355765.1 | site-specific DNA-methyltransferase | taxID used:1307414 OG01698|WP\_076613612.1 | site-specific DNA-methyltransferase | taxID used:1307414 
1307414 

135614 
43710382395

OG01094|WP\_038230048.1 | 4-hydroxybenzoate octaprenyltransferase | taxID used:135614 OG01001|WP\_004084423.1 | 4-hydroxythreonine-4-phosphate dehydrogenase PdxA | taxID used:135614 OG01005|WP\_020852040.1 | RluA family pseudouridine synthase | taxID used:135614 OG01007|WP\_004090142.1 | DNA-directed RNA polymerase subunit alpha | taxID used:135614 OG01008|WP\_023908084.1 | 16S rRNA (cytosine(1402)-N(4))-methyltransferase RsmH | taxID used:135614 OG01012|WP\_046418737.1 | 23S rRNA pseudouridine(1911/1915/1917) synthase RluD | taxID used:135614 OG01014|WP\_020851606.1 | thiamine-phosphate kinase | taxID used:135614 OG01025|WP\_004083746.1 | thioredoxin-disulfide reductase | taxID used:135614 OG00102|WP\_010892912.1 | ATP-dependent chaperone ClpB | taxID used:135614 OG01031|WP\_038211447.1 | tRNA (adenosine(37)-N6)-dimethylallyltransferase MiaA | taxID used:135614 OG01036|WP\_012337572.1 | acetyl-CoA carboxylase carboxyl transferase subunit alpha | taxID used:135614 OG01045|WP\_012337991.1 | 4-hydroxy-3-methylbut-2-enyl diphosphate reductase | taxID used:135614 OG01052|WP\_024748796.1 | ribose-phosphate diphosphokinase | taxID used:135614 OG01058|WP\_011098115.1 | bifunctional riboflavin kinase/FAD synthetase | taxID used:135614 OG01067|WP\_004085463.1 | glycine--tRNA ligase subunit alpha | taxID used:135614 OG01072|WP\_057683103.1 | RNA polymerase-binding protein DksA | taxID used:135614 OG01075|WP\_088578037.1 | bifunctional biotin--[acetyl-CoA-carboxylase] ligase/biotin operon repressor BirA | taxID used:135614 OG01078|WP\_010893816.1 | rod shape-determining protein MreC | taxID used:135614 OG01087|WP\_004083837.1 | 50S ribosomal protein L3 N(5)-glutamine methyltransferase | taxID used:135614 OG01089|WP\_038228385.1 | prolipoprotein diacylglyceryl transferase | taxID used:135614 OG00108|WP\_040123247.1 | penicillin-binding protein 1A | taxID used:135614 OG01095|WP\_038228650.1 | UDP-3-O-[3-hydroxymyristoyl] N-acetylglucosamine deacetylase | taxID used:135614 OG01096|WP\_004086445.1 | methionyl-tRNA formyltransferase | taxID used:135614 OG01100|WP\_010893095.1 | tRNA 2-thiocytidine(32) synthetase TtcA | taxID used:135614 OG01102|WP\_023907505.1 | ATP phosphoribosyltransferase | taxID used:135614 OG01116|WP\_004089081.1 | acetyl-CoA carboxylase carboxyltransferase subunit beta | taxID used:135614 OG01117|WP\_004083393.1 | 50S ribosomal protein L11 methyltransferase | taxID used:135614 OG01118|WP\_004088630.1 | bis(5'-nucleosyl)-tetraphosphatase (symmetrical) | taxID used:135614 OG00111|WP\_038229293.1 | ribonuclease R | taxID used:135614 OG01123|WP\_046419711.1 | hydroxymethylbilane synthase | taxID used:135614 OG01126|WP\_011098000.1 | ribosomal RNA small subunit methyltransferase A | taxID used:135614 OG01128|WP\_020851593.1 | outer membrane protein assembly factor BamD | taxID used:135614 OG01145|WP\_010894695.1 | GTPase Era | taxID used:135614 OG01158|WP\_023907818.1 | phosphatidylserine decarboxylase | taxID used:135614 OG01161|WP\_004087397.1 | 4-(cytidine 5'-diphospho)-2-C-methyl-D-erythritol kinase | taxID used:135614 OG01165|WP\_024749074.1 | HlyC/CorC family transporter | taxID used:135614 OG00116|WP\_058564307.1 | NAD-dependent DNA ligase LigA | taxID used:135614 OG01175|WP\_027700580.1 | iron-sulfur cluster carrier protein ApbC | taxID used:135614 OG01176|WP\_031336187.1 | tRNA pseudouridine(55) synthase TruB | taxID used:135614 OG01179|WP\_038229813.1 | RNase adapter RapZ | taxID used:135614 OG01192|WP\_010893661.1 | F0F1 ATP synthase subunit gamma | taxID used:135614 OG01203|WP\_088577737.1 | geranyl transferase | taxID used:135614 OG01204|WP\_011098187.1 | helix-turn-helix domain-containing protein | taxID used:135614 OG01205|WP\_046418458.1 | succinate--CoA ligase subunit alpha | taxID used:135614 OG01207|WP\_104993199.1 | RNA polymerase sigma factor RpoH | taxID used:135614 OG01211|WP\_023908120.1 | elongation factor Ts | taxID used:135614 OG01212|WP\_023906622.1 | Hsp33 protein | taxID used:135614 OG01219|WP\_046419544.1 | diaminopimelate epimerase | taxID used:135614 OG01229|WP\_010895042.1 | protease HtpX | taxID used:135614 OG01236|WP\_004086273.1 | pantoate--beta-alanine ligase | taxID used:135614 OG00123|WP\_046418721.1 | DNA topoisomerase 1 | taxID used:135614 OG01242|WP\_038211122.1 | pyrroline-5-carboxylate reductase | taxID used:135614 OG01245|WP\_058564409.1 | nucleoside triphosphate pyrophosphohydrolase | taxID used:135614 OG01249|WP\_046418119.1 | shikimate dehydrogenase | taxID used:135614 OG00124|WP\_088371291.1 | endopeptidase La | taxID used:135614 OG01253|WP\_058569765.1 | NADPH-dependent 7-cyano-7-deazaguanine reductase QueF | taxID used:135614 OG01262|WP\_038211286.1 | cell division protein FtsQ/DivIB | taxID used:135614 OG01264|WP\_004090098.1 | 50S ribosomal protein L2 | taxID used:135614 OG00126|WP\_058569803.1 | DNA topoisomerase (ATP-hydrolyzing) subunit B | taxID used:135614 OG01277|WP\_004085054.1 | bifunctional DNA-formamidopyrimidine glycosylase/DNA-(apurinic or apyrimidinic site) lyase | taxID used:135614 OG00127|WP\_057682436.1 | LPS-assembly protein LptD | taxID used:135614 OG01281|WP\_004084011.1 | 2-dehydro-3-deoxyphosphooctonate aldolase | taxID used:135614 OG01284|WP\_010893560.1 | ribonuclease HII | taxID used:135614 OG01289|WP\_042836391.1 | F0F1 ATP synthase subunit A | taxID used:135614 OG01291|WP\_046419578.1 | peptide chain release factor N(5)-glutamine methyltransferase | taxID used:135614 OG01301|WP\_020851271.1 | thymidylate synthase | taxID used:135614 OG01309|WP\_004083553.1 | 16S rRNA (cytidine(1402)-2'-O)-methyltransferase | taxID used:135614 OG01314|WP\_004083405.1 | 1-(5-phosphoribosyl)-5-[(5-phosphoribosylamino)methylideneamino]imidazole-4-carboxamide isomerase | taxID used:135614 OG01317|WP\_004085053.1 | inner membrane protein YpjD | taxID used:135614 OG01323|WP\_004090321.1 | 30S ribosomal protein S2 | taxID used:135614 OG01324|WP\_004089294.1 | folate-binding protein | taxID used:135614 OG01325|WP\_080939607.1 | exodeoxyribonuclease III | taxID used:135614 OG01326|WP\_011097591.1 | acyl-[acyl-carrier-protein]--UDP-N-acetylglucosamine O-acyltransferase | taxID used:135614 OG01337|WP\_021358311.1 | tRNA (guanosine(37)-N1)-methyltransferase TrmD | taxID used:135614 OG01339|WP\_004090667.1 | tryptophan synthase subunit alpha | taxID used:135614 OG01346|WP\_011098005.1 | hydroxyacylglutathione hydrolase | taxID used:135614 OG01347|WP\_024749251.1 | tRNA pseudouridine(38-40) synthase TruA | taxID used:135614 OG01348|WP\_031336649.1 | YggS family pyridoxal phosphate-dependent enzyme | taxID used:135614 OG01352|WP\_004083605.1 | ABC transporter ATP-binding protein | taxID used:135614 OG01353|WP\_024748784.1 | RNA methyltransferase | taxID used:135614 OG01356|WP\_004572975.1 | indole-3-glycerol phosphate synthase TrpC | taxID used:135614 OG00135|WP\_012337629.1 | outer membrane protein assembly factor BamA | taxID used:135614 OG01368|WP\_010893569.1 | ditrans,polycis-undecaprenyl-diphosphate synthase ((2E,6E)-farnesyl-diphosphate specific) | taxID used:135614 OG00136|WP\_023906998.1 | phenylalanine--tRNA ligase subunit beta | taxID used:135614 OG01376|WP\_057683248.1 | uroporphyrinogen-III synthase | taxID used:135614 OG01377|WP\_004083404.1 | imidazole glycerol phosphate synthase cyclase subunit | taxID used:135614 OG01380|WP\_057682898.1 | 3-deoxy-manno-octulosonate cytidylyltransferase | taxID used:135614 OG01389|WP\_010895106.1 | uracil-DNA glycosylase | taxID used:135614 OG01393|WP\_057682606.1 | NAD kinase | taxID used:135614 OG01412|WP\_058564316.1 | UDP-2,3-diacylglucosamine diphosphatase | taxID used:135614 OG01423|WP\_004090474.1 | tRNA (guanine-N(7)-)-methyltransferase | taxID used:135614 OG01424|WP\_010892691.1 | SPOR domain-containing protein | taxID used:135614 OG01426|WP\_010892612.1 | amidophosphoribosyltransferase | taxID used:135614 OG01431|WP\_004086023.1 | bifunctional 3-demethylubiquinone 3-O-methyltransferase/2-octaprenyl-6-hydroxy phenol methylase | taxID used:135614 OG01436|WP\_027700541.1 | orotidine-5'-phosphate decarboxylase | taxID used:135614 OG01441|WP\_004087940.1 | triose-phosphate isomerase | taxID used:135614 OG01447|WP\_046418442.1 | cell division protein ZipA | taxID used:135614 OG01456|WP\_004091141.1 | 23S rRNA (guanosine(2251)-2'-O)-methyltransferase RlmB | taxID used:135614 OG01467|WP\_057683340.1 | 3-oxoacyl-ACP reductase FabG | taxID used:135614 OG01468|WP\_004083606.1 | ABC transporter permease | taxID used:135614 OG01471|WP\_010892733.1 | 16S rRNA (uracil(1498)-N(3))-methyltransferase | taxID used:135614 OG01473|WP\_027700097.1 | DNA repair protein RecO | taxID used:135614 OG01478|WP\_010893674.1 | 30S ribosomal protein S3 | taxID used:135614 OG01485|WP\_038230128.1 | UMP kinase | taxID used:135614 OG01487|WP\_023906693.1 | ribonuclease PH | taxID used:135614 OG01494|WP\_010893911.1 | LPS export ABC transporter ATP-binding protein | taxID used:135614 OG01496|WP\_010894606.1 | DNA polymerase III subunit epsilon | taxID used:135614 OG01511|WP\_010893622.1 | 4-hydroxy-tetrahydrodipicolinate reductase | taxID used:135614 OG01533|WP\_058569681.1 | lipoyl(octanoyl) transferase LipB | taxID used:135614 OG00153|WP\_058569154.1 | DNA topoisomerase IV subunit A | taxID used:135614 OG01547|WP\_004085576.1 | 7-cyano-7-deazaguanine synthase QueC | taxID used:135614 OG01550|WP\_020851534.1 | 2-C-methyl-D-erythritol 4-phosphate cytidylyltransferase | taxID used:135614 OG01556|WP\_058564800.1 | endonuclease III | taxID used:135614 OG01558|WP\_010895052.1 | 50S ribosomal protein L1 | taxID used:135614 OG01564|WP\_010894034.1 | tRNA (adenosine(37)-N6)-threonylcarbamoyltransferase complex dimerization subunit type 1 TsaB | taxID used:135614 OG01566|WP\_023906310.1 | ribonuclease 3 | taxID used:135614 OG00156|WP\_126715037.1 | primosomal protein N' | taxID used:135614 OG01576|WP\_046417796.1 | ribulose-phosphate 3-epimerase | taxID used:135614 OG01591|WP\_023907432.1 | cytidylate kinase | taxID used:135614 OG01598|WP\_021358244.1 | outer membrane lipoprotein LolB | taxID used:135614 OG01612|WP\_004572852.1 | CvpA family protein | taxID used:135614 OG01613|WP\_010893111.1 | phosphoribosylglycinamide formyltransferase | taxID used:135614 OG01617|WP\_010892698.1 | orotate phosphoribosyltransferase | taxID used:135614 OG01629|WP\_027699991.1 | outer membrane lipoprotein chaperone LolA | taxID used:135614 OG01632|WP\_071869641.1 | RlmE family RNA methyltransferase | taxID used:135614 OG01633|WP\_004091185.1 | ribose-5-phosphate isomerase RpiA | taxID used:135614 OG01634|WP\_081033486.1 | ribosome maturation factor RimP | taxID used:135614 OG01638|WP\_031336908.1 | 50S ribosomal protein L3 | taxID used:135614 OG01642|WP\_031336485.1 | ribonuclease T | taxID used:135614 OG01648|WP\_010894757.1 | CDP-diacylglycerol--glycerol-3-phosphate 3-phosphatidyltransferase | taxID used:135614 OG01658|WP\_038229910.1 | stringent starvation protein A | taxID used:135614 OG01675|WP\_010893439.1 | tRNA threonylcarbamoyladenosine biosynthesis protein RimN | taxID used:135614 OG01679|WP\_004087395.1 | 50S ribosomal protein L25/general stress protein Ctc | taxID used:135614 OG01686|WP\_004572865.1 | ribosomal RNA small subunit methyltransferase G | taxID used:135614 OG01690|WP\_004087911.1 | 16S rRNA (guanine(966)-N(2))-methyltransferase RsmD | taxID used:135614 OG01694|WP\_010893688.1 | 30S ribosomal protein S4 | taxID used:135614 OG01699|WP\_020851188.1 | membrane protein | taxID used:135614 OG01700|WP\_011097655.1 | ATP-dependent Clp protease proteolytic subunit | taxID used:135614 OG01703|WP\_046419569.1 | guanylate kinase | taxID used:135614 OG00170|WP\_046417956.1 | guanosine-3',5'-bis(diphosphate) 3'-diphosphatase | taxID used:135614 OG01722|WP\_031337900.1 | YihA family ribosome biogenesis GTP-binding protein | taxID used:135614 OG01728|WP\_020851236.1 | dephospho-CoA kinase | taxID used:135614 OG01751|WP\_071869539.1 | 50S ribosomal protein L4 | taxID used:135614 OG01753|WP\_010893940.1 | lysogenization protein HflD | taxID used:135614 OG01754|WP\_057682614.1 | 5-formyltetrahydrofolate cyclo-ligase | taxID used:135614 OG01756|WP\_011097758.1 | non-canonical purine NTP pyrophosphatase, RdgB/HAM1 family | taxID used:135614 OG01758|WP\_004084669.1 | Fe-S biogenesis protein NfuA | taxID used:135614 OG01763|WP\_023906328.1 | imidazole glycerol phosphate synthase subunit HisH | taxID used:135614 OG01766|WP\_046417983.1 | RNA pyrophosphohydrolase | taxID used:135614 OG01769|WP\_020852363.1 | oligoribonuclease | taxID used:135614 OG01776|WP\_004084887.1 | recombination protein RecR | taxID used:135614 OG00177|WP\_023906772.1 | bifunctional (p)ppGpp synthetase/guanosine-3',5'-bis(diphosphate) 3'-pyrophosphohydrolase | taxID used:135614 OG00179|WP\_011098330.1 | ribosomal RNA large subunit methyltransferase K/L | taxID used:135614 OG01801|WP\_004087394.1 | aminoacyl-tRNA hydrolase | taxID used:135614 OG01825|WP\_046420118.1 | Holliday junction branch migration protein RuvA | taxID used:135614 OG00182|WP\_004572844.1 | glycine--tRNA ligase subunit beta | taxID used:135614 OG01835|WP\_004085654.1 | DUF615 family protein | taxID used:135614 OG01841|WP\_010894676.1 | YqgE/AlgH family protein | taxID used:135614 OG01856|WP\_004084694.1 | transcription termination/antitermination protein NusG | taxID used:135614 OG00186|WP\_038210656.1 | ATP-dependent DNA helicase RecG | taxID used:135614 OG01897|WP\_020851087.1 | YecA family protein | taxID used:135614 OG00018|WP\_011098326.1 | DNA-directed RNA polymerase subunit beta' | taxID used:135614 OG01900|WP\_004085328.1 | adenylate kinase | taxID used:135614 OG01906|WP\_004089320.1 | ribosome recycling factor | taxID used:135614 OG01909|WP\_004083880.1 | shikimate kinase | taxID used:135614 OG01910|WP\_046418751.1 | transcriptional repressor NrdR | taxID used:135614 OG01916|WP\_004089213.1 | HAD family hydrolase | taxID used:135614 OG01919|WP\_010893678.1 | 50S ribosomal protein L5 | taxID used:135614 OG00191|WP\_088577712.1 | methionine--tRNA ligase | taxID used:135614 OG01936|WP\_004086539.1 | 30S ribosomal protein S5 | taxID used:135614 OG01941|WP\_075584689.1 | translation initiation factor IF-3 | taxID used:135614 OG01946|WP\_004089217.1 | lipopolysaccharide transport periplasmic protein LptA | taxID used:135614 OG01972|WP\_004085641.1 | F0F1 ATP synthase subunit delta | taxID used:135614 OG01975|WP\_010893681.1 | 50S ribosomal protein L6 | taxID used:135614 OG01980|WP\_010892654.1 | ribosome maturation factor RimM | taxID used:135614 OG01981|WP\_004084893.1 | protein-export protein SecB | taxID used:135614 OG01985|WP\_080507231.1 | tRNA adenosine(34) deaminase TadA | taxID used:135614 OG01987|WP\_010894371.1 | crossover junction endodeoxyribonuclease RuvC | taxID used:135614 OG01993|WP\_004084691.1 | 50S ribosomal protein L10 | taxID used:135614 OG01999|WP\_004085850.1 | nucleotide exchange factor GrpE | taxID used:135614 OG00199|WP\_060872247.1 | polyribonucleotide nucleotidyltransferase | taxID used:135614 OG02002|WP\_004085836.1 | SsrA-binding protein SmpB | taxID used:135614 OG02011|WP\_031337936.1 | 2-C-methyl-D-erythritol 2,4-cyclodiphosphate synthase | taxID used:135614 OG02017|WP\_023906584.1 | DUF177 domain-containing protein | taxID used:135614 OG02029|WP\_046420774.1 | dihydrofolate reductase | taxID used:135614 OG02031|WP\_004088821.1 | Rnf electron transport complex subunit RnfB | taxID used:135614 OG02038|WP\_004084756.1 | 5-(carboxyamino)imidazole ribonucleotide mutase | taxID used:135614 OG02064|WP\_010893563.1 | 3-hydroxyacyl-ACP dehydratase FabZ | taxID used:135614 OG02079|WP\_004089607.1 | endoribonuclease YbeY | taxID used:135614 OG02083|WP\_020851923.1 | acetyl-CoA carboxylase biotin carboxyl carrier protein | taxID used:135614 OG02087|WP\_010893499.1 | phosphopantetheine adenylyltransferase | taxID used:135614 OG02090|WP\_010892970.1 | ribosomal-protein-alanine N-acetyltransferase | taxID used:135614 OG02097|WP\_004090587.1 | rod shape-determining protein MreD | taxID used:135614 OG00020|WP\_031337140.1 | DNA-directed RNA polymerase subunit beta | taxID used:135614 OG02100|WP\_004086621.1 | 23S rRNA (pseudouridine(1915)-N(3))-methyltransferase RlmH | taxID used:135614 OG02122|WP\_004084553.1 | tRNA (adenosine(37)-N6)-threonylcarbamoyltransferase complex ATPase subunit type 1 TsaE | taxID used:135614 OG02134|WP\_004085473.1 | tRNA (cytidine(34)-2'-O)-methyltransferase | taxID used:135614 OG02139|WP\_004086409.1 | transcription antitermination factor NusB | taxID used:135614 OG02140|WP\_010895047.1 | 30S ribosomal protein S7 | taxID used:135614 OG02149|WP\_011097633.1 | transcription elongation factor GreA | taxID used:135614 OG02158|WP\_010893664.1 | F0F1 ATP synthase subunit B | taxID used:135614 OG02166|WP\_004083417.1 | Holliday junction resolvase RuvX | taxID used:135614 OG00218|WP\_038229932.1 | excinuclease ABC subunit UvrB | taxID used:135614 OG00221|WP\_046420843.1 | DNA helicase Rep | taxID used:135614 OG02228|WP\_004086143.1 | ribosome silencing factor | taxID used:135614 OG02255|WP\_031337016.1 | ClpXP protease specificity-enhancing factor | taxID used:135614 OG02260|WP\_004090345.1 | 30S ribosomal protein S6 | taxID used:135614 OG02264|WP\_010894980.1 | 50S ribosomal protein L9 | taxID used:135614 OG02274|WP\_058569577.1 | DNA polymerase III subunit chi | taxID used:135614 OG02275|WP\_031336902.1 | 50S ribosomal protein L15 | taxID used:135614 OG02293|WP\_004083669.1 | 50S ribosomal protein L13 | taxID used:135614 OG02310|WP\_027700564.1 | 50S ribosomal protein L11 | taxID used:135614 OG02318|WP\_004083578.1 | nucleoside-diphosphate kinase | taxID used:135614 OG00231|WP\_004084578.1 | threonine--tRNA ligase | taxID used:135614 OG02336|WP\_004086531.1 | 50S ribosomal protein L16 | taxID used:135614 OG02338|WP\_004090067.1 | F0F1 ATP synthase subunit epsilon | taxID used:135614 OG02350|WP\_011098077.1 | ferric iron uptake transcriptional regulator | taxID used:135614 OG02359|WP\_010895054.1 | preprotein translocase subunit SecE | taxID used:135614 OG02366|WP\_075584703.1 | ribonuclease P protein component | taxID used:135614 OG02374|WP\_004085540.1 | 50S ribosomal protein L19 | taxID used:135614 OG00237|WP\_057683503.1 | ABC transporter ATP-binding protein | taxID used:135614 OG00023|WP\_011097732.1 | phosphoribosylformylglycinamidine synthase | taxID used:135614 OG02401|WP\_004085841.1 | outer membrane protein assembly factor BamE | taxID used:135614 OG02408|WP\_004086543.1 | 30S ribosomal protein S11 | taxID used:135614 OG02410|WP\_004087939.1 | preprotein translocase subunit SecG | taxID used:135614 OG02416|WP\_004086536.1 | 30S ribosomal protein S8 | taxID used:135614 OG00242|WP\_060872172.1 | molecular chaperone HtpG | taxID used:135614 OG00243|WP\_004083434.1 | 1-deoxy-D-xylulose-5-phosphate synthase | taxID used:135614 OG02444|WP\_004083670.1 | 30S ribosomal protein S9 | taxID used:135614 OG00246|WP\_058564962.1 | tRNA uridine-5-carboxymethylaminomethyl(34) synthesis enzyme MnmG | taxID used:135614 OG00247|WP\_057683363.1 | DNA topoisomerase IV subunit B | taxID used:135614 OG02482|WP\_010893689.1 | 50S ribosomal protein L17 | taxID used:135614 OG02497|WP\_010892777.1 | 30S ribosome-binding factor RbfA | taxID used:135614 OG02507|WP\_004084687.1 | 30S ribosomal protein S12 | taxID used:135614 OG00250|WP\_046420641.1 | molecular chaperone DnaK | taxID used:135614 OG02514|WP\_004083551.1 | YraN family protein | taxID used:135614 OG02535|WP\_031336149.1 | Spx/MgsR family RNA polymerase-binding regulatory protein | taxID used:135614 OG02536|WP\_012382427.1 | preprotein translocase subunit YajC | taxID used:135614 OG02537|WP\_004086533.1 | 50S ribosomal protein L14 | taxID used:135614 OG02546|WP\_011097688.1 | cell division protein FtsB | taxID used:135614 OG02547|WP\_010893263.1 | 50S ribosomal protein L20 | taxID used:135614 OG02558|WP\_004086542.1 | 30S ribosomal protein S13 | taxID used:135614 OG00257|WP\_038228283.1 | excinuclease ABC subunit UvrC | taxID used:135614 OG02583|WP\_010895050.1 | 50S ribosomal protein L7/L12 | taxID used:135614 OG00258|WP\_038228562.1 | ABC transporter ATP-binding protein | taxID used:135614 OG02596|WP\_004086538.1 | 50S ribosomal protein L18 | taxID used:135614 OG00259|WP\_014607668.1 | RNA polymerase sigma factor RpoD | taxID used:135614 OG02603|WP\_020852709.1 | thioredoxin | taxID used:135614 OG00263|WP\_004090429.1 | DNA mismatch repair protein MutL | taxID used:135614 OG02667|WP\_010893673.1 | 50S ribosomal protein L22 | taxID used:135614 OG00269|WP\_004087662.1 | dihydroxy-acid dehydratase | taxID used:135614 OG00026|WP\_038227928.1 | UvrD/REP helicase | taxID used:135614 OG02714|WP\_004088409.1 | 50S ribosomal protein L21 | taxID used:135614 OG02722|WP\_004572794.1 | YbaB/EbfC family nucleoid-associated protein | taxID used:135614 OG02739|WP\_004086577.1 | 50S ribosomal protein L24 | taxID used:135614 OG00273|WP\_038232083.1 | single-stranded-DNA-specific exonuclease RecJ | taxID used:135614 OG02746|WP\_004084751.1 | Grx4 family monothiol glutaredoxin | taxID used:135614 OG02756|WP\_004085973.1 | integration host factor subunit beta | taxID used:135614 OG00275|WP\_004090207.1 | translational GTPase TypA | taxID used:135614 OG00276|WP\_023907197.1 | glutamine--fructose-6-phosphate aminotransferase | taxID used:135614 OG02770|WP\_004090086.1 | 30S ribosomal protein S10 | taxID used:135614 OG00277|WP\_012382432.1 | RNA helicase | taxID used:135614 OG02794|WP\_004086535.1 | 30S ribosomal protein S14 | taxID used:135614 OG02802|WP\_058569559.1 | YhbY family RNA-binding protein | taxID used:135614 OG02828|WP\_012337685.1 | 50S ribosomal protein L23 | taxID used:135614 OG02830|WP\_010894003.1 | DNA-directed RNA polymerase subunit omega | taxID used:135614 OG02850|WP\_004084568.1 | integration host factor subunit alpha | taxID used:135614 OG00285|WP\_057682928.1 | elongation factor 4 | taxID used:135614 OG02880|WP\_024748689.1 | F0F1 ATP synthase subunit C | taxID used:135614 OG02928|WP\_004088683.1 | molecular chaperone GroES | taxID used:135614 OG00292|WP\_088578238.1 | glutamine--tRNA ligase/YqeY domain fusion protein | taxID used:135614 OG02938|WP\_004084034.1 | DUF493 domain-containing protein | taxID used:135614 OG00293|WP\_023907627.1 | aspartate--tRNA ligase | taxID used:135614 OG02951|WP\_011097829.1 | Fe(2+)-trafficking protein | taxID used:135614 OG02970|WP\_004085558.1 | RNA-binding protein Hfq | taxID used:135614 OG02973|WP\_004086525.1 | 30S ribosomal protein S19 | taxID used:135614 OG00297|WP\_031336883.1 | outer membrane protein assembly factor | taxID used:135614 OG03001|WP\_004085493.1 | Fis family transcriptional regulator | taxID used:135614 OG03020|WP\_024749209.1 | 30S ribosomal protein S17 | taxID used:135614 OG00302|WP\_046418009.1 | DNA primase | taxID used:135614 OG03060|WP\_010894866.1 | 30S ribosomal protein S20 | taxID used:135614 OG03067|WP\_004084487.1 | cell division protein FtsL | taxID used:135614 OG00306|WP\_046417565.1 | membrane protein insertase YidC | taxID used:135614 OG03083|WP\_004086259.1 | 30S ribosomal protein S15 | taxID used:135614 OG03085|WP\_004087643.1 | 30S ribosomal protein S16 | taxID used:135614 OG03104|WP\_060871965.1 | succinate dehydrogenase assembly factor 2 family protein | taxID used:135614 OG03118|WP\_004085949.1 | 50S ribosomal protein L27 | taxID used:135614 OG03132|WP\_004086547.1 | accessory factor UbiK family protein | taxID used:135614 OG03174|WP\_004083672.1 | type B 50S ribosomal protein L31 | taxID used:135614 OG00318|WP\_024748809.1 | transcription termination factor Rho | taxID used:135614 OG00319|WP\_038233155.1 | proline--tRNA ligase | taxID used:135614 OG03217|WP\_010894792.1 | RnfH family protein | taxID used:135614 OG03236|WP\_004083472.1 | MULTISPECIES: acyl carri | taxID used:135614 OG03237|WP\_004086565.1 | 50S ribosomal protein L28 | taxID used:135614 OG00326|WP\_023907466.1 | DNA repair protein RecN | taxID used:135614 OG03287|WP\_010894981.1 | 30S ribosomal protein S18 | taxID used:135614 OG00032|WP\_058564412.1 | DNA polymerase III subunit alpha | taxID used:135614 OG00331|WP\_004090560.1 | CTP synthetase | taxID used:135614 OG00334|WP\_046420038.1 | ubiquinone biosynthesis regulatory protein kinase UbiB | taxID used:135614 OG03354|WP\_004083749.1 | translation initiation factor IF-1 | taxID used:135614 OG00335|WP\_057683315.1 | 30S ribosomal protein S1 | taxID used:135614 OG00336|WP\_011098235.1 | energy-dependent translational throttle protein EttA | taxID used:135614 OG03388|WP\_004083596.1 | 30S ribosomal protein S21 | taxID used:135614 OG00033|WP\_023906015.1 | transcription-repair coupling factor | taxID used:135614 OG03464|WP\_031345749.1 | 50S ribosomal protein L29 | taxID used:135614 OG00349|WP\_004088684.1 | molecular chaperone GroEL | taxID used:135614 OG00351|WP\_010895110.1 | RNA helicase | taxID used:135614 OG03530|WP\_010894283.1 | 50S ribosomal protein L32 | taxID used:135614 OG03545|WP\_075584665.1 | 50S ribosomal protein L30 | taxID used:135614 OG00356|WP\_004087188.1 | peptide chain release factor 3 | taxID used:135614 OG00360|WP\_020851064.1 | bifunctional phosphoribosylaminoimidazolecarboxamide formyltransferase/IMP cyclohydrolase PurH | taxID used:135614 OG00363|WP\_038283983.1 | murein biosynthesis integral membrane protein MurJ | taxID used:135614 OG03647|WP\_004086566.1 | 50S ribosomal protein L33 | taxID used:135614 OG00364|WP\_057683313.1 | GMP synthase (glutamine-hydrolyzing) | taxID used:135614 OG00036|WP\_010895025.1 | Rne/Rng family ribonuclease | taxID used:135614 OG00037|WP\_126715034.1 | exodeoxyribonuclease V subunit gamma | taxID used:135614 OG00382|WP\_010893662.1 | F0F1 ATP synthase subunit alpha | taxID used:135614 OG00389|WP\_004085668.1 | lysine--tRNA ligase | taxID used:135614 OG00394|WP\_010892710.1 | MFS transporter | taxID used:135614 OG00397|WP\_058569363.1 | tRNA (N6-isopentenyl adenosine(37)-C2)-methylthiotransferase MiaB | taxID used:135614 OG00402|WP\_010892773.1 | glucose-6-phosphate isomerase | taxID used:135614 OG00404|WP\_004572953.1 | transcription termination/antitermination protein NusA | taxID used:135614 OG00409|WP\_058564509.1 | Rne/Rng family ribonuclease | taxID used:135614 OG00414|WP\_081364544.1 | UDP-N-acetylmuramoyl-L-alanyl-D-glutamate--2,6-diaminopimelate ligase | taxID used:135614 OG00415|WP\_004085038.1 | adenosylmethionine--8-amino-7-oxononanoate transaminase | taxID used:135614 OG00416|WP\_038229315.1 | amidophosphoribosyltransferase | taxID used:135614 OG00419|WP\_058569579.1 | leucyl aminopeptidase | taxID used:135614 OG00041|WP\_057682882.1 | carbamoyl-phosphate synthase large subunit | taxID used:135614 OG00432|WP\_046418200.1 | IMP dehydrogenase | taxID used:135614 OG00442|WP\_010894897.1 | ribosomal large subunit pseudouridine synthase B | taxID used:135614 OG00448|WP\_031336960.1 | cysteine--tRNA ligase | taxID used:135614 OG00453|WP\_023906105.1 | UDP-N-acetylmuramate:L-alanyl-gamma-D-glutamyl-meso-diaminopimelate ligase | taxID used:135614 OG00457|WP\_020851208.1 | UDP-N-acetylmuramate--L-alanine ligase | taxID used:135614 OG00461|WP\_004084450.1 | glutamate--tRNA ligase | taxID used:135614 OG00478|WP\_010893660.1 | F0F1 ATP synthase subunit beta | taxID used:135614 OG00485|WP\_046420507.1 | histidine--tRNA ligase | taxID used:135614 OG00490|WP\_038227947.1 | ribosome biogenesis GTPase Der | taxID used:135614 OG00493|WP\_057682418.1 | UDP-N-acetylmuramoylalanyl-D-glutamyl-2, 6-diaminopimelate--D-alanyl-D-alanine ligase | taxID used:135614 OG00496|WP\_031336811.1 | DNA repair protein RadA | taxID used:135614 OG00507|WP\_010893685.1 | preprotein translocase subunit SecY | taxID used:135614 OG00508|WP\_046419044.1 | bifunctional N-acetylglucosamine-1-phosphate uridyltransferase/glucosamine-1-phosphate acetyltransferase | taxID used:135614 OG00512|WP\_027700554.1 | adenylosuccinate lyase | taxID used:135614 OG00514|WP\_023906726.1 | replication-associated recombination protein A | taxID used:135614 OG00518|WP\_011097494.1 | 3-deoxy-D-manno-octulosonic acid transferase | taxID used:135614 OG00520|WP\_020852871.1 | signal recognition particle protein | taxID used:135614 OG00535|WP\_027700579.1 | glutamate-1-semialdehyde 2,1-aminomutase | taxID used:135614 OG00552|WP\_010892635.1 | GTPase HflX | taxID used:135614 OG00553|WP\_046418128.1 | tRNA lysidine(34) synthetase TilS | taxID used:135614 OG00555|WP\_004085214.1 | RIP metalloprotease RseP | taxID used:135614 OG00558|WP\_038228626.1 | exodeoxyribonuclease VII large subunit | taxID used:135614 OG00055|WP\_004087603.1 | valine--tRNA ligase | taxID used:135614 OG00560|WP\_046418225.1 | chromosomal replication initiator protein DnaA | taxID used:135614 OG00562|WP\_023906495.1 | Tol-Pal system beta propeller repeat protein TolB | taxID used:135614 OG00565|WP\_010893969.1 | phosphoglucosamine mutase | taxID used:135614 OG00574|WP\_023906462.1 | phosphoribosylamine--glycine ligase | taxID used:135614 OG00590|WP\_057682345.1 | glutamyl-tRNA reductase | taxID used:135614 OG00593|WP\_004083580.1 | adenylosuccinate synthase | taxID used:135614 OG00598|WP\_004083457.1 | serine--tRNA ligase | taxID used:135614 OG00599|WP\_004086551.1 | trigger factor | taxID used:135614 OG00602|WP\_027700705.1 | bifunctional tetrahydrofolate synthase/dihydrofolate synthase | taxID used:135614 OG00605|WP\_023906922.1 | ribosomal RNA small subunit methyltransferase B | taxID used:135614 OG00614|WP\_010894994.1 | flavodoxin-dependent (E)-4-hydroxy-3-methylbut-2-enyl-diphosphate synthase | taxID used:135614 OG00617|WP\_057683365.1 | phosphopyruvate hydratase | taxID used:135614 OG00618|WP\_038228891.1 | ATP-dependent Clp protease ATP-binding subunit ClpX | taxID used:135614 OG00620|WP\_038211284.1 | putative lipid II flippase FtsW | taxID used:135614 OG00623|WP\_004083770.1 | UDP-N-acetylglucosamine 1-carboxyvinyltransferase | taxID used:135614 OG00624|WP\_046419397.1 | multifunctional CCA tRNA nucleotidyl transferase/2'3'-cyclic phosphodiesterase/2'nucleotidase/phosphatase | taxID used:135614 OG00062|WP\_058569525.1 | excinuclease ABC subunit A | taxID used:135614 OG00630|WP\_004086282.1 | polynucleotide adenylyltransferase PcnB | taxID used:135614 OG00649|WP\_012382717.1 | 23S rRNA (adenine(2503)-C(2))-methyltransferase RlmN | taxID used:135614 OG00651|WP\_004083471.1 | beta-ketoacyl-[acyl-carrier-protein] synthase II | taxID used:135614 OG00653|WP\_004084469.1 | cell division protein FtsA | taxID used:135614 OG00655|WP\_010894654.1 | phosphoglycerate dehydrogenase | taxID used:135614 OG00065|WP\_020852507.1 | isoleucine--tRNA ligase | taxID used:135614 OG00667|WP\_058569584.1 | bifunctional phosphopantothenoylcysteine decarboxylase/phosphopantothenate--cysteine ligase CoaBC | taxID used:135614 OG00066|WP\_046419622.1 | 2-oxoglutarate dehydrogenase E1 component | taxID used:135614 OG00675|WP\_004090511.1 | cell division protein FtsZ | taxID used:135614 OG00679|WP\_010892922.1 | S-adenosylmethionine synthase | taxID used:135614 OG00719|WP\_046420832.1 | 5-(carboxyamino)imidazole ribonucleotide synthase | taxID used:135614 OG00733|WP\_010894376.1 | signal recognition particle-docking protein FtsY | taxID used:135614 OG00735|WP\_004089326.1 | 1-deoxy-D-xylulose-5-phosphate reductoisomerase | taxID used:135614 OG00741|WP\_004090622.1 | 3-dehydroquinate synthase | taxID used:135614 OG00742|WP\_088577634.1 | tRNA guanosine(34) transglycosylase Tgt | taxID used:135614 OG00743|WP\_020852152.1 | YggW family oxidoreductase | taxID used:135614 OG00749|WP\_011097736.1 | tRNA 2-thiouridine(34) synthase MnmA | taxID used:135614 OG00074|WP\_010893327.1 | protein translocase subunit SecA | taxID used:135614 OG00760|WP\_104993207.1 | lipid-A-disaccharide synthase | taxID used:135614 OG00076|WP\_057683342.1 | pyruvate dehydrogenase (acetyl-transferring), homodimeric type | taxID used:135614 OG00770|WP\_031337074.1 | phosphoglycerate kinase | taxID used:135614 OG00771|WP\_004090371.1 | succinyl-CoA ligase subunit beta | taxID used:135614 OG00772|WP\_038229707.1 | anhydro-N-acetylmuramic acid kinase | taxID used:135614 OG00774|WP\_038229914.1 | DNA-protecting protein DprA | taxID used:135614 OG00776|WP\_010893775.1 | lipoyl synthase | taxID used:135614 OG00779|WP\_010894665.1 | bifunctional histidinol-phosphatase/imidazoleglycerol-phosphate dehydratase | taxID used:135614 OG00782|WP\_057682978.1 | succinyl-diaminopimelate desuccinylase | taxID used:135614 OG00786|WP\_031336924.1 | carbamoyl-phosphate synthase small subunit | taxID used:135614 OG00796|WP\_071869544.1 | peptide chain release factor 2 | taxID used:135614 OG00803|WP\_023906755.1 | chorismate synthase | taxID used:135614 OG00804|WP\_057683270.1 | molecular chaperone DnaJ | taxID used:135614 OG00808|WP\_010892552.1 | DNA replication and repair protein RecF | taxID used:135614 OG00809|WP\_046417730.1 | LPS export ABC transporter permease LptG | taxID used:135614 OG00081|WP\_004086138.1 | leucine--tRNA ligase | taxID used:135614 OG00829|WP\_011098070.1 | phosphoserine transaminase | taxID used:135614 OG00082|WP\_004084616.1 | DNA gyrase subunit A | taxID used:135614 OG00839|WP\_010892551.1 | DNA polymerase III subunit beta | taxID used:135614 OG00845|WP\_058569486.1 | undecaprenyldiphospho-muramoylpentapeptide beta-N-acetylglucosaminyltransferase | taxID used:135614 OG00848|WP\_010893316.1 | phospho-N-acetylmuramoyl-pentapeptide-transferase | taxID used:135614 OG00084|WP\_027700368.1 | alanine--tRNA ligase | taxID used:135614 OG00852|WP\_012337564.1 | LPS export ABC transporter permease LptF | taxID used:135614 OG00853|WP\_004087401.1 | peptide chain release factor 1 | taxID used:135614 OG00855|WP\_004089675.1 | bifunctional diaminohydroxyphosphoribosylaminopyrimidine deaminase/5-amino-6-(5-phosphoribosylamino)uracil reductase RibD | taxID used:135614 OG00085|WP\_010892776.1 | translation initiation factor IF-2 | taxID used:135614 OG00861|WP\_046420808.1 | redox-regulated ATPase YchF | taxID used:135614 OG00867|WP\_057683309.1 | GTPase ObgE | taxID used:135614 OG00869|WP\_031336560.1 | A/G-specific adenine glycosylase | taxID used:135614 OG00873|WP\_010893837.1 | uroporphyrinogen decarboxylase | taxID used:135614 OG00876|WP\_004085791.1 | 3-isopropylmalate dehydrogenase | taxID used:135614 OG00885|WP\_004084645.1 | UDP-N-acetylenolpyruvoylglucosamine reductase | taxID used:135614 OG00892|WP\_027700304.1 | phosphoribosylformylglycinamidine cyclo-ligase | taxID used:135614 OG00894|WP\_049767181.1 | endolytic transglycosylase MltG | taxID used:135614 OG00903|WP\_004090333.1 | quinone-dependent dihydroorotate dehydrogenase | taxID used:135614 OG00910|WP\_042463646.1 | S-adenosylmethionine:tRNA ribosyltransferase-isomerase | taxID used:135614 OG00912|WP\_038211538.1 | biotin synthase BioB | taxID used:135614 OG00916|WP\_060871962.1 | tetraacyldisaccharide 4'-kinase | taxID used:135614 OG00920|WP\_004083595.1 | tRNA (adenosine(37)-N6)-threonylcarbamoyltransferase complex transferase subunit TsaD | taxID used:135614 OG00925|WP\_071869658.1 | rod shape-determining protein | taxID used:135614 OG00926|WP\_046417698.1 | DNA recombination/repair protein RecA | taxID used:135614 OG00928|WP\_023906078.1 | anthranilate phosphoribosyltransferase | taxID used:135614 OG00929|WP\_020852809.1 | tRNA dihydrouridine(20/20a) synthase DusA | taxID used:135614 OG00933|WP\_027700744.1 | glycerol-3-phosphate dehydrogenase | taxID used:135614 OG00935|WP\_020851363.1 | DNA polymerase III subunit delta | taxID used:135614 OG00949|WP\_004085423.1 | Holliday junction branch migration DNA helicase RuvB | taxID used:135614 OG00094|WP\_088578586.1 | DNA mismatch repair protein MutS | taxID used:135614 OG00959|WP\_004085216.1 | UDP-3-O-(3-hydroxymyristoyl)glucosamine N-acyltransferase | taxID used:135614 OG00973|WP\_038228746.1 | phenylalanine--tRNA ligase subunit alpha | taxID used:135614 OG00979|WP\_004083792.1 | trans-hexaprenyltranstransferase | taxID used:135614 OG00999|WP\_031337572.1 | ferrochelatase | taxID used:135614 
OG01557|WP\_027700691.1 | DUF3426 domain-containing protein | taxID used:135614 OG02381|WP\_010893418.1 | hypothetical protein | taxID used:135614 OG02600|WP\_004084535.1 | ArsR family transcriptional regulator | taxID used:135614 OG02742|WP\_038200376.1 | membrane protein | taxID used:135614 OG00301|WP\_071869704.1 | sulfonate ABC transporter permease | taxID used:135614 OG00923|WP\_004087145.1 | ParB/RepB/Spo0J family partition protein | taxID used:135614 OG01000|WP\_011097726.1 | ubiquinol oxidase subunit II | taxID used:135614 OG01001|WP\_004084423.1 | 4-hydroxythreonine-4-phosphate dehydrogenase PdxA | taxID used:135614 OG01002|WP\_031336621.1 | uroporphyrin-III C-methyltransferase | taxID used:135614 OG01004|WP\_004083763.1 | site-specific tyrosine recombinase XerD | taxID used:135614 OG01006|WP\_010893424.1 | PhoH family protein | taxID used:135614 OG01007|WP\_004090142.1 | DNA-directed RNA polymerase subunit alpha | taxID used:135614 OG01008|WP\_023908084.1 | 16S rRNA (cytosine(1402)-N(4))-methyltransferase RsmH | taxID used:135614 OG01012|WP\_046418737.1 | 23S rRNA pseudouridine(1911/1915/1917) synthase RluD | taxID used:135614 OG01013|WP\_023906990.1 | DUF1684 domain-containing protein | taxID used:135614 OG01016|WP\_058564676.1 | LysR family transcriptional regulator | taxID used:135614 OG01017|WP\_024748785.1 | DNA polymerase III subunit delta' | taxID used:135614 OG01018|WP\_023907647.1 | DUF58 domain-containing protein | taxID used:135614 OG01019|WP\_004086600.1 | aldo/keto reductase | taxID used:135614 OG01020|WP\_004085451.1 | SPOR domain-containing protein | taxID used:135614 OG01024|WP\_023907715.1 | prolyl aminopeptidase | taxID used:135614 OG01025|WP\_004083746.1 | thioredoxin-disulfide reductase | taxID used:135614 OG01026|WP\_088371850.1 | tellurium resistance protein TerC | taxID used:135614 OG01031|WP\_038211447.1 | tRNA (adenosine(37)-N6)-dimethylallyltransferase MiaA | taxID used:135614 OG01034|WP\_004084779.1 | hypothetical protein | taxID used:135614 OG01035|WP\_010894591.1 | phosphate ABC transporter permease subunit PstC | taxID used:135614 OG01036|WP\_012337572.1 | acetyl-CoA carboxylase carboxyl transferase subunit alpha | taxID used:135614 OG01037|WP\_057682909.1 | MBL fold hydrolase | taxID used:135614 OG00103|WP\_010893707.1 | ribonucleoside-diphosphate reductase subunit alpha | taxID used:135614 OG01040|WP\_023907069.1 | EF-P lysine aminoacylase GenX | taxID used:135614 OG01041|WP\_004083864.1 | sulfate ABC transporter permease subunit CysW | taxID used:135614 OG01043|WP\_004090506.1 | D-alanine--D-alanine ligase | taxID used:135614 OG01046|WP\_057682432.1 | cysteine synthase A | taxID used:135614 OG01048|WP\_020851934.1 | pilus assembly protein PilW | taxID used:135614 OG01051|WP\_004083414.1 | homoserine kinase | taxID used:135614 OG01053|WP\_004085460.1 | glutathione synthase | taxID used:135614 OG01054|WP\_031336446.1 | aspartate carbamoyltransferase catalytic subunit | taxID used:135614 OG01055|WP\_004084986.1 | FKBP-type peptidyl-prolyl cis-trans isomerase | taxID used:135614 OG01059|WP\_004083779.1 | HPr kinase/phosphorylase | taxID used:135614 OG01062|WP\_057683341.1 | [acyl-carrier-protein] S-malonyltransferase | taxID used:135614 OG01063|WP\_057682734.1 | ribokinase | taxID used:135614 OG01066|WP\_020851797.1 | LpxL/LpxP family Kdo(2)-lipid IV(A) lauroyl/palmitoleoyl acyltransferase | taxID used:135614 OG01067|WP\_004085463.1 | glycine--tRNA ligase subunit alpha | taxID used:135614 OG01068|WP\_023908118.1 | EamA/RhaT family transporter | taxID used:135614 OG01071|WP\_058564428.1 | electron transfer flavoprotein subunit alpha/FixB family protein | taxID used:135614 OG01072|WP\_057683103.1 | RNA polymerase-binding protein DksA | taxID used:135614 OG01075|WP\_088578037.1 | bifunctional biotin--[acetyl-CoA-carboxylase] ligase/biotin operon repressor BirA | taxID used:135614 OG01076|WP\_004083674.1 | LysR family transcriptional regulator | taxID used:135614 OG01077|WP\_023906075.1 | phosphoribosylaminoimidazolesuccinocarboxamide synthase | taxID used:135614 OG01078|WP\_010893816.1 | rod shape-determining protein MreC | taxID used:135614 OG01081|WP\_010894280.1 | MoxR family ATPase | taxID used:135614 OG01082|WP\_004086576.1 | ABC transporter ATP-binding protein | taxID used:135614 OG01083|WP\_024748769.1 | sulfate adenylyltransferase subunit CysD | taxID used:135614 OG01085|WP\_058564505.1 | phosphoglycerate mutase | taxID used:135614 OG01087|WP\_004083837.1 | 50S ribosomal protein L3 N(5)-glutamine methyltransferase | taxID used:135614 OG01088|WP\_058564347.1 | ABC transporter permease | taxID used:135614 OG01089|WP\_038228385.1 | prolipoprotein diacylglyceryl transferase | taxID used:135614 OG00108|WP\_040123247.1 | penicillin-binding protein 1A | taxID used:135614 OG01091|WP\_004087323.1 | SAM-dependent methyltransferase | taxID used:135614 OG01093|WP\_027700312.1 | GDP-6-deoxy-D-lyxo-4-hexulose reductase | taxID used:135614 OG01096|WP\_004086445.1 | methionyl-tRNA formyltransferase | taxID used:135614 OG01098|WP\_020850906.1 | ion transporter | taxID used:135614 OG01099|WP\_004083985.1 | MCE family protein | taxID used:135614 OG01100|WP\_010893095.1 | tRNA 2-thiocytidine(32) synthetase TtcA | taxID used:135614 OG01102|WP\_023907505.1 | ATP phosphoribosyltransferase | taxID used:135614 OG01105|WP\_021358444.1 | hypothetical protein | taxID used:135614 OG01107|WP\_031345833.1 | chromosome partitioning protein ParB | taxID used:135614 OG01108|WP\_004090646.1 | protoheme IX farnesyltransferase | taxID used:135614 OG01110|WP\_004087373.1 | mechanosensitive ion channel protein MscS | taxID used:135614 OG01113|WP\_023906817.1 | LysR family transcriptional regulator | taxID used:135614 OG01115|WP\_004087631.1 | 2,3,4,5-tetrahydropyridine-2,6-dicarboxylate N-succinyltransferase | taxID used:135614 OG01118|WP\_004088630.1 | bis(5'-nucleosyl)-tetraphosphatase (symmetrical) | taxID used:135614 OG01119|WP\_004086003.1 | segregation/condensation protein A | taxID used:135614 OG01123|WP\_046419711.1 | hydroxymethylbilane synthase | taxID used:135614 OG01126|WP\_011098000.1 | ribosomal RNA small subunit methyltransferase A | taxID used:135614 OG01127|WP\_004084369.1 | cation transporter | taxID used:135614 OG01128|WP\_020851593.1 | outer membrane protein assembly factor BamD | taxID used:135614 OG01133|WP\_071869898.1 | TIGR01777 family protein | taxID used:135614 OG01134|WP\_023907570.1 | glycosyltransferase family 2 protein | taxID used:135614 OG01135|WP\_046418189.1 | nitrilase/cyanide hydratase | taxID used:135614 OG01136|WP\_057683465.1 | glycosyltransferase family 2 protein | taxID used:135614 OG01137|WP\_020852274.1 | ligand-binding protein SH3 | taxID used:135614 OG01138|WP\_038229140.1 | YicC family protein | taxID used:135614 OG01140|WP\_021358338.1 | energy transducer TonB | taxID used:135614 OG01145|WP\_010894695.1 | GTPase Era | taxID used:135614 OG01147|WP\_071869637.1 | 4-hydroxy-tetrahydrodipicolinate synthase | taxID used:135614 OG00114|WP\_046419574.1 | DUF1631 domain-containing protein | taxID used:135614 OG01152|WP\_023906245.1 | sugar ABC transporter permease | taxID used:135614 OG01158|WP\_023907818.1 | phosphatidylserine decarboxylase | taxID used:135614 OG01160|WP\_004085665.1 | enoyl-CoA hydratase | taxID used:135614 OG01161|WP\_004087397.1 | 4-(cytidine 5'-diphospho)-2-C-methyl-D-erythritol kinase | taxID used:135614 OG01162|WP\_004083728.1 | polysaccharide deacetylase | taxID used:135614 OG01165|WP\_024749074.1 | HlyC/CorC family transporter | taxID used:135614 OG01166|WP\_004085962.1 | UTP--glucose-1-phosphate uridylyltransferase GalU | taxID used:135614 OG01167|WP\_012337957.1 | D-hexose-6-phosphate mutarotase | taxID used:135614 OG01168|WP\_010892638.1 | dihydropteroate synthase | taxID used:135614 OG01170|WP\_058569630.1 | M23 family peptidase | taxID used:135614 OG01175|WP\_027700580.1 | iron-sulfur cluster carrier protein ApbC | taxID used:135614 OG01179|WP\_038229813.1 | RNase adapter RapZ | taxID used:135614 OG00117|WP\_004088308.1 | bifunctional aspartate kinase/homoserine dehydrogenase I | taxID used:135614 OG01185|WP\_004084990.1 | ABC transporter ATP-binding protein | taxID used:135614 OG01190|WP\_046418125.1 | NlpC/P60 family protein | taxID used:135614 OG01191|WP\_046418909.1 | acyl-CoA thioesterase II | taxID used:135614 OG01192|WP\_010893661.1 | F0F1 ATP synthase subunit gamma | taxID used:135614 OG01199|WP\_027700202.1 | YihY/virulence factor BrkB family protein | taxID used:135614 OG01201|WP\_057683202.1 | formyltetrahydrofolate deformylase | taxID used:135614 OG01203|WP\_088577737.1 | geranyl transferase | taxID used:135614 OG01205|WP\_046418458.1 | succinate--CoA ligase subunit alpha | taxID used:135614 OG01207|WP\_104993199.1 | RNA polymerase sigma factor RpoH | taxID used:135614 OG01208|WP\_031336365.1 | bifunctional methylenetetrahydrofolate dehydrogenase/methenyltetrahydrofolate cyclohydrolase FolD | taxID used:135614 OG01209|WP\_004084594.1 | prepilin peptidase | taxID used:135614 OG01210|WP\_046418088.1 | DUF3108 domain-containing protein | taxID used:135614 OG01211|WP\_023908120.1 | elongation factor Ts | taxID used:135614 OG01217|WP\_004084048.1 | AraC family transcriptional regulator | taxID used:135614 OG01219|WP\_046419544.1 | diaminopimelate epimerase | taxID used:135614 OG01224|WP\_031337079.1 | SAM-dependent methyltransferase | taxID used:135614 OG01227|WP\_031336669.1 | hypothetical protein | taxID used:135614 OG01229|WP\_010895042.1 | protease HtpX | taxID used:135614 OG01230|WP\_004089788.1 | protease modulator HflC | taxID used:135614 OG01231|WP\_004086094.1 | phosphate ABC transporter permease | taxID used:135614 OG01234|WP\_023907952.1 | M23 family metallopeptidase | taxID used:135614 OG01236|WP\_004086273.1 | pantoate--beta-alanine ligase | taxID used:135614 OG01238|WP\_031336480.1 | co-chaperone YbbN | taxID used:135614 OG01240|WP\_010893849.1 | sulfate ABC transporter permease subunit CysT | taxID used:135614 OG01242|WP\_038211122.1 | pyrroline-5-carboxylate reductase | taxID used:135614 OG01243|WP\_004083708.1 | sirC regulator SirB | taxID used:135614 OG01244|WP\_046418940.1 | phosphatidate cytidylyltransferase | taxID used:135614 OG01247|WP\_021358442.1 | glycosyltransferase family 2 protein | taxID used:135614 OG01249|WP\_046418119.1 | shikimate dehydrogenase | taxID used:135614 OG01255|WP\_088577995.1 | hydrolase TatD | taxID used:135614 OG01256|WP\_004088277.1 | aquaporin family protein | taxID used:135614 OG00125|WP\_046417588.1 | S9 family peptidase | taxID used:135614 OG01261|WP\_038230072.1 | phosphate ABC transporter ATP-binding protein PstB | taxID used:135614 OG01265|WP\_004084787.1 | response regulator | taxID used:135614 OG01268|WP\_004086033.1 | inositol monophosphatase | taxID used:135614 OG00126|WP\_058569803.1 | DNA topoisomerase (ATP-hydrolyzing) subunit B | taxID used:135614 OG01272|WP\_010894044.1 | tRNA threonylcarbamoyladenosine dehydratase | taxID used:135614 OG01277|WP\_004085054.1 | bifunctional DNA-formamidopyrimidine glycosylase/DNA-(apurinic or apyrimidinic site) lyase | taxID used:135614 OG01278|WP\_027699983.1 | Fe-S cluster assembly ATPase SufC | taxID used:135614 OG01279|WP\_046420112.1 | tol-pal system protein YbgF | taxID used:135614 OG01281|WP\_004084011.1 | 2-dehydro-3-deoxyphosphooctonate aldolase | taxID used:135614 OG01282|WP\_010892730.1 | 3'(2'),5'-bisphosphate nucleotidase | taxID used:135614 OG01283|WP\_004084043.1 | phosphoenolpyruvate synthetase regulatory protein | taxID used:135614 OG01284|WP\_010893560.1 | ribonuclease HII | taxID used:135614 OG01286|WP\_038200741.1 | ABC transporter ATP-binding protein | taxID used:135614 OG01287|WP\_058569805.1 | M48 family peptidase | taxID used:135614 OG01290|WP\_023907074.1 | ABC transporter permease | taxID used:135614 OG01291|WP\_046419578.1 | peptide chain release factor N(5)-glutamine methyltransferase | taxID used:135614 OG01293|WP\_020851812.1 | SDR family NAD(P)-dependent oxidoreductase | taxID used:135614 OG01294|WP\_109160974.1 | M48 family peptidase | taxID used:135614 OG01297|WP\_088578311.1 | succinate dehydrogenase iron-sulfur subunit | taxID used:135614 OG01301|WP\_020851271.1 | thymidylate synthase | taxID used:135614 OG01303|WP\_020852605.1 | 3-methyl-2-oxobutanoate hydroxymethyltransferase | taxID used:135614 OG01308|WP\_023906157.1 | type IV pilus biogenesis/stability protein PilW | taxID used:135614 OG01309|WP\_004083553.1 | 16S rRNA (cytidine(1402)-2'-O)-methyltransferase | taxID used:135614 OG01310|WP\_004088701.1 | DUF4198 domain-containing protein | taxID used:135614 OG01311|WP\_020853073.1 | bifunctional hydroxymethylpyrimidine kinase/phosphomethylpyrimidine kinase | taxID used:135614 OG01312|WP\_038229782.1 | pilus assembly protein PapD | taxID used:135614 OG01314|WP\_004083405.1 | 1-(5-phosphoribosyl)-5-[(5-phosphoribosylamino)methylideneamino]imidazole-4-carboxamide isomerase | taxID used:135614 OG01315|WP\_004087867.1 | S-adenosylmethionine decarboxylase proenzyme | taxID used:135614 OG01323|WP\_004090321.1 | 30S ribosomal protein S2 | taxID used:135614 OG01324|WP\_004089294.1 | folate-binding protein | taxID used:135614 OG01325|WP\_080939607.1 | exodeoxyribonuclease III | taxID used:135614 OG01326|WP\_011097591.1 | acyl-[acyl-carrier-protein]--UDP-N-acetylglucosamine O-acyltransferase | taxID used:135614 OG01330|WP\_010893378.1 | 5'-nucleotidase SurE | taxID used:135614 OG01332|WP\_004083895.1 | septum site-determining protein MinD | taxID used:135614 OG01334|WP\_014607604.1 | arginyltransferase | taxID used:135614 OG01336|WP\_023907783.1 | thiol:disulfide interchange protein DsbA/DsbL | taxID used:135614 OG01337|WP\_021358311.1 | tRNA (guanosine(37)-N1)-methyltransferase TrmD | taxID used:135614 OG01338|WP\_038232779.1 | type I methionyl aminopeptidase | taxID used:135614 OG01339|WP\_004090667.1 | tryptophan synthase subunit alpha | taxID used:135614 OG01347|WP\_024749251.1 | tRNA pseudouridine(38-40) synthase TruA | taxID used:135614 OG01348|WP\_031336649.1 | YggS family pyridoxal phosphate-dependent enzyme | taxID used:135614 OG01349|WP\_020852765.1 | ferredoxin--NADP reductase | taxID used:135614 OG00134|WP\_088578610.1 | phosphoenolpyruvate synthase | taxID used:135614 OG01351|WP\_031337052.1 | methionine ABC transporter substrate-binding protein | taxID used:135614 OG01353|WP\_024748784.1 | RNA methyltransferase | taxID used:135614 OG01356|WP\_004572975.1 | indole-3-glycerol phosphate synthase TrpC | taxID used:135614 OG01358|WP\_058564904.1 | pyridoxine 5'-phosphate synthase | taxID used:135614 OG01359|WP\_004090471.1 | thiazole synthase | taxID used:135614 OG01362|WP\_004089155.1 | endonuclease/exonuclease/phosphatase family protein | taxID used:135614 OG01363|WP\_058564322.1 | sulfurtransferase | taxID used:135614 OG01364|WP\_010892721.1 | outer membrane protein | taxID used:135614 OG01365|WP\_004085817.1 | glycosyltransferase | taxID used:135614 OG01367|WP\_004088266.1 | ParA family protein | taxID used:135614 OG01368|WP\_010893569.1 | ditrans,polycis-undecaprenyl-diphosphate synthase ((2E,6E)-farnesyl-diphosphate specific) | taxID used:135614 OG01369|WP\_023906627.1 | hypothetical protein | taxID used:135614 OG00136|WP\_023906998.1 | phenylalanine--tRNA ligase subunit beta | taxID used:135614 OG01372|WP\_038228299.1 | epoxyqueuosine reductase QueH | taxID used:135614 OG01375|WP\_088572644.1 | short-chain dehydrogenase/reductase SDR | taxID used:135614 OG01377|WP\_004083404.1 | imidazole glycerol phosphate synthase cyclase subunit | taxID used:135614 OG01380|WP\_057682898.1 | 3-deoxy-manno-octulosonate cytidylyltransferase | taxID used:135614 OG01383|WP\_004086464.1 | cytochrome c1 | taxID used:135614 OG01385|WP\_004083589.1 | CDP-diacylglycerol--serine O-phosphatidyltransferase | taxID used:135614 OG01389|WP\_010895106.1 | uracil-DNA glycosylase | taxID used:135614 OG00138|WP\_038229842.1 | DNA translocase FtsK | taxID used:135614 OG01394|WP\_023907825.1 | pimeloyl-[acyl-carrier protein] methyl ester esterase | taxID used:135614 OG01395|WP\_020851909.1 | hypothetical protein | taxID used:135614 OG01399|WP\_010893988.1 | bifunctional demethylmenaquinone methyltransferase/2-methoxy-6-polyprenyl-1,4-benzoquinol methylase UbiE | taxID used:135614 OG01403|WP\_004088479.1 | heme ABC transporter permease | taxID used:135614 OG01405|WP\_004084957.1 | DNA-binding response regulator | taxID used:135614 OG01407|WP\_004087841.1 | MotA/TolQ/ExbB proton channel family protein | taxID used:135614 OG01410|WP\_004085414.1 | 2,3-bisphosphoglycerate-dependent phosphoglycerate mutase | taxID used:135614 OG01412|WP\_058564316.1 | UDP-2,3-diacylglucosamine diphosphatase | taxID used:135614 OG01415|WP\_004085434.1 | superoxide dismutase | taxID used:135614 OG01416|WP\_012382616.1 | monofunctional biosynthetic peptidoglycan transglycosylase | taxID used:135614 OG00141|WP\_038211324.1 | membrane protein | taxID used:135614 OG01421|WP\_058564233.1 | leucyl/phenylalanyl-tRNA--protein transferase | taxID used:135614 OG01423|WP\_004090474.1 | tRNA (guanine-N(7)-)-methyltransferase | taxID used:135614 OG01426|WP\_010892612.1 | amidophosphoribosyltransferase | taxID used:135614 OG00142|WP\_031336322.1 | TonB-dependent siderophore receptor | taxID used:135614 OG01431|WP\_004086023.1 | bifunctional 3-demethylubiquinone 3-O-methyltransferase/2-octaprenyl-6-hydroxy phenol methylase | taxID used:135614 OG01434|WP\_012338095.1 | ketosynthase | taxID used:135614 OG01436|WP\_027700541.1 | orotidine-5'-phosphate decarboxylase | taxID used:135614 OG00143|WP\_058564857.1 | RNA-binding transcriptional accessory protein | taxID used:135614 OG01441|WP\_004087940.1 | triose-phosphate isomerase | taxID used:135614 OG01443|WP\_011098260.1 | polysaccharide deacetylase family protein | taxID used:135614 OG01444|WP\_004085871.1 | ABC transporter ATP-binding protein | taxID used:135614 OG01446|WP\_057683156.1 | glutamine amidotransferase | taxID used:135614 OG01447|WP\_046418442.1 | cell division protein ZipA | taxID used:135614 OG01448|WP\_057682448.1 | LysM peptidoglycan-binding domain-containing protein | taxID used:135614 OG01451|WP\_004084642.1 | ABC transporter ATP-binding protein | taxID used:135614 OG01452|WP\_038228024.1 | twin-arginine translocase subunit TatC | taxID used:135614 OG01454|WP\_020852327.1 | haloacid dehalogenase | taxID used:135614 OG01456|WP\_004091141.1 | 23S rRNA (guanosine(2251)-2'-O)-methyltransferase RlmB | taxID used:135614 OG01457|WP\_004083738.1 | short-chain dehydrogenase/reductase SDR | taxID used:135614 OG01458|WP\_023906699.1 | phosphoadenosine phosphosulfate reductase | taxID used:135614 OG01459|WP\_010894372.1 | YebC/PmpR family DNA-binding transcriptional regulator | taxID used:135614 OG00145|WP\_038232863.1 | type II secretion system protein GspD | taxID used:135614 OG01463|WP\_004572909.1 | deoxyribonuclease V | taxID used:135614 OG01467|WP\_057683340.1 | 3-oxoacyl-ACP reductase FabG | taxID used:135614 OG01468|WP\_004083606.1 | ABC transporter permease | taxID used:135614 OG00146|WP\_012337608.1 | NADP-dependent malic enzyme | taxID used:135614 OG01471|WP\_010892733.1 | 16S rRNA (uracil(1498)-N(3))-methyltransferase | taxID used:135614 OG01472|WP\_010894605.1 | serine/threonine-protein phosphatase | taxID used:135614 OG01473|WP\_027700097.1 | DNA repair protein RecO | taxID used:135614 OG01474|WP\_010893116.1 | nucleotidyltransferase family protein | taxID used:135614 OG01475|WP\_004088269.1 | dolichol-phosphate mannosyltransferase | taxID used:135614 OG01476|WP\_038230136.1 | 6-phosphogluconolactonase | taxID used:135614 OG01480|WP\_004085659.1 | SIMPL domain-containing protein | taxID used:135614 OG01482|WP\_004088636.1 | phosphate-specific transport system accessory protein PhoU | taxID used:135614 OG01491|WP\_031336234.1 | cell envelope biogenesis protein OmpA | taxID used:135614 OG01493|WP\_038229647.1 | type III pantothenate kinase | taxID used:135614 OG01494|WP\_010893911.1 | LPS export ABC transporter ATP-binding protein | taxID used:135614 OG01495|WP\_010894975.1 | DUF3011 domain-containing protein | taxID used:135614 OG01496|WP\_010894606.1 | DNA polymerase III subunit epsilon | taxID used:135614 OG01499|WP\_023907913.1 | lipoprotein-releasing system ATP-binding protein LolD | taxID used:135614 OG01500|WP\_046419348.1 | septum site-determining protein MinC | taxID used:135614 OG01501|WP\_020851394.1 | polyisoprenoid-binding protein | taxID used:135614 OG01506|WP\_058564891.1 | DUF2461 domain-containing protein | taxID used:135614 OG01508|WP\_010892740.1 | DUF502 domain-containing protein | taxID used:135614 OG01509|WP\_020851329.1 | acireductone synthase | taxID used:135614 OG00150|WP\_038228064.1 | cytochrome c biogenesis protein | taxID used:135614 OG01511|WP\_010893622.1 | 4-hydroxy-tetrahydrodipicolinate reductase | taxID used:135614 OG01519|WP\_004086562.1 | 16S rRNA pseudouridine(516) synthase | taxID used:135614 OG01527|WP\_004088673.1 | peptidylprolyl isomerase | taxID used:135614 OG01529|WP\_004084660.1 | phosphate regulon transcriptional regulatory protein PhoB | taxID used:135614 OG00152|WP\_004089140.1 | ATP-dependent Clp protease ATP-binding subunit ClpA | taxID used:135614 OG01530|WP\_058569536.1 | phytoene/squalene synthase family protein | taxID used:135614 OG01533|WP\_058569681.1 | lipoyl(octanoyl) transferase LipB | taxID used:135614 OG01538|WP\_023906769.1 | DNA-3-methyladenine glycosylase 2 family protein | taxID used:135614 OG01539|WP\_057683331.1 | phosphoglycolate phosphatase | taxID used:135614 OG01547|WP\_004085576.1 | 7-cyano-7-deazaguanine synthase QueC | taxID used:135614 OG01548|WP\_046419709.1 | alpha/beta hydrolase | taxID used:135614 OG00154|WP\_046420864.1 | NADP-dependent isocitrate dehydrogenase | taxID used:135614 OG01550|WP\_020851534.1 | 2-C-methyl-D-erythritol 4-phosphate cytidylyltransferase | taxID used:135614 OG01551|WP\_038229417.1 | DNA-binding response regulator | taxID used:135614 OG01553|WP\_004083529.1 | DnaA regulatory inactivator Hda | taxID used:135614 OG01554|WP\_060872255.1 | hypothetical protein | taxID used:135614 OG01555|WP\_038210738.1 | DUF3108 domain-containing protein | taxID used:135614 OG01556|WP\_058564800.1 | endonuclease III | taxID used:135614 OG01558|WP\_010895052.1 | 50S ribosomal protein L1 | taxID used:135614 OG01559|WP\_058569836.1 | cAMP-activated global transcriptional regulator CRP | taxID used:135614 OG01563|WP\_058569547.1 | dethiobiotin synthase | taxID used:135614 OG01564|WP\_010894034.1 | tRNA (adenosine(37)-N6)-threonylcarbamoyltransferase complex dimerization subunit type 1 TsaB | taxID used:135614 OG01566|WP\_023906310.1 | ribonuclease 3 | taxID used:135614 OG01567|WP\_020852451.1 | HAD family phosphatase | taxID used:135614 OG01569|WP\_004088218.1 | carbonate dehydratase | taxID used:135614 OG00156|WP\_126715037.1 | primosomal protein N' | taxID used:135614 OG01572|WP\_004087507.1 | cell division ATP-binding protein FtsE | taxID used:135614 OG01575|WP\_020851467.1 | rhomboid family intramembrane serine protease | taxID used:135614 OG01576|WP\_046417796.1 | ribulose-phosphate 3-epimerase | taxID used:135614 OG01577|WP\_004085207.1 | rhomboid family intramembrane serine protease | taxID used:135614 OG01578|WP\_004083627.1 | response regulator transcription factor | taxID used:135614 OG01579|WP\_004087991.1 | serine/threonine protein kinase | taxID used:135614 OG01585|WP\_023906947.1 | protein-L-isoaspartate O-methyltransferase | taxID used:135614 OG01587|WP\_004085446.1 | peptide-methionine (S)-S-oxide reductase MsrA | taxID used:135614 OG01588|WP\_057682944.1 | methylthioribulose 1-phosphate dehydratase | taxID used:135614 OG01590|WP\_004085415.1 | 7-carboxy-7-deazaguanine synthase QueE | taxID used:135614 OG01591|WP\_023907432.1 | cytidylate kinase | taxID used:135614 OG01592|WP\_010894955.1 | DNA-binding response regulator | taxID used:135614 OG01594|WP\_004083718.1 | DUF484 domain-containing protein | taxID used:135614 OG01596|WP\_081089885.1 | heme ABC exporter ATP-binding protein CcmA | taxID used:135614 OG01599|WP\_046419411.1 | N-(5'-phosphoribosyl)anthranilate isomerase | taxID used:135614 OG01602|WP\_080507225.1 | NADH-quinone oxidoreductase subunit J | taxID used:135614 OG01606|WP\_027700531.1 | hypothetical protein | taxID used:135614 OG01608|WP\_027700549.1 | 2-nonaprenyl-3-methyl-6-methoxy-1,4-benzoquinol hydroxylase | taxID used:135614 OG01609|WP\_060870113.1 | DUF2076 domain-containing protein | taxID used:135614 OG01613|WP\_010893111.1 | phosphoribosylglycinamide formyltransferase | taxID used:135614 OG01615|WP\_020851828.1 | phospholipid-binding protein MlaC | taxID used:135614 OG01617|WP\_010892698.1 | orotate phosphoribosyltransferase | taxID used:135614 OG01620|WP\_020851296.1 | 3-isopropylmalate dehydratase small subunit | taxID used:135614 OG01625|WP\_004085189.1 | MotA/TolQ/ExbB proton channel family protein | taxID used:135614 OG01626|WP\_004085033.1 | phospholipid/glycerol acyltransferase | taxID used:135614 OG01627|WP\_038230133.1 | ketohydroxyglutarate aldolase | taxID used:135614 OG01629|WP\_027699991.1 | outer membrane lipoprotein chaperone LolA | taxID used:135614 OG01630|WP\_004091006.1 | polysaccharide biosynthesis protein GumB | taxID used:135614 OG01631|WP\_010893937.1 | thiol:disulfide interchange protein DsbA/DsbL | taxID used:135614 OG01632|WP\_071869641.1 | RlmE family RNA methyltransferase | taxID used:135614 OG01633|WP\_004091185.1 | ribose-5-phosphate isomerase RpiA | taxID used:135614 OG01635|WP\_004083987.1 | ABC transporter | taxID used:135614 OG01638|WP\_031336908.1 | 50S ribosomal protein L3 | taxID used:135614 OG01639|WP\_004087541.1 | DNA-binding response regulator | taxID used:135614 OG01642|WP\_031336485.1 | ribonuclease T | taxID used:135614 OG01644|WP\_004085049.1 | hemolysin III family protein | taxID used:135614 OG01647|WP\_010893601.1 | ParA family protein | taxID used:135614 OG01648|WP\_010894757.1 | CDP-diacylglycerol--glycerol-3-phosphate 3-phosphatidyltransferase | taxID used:135614 OG01649|WP\_004085407.1 | histidine phosphatase family protein | taxID used:135614 OG01652|WP\_080507193.1 | fatty acyl CoA synthetase | taxID used:135614 OG01660|WP\_010894298.1 | SCP2 domain-containing protein | taxID used:135614 OG01672|WP\_004083425.1 | RNA polymerase sigma factor RpoE | taxID used:135614 OG01673|WP\_004087331.1 | DUF2058 domain-containing protein | taxID used:135614 OG01677|WP\_080507190.1 | peptidoglycan endopeptidase | taxID used:135614 OG01682|WP\_020852082.1 | transcriptional repressor LexA | taxID used:135614 OG01683|WP\_057683487.1 | YbhB/YbcL family Raf kinase inhibitor-like protein | taxID used:135614 OG01685|WP\_004083680.1 | general secretion pathway protein GspM | taxID used:135614 OG01686|WP\_004572865.1 | ribosomal RNA small subunit methyltransferase G | taxID used:135614 OG01687|WP\_011097763.1 | general secretory pathway protein GspJ | taxID used:135614 OG01688|WP\_010894263.1 | SPOR domain-containing protein | taxID used:135614 OG01690|WP\_004087911.1 | 16S rRNA (guanine(966)-N(2))-methyltransferase RsmD | taxID used:135614 OG01691|WP\_020852792.1 | HNH endonuclease | taxID used:135614 OG01692|WP\_010893491.1 | response regulator transcription factor | taxID used:135614 OG01699|WP\_020851188.1 | membrane protein | taxID used:135614 OG01701|WP\_031336534.1 | GTP cyclohydrolase I FolE | taxID used:135614 OG01708|WP\_010895031.1 | superoxide dismutase | taxID used:135614 OG00170|WP\_046417956.1 | guanosine-3',5'-bis(diphosphate) 3'-diphosphatase | taxID used:135614 OG01712|WP\_004083711.1 | thioredoxin family protein | taxID used:135614 OG01716|WP\_011098259.1 | D-alanyl-D-alanine carboxypeptidase family protein | taxID used:135614 OG01720|WP\_004085604.1 | hypothetical protein | taxID used:135614 OG01721|WP\_023906948.1 | DedA family protein | taxID used:135614 OG01722|WP\_031337900.1 | YihA family ribosome biogenesis GTP-binding protein | taxID used:135614 OG01723|WP\_004089235.1 | cytochrome o ubiquinol oxidase subunit III | taxID used:135614 OG01725|WP\_021358213.1 | 2-amino-4-hydroxy-6-hydroxymethyldihydropteridine diphosphokinase | taxID used:135614 OG01728|WP\_020851236.1 | dephospho-CoA kinase | taxID used:135614 OG01729|WP\_004088320.1 | bifunctional phosphoribosyl-AMP cyclohydrolase/phosphoribosyl-ATP diphosphatase HisIE | taxID used:135614 OG01733|WP\_088577668.1 | thiamine phosphate synthase | taxID used:135614 OG01738|WP\_010892742.1 | class I SAM-dependent methyltransferase | taxID used:135614 OG01740|WP\_052151232.1 | hypothetical protein | taxID used:135614 OG01742|WP\_031336824.1 | pyridoxamine 5'-phosphate oxidase | taxID used:135614 OG01747|WP\_021358333.1 | 4-phosphopantetheinyl transferase | taxID used:135614 OG00174|WP\_027699982.1 | S9 family peptidase | taxID used:135614 OG01751|WP\_071869539.1 | 50S ribosomal protein L4 | taxID used:135614 OG01753|WP\_010893940.1 | lysogenization protein HflD | taxID used:135614 OG01754|WP\_057682614.1 | 5-formyltetrahydrofolate cyclo-ligase | taxID used:135614 OG01755|WP\_004086014.1 | thiol:disulfide interchange protein DsbE | taxID used:135614 OG01756|WP\_011097758.1 | non-canonical purine NTP pyrophosphatase, RdgB/HAM1 family | taxID used:135614 OG01758|WP\_004084669.1 | Fe-S biogenesis protein NfuA | taxID used:135614 OG00175|WP\_046420029.1 | peptidase S41 | taxID used:135614 OG01763|WP\_023906328.1 | imidazole glycerol phosphate synthase subunit HisH | taxID used:135614 OG01766|WP\_046417983.1 | RNA pyrophosphohydrolase | taxID used:135614 OG01769|WP\_020852363.1 | oligoribonuclease | taxID used:135614 OG00176|WP\_004087928.1 | NADH-quinone oxidoreductase subunit L | taxID used:135614 OG01771|WP\_046418994.1 | NAD(P)H:quinone oxidoreductase | taxID used:135614 OG01778|WP\_004089677.1 | riboflavin synthase | taxID used:135614 OG00177|WP\_023906772.1 | bifunctional (p)ppGpp synthetase/guanosine-3',5'-bis(diphosphate) 3'-pyrophosphohydrolase | taxID used:135614 OG01781|WP\_046420827.1 | YhgN family NAAT transporter | taxID used:135614 OG01783|WP\_023906782.1 | alpha-ketoglutarate-dependent dioxygenase AlkB | taxID used:135614 OG01784|WP\_010893285.1 | dCTP deaminase | taxID used:135614 OG01785|WP\_020851300.1 | hypothetical protein | taxID used:135614 OG01786|WP\_023906077.1 | aminodeoxychorismate/anthranilate synthase component II | taxID used:135614 OG00178|WP\_010894353.1 | S46 family peptidase | taxID used:135614 OG01790|WP\_004083893.1 | GNAT family N-acetyltransferase | taxID used:135614 OG01796|WP\_004084727.1 | N-acetylmuramoyl-L-alanine amidase | taxID used:135614 OG01800|WP\_004091174.1 | nitroreductase | taxID used:135614 OG01802|WP\_010893603.1 | polyisoprenoid-binding protein | taxID used:135614 OG01804|WP\_023906333.1 | acireductone dioxygenase | taxID used:135614 OG01808|WP\_020851002.1 | DUF1439 domain-containing protein | taxID used:135614 OG01811|WP\_010893913.1 | LPS export ABC transporter periplasmic protein LptC | taxID used:135614 OG01814|WP\_031336436.1 | manganese efflux pump MntP family protein | taxID used:135614 OG01816|WP\_020852749.1 | DUF3106 domain-containing protein | taxID used:135614 OG01817|WP\_031337124.1 | DUF4019 domain-containing protein | taxID used:135614 OG01819|WP\_038211556.1 | glycine cleavage system regulatory protein | taxID used:135614 OG01824|WP\_027700064.1 | flavodoxin family protein | taxID used:135614 OG01825|WP\_046420118.1 | Holliday junction branch migration protein RuvA | taxID used:135614 OG01826|WP\_004090608.1 | cytochrome b | taxID used:135614 OG01827|WP\_046419046.1 | chorismate mutase AroQ, gamma subclass | taxID used:135614 OG01828|WP\_004083888.1 | polyisoprenoid-binding protein | taxID used:135614 OG00182|WP\_004572844.1 | glycine--tRNA ligase subunit beta | taxID used:135614 OG01831|WP\_081046815.1 | RDD family protein | taxID used:135614 OG01833|WP\_012338001.1 | elongation factor P | taxID used:135614 OG01834|WP\_010893403.1 | hypothetical protein | taxID used:135614 OG01835|WP\_004085654.1 | DUF615 family protein | taxID used:135614 OG01837|WP\_004085302.1 | NADH-quinone oxidoreductase subunit B | taxID used:135614 OG01838|WP\_058569279.1 | DNA-binding response regulator | taxID used:135614 OG00183|WP\_004088742.1 | peptidase | taxID used:135614 OG01840|WP\_020851452.1 | dTDP-4-dehydrorhamnose 3,5-epimerase | taxID used:135614 OG01841|WP\_010894676.1 | YqgE/AlgH family protein | taxID used:135614 OG01842|WP\_012337808.1 | gamma carbonic anhydrase family protein | taxID used:135614 OG01850|WP\_020852892.1 | peroxiredoxin | taxID used:135614 OG01852|WP\_004087851.1 | prepilin-type N-terminal cleavage/methylation domain-containing protein | taxID used:135614 OG01857|WP\_088577970.1 | hypothetical protein | taxID used:135614 OG00185|WP\_046420800.1 | elongation factor G | taxID used:135614 OG01863|WP\_046419079.1 | DUF1415 domain-containing protein | taxID used:135614 OG01873|WP\_021358237.1 | Maf-like protein | taxID used:135614 OG01875|WP\_027700732.1 | hypothetical protein | taxID used:135614 OG01876|WP\_010894457.1 | putative Fe-S cluster assembly protein SufT | taxID used:135614 OG01889|WP\_012337802.1 | NUDIX hydrolase | taxID used:135614 OG01890|WP\_046417645.1 | type 1 fimbrial protein | taxID used:135614 OG01892|WP\_024748852.1 | SMR domain protein | taxID used:135614 OG01895|WP\_004085245.1 | hypothetical protein | taxID used:135614 OG01899|WP\_004090677.1 | DNA starvation/stationary phase protection protein | taxID used:135614 OG00018|WP\_011098326.1 | DNA-directed RNA polymerase subunit beta' | taxID used:135614 OG01900|WP\_004085328.1 | adenylate kinase | taxID used:135614 OG01904|WP\_004085039.1 | ADP compounds hydrolase NudE | taxID used:135614 OG01906|WP\_004089320.1 | ribosome recycling factor | taxID used:135614 OG01909|WP\_004083880.1 | shikimate kinase | taxID used:135614 OG01910|WP\_046418751.1 | transcriptional repressor NrdR | taxID used:135614 OG01916|WP\_004089213.1 | HAD family hydrolase | taxID used:135614 OG01918|WP\_004088597.1 | inorganic pyrophosphatase | taxID used:135614 OG01919|WP\_010893678.1 | 50S ribosomal protein L5 | taxID used:135614 OG01923|WP\_031336568.1 | peptidoglycan-associated lipoprotein Pal | taxID used:135614 OG01927|WP\_100206159.1 | membrane protein | taxID used:135614 OG00192|WP\_004087521.1 | polyphosphate kinase 1 | taxID used:135614 OG01930|WP\_010893985.1 | ATP-dependent protease subunit HslV | taxID used:135614 OG01931|WP\_004083607.1 | outer membrane lipid asymmetry maintenance protein MlaD | taxID used:135614 OG01936|WP\_004086539.1 | 30S ribosomal protein S5 | taxID used:135614 OG01939|WP\_004087934.1 | NADH-quinone oxidoreductase subunit NuoE | taxID used:135614 OG00193|WP\_057682651.1 | transketolase | taxID used:135614 OG01941|WP\_075584689.1 | translation initiation factor IF-3 | taxID used:135614 OG01946|WP\_004089217.1 | lipopolysaccharide transport periplasmic protein LptA | taxID used:135614 OG01949|WP\_004085458.1 | purine-binding chemotaxis protein CheW | taxID used:135614 OG01951|WP\_010892873.1 | disulfide bond formation protein B | taxID used:135614 OG01952|WP\_004088700.1 | DUF2271 domain-containing protein | taxID used:135614 OG01953|WP\_031345912.1 | methylated-DNA--[protein]-cysteine S-methyltransferase | taxID used:135614 OG00195|WP\_046418373.1 | EAL domain-containing protein | taxID used:135614 OG01960|WP\_004088753.1 | 3-hydroxyacyl-[acyl-carrier-protein] dehydratase FabA | taxID used:135614 OG01966|WP\_004084453.1 | transcriptional repressor | taxID used:135614 OG01972|WP\_004085641.1 | F0F1 ATP synthase subunit delta | taxID used:135614 OG01974|WP\_027700246.1 | alpha/beta hydrolase | taxID used:135614 OG01975|WP\_010893681.1 | 50S ribosomal protein L6 | taxID used:135614 OG01980|WP\_010892654.1 | ribosome maturation factor RimM | taxID used:135614 OG01981|WP\_004084893.1 | protein-export protein SecB | taxID used:135614 OG01982|WP\_004089770.1 | tfp pilus assembly protein fimT | taxID used:135614 OG01988|WP\_031336846.1 | poly(hydroxyalcanoate) granule associated protein | taxID used:135614 OG00198|WP\_057683072.1 | phosphoesterase | taxID used:135614 OG01991|WP\_004089639.1 | peptide deformylase | taxID used:135614 OG01993|WP\_004084691.1 | 50S ribosomal protein L10 | taxID used:135614 OG01996|WP\_023907227.1 | 2-amino-4-hydroxy-6-hydroxymethyldihydropteridine diphosphokinase | taxID used:135614 OG01997|WP\_020852367.1 | DUF1249 domain-containing protein | taxID used:135614 OG01998|WP\_004085935.1 | lipoprotein signal peptidase | taxID used:135614 OG01999|WP\_004085850.1 | nucleotide exchange factor GrpE | taxID used:135614 OG00199|WP\_060872247.1 | polyribonucleotide nucleotidyltransferase | taxID used:135614 OG02002|WP\_004085836.1 | SsrA-binding protein SmpB | taxID used:135614 OG02003|WP\_031336828.1 | hypothetical protein | taxID used:135614 OG02005|WP\_010892757.1 | MarR family transcriptional regulator | taxID used:135614 OG00200|WP\_058569712.1 | tetratricopeptide repeat protein | taxID used:135614 OG02011|WP\_031337936.1 | 2-C-methyl-D-erythritol 2,4-cyclodiphosphate synthase | taxID used:135614 OG02017|WP\_023906584.1 | DUF177 domain-containing protein | taxID used:135614 OG02022|WP\_004083791.1 | Single-stranded DNA-binding protein 2 | taxID used:135614 OG02024|WP\_004083685.1 | prepilin-type N-terminal cleavage/methylation domain-containing protein | taxID used:135614 OG02027|WP\_011097819.1 | SgcJ/EcaC family oxidoreductase | taxID used:135614 OG02029|WP\_046420774.1 | dihydrofolate reductase | taxID used:135614 OG02031|WP\_004088821.1 | Rnf electron transport complex subunit RnfB | taxID used:135614 OG02033|WP\_004086570.1 | peptidylprolyl isomerase | taxID used:135614 OG02035|WP\_020851178.1 | fimbrial assembly protein | taxID used:135614 OG02036|WP\_004085823.1 | CYTH domain-containing protein | taxID used:135614 OG02038|WP\_004084756.1 | 5-(carboxyamino)imidazole ribonucleotide mutase | taxID used:135614 OG02047|WP\_027700520.1 | hypothetical protein | taxID used:135614 OG02048|WP\_004085295.1 | NADH-quinone oxidoreductase subunit NuoI | taxID used:135614 OG02049|WP\_023907910.1 | L-asparaginase | taxID used:135614 OG02053|WP\_027700581.1 | low molecular weight phosphotyrosine protein phosphatase | taxID used:135614 OG02062|WP\_031337990.1 | hypothetical protein | taxID used:135614 OG02064|WP\_010893563.1 | 3-hydroxyacyl-ACP dehydratase FabZ | taxID used:135614 OG02069|WP\_004086560.1 | flavodoxin | taxID used:135614 OG02074|WP\_004083567.1 | type IV pilus modification protein PilV | taxID used:135614 OG02075|WP\_004083422.1 | Hsp20/alpha crystallin family protein | taxID used:135614 OG02076|WP\_010893991.1 | MarR family transcriptional regulator | taxID used:135614 OG02079|WP\_004089607.1 | endoribonuclease YbeY | taxID used:135614 OG02083|WP\_020851923.1 | acetyl-CoA carboxylase biotin carboxyl carrier protein | taxID used:135614 OG02087|WP\_010893499.1 | phosphopantetheine adenylyltransferase | taxID used:135614 OG00208|WP\_038227902.1 | ligand-gated channel | taxID used:135614 OG02090|WP\_010892970.1 | ribosomal-protein-alanine N-acetyltransferase | taxID used:135614 OG02093|WP\_010893532.1 | manganese-binding transcriptional regulator MntR | taxID used:135614 OG02094|WP\_004088666.1 | peptidylprolyl isomerase | taxID used:135614 OG02096|WP\_010892578.1 | type IV pilus modification protein PilV | taxID used:135614 OG02097|WP\_004090587.1 | rod shape-determining protein MreD | taxID used:135614 OG02099|WP\_004088178.1 | peptide-methionine (R)-S-oxide reductase | taxID used:135614 OG02100|WP\_004086621.1 | 23S rRNA (pseudouridine(1915)-N(3))-methyltransferase RlmH | taxID used:135614 OG02111|WP\_031336885.1 | diguanylate cyclase | taxID used:135614 OG02115|WP\_004083722.1 | SET domain-containing protein-lysine N-methyltransferase | taxID used:135614 OG02120|WP\_031336929.1 | histidine phosphatase family protein | taxID used:135614 OG02121|WP\_038229263.1 | EVE domain-containing protein | taxID used:135614 OG02122|WP\_004084553.1 | tRNA (adenosine(37)-N6)-threonylcarbamoyltransferase complex ATPase subunit type 1 TsaE | taxID used:135614 OG02126|WP\_004089636.1 | DUF494 domain-containing protein | taxID used:135614 OG02132|WP\_023906109.1 | hypothetical protein | taxID used:135614 OG02134|WP\_004085473.1 | tRNA (cytidine(34)-2'-O)-methyltransferase | taxID used:135614 OG02136|WP\_004572890.1 | hypothetical protein | taxID used:135614 OG02138|WP\_012338106.1 | GNAT family N-acetyltransferase | taxID used:135614 OG02139|WP\_004086409.1 | transcription antitermination factor NusB | taxID used:135614 OG00213|WP\_031336549.1 | methyl-accepting chemotaxis protein | taxID used:135614 OG02140|WP\_010895047.1 | 30S ribosomal protein S7 | taxID used:135614 OG02141|WP\_004085454.1 | chemotaxis protein CheW | taxID used:135614 OG02148|WP\_004086550.1 | hypothetical protein | taxID used:135614 OG02149|WP\_011097633.1 | transcription elongation factor GreA | taxID used:135614 OG02155|WP\_004086121.1 | ribonuclease HI | taxID used:135614 OG02158|WP\_010893664.1 | F0F1 ATP synthase subunit B | taxID used:135614 OG02161|WP\_024748779.1 | cytochrome c biogenesis protein CcmE | taxID used:135614 OG02162|WP\_010894300.1 | thioredoxin family protein | taxID used:135614 OG02166|WP\_004083417.1 | Holliday junction resolvase RuvX | taxID used:135614 OG02167|WP\_010892925.1 | bacterioferritin | taxID used:135614 OG02171|WP\_004083563.1 | type IV pilin protein | taxID used:135614 OG02173|WP\_058564932.1 | azurin | taxID used:135614 OG02182|WP\_031336766.1 | SUF system Fe-S cluster assembly regulator | taxID used:135614 OG02184|WP\_010894755.1 | hypothetical protein | taxID used:135614 OG02188|WP\_004087579.1 | dUTP diphosphatase | taxID used:135614 OG00218|WP\_038229932.1 | excinuclease ABC subunit UvrB | taxID used:135614 OG02195|WP\_004088329.1 | hypothetical protein | taxID used:135614 OG02202|WP\_010894046.1 | glycine zipper 2TM domain-containing protein | taxID used:135614 OG02206|WP\_020851432.1 | CopD family protein | taxID used:135614 OG02207|WP\_038211276.1 | transcriptional regulator MraZ | taxID used:135614 OG02208|WP\_004085838.1 | type II toxin-antitoxin system RatA family toxin | taxID used:135614 OG02213|WP\_010893996.1 | DUF192 domain-containing protein | taxID used:135614 OG02216|WP\_010894960.1 | pilin | taxID used:135614 OG02217|WP\_038211361.1 | hypothetical protein | taxID used:135614 OG02219|WP\_057682412.1 | phosphotransferase | taxID used:135614 OG00221|WP\_046420843.1 | DNA helicase Rep | taxID used:135614 OG02222|WP\_020852035.1 | guanine-specific ribonuclease N1 and T1 | taxID used:135614 OG02223|WP\_058569726.1 | hypothetical protein | taxID used:135614 OG02228|WP\_004086143.1 | ribosome silencing factor | taxID used:135614 OG02240|WP\_020852340.1 | Fe/S cluster cysteine desulfuration protein | taxID used:135614 OG02244|WP\_023906152.1 | hypothetical protein | taxID used:135614 OG02245|WP\_004572855.1 | VirK protein | taxID used:135614 OG00224|WP\_088372189.1 | M1 family peptidase | taxID used:135614 OG02250|WP\_057683326.1 | cytochrome c-type biogenesis protein CcmH | taxID used:135614 OG02251|WP\_021358549.1 | type II 3-dehydroquinate dehydratase | taxID used:135614 OG02254|WP\_004083857.1 | MarR family transcriptional regulator | taxID used:135614 OG02255|WP\_031337016.1 | ClpXP protease specificity-enhancing factor | taxID used:135614 OG02260|WP\_004090345.1 | 30S ribosomal protein S6 | taxID used:135614 OG02265|WP\_031336933.1 | succinate dehydrogenase, cytochrome b556 subunit | taxID used:135614 OG02269|WP\_057683058.1 | NfeD family protein | taxID used:135614 OG02272|WP\_004083686.1 | type II secretion system protein GspG | taxID used:135614 OG02274|WP\_058569577.1 | DNA polymerase III subunit chi | taxID used:135614 OG00227|WP\_058564252.1 | lytic murein transglycosylase | taxID used:135614 OG02280|WP\_011097832.1 | protein TolR | taxID used:135614 OG02281|WP\_031336780.1 | fluoride efflux transporter CrcB | taxID used:135614 OG02282|WP\_031336563.1 | tol-pal system-associated acyl-CoA thioesterase | taxID used:135614 OG02285|WP\_027700190.1 | polymer-forming cytoskeletal family protein | taxID used:135614 OG02288|WP\_010894458.1 | hypothetical protein | taxID used:135614 OG02291|WP\_080679637.1 | hypothetical protein | taxID used:135614 OG02293|WP\_004083669.1 | 50S ribosomal protein L13 | taxID used:135614 OG02297|WP\_010894293.1 | organic hydroperoxide resistance protein | taxID used:135614 OG02299|WP\_004087842.1 | biopolymer transporter ExbD | taxID used:135614 OG00229|WP\_004086556.1 | peptidyl-prolyl cis-trans isomerase | taxID used:135614 OG02310|WP\_027700564.1 | 50S ribosomal protein L11 | taxID used:135614 OG02318|WP\_004083578.1 | nucleoside-diphosphate kinase | taxID used:135614 OG00231|WP\_004084578.1 | threonine--tRNA ligase | taxID used:135614 OG02320|WP\_004085082.1 | biopolymer transporter ExbD | taxID used:135614 OG02322|WP\_004083709.1 | DNA-binding protein H-NS | taxID used:135614 OG00232|WP\_010892715.1 | DUF885 domain-containing protein | taxID used:135614 OG02336|WP\_004086531.1 | 50S ribosomal protein L16 | taxID used:135614 OG02338|WP\_004090067.1 | F0F1 ATP synthase subunit epsilon | taxID used:135614 OG00233|WP\_020850921.1 | transglutaminase | taxID used:135614 OG02340|WP\_020852034.1 | hypothetical protein | taxID used:135614 OG02343|WP\_004085459.1 | response regulator | taxID used:135614 OG02347|WP\_004086588.1 | MerC domain-containing protein | taxID used:135614 OG02349|WP\_046419512.1 | antibiotic resistance protein VanZ | taxID used:135614 OG02350|WP\_011098077.1 | ferric iron uptake transcriptional regulator | taxID used:135614 OG02351|WP\_021358177.1 | DUF4398 domain-containing protein | taxID used:135614 OG02352|WP\_020852461.1 | DNA-binding protein H-NS | taxID used:135614 OG00235|WP\_004088280.1 | oligopeptide transporter, OPT family | taxID used:135614 OG02361|WP\_023906384.1 | hypothetical protein | taxID used:135614 OG02366|WP\_075584703.1 | ribonuclease P protein component | taxID used:135614 OG02374|WP\_004085540.1 | 50S ribosomal protein L19 | taxID used:135614 OG00237|WP\_057683503.1 | ABC transporter ATP-binding protein | taxID used:135614 OG00238|WP\_010894369.1 | potassium transporter Kup | taxID used:135614 OG02393|WP\_004083684.1 | prepilin-type N-terminal cleavage/methylation domain-containing protein | taxID used:135614 OG02399|WP\_011097555.1 | type II toxin-antitoxin system VapC family toxin | taxID used:135614 OG02400|WP\_011098322.1 | response regulator | taxID used:135614 OG02401|WP\_004085841.1 | outer membrane protein assembly factor BamE | taxID used:135614 OG02402|WP\_010894423.1 | YkgJ family cysteine cluster protein | taxID used:135614 OG02407|WP\_010892724.1 | glycine cleavage system protein GcvH | taxID used:135614 OG02408|WP\_004086543.1 | 30S ribosomal protein S11 | taxID used:135614 OG00240|WP\_046418193.1 | polysaccharide biosynthesis protein | taxID used:135614 OG02410|WP\_004087939.1 | preprotein translocase subunit SecG | taxID used:135614 OG02416|WP\_004086536.1 | 30S ribosomal protein S8 | taxID used:135614 OG02418|WP\_010893736.1 | hypothetical protein | taxID used:135614 OG00241|WP\_046418948.1 | phosphogluconate dehydratase | taxID used:135614 OG02428|WP\_011097604.1 | hypothetical protein | taxID used:135614 OG00242|WP\_060872172.1 | molecular chaperone HtpG | taxID used:135614 OG02431|WP\_004086104.1 | Co2+/Mg2+ efflux protein ApaG | taxID used:135614 OG00243|WP\_004083434.1 | 1-deoxy-D-xylulose-5-phosphate synthase | taxID used:135614 OG02444|WP\_004083670.1 | 30S ribosomal protein S9 | taxID used:135614 OG02449|WP\_004083781.1 | PTS fructose IIA subunit family protein | taxID used:135614 OG00245|WP\_023907200.1 | arginine decarboxylase | taxID used:135614 OG02464|WP\_004083584.1 | response regulator | taxID used:135614 OG02473|WP\_010892935.1 | iron-sulfur cluster insertion protein ErpA | taxID used:135614 OG02480|WP\_004085448.1 | DNA-binding protein | taxID used:135614 OG02483|WP\_004089926.1 | RidA family protein | taxID used:135614 OG02485|WP\_004086272.1 | aspartate 1-decarboxylase | taxID used:135614 OG02490|WP\_004085491.1 | XRE family transcriptional regulator | taxID used:135614 OG02491|WP\_010892919.1 | hypothetical protein | taxID used:135614 OG02494|WP\_004085551.1 | hypothetical protein | taxID used:135614 OG02496|WP\_020851197.1 | DUF4124 domain-containing protein | taxID used:135614 OG02497|WP\_010892777.1 | 30S ribosome-binding factor RbfA | taxID used:135614 OG02499|WP\_004085860.1 | diacylglycerol kinase | taxID used:135614 OG02507|WP\_004084687.1 | 30S ribosomal protein S12 | taxID used:135614 OG02509|WP\_010893590.1 | succinate dehydrogenase, hydrophobic membrane anchor protein | taxID used:135614 OG02512|WP\_004086459.1 | XRE family transcriptional regulator | taxID used:135614 OG02514|WP\_004083551.1 | YraN family protein | taxID used:135614 OG02521|WP\_010892975.1 | DNA-binding protein | taxID used:135614 OG02523|WP\_031336173.1 | 6-carboxytetrahydropterin synthase QueD | taxID used:135614 OG02535|WP\_031336149.1 | Spx/MgsR family RNA polymerase-binding regulatory protein | taxID used:135614 OG02537|WP\_004086533.1 | 50S ribosomal protein L14 | taxID used:135614 OG02538|WP\_010892965.1 | dihydroneopterin aldolase | taxID used:135614 OG02546|WP\_011097688.1 | cell division protein FtsB | taxID used:135614 OG02547|WP\_010893263.1 | 50S ribosomal protein L20 | taxID used:135614 OG02553|WP\_011097560.1 | NADH-quinone oxidoreductase subunit A | taxID used:135614 OG02555|WP\_004085637.1 | hypothetical protein | taxID used:135614 OG02558|WP\_004086542.1 | 30S ribosomal protein S13 | taxID used:135614 OG00255|WP\_057682439.1 | beta-galactosidase | taxID used:135614 OG02561|WP\_020852228.1 | histidine triad nucleotide-binding protein | taxID used:135614 OG02563|WP\_004083585.1 | DUF486 domain-containing protein | taxID used:135614 OG02569|WP\_014607493.1 | MerR family transcriptional regulator | taxID used:135614 OG02577|WP\_010892823.1 | hypothetical protein | taxID used:135614 OG02581|WP\_004083467.1 | type IV pilus assembly PilZ | taxID used:135614 OG00258|WP\_038228562.1 | ABC transporter ATP-binding protein | taxID used:135614 OG02596|WP\_004086538.1 | 50S ribosomal protein L18 | taxID used:135614 OG02598|WP\_004089237.1 | cytochrome o ubiquinol oxidase subunit IV | taxID used:135614 OG02599|WP\_010894010.1 | PilZ domain-containing protein | taxID used:135614 OG00259|WP\_014607668.1 | RNA polymerase sigma factor RpoD | taxID used:135614 OG02601|WP\_004084923.1 | RidA family protein | taxID used:135614 OG02603|WP\_020852709.1 | thioredoxin | taxID used:135614 OG02607|WP\_004089069.1 | non-heme iron oxygenase ferredoxin subunit | taxID used:135614 OG02611|WP\_004084639.1 | iron-sulfur cluster assembly accessory protein | taxID used:135614 OG02617|WP\_004083506.1 | divalent-cation tolerance protein CutA | taxID used:135614 OG02618|WP\_004085696.1 | hypothetical protein | taxID used:135614 OG02619|WP\_020851391.1 | nitrogen regulatory protein P-II 1 | taxID used:135614 OG02624|WP\_004088312.1 | Trp operon repressor | taxID used:135614 OG02628|WP\_004084667.1 | ferredoxin family protein | taxID used:135614 OG02648|WP\_004572981.1 | J domain-containing protein | taxID used:135614 OG02649|WP\_010894885.1 | hypothetical protein | taxID used:135614 OG02667|WP\_010893673.1 | 50S ribosomal protein L22 | taxID used:135614 OG02670|WP\_004083751.1 | ATP-dependent Clp protease adapter ClpS | taxID used:135614 OG02688|WP\_010893574.1 | DUF1820 family protein | taxID used:135614 OG00268|WP\_046418733.1 | ferrous iron transporter B | taxID used:135614 OG02697|WP\_004088703.1 | DNA uptake protein | taxID used:135614 OG00269|WP\_004087662.1 | dihydroxy-acid dehydratase | taxID used:135614 OG02702|WP\_004086561.1 | thioredoxin | taxID used:135614 OG02714|WP\_004088409.1 | 50S ribosomal protein L21 | taxID used:135614 OG02715|WP\_020851063.1 | DUF4156 domain-containing protein | taxID used:135614 OG00271|WP\_004084485.1 | penicillin-binding protein 2 | taxID used:135614 OG02720|WP\_004088463.1 | BolA family transcriptional regulator | taxID used:135614 OG02722|WP\_004572794.1 | YbaB/EbfC family nucleoid-associated protein | taxID used:135614 OG02731|WP\_027700095.1 | DUF485 domain-containing protein | taxID used:135614 OG02739|WP\_004086577.1 | 50S ribosomal protein L24 | taxID used:135614 OG00273|WP\_038232083.1 | single-stranded-DNA-specific exonuclease RecJ | taxID used:135614 OG02746|WP\_004084751.1 | Grx4 family monothiol glutaredoxin | taxID used:135614 OG00274|WP\_010894354.1 | phosphomethylpyrimidine synthase ThiC | taxID used:135614 OG02751|WP\_004087527.1 | glutaredoxin 3 | taxID used:135614 OG02756|WP\_004085973.1 | integration host factor subunit beta | taxID used:135614 OG02758|WP\_010893917.1 | EF-hand domain-containing protein | taxID used:135614 OG00276|WP\_023907197.1 | glutamine--fructose-6-phosphate aminotransferase | taxID used:135614 OG02778|WP\_031345883.1 | hypothetical protein | taxID used:135614 OG00277|WP\_012382432.1 | RNA helicase | taxID used:135614 OG02782|WP\_004089684.1 | hypothetical protein | taxID used:135614 OG02794|WP\_004086535.1 | 30S ribosomal protein S14 | taxID used:135614 OG02802|WP\_058569559.1 | YhbY family RNA-binding protein | taxID used:135614 OG00280|WP\_010894275.1 | DNA polymerase III subunit gamma/tau | taxID used:135614 OG00281|WP\_010893591.1 | succinate dehydrogenase flavoprotein subunit | taxID used:135614 OG02827|WP\_010892947.1 | STAS domain-containing protein | taxID used:135614 OG02830|WP\_010894003.1 | DNA-directed RNA polymerase subunit omega | taxID used:135614 OG02833|WP\_004086434.1 | hypothetical protein | taxID used:135614 OG00283|WP\_088372274.1 | formylglycine-generating enzyme family protein | taxID used:135614 OG02842|WP\_080507217.1 | LapA family protein | taxID used:135614 OG02844|WP\_004089989.1 | DUF2782 domain-containing protein | taxID used:135614 OG02850|WP\_004084568.1 | integration host factor subunit alpha | taxID used:135614 OG02851|WP\_004085382.1 | cell division protein ZapA | taxID used:135614 OG00285|WP\_057682928.1 | elongation factor 4 | taxID used:135614 OG02861|WP\_020851042.1 | NADH-quinone oxidoreductase subunit NuoK | taxID used:135614 OG02870|WP\_004087908.1 | YfhL family 4Fe-4S dicluster ferredoxin | taxID used:135614 OG00287|WP\_004084501.1 | autotransporter domain-containing esterase | taxID used:135614 OG02880|WP\_024748689.1 | F0F1 ATP synthase subunit C | taxID used:135614 OG02895|WP\_004090455.1 | beta-hydroxyacyl-ACP dehydratase | taxID used:135614 OG02907|WP\_023906865.1 | membrane protein insertion efficiency factor YidD | taxID used:135614 OG02928|WP\_004088683.1 | molecular chaperone GroES | taxID used:135614 OG00292|WP\_088578238.1 | glutamine--tRNA ligase/YqeY domain fusion protein | taxID used:135614 OG02938|WP\_004084034.1 | DUF493 domain-containing protein | taxID used:135614 OG00293|WP\_023907627.1 | aspartate--tRNA ligase | taxID used:135614 OG02951|WP\_011097829.1 | Fe(2+)-trafficking protein | taxID used:135614 OG02952|WP\_046418477.1 | hypothetical protein | taxID used:135614 OG02967|WP\_031336129.1 | hypothetical protein | taxID used:135614 OG02969|WP\_004086555.1 | HU family DNA-binding protein | taxID used:135614 OG02970|WP\_004085558.1 | RNA-binding protein Hfq | taxID used:135614 OG02973|WP\_004086525.1 | 30S ribosomal protein S19 | taxID used:135614 OG00297|WP\_031336883.1 | outer membrane protein assembly factor | taxID used:135614 OG03001|WP\_004085493.1 | Fis family transcriptional regulator | taxID used:135614 OG00300|WP\_031337108.1 | ShlB/FhaC/HecB family hemolysin secretion/activation protein | taxID used:135614 OG03020|WP\_024749209.1 | 30S ribosomal protein S17 | taxID used:135614 OG03025|WP\_010893184.1 | exodeoxyribonuclease 7 small subunit | taxID used:135614 OG00302|WP\_046418009.1 | DNA primase | taxID used:135614 OG03033|WP\_004089222.1 | HPr family phosphocarrier protein | taxID used:135614 OG03036|WP\_027700582.1 | hypothetical protein | taxID used:135614 OG00303|WP\_020851262.1 | ABC transporter ATP-binding protein/permease | taxID used:135614 OG03042|WP\_004090451.1 | acyl carrier protein | taxID used:135614 OG03053|WP\_004084759.1 | Trm112 family protein | taxID used:135614 OG00305|WP\_023906243.1 | hypothetical protein | taxID used:135614 OG03060|WP\_010894866.1 | 30S ribosomal protein S20 | taxID used:135614 OG03067|WP\_004084487.1 | cell division protein FtsL | taxID used:135614 OG03083|WP\_004086259.1 | 30S ribosomal protein S15 | taxID used:135614 OG03085|WP\_004087643.1 | 30S ribosomal protein S16 | taxID used:135614 OG03104|WP\_060871965.1 | succinate dehydrogenase assembly factor 2 family protein | taxID used:135614 OG00310|WP\_060871718.1 | type IV-A pilus assembly ATPase PilB | taxID used:135614 OG03118|WP\_004085949.1 | 50S ribosomal protein L27 | taxID used:135614 OG00311|WP\_004087620.1 | asparagine synthase B | taxID used:135614 OG03132|WP\_004086547.1 | accessory factor UbiK family protein | taxID used:135614 OG00313|WP\_004086424.1 | malate dehydrogenase (quinone) | taxID used:135614 OG03146|WP\_004090594.1 | cell division topological specificity factor MinE | taxID used:135614 OG00314|WP\_004083620.1 | GGDEF domain-containing response regulator | taxID used:135614 OG00316|WP\_011097507.1 | arginine--tRNA ligase | taxID used:135614 OG03174|WP\_004083672.1 | type B 50S ribosomal protein L31 | taxID used:135614 OG00318|WP\_024748809.1 | transcription termination factor Rho | taxID used:135614 OG03190|WP\_010893451.1 | ferrous iron transport protein A | taxID used:135614 OG03192|WP\_004572808.1 | acetolactate synthase | taxID used:135614 OG00319|WP\_038233155.1 | proline--tRNA ligase | taxID used:135614 OG00320|WP\_088371784.1 | LppC family lipoprotein | taxID used:135614 OG03216|WP\_012337924.1 | plasmid protein | taxID used:135614 OG03217|WP\_010894792.1 | RnfH family protein | taxID used:135614 OG00321|WP\_038229363.1 | Na+/H+ antiporter | taxID used:135614 OG00322|WP\_057682738.1 | phosphoethanolamine transferase | taxID used:135614 OG03236|WP\_004083472.1 | MULTISPECIES: acyl carri | taxID used:135614 OG03237|WP\_004086565.1 | 50S ribosomal protein L28 | taxID used:135614 OG03245|WP\_004087333.1 | protein SlyX | taxID used:135614 OG00324|WP\_004088642.1 | Kef family K(+) transporter | taxID used:135614 OG00325|WP\_027700575.1 | type II secretion system protein GspE | taxID used:135614 OG00326|WP\_023907466.1 | DNA repair protein RecN | taxID used:135614 OG00327|WP\_004083995.1 | electron transfer flavoprotein-ubiquinone oxidoreductase | taxID used:135614 OG03287|WP\_010894981.1 | 30S ribosomal protein S18 | taxID used:135614 OG03297|WP\_004083623.1 | bacterioferritin-associated ferredoxin | taxID used:135614 OG03315|WP\_004085381.1 | TIGR02449 family protein | taxID used:135614 OG03316|WP\_012337925.1 | hypothetical protein | taxID used:135614 OG03318|WP\_004084681.1 | cold-shock protein | taxID used:135614 OG00331|WP\_004090560.1 | CTP synthetase | taxID used:135614 OG03349|WP\_011097601.1 | DUF1674 domain-containing protein | taxID used:135614 OG00334|WP\_046420038.1 | ubiquinone biosynthesis regulatory protein kinase UbiB | taxID used:135614 OG03351|WP\_004088694.1 | DUF465 domain-containing protein | taxID used:135614 OG03354|WP\_004083749.1 | translation initiation factor IF-1 | taxID used:135614 OG00335|WP\_057683315.1 | 30S ribosomal protein S1 | taxID used:135614 OG00336|WP\_011098235.1 | energy-dependent translational throttle protein EttA | taxID used:135614 OG00337|WP\_038228516.1 | membrane protein | taxID used:135614 OG03388|WP\_004083596.1 | 30S ribosomal protein S21 | taxID used:135614 OG03396|WP\_004085529.1 | carbon storage regulator | taxID used:135614 OG03429|WP\_004085832.1 | cold-shock protein | taxID used:135614 OG03454|WP\_010892647.1 | zinc-finger domain-containing protein | taxID used:135614 OG03464|WP\_031345749.1 | 50S ribosomal protein L29 | taxID used:135614 OG00346|WP\_088371685.1 | peptide synthase | taxID used:135614 OG03485|WP\_004090468.1 | sulfur carrier protein ThiS | taxID used:135614 OG03491|WP\_004090402.1 | 50S ribosomal protein L35 | taxID used:135614 OG00034|WP\_057682376.1 | chromosome segregation protein SMC | taxID used:135614 OG00351|WP\_010895110.1 | RNA helicase | taxID used:135614 OG03526|WP\_023907191.1 | hypothetical protein | taxID used:135614 OG03530|WP\_010894283.1 | 50S ribosomal protein L32 | taxID used:135614 OG03545|WP\_075584665.1 | 50S ribosomal protein L30 | taxID used:135614 OG00356|WP\_004087188.1 | peptide chain release factor 3 | taxID used:135614 OG00357|WP\_031337130.1 | YdiU family protein | taxID used:135614 OG03602|WP\_031336773.1 | hypothetical protein | taxID used:135614 OG00360|WP\_020851064.1 | bifunctional phosphoribosylaminoimidazolecarboxamide formyltransferase/IMP cyclohydrolase PurH | taxID used:135614 OG03622|WP\_004086643.1 | 30S ribosomal protein THX | taxID used:135614 OG03647|WP\_004086566.1 | 50S ribosomal protein L33 | taxID used:135614 OG00364|WP\_057683313.1 | GMP synthase (glutamine-hydrolyzing) | taxID used:135614 OG00369|WP\_031336299.1 | EAL domain-containing protein | taxID used:135614 OG00376|WP\_023907644.1 | 2-isopropylmalate synthase | taxID used:135614 OG00379|WP\_088577796.1 | alpha/beta hydrolase | taxID used:135614 OG00380|WP\_071869861.1 | S10 family peptidase | taxID used:135614 OG00381|WP\_010894357.1 | peptide MFS transporter | taxID used:135614 OG00382|WP\_010893662.1 | F0F1 ATP synthase subunit alpha | taxID used:135614 OG00387|WP\_027700101.1 | peptidase S1 | taxID used:135614 OG00390|WP\_023908123.1 | exopolyphosphatase | taxID used:135614 OG00392|WP\_088578604.1 | glycerol kinase | taxID used:135614 OG00393|WP\_024749267.1 | fumarate hydratase | taxID used:135614 OG00398|WP\_080939581.1 | hypothetical protein | taxID used:135614 OG00399|WP\_010893691.1 | ATP-binding protein | taxID used:135614 OG00404|WP\_004572953.1 | transcription termination/antitermination protein NusA | taxID used:135614 OG00405|WP\_027700228.1 | glutamate synthase subunit beta | taxID used:135614 OG00407|WP\_058564434.1 | anthranilate synthase component I | taxID used:135614 OG00409|WP\_058564509.1 | Rne/Rng family ribonuclease | taxID used:135614 OG00410|WP\_004089062.1 | Fe-S cluster assembly protein SufB | taxID used:135614 OG00414|WP\_081364544.1 | UDP-N-acetylmuramoyl-L-alanyl-D-glutamate--2,6-diaminopimelate ligase | taxID used:135614 OG00415|WP\_004085038.1 | adenosylmethionine--8-amino-7-oxononanoate transaminase | taxID used:135614 OG00416|WP\_038229315.1 | amidophosphoribosyltransferase | taxID used:135614 OG00419|WP\_058569579.1 | leucyl aminopeptidase | taxID used:135614 OG00422|WP\_024749123.1 | exodeoxyribonuclease I | taxID used:135614 OG00423|WP\_023906336.1 | amino acid permease | taxID used:135614 OG00425|WP\_046418573.1 | pyruvate kinase | taxID used:135614 OG00426|WP\_058564382.1 | NADH-quinone oxidoreductase subunit NuoN | taxID used:135614 OG00427|WP\_004089347.1 | S-adenosyl-L-homocysteine hydrolase | taxID used:135614 OG00432|WP\_046418200.1 | IMP dehydrogenase | taxID used:135614 OG00433|WP\_051606222.1 | two-component sensor histidine kinase | taxID used:135614 OG00449|WP\_058564344.1 | S41 family peptidase | taxID used:135614 OG00044|WP\_004085780.1 | acriflavine resistance protein B | taxID used:135614 OG00453|WP\_023906105.1 | UDP-N-acetylmuramate:L-alanyl-gamma-D-glutamyl-meso-diaminopimelate ligase | taxID used:135614 OG00454|WP\_020851010.1 | peptidase S1 | taxID used:135614 OG00455|WP\_088371846.1 | amino acid permease | taxID used:135614 OG00456|WP\_010894982.1 | asparagine--tRNA ligase | taxID used:135614 OG00457|WP\_020851208.1 | UDP-N-acetylmuramate--L-alanine ligase | taxID used:135614 OG00460|WP\_088577601.1 | alpha-L-fucosidase | taxID used:135614 OG00461|WP\_004084450.1 | glutamate--tRNA ligase | taxID used:135614 OG00462|WP\_020851308.1 | HlyD family type I secretion periplasmic adaptor subunit | taxID used:135614 OG00463|WP\_031337514.1 | mannose-1-phosphate guanylyltransferase/mannose-6-phosphate isomerase | taxID used:135614 OG00466|WP\_010894314.1 | nitrogen regulation protein NR(I) | taxID used:135614 OG00471|WP\_038227603.1 | divalent metal cation transporter MntH | taxID used:135614 OG00475|WP\_023908060.1 | chaperone SurA | taxID used:135614 OG00477|WP\_058569761.1 | exopolysaccharide biosynthesis protein | taxID used:135614 OG00478|WP\_010893660.1 | F0F1 ATP synthase subunit beta | taxID used:135614 OG00480|WP\_031338065.1 | sigma-54-dependent Fis family transcriptional regulator | taxID used:135614 OG00481|WP\_058564945.1 | class II fumarate hydratase | taxID used:135614 OG00485|WP\_046420507.1 | histidine--tRNA ligase | taxID used:135614 OG00488|WP\_010893929.1 | glutamate--cysteine ligase | taxID used:135614 OG00489|WP\_004085376.1 | DUF1631 family protein | taxID used:135614 OG00490|WP\_038227947.1 | ribosome biogenesis GTPase Der | taxID used:135614 OG00491|WP\_020851922.1 | acetyl-CoA carboxylase biotin carboxylase subunit | taxID used:135614 OG00493|WP\_057682418.1 | UDP-N-acetylmuramoylalanyl-D-glutamyl-2, 6-diaminopimelate--D-alanyl-D-alanine ligase | taxID used:135614 OG00499|WP\_004090028.1 | hypothetical protein | taxID used:135614 OG00500|WP\_010892880.1 | FAD-binding oxidoreductase | taxID used:135614 OG00502|WP\_004085324.1 | cytochrome c oxidase subunit II | taxID used:135614 OG00503|WP\_004083516.1 | CBS domain-containing protein | taxID used:135614 OG00505|WP\_004084964.1 | serine hydrolase | taxID used:135614 OG00508|WP\_046419044.1 | bifunctional N-acetylglucosamine-1-phosphate uridyltransferase/glucosamine-1-phosphate acetyltransferase | taxID used:135614 OG00509|WP\_027700773.1 | DUF445 domain-containing protein | taxID used:135614 OG00050|WP\_058564715.1 | efflux RND transporter permease subunit | taxID used:135614 OG00512|WP\_027700554.1 | adenylosuccinate lyase | taxID used:135614 OG00514|WP\_023906726.1 | replication-associated recombination protein A | taxID used:135614 OG00519|WP\_010892943.1 | DNA recombination protein RmuC | taxID used:135614 OG00051|WP\_023906846.1 | membrane protein | taxID used:135614 OG00520|WP\_020852871.1 | signal recognition particle protein | taxID used:135614 OG00521|WP\_020852941.1 | virulence factor family protein | taxID used:135614 OG00522|WP\_046420849.1 | MATE family multidrug exporter | taxID used:135614 OG00524|WP\_004086224.1 | phosphomannomutase/phosphoglucomutase | taxID used:135614 OG00525|WP\_023906937.1 | sugar transferase | taxID used:135614 OG00529|WP\_057683102.1 | allantoinase | taxID used:135614 OG00535|WP\_027700579.1 | glutamate-1-semialdehyde 2,1-aminomutase | taxID used:135614 OG00537|WP\_004086025.1 | N-ethylammeline chlorohydrolase | taxID used:135614 OG00538|WP\_010895004.1 | type I secretion system protein TolC | taxID used:135614 OG00542|WP\_004084867.1 | GTP-binding protein | taxID used:135614 OG00543|WP\_058564377.1 | NADH-quinone oxidoreductase subunit D | taxID used:135614 OG00545|WP\_058564484.1 | mechanosensitive ion channel family protein | taxID used:135614 OG00546|WP\_031336524.1 | Xaa-Pro aminopeptidase | taxID used:135614 OG00550|WP\_031336966.1 | dicarboxylate/amino acid:cation symporter | taxID used:135614 OG00553|WP\_046418128.1 | tRNA lysidine(34) synthetase TilS | taxID used:135614 OG00554|WP\_046417781.1 | RNA helicase | taxID used:135614 OG00556|WP\_057683582.1 | tRNA uridine-5-carboxymethylaminomethyl(34) synthesis GTPase MnmE | taxID used:135614 OG00559|WP\_058569562.1 | membrane protein | taxID used:135614 OG00055|WP\_004087603.1 | valine--tRNA ligase | taxID used:135614 OG00560|WP\_046418225.1 | chromosomal replication initiator protein DnaA | taxID used:135614 OG00561|WP\_024748939.1 | citrate transporter | taxID used:135614 OG00562|WP\_023906495.1 | Tol-Pal system beta propeller repeat protein TolB | taxID used:135614 OG00563|WP\_004085241.1 | acetylglutamate kinase | taxID used:135614 OG00564|WP\_038211076.1 | alkaline phosphatase family protein | taxID used:135614 OG00566|WP\_042466566.1 | ABC transporter substrate-binding protein | taxID used:135614 OG00569|WP\_010893371.1 | D-amino acid dehydrogenase | taxID used:135614 OG00570|WP\_004084023.1 | HlyC/CorC family transporter | taxID used:135614 OG00571|WP\_057683263.1 | 3-phosphoshikimate 1-carboxyvinyltransferase | taxID used:135614 OG00579|WP\_020851469.1 | DUF418 domain-containing protein | taxID used:135614 OG00583|WP\_021358468.1 | acyl-CoA synthetase | taxID used:135614 OG00585|WP\_020851473.1 | dicarboxylate/amino acid:cation symporter | taxID used:135614 OG00587|WP\_010894035.1 | citrate synthase | taxID used:135614 OG00590|WP\_057682345.1 | glutamyl-tRNA reductase | taxID used:135614 OG00596|WP\_058569342.1 | dicarboxylate/amino acid:cation symporter | taxID used:135614 OG00598|WP\_004083457.1 | serine--tRNA ligase | taxID used:135614 OG00599|WP\_004086551.1 | trigger factor | taxID used:135614 OG00601|WP\_020851831.1 | nitrate ABC transporter ATP-binding protein | taxID used:135614 OG00602|WP\_027700705.1 | bifunctional tetrahydrofolate synthase/dihydrofolate synthase | taxID used:135614 OG00605|WP\_023906922.1 | ribosomal RNA small subunit methyltransferase B | taxID used:135614 OG00606|WP\_004088628.1 | alpha/beta hydrolase | taxID used:135614 OG00607|WP\_058564442.1 | threonine synthase | taxID used:135614 OG00614|WP\_010894994.1 | flavodoxin-dependent (E)-4-hydroxy-3-methylbut-2-enyl-diphosphate synthase | taxID used:135614 OG00617|WP\_057683365.1 | phosphopyruvate hydratase | taxID used:135614 OG00618|WP\_038228891.1 | ATP-dependent Clp protease ATP-binding subunit ClpX | taxID used:135614 OG00620|WP\_038211284.1 | putative lipid II flippase FtsW | taxID used:135614 OG00621|WP\_020851306.1 | pyridoxal phosphate-dependent aminotransferase | taxID used:135614 OG00624|WP\_046419397.1 | multifunctional CCA tRNA nucleotidyl transferase/2'3'-cyclic phosphodiesterase/2'nucleotidase/phosphatase | taxID used:135614 OG00626|WP\_031337004.1 | serine hydroxymethyltransferase | taxID used:135614 OG00627|WP\_004084972.1 | AGE family epimerase/isomerase | taxID used:135614 OG00629|WP\_038283864.1 | SufS family cysteine desulfurase | taxID used:135614 OG00630|WP\_004086282.1 | polynucleotide adenylyltransferase PcnB | taxID used:135614 OG00631|WP\_011097661.1 | HlyD family efflux transporter periplasmic adaptor subunit | taxID used:135614 OG00063|WP\_058564615.1 | insulinase family protein | taxID used:135614 OG00643|WP\_004083566.1 | pilus assembly protein PilW | taxID used:135614 OG00647|WP\_004085192.1 | ABC transporter permease | taxID used:135614 OG00649|WP\_012382717.1 | 23S rRNA (adenine(2503)-C(2))-methyltransferase RlmN | taxID used:135614 OG00653|WP\_004084469.1 | cell division protein FtsA | taxID used:135614 OG00654|WP\_058569384.1 | MFS transporter | taxID used:135614 OG00655|WP\_010894654.1 | phosphoglycerate dehydrogenase | taxID used:135614 OG00659|WP\_031336297.1 | outer membrane protein assembly factor BamB | taxID used:135614 OG00660|WP\_046420691.1 | efflux RND transporter periplasmic adaptor subunit | taxID used:135614 OG00661|WP\_038228585.1 | sensor histidine kinase | taxID used:135614 OG00667|WP\_058569584.1 | bifunctional phosphopantothenoylcysteine decarboxylase/phosphopantothenate--cysteine ligase CoaBC | taxID used:135614 OG00668|WP\_046417619.1 | NO-inducible flavohemoprotein | taxID used:135614 OG00672|WP\_031336812.1 | tryptophan synthase subunit beta | taxID used:135614 OG00673|WP\_004572970.1 | aminopeptidase P family protein | taxID used:135614 OG00686|WP\_020851546.1 | 2-methylaconitate cis-trans isomerase PrpF | taxID used:135614 OG00688|WP\_004085243.1 | argininosuccinate synthase | taxID used:135614 OG00690|WP\_023906270.1 | UDP-glucuronate--glycolipid 2-beta-glucuronosyltransferase | taxID used:135614 OG00692|WP\_010894678.1 | D-alanyl-D-alanine carboxypeptidase | taxID used:135614 OG00693|WP\_038228799.1 | nicotinate phosphoribosyltransferase | taxID used:135614 OG00695|WP\_010894134.1 | beta-ketoacyl-[acyl-carrier-protein] synthase family protein | taxID used:135614 OG00697|WP\_020852201.1 | TraB/GumN family protein | taxID used:135614 OG00700|WP\_020851976.1 | 8-amino-7-oxononanoate synthase | taxID used:135614 OG00702|WP\_058569775.1 | GTP cyclohydrolase II RibA | taxID used:135614 OG00705|WP\_004088002.1 | MFS transporter | taxID used:135614 OG00707|WP\_038210951.1 | type II secretion system F family protein | taxID used:135614 OG00709|WP\_046419351.1 | polyketide cyclase | taxID used:135614 OG00711|WP\_031336363.1 | lipopolysaccharide assembly protein LapB | taxID used:135614 OG00713|WP\_004083737.1 | class I SAM-dependent methyltransferase | taxID used:135614 OG00716|WP\_020851944.1 | tetratricopeptide repeat protein | taxID used:135614 OG00717|WP\_031337462.1 | patatin | taxID used:135614 OG00719|WP\_046420832.1 | 5-(carboxyamino)imidazole ribonucleotide synthase | taxID used:135614 OG00722|WP\_010893494.1 | porin | taxID used:135614 OG00724|WP\_004084431.1 | Ubiquinone biosynthesis hydroxylase UbiH/UbiF/VisC/COQ6 | taxID used:135614 OG00729|WP\_004083615.1 | class I SAM-dependent rRNA methyltransferase | taxID used:135614 OG00732|WP\_010894266.1 | heme biosynthesis protein HemY | taxID used:135614 OG00733|WP\_010894376.1 | signal recognition particle-docking protein FtsY | taxID used:135614 OG00735|WP\_004089326.1 | 1-deoxy-D-xylulose-5-phosphate reductoisomerase | taxID used:135614 OG00736|WP\_004085873.1 | ABC transporter permease | taxID used:135614 OG00073|WP\_071869546.1 | DNA polymerase I | taxID used:135614 OG00742|WP\_088577634.1 | tRNA guanosine(34) transglycosylase Tgt | taxID used:135614 OG00744|WP\_027699985.1 | glycosyltransferase family 1 protein | taxID used:135614 OG00745|WP\_010894048.1 | dihydrolipoyllysine-residue succinyltransferase | taxID used:135614 OG00074|WP\_010893327.1 | protein translocase subunit SecA | taxID used:135614 OG00755|WP\_057682878.1 | cation tolerance protein CutA | taxID used:135614 OG00757|WP\_088578350.1 | LysM peptidoglycan-binding domain-containing protein | taxID used:135614 OG00075|WP\_011097554.1 | aconitate hydratase AcnA | taxID used:135614 OG00760|WP\_104993207.1 | lipid-A-disaccharide synthase | taxID used:135614 OG00765|WP\_004087889.1 | glutamate 5-kinase | taxID used:135614 OG00766|WP\_004083731.1 | N-acetylglucosamine-6-phosphate deacetylase | taxID used:135614 OG00768|WP\_088578148.1 | type IV pili twitching motility protein PilT | taxID used:135614 OG00076|WP\_057683342.1 | pyruvate dehydrogenase (acetyl-transferring), homodimeric type | taxID used:135614 OG00770|WP\_031337074.1 | phosphoglycerate kinase | taxID used:135614 OG00771|WP\_004090371.1 | succinyl-CoA ligase subunit beta | taxID used:135614 OG00774|WP\_038229914.1 | DNA-protecting protein DprA | taxID used:135614 OG00775|WP\_004083681.1 | general secretion pathway protein GspL | taxID used:135614 OG00776|WP\_010893775.1 | lipoyl synthase | taxID used:135614 OG00777|WP\_046417955.1 | OmpA family protein | taxID used:135614 OG00779|WP\_010894665.1 | bifunctional histidinol-phosphatase/imidazoleglycerol-phosphate dehydratase | taxID used:135614 OG00782|WP\_057682978.1 | succinyl-diaminopimelate desuccinylase | taxID used:135614 OG00784|WP\_010892648.1 | glycosyl transferase group 1 | taxID used:135614 OG00785|WP\_057683266.1 | energy transducer TonB | taxID used:135614 OG00786|WP\_031336924.1 | carbamoyl-phosphate synthase small subunit | taxID used:135614 OG00788|WP\_011098075.1 | Amino acid-binding ACT:Prephenate dehydrogenase | taxID used:135614 OG00790|WP\_038228106.1 | homoserine O-acetyltransferase | taxID used:135614 OG00793|WP\_038229930.1 | 3,4-dihydroxy-2-butanone-4-phosphate synthase | taxID used:135614 OG00796|WP\_071869544.1 | peptide chain release factor 2 | taxID used:135614 OG00798|WP\_088371226.1 | PLP-dependent cysteine synthase family protein | taxID used:135614 OG00007|WP\_057682684.1 | response regulator | taxID used:135614 OG00800|WP\_004086145.1 | lytic murein transglycosylase B | taxID used:135614 OG00808|WP\_010892552.1 | DNA replication and repair protein RecF | taxID used:135614 OG00809|WP\_046417730.1 | LPS export ABC transporter permease LptG | taxID used:135614 OG00814|WP\_038231772.1 | agamatine deiminase | taxID used:135614 OG00815|WP\_004083972.1 | ABC transporter permease | taxID used:135614 OG00817|WP\_010894590.1 | phosphate ABC transporter substrate-binding protein PstS | taxID used:135614 OG00081|WP\_004086138.1 | leucine--tRNA ligase | taxID used:135614 OG00821|WP\_010892575.1 | 3-deoxy-7-phosphoheptulonate synthase | taxID used:135614 OG00822|WP\_038228624.1 | ribonuclease D | taxID used:135614 OG00823|WP\_057683074.1 | AI-2E family transporter | taxID used:135614 OG00827|WP\_004088182.1 | alanine racemase | taxID used:135614 OG00829|WP\_011098070.1 | phosphoserine transaminase | taxID used:135614 OG00833|WP\_004085494.1 | branched-chain amino acid aminotransferase | taxID used:135614 OG00840|WP\_027700229.1 | cellulase | taxID used:135614 OG00843|WP\_011097599.1 | sn-glycerol-3-phosphate ABC transporter ATP-binding protein UgpC | taxID used:135614 OG00845|WP\_058569486.1 | undecaprenyldiphospho-muramoylpentapeptide beta-N-acetylglucosaminyltransferase | taxID used:135614 OG00851|WP\_004089089.1 | LacI family transcriptional regulator | taxID used:135614 OG00852|WP\_012337564.1 | LPS export ABC transporter permease LptF | taxID used:135614 OG00853|WP\_004087401.1 | peptide chain release factor 1 | taxID used:135614 OG00855|WP\_004089675.1 | bifunctional diaminohydroxyphosphoribosylaminopyrimidine deaminase/5-amino-6-(5-phosphoribosylamino)uracil reductase RibD | taxID used:135614 OG00085|WP\_010892776.1 | translation initiation factor IF-2 | taxID used:135614 OG00865|WP\_004086116.1 | lipopolysaccharide heptosyltransferase family protein | taxID used:135614 OG00867|WP\_057683309.1 | GTPase ObgE | taxID used:135614 OG00869|WP\_031336560.1 | A/G-specific adenine glycosylase | taxID used:135614 OG00873|WP\_010893837.1 | uroporphyrinogen decarboxylase | taxID used:135614 OG00875|WP\_020851248.1 | S-methyl-5-thioribose-1-phosphate isomerase | taxID used:135614 OG00876|WP\_004085791.1 | 3-isopropylmalate dehydrogenase | taxID used:135614 OG00877|WP\_104996237.1 | intercellular spreading VacJ lipoprotein | taxID used:135614 OG00885|WP\_004084645.1 | UDP-N-acetylenolpyruvoylglucosamine reductase | taxID used:135614 OG00889|WP\_004090192.1 | ribonucleotide-diphosphate reductase subunit beta | taxID used:135614 OG00088|WP\_088578321.1 | glycerol-3-phosphate 1-O-acyltransferase PlsB | taxID used:135614 OG00892|WP\_027700304.1 | phosphoribosylformylglycinamidine cyclo-ligase | taxID used:135614 OG00894|WP\_049767181.1 | endolytic transglycosylase MltG | taxID used:135614 OG00895|WP\_004085486.1 | CDP-glycerol glycerophosphotransferase family protein | taxID used:135614 OG00899|WP\_010892665.1 | right-handed parallel beta-helix repeat-containing protein | taxID used:135614 OG00900|WP\_004083513.1 | GDP-mannose 4,6-dehydratase | taxID used:135614 OG00908|WP\_020852236.1 | threonine dehydratase | taxID used:135614 OG00090|WP\_060871960.1 | bifunctional aspartate kinase/diaminopimelate decarboxylase | taxID used:135614 OG00910|WP\_042463646.1 | S-adenosylmethionine:tRNA ribosyltransferase-isomerase | taxID used:135614 OG00912|WP\_038211538.1 | biotin synthase BioB | taxID used:135614 OG00914|WP\_004085284.1 | ABC transporter substrate-binding protein | taxID used:135614 OG00916|WP\_060871962.1 | tetraacyldisaccharide 4'-kinase | taxID used:135614 OG00918|WP\_058569213.1 | aspartate-semialdehyde dehydrogenase | taxID used:135614 OG00919|WP\_004086557.1 | DUF475 domain-containing protein | taxID used:135614 OG00091|WP\_088577652.1 | bifunctional aconitate hydratase 2/2-methylisocitrate dehydratase | taxID used:135614 OG00920|WP\_004083595.1 | tRNA (adenosine(37)-N6)-threonylcarbamoyltransferase complex transferase subunit TsaD | taxID used:135614 OG00922|WP\_057683107.1 | N-acetylornithine carbamoyltransferase | taxID used:135614 OG00924|WP\_038228019.1 | ribosome small subunit-dependent GTPase A | taxID used:135614 OG00925|WP\_071869658.1 | rod shape-determining protein | taxID used:135614 OG00926|WP\_046417698.1 | DNA recombination/repair protein RecA | taxID used:135614 OG00928|WP\_023906078.1 | anthranilate phosphoribosyltransferase | taxID used:135614 OG00092|WP\_046419728.1 | bifunctional lysylphosphatidylglycerol flippase/synthetase MprF | taxID used:135614 OG00930|WP\_004084959.1 | sensor histidine kinase | taxID used:135614 OG00933|WP\_027700744.1 | glycerol-3-phosphate dehydrogenase | taxID used:135614 OG00934|WP\_058569358.1 | magnesium and cobalt transport protein CorA | taxID used:135614 OG00935|WP\_020851363.1 | DNA polymerase III subunit delta | taxID used:135614 OG00946|WP\_027700281.1 | EF-P beta-lysylation protein EpmB | taxID used:135614 OG00949|WP\_004085423.1 | Holliday junction branch migration DNA helicase RuvB | taxID used:135614 OG00094|WP\_088578586.1 | DNA mismatch repair protein MutS | taxID used:135614 OG00952|WP\_088577873.1 | SIS domain-containing protein | taxID used:135614 OG00953|WP\_004083996.1 | SMP-30/gluconolactonase/LRE family protein | taxID used:135614 OG00955|WP\_012382741.1 | homoserine acetyltransferase | taxID used:135614 OG00957|WP\_057683427.1 | glucokinase | taxID used:135614 OG00959|WP\_004085216.1 | UDP-3-O-(3-hydroxymyristoyl)glucosamine N-acyltransferase | taxID used:135614 OG00960|WP\_004085471.1 | 3-oxoacyl-ACP synthase III | taxID used:135614 OG00961|WP\_004085533.1 | nitronate monooxygenase | taxID used:135614 OG00963|WP\_031336169.1 | glycosyltransferase | taxID used:135614 OG00965|WP\_058569918.1 | porphobilinogen synthase | taxID used:135614 OG00966|WP\_004084873.1 | ketol-acid reductoisomerase | taxID used:135614 OG00967|WP\_023906581.1 | 3-beta hydroxysteroid dehydrogenase/isomerase | taxID used:135614 OG00968|WP\_058569478.1 | fructose-bisphosphate aldolase class I | taxID used:135614 OG00970|WP\_020851191.1 | type I glyceraldehyde-3-phosphate dehydrogenase | taxID used:135614 OG00972|WP\_038229855.1 | glucokinase | taxID used:135614 OG00973|WP\_038228746.1 | phenylalanine--tRNA ligase subunit alpha | taxID used:135614 OG00975|WP\_004084859.1 | nucleoside-diphosphate sugar epimerase | taxID used:135614 OG00978|WP\_031337050.1 | methionine ABC transporter ATP-binding protein | taxID used:135614 OG00979|WP\_004083792.1 | trans-hexaprenyltranstransferase | taxID used:135614 OG00982|WP\_023906989.1 | pteridine-dependent deoxygenase | taxID used:135614 OG00984|WP\_004087529.1 | NAD-dependent isocitrate dehydrogenase | taxID used:135614 OG00990|WP\_027700003.1 | KpsF/GutQ family sugar-phosphate isomerase | taxID used:135614 OG00994|WP\_057682775.1 | oxidoreductase | taxID used:135614 OG00997|WP\_071869668.1 | LLM class flavin-dependent oxidoreductase | taxID used:135614 OG00998|WP\_038228905.1 | malate dehydrogenase | taxID used:135614 OG00999|WP\_031337572.1 | ferrochelatase | taxID used:135614 OG00009|WP\_038274152.1 | alpha-2-macroglobulin family protein | taxID used:135614 
OG01023|WP\_020852120.1 | HrgA protein | taxID used:135614 OG01030|WP\_038231476.1 | phage portal protein, partial | taxID used:135614 OG01065|WP\_004085608.1 | hypothetical protein | taxID used:135614 OG01092|WP\_081089776.1 | phage tail protein | taxID used:135614 OG01094|WP\_038230048.1 | 4-hydroxybenzoate octaprenyltransferase | taxID used:135614 OG01106|WP\_004085607.1 | DUF769 domain-containing protein | taxID used:135614 OG01148|WP\_024749292.1 | acetyltransferase | taxID used:135614 OG01151|WP\_046420007.1 | DNA methyltransferase | taxID used:135614 OG01164|WP\_011097886.1 | hypothetical protein | taxID used:135614 OG01186|WP\_011098250.1 | hypothetical protein | taxID used:135614 OG01194|WP\_010893410.1 | hypothetical protein | taxID used:135614 OG00011|WP\_088572685.1 | hemagglutinin | taxID used:135614 OG01215|WP\_010894234.1 | Nin-like protein | taxID used:135614 OG01427|WP\_010894250.1 | TIGR03761 family integrating conjugative element protein | taxID used:135614 OG01445|WP\_020852701.1 | hypothetical protein | taxID used:135614 OG01455|WP\_053014151.1 | KilA-N domain-containing protein | taxID used:135614 OG00014|WP\_060871689.1 | DNA primase | taxID used:135614 OG01503|WP\_004088676.1 | DNA-binding protein | taxID used:135614 OG01514|WP\_023906440.1 | hypothetical protein | taxID used:135614 OG01517|WP\_038211144.1 | HNH endonuclease | taxID used:135614 OG01557|WP\_027700691.1 | DUF3426 domain-containing protein | taxID used:135614 OG01571|WP\_109161033.1 | hypothetical protein | taxID used:135614 OG01574|WP\_058565223.1 | UDP-3-O-(3-hydroxymyristoyl)glucosamine N-acyltransferase | taxID used:135614 OG01593|WP\_060871681.1 | hypothetical protein | taxID used:135614 OG01611|WP\_010895154.1 | hypothetical protein | taxID used:135614 OG01619|WP\_081089792.1 | phage tail protein | taxID used:135614 OG01621|WP\_081089770.1 | phage tail protein | taxID used:135614 OG01650|WP\_058565219.1 | phage-related protein | taxID used:135614 OG01651|WP\_076613276.1 | hypothetical protein | taxID used:135614 OG01653|WP\_023906644.1 | hypothetical protein | taxID used:135614 OG01654|WP\_038232805.1 | phage portal protein | taxID used:135614 OG01704|WP\_038283980.1 | MerR family DNA-binding transcriptional regulator | taxID used:135614 OG01719|WP\_071869955.1 | hypothetical protein | taxID used:135614 OG01737|WP\_088577852.1 | phage tail protein | taxID used:135614 OG01749|WP\_060871444.1 | hypothetical protein | taxID used:135614 OG01844|WP\_004086586.1 | phage-related protein | taxID used:135614 OG01845|WP\_088371557.1 | DUF2612 domain-containing protein | taxID used:135614 OG01855|WP\_060871828.1 | hypothetical protein | taxID used:135614 OG01869|WP\_104993163.1 | pilin | taxID used:135614 OG01877|WP\_046419988.1 | prepilin-type cleavage/methylation domain-containing protein | taxID used:135614 OG01880|WP\_010893754.1 | hypothetical protein | taxID used:135614 OG01886|WP\_076613614.1 | hypothetical protein | taxID used:135614 OG01888|WP\_060872403.1 | hypothetical protein | taxID used:135614 OG01912|WP\_004087359.1 | pilin | taxID used:135614 OG01917|WP\_060872105.1 | DNA-packaging protein | taxID used:135614 OG01940|WP\_010894249.1 | DUF3158 domain-containing protein | taxID used:135614 OG01942|WP\_004083639.1 | site-specific DNA-methyltransferase | taxID used:135614 OG01963|WP\_004086964.1 | hypothetical protein | taxID used:135614 OG02004|WP\_024748582.1 | hypothetical protein | taxID used:135614 OG02012|WP\_027700433.1 | phage major tail tube protein | taxID used:135614 OG02014|WP\_038229184.1 | hypothetical protein | taxID used:135614 OG02015|WP\_060872416.1 | phage major tail tube protein | taxID used:135614 OG02030|WP\_010893055.1 | hypothetical protein | taxID used:135614 OG02055|WP\_057682796.1 | DUF4411 domain-containing protein | taxID used:135614 OG02060|WP\_080679633.1 | DUF5131 domain-containing protein | taxID used:135614 OG02070|WP\_004088370.1 | phage tail protein | taxID used:135614 OG02084|WP\_040123074.1 | hypothetical protein | taxID used:135614 OG02106|WP\_088572654.1 | phage tail protein | taxID used:135614 OG02110|WP\_038210550.1 | RTX toxin | taxID used:135614 OG02135|WP\_060872426.1 | DUF1566 domain-containing protein | taxID used:135614 OG00216|WP\_057683755.1 | phage terminase large subunit family protein | taxID used:135614 OG00217|WP\_004086889.1 | phage terminase large subunit family protein | taxID used:135614 OG00220|WP\_023908213.1 | conjugal transfer protein TraG | taxID used:135614 OG02239|WP\_031336412.1 | hypothetical protein | taxID used:135614 OG02256|WP\_060870416.1 | hypothetical protein | taxID used:135614 OG02262|WP\_011098299.1 | YeeE/YedE family protein | taxID used:135614 OG02316|WP\_118853448.1 | hypothetical protein, partial | taxID used:135614 OG02325|WP\_023908221.1 | putative toxin-antitoxin system toxin component, PIN family | taxID used:135614 OG02381|WP\_010893418.1 | hypothetical protein | taxID used:135614 OG02466|WP\_011097945.1 | phage antirepressor | taxID used:135614 OG02469|WP\_012382625.1 | phage antirepressor protein | taxID used:135614 OG02475|WP\_014607382.1 | hypothetical protein | taxID used:135614 OG00249|WP\_060872412.1 | phage major capsid protein | taxID used:135614 OG02501|WP\_075584619.1 | hypothetical protein | taxID used:135614 OG02517|WP\_080939537.1 | hypothetical protein | taxID used:135614 OG00252|WP\_038210487.1 | phage major capsid protein | taxID used:135614 OG00256|WP\_010894224.1 | integrating conjugative element relaxase | taxID used:135614 OG02600|WP\_004084535.1 | ArsR family transcriptional regulator | taxID used:135614 OG02621|WP\_042462902.1 | hypothetical protein | taxID used:135614 OG02626|WP\_080679716.1 | hypothetical protein | taxID used:135614 OG02651|WP\_038230621.1 | phage portal protein, partial | taxID used:135614 OG02679|WP\_004085001.1 | hypothetical protein | taxID used:135614 OG00267|WP\_126709114.1 | phage major capsid protein | taxID used:135614 OG02708|WP\_126715044.1 | hypothetical protein | taxID used:135614 OG02742|WP\_038200376.1 | membrane protein | taxID used:135614 OG02755|WP\_011097940.1 | hypothetical protein | taxID used:135614 OG02768|WP\_046419179.1 | hypothetical protein | taxID used:135614 OG02773|WP\_023907295.1 | hypothetical protein | taxID used:135614 OG02774|WP\_038210573.1 | hypothetical protein | taxID used:135614 OG02781|WP\_004572790.1 | DNA methyltransferase | taxID used:135614 OG00282|WP\_004087780.1 | DUF2326 domain-containing protein | taxID used:135614 OG02916|WP\_057683748.1 | phage tail assembly protein | taxID used:135614 OG02918|WP\_010893253.1 | phage tail assembly protein | taxID used:135614 OG02933|WP\_060870090.1 | hypothetical protein | taxID used:135614 OG02958|WP\_004091326.1 | hypothetical protein | taxID used:135614 OG02994|WP\_023908223.1 | DUF1778 domain-containing protein | taxID used:135614 OG03002|WP\_010894337.1 | hypothetical protein | taxID used:135614 OG00301|WP\_071869704.1 | sulfonate ABC transporter permease | taxID used:135614 OG03024|WP\_051606224.1 | hypothetical protein | taxID used:135614 OG03040|WP\_076613308.1 | phage major tail tube protein, partial | taxID used:135614 OG03041|WP\_076613309.1 | phage major tail tube protein, partial | taxID used:135614 OG03092|WP\_042464021.1 | XRE family transcriptional regulator | taxID used:135614 OG03180|WP\_020852953.1 | hypothetical protein | taxID used:135614 OG03184|WP\_050765464.1 | hypothetical protein | taxID used:135614 OG03204|WP\_020851102.1 | methionine repressor-like protein | taxID used:135614 OG03225|WP\_021358193.1 | hypothetical protein | taxID used:135614 OG03264|WP\_058569894.1 | hypothetical protein | taxID used:135614 OG03282|WP\_014607805.1 | DUF3383 domain-containing protein | taxID used:135614 OG03300|WP\_046419240.1 | DUF3018 domain-containing protein | taxID used:135614 OG03323|WP\_081046879.1 | hypothetical protein | taxID used:135614 OG03353|WP\_060870204.1 | phage tail protein | taxID used:135614 OG03389|WP\_021358299.1 | hypothetical protein | taxID used:135614 OG00341|WP\_011097937.1 | phage portal protein | taxID used:135614 OG00342|WP\_010893237.1 | phage portal protein | taxID used:135614 OG03442|WP\_058564340.1 | hypothetical protein | taxID used:135614 OG03570|WP\_081089752.1 | hypothetical protein | taxID used:135614 OG00035|WP\_004087437.1 | ATP-dependent exonuclease SbcCD, C subunit-like protein | taxID used:135614 OG03644|WP\_076613566.1 | lysozyme, partial | taxID used:135614 OG03648|WP\_081033553.1 | methyltransferase | taxID used:135614 OG00424|WP\_080939574.1 | DNA packaging protein | taxID used:135614 OG00428|WP\_027700587.1 | DNA packaging protein | taxID used:135614 OG00446|WP\_088371998.1 | restriction endonuclease subunit S | taxID used:135614 OG00047|WP\_088372018.1 | type I restriction endonuclease subunit R | taxID used:135614 OG00573|WP\_010892634.1 | alpha-ketoglutarate permease | taxID used:135614 OG00622|WP\_023906211.1 | DUF4102 domain-containing protein | taxID used:135614 OG00637|WP\_118853428.1 | phage major capsid protein | taxID used:135614 OG00648|WP\_023908151.1 | restriction endonuclease subunit S | taxID used:135614 OG00669|WP\_118853427.1 | phage major capsid protein | taxID used:135614 OG00737|WP\_076613263.1 | site-specific integrase | taxID used:135614 OG00767|WP\_088578043.1 | phage-related protein | taxID used:135614 OG00837|WP\_027700429.1 | phage late control D | taxID used:135614 OG00841|WP\_011097927.1 | phage tail protein | taxID used:135614 OG00847|WP\_060870461.1 | phage tail protein | taxID used:135614 OG00891|WP\_010894205.1 | NAD(P)-dependent alcohol dehydrogenase | taxID used:135614 OG00896|WP\_004091498.1 | P-type conjugative transfer ATPase TrbB | taxID used:135614 OG00923|WP\_004087145.1 | ParB/RepB/Spo0J family partition protein | taxID used:135614 OG00927|WP\_023907561.1 | membrane protein | taxID used:135614 OG00956|WP\_060872001.1 | phage major capsid protein, partial | taxID used:135614 OG00096|WP\_046417530.1 | conjugal transfer protein TrbE | taxID used:135614 OG01965|WP\_050765457.1 | DNA-packaging protein | taxID used:135614 OG02639|WP\_011097935.1 | hypothetical protein | taxID used:135614 OG02757|WP\_038231493.1 | hypothetical protein | taxID used:135614 OG01000|WP\_011097726.1 | ubiquinol oxidase subunit II | taxID used:135614 OG01001|WP\_004084423.1 | 4-hydroxythreonine-4-phosphate dehydrogenase PdxA | taxID used:135614 OG01002|WP\_031336621.1 | uroporphyrin-III C-methyltransferase | taxID used:135614 OG01003|WP\_058564605.1 | acyltransferase | taxID used:135614 OG01004|WP\_004083763.1 | site-specific tyrosine recombinase XerD | taxID used:135614 OG01005|WP\_020852040.1 | RluA family pseudouridine synthase | taxID used:135614 OG01006|WP\_010893424.1 | PhoH family protein | taxID used:135614 OG01007|WP\_004090142.1 | DNA-directed RNA polymerase subunit alpha | taxID used:135614 OG01008|WP\_023908084.1 | 16S rRNA (cytosine(1402)-N(4))-methyltransferase RsmH | taxID used:135614 OG01009|WP\_010894223.1 | LysR family transcriptional regulator | taxID used:135614 OG01011|WP\_080654456.1 | AcrB/AcrD/AcrF family protein | taxID used:135614 OG01012|WP\_046418737.1 | 23S rRNA pseudouridine(1911/1915/1917) synthase RluD | taxID used:135614 OG01013|WP\_023906990.1 | DUF1684 domain-containing protein | taxID used:135614 OG01014|WP\_020851606.1 | thiamine-phosphate kinase | taxID used:135614 OG01016|WP\_058564676.1 | LysR family transcriptional regulator | taxID used:135614 OG01017|WP\_024748785.1 | DNA polymerase III subunit delta' | taxID used:135614 OG01018|WP\_023907647.1 | DUF58 domain-containing protein | taxID used:135614 OG01019|WP\_004086600.1 | aldo/keto reductase | taxID used:135614 OG01020|WP\_004085451.1 | SPOR domain-containing protein | taxID used:135614 OG01022|WP\_027700024.1 | lipid A biosynthesis lauroyl acyltransferase | taxID used:135614 OG01024|WP\_023907715.1 | prolyl aminopeptidase | taxID used:135614 OG01025|WP\_004083746.1 | thioredoxin-disulfide reductase | taxID used:135614 OG01026|WP\_088371850.1 | tellurium resistance protein TerC | taxID used:135614 OG01027|WP\_004090037.1 | NUDIX domain-containing protein | taxID used:135614 OG01028|WP\_080939527.1 | P-type conjugative transfer ATPase TrbB | taxID used:135614 OG01029|WP\_004084878.1 | ketoacyl-ACP synthase III | taxID used:135614 OG00102|WP\_010892912.1 | ATP-dependent chaperone ClpB | taxID used:135614 OG01031|WP\_038211447.1 | tRNA (adenosine(37)-N6)-dimethylallyltransferase MiaA | taxID used:135614 OG01032|WP\_004083511.1 | NAD-dependent epimerase/dehydratase family protein | taxID used:135614 OG01034|WP\_004084779.1 | hypothetical protein | taxID used:135614 OG01035|WP\_010894591.1 | phosphate ABC transporter permease subunit PstC | taxID used:135614 OG01036|WP\_012337572.1 | acetyl-CoA carboxylase carboxyl transferase subunit alpha | taxID used:135614 OG01037|WP\_057682909.1 | MBL fold hydrolase | taxID used:135614 OG01038|WP\_060872294.1 | 5'-nucleotidase | taxID used:135614 OG01039|WP\_031337080.1 | M23 family peptidase | taxID used:135614 OG00103|WP\_010893707.1 | ribonucleoside-diphosphate reductase subunit alpha | taxID used:135614 OG01040|WP\_023907069.1 | EF-P lysine aminoacylase GenX | taxID used:135614 OG01041|WP\_004083864.1 | sulfate ABC transporter permease subunit CysW | taxID used:135614 OG01042|WP\_004572963.1 | protein translocase subunit SecF | taxID used:135614 OG01043|WP\_004090506.1 | D-alanine--D-alanine ligase | taxID used:135614 OG01044|WP\_010893454.1 | DNA adenine methylase | taxID used:135614 OG01045|WP\_012337991.1 | 4-hydroxy-3-methylbut-2-enyl diphosphate reductase | taxID used:135614 OG01046|WP\_057682432.1 | cysteine synthase A | taxID used:135614 OG01047|WP\_004085042.1 | SPFH/Band 7/PHB domain protein | taxID used:135614 OG01048|WP\_020851934.1 | pilus assembly protein PilW | taxID used:135614 OG01049|WP\_057683630.1 | phage recombination protein Bet | taxID used:135614 OG00104|WP\_046420174.1 | helicase SNF2 | taxID used:135614 OG01051|WP\_004083414.1 | homoserine kinase | taxID used:135614 OG01052|WP\_024748796.1 | ribose-phosphate diphosphokinase | taxID used:135614 OG01053|WP\_004085460.1 | glutathione synthase | taxID used:135614 OG01054|WP\_031336446.1 | aspartate carbamoyltransferase catalytic subunit | taxID used:135614 OG01055|WP\_004084986.1 | FKBP-type peptidyl-prolyl cis-trans isomerase | taxID used:135614 OG01056|WP\_004086320.1 | peptidase | taxID used:135614 OG01057|WP\_004083510.1 | dolichol-phosphate mannosyltransferase | taxID used:135614 OG01058|WP\_011098115.1 | bifunctional riboflavin kinase/FAD synthetase | taxID used:135614 OG01059|WP\_004083779.1 | HPr kinase/phosphorylase | taxID used:135614 OG00105|WP\_088578458.1 | S9 family peptidase | taxID used:135614 OG01060|WP\_038210604.1 | EamA/RhaT family transporter | taxID used:135614 OG01061|WP\_046418483.1 | virulence factor family protein | taxID used:135614 OG01062|WP\_057683341.1 | [acyl-carrier-protein] S-malonyltransferase | taxID used:135614 OG01063|WP\_057682734.1 | ribokinase | taxID used:135614 OG01066|WP\_020851797.1 | LpxL/LpxP family Kdo(2)-lipid IV(A) lauroyl/palmitoleoyl acyltransferase | taxID used:135614 OG01067|WP\_004085463.1 | glycine--tRNA ligase subunit alpha | taxID used:135614 OG01068|WP\_023908118.1 | EamA/RhaT family transporter | taxID used:135614 OG01069|WP\_010892566.1 | coproporphyrinogen III oxidase | taxID used:135614 OG00106|WP\_080939564.1 | hypothetical protein | taxID used:135614 OG01071|WP\_058564428.1 | electron transfer flavoprotein subunit alpha/FixB family protein | taxID used:135614 OG01072|WP\_057683103.1 | RNA polymerase-binding protein DksA | taxID used:135614 OG01073|WP\_004086760.1 | lipid A hydroxylase LpxO | taxID used:135614 OG01074|WP\_010893900.1 | methyltransferase domain-containing protein | taxID used:135614 OG01075|WP\_088578037.1 | bifunctional biotin--[acetyl-CoA-carboxylase] ligase/biotin operon repressor BirA | taxID used:135614 OG01076|WP\_004083674.1 | LysR family transcriptional regulator | taxID used:135614 OG01077|WP\_023906075.1 | phosphoribosylaminoimidazolesuccinocarboxamide synthase | taxID used:135614 OG01078|WP\_010893816.1 | rod shape-determining protein MreC | taxID used:135614 OG01079|WP\_031337764.1 | alpha/beta hydrolase | taxID used:135614 OG00107|WP\_023907202.1 | phosphomannomutase/phosphoglucomutase | taxID used:135614 OG01080|WP\_038230604.1 | integrase | taxID used:135614 OG01081|WP\_010894280.1 | MoxR family ATPase | taxID used:135614 OG01082|WP\_004086576.1 | ABC transporter ATP-binding protein | taxID used:135614 OG01083|WP\_024748769.1 | sulfate adenylyltransferase subunit CysD | taxID used:135614 OG01084|WP\_050812599.1 | hypothetical protein | taxID used:135614 OG01085|WP\_058564505.1 | phosphoglycerate mutase | taxID used:135614 OG01086|WP\_004089711.1 | peptidase | taxID used:135614 OG01087|WP\_004083837.1 | 50S ribosomal protein L3 N(5)-glutamine methyltransferase | taxID used:135614 OG01088|WP\_058564347.1 | ABC transporter permease | taxID used:135614 OG01089|WP\_038228385.1 | prolipoprotein diacylglyceryl transferase | taxID used:135614 OG00108|WP\_040123247.1 | penicillin-binding protein 1A | taxID used:135614 OG01090|WP\_020851304.1 | Grx4 family monothiol glutaredoxin | taxID used:135614 OG01091|WP\_004087323.1 | SAM-dependent methyltransferase | taxID used:135614 OG01093|WP\_027700312.1 | GDP-6-deoxy-D-lyxo-4-hexulose reductase | taxID used:135614 OG01095|WP\_038228650.1 | UDP-3-O-[3-hydroxymyristoyl] N-acetylglucosamine deacetylase | taxID used:135614 OG01096|WP\_004086445.1 | methionyl-tRNA formyltransferase | taxID used:135614 OG01097|WP\_088371824.1 | DUF1566 domain-containing protein | taxID used:135614 OG01098|WP\_020850906.1 | ion transporter | taxID used:135614 OG01099|WP\_004083985.1 | MCE family protein | taxID used:135614 OG00010|WP\_080703215.1 | calcium-binding protein | taxID used:135614 OG01100|WP\_010893095.1 | tRNA 2-thiocytidine(32) synthetase TtcA | taxID used:135614 OG01101|WP\_004086228.1 | dTDP-4-dehydrorhamnose reductase | taxID used:135614 OG01102|WP\_023907505.1 | ATP phosphoribosyltransferase | taxID used:135614 OG01103|WP\_004088776.1 | hypothetical protein | taxID used:135614 OG01105|WP\_021358444.1 | hypothetical protein | taxID used:135614 OG01107|WP\_031345833.1 | chromosome partitioning protein ParB | taxID used:135614 OG01108|WP\_004090646.1 | protoheme IX farnesyltransferase | taxID used:135614 OG01109|WP\_020851126.1 | GTP cyclohydrolase I FolE2 | taxID used:135614 OG01110|WP\_004087373.1 | mechanosensitive ion channel protein MscS | taxID used:135614 OG01111|WP\_088577935.1 | hypothetical protein | taxID used:135614 OG01112|WP\_010894015.1 | AEC family transporter | taxID used:135614 OG01113|WP\_023906817.1 | LysR family transcriptional regulator | taxID used:135614 OG01114|WP\_088372240.1 | membrane protein | taxID used:135614 OG01115|WP\_004087631.1 | 2,3,4,5-tetrahydropyridine-2,6-dicarboxylate N-succinyltransferase | taxID used:135614 OG01116|WP\_004089081.1 | acetyl-CoA carboxylase carboxyltransferase subunit beta | taxID used:135614 OG01117|WP\_004083393.1 | 50S ribosomal protein L11 methyltransferase | taxID used:135614 OG01118|WP\_004088630.1 | bis(5'-nucleosyl)-tetraphosphatase (symmetrical) | taxID used:135614 OG01119|WP\_004086003.1 | segregation/condensation protein A | taxID used:135614 OG00111|WP\_038229293.1 | ribonuclease R | taxID used:135614 OG01120|WP\_004088756.1 | recombination-associated protein RdgC | taxID used:135614 OG01122|WP\_038228620.1 | alpha-L-glutamate ligase | taxID used:135614 OG01123|WP\_046419711.1 | hydroxymethylbilane synthase | taxID used:135614 OG01126|WP\_011098000.1 | ribosomal RNA small subunit methyltransferase A | taxID used:135614 OG01127|WP\_004084369.1 | cation transporter | taxID used:135614 OG01128|WP\_020851593.1 | outer membrane protein assembly factor BamD | taxID used:135614 OG01129|WP\_071870040.1 | P-type conjugative transfer protein TrbG | taxID used:135614 OG01132|WP\_010894506.1 | P-type conjugative transfer protein TrbG | taxID used:135614 OG01133|WP\_071869898.1 | TIGR01777 family protein | taxID used:135614 OG01134|WP\_023907570.1 | glycosyltransferase family 2 protein | taxID used:135614 OG01135|WP\_046418189.1 | nitrilase/cyanide hydratase | taxID used:135614 OG01136|WP\_057683465.1 | glycosyltransferase family 2 protein | taxID used:135614 OG01137|WP\_020852274.1 | ligand-binding protein SH3 | taxID used:135614 OG01138|WP\_038229140.1 | YicC family protein | taxID used:135614 OG00113|WP\_088578275.1 | ATP-dependent helicase HrpB | taxID used:135614 OG01140|WP\_021358338.1 | energy transducer TonB | taxID used:135614 OG01141|WP\_010895130.1 | ketoreductase | taxID used:135614 OG01143|WP\_004086758.1 | malonyl-[acyl-carrier protein] O-methyltransferase BioC | taxID used:135614 OG01145|WP\_010894695.1 | GTPase Era | taxID used:135614 OG01146|WP\_031336539.1 | membrane protein | taxID used:135614 OG01147|WP\_071869637.1 | 4-hydroxy-tetrahydrodipicolinate synthase | taxID used:135614 OG01149|WP\_020851570.1 | transglycosylase | taxID used:135614 OG00114|WP\_046419574.1 | DUF1631 domain-containing protein | taxID used:135614 OG01150|WP\_010892796.1 | glucose-1-phosphate thymidylyltransferase | taxID used:135614 OG01152|WP\_023906245.1 | sugar ABC transporter permease | taxID used:135614 OG01153|WP\_012337702.1 | methylisocitrate lyase | taxID used:135614 OG01155|WP\_060872408.1 | hypothetical protein | taxID used:135614 OG01156|WP\_004087031.1 | baseplate assembly protein J | taxID used:135614 OG01158|WP\_023907818.1 | phosphatidylserine decarboxylase | taxID used:135614 OG00115|WP\_088578690.1 | conjugal transfer protein TrbI | taxID used:135614 OG01160|WP\_004085665.1 | enoyl-CoA hydratase | taxID used:135614 OG01161|WP\_004087397.1 | 4-(cytidine 5'-diphospho)-2-C-methyl-D-erythritol kinase | taxID used:135614 OG01162|WP\_004083728.1 | polysaccharide deacetylase | taxID used:135614 OG01165|WP\_024749074.1 | HlyC/CorC family transporter | taxID used:135614 OG01166|WP\_004085962.1 | UTP--glucose-1-phosphate uridylyltransferase GalU | taxID used:135614 OG01167|WP\_012337957.1 | D-hexose-6-phosphate mutarotase | taxID used:135614 OG01168|WP\_010892638.1 | dihydropteroate synthase | taxID used:135614 OG01169|WP\_024749281.1 | tyrosine recombinase XerC | taxID used:135614 OG00116|WP\_058564307.1 | NAD-dependent DNA ligase LigA | taxID used:135614 OG01170|WP\_058569630.1 | M23 family peptidase | taxID used:135614 OG01171|WP\_057682827.1 | 23S rRNA (adenine(2030)-N(6))-methyltransferase RlmJ | taxID used:135614 OG01172|WP\_004083646.1 | hypothetical protein | taxID used:135614 OG01173|WP\_020851810.1 | polyamine aminopropyltransferase | taxID used:135614 OG01175|WP\_027700580.1 | iron-sulfur cluster carrier protein ApbC | taxID used:135614 OG01176|WP\_031336187.1 | tRNA pseudouridine(55) synthase TruB | taxID used:135614 OG01177|WP\_057682936.1 | cytochrome c biogenesis protein | taxID used:135614 OG01178|WP\_040123073.1 | baseplate assembly protein J | taxID used:135614 OG01179|WP\_038229813.1 | RNase adapter RapZ | taxID used:135614 OG00117|WP\_004088308.1 | bifunctional aspartate kinase/homoserine dehydrogenase I | taxID used:135614 OG01181|WP\_004572845.1 | hypothetical protein | taxID used:135614 OG01185|WP\_004084990.1 | ABC transporter ATP-binding protein | taxID used:135614 OG01189|WP\_031336697.1 | DUF1376 domain-containing protein | taxID used:135614 OG00118|WP\_020851724.1 | beta-N-acetylhexosaminidase | taxID used:135614 OG01190|WP\_046418125.1 | NlpC/P60 family protein | taxID used:135614 OG01191|WP\_046418909.1 | acyl-CoA thioesterase II | taxID used:135614 OG01192|WP\_010893661.1 | F0F1 ATP synthase subunit gamma | taxID used:135614 OG01193|WP\_046419211.1 | hypothetical protein | taxID used:135614 OG01199|WP\_027700202.1 | YihY/virulence factor BrkB family protein | taxID used:135614 OG00119|WP\_071869475.1 | DNA internalization-related competence protein ComEC/Rec2 | taxID used:135614 OG01200|WP\_010894200.1 | aldo/keto reductase | taxID used:135614 OG01201|WP\_057683202.1 | formyltetrahydrofolate deformylase | taxID used:135614 OG01203|WP\_088577737.1 | geranyl transferase | taxID used:135614 OG01204|WP\_011098187.1 | helix-turn-helix domain-containing protein | taxID used:135614 OG01205|WP\_046418458.1 | succinate--CoA ligase subunit alpha | taxID used:135614 OG01207|WP\_104993199.1 | RNA polymerase sigma factor RpoH | taxID used:135614 OG01208|WP\_031336365.1 | bifunctional methylenetetrahydrofolate dehydrogenase/methenyltetrahydrofolate cyclohydrolase FolD | taxID used:135614 OG01209|WP\_004084594.1 | prepilin peptidase | taxID used:135614 OG00120|WP\_004083638.1 | VWA domain-containing protein | taxID used:135614 OG01210|WP\_046418088.1 | DUF3108 domain-containing protein | taxID used:135614 OG01211|WP\_023908120.1 | elongation factor Ts | taxID used:135614 OG01212|WP\_023906622.1 | Hsp33 protein | taxID used:135614 OG01213|WP\_060870441.1 | integrase | taxID used:135614 OG01217|WP\_004084048.1 | AraC family transcriptional regulator | taxID used:135614 OG01218|WP\_046417830.1 | SDR family oxidoreductase | taxID used:135614 OG01219|WP\_046419544.1 | diaminopimelate epimerase | taxID used:135614 OG00121|WP\_010895204.1 | VirB4 family type IV secretion/conjugal transfer ATPase | taxID used:135614 OG01220|WP\_038211649.1 | hypothetical protein | taxID used:135614 OG01222|WP\_012337824.1 | nicotinate-nucleotide diphosphorylase (carboxylating) | taxID used:135614 OG01223|WP\_081046843.1 | DUF2145 domain-containing protein | taxID used:135614 OG01224|WP\_031337079.1 | SAM-dependent methyltransferase | taxID used:135614 OG01225|WP\_010894254.1 | ParA family protein | taxID used:135614 OG01226|WP\_118853429.1 | hypothetical protein, partial | taxID used:135614 OG01227|WP\_031336669.1 | hypothetical protein | taxID used:135614 OG01228|WP\_004090288.1 | phage head morphogenesis protein, SPP1 gp7 | taxID used:135614 OG01229|WP\_010895042.1 | protease HtpX | taxID used:135614 OG00122|WP\_010893049.1 | hypothetical protein | taxID used:135614 OG01230|WP\_004089788.1 | protease modulator HflC | taxID used:135614 OG01231|WP\_004086094.1 | phosphate ABC transporter permease | taxID used:135614 OG01232|WP\_021358437.1 | DUF481 domain-containing protein | taxID used:135614 OG01233|WP\_023907981.1 | phage head morphogenesis protein | taxID used:135614 OG01234|WP\_023907952.1 | M23 family metallopeptidase | taxID used:135614 OG01236|WP\_004086273.1 | pantoate--beta-alanine ligase | taxID used:135614 OG01237|WP\_020851979.1 | MBL fold metallo-hydrolase | taxID used:135614 OG01238|WP\_031336480.1 | co-chaperone YbbN | taxID used:135614 OG00123|WP\_046418721.1 | DNA topoisomerase 1 | taxID used:135614 OG01240|WP\_010893849.1 | sulfate ABC transporter permease subunit CysT | taxID used:135614 OG01241|WP\_023906259.1 | polysaccharide deacetylase family protein | taxID used:135614 OG01242|WP\_038211122.1 | pyrroline-5-carboxylate reductase | taxID used:135614 OG01243|WP\_004083708.1 | sirC regulator SirB | taxID used:135614 OG01244|WP\_046418940.1 | phosphatidate cytidylyltransferase | taxID used:135614 OG01245|WP\_058564409.1 | nucleoside triphosphate pyrophosphohydrolase | taxID used:135614 OG01247|WP\_021358442.1 | glycosyltransferase family 2 protein | taxID used:135614 OG01248|WP\_004086387.1 | phage antirepressor | taxID used:135614 OG01249|WP\_046418119.1 | shikimate dehydrogenase | taxID used:135614 OG00124|WP\_088371291.1 | endopeptidase La | taxID used:135614 OG01250|WP\_010894024.1 | general secretion pathway protein GspK | taxID used:135614 OG01252|WP\_023906820.1 | methylenetetrahydrofolate reductase [NAD(P)H] | taxID used:135614 OG01253|WP\_058569765.1 | NADPH-dependent 7-cyano-7-deazaguanine reductase QueF | taxID used:135614 OG01254|WP\_010894890.1 | carbohydrate ABC transporter permease | taxID used:135614 OG01255|WP\_088577995.1 | hydrolase TatD | taxID used:135614 OG01256|WP\_004088277.1 | aquaporin family protein | taxID used:135614 OG01257|WP\_010892701.1 | peptidase C1 | taxID used:135614 OG01259|WP\_024749083.1 | phage recombination protein Bet | taxID used:135614 OG00125|WP\_046417588.1 | S9 family peptidase | taxID used:135614 OG01260|WP\_011098196.1 | site-specific DNA-methyltransferase | taxID used:135614 OG01261|WP\_038230072.1 | phosphate ABC transporter ATP-binding protein PstB | taxID used:135614 OG01262|WP\_038211286.1 | cell division protein FtsQ/DivIB | taxID used:135614 OG01264|WP\_004090098.1 | 50S ribosomal protein L2 | taxID used:135614 OG01265|WP\_004084787.1 | response regulator | taxID used:135614 OG01266|WP\_010895209.1 | virB8 family protein | taxID used:135614 OG01267|WP\_046419672.1 | hypothetical protein | taxID used:135614 OG01268|WP\_004086033.1 | inositol monophosphatase | taxID used:135614 OG01269|WP\_010895210.1 | P-type conjugative transfer protein VirB9 | taxID used:135614 OG00126|WP\_058569803.1 | DNA topoisomerase (ATP-hydrolyzing) subunit B | taxID used:135614 OG01271|WP\_004085000.1 | hypothetical protein | taxID used:135614 OG01272|WP\_010894044.1 | tRNA threonylcarbamoyladenosine dehydratase | taxID used:135614 OG01273|WP\_126715058.1 | autotransporter domain-containing protein, partial | taxID used:135614 OG01274|WP\_027700356.1 | exodeoxyribonuclease III | taxID used:135614 OG01275|WP\_004085088.1 | CPBP family intramembrane metalloprotease | taxID used:135614 OG01277|WP\_004085054.1 | bifunctional DNA-formamidopyrimidine glycosylase/DNA-(apurinic or apyrimidinic site) lyase | taxID used:135614 OG01278|WP\_027699983.1 | Fe-S cluster assembly ATPase SufC | taxID used:135614 OG01279|WP\_046420112.1 | tol-pal system protein YbgF | taxID used:135614 OG00127|WP\_057682436.1 | LPS-assembly protein LptD | taxID used:135614 OG01280|WP\_010894090.1 | hypothetical protein | taxID used:135614 OG01281|WP\_004084011.1 | 2-dehydro-3-deoxyphosphooctonate aldolase | taxID used:135614 OG01282|WP\_010892730.1 | 3'(2'),5'-bisphosphate nucleotidase | taxID used:135614 OG01283|WP\_004084043.1 | phosphoenolpyruvate synthetase regulatory protein | taxID used:135614 OG01284|WP\_010893560.1 | ribonuclease HII | taxID used:135614 OG01286|WP\_038200741.1 | ABC transporter ATP-binding protein | taxID used:135614 OG01287|WP\_058569805.1 | M48 family peptidase | taxID used:135614 OG01288|WP\_038210872.1 | amidohydrolase | taxID used:135614 OG01289|WP\_042836391.1 | F0F1 ATP synthase subunit A | taxID used:135614 OG00128|WP\_058564668.1 | alpha-1,2-mannosidase | taxID used:135614 OG01290|WP\_023907074.1 | ABC transporter permease | taxID used:135614 OG01291|WP\_046419578.1 | peptide chain release factor N(5)-glutamine methyltransferase | taxID used:135614 OG01292|WP\_081090423.1 | baseplate assembly protein | taxID used:135614 OG01293|WP\_020851812.1 | SDR family NAD(P)-dependent oxidoreductase | taxID used:135614 OG01294|WP\_109160974.1 | M48 family peptidase | taxID used:135614 OG01295|WP\_071869616.1 | DUF2303 domain-containing protein | taxID used:135614 OG01297|WP\_088578311.1 | succinate dehydrogenase iron-sulfur subunit | taxID used:135614 OG01298|WP\_081090394.1 | replication protein PDa0002 | taxID used:135614 OG00129|WP\_023907531.1 | TonB-dependent receptor | taxID used:135614 OG00012|WP\_024748949.1 | calcium-binding protein | taxID used:135614 OG01300|WP\_004089758.1 | hypothetical protein | taxID used:135614 OG01301|WP\_020851271.1 | thymidylate synthase | taxID used:135614 OG01303|WP\_020852605.1 | 3-methyl-2-oxobutanoate hydroxymethyltransferase | taxID used:135614 OG01305|WP\_027700039.1 | N6 adenine-specific DNA methyltransferase D12 class | taxID used:135614 OG01306|WP\_031336632.1 | GTPase | taxID used:135614 OG01308|WP\_023906157.1 | type IV pilus biogenesis/stability protein PilW | taxID used:135614 OG01309|WP\_004083553.1 | 16S rRNA (cytidine(1402)-2'-O)-methyltransferase | taxID used:135614 OG00130|WP\_057682313.1 | SAM-dependent DNA methyltransferase | taxID used:135614 OG01310|WP\_004088701.1 | DUF4198 domain-containing protein | taxID used:135614 OG01311|WP\_020853073.1 | bifunctional hydroxymethylpyrimidine kinase/phosphomethylpyrimidine kinase | taxID used:135614 OG01312|WP\_038229782.1 | pilus assembly protein PapD | taxID used:135614 OG01313|WP\_050765441.1 | FHA domain-containing protein | taxID used:135614 OG01314|WP\_004083405.1 | 1-(5-phosphoribosyl)-5-[(5-phosphoribosylamino)methylideneamino]imidazole-4-carboxamide isomerase | taxID used:135614 OG01315|WP\_004087867.1 | S-adenosylmethionine decarboxylase proenzyme | taxID used:135614 OG01316|WP\_023906557.1 | hypothetical protein | taxID used:135614 OG01317|WP\_004085053.1 | inner membrane protein YpjD | taxID used:135614 OG01318|WP\_004083429.1 | signal peptidase I | taxID used:135614 OG00131|WP\_060871822.1 | TonB-dependent receptor | taxID used:135614 OG01320|WP\_071869963.1 | oxidoreductase | taxID used:135614 OG01323|WP\_004090321.1 | 30S ribosomal protein S2 | taxID used:135614 OG01324|WP\_004089294.1 | folate-binding protein | taxID used:135614 OG01325|WP\_080939607.1 | exodeoxyribonuclease III | taxID used:135614 OG01326|WP\_011097591.1 | acyl-[acyl-carrier-protein]--UDP-N-acetylglucosamine O-acyltransferase | taxID used:135614 OG01327|WP\_010893043.1 | hypothetical protein | taxID used:135614 OG01329|WP\_057682964.1 | hypothetical protein | taxID used:135614 OG00132|WP\_012382661.1 | penicillin-binding protein 1C | taxID used:135614 OG01330|WP\_010893378.1 | 5'-nucleotidase SurE | taxID used:135614 OG01331|WP\_060871458.1 | P-type conjugative transfer protein TrbJ | taxID used:135614 OG01332|WP\_004083895.1 | septum site-determining protein MinD | taxID used:135614 OG01334|WP\_014607604.1 | arginyltransferase | taxID used:135614 OG01335|WP\_023907210.1 | TatD family deoxyribonuclease | taxID used:135614 OG01336|WP\_023907783.1 | thiol:disulfide interchange protein DsbA/DsbL | taxID used:135614 OG01337|WP\_021358311.1 | tRNA (guanosine(37)-N1)-methyltransferase TrmD | taxID used:135614 OG01338|WP\_038232779.1 | type I methionyl aminopeptidase | taxID used:135614 OG01339|WP\_004090667.1 | tryptophan synthase subunit alpha | taxID used:135614 OG00133|WP\_058569645.1 | penicillin-binding protein 1B | taxID used:135614 OG01340|WP\_060870069.1 | hypothetical protein | taxID used:135614 OG01341|WP\_023906737.1 | disulfide bond formation protein DsbC | taxID used:135614 OG01342|WP\_031336133.1 | hypothetical protein | taxID used:135614 OG01344|WP\_023907998.1 | phage repressor protein | taxID used:135614 OG01346|WP\_011098005.1 | hydroxyacylglutathione hydrolase | taxID used:135614 OG01347|WP\_024749251.1 | tRNA pseudouridine(38-40) synthase TruA | taxID used:135614 OG01348|WP\_031336649.1 | YggS family pyridoxal phosphate-dependent enzyme | taxID used:135614 OG01349|WP\_020852765.1 | ferredoxin--NADP reductase | taxID used:135614 OG00134|WP\_088578610.1 | phosphoenolpyruvate synthase | taxID used:135614 OG01350|WP\_004086575.1 | ABC transporter permease | taxID used:135614 OG01351|WP\_031337052.1 | methionine ABC transporter substrate-binding protein | taxID used:135614 OG01352|WP\_004083605.1 | ABC transporter ATP-binding protein | taxID used:135614 OG01353|WP\_024748784.1 | RNA methyltransferase | taxID used:135614 OG01354|WP\_071869626.1 | laccase | taxID used:135614 OG01356|WP\_004572975.1 | indole-3-glycerol phosphate synthase TrpC | taxID used:135614 OG01358|WP\_058564904.1 | pyridoxine 5'-phosphate synthase | taxID used:135614 OG01359|WP\_004090471.1 | thiazole synthase | taxID used:135614 OG00135|WP\_012337629.1 | outer membrane protein assembly factor BamA | taxID used:135614 OG01360|WP\_004572826.1 | undecaprenyl-diphosphatase | taxID used:135614 OG01361|WP\_052170053.1 | XRE family transcriptional regulator | taxID used:135614 OG01362|WP\_004089155.1 | endonuclease/exonuclease/phosphatase family protein | taxID used:135614 OG01363|WP\_058564322.1 | sulfurtransferase | taxID used:135614 OG01364|WP\_010892721.1 | outer membrane protein | taxID used:135614 OG01365|WP\_004085817.1 | glycosyltransferase | taxID used:135614 OG01366|WP\_027700669.1 | hypothetical protein | taxID used:135614 OG01367|WP\_004088266.1 | ParA family protein | taxID used:135614 OG01368|WP\_010893569.1 | ditrans,polycis-undecaprenyl-diphosphate synthase ((2E,6E)-farnesyl-diphosphate specific) | taxID used:135614 OG01369|WP\_023906627.1 | hypothetical protein | taxID used:135614 OG00136|WP\_023906998.1 | phenylalanine--tRNA ligase subunit beta | taxID used:135614 OG01370|WP\_046419223.1 | hypothetical protein | taxID used:135614 OG01372|WP\_038228299.1 | epoxyqueuosine reductase QueH | taxID used:135614 OG01373|WP\_010894497.1 | iron-containing redox enzyme family protein | taxID used:135614 OG01374|WP\_020852736.1 | hypothetical protein | taxID used:135614 OG01375|WP\_088572644.1 | short-chain dehydrogenase/reductase SDR | taxID used:135614 OG01376|WP\_057683248.1 | uroporphyrinogen-III synthase | taxID used:135614 OG01377|WP\_004083404.1 | imidazole glycerol phosphate synthase cyclase subunit | taxID used:135614 OG01378|WP\_060870294.1 | site-specific DNA-methyltransferase | taxID used:135614 OG00137|WP\_004088383.1 | hypothetical protein | taxID used:135614 OG01380|WP\_057682898.1 | 3-deoxy-manno-octulosonate cytidylyltransferase | taxID used:135614 OG01381|WP\_004085421.1 | protein TolQ | taxID used:135614 OG01382|WP\_010895179.1 | hypothetical protein | taxID used:135614 OG01383|WP\_004086464.1 | cytochrome c1 | taxID used:135614 OG01384|WP\_024749165.1 | hypothetical protein | taxID used:135614 OG01385|WP\_004083589.1 | CDP-diacylglycerol--serine O-phosphatidyltransferase | taxID used:135614 OG01386|WP\_046418504.1 | sulfite exporter TauE/SafE family protein | taxID used:135614 OG01387|WP\_010894935.1 | helix-turn-helix transcriptional regulator | taxID used:135614 OG01388|WP\_004086593.1 | phosphatase PAP2 family protein | taxID used:135614 OG01389|WP\_010895106.1 | uracil-DNA glycosylase | taxID used:135614 OG00138|WP\_038229842.1 | DNA translocase FtsK | taxID used:135614 OG01390|WP\_038230300.1 | hypothetical protein | taxID used:135614 OG01391|WP\_088578182.1 | 3-deoxy-D-manno-octulosonic acid kinase | taxID used:135614 OG01392|WP\_060870131.1 | hypothetical protein | taxID used:135614 OG01393|WP\_057682606.1 | NAD kinase | taxID used:135614 OG01394|WP\_023907825.1 | pimeloyl-[acyl-carrier protein] methyl ester esterase | taxID used:135614 OG01395|WP\_020851909.1 | hypothetical protein | taxID used:135614 OG01396|WP\_011098376.1 | hypothetical protein | taxID used:135614 OG01398|WP\_046420558.1 | 3-alpha-hydroxysteroid dehydrogenase | taxID used:135614 OG01399|WP\_010893988.1 | bifunctional demethylmenaquinone methyltransferase/2-methoxy-6-polyprenyl-1,4-benzoquinol methylase UbiE | taxID used:135614 OG00139|WP\_012337798.1 | DNA primase | taxID used:135614 OG00013|WP\_038210721.1 | pilus assembly protein | taxID used:135614 OG01400|WP\_038230430.1 | peptidase S24 | taxID used:135614 OG01401|WP\_038231425.1 | ParA family protein | taxID used:135614 OG01402|WP\_010894256.1 | hypothetical protein | taxID used:135614 OG01403|WP\_004088479.1 | heme ABC transporter permease | taxID used:135614 OG01404|WP\_023906120.1 | NADH-quinone oxidoreductase subunit C | taxID used:135614 OG01405|WP\_004084957.1 | DNA-binding response regulator | taxID used:135614 OG01406|WP\_053014136.1 | XRE family transcriptional regulator | taxID used:135614 OG01407|WP\_004087841.1 | MotA/TolQ/ExbB proton channel family protein | taxID used:135614 OG01408|WP\_046417487.1 | conjugal transfer protein TraL | taxID used:135614 OG00140|WP\_060872435.1 | Clp protease ClpP | taxID used:135614 OG01410|WP\_004085414.1 | 2,3-bisphosphoglycerate-dependent phosphoglycerate mutase | taxID used:135614 OG01411|WP\_012337788.1 | hypothetical protein | taxID used:135614 OG01412|WP\_058564316.1 | UDP-2,3-diacylglucosamine diphosphatase | taxID used:135614 OG01413|WP\_004091236.1 | XRE family transcriptional regulator | taxID used:135614 OG01415|WP\_004085434.1 | superoxide dismutase | taxID used:135614 OG01416|WP\_012382616.1 | monofunctional biosynthetic peptidoglycan transglycosylase | taxID used:135614 OG01417|WP\_010895252.1 | ParA family protein | taxID used:135614 OG01418|WP\_010894197.1 | 3-oxoacyl-ACP reductase | taxID used:135614 OG01419|WP\_010895228.1 | alpha/beta hydrolase | taxID used:135614 OG00141|WP\_038211324.1 | membrane protein | taxID used:135614 OG01420|WP\_046420943.1 | Bax inhibitor-1/YccA family protein | taxID used:135614 OG01421|WP\_058564233.1 | leucyl/phenylalanyl-tRNA--protein transferase | taxID used:135614 OG01423|WP\_004090474.1 | tRNA (guanine-N(7)-)-methyltransferase | taxID used:135614 OG01424|WP\_010892691.1 | SPOR domain-containing protein | taxID used:135614 OG01425|WP\_004091032.1 | S-methyl-5'-thioinosine phosphorylase | taxID used:135614 OG01426|WP\_010892612.1 | amidophosphoribosyltransferase | taxID used:135614 OG01429|WP\_057682812.1 | hypothetical protein | taxID used:135614 OG00142|WP\_031336322.1 | TonB-dependent siderophore receptor | taxID used:135614 OG01430|WP\_023907315.1 | hypothetical protein | taxID used:135614 OG01431|WP\_004086023.1 | bifunctional 3-demethylubiquinone 3-O-methyltransferase/2-octaprenyl-6-hydroxy phenol methylase | taxID used:135614 OG01432|WP\_023907257.1 | 3-ketoacyl-ACP reductase | taxID used:135614 OG01433|WP\_038210238.1 | Negative regulator of sigma E activity RseA | taxID used:135614 OG01434|WP\_012338095.1 | ketosynthase | taxID used:135614 OG01435|WP\_004085051.1 | 3-oxoacyl-ACP reductase FabG | taxID used:135614 OG01436|WP\_027700541.1 | orotidine-5'-phosphate decarboxylase | taxID used:135614 OG01437|WP\_080702474.1 | XRE family transcriptional regulator | taxID used:135614 OG01438|WP\_020851781.1 | hypothetical protein | taxID used:135614 OG01439|WP\_060870265.1 | hypothetical protein | taxID used:135614 OG00143|WP\_058564857.1 | RNA-binding transcriptional accessory protein | taxID used:135614 OG01441|WP\_004087940.1 | triose-phosphate isomerase | taxID used:135614 OG01442|WP\_010894215.1 | SDR family NAD(P)-dependent oxidoreductase | taxID used:135614 OG01443|WP\_011098260.1 | polysaccharide deacetylase family protein | taxID used:135614 OG01444|WP\_004085871.1 | ABC transporter ATP-binding protein | taxID used:135614 OG01446|WP\_057683156.1 | glutamine amidotransferase | taxID used:135614 OG01447|WP\_046418442.1 | cell division protein ZipA | taxID used:135614 OG01448|WP\_057682448.1 | LysM peptidoglycan-binding domain-containing protein | taxID used:135614 OG00144|WP\_060872012.1 | catalase/peroxidase HPI | taxID used:135614 OG01450|WP\_010892794.1 | electron transfer flavoprotein subunit beta/FixA family protein | taxID used:135614 OG01451|WP\_004084642.1 | ABC transporter ATP-binding protein | taxID used:135614 OG01452|WP\_038228024.1 | twin-arginine translocase subunit TatC | taxID used:135614 OG01454|WP\_020852327.1 | haloacid dehalogenase | taxID used:135614 OG01456|WP\_004091141.1 | 23S rRNA (guanosine(2251)-2'-O)-methyltransferase RlmB | taxID used:135614 OG01457|WP\_004083738.1 | short-chain dehydrogenase/reductase SDR | taxID used:135614 OG01458|WP\_023906699.1 | phosphoadenosine phosphosulfate reductase | taxID used:135614 OG01459|WP\_010894372.1 | YebC/PmpR family DNA-binding transcriptional regulator | taxID used:135614 OG00145|WP\_038232863.1 | type II secretion system protein GspD | taxID used:135614 OG01460|WP\_046417528.1 | conjugal transfer protein TrbF | taxID used:135614 OG01462|WP\_004090628.1 | copper homeostasis protein CutC | taxID used:135614 OG01463|WP\_004572909.1 | deoxyribonuclease V | taxID used:135614 OG01464|WP\_011098057.1 | site-specific DNA-methyltransferase | taxID used:135614 OG01465|WP\_040123137.1 | conjugal transfer protein TrbF | taxID used:135614 OG01467|WP\_057683340.1 | 3-oxoacyl-ACP reductase FabG | taxID used:135614 OG01468|WP\_004083606.1 | ABC transporter permease | taxID used:135614 OG00146|WP\_012337608.1 | NADP-dependent malic enzyme | taxID used:135614 OG01471|WP\_010892733.1 | 16S rRNA (uracil(1498)-N(3))-methyltransferase | taxID used:135614 OG01472|WP\_010894605.1 | serine/threonine-protein phosphatase | taxID used:135614 OG01473|WP\_027700097.1 | DNA repair protein RecO | taxID used:135614 OG01474|WP\_010893116.1 | nucleotidyltransferase family protein | taxID used:135614 OG01475|WP\_004088269.1 | dolichol-phosphate mannosyltransferase | taxID used:135614 OG01476|WP\_038230136.1 | 6-phosphogluconolactonase | taxID used:135614 OG01478|WP\_010893674.1 | 30S ribosomal protein S3 | taxID used:135614 OG01479|WP\_046419197.1 | hypothetical protein | taxID used:135614 OG00147|WP\_012337958.1 | 5-methyltetrahydropteroyltriglutamate--homocysteine S-methyltransferase | taxID used:135614 OG01480|WP\_004085659.1 | SIMPL domain-containing protein | taxID used:135614 OG01481|WP\_046419220.1 | hypothetical protein | taxID used:135614 OG01482|WP\_004088636.1 | phosphate-specific transport system accessory protein PhoU | taxID used:135614 OG01483|WP\_126709115.1 | phage-related protein | taxID used:135614 OG01484|WP\_010894805.1 | response regulator | taxID used:135614 OG01485|WP\_038230128.1 | UMP kinase | taxID used:135614 OG01487|WP\_023906693.1 | ribonuclease PH | taxID used:135614 OG01489|WP\_004083884.1 | polyisoprenoid-binding protein | taxID used:135614 OG01490|WP\_020852126.1 | DUF4194 domain-containing protein | taxID used:135614 OG01491|WP\_031336234.1 | cell envelope biogenesis protein OmpA | taxID used:135614 OG01492|WP\_010894208.1 | coenzyme F420-dependent NADP oxidoreductase | taxID used:135614 OG01493|WP\_038229647.1 | type III pantothenate kinase | taxID used:135614 OG01494|WP\_010893911.1 | LPS export ABC transporter ATP-binding protein | taxID used:135614 OG01495|WP\_010894975.1 | DUF3011 domain-containing protein | taxID used:135614 OG01496|WP\_010894606.1 | DNA polymerase III subunit epsilon | taxID used:135614 OG01497|WP\_060870501.1 | DNA-binding protein | taxID used:135614 OG01498|WP\_057682371.1 | PAP2 family protein | taxID used:135614 OG01499|WP\_023907913.1 | lipoprotein-releasing system ATP-binding protein LolD | taxID used:135614 OG01500|WP\_046419348.1 | septum site-determining protein MinC | taxID used:135614 OG01501|WP\_020851394.1 | polyisoprenoid-binding protein | taxID used:135614 OG01505|WP\_088578172.1 | phage-related protein | taxID used:135614 OG01506|WP\_058564891.1 | DUF2461 domain-containing protein | taxID used:135614 OG01508|WP\_010892740.1 | DUF502 domain-containing protein | taxID used:135614 OG01509|WP\_020851329.1 | acireductone synthase | taxID used:135614 OG00150|WP\_038228064.1 | cytochrome c biogenesis protein | taxID used:135614 OG01511|WP\_010893622.1 | 4-hydroxy-tetrahydrodipicolinate reductase | taxID used:135614 OG01513|WP\_010895242.1 | hypothetical protein | taxID used:135614 OG01515|WP\_046420777.1 | DNA-binding response regulator | taxID used:135614 OG01518|WP\_011098061.1 | hypothetical protein | taxID used:135614 OG01519|WP\_004086562.1 | 16S rRNA pseudouridine(516) synthase | taxID used:135614 OG01520|WP\_088572695.1 | hypothetical protein | taxID used:135614 OG01521|WP\_004088263.1 | energy transducer TonB | taxID used:135614 OG01523|WP\_023906667.1 | hypothetical protein | taxID used:135614 OG01524|WP\_023906194.1 | PKHD-type hydroxylase | taxID used:135614 OG01525|WP\_042463215.1 | hypothetical protein | taxID used:135614 OG01526|WP\_058564658.1 | ABC transporter permease | taxID used:135614 OG01527|WP\_004088673.1 | peptidylprolyl isomerase | taxID used:135614 OG01528|WP\_071869953.1 | hypothetical protein | taxID used:135614 OG01529|WP\_004084660.1 | phosphate regulon transcriptional regulatory protein PhoB | taxID used:135614 OG00152|WP\_004089140.1 | ATP-dependent Clp protease ATP-binding subunit ClpA | taxID used:135614 OG01530|WP\_058569536.1 | phytoene/squalene synthase family protein | taxID used:135614 OG01532|WP\_011098059.1 | hypothetical protein | taxID used:135614 OG01533|WP\_058569681.1 | lipoyl(octanoyl) transferase LipB | taxID used:135614 OG01534|WP\_040123197.1 | hypothetical protein | taxID used:135614 OG01538|WP\_023906769.1 | DNA-3-methyladenine glycosylase 2 family protein | taxID used:135614 OG01539|WP\_057683331.1 | phosphoglycolate phosphatase | taxID used:135614 OG00153|WP\_058569154.1 | DNA topoisomerase IV subunit A | taxID used:135614 OG01544|WP\_020852525.1 | type 1 glutamine amidotransferase domain-containing protein | taxID used:135614 OG01545|WP\_010895205.1 | P-type DNA transfer protein VirB5 | taxID used:135614 OG01547|WP\_004085576.1 | 7-cyano-7-deazaguanine synthase QueC | taxID used:135614 OG01548|WP\_046419709.1 | alpha/beta hydrolase | taxID used:135614 OG01549|WP\_004084731.1 | DUF2290 domain-containing protein | taxID used:135614 OG00154|WP\_046420864.1 | NADP-dependent isocitrate dehydrogenase | taxID used:135614 OG01550|WP\_020851534.1 | 2-C-methyl-D-erythritol 4-phosphate cytidylyltransferase | taxID used:135614 OG01551|WP\_038229417.1 | DNA-binding response regulator | taxID used:135614 OG01552|WP\_031336629.1 | hypothetical protein | taxID used:135614 OG01553|WP\_004083529.1 | DnaA regulatory inactivator Hda | taxID used:135614 OG01554|WP\_060872255.1 | hypothetical protein | taxID used:135614 OG01555|WP\_038210738.1 | DUF3108 domain-containing protein | taxID used:135614 OG01556|WP\_058564800.1 | endonuclease III | taxID used:135614 OG01558|WP\_010895052.1 | 50S ribosomal protein L1 | taxID used:135614 OG01559|WP\_058569836.1 | cAMP-activated global transcriptional regulator CRP | taxID used:135614 OG00155|WP\_057683088.1 | NADH dehydrogenase (quinone) subunit G | taxID used:135614 OG01560|WP\_023906167.1 | hypothetical protein | taxID used:135614 OG01563|WP\_058569547.1 | dethiobiotin synthase | taxID used:135614 OG01564|WP\_010894034.1 | tRNA (adenosine(37)-N6)-threonylcarbamoyltransferase complex dimerization subunit type 1 TsaB | taxID used:135614 OG01565|WP\_004084868.1 | alpha/beta hydrolase | taxID used:135614 OG01566|WP\_023906310.1 | ribonuclease 3 | taxID used:135614 OG01567|WP\_020852451.1 | HAD family phosphatase | taxID used:135614 OG01568|WP\_023907525.1 | nicotinate-nicotinamide nucleotide adenylyltransferase | taxID used:135614 OG01569|WP\_004088218.1 | carbonate dehydratase | taxID used:135614 OG00156|WP\_126715037.1 | primosomal protein N' | taxID used:135614 OG01570|WP\_060870379.1 | phage repressor protein C | taxID used:135614 OG01572|WP\_004087507.1 | cell division ATP-binding protein FtsE | taxID used:135614 OG01573|WP\_042463200.1 | phage repressor protein C | taxID used:135614 OG01575|WP\_020851467.1 | rhomboid family intramembrane serine protease | taxID used:135614 OG01576|WP\_046417796.1 | ribulose-phosphate 3-epimerase | taxID used:135614 OG01577|WP\_004085207.1 | rhomboid family intramembrane serine protease | taxID used:135614 OG01578|WP\_004083627.1 | response regulator transcription factor | taxID used:135614 OG01579|WP\_004087991.1 | serine/threonine protein kinase | taxID used:135614 OG00157|WP\_088372197.1 | TIGR01666 family membrane protein | taxID used:135614 OG01580|WP\_010894900.1 | heme exporter protein CcmB | taxID used:135614 OG01581|WP\_031336487.1 | DsbA family oxidoreductase | taxID used:135614 OG01582|WP\_004089909.1 | fimbrial protein | taxID used:135614 OG01583|WP\_011097508.1 | JAB domain-containing protein | taxID used:135614 OG01584|WP\_004083522.1 | peptidase | taxID used:135614 OG01585|WP\_023906947.1 | protein-L-isoaspartate O-methyltransferase | taxID used:135614 OG01587|WP\_004085446.1 | peptide-methionine (S)-S-oxide reductase MsrA | taxID used:135614 OG01588|WP\_057682944.1 | methylthioribulose 1-phosphate dehydratase | taxID used:135614 OG01589|WP\_004086597.1 | fimbrial protein | taxID used:135614 OG00158|WP\_088577680.1 | beta-glucosidase BglX | taxID used:135614 OG01590|WP\_004085415.1 | 7-carboxy-7-deazaguanine synthase QueE | taxID used:135614 OG01591|WP\_023907432.1 | cytidylate kinase | taxID used:135614 OG01592|WP\_010894955.1 | DNA-binding response regulator | taxID used:135614 OG01594|WP\_004083718.1 | DUF484 domain-containing protein | taxID used:135614 OG01596|WP\_081089885.1 | heme ABC exporter ATP-binding protein CcmA | taxID used:135614 OG01597|WP\_020851943.1 | energy transducer TonB | taxID used:135614 OG01598|WP\_021358244.1 | outer membrane lipoprotein LolB | taxID used:135614 OG01599|WP\_046419411.1 | N-(5'-phosphoribosyl)anthranilate isomerase | taxID used:135614 OG00159|WP\_071869751.1 | DNA helicase II | taxID used:135614 OG00015|WP\_038228479.1 | glutamate synthase large subunit | taxID used:135614 OG01602|WP\_080507225.1 | NADH-quinone oxidoreductase subunit J | taxID used:135614 OG01603|WP\_058564533.1 | tetratricopeptide repeat protein | taxID used:135614 OG01604|WP\_058565058.1 | XRE family transcriptional regulator | taxID used:135614 OG01605|WP\_004084654.1 | protein-L-isoaspartate O-methyltransferase | taxID used:135614 OG01606|WP\_027700531.1 | hypothetical protein | taxID used:135614 OG01608|WP\_027700549.1 | 2-nonaprenyl-3-methyl-6-methoxy-1,4-benzoquinol hydroxylase | taxID used:135614 OG01609|WP\_060870113.1 | DUF2076 domain-containing protein | taxID used:135614 OG00160|WP\_011097929.1 | phage tail tape measure protein | taxID used:135614 OG01610|WP\_010894494.1 | FMN-binding negative transcriptional regulator | taxID used:135614 OG01612|WP\_004572852.1 | CvpA family protein | taxID used:135614 OG01613|WP\_010893111.1 | phosphoribosylglycinamide formyltransferase | taxID used:135614 OG01615|WP\_020851828.1 | phospholipid-binding protein MlaC | taxID used:135614 OG01617|WP\_010892698.1 | orotate phosphoribosyltransferase | taxID used:135614 OG01618|WP\_004088385.1 | hypothetical protein | taxID used:135614 OG01620|WP\_020851296.1 | 3-isopropylmalate dehydratase small subunit | taxID used:135614 OG01622|WP\_010894385.1 | flavodoxin family protein | taxID used:135614 OG01623|WP\_058569633.1 | outer membrane protein W OmpW | taxID used:135614 OG01624|WP\_010894231.1 | DUF3275 domain-containing protein | taxID used:135614 OG01625|WP\_004085189.1 | MotA/TolQ/ExbB proton channel family protein | taxID used:135614 OG01626|WP\_004085033.1 | phospholipid/glycerol acyltransferase | taxID used:135614 OG01627|WP\_038230133.1 | ketohydroxyglutarate aldolase | taxID used:135614 OG01629|WP\_027699991.1 | outer membrane lipoprotein chaperone LolA | taxID used:135614 OG00162|WP\_046420838.1 | L-ascorbate oxidase | taxID used:135614 OG01630|WP\_004091006.1 | polysaccharide biosynthesis protein GumB | taxID used:135614 OG01631|WP\_010893937.1 | thiol:disulfide interchange protein DsbA/DsbL | taxID used:135614 OG01632|WP\_071869641.1 | RlmE family RNA methyltransferase | taxID used:135614 OG01633|WP\_004091185.1 | ribose-5-phosphate isomerase RpiA | taxID used:135614 OG01634|WP\_081033486.1 | ribosome maturation factor RimP | taxID used:135614 OG01635|WP\_004083987.1 | ABC transporter | taxID used:135614 OG01636|WP\_010894484.1 | YkgJ family cysteine cluster protein | taxID used:135614 OG01638|WP\_031336908.1 | 50S ribosomal protein L3 | taxID used:135614 OG01639|WP\_004087541.1 | DNA-binding response regulator | taxID used:135614 OG00163|WP\_010894922.1 | phage tail tape measure protein | taxID used:135614 OG01641|WP\_088371519.1 | aminodeoxychorismate/anthranilate synthase component II | taxID used:135614 OG01642|WP\_031336485.1 | ribonuclease T | taxID used:135614 OG01644|WP\_004085049.1 | hemolysin III family protein | taxID used:135614 OG01646|WP\_004086458.1 | acyl-ACP--UDP-N- acetylglucosamine O-acyltransferase | taxID used:135614 OG01647|WP\_010893601.1 | ParA family protein | taxID used:135614 OG01648|WP\_010894757.1 | CDP-diacylglycerol--glycerol-3-phosphate 3-phosphatidyltransferase | taxID used:135614 OG01649|WP\_004085407.1 | histidine phosphatase family protein | taxID used:135614 OG01652|WP\_080507193.1 | fatty acyl CoA synthetase | taxID used:135614 OG01655|WP\_038210736.1 | thymidylate kinase | taxID used:135614 OG01656|WP\_004088271.1 | nicotinamidase | taxID used:135614 OG01658|WP\_038229910.1 | stringent starvation protein A | taxID used:135614 OG01659|WP\_004083784.1 | magnesium transporter | taxID used:135614 OG00165|WP\_012337637.1 | phage tail tape measure protein | taxID used:135614 OG01660|WP\_010894298.1 | SCP2 domain-containing protein | taxID used:135614 OG01664|WP\_004086468.1 | ubiquinol-cytochrome c reductase iron-sulfur subunit | taxID used:135614 OG01669|WP\_126709105.1 | DUF4105 domain-containing protein, partial | taxID used:135614 OG01670|WP\_010894838.1 | glycoside hydrolase family 25 | taxID used:135614 OG01672|WP\_004083425.1 | RNA polymerase sigma factor RpoE | taxID used:135614 OG01673|WP\_004087331.1 | DUF2058 domain-containing protein | taxID used:135614 OG01674|WP\_004088341.1 | phage-related protein | taxID used:135614 OG01675|WP\_010893439.1 | tRNA threonylcarbamoyladenosine biosynthesis protein RimN | taxID used:135614 OG01677|WP\_080507190.1 | peptidoglycan endopeptidase | taxID used:135614 OG01678|WP\_118853413.1 | toprim domain-containing protein, partial | taxID used:135614 OG01679|WP\_004087395.1 | 50S ribosomal protein L25/general stress protein Ctc | taxID used:135614 OG01680|WP\_012337602.1 | hemolysin D | taxID used:135614 OG01681|WP\_088578071.1 | hypothetical protein | taxID used:135614 OG01682|WP\_020852082.1 | transcriptional repressor LexA | taxID used:135614 OG01683|WP\_057683487.1 | YbhB/YbcL family Raf kinase inhibitor-like protein | taxID used:135614 OG01685|WP\_004083680.1 | general secretion pathway protein GspM | taxID used:135614 OG01686|WP\_004572865.1 | ribosomal RNA small subunit methyltransferase G | taxID used:135614 OG01687|WP\_011097763.1 | general secretory pathway protein GspJ | taxID used:135614 OG01688|WP\_010894263.1 | SPOR domain-containing protein | taxID used:135614 OG01690|WP\_004087911.1 | 16S rRNA (guanine(966)-N(2))-methyltransferase RsmD | taxID used:135614 OG01691|WP\_020852792.1 | HNH endonuclease | taxID used:135614 OG01692|WP\_010893491.1 | response regulator transcription factor | taxID used:135614 OG01694|WP\_010893688.1 | 30S ribosomal protein S4 | taxID used:135614 OG01699|WP\_020851188.1 | membrane protein | taxID used:135614 OG00016|WP\_010893887.1 | ATP-dependent helicase | taxID used:135614 OG01700|WP\_011097655.1 | ATP-dependent Clp protease proteolytic subunit | taxID used:135614 OG01701|WP\_031336534.1 | GTP cyclohydrolase I FolE | taxID used:135614 OG01702|WP\_060871952.1 | glutathione S-transferase | taxID used:135614 OG01703|WP\_046419569.1 | guanylate kinase | taxID used:135614 OG01708|WP\_010895031.1 | superoxide dismutase | taxID used:135614 OG00170|WP\_046417956.1 | guanosine-3',5'-bis(diphosphate) 3'-diphosphatase | taxID used:135614 OG01710|WP\_060871813.1 | hypothetical protein | taxID used:135614 OG01711|WP\_010894225.1 | MULTISPECIES: integrase [ | taxID used:135614 OG01712|WP\_004083711.1 | thioredoxin family protein | taxID used:135614 OG01713|WP\_072866352.1 | conjugal transfer protein TrbN | taxID used:135614 OG01714|WP\_076613237.1 | hypothetical protein | taxID used:135614 OG01715|WP\_010894538.1 | TetR/AcrR family transcriptional regulator | taxID used:135614 OG01716|WP\_011098259.1 | D-alanyl-D-alanine carboxypeptidase family protein | taxID used:135614 OG01717|WP\_011097781.1 | YdcF family protein | taxID used:135614 OG01718|WP\_060872074.1 | hypothetical protein | taxID used:135614 OG01720|WP\_004085604.1 | hypothetical protein | taxID used:135614 OG01721|WP\_023906948.1 | DedA family protein | taxID used:135614 OG01722|WP\_031337900.1 | YihA family ribosome biogenesis GTP-binding protein | taxID used:135614 OG01723|WP\_004089235.1 | cytochrome o ubiquinol oxidase subunit III | taxID used:135614 OG01725|WP\_021358213.1 | 2-amino-4-hydroxy-6-hydroxymethyldihydropteridine diphosphokinase | taxID used:135614 OG01726|WP\_004087040.1 | cell filamentation protein Fic | taxID used:135614 OG01727|WP\_004088572.1 | rhomboid family intramembrane serine protease | taxID used:135614 OG01728|WP\_020851236.1 | dephospho-CoA kinase | taxID used:135614 OG01729|WP\_004088320.1 | bifunctional phosphoribosyl-AMP cyclohydrolase/phosphoribosyl-ATP diphosphatase HisIE | taxID used:135614 OG01732|WP\_010895232.1 | conjugal transfer protein TrbN | taxID used:135614 OG01733|WP\_088577668.1 | thiamine phosphate synthase | taxID used:135614 OG01735|WP\_038228342.1 | hypothetical protein | taxID used:135614 OG01736|WP\_004083634.1 | hypothetical protein | taxID used:135614 OG01738|WP\_010892742.1 | class I SAM-dependent methyltransferase | taxID used:135614 OG01739|WP\_058570052.1 | hypothetical protein | taxID used:135614 OG00173|WP\_031336553.1 | peptidyl-dipeptidase Dcp | taxID used:135614 OG01740|WP\_052151232.1 | hypothetical protein | taxID used:135614 OG01741|WP\_004088848.1 | hypothetical protein | taxID used:135614 OG01742|WP\_031336824.1 | pyridoxamine 5'-phosphate oxidase | taxID used:135614 OG01743|WP\_071869833.1 | DUF998 domain-containing protein | taxID used:135614 OG01745|WP\_081089742.1 | hypothetical protein | taxID used:135614 OG01746|WP\_042463485.1 | LysE family translocator | taxID used:135614 OG01747|WP\_021358333.1 | 4-phosphopantetheinyl transferase | taxID used:135614 OG01748|WP\_042462768.1 | hypothetical protein | taxID used:135614 OG00174|WP\_027699982.1 | S9 family peptidase | taxID used:135614 OG01750|WP\_060870337.1 | hypothetical protein | taxID used:135614 OG01751|WP\_071869539.1 | 50S ribosomal protein L4 | taxID used:135614 OG01753|WP\_010893940.1 | lysogenization protein HflD | taxID used:135614 OG01754|WP\_057682614.1 | 5-formyltetrahydrofolate cyclo-ligase | taxID used:135614 OG01755|WP\_004086014.1 | thiol:disulfide interchange protein DsbE | taxID used:135614 OG01756|WP\_011097758.1 | non-canonical purine NTP pyrophosphatase, RdgB/HAM1 family | taxID used:135614 OG01757|WP\_058569718.1 | hypothetical protein | taxID used:135614 OG01758|WP\_004084669.1 | Fe-S biogenesis protein NfuA | taxID used:135614 OG00175|WP\_046420029.1 | peptidase S41 | taxID used:135614 OG01761|WP\_024748621.1 | hypothetical protein | taxID used:135614 OG01763|WP\_023906328.1 | imidazole glycerol phosphate synthase subunit HisH | taxID used:135614 OG01765|WP\_023907960.1 | hypothetical protein | taxID used:135614 OG01766|WP\_046417983.1 | RNA pyrophosphohydrolase | taxID used:135614 OG01767|WP\_057683711.1 | hypothetical protein | taxID used:135614 OG01768|WP\_080939604.1 | hypothetical protein | taxID used:135614 OG01769|WP\_020852363.1 | oligoribonuclease | taxID used:135614 OG00176|WP\_004087928.1 | NADH-quinone oxidoreductase subunit L | taxID used:135614 OG01770|WP\_004085584.1 | DNA topoisomerase III | taxID used:135614 OG01771|WP\_046418994.1 | NAD(P)H:quinone oxidoreductase | taxID used:135614 OG01776|WP\_004084887.1 | recombination protein RecR | taxID used:135614 OG01777|WP\_011098232.1 | pilus assembly protein | taxID used:135614 OG01778|WP\_004089677.1 | riboflavin synthase | taxID used:135614 OG00177|WP\_023906772.1 | bifunctional (p)ppGpp synthetase/guanosine-3',5'-bis(diphosphate) 3'-pyrophosphohydrolase | taxID used:135614 OG01780|WP\_021358670.1 | hypothetical protein | taxID used:135614 OG01781|WP\_046420827.1 | YhgN family NAAT transporter | taxID used:135614 OG01782|WP\_088371597.1 | hypothetical protein | taxID used:135614 OG01783|WP\_023906782.1 | alpha-ketoglutarate-dependent dioxygenase AlkB | taxID used:135614 OG01784|WP\_010893285.1 | dCTP deaminase | taxID used:135614 OG01785|WP\_020851300.1 | hypothetical protein | taxID used:135614 OG01786|WP\_023906077.1 | aminodeoxychorismate/anthranilate synthase component II | taxID used:135614 OG01788|WP\_023906005.1 | pilus assembly protein | taxID used:135614 OG01789|WP\_011097612.1 | phage baseplate assembly protein V | taxID used:135614 OG00178|WP\_010894353.1 | S46 family peptidase | taxID used:135614 OG01790|WP\_004083893.1 | GNAT family N-acetyltransferase | taxID used:135614 OG01791|WP\_060872144.1 | phage baseplate assembly protein V | taxID used:135614 OG01792|WP\_004086710.1 | recombinase family protein | taxID used:135614 OG01793|WP\_004085209.1 | membrane protein | taxID used:135614 OG01794|WP\_010894927.1 | phage baseplate assembly protein V | taxID used:135614 OG01796|WP\_004084727.1 | N-acetylmuramoyl-L-alanine amidase | taxID used:135614 OG01797|WP\_060872324.1 | DUF4065 domain-containing protein | taxID used:135614 OG01799|WP\_060870181.1 | DUF4065 domain-containing protein | taxID used:135614 OG00179|WP\_011098330.1 | ribosomal RNA large subunit methyltransferase K/L | taxID used:135614 OG00017|WP\_023907139.1 | hemolysin | taxID used:135614 OG01800|WP\_004091174.1 | nitroreductase | taxID used:135614 OG01801|WP\_004087394.1 | aminoacyl-tRNA hydrolase | taxID used:135614 OG01802|WP\_010893603.1 | polyisoprenoid-binding protein | taxID used:135614 OG01803|WP\_004088656.1 | hypothetical protein | taxID used:135614 OG01804|WP\_023906333.1 | acireductone dioxygenase | taxID used:135614 OG01805|WP\_042466509.1 | site-specific integrase | taxID used:135614 OG01806|WP\_011097826.1 | iron-containing redox enzyme family protein | taxID used:135614 OG01807|WP\_010894101.1 | glutathione peroxidase | taxID used:135614 OG01808|WP\_020851002.1 | DUF1439 domain-containing protein | taxID used:135614 OG01809|WP\_010894204.1 | flavodoxin family protein | taxID used:135614 OG00180|WP\_038228806.1 | hybrid sensor histidine kinase/response regulator | taxID used:135614 OG01811|WP\_010893913.1 | LPS export ABC transporter periplasmic protein LptC | taxID used:135614 OG01812|WP\_010893041.1 | glycoside hydrolase family 24 | taxID used:135614 OG01814|WP\_031336436.1 | manganese efflux pump MntP family protein | taxID used:135614 OG01815|WP\_010894272.1 | Ax21 family protein | taxID used:135614 OG01816|WP\_020852749.1 | DUF3106 domain-containing protein | taxID used:135614 OG01817|WP\_031337124.1 | DUF4019 domain-containing protein | taxID used:135614 OG01818|WP\_088371816.1 | hypothetical protein | taxID used:135614 OG01819|WP\_038211556.1 | glycine cleavage system regulatory protein | taxID used:135614 OG00181|WP\_032489973.1 | repB/MobA-like protein | taxID used:135614 OG01821|WP\_012382746.1 | hypothetical protein | taxID used:135614 OG01822|WP\_081089929.1 | hypothetical protein | taxID used:135614 OG01824|WP\_027700064.1 | flavodoxin family protein | taxID used:135614 OG01825|WP\_046420118.1 | Holliday junction branch migration protein RuvA | taxID used:135614 OG01826|WP\_004090608.1 | cytochrome b | taxID used:135614 OG01827|WP\_046419046.1 | chorismate mutase AroQ, gamma subclass | taxID used:135614 OG01828|WP\_004083888.1 | polyisoprenoid-binding protein | taxID used:135614 OG01829|WP\_046420888.1 | TetR/AcrR family transcriptional regulator | taxID used:135614 OG00182|WP\_004572844.1 | glycine--tRNA ligase subunit beta | taxID used:135614 OG01831|WP\_081046815.1 | RDD family protein | taxID used:135614 OG01833|WP\_012338001.1 | elongation factor P | taxID used:135614 OG01834|WP\_010893403.1 | hypothetical protein | taxID used:135614 OG01835|WP\_004085654.1 | DUF615 family protein | taxID used:135614 OG01837|WP\_004085302.1 | NADH-quinone oxidoreductase subunit B | taxID used:135614 OG01838|WP\_058569279.1 | DNA-binding response regulator | taxID used:135614 OG01839|WP\_038230568.1 | phage tail protein I | taxID used:135614 OG00183|WP\_004088742.1 | peptidase | taxID used:135614 OG01840|WP\_020851452.1 | dTDP-4-dehydrorhamnose 3,5-epimerase | taxID used:135614 OG01841|WP\_010894676.1 | YqgE/AlgH family protein | taxID used:135614 OG01842|WP\_012337808.1 | gamma carbonic anhydrase family protein | taxID used:135614 OG00184|WP\_057683291.1 | type I secretion system permease/ATPase | taxID used:135614 OG01850|WP\_020852892.1 | peroxiredoxin | taxID used:135614 OG01852|WP\_004087851.1 | prepilin-type N-terminal cleavage/methylation domain-containing protein | taxID used:135614 OG01856|WP\_004084694.1 | transcription termination/antitermination protein NusG | taxID used:135614 OG01857|WP\_088577970.1 | hypothetical protein | taxID used:135614 OG01859|WP\_031336449.1 | elongation factor P-like protein YeiP | taxID used:135614 OG00185|WP\_046420800.1 | elongation factor G | taxID used:135614 OG01860|WP\_046420657.1 | hypoxanthine-guanine phosphoribosyltransferase | taxID used:135614 OG01862|WP\_010893065.1 | pilin | taxID used:135614 OG01863|WP\_046419079.1 | DUF1415 domain-containing protein | taxID used:135614 OG01864|WP\_023906354.1 | prepilin-type cleavage/methylation domain-containing protein | taxID used:135614 OG01866|WP\_003821054.1 | MULTISPECIES: DUF2857 domain-containing prot | taxID used:135614 OG01867|WP\_012382543.1 | carbonate dehydratase | taxID used:135614 OG01868|WP\_071869506.1 | recombinase family protein | taxID used:135614 OG00186|WP\_038210656.1 | ATP-dependent DNA helicase RecG | taxID used:135614 OG01871|WP\_004572792.1 | hypothetical protein | taxID used:135614 OG01872|WP\_040123213.1 | hypothetical protein | taxID used:135614 OG01873|WP\_021358237.1 | Maf-like protein | taxID used:135614 OG01875|WP\_027700732.1 | hypothetical protein | taxID used:135614 OG01876|WP\_010894457.1 | putative Fe-S cluster assembly protein SufT | taxID used:135614 OG01878|WP\_058570057.1 | hypothetical protein | taxID used:135614 OG00187|WP\_071869975.1 | phage terminase GpA | taxID used:135614 OG01882|WP\_060872235.1 | hypothetical protein | taxID used:135614 OG01884|WP\_058565101.1 | hypothetical protein | taxID used:135614 OG01885|WP\_012337826.1 | DUF2726 domain-containing protein | taxID used:135614 OG01889|WP\_012337802.1 | NUDIX hydrolase | taxID used:135614 OG00188|WP\_057682834.1 | ABC transporter | taxID used:135614 OG01890|WP\_046417645.1 | type 1 fimbrial protein | taxID used:135614 OG01891|WP\_031336627.1 | hypothetical protein | taxID used:135614 OG01892|WP\_024748852.1 | SMR domain protein | taxID used:135614 OG01893|WP\_031336530.1 | TlpA family protein disulfide reductase | taxID used:135614 OG01894|WP\_060871902.1 | hypothetical protein | taxID used:135614 OG01895|WP\_004085245.1 | hypothetical protein | taxID used:135614 OG01896|WP\_004088351.1 | hypothetical protein | taxID used:135614 OG01897|WP\_020851087.1 | YecA family protein | taxID used:135614 OG01898|WP\_058564793.1 | hypothetical protein | taxID used:135614 OG01899|WP\_004090677.1 | DNA starvation/stationary phase protection protein | taxID used:135614 OG00189|WP\_071869724.1 | S9 family peptidase | taxID used:135614 OG00018|WP\_011098326.1 | DNA-directed RNA polymerase subunit beta' | taxID used:135614 OG01900|WP\_004085328.1 | adenylate kinase | taxID used:135614 OG01901|WP\_004084122.1 | hypothetical protein | taxID used:135614 OG01902|WP\_060870086.1 | hypothetical protein | taxID used:135614 OG01903|WP\_004091417.1 | recombinase family protein | taxID used:135614 OG01904|WP\_004085039.1 | ADP compounds hydrolase NudE | taxID used:135614 OG01905|WP\_046420226.1 | recombinase family protein | taxID used:135614 OG01906|WP\_004089320.1 | ribosome recycling factor | taxID used:135614 OG01907|WP\_020852310.1 | integrase, partial | taxID used:135614 OG01908|WP\_058564581.1 | hypothetical protein | taxID used:135614 OG01909|WP\_004083880.1 | shikimate kinase | taxID used:135614 OG00190|WP\_046419208.1 | portal protein | taxID used:135614 OG01910|WP\_046418751.1 | transcriptional repressor NrdR | taxID used:135614 OG01915|WP\_004089226.1 | lactoylglutathione lyase | taxID used:135614 OG01916|WP\_004089213.1 | HAD family hydrolase | taxID used:135614 OG01918|WP\_004088597.1 | inorganic pyrophosphatase | taxID used:135614 OG01919|WP\_010893678.1 | 50S ribosomal protein L5 | taxID used:135614 OG00191|WP\_088577712.1 | methionine--tRNA ligase | taxID used:135614 OG01920|WP\_081089785.1 | pilin | taxID used:135614 OG01921|WP\_081089930.1 | hypothetical protein | taxID used:135614 OG01923|WP\_031336568.1 | peptidoglycan-associated lipoprotein Pal | taxID used:135614 OG01927|WP\_100206159.1 | membrane protein | taxID used:135614 OG01928|WP\_004085066.1 | GNAT family N-acetyltransferase | taxID used:135614 OG01929|WP\_088577940.1 | conjugative transfer signal peptidase TraF | taxID used:135614 OG00192|WP\_004087521.1 | polyphosphate kinase 1 | taxID used:135614 OG01930|WP\_010893985.1 | ATP-dependent protease subunit HslV | taxID used:135614 OG01931|WP\_004083607.1 | outer membrane lipid asymmetry maintenance protein MlaD | taxID used:135614 OG01932|WP\_057683592.1 | hypothetical protein | taxID used:135614 OG01933|WP\_046419794.1 | hypothetical protein | taxID used:135614 OG01936|WP\_004086539.1 | 30S ribosomal protein S5 | taxID used:135614 OG01937|WP\_024748573.1 | hypothetical protein | taxID used:135614 OG01939|WP\_004087934.1 | NADH-quinone oxidoreductase subunit NuoE | taxID used:135614 OG00193|WP\_057682651.1 | transketolase | taxID used:135614 OG01941|WP\_075584689.1 | translation initiation factor IF-3 | taxID used:135614 OG01944|WP\_023907472.1 | peptidase | taxID used:135614 OG01946|WP\_004089217.1 | lipopolysaccharide transport periplasmic protein LptA | taxID used:135614 OG01947|WP\_004572990.1 | DUF2059 domain-containing protein | taxID used:135614 OG01949|WP\_004085458.1 | purine-binding chemotaxis protein CheW | taxID used:135614 OG00194|WP\_060872173.1 | TonB-dependent receptor | taxID used:135614 OG01950|WP\_011098214.1 | fimbrial protein | taxID used:135614 OG01951|WP\_010892873.1 | disulfide bond formation protein B | taxID used:135614 OG01952|WP\_004088700.1 | DUF2271 domain-containing protein | taxID used:135614 OG01953|WP\_031345912.1 | methylated-DNA--[protein]-cysteine S-methyltransferase | taxID used:135614 OG01955|WP\_046420230.1 | hypothetical protein | taxID used:135614 OG01959|WP\_012338155.1 | hypothetical protein | taxID used:135614 OG00195|WP\_046418373.1 | EAL domain-containing protein | taxID used:135614 OG01960|WP\_004088753.1 | 3-hydroxyacyl-[acyl-carrier-protein] dehydratase FabA | taxID used:135614 OG01962|WP\_024749190.1 | hypothetical protein | taxID used:135614 OG01966|WP\_004084453.1 | transcriptional repressor | taxID used:135614 OG01967|WP\_004089606.1 | hypothetical protein | taxID used:135614 OG00196|WP\_057683128.1 | hypothetical protein | taxID used:135614 OG01972|WP\_004085641.1 | F0F1 ATP synthase subunit delta | taxID used:135614 OG01974|WP\_027700246.1 | alpha/beta hydrolase | taxID used:135614 OG01975|WP\_010893681.1 | 50S ribosomal protein L6 | taxID used:135614 OG01978|WP\_088371452.1 | phage portal protein | taxID used:135614 OG01979|WP\_004086991.1 | phage tail protein I | taxID used:135614 OG00197|WP\_011097916.1 | DUF3488 domain-containing protein | taxID used:135614 OG01980|WP\_010892654.1 | ribosome maturation factor RimM | taxID used:135614 OG01981|WP\_004084893.1 | protein-export protein SecB | taxID used:135614 OG01982|WP\_004089770.1 | tfp pilus assembly protein fimT | taxID used:135614 OG01985|WP\_080507231.1 | tRNA adenosine(34) deaminase TadA | taxID used:135614 OG01986|WP\_004572911.1 | glutathione peroxidase | taxID used:135614 OG01987|WP\_010894371.1 | crossover junction endodeoxyribonuclease RuvC | taxID used:135614 OG01988|WP\_031336846.1 | poly(hydroxyalcanoate) granule associated protein | taxID used:135614 OG00198|WP\_057683072.1 | phosphoesterase | taxID used:135614 OG01991|WP\_004089639.1 | peptide deformylase | taxID used:135614 OG01993|WP\_004084691.1 | 50S ribosomal protein L10 | taxID used:135614 OG01995|WP\_038228929.1 | hypothetical protein | taxID used:135614 OG01996|WP\_023907227.1 | 2-amino-4-hydroxy-6-hydroxymethyldihydropteridine diphosphokinase | taxID used:135614 OG01997|WP\_020852367.1 | DUF1249 domain-containing protein | taxID used:135614 OG01998|WP\_004085935.1 | lipoprotein signal peptidase | taxID used:135614 OG01999|WP\_004085850.1 | nucleotide exchange factor GrpE | taxID used:135614 OG00199|WP\_060872247.1 | polyribonucleotide nucleotidyltransferase | taxID used:135614 OG00019|WP\_118853458.1 | adhesin | taxID used:135614 OG02001|WP\_010894531.1 | hypothetical protein | taxID used:135614 OG02002|WP\_004085836.1 | SsrA-binding protein SmpB | taxID used:135614 OG02003|WP\_031336828.1 | hypothetical protein | taxID used:135614 OG02005|WP\_010892757.1 | MarR family transcriptional regulator | taxID used:135614 OG02008|WP\_004086635.1 | hypothetical protein | taxID used:135614 OG02009|WP\_004088474.1 | hypothetical protein | taxID used:135614 OG00200|WP\_058569712.1 | tetratricopeptide repeat protein | taxID used:135614 OG02011|WP\_031337936.1 | 2-C-methyl-D-erythritol 2,4-cyclodiphosphate synthase | taxID used:135614 OG02013|WP\_080679705.1 | hypothetical protein | taxID used:135614 OG02016|WP\_004085228.1 | pathogenicity | taxID used:135614 OG02017|WP\_023906584.1 | DUF177 domain-containing protein | taxID used:135614 OG02018|WP\_053014138.1 | lysozyme | taxID used:135614 OG02019|WP\_010893466.1 | hypothetical protein | taxID used:135614 OG00201|WP\_088578119.1 | DNA topoisomerase III | taxID used:135614 OG02022|WP\_004083791.1 | Single-stranded DNA-binding protein 2 | taxID used:135614 OG02024|WP\_004083685.1 | prepilin-type N-terminal cleavage/methylation domain-containing protein | taxID used:135614 OG02025|WP\_072866363.1 | integrase | taxID used:135614 OG02026|WP\_004087369.1 | 8-oxo-dGTP diphosphatase | taxID used:135614 OG02027|WP\_011097819.1 | SgcJ/EcaC family oxidoreductase | taxID used:135614 OG02029|WP\_046420774.1 | dihydrofolate reductase | taxID used:135614 OG00202|WP\_058565150.1 | 1,4-beta-cellobiosidase | taxID used:135614 OG02031|WP\_004088821.1 | Rnf electron transport complex subunit RnfB | taxID used:135614 OG02033|WP\_004086570.1 | peptidylprolyl isomerase | taxID used:135614 OG02035|WP\_020851178.1 | fimbrial assembly protein | taxID used:135614 OG02036|WP\_004085823.1 | CYTH domain-containing protein | taxID used:135614 OG02037|WP\_072866390.1 | peptidase | taxID used:135614 OG02038|WP\_004084756.1 | 5-(carboxyamino)imidazole ribonucleotide mutase | taxID used:135614 OG02045|WP\_020853053.1 | DUF1566 domain-containing protein | taxID used:135614 OG02047|WP\_027700520.1 | hypothetical protein | taxID used:135614 OG02048|WP\_004085295.1 | NADH-quinone oxidoreductase subunit NuoI | taxID used:135614 OG02049|WP\_023907910.1 | L-asparaginase | taxID used:135614 OG00204|WP\_004085393.1 | glycosyl hydrolase family 3 | taxID used:135614 OG02051|WP\_023907752.1 | peptidase | taxID used:135614 OG02052|WP\_046420603.1 | hypothetical protein | taxID used:135614 OG02053|WP\_027700581.1 | low molecular weight phosphotyrosine protein phosphatase | taxID used:135614 OG02057|WP\_051372743.1 | hypothetical protein | taxID used:135614 OG02058|WP\_010893460.1 | thiol-disulfide oxidoreductase DCC family protein | taxID used:135614 OG00205|WP\_038211027.1 | penicillin-binding protein 2 | taxID used:135614 OG02062|WP\_031337990.1 | hypothetical protein | taxID used:135614 OG02064|WP\_010893563.1 | 3-hydroxyacyl-ACP dehydratase FabZ | taxID used:135614 OG02065|WP\_004085587.1 | hypothetical protein | taxID used:135614 OG02069|WP\_004086560.1 | flavodoxin | taxID used:135614 OG00206|WP\_023906848.1 | peptidase S15 | taxID used:135614 OG02071|WP\_010895221.1 | GNAT family N-acetyltransferase | taxID used:135614 OG02073|WP\_012382473.1 | DUF2127 domain-containing protein | taxID used:135614 OG02074|WP\_004083567.1 | type IV pilus modification protein PilV | taxID used:135614 OG02075|WP\_004083422.1 | Hsp20/alpha crystallin family protein | taxID used:135614 OG02076|WP\_010893991.1 | MarR family transcriptional regulator | taxID used:135614 OG02077|WP\_053014137.1 | hypothetical protein | taxID used:135614 OG02078|WP\_020852759.1 | hypothetical protein | taxID used:135614 OG02079|WP\_004089607.1 | endoribonuclease YbeY | taxID used:135614 OG00207|WP\_081089733.1 | phosphoribosylformylglycinamidine synthase | taxID used:135614 OG02080|WP\_046418777.1 | peroxiredoxin | taxID used:135614 OG02081|WP\_004087680.1 | CinA family protein | taxID used:135614 OG02082|WP\_060871794.1 | hypothetical protein | taxID used:135614 OG02083|WP\_020851923.1 | acetyl-CoA carboxylase biotin carboxyl carrier protein | taxID used:135614 OG02086|WP\_004087236.1 | DUF1566 domain-containing protein | taxID used:135614 OG02087|WP\_010893499.1 | phosphopantetheine adenylyltransferase | taxID used:135614 OG02088|WP\_010893019.1 | DUF1566 domain-containing protein | taxID used:135614 OG00208|WP\_038227902.1 | ligand-gated channel | taxID used:135614 OG02090|WP\_010892970.1 | ribosomal-protein-alanine N-acetyltransferase | taxID used:135614 OG02093|WP\_010893532.1 | manganese-binding transcriptional regulator MntR | taxID used:135614 OG02094|WP\_004088666.1 | peptidylprolyl isomerase | taxID used:135614 OG02095|WP\_020852698.1 | DUF1566 domain-containing protein | taxID used:135614 OG02096|WP\_010892578.1 | type IV pilus modification protein PilV | taxID used:135614 OG02097|WP\_004090587.1 | rod shape-determining protein MreD | taxID used:135614 OG02098|WP\_031336262.1 | hypothetical protein | taxID used:135614 OG02099|WP\_004088178.1 | peptide-methionine (R)-S-oxide reductase | taxID used:135614 OG00209|WP\_004083794.1 | cytochrome o ubiquinol oxidase subunit I | taxID used:135614 OG00020|WP\_031337140.1 | DNA-directed RNA polymerase subunit beta | taxID used:135614 OG02100|WP\_004086621.1 | 23S rRNA (pseudouridine(1915)-N(3))-methyltransferase RlmH | taxID used:135614 OG02101|WP\_027700668.1 | hypothetical protein | taxID used:135614 OG02102|WP\_038228352.1 | DUF2321 domain-containing protein | taxID used:135614 OG02108|WP\_046419166.1 | DUF1566 domain-containing protein | taxID used:135614 OG00210|WP\_071870001.1 | hypothetical protein | taxID used:135614 OG02111|WP\_031336885.1 | diguanylate cyclase | taxID used:135614 OG02114|WP\_023907979.1 | hypothetical protein | taxID used:135614 OG02115|WP\_004083722.1 | SET domain-containing protein-lysine N-methyltransferase | taxID used:135614 OG02118|WP\_004090295.1 | hypothetical protein | taxID used:135614 OG00211|WP\_042836615.1 | terminase | taxID used:135614 OG02120|WP\_031336929.1 | histidine phosphatase family protein | taxID used:135614 OG02121|WP\_038229263.1 | EVE domain-containing protein | taxID used:135614 OG02122|WP\_004084553.1 | tRNA (adenosine(37)-N6)-threonylcarbamoyltransferase complex ATPase subunit type 1 TsaE | taxID used:135614 OG02123|WP\_004086697.1 | conjugal transfer protein TrbH | taxID used:135614 OG02126|WP\_004089636.1 | DUF494 domain-containing protein | taxID used:135614 OG02128|WP\_024749174.1 | hypothetical protein | taxID used:135614 OG00212|WP\_058564579.1 | oligopeptidase A | taxID used:135614 OG02130|WP\_012382589.1 | hypothetical protein | taxID used:135614 OG02131|WP\_126715060.1 | hypothetical protein | taxID used:135614 OG02132|WP\_023906109.1 | hypothetical protein | taxID used:135614 OG02133|WP\_023907687.1 | hypothetical protein | taxID used:135614 OG02134|WP\_004085473.1 | tRNA (cytidine(34)-2'-O)-methyltransferase | taxID used:135614 OG02136|WP\_004572890.1 | hypothetical protein | taxID used:135614 OG02138|WP\_012338106.1 | GNAT family N-acetyltransferase | taxID used:135614 OG02139|WP\_004086409.1 | transcription antitermination factor NusB | taxID used:135614 OG00213|WP\_031336549.1 | methyl-accepting chemotaxis protein | taxID used:135614 OG02140|WP\_010895047.1 | 30S ribosomal protein S7 | taxID used:135614 OG02141|WP\_004085454.1 | chemotaxis protein CheW | taxID used:135614 OG02144|WP\_038231132.1 | hypothetical protein | taxID used:135614 OG02147|WP\_081089832.1 | hypothetical protein | taxID used:135614 OG02148|WP\_004086550.1 | hypothetical protein | taxID used:135614 OG02149|WP\_011097633.1 | transcription elongation factor GreA | taxID used:135614 OG00214|WP\_023908042.1 | ATP-dependent DNA helicase | taxID used:135614 OG02151|WP\_023907685.1 | hypothetical protein | taxID used:135614 OG02153|WP\_080939601.1 | hypothetical protein | taxID used:135614 OG02154|WP\_010894248.1 | single-stranded DNA-binding protein | taxID used:135614 OG02155|WP\_004086121.1 | ribonuclease HI | taxID used:135614 OG02156|WP\_038227621.1 | hypothetical protein | taxID used:135614 OG02158|WP\_010893664.1 | F0F1 ATP synthase subunit B | taxID used:135614 OG02160|WP\_004086411.1 | 6,7-dimethyl-8-ribityllumazine synthase | taxID used:135614 OG02161|WP\_024748779.1 | cytochrome c biogenesis protein CcmE | taxID used:135614 OG02162|WP\_010894300.1 | thioredoxin family protein | taxID used:135614 OG02164|WP\_004090375.1 | pilin | taxID used:135614 OG02165|WP\_058565010.1 | DUF1566 domain-containing protein | taxID used:135614 OG02166|WP\_004083417.1 | Holliday junction resolvase RuvX | taxID used:135614 OG02167|WP\_010892925.1 | bacterioferritin | taxID used:135614 OG02168|WP\_072866346.1 | single-stranded DNA-binding protein | taxID used:135614 OG02171|WP\_004083563.1 | type IV pilin protein | taxID used:135614 OG02173|WP\_058564932.1 | azurin | taxID used:135614 OG02177|WP\_004083752.1 | NUDIX hydrolase | taxID used:135614 OG02179|WP\_011097939.1 | hypothetical protein | taxID used:135614 OG02180|WP\_060870276.1 | hypothetical protein | taxID used:135614 OG02181|WP\_049756312.1 | DUF4065 domain-containing protein, partial | taxID used:135614 OG02182|WP\_031336766.1 | SUF system Fe-S cluster assembly regulator | taxID used:135614 OG02183|WP\_042462889.1 | hypothetical protein | taxID used:135614 OG02184|WP\_010894755.1 | hypothetical protein | taxID used:135614 OG02185|WP\_058565250.1 | hypothetical protein | taxID used:135614 OG02186|WP\_024749125.1 | hypothetical protein | taxID used:135614 OG02187|WP\_038230185.1 | hypothetical protein | taxID used:135614 OG02188|WP\_004087579.1 | dUTP diphosphatase | taxID used:135614 OG02189|WP\_024748697.1 | hypothetical protein | taxID used:135614 OG00218|WP\_038229932.1 | excinuclease ABC subunit UvrB | taxID used:135614 OG02191|WP\_010894521.1 | hypothetical protein | taxID used:135614 OG02193|WP\_010895248.1 | hypothetical protein | taxID used:135614 OG02195|WP\_004088329.1 | hypothetical protein | taxID used:135614 OG02199|WP\_010895255.1 | hypothetical protein | taxID used:135614 OG00021|WP\_080715122.1 | hemagglutinin, partial | taxID used:135614 OG02202|WP\_010894046.1 | glycine zipper 2TM domain-containing protein | taxID used:135614 OG02203|WP\_072866399.1 | single-stranded DNA-binding protein | taxID used:135614 OG02204|WP\_058565232.1 | single-stranded DNA-binding protein | taxID used:135614 OG02206|WP\_020851432.1 | CopD family protein | taxID used:135614 OG02207|WP\_038211276.1 | transcriptional regulator MraZ | taxID used:135614 OG02208|WP\_004085838.1 | type II toxin-antitoxin system RatA family toxin | taxID used:135614 OG02209|WP\_020851983.1 | SCO family protein | taxID used:135614 OG02210|WP\_004091442.1 | conjugal transfer protein TraM | taxID used:135614 OG02213|WP\_010893996.1 | DUF192 domain-containing protein | taxID used:135614 OG02214|WP\_020852400.1 | single-stranded DNA-binding protein | taxID used:135614 OG02216|WP\_010894960.1 | pilin | taxID used:135614 OG02217|WP\_038211361.1 | hypothetical protein | taxID used:135614 OG02218|WP\_081089744.1 | phage integrase | taxID used:135614 OG02219|WP\_057682412.1 | phosphotransferase | taxID used:135614 OG00221|WP\_046420843.1 | DNA helicase Rep | taxID used:135614 OG02221|WP\_058570083.1 | DUF4065 domain-containing protein | taxID used:135614 OG02222|WP\_020852035.1 | guanine-specific ribonuclease N1 and T1 | taxID used:135614 OG02223|WP\_058569726.1 | hypothetical protein | taxID used:135614 OG02227|WP\_076613232.1 | hypothetical protein | taxID used:135614 OG02228|WP\_004086143.1 | ribosome silencing factor | taxID used:135614 OG02229|WP\_082355755.1 | acetyl-CoA carboxylase biotin carboxyl carrier protein subunit | taxID used:135614 OG00222|WP\_060870229.1 | conjugal transfer protein | taxID used:135614 OG02233|WP\_024749309.1 | transcriptional regulator | taxID used:135614 OG02234|WP\_052262867.1 | hypothetical protein | taxID used:135614 OG02235|WP\_038227931.1 | GatB/YqeY domain-containing protein | taxID used:135614 OG02237|WP\_069107128.1 | hypothetical protein | taxID used:135614 OG00223|WP\_057682498.1 | bifunctional sulfate adenylyltransferase subunit 1/adenylylsulfate kinase | taxID used:135614 OG02240|WP\_020852340.1 | Fe/S cluster cysteine desulfuration protein | taxID used:135614 OG02241|WP\_004090438.1 | YeeE/YedE family protein | taxID used:135614 OG02243|WP\_038211800.1 | hypothetical protein | taxID used:135614 OG02244|WP\_023906152.1 | hypothetical protein | taxID used:135614 OG02245|WP\_004572855.1 | VirK protein | taxID used:135614 OG02246|WP\_023907230.1 | transcriptional regulator | taxID used:135614 OG02247|WP\_027700163.1 | DUF721 domain-containing protein | taxID used:135614 OG02249|WP\_081089898.1 | hypothetical protein | taxID used:135614 OG00224|WP\_088372189.1 | M1 family peptidase | taxID used:135614 OG02250|WP\_057683326.1 | cytochrome c-type biogenesis protein CcmH | taxID used:135614 OG02251|WP\_021358549.1 | type II 3-dehydroquinate dehydratase | taxID used:135614 OG02254|WP\_004083857.1 | MarR family transcriptional regulator | taxID used:135614 OG02255|WP\_031337016.1 | ClpXP protease specificity-enhancing factor | taxID used:135614 OG02257|WP\_040123234.1 | hypothetical protein | taxID used:135614 OG02259|WP\_011097876.1 | DUF3277 domain-containing protein | taxID used:135614 OG00225|WP\_027700094.1 | acetate--CoA ligase | taxID used:135614 OG02260|WP\_004090345.1 | 30S ribosomal protein S6 | taxID used:135614 OG02261|WP\_004087249.1 | DUF3277 family protein | taxID used:135614 OG02264|WP\_010894980.1 | 50S ribosomal protein L9 | taxID used:135614 OG02265|WP\_031336933.1 | succinate dehydrogenase, cytochrome b556 subunit | taxID used:135614 OG02266|WP\_060870434.1 | hypothetical protein | taxID used:135614 OG02267|WP\_004084894.1 | rhodanese-like domain-containing protein | taxID used:135614 OG02269|WP\_057683058.1 | NfeD family protein | taxID used:135614 OG00226|WP\_023907661.1 | integrase | taxID used:135614 OG02272|WP\_004083686.1 | type II secretion system protein GspG | taxID used:135614 OG02274|WP\_058569577.1 | DNA polymerase III subunit chi | taxID used:135614 OG02275|WP\_031336902.1 | 50S ribosomal protein L15 | taxID used:135614 OG02276|WP\_004089267.1 | biopolymer transporter ExbD | taxID used:135614 OG02277|WP\_069107115.1 | phage-related protein | taxID used:135614 OG00227|WP\_058564252.1 | lytic murein transglycosylase | taxID used:135614 OG02280|WP\_011097832.1 | protein TolR | taxID used:135614 OG02281|WP\_031336780.1 | fluoride efflux transporter CrcB | taxID used:135614 OG02282|WP\_031336563.1 | tol-pal system-associated acyl-CoA thioesterase | taxID used:135614 OG02285|WP\_027700190.1 | polymer-forming cytoskeletal family protein | taxID used:135614 OG02286|WP\_024749188.1 | DUF2335 domain-containing protein | taxID used:135614 OG02287|WP\_004091423.1 | antirestriction protein | taxID used:135614 OG02288|WP\_010894458.1 | hypothetical protein | taxID used:135614 OG00228|WP\_038231298.1 | phospholipase D family protein | taxID used:135614 OG02290|WP\_004087199.1 | hypothetical protein | taxID used:135614 OG02291|WP\_080679637.1 | hypothetical protein | taxID used:135614 OG02292|WP\_046417602.1 | Tfp pilus assembly protein pilE | taxID used:135614 OG02293|WP\_004083669.1 | 50S ribosomal protein L13 | taxID used:135614 OG02296|WP\_088577928.1 | PIN domain-containing protein | taxID used:135614 OG02297|WP\_010894293.1 | organic hydroperoxide resistance protein | taxID used:135614 OG02299|WP\_004087842.1 | biopolymer transporter ExbD | taxID used:135614 OG00229|WP\_004086556.1 | peptidyl-prolyl cis-trans isomerase | taxID used:135614 OG02301|WP\_004088772.1 | twin-arginine translocase subunit TatB | taxID used:135614 OG02302|WP\_038211798.1 | hypothetical protein | taxID used:135614 OG02303|WP\_058565248.1 | hypothetical protein | taxID used:135614 OG02307|WP\_076613624.1 | hypothetical protein | taxID used:135614 OG02309|WP\_010892927.1 | response regulator | taxID used:135614 OG00230|WP\_046419217.1 | head completion protein | taxID used:135614 OG02310|WP\_027700564.1 | 50S ribosomal protein L11 | taxID used:135614 OG02314|WP\_027700314.1 | GtrA family protein | taxID used:135614 OG02315|WP\_126709131.1 | hypothetical protein | taxID used:135614 OG02318|WP\_004083578.1 | nucleoside-diphosphate kinase | taxID used:135614 OG02319|WP\_076613213.1 | integrase | taxID used:135614 OG00231|WP\_004084578.1 | threonine--tRNA ligase | taxID used:135614 OG02320|WP\_004085082.1 | biopolymer transporter ExbD | taxID used:135614 OG02322|WP\_004083709.1 | DNA-binding protein H-NS | taxID used:135614 OG02323|WP\_012382712.1 | hypothetical protein | taxID used:135614 OG02324|WP\_060872304.1 | recombinase family protein | taxID used:135614 OG02326|WP\_057683716.1 | hypothetical protein | taxID used:135614 OG02327|WP\_038228391.1 | hypothetical protein | taxID used:135614 OG02328|WP\_010895253.1 | Single-stranded DNA-binding protein 1 | taxID used:135614 OG02329|WP\_020851103.1 | putative toxin-antitoxin system toxin component, PIN family | taxID used:135614 OG00232|WP\_010892715.1 | DUF885 domain-containing protein | taxID used:135614 OG02330|WP\_057682357.1 | energy transducer TonB | taxID used:135614 OG02332|WP\_011098037.1 | DUF4845 domain-containing protein | taxID used:135614 OG02333|WP\_060871667.1 | hypothetical protein | taxID used:135614 OG02335|WP\_058565210.1 | DUF1566 domain-containing protein | taxID used:135614 OG02336|WP\_004086531.1 | 50S ribosomal protein L16 | taxID used:135614 OG02337|WP\_031345921.1 | hypothetical protein | taxID used:135614 OG02338|WP\_004090067.1 | F0F1 ATP synthase subunit epsilon | taxID used:135614 OG02339|WP\_010894229.1 | hypothetical protein | taxID used:135614 OG00233|WP\_020850921.1 | transglutaminase | taxID used:135614 OG02340|WP\_020852034.1 | hypothetical protein | taxID used:135614 OG02341|WP\_118853409.1 | hypothetical protein | taxID used:135614 OG02342|WP\_004087234.1 | hypothetical protein | taxID used:135614 OG02343|WP\_004085459.1 | response regulator | taxID used:135614 OG02347|WP\_004086588.1 | MerC domain-containing protein | taxID used:135614 OG02349|WP\_046419512.1 | antibiotic resistance protein VanZ | taxID used:135614 OG00234|WP\_027700375.1 | ATP-dependent zinc metalloprotease FtsH | taxID used:135614 OG02350|WP\_011098077.1 | ferric iron uptake transcriptional regulator | taxID used:135614 OG02351|WP\_021358177.1 | DUF4398 domain-containing protein | taxID used:135614 OG02352|WP\_020852461.1 | DNA-binding protein H-NS | taxID used:135614 OG02356|WP\_004083648.1 | hypothetical protein | taxID used:135614 OG02358|WP\_010893215.1 | hypothetical protein | taxID used:135614 OG02359|WP\_010895054.1 | preprotein translocase subunit SecE | taxID used:135614 OG00235|WP\_004088280.1 | oligopeptide transporter, OPT family | taxID used:135614 OG02360|WP\_020852214.1 | hypothetical protein | taxID used:135614 OG02361|WP\_023906384.1 | hypothetical protein | taxID used:135614 OG02362|WP\_010892930.1 | hypothetical protein | taxID used:135614 OG02363|WP\_010894565.1 | hypothetical protein | taxID used:135614 OG02365|WP\_023906903.1 | hypothetical protein | taxID used:135614 OG02366|WP\_075584703.1 | ribonuclease P protein component | taxID used:135614 OG02367|WP\_072866385.1 | DUF1566 domain-containing protein | taxID used:135614 OG02368|WP\_081089728.1 | helix-turn-helix domain-containing protein | taxID used:135614 OG00236|WP\_004086013.1 | heme lyase CcmF/NrfE family subunit | taxID used:135614 OG02374|WP\_004085540.1 | 50S ribosomal protein L19 | taxID used:135614 OG02377|WP\_046419214.1 | DNA stabilization protein | taxID used:135614 OG02378|WP\_060871772.1 | hypothetical protein | taxID used:135614 OG00237|WP\_057683503.1 | ABC transporter ATP-binding protein | taxID used:135614 OG00238|WP\_010894369.1 | potassium transporter Kup | taxID used:135614 OG02393|WP\_004083684.1 | prepilin-type N-terminal cleavage/methylation domain-containing protein | taxID used:135614 OG02395|WP\_010894242.1 | hypothetical protein | taxID used:135614 OG02399|WP\_011097555.1 | type II toxin-antitoxin system VapC family toxin | taxID used:135614 OG00239|WP\_024749213.1 | glucans biosynthesis glucosyltransferase MdoH | taxID used:135614 OG00023|WP\_011097732.1 | phosphoribosylformylglycinamidine synthase | taxID used:135614 OG02400|WP\_011098322.1 | response regulator | taxID used:135614 OG02401|WP\_004085841.1 | outer membrane protein assembly factor BamE | taxID used:135614 OG02402|WP\_010894423.1 | YkgJ family cysteine cluster protein | taxID used:135614 OG02405|WP\_004085069.1 | large-conductance mechanosensitive channel protein MscL | taxID used:135614 OG02407|WP\_010892724.1 | glycine cleavage system protein GcvH | taxID used:135614 OG02408|WP\_004086543.1 | 30S ribosomal protein S11 | taxID used:135614 OG00240|WP\_046418193.1 | polysaccharide biosynthesis protein | taxID used:135614 OG02410|WP\_004087939.1 | preprotein translocase subunit SecG | taxID used:135614 OG02414|WP\_038274572.1 | hypothetical protein | taxID used:135614 OG02416|WP\_004086536.1 | 30S ribosomal protein S8 | taxID used:135614 OG02417|WP\_004091439.1 | hypothetical protein | taxID used:135614 OG02418|WP\_010893736.1 | hypothetical protein | taxID used:135614 OG00241|WP\_046418948.1 | phosphogluconate dehydratase | taxID used:135614 OG02423|WP\_080654482.1 | hypothetical protein | taxID used:135614 OG02428|WP\_011097604.1 | hypothetical protein | taxID used:135614 OG02429|WP\_004089538.1 | VOC family protein | taxID used:135614 OG00242|WP\_060872172.1 | molecular chaperone HtpG | taxID used:135614 OG02431|WP\_004086104.1 | Co2+/Mg2+ efflux protein ApaG | taxID used:135614 OG02432|WP\_010892821.1 | hypothetical protein | taxID used:135614 OG02438|WP\_010894078.1 | hypothetical protein | taxID used:135614 OG02439|WP\_088569742.1 | hypothetical protein | taxID used:135614 OG00243|WP\_004083434.1 | 1-deoxy-D-xylulose-5-phosphate synthase | taxID used:135614 OG02441|WP\_021358622.1 | hypothetical protein | taxID used:135614 OG02443|WP\_023907974.1 | hypothetical protein | taxID used:135614 OG02444|WP\_004083670.1 | 30S ribosomal protein S9 | taxID used:135614 OG02447|WP\_071869957.1 | hypothetical protein, partial | taxID used:135614 OG02449|WP\_004083781.1 | PTS fructose IIA subunit family protein | taxID used:135614 OG00244|WP\_057682736.1 | type IV pilus secretin PilQ | taxID used:135614 OG02457|WP\_046420072.1 | DUF4065 domain-containing protein | taxID used:135614 OG02458|WP\_004090932.1 | DUF596 domain-containing protein | taxID used:135614 OG00245|WP\_023907200.1 | arginine decarboxylase | taxID used:135614 OG02462|WP\_020852637.1 | hypothetical protein | taxID used:135614 OG02464|WP\_004083584.1 | response regulator | taxID used:135614 OG00246|WP\_058564962.1 | tRNA uridine-5-carboxymethylaminomethyl(34) synthesis enzyme MnmG | taxID used:135614 OG02471|WP\_042836702.1 | hypothetical protein | taxID used:135614 OG02472|WP\_046417535.1 | conjugal transfer protein | taxID used:135614 OG02473|WP\_010892935.1 | iron-sulfur cluster insertion protein ErpA | taxID used:135614 OG02474|WP\_046420352.1 | hypothetical protein | taxID used:135614 OG00247|WP\_057683363.1 | DNA topoisomerase IV subunit B | taxID used:135614 OG02480|WP\_004085448.1 | DNA-binding protein | taxID used:135614 OG02482|WP\_010893689.1 | 50S ribosomal protein L17 | taxID used:135614 OG02483|WP\_004089926.1 | RidA family protein | taxID used:135614 OG02485|WP\_004086272.1 | aspartate 1-decarboxylase | taxID used:135614 OG02487|WP\_076613270.1 | hypothetical protein | taxID used:135614 OG00248|WP\_004084772.1 | signal peptide peptidase SppA | taxID used:135614 OG02490|WP\_004085491.1 | XRE family transcriptional regulator | taxID used:135614 OG02491|WP\_010892919.1 | hypothetical protein | taxID used:135614 OG02492|WP\_023907996.1 | hypothetical protein | taxID used:135614 OG02494|WP\_004085551.1 | hypothetical protein | taxID used:135614 OG02495|WP\_004088353.1 | hypothetical protein | taxID used:135614 OG02496|WP\_020851197.1 | DUF4124 domain-containing protein | taxID used:135614 OG02497|WP\_010892777.1 | 30S ribosome-binding factor RbfA | taxID used:135614 OG02499|WP\_004085860.1 | diacylglycerol kinase | taxID used:135614 OG00024|WP\_058564510.1 | TIGR02099 family protein | taxID used:135614 OG02502|WP\_004089956.1 | hypothetical protein | taxID used:135614 OG02503|WP\_081392428.1 | DUF86 domain-containing protein | taxID used:135614 OG02506|WP\_010894076.1 | DUF4054 domain-containing protein | taxID used:135614 OG02507|WP\_004084687.1 | 30S ribosomal protein S12 | taxID used:135614 OG02508|WP\_046419779.1 | hypothetical protein | taxID used:135614 OG02509|WP\_010893590.1 | succinate dehydrogenase, hydrophobic membrane anchor protein | taxID used:135614 OG00250|WP\_046420641.1 | molecular chaperone DnaK | taxID used:135614 OG02511|WP\_010895203.1 | conjugal transfer protein | taxID used:135614 OG02512|WP\_004086459.1 | XRE family transcriptional regulator | taxID used:135614 OG02514|WP\_004083551.1 | YraN family protein | taxID used:135614 OG02516|WP\_024748713.1 | hypothetical protein | taxID used:135614 OG02519|WP\_058565258.1 | DUF4054 domain-containing protein | taxID used:135614 OG00251|WP\_057682762.1 | exodeoxyribonuclease V subunit alpha | taxID used:135614 OG02520|WP\_011097845.1 | DUF2523 domain-containing protein | taxID used:135614 OG02521|WP\_010892975.1 | DNA-binding protein | taxID used:135614 OG02522|WP\_004084442.1 | hypothetical protein | taxID used:135614 OG02523|WP\_031336173.1 | 6-carboxytetrahydropterin synthase QueD | taxID used:135614 OG02525|WP\_023907976.1 | DUF4054 domain-containing protein | taxID used:135614 OG02526|WP\_004091494.1 | single-stranded DNA-binding protein | taxID used:135614 OG02527|WP\_058570079.1 | N-acetyltransferase | taxID used:135614 OG02530|WP\_042463672.1 | hypothetical protein | taxID used:135614 OG02535|WP\_031336149.1 | Spx/MgsR family RNA polymerase-binding regulatory protein | taxID used:135614 OG02536|WP\_012382427.1 | preprotein translocase subunit YajC | taxID used:135614 OG02537|WP\_004086533.1 | 50S ribosomal protein L14 | taxID used:135614 OG02538|WP\_010892965.1 | dihydroneopterin aldolase | taxID used:135614 OG00253|WP\_004087476.1 | alpha-L-fucosidase | taxID used:135614 OG02542|WP\_058565186.1 | hypothetical protein | taxID used:135614 OG02543|WP\_004091455.1 | conjugal transfer protein TraO | taxID used:135614 OG02544|WP\_080702481.1 | hypothetical protein | taxID used:135614 OG02546|WP\_011097688.1 | cell division protein FtsB | taxID used:135614 OG02547|WP\_010893263.1 | 50S ribosomal protein L20 | taxID used:135614 OG02548|WP\_010895022.1 | 4a-hydroxytetrahydrobiopterin dehydratase | taxID used:135614 OG00254|WP\_058564582.1 | copper resistance system multicopper oxidase | taxID used:135614 OG02553|WP\_011097560.1 | NADH-quinone oxidoreductase subunit A | taxID used:135614 OG02555|WP\_004085637.1 | hypothetical protein | taxID used:135614 OG02556|WP\_038229204.1 | hypothetical protein | taxID used:135614 OG02558|WP\_004086542.1 | 30S ribosomal protein S13 | taxID used:135614 OG00255|WP\_057682439.1 | beta-galactosidase | taxID used:135614 OG02561|WP\_020852228.1 | histidine triad nucleotide-binding protein | taxID used:135614 OG02563|WP\_004083585.1 | DUF486 domain-containing protein | taxID used:135614 OG02566|WP\_004087000.1 | hypothetical protein | taxID used:135614 OG02568|WP\_012337848.1 | hypothetical protein | taxID used:135614 OG02569|WP\_014607493.1 | MerR family transcriptional regulator | taxID used:135614 OG02570|WP\_010894499.1 | hypothetical protein | taxID used:135614 OG02571|WP\_011097986.1 | hypothetical protein | taxID used:135614 OG02572|WP\_023907309.1 | DUF2190 domain-containing protein | taxID used:135614 OG02574|WP\_060872334.1 | hypothetical protein | taxID used:135614 OG02576|WP\_023906519.1 | hypothetical protein | taxID used:135614 OG02577|WP\_010892823.1 | hypothetical protein | taxID used:135614 OG02578|WP\_081033438.1 | helix-turn-helix domain-containing protein | taxID used:135614 OG02579|WP\_012382841.1 | transcriptional regulator | taxID used:135614 OG00257|WP\_038228283.1 | excinuclease ABC subunit UvrC | taxID used:135614 OG02581|WP\_004083467.1 | type IV pilus assembly PilZ | taxID used:135614 OG02582|WP\_046419262.1 | hypothetical protein | taxID used:135614 OG02583|WP\_010895050.1 | 50S ribosomal protein L7/L12 | taxID used:135614 OG02584|WP\_060870091.1 | hypothetical protein | taxID used:135614 OG02589|WP\_080679731.1 | hypothetical protein | taxID used:135614 OG00258|WP\_038228562.1 | ABC transporter ATP-binding protein | taxID used:135614 OG02590|WP\_085808101.1 | hypothetical protein | taxID used:135614 OG02591|WP\_010894235.1 | DUF3085 domain-containing protein | taxID used:135614 OG02593|WP\_031337234.1 | toxin RelE | taxID used:135614 OG02594|WP\_016024091.1 | hypothetical protein | taxID used:135614 OG02596|WP\_004086538.1 | 50S ribosomal protein L18 | taxID used:135614 OG02597|WP\_057683540.1 | conjugal transfer protein TraJ | taxID used:135614 OG02598|WP\_004089237.1 | cytochrome o ubiquinol oxidase subunit IV | taxID used:135614 OG02599|WP\_010894010.1 | PilZ domain-containing protein | taxID used:135614 OG00259|WP\_014607668.1 | RNA polymerase sigma factor RpoD | taxID used:135614 OG00025|WP\_088578274.1 | translocation/assembly module TamB | taxID used:135614 OG02601|WP\_004084923.1 | RidA family protein | taxID used:135614 OG02603|WP\_020852709.1 | thioredoxin | taxID used:135614 OG02605|WP\_046419917.1 | hypothetical protein | taxID used:135614 OG02607|WP\_004089069.1 | non-heme iron oxygenase ferredoxin subunit | taxID used:135614 OG02609|WP\_060872048.1 | DUF2523 domain-containing protein | taxID used:135614 OG00260|WP\_004085004.1 | hypothetical protein | taxID used:135614 OG02610|WP\_046419644.1 | type II toxin-antitoxin system RelE/ParE family toxin | taxID used:135614 OG02611|WP\_004084639.1 | iron-sulfur cluster assembly accessory protein | taxID used:135614 OG02616|WP\_010893387.1 | hypothetical protein | taxID used:135614 OG02617|WP\_004083506.1 | divalent-cation tolerance protein CutA | taxID used:135614 OG02618|WP\_004085696.1 | hypothetical protein | taxID used:135614 OG02619|WP\_020851391.1 | nitrogen regulatory protein P-II 1 | taxID used:135614 OG00261|WP\_057682299.1 | hypothetical protein | taxID used:135614 OG02622|WP\_080507235.1 | hypothetical protein | taxID used:135614 OG02624|WP\_004088312.1 | Trp operon repressor | taxID used:135614 OG02628|WP\_004084667.1 | ferredoxin family protein | taxID used:135614 OG02629|WP\_023906441.1 | hypothetical protein | taxID used:135614 OG00262|WP\_004084881.1 | glycosyltransferase family 39 protein | taxID used:135614 OG02630|WP\_126709107.1 | hypothetical protein | taxID used:135614 OG02634|WP\_012382839.1 | protein kleE | taxID used:135614 OG02635|WP\_072866418.1 | phage baseplate protein | taxID used:135614 OG00263|WP\_004090429.1 | DNA mismatch repair protein MutL | taxID used:135614 OG02640|WP\_010894230.1 | hypothetical protein | taxID used:135614 OG02642|WP\_021358671.1 | phage coat protein | taxID used:135614 OG02648|WP\_004572981.1 | J domain-containing protein | taxID used:135614 OG02649|WP\_010894885.1 | hypothetical protein | taxID used:135614 OG00264|WP\_011097989.1 | phage-related protein | taxID used:135614 OG02650|WP\_058564947.1 | DNA-binding transcriptional regulator | taxID used:135614 OG02654|WP\_057683533.1 | conjugal transfer protein TrbD | taxID used:135614 OG02657|WP\_004089713.1 | DNA-binding transcriptional regulator | taxID used:135614 OG02659|WP\_038211662.1 | hypothetical protein | taxID used:135614 OG00265|WP\_060870104.1 | endoglucanase | taxID used:135614 OG02660|WP\_046420266.1 | DNA-binding transcriptional regulator | taxID used:135614 OG02661|WP\_060872195.1 | hypothetical protein | taxID used:135614 OG02662|WP\_020853027.1 | IS200/IS605 family transposase | taxID used:135614 OG02667|WP\_010893673.1 | 50S ribosomal protein L22 | taxID used:135614 OG02668|WP\_081392198.1 | integrase | taxID used:135614 OG00266|WP\_057682414.1 | SLC13 family permease | taxID used:135614 OG02670|WP\_004083751.1 | ATP-dependent Clp protease adapter ClpS | taxID used:135614 OG02675|WP\_126715059.1 | hypothetical protein | taxID used:135614 OG02678|WP\_126715057.1 | DUF4224 domain-containing protein | taxID used:135614 OG02682|WP\_004091396.1 | DNA-binding protein | taxID used:135614 OG02685|WP\_060872277.1 | DNA-binding protein | taxID used:135614 OG02688|WP\_010893574.1 | DUF1820 family protein | taxID used:135614 OG02689|WP\_004083778.1 | ribosome-associated translation inhibitor RaiA | taxID used:135614 OG00268|WP\_046418733.1 | ferrous iron transporter B | taxID used:135614 OG02691|WP\_031338015.1 | DNA-binding protein | taxID used:135614 OG02693|WP\_004091397.1 | type II toxin-antitoxin system RelE/ParE family toxin | taxID used:135614 OG02696|WP\_060871452.1 | hypothetical protein | taxID used:135614 OG02697|WP\_004088703.1 | DNA uptake protein | taxID used:135614 OG00269|WP\_004087662.1 | dihydroxy-acid dehydratase | taxID used:135614 OG00026|WP\_038227928.1 | UvrD/REP helicase | taxID used:135614 OG02702|WP\_004086561.1 | thioredoxin | taxID used:135614 OG02705|WP\_027700521.1 | DNA-binding protein | taxID used:135614 OG02706|WP\_081089873.1 | hypothetical protein | taxID used:135614 OG02707|WP\_060870258.1 | type II toxin-antitoxin system PemK/MazF family toxin | taxID used:135614 OG00270|WP\_004089012.1 | assimilatory sulfite reductase (NADPH) flavoprotein subunit | taxID used:135614 OG02714|WP\_004088409.1 | 50S ribosomal protein L21 | taxID used:135614 OG02715|WP\_020851063.1 | DUF4156 domain-containing protein | taxID used:135614 OG02717|WP\_010893044.1 | hypothetical protein | taxID used:135614 OG00271|WP\_004084485.1 | penicillin-binding protein 2 | taxID used:135614 OG02720|WP\_004088463.1 | BolA family transcriptional regulator | taxID used:135614 OG02721|WP\_042464056.1 | hypothetical protein | taxID used:135614 OG02722|WP\_004572794.1 | YbaB/EbfC family nucleoid-associated protein | taxID used:135614 OG02727|WP\_118853455.1 | integrase | taxID used:135614 OG00272|WP\_042462625.1 | DUF885 family protein | taxID used:135614 OG02731|WP\_027700095.1 | DUF485 domain-containing protein | taxID used:135614 OG02732|WP\_053014131.1 | hypothetical protein | taxID used:135614 OG02733|WP\_031336866.1 | type II toxin-antitoxin system PemK/MazF family toxin | taxID used:135614 OG02738|WP\_031336996.1 | DNA-binding protein | taxID used:135614 OG02739|WP\_004086577.1 | 50S ribosomal protein L24 | taxID used:135614 OG00273|WP\_038232083.1 | single-stranded-DNA-specific exonuclease RecJ | taxID used:135614 OG02746|WP\_004084751.1 | Grx4 family monothiol glutaredoxin | taxID used:135614 OG00274|WP\_010894354.1 | phosphomethylpyrimidine synthase ThiC | taxID used:135614 OG02751|WP\_004087527.1 | glutaredoxin 3 | taxID used:135614 OG02753|WP\_088371405.1 | hypothetical protein | taxID used:135614 OG02756|WP\_004085973.1 | integration host factor subunit beta | taxID used:135614 OG02758|WP\_010893917.1 | EF-hand domain-containing protein | taxID used:135614 OG00275|WP\_004090207.1 | translational GTPase TypA | taxID used:135614 OG02764|WP\_060870052.1 | type II toxin-antitoxin system RelE/ParE family toxin | taxID used:135614 OG02766|WP\_010894519.1 | type II toxin-antitoxin system RelE/ParE family toxin | taxID used:135614 OG00276|WP\_023907197.1 | glutamine--fructose-6-phosphate aminotransferase | taxID used:135614 OG02770|WP\_004090086.1 | 30S ribosomal protein S10 | taxID used:135614 OG02776|WP\_010894460.1 | DUF1631 family protein | taxID used:135614 OG02778|WP\_031345883.1 | hypothetical protein | taxID used:135614 OG02779|WP\_004091293.1 | hypothetical protein | taxID used:135614 OG00277|WP\_012382432.1 | RNA helicase | taxID used:135614 OG02782|WP\_004089684.1 | hypothetical protein | taxID used:135614 OG02783|WP\_011097842.1 | hypothetical protein | taxID used:135614 OG02788|WP\_081033573.1 | hypothetical protein | taxID used:135614 OG02789|WP\_038231486.1 | transcriptional regulator | taxID used:135614 OG00278|WP\_012337750.1 | DNA helicase RecQ | taxID used:135614 OG02791|WP\_010895217.1 | hypothetical protein | taxID used:135614 OG02792|WP\_046418838.1 | DUF4190 domain-containing protein | taxID used:135614 OG02794|WP\_004086535.1 | 30S ribosomal protein S14 | taxID used:135614 OG02795|WP\_004085585.1 | plasmid maintenance system killer | taxID used:135614 OG02796|WP\_004084837.1 | hypothetical protein | taxID used:135614 OG00279|WP\_038229515.1 | protein translocase subunit SecD | taxID used:135614 OG00027|WP\_088577583.1 | type IV pilus biogenesis factor PilY | taxID used:135614 OG02802|WP\_058569559.1 | YhbY family RNA-binding protein | taxID used:135614 OG02807|WP\_046417543.1 | transcriptional regulator | taxID used:135614 OG00280|WP\_010894275.1 | DNA polymerase III subunit gamma/tau | taxID used:135614 OG02812|WP\_004089254.1 | putative addiction module antidote protein | taxID used:135614 OG02813|WP\_060872163.1 | hypothetical protein | taxID used:135614 OG02814|WP\_004085003.1 | putative addiction module antidote protein | taxID used:135614 OG02817|WP\_060871756.1 | type II toxin-antitoxin system RelE/ParE family toxin | taxID used:135614 OG02819|WP\_060872108.1 | type II toxin-antitoxin system RelE/ParE family toxin | taxID used:135614 OG00281|WP\_010893591.1 | succinate dehydrogenase flavoprotein subunit | taxID used:135614 OG02824|WP\_010894527.1 | type II toxin-antitoxin system RelE/ParE family toxin | taxID used:135614 OG02827|WP\_010892947.1 | STAS domain-containing protein | taxID used:135614 OG02828|WP\_012337685.1 | 50S ribosomal protein L23 | taxID used:135614 OG02830|WP\_010894003.1 | DNA-directed RNA polymerase subunit omega | taxID used:135614 OG02831|WP\_004087025.1 | addiction module antidote protein, HigA family | taxID used:135614 OG02833|WP\_004086434.1 | hypothetical protein | taxID used:135614 OG02835|WP\_004089519.1 | addiction module antidote protein, HigA family | taxID used:135614 OG02837|WP\_010894925.1 | type II toxin-antitoxin system MqsR family toxin | taxID used:135614 OG00283|WP\_088372274.1 | formylglycine-generating enzyme family protein | taxID used:135614 OG02842|WP\_080507217.1 | LapA family protein | taxID used:135614 OG02844|WP\_004089989.1 | DUF2782 domain-containing protein | taxID used:135614 OG00284|WP\_058564391.1 | gamma-glutamyltransferase | taxID used:135614 OG02850|WP\_004084568.1 | integration host factor subunit alpha | taxID used:135614 OG02851|WP\_004085382.1 | cell division protein ZapA | taxID used:135614 OG02855|WP\_046419679.1 | type II toxin-antitoxin system RelE/ParE family toxin | taxID used:135614 OG00285|WP\_057682928.1 | elongation factor 4 | taxID used:135614 OG02861|WP\_020851042.1 | NADH-quinone oxidoreductase subunit NuoK | taxID used:135614 OG02870|WP\_004087908.1 | YfhL family 4Fe-4S dicluster ferredoxin | taxID used:135614 OG02879|WP\_004089716.1 | topoisomerase I | taxID used:135614 OG00287|WP\_004084501.1 | autotransporter domain-containing esterase | taxID used:135614 OG02880|WP\_024748689.1 | F0F1 ATP synthase subunit C | taxID used:135614 OG02884|WP\_004083921.1 | hypothetical protein | taxID used:135614 OG02885|WP\_011098058.1 | plasmid maintenance system killer | taxID used:135614 OG00288|WP\_060870112.1 | dihydrolipoyl dehydrogenase | taxID used:135614 OG02895|WP\_004090455.1 | beta-hydroxyacyl-ACP dehydratase | taxID used:135614 OG00289|WP\_038211007.1 | TspA protein | taxID used:135614 OG00028|WP\_080939572.1 | pilus assembly protein | taxID used:135614 OG02901|WP\_023906601.1 | uridylate kinase | taxID used:135614 OG02907|WP\_023906865.1 | membrane protein insertion efficiency factor YidD | taxID used:135614 OG02908|WP\_004090695.1 | type II toxin-antitoxin system RelE/ParE family toxin | taxID used:135614 OG00290|WP\_010895176.1 | hypothetical protein | taxID used:135614 OG02917|WP\_024748648.1 | DUF1566 domain-containing protein | taxID used:135614 OG00291|WP\_081090401.1 | phosphoribosylformylglycinamidine synthase, partial | taxID used:135614 OG02921|WP\_010894528.1 | ribbon-helix-helix protein, CopG family | taxID used:135614 OG02927|WP\_010895222.1 | DUF4190 domain-containing protein | taxID used:135614 OG02928|WP\_004088683.1 | molecular chaperone GroES | taxID used:135614 OG00292|WP\_088578238.1 | glutamine--tRNA ligase/YqeY domain fusion protein | taxID used:135614 OG02931|WP\_004090283.1 | XRE family transcriptional regulator | taxID used:135614 OG02936|WP\_004086345.1 | DNA polymerase | taxID used:135614 OG02938|WP\_004084034.1 | DUF493 domain-containing protein | taxID used:135614 OG00293|WP\_023907627.1 | aspartate--tRNA ligase | taxID used:135614 OG02946|WP\_010894520.1 | hypothetical protein | taxID used:135614 OG00294|WP\_042462939.1 | endoglucanase | taxID used:135614 OG02950|WP\_004087942.1 | hypothetical protein | taxID used:135614 OG02951|WP\_011097829.1 | Fe(2+)-trafficking protein | taxID used:135614 OG02952|WP\_046418477.1 | hypothetical protein | taxID used:135614 OG00295|WP\_031336180.1 | DUF885 family protein | taxID used:135614 OG02960|WP\_027700467.1 | hypothetical protein | taxID used:135614 OG02963|WP\_038228339.1 | hypothetical protein | taxID used:135614 OG02967|WP\_031336129.1 | hypothetical protein | taxID used:135614 OG02969|WP\_004086555.1 | HU family DNA-binding protein | taxID used:135614 OG00296|WP\_080679701.1 | phosphoenolpyruvate--protein phosphotransferase | taxID used:135614 OG02970|WP\_004085558.1 | RNA-binding protein Hfq | taxID used:135614 OG02973|WP\_004086525.1 | 30S ribosomal protein S19 | taxID used:135614 OG02976|WP\_004083966.1 | hypothetical protein | taxID used:135614 OG02977|WP\_004085172.1 | addiction module antidote protein, HigA family | taxID used:135614 OG00297|WP\_031336883.1 | outer membrane protein assembly factor | taxID used:135614 OG02984|WP\_004083631.1 | addiction module antidote protein, HigA family | taxID used:135614 OG02987|WP\_012382594.1 | hypothetical protein | taxID used:135614 OG02989|WP\_080673504.1 | hypothetical protein | taxID used:135614 OG00298|WP\_020852237.1 | acetolactate synthase 2 catalytic subunit | taxID used:135614 OG02991|WP\_038211658.1 | DUF2523 domain-containing protein | taxID used:135614 OG02997|WP\_081089894.1 | hypothetical protein | taxID used:135614 OG00299|WP\_011098351.1 | aminopeptidase | taxID used:135614 OG00029|WP\_082355539.1 | hypothetical protein | taxID used:135614 OG00002|WP\_012382737.1 | filamentous hemagglutinin N-terminal domain-containing protein | taxID used:135614 OG03001|WP\_004085493.1 | Fis family transcriptional regulator | taxID used:135614 OG03004|WP\_109160998.1 | hypothetical protein | taxID used:135614 OG03006|WP\_010894518.1 | hypothetical protein | taxID used:135614 OG03008|WP\_080507180.1 | Hpt domain-containing protein | taxID used:135614 OG00300|WP\_031337108.1 | ShlB/FhaC/HecB family hemolysin secretion/activation protein | taxID used:135614 OG03016|WP\_058569933.1 | hypothetical protein | taxID used:135614 OG03020|WP\_024749209.1 | 30S ribosomal protein S17 | taxID used:135614 OG03025|WP\_010893184.1 | exodeoxyribonuclease 7 small subunit | taxID used:135614 OG00302|WP\_046418009.1 | DNA primase | taxID used:135614 OG03033|WP\_004089222.1 | HPr family phosphocarrier protein | taxID used:135614 OG03036|WP\_027700582.1 | hypothetical protein | taxID used:135614 OG00303|WP\_020851262.1 | ABC transporter ATP-binding protein/permease | taxID used:135614 OG03042|WP\_004090451.1 | acyl carrier protein | taxID used:135614 OG00304|WP\_088578309.1 | lipid A export permease/ATP-binding protein MsbA | taxID used:135614 OG03050|WP\_010895201.1 | hypothetical protein | taxID used:135614 OG03053|WP\_004084759.1 | Trm112 family protein | taxID used:135614 OG00305|WP\_023906243.1 | hypothetical protein | taxID used:135614 OG03060|WP\_010894866.1 | 30S ribosomal protein S20 | taxID used:135614 OG03063|WP\_076613312.1 | DUF4224 domain-containing protein | taxID used:135614 OG03067|WP\_004084487.1 | cell division protein FtsL | taxID used:135614 OG03069|WP\_004089715.1 | hypothetical protein | taxID used:135614 OG00306|WP\_046417565.1 | membrane protein insertase YidC | taxID used:135614 OG03076|WP\_012382631.1 | hypothetical protein | taxID used:135614 OG03078|WP\_060872273.1 | hypothetical protein | taxID used:135614 OG03079|WP\_010894228.1 | hypothetical protein | taxID used:135614 OG00307|WP\_023906921.1 | dolichyl-phosphate-mannose--protein mannosyltransferase | taxID used:135614 OG03083|WP\_004086259.1 | 30S ribosomal protein S15 | taxID used:135614 OG03085|WP\_004087643.1 | 30S ribosomal protein S16 | taxID used:135614 OG03086|WP\_012382539.1 | DUF4224 domain-containing protein | taxID used:135614 OG00308|WP\_088577650.1 | long-chain fatty acid--CoA ligase | taxID used:135614 OG03090|WP\_080703211.1 | hypothetical protein | taxID used:135614 OG03095|WP\_060870480.1 | hypothetical protein | taxID used:135614 OG03098|WP\_023906643.1 | hypothetical protein | taxID used:135614 OG00309|WP\_046420543.1 | cation acetate symporter | taxID used:135614 OG00030|WP\_051606200.1 | hybrid sensor histidine kinase/response regulator | taxID used:135614 OG03102|WP\_053014122.1 | hypothetical protein | taxID used:135614 OG03104|WP\_060871965.1 | succinate dehydrogenase assembly factor 2 family protein | taxID used:135614 OG03105|WP\_023906214.1 | hypothetical protein | taxID used:135614 OG03109|WP\_040123140.1 | transcriptional regulator | taxID used:135614 OG00310|WP\_060871718.1 | type IV-A pilus assembly ATPase PilB | taxID used:135614 OG03115|WP\_024749085.1 | DUF4224 domain-containing protein | taxID used:135614 OG03118|WP\_004085949.1 | 50S ribosomal protein L27 | taxID used:135614 OG00311|WP\_004087620.1 | asparagine synthase B | taxID used:135614 OG03120|WP\_046420299.1 | DUF4224 domain-containing protein | taxID used:135614 OG03121|WP\_057682597.1 | DUF4065 domain-containing protein | taxID used:135614 OG03122|WP\_080673511.1 | hypothetical protein | taxID used:135614 OG00312|WP\_071869762.1 | assimilatory sulfite reductase (NADPH) hemoprotein subunit | taxID used:135614 OG03132|WP\_004086547.1 | accessory factor UbiK family protein | taxID used:135614 OG03136|WP\_038211492.1 | DUF4224 domain-containing protein | taxID used:135614 OG03137|WP\_020850879.1 | hypothetical protein | taxID used:135614 OG00313|WP\_004086424.1 | malate dehydrogenase (quinone) | taxID used:135614 OG03142|WP\_020852896.1 | hypothetical protein | taxID used:135614 OG03144|WP\_011097904.1 | DUF4224 domain-containing protein | taxID used:135614 OG03146|WP\_004090594.1 | cell division topological specificity factor MinE | taxID used:135614 OG00314|WP\_004083620.1 | GGDEF domain-containing response regulator | taxID used:135614 OG03151|WP\_020852988.1 | hypothetical protein | taxID used:135614 OG03152|WP\_076613306.1 | hypothetical protein | taxID used:135614 OG03155|WP\_046419219.1 | hypothetical protein | taxID used:135614 OG00315|WP\_021358286.1 | alkaline phosphatase | taxID used:135614 OG03162|WP\_011097950.1 | hypothetical protein | taxID used:135614 OG03163|WP\_088578132.1 | hypothetical protein | taxID used:135614 OG03164|WP\_010894487.1 | antitoxin | taxID used:135614 OG03167|WP\_023906594.1 | hypothetical protein | taxID used:135614 OG03168|WP\_046419185.1 | hypothetical protein | taxID used:135614 OG00316|WP\_011097507.1 | arginine--tRNA ligase | taxID used:135614 OG03174|WP\_004083672.1 | type B 50S ribosomal protein L31 | taxID used:135614 OG03175|WP\_031336990.1 | hypothetical protein | taxID used:135614 OG03177|WP\_038227987.1 | hypothetical protein | taxID used:135614 OG03178|WP\_004084211.1 | hypothetical protein | taxID used:135614 OG00317|WP\_058565032.1 | hypothetical protein | taxID used:135614 OG03182|WP\_010894057.1 | single-stranded DNA-binding protein | taxID used:135614 OG03185|WP\_080702466.1 | hypothetical protein | taxID used:135614 OG03186|WP\_023907036.1 | transcriptional regulator | taxID used:135614 OG00318|WP\_024748809.1 | transcription termination factor Rho | taxID used:135614 OG03190|WP\_010893451.1 | ferrous iron transport protein A | taxID used:135614 OG03192|WP\_004572808.1 | acetolactate synthase | taxID used:135614 OG03193|WP\_031336702.1 | single-stranded DNA-binding protein | taxID used:135614 OG00319|WP\_038233155.1 | proline--tRNA ligase | taxID used:135614 OG00031|WP\_004088084.1 | phospholipase | taxID used:135614 OG03200|WP\_126709116.1 | hypothetical protein | taxID used:135614 OG03202|WP\_004087778.1 | hypothetical protein | taxID used:135614 OG03203|WP\_004085758.1 | type II toxin-antitoxin system ParD family antitoxin | taxID used:135614 OG03207|WP\_010894575.1 | transcriptional regulator | taxID used:135614 OG00320|WP\_088371784.1 | LppC family lipoprotein | taxID used:135614 OG03214|WP\_046419181.1 | hypothetical protein | taxID used:135614 OG03216|WP\_012337924.1 | plasmid protein | taxID used:135614 OG03217|WP\_010894792.1 | RnfH family protein | taxID used:135614 OG00321|WP\_038229363.1 | Na+/H+ antiporter | taxID used:135614 OG03220|WP\_057683576.1 | hypothetical protein | taxID used:135614 OG03223|WP\_060872213.1 | hypothetical protein | taxID used:135614 OG03224|WP\_011097854.1 | hypothetical protein | taxID used:135614 OG03226|WP\_011097889.1 | AlpA family phage regulatory protein | taxID used:135614 OG00322|WP\_057682738.1 | phosphoethanolamine transferase | taxID used:135614 OG03230|WP\_088569729.1 | hypothetical protein | taxID used:135614 OG03232|WP\_023906434.1 | hypothetical protein | taxID used:135614 OG03233|WP\_081089819.1 | hypothetical protein | taxID used:135614 OG03234|WP\_004085761.1 | methionine repressor-like protein | taxID used:135614 OG03236|WP\_004083472.1 | MULTISPECIES: acyl carri | taxID used:135614 OG03237|WP\_004086565.1 | 50S ribosomal protein L28 | taxID used:135614 OG03238|WP\_046419252.1 | phage-related protein | taxID used:135614 OG00323|WP\_058564662.1 | PAS domain-containing sensor histidine kinase | taxID used:135614 OG03243|WP\_118853462.1 | hypothetical protein | taxID used:135614 OG03245|WP\_004087333.1 | protein SlyX | taxID used:135614 OG00324|WP\_004088642.1 | Kef family K(+) transporter | taxID used:135614 OG03250|WP\_046419974.1 | hypothetical protein | taxID used:135614 OG03254|WP\_020852703.1 | hypothetical protein | taxID used:135614 OG03255|WP\_011097871.1 | antitoxin | taxID used:135614 OG03258|WP\_038230585.1 | hypothetical protein | taxID used:135614 OG00325|WP\_027700575.1 | type II secretion system protein GspE | taxID used:135614 OG03265|WP\_088372171.1 | hypothetical protein | taxID used:135614 OG00326|WP\_023907466.1 | DNA repair protein RecN | taxID used:135614 OG03270|WP\_060870079.1 | DUF3018 domain-containing protein | taxID used:135614 OG03273|WP\_038228002.1 | hypothetical protein | taxID used:135614 OG03274|WP\_058565252.1 | hypothetical protein | taxID used:135614 OG00327|WP\_004083995.1 | electron transfer flavoprotein-ubiquinone oxidoreductase | taxID used:135614 OG03287|WP\_010894981.1 | 30S ribosomal protein S18 | taxID used:135614 OG03288|WP\_049756319.1 | hypothetical protein | taxID used:135614 OG03289|WP\_004083786.1 | magnesium transporter | taxID used:135614 OG00328|WP\_042836520.1 | hypothetical protein | taxID used:135614 OG03296|WP\_004083491.1 | MULTISPECIES: hypothetical protein [ | taxID used:135614 OG03297|WP\_004083623.1 | bacterioferritin-associated ferredoxin | taxID used:135614 OG00329|WP\_038233251.1 | M28 family peptidase | taxID used:135614 OG00032|WP\_058564412.1 | DNA polymerase III subunit alpha | taxID used:135614 OG00330|WP\_058564531.1 | polygalacturonase | taxID used:135614 OG03315|WP\_004085381.1 | TIGR02449 family protein | taxID used:135614 OG03316|WP\_012337925.1 | hypothetical protein | taxID used:135614 OG03317|WP\_046420209.1 | hypothetical protein | taxID used:135614 OG03318|WP\_004084681.1 | cold-shock protein | taxID used:135614 OG03319|WP\_004089208.1 | BolA family transcriptional regulator | taxID used:135614 OG00331|WP\_004090560.1 | CTP synthetase | taxID used:135614 OG00332|WP\_060871931.1 | hypothetical protein | taxID used:135614 OG03330|WP\_011098370.1 | hypothetical protein | taxID used:135614 OG03333|WP\_057683524.1 | hypothetical protein | taxID used:135614 OG03335|WP\_060871993.1 | hypothetical protein | taxID used:135614 OG03337|WP\_053014125.1 | hypothetical protein | taxID used:135614 OG00333|WP\_004085267.1 | GGDEF domain-containing protein | taxID used:135614 OG03340|WP\_060870419.1 | hypothetical protein | taxID used:135614 OG03345|WP\_020852693.1 | hypothetical protein | taxID used:135614 OG03346|WP\_004087472.1 | hypothetical protein | taxID used:135614 OG03349|WP\_011097601.1 | DUF1674 domain-containing protein | taxID used:135614 OG00334|WP\_046420038.1 | ubiquinone biosynthesis regulatory protein kinase UbiB | taxID used:135614 OG03351|WP\_004088694.1 | DUF465 domain-containing protein | taxID used:135614 OG03354|WP\_004083749.1 | translation initiation factor IF-1 | taxID used:135614 OG03357|WP\_004088371.1 | phage tail protein | taxID used:135614 OG00335|WP\_057683315.1 | 30S ribosomal protein S1 | taxID used:135614 OG03360|WP\_023906409.1 | hypothetical protein | taxID used:135614 OG03361|WP\_051604010.1 | hypothetical protein | taxID used:135614 OG03368|WP\_020851016.1 | AbrB/MazE/SpoVT family DNA-binding domain-containing protein | taxID used:135614 OG03369|WP\_027700173.1 | helix-turn-helix transcriptional regulator | taxID used:135614 OG00336|WP\_011098235.1 | energy-dependent translational throttle protein EttA | taxID used:135614 OG03372|WP\_004083544.1 | Sec-independent protein translocase subunit TatA | taxID used:135614 OG03375|WP\_042836479.1 | Rha family transcriptional regulator | taxID used:135614 OG00337|WP\_038228516.1 | membrane protein | taxID used:135614 OG03382|WP\_080703193.1 | DUF4224 domain-containing protein | taxID used:135614 OG03385|WP\_010893161.1 | hypothetical protein | taxID used:135614 OG03388|WP\_004083596.1 | 30S ribosomal protein S21 | taxID used:135614 OG00338|WP\_004572839.1 | site-specific DNA-methyltransferase | taxID used:135614 OG03392|WP\_010894258.1 | integrase | taxID used:135614 OG03396|WP\_004085529.1 | carbon storage regulator | taxID used:135614 OG03399|WP\_011347600.1 | MULTISPECIES: AlpA family transcriptional regula | taxID used:135614 OG00339|WP\_080679662.1 | M48 family peptidase | taxID used:135614 OG00033|WP\_023906015.1 | transcription-repair coupling factor | taxID used:135614 OG03400|WP\_081089741.1 | DUF1376 domain-containing protein | taxID used:135614 OG03404|WP\_088371434.1 | hypothetical protein | taxID used:135614 OG03408|WP\_003290186.1 | MULTISPECIES: hypothetical prot | taxID used:135614 OG00340|WP\_004087917.1 | hybrid sensor histidine kinase/response regulator | taxID used:135614 OG03419|WP\_060870463.1 | hypothetical protein | taxID used:135614 OG03429|WP\_004085832.1 | cold-shock protein | taxID used:135614 OG00343|WP\_088572609.1 | hypothetical protein | taxID used:135614 OG00344|WP\_021358252.1 | glucan biosynthesis protein D | taxID used:135614 OG03454|WP\_010892647.1 | zinc-finger domain-containing protein | taxID used:135614 OG00345|WP\_126715038.1 | polyvinylalcohol dehydrogenase | taxID used:135614 OG03461|WP\_046419249.1 | hypothetical protein | taxID used:135614 OG03464|WP\_031345749.1 | 50S ribosomal protein L29 | taxID used:135614 OG03467|WP\_004087870.1 | hypothetical protein | taxID used:135614 OG00346|WP\_088371685.1 | peptide synthase | taxID used:135614 OG03471|WP\_038232929.1 | hypothetical protein | taxID used:135614 OG03474|WP\_038230226.1 | hypothetical protein | taxID used:135614 OG00347|WP\_058564781.1 | glutamine-dependent NAD+ synthetase | taxID used:135614 OG03481|WP\_023907740.1 | Rha family transcriptional regulator | taxID used:135614 OG03482|WP\_081090404.1 | phage coat protein, partial | taxID used:135614 OG03485|WP\_004090468.1 | sulfur carrier protein ThiS | taxID used:135614 OG00348|WP\_058564659.1 | dihydrolipoyllysine-residue acetyltransferase | taxID used:135614 OG03491|WP\_004090402.1 | 50S ribosomal protein L35 | taxID used:135614 OG03498|WP\_060870442.1 | DUF4224 domain-containing protein | taxID used:135614 OG00349|WP\_004088684.1 | molecular chaperone GroEL | taxID used:135614 OG00034|WP\_057682376.1 | chromosome segregation protein SMC | taxID used:135614 OG00350|WP\_080939573.1 | hypothetical protein | taxID used:135614 OG03514|WP\_076613603.1 | single-stranded DNA-binding protein | taxID used:135614 OG00351|WP\_010895110.1 | RNA helicase | taxID used:135614 OG03524|WP\_010892929.1 | hypothetical protein | taxID used:135614 OG03526|WP\_023907191.1 | hypothetical protein | taxID used:135614 OG03529|WP\_060870346.1 | hypothetical protein | taxID used:135614 OG00352|WP\_058565169.1 | peptidase S41 | taxID used:135614 OG03530|WP\_010894283.1 | 50S ribosomal protein L32 | taxID used:135614 OG03537|WP\_081089878.1 | hypothetical protein | taxID used:135614 OG03538|WP\_081033453.1 | histidine kinase | taxID used:135614 OG00353|WP\_038230200.1 | peptidase S41 | taxID used:135614 OG03545|WP\_075584665.1 | 50S ribosomal protein L30 | taxID used:135614 OG00354|WP\_004085580.1 | peptidase S41 | taxID used:135614 OG03550|WP\_023906448.1 | hypothetical protein | taxID used:135614 OG03566|WP\_004086874.1 | hypothetical protein | taxID used:135614 OG03568|WP\_046419769.1 | hypothetical protein | taxID used:135614 OG00356|WP\_004087188.1 | peptide chain release factor 3 | taxID used:135614 OG03571|WP\_023906503.1 | hypothetical protein | taxID used:135614 OG03572|WP\_109160991.1 | hypothetical protein | taxID used:135614 OG03573|WP\_060872419.1 | hypothetical protein | taxID used:135614 OG03576|WP\_004087046.1 | hypothetical protein | taxID used:135614 OG03579|WP\_060872274.1 | hypothetical protein | taxID used:135614 OG00357|WP\_031337130.1 | YdiU family protein | taxID used:135614 OG03582|WP\_004085135.1 | hypothetical protein | taxID used:135614 OG03589|WP\_038232921.1 | hypothetical protein | taxID used:135614 OG03593|WP\_004087470.1 | hypothetical protein | taxID used:135614 OG03594|WP\_081033439.1 | hypothetical protein | taxID used:135614 OG03596|WP\_046420103.1 | hypothetical protein | taxID used:135614 OG00359|WP\_012337845.1 | peptidase S41 | taxID used:135614 OG03602|WP\_031336773.1 | hypothetical protein | taxID used:135614 OG03603|WP\_023906213.1 | hypothetical protein | taxID used:135614 OG03605|WP\_010894630.1 | hypothetical protein | taxID used:135614 OG03606|WP\_109161004.1 | DUF1631 domain-containing protein | taxID used:135614 OG03607|WP\_081089780.1 | hypothetical protein | taxID used:135614 OG03609|WP\_010892910.1 | rubredoxin | taxID used:135614 OG00360|WP\_020851064.1 | bifunctional phosphoribosylaminoimidazolecarboxamide formyltransferase/IMP cyclohydrolase PurH | taxID used:135614 OG03615|WP\_109160989.1 | hypothetical protein | taxID used:135614 OG03618|WP\_004089787.1 | DUF2065 family protein | taxID used:135614 OG00361|WP\_020851243.1 | two-component sensor histidine kinase | taxID used:135614 OG03621|WP\_060871479.1 | hypothetical protein, partial | taxID used:135614 OG03622|WP\_004086643.1 | 30S ribosomal protein THX | taxID used:135614 OG00362|WP\_004090427.1 | N-acetylmuramoyl-L-alanine amidase | taxID used:135614 OG03631|WP\_058569976.1 | hypothetical protein | taxID used:135614 OG03633|WP\_080502613.1 | hypothetical protein | taxID used:135614 OG03638|WP\_004086144.1 | hypothetical protein | taxID used:135614 OG00363|WP\_038283983.1 | murein biosynthesis integral membrane protein MurJ | taxID used:135614 OG03640|WP\_080939545.1 | heme exporter protein CcmD | taxID used:135614 OG03643|WP\_088371569.1 | hypothetical protein | taxID used:135614 OG03645|WP\_081033562.1 | hypothetical protein | taxID used:135614 OG03646|WP\_080702483.1 | hypothetical protein | taxID used:135614 OG03647|WP\_004086566.1 | 50S ribosomal protein L33 | taxID used:135614 OG00364|WP\_057683313.1 | GMP synthase (glutamine-hydrolyzing) | taxID used:135614 OG03650|WP\_012382551.1 | DUF1631 family protein | taxID used:135614 OG03652|WP\_004083785.1 | hypothetical protein | taxID used:135614 OG03654|WP\_004086061.1 | transcriptional regulator | taxID used:135614 OG00365|WP\_046420930.1 | SAM-dependent DNA methyltransferase | taxID used:135614 OG00366|WP\_012337641.1 | hypothetical protein | taxID used:135614 OG00367|WP\_058569312.1 | type I restriction-modification system subunit M | taxID used:135614 OG00368|WP\_058564940.1 | alkyl hydroperoxide reductase subunit F | taxID used:135614 OG00369|WP\_031336299.1 | EAL domain-containing protein | taxID used:135614 OG00036|WP\_010895025.1 | Rne/Rng family ribonuclease | taxID used:135614 OG00370|WP\_060870333.1 | phage portal protein | taxID used:135614 OG00371|WP\_031336803.1 | PhoPQ-regulated protein | taxID used:135614 OG00374|WP\_088372149.1 | metal-independent alpha-mannosidase | taxID used:135614 OG00375|WP\_012382573.1 | hypothetical protein | taxID used:135614 OG00376|WP\_023907644.1 | 2-isopropylmalate synthase | taxID used:135614 OG00377|WP\_004088278.1 | glycerol-3-phosphate dehydrogenase | taxID used:135614 OG00378|WP\_058569996.1 | peptidase M23, partial | taxID used:135614 OG00379|WP\_088577796.1 | alpha/beta hydrolase | taxID used:135614 OG00037|WP\_126715034.1 | exodeoxyribonuclease V subunit gamma | taxID used:135614 OG00380|WP\_071869861.1 | S10 family peptidase | taxID used:135614 OG00381|WP\_010894357.1 | peptide MFS transporter | taxID used:135614 OG00382|WP\_010893662.1 | F0F1 ATP synthase subunit alpha | taxID used:135614 OG00383|WP\_020852722.1 | hypothetical protein | taxID used:135614 OG00384|WP\_012337974.1 | lipopolysaccharide biosynthesis protein | taxID used:135614 OG00385|WP\_057682654.1 | L-aspartate oxidase | taxID used:135614 OG00386|WP\_046420290.1 | virulence factor | taxID used:135614 OG00387|WP\_027700101.1 | peptidase S1 | taxID used:135614 OG00388|WP\_058564329.1 | DUF3375 domain-containing protein | taxID used:135614 OG00389|WP\_004085668.1 | lysine--tRNA ligase | taxID used:135614 OG00038|WP\_024749033.1 | calcium-binding protein | taxID used:135614 OG00390|WP\_023908123.1 | exopolyphosphatase | taxID used:135614 OG00391|WP\_010894947.1 | hypothetical protein | taxID used:135614 OG00392|WP\_088578604.1 | glycerol kinase | taxID used:135614 OG00393|WP\_024749267.1 | fumarate hydratase | taxID used:135614 OG00394|WP\_010892710.1 | MFS transporter | taxID used:135614 OG00395|WP\_020852042.1 | hypothetical protein | taxID used:135614 OG00397|WP\_058569363.1 | tRNA (N6-isopentenyl adenosine(37)-C2)-methylthiotransferase MiaB | taxID used:135614 OG00398|WP\_080939581.1 | hypothetical protein | taxID used:135614 OG00399|WP\_010893691.1 | ATP-binding protein | taxID used:135614 OG00039|WP\_060872177.1 | membrane protein | taxID used:135614 OG00003|WP\_088569701.1 | adhesin | taxID used:135614 OG00401|WP\_088577657.1 | NADH-quinone oxidoreductase subunit M | taxID used:135614 OG00402|WP\_010892773.1 | glucose-6-phosphate isomerase | taxID used:135614 OG00403|WP\_057682801.1 | Virulence-associated E | taxID used:135614 OG00404|WP\_004572953.1 | transcription termination/antitermination protein NusA | taxID used:135614 OG00405|WP\_027700228.1 | glutamate synthase subunit beta | taxID used:135614 OG00406|WP\_046419225.1 | hypothetical protein | taxID used:135614 OG00407|WP\_058564434.1 | anthranilate synthase component I | taxID used:135614 OG00409|WP\_058564509.1 | Rne/Rng family ribonuclease | taxID used:135614 OG00040|WP\_046419726.1 | hypothetical protein | taxID used:135614 OG00410|WP\_004089062.1 | Fe-S cluster assembly protein SufB | taxID used:135614 OG00411|WP\_023906724.1 | hybrid sensor histidine kinase/response regulator | taxID used:135614 OG00412|WP\_020853093.1 | replicative DNA helicase | taxID used:135614 OG00413|WP\_020851291.1 | undecaprenyl-phosphate glucose phosphotransferase | taxID used:135614 OG00414|WP\_081364544.1 | UDP-N-acetylmuramoyl-L-alanyl-D-glutamate--2,6-diaminopimelate ligase | taxID used:135614 OG00415|WP\_004085038.1 | adenosylmethionine--8-amino-7-oxononanoate transaminase | taxID used:135614 OG00416|WP\_038229315.1 | amidophosphoribosyltransferase | taxID used:135614 OG00417|WP\_046420052.1 | ammonia channel protein | taxID used:135614 OG00419|WP\_058569579.1 | leucyl aminopeptidase | taxID used:135614 OG00041|WP\_057682882.1 | carbamoyl-phosphate synthase large subunit | taxID used:135614 OG00420|WP\_088578410.1 | bifunctional ADP-dependent NAD(P)H-hydrate dehydratase/NAD(P)H-hydrate epimerase | taxID used:135614 OG00422|WP\_024749123.1 | exodeoxyribonuclease I | taxID used:135614 OG00423|WP\_023906336.1 | amino acid permease | taxID used:135614 OG00425|WP\_046418573.1 | pyruvate kinase | taxID used:135614 OG00426|WP\_058564382.1 | NADH-quinone oxidoreductase subunit NuoN | taxID used:135614 OG00427|WP\_004089347.1 | S-adenosyl-L-homocysteine hydrolase | taxID used:135614 OG00429|WP\_046420481.1 | amino acid permease | taxID used:135614 OG00042|WP\_004088450.1 | hypothetical protein | taxID used:135614 OG00431|WP\_021358465.1 | glucose-6-phosphate dehydrogenase | taxID used:135614 OG00432|WP\_046418200.1 | IMP dehydrogenase | taxID used:135614 OG00433|WP\_051606222.1 | two-component sensor histidine kinase | taxID used:135614 OG00436|WP\_004085498.1 | membrane protein | taxID used:135614 OG00437|WP\_057682870.1 | metalloprotease TldD | taxID used:135614 OG00441|WP\_004091001.1 | 3-isopropylmalate dehydratase large subunit | taxID used:135614 OG00442|WP\_010894897.1 | ribosomal large subunit pseudouridine synthase B | taxID used:135614 OG00444|WP\_060872231.1 | type I glutamate--ammonia ligase | taxID used:135614 OG00447|WP\_060870510.1 | virulence factor | taxID used:135614 OG00448|WP\_031336960.1 | cysteine--tRNA ligase | taxID used:135614 OG00449|WP\_058564344.1 | S41 family peptidase | taxID used:135614 OG00044|WP\_004085780.1 | acriflavine resistance protein B | taxID used:135614 OG00450|WP\_031337879.1 | dihydrolipoyl dehydrogenase | taxID used:135614 OG00451|WP\_004088148.1 | uroporphyrinogen-III C-methyltransferase | taxID used:135614 OG00452|WP\_053014128.1 | hypothetical protein | taxID used:135614 OG00453|WP\_023906105.1 | UDP-N-acetylmuramate:L-alanyl-gamma-D-glutamyl-meso-diaminopimelate ligase | taxID used:135614 OG00454|WP\_020851010.1 | peptidase S1 | taxID used:135614 OG00455|WP\_088371846.1 | amino acid permease | taxID used:135614 OG00456|WP\_010894982.1 | asparagine--tRNA ligase | taxID used:135614 OG00457|WP\_020851208.1 | UDP-N-acetylmuramate--L-alanine ligase | taxID used:135614 OG00458|WP\_057682840.1 | cardiolipin synthase | taxID used:135614 OG00459|WP\_038210663.1 | replicative DNA helicase | taxID used:135614 OG00045|WP\_004086751.1 | multidrug efflux RND transporter permease subunit | taxID used:135614 OG00460|WP\_088577601.1 | alpha-L-fucosidase | taxID used:135614 OG00461|WP\_004084450.1 | glutamate--tRNA ligase | taxID used:135614 OG00462|WP\_020851308.1 | HlyD family type I secretion periplasmic adaptor subunit | taxID used:135614 OG00463|WP\_031337514.1 | mannose-1-phosphate guanylyltransferase/mannose-6-phosphate isomerase | taxID used:135614 OG00466|WP\_010894314.1 | nitrogen regulation protein NR(I) | taxID used:135614 OG00469|WP\_060872077.1 | virulence factor | taxID used:135614 OG00046|WP\_011098173.1 | TonB-dependent receptor | taxID used:135614 OG00471|WP\_038227603.1 | divalent metal cation transporter MntH | taxID used:135614 OG00472|WP\_020851182.1 | LOG family protein YgdH | taxID used:135614 OG00473|WP\_046420474.1 | FAD-binding oxidoreductase | taxID used:135614 OG00475|WP\_023908060.1 | chaperone SurA | taxID used:135614 OG00476|WP\_057683133.1 | FkbM family methyltransferase | taxID used:135614 OG00477|WP\_058569761.1 | exopolysaccharide biosynthesis protein | taxID used:135614 OG00478|WP\_010893660.1 | F0F1 ATP synthase subunit beta | taxID used:135614 OG00479|WP\_072866383.1 | DUF1073 domain-containing protein | taxID used:135614 OG00480|WP\_031338065.1 | sigma-54-dependent Fis family transcriptional regulator | taxID used:135614 OG00481|WP\_058564945.1 | class II fumarate hydratase | taxID used:135614 OG00482|WP\_004090031.1 | UDP-N-acetylmuramoyl-L-alanine--D-glutamate ligase | taxID used:135614 OG00484|WP\_020851237.1 | type II secretion system F family protein | taxID used:135614 OG00485|WP\_046420507.1 | histidine--tRNA ligase | taxID used:135614 OG00486|WP\_010894236.1 | MFS transporter | taxID used:135614 OG00487|WP\_011097510.1 | phosphomannomutase/phosphoglucomutase | taxID used:135614 OG00488|WP\_010893929.1 | glutamate--cysteine ligase | taxID used:135614 OG00489|WP\_004085376.1 | DUF1631 family protein | taxID used:135614 OG00048|WP\_031337635.1 | calcium-binding protein, partial | taxID used:135614 OG00490|WP\_038227947.1 | ribosome biogenesis GTPase Der | taxID used:135614 OG00491|WP\_020851922.1 | acetyl-CoA carboxylase biotin carboxylase subunit | taxID used:135614 OG00492|WP\_042463301.1 | P-type conjugative transfer protein TrbL | taxID used:135614 OG00493|WP\_057682418.1 | UDP-N-acetylmuramoylalanyl-D-glutamyl-2, 6-diaminopimelate--D-alanyl-D-alanine ligase | taxID used:135614 OG00495|WP\_080939583.1 | RNA polymerase factor sigma-54 | taxID used:135614 OG00496|WP\_031336811.1 | DNA repair protein RadA | taxID used:135614 OG00497|WP\_027700404.1 | sensor histidine kinase | taxID used:135614 OG00498|WP\_010893986.1 | HslU--HslV peptidase ATPase subunit | taxID used:135614 OG00499|WP\_004090028.1 | hypothetical protein | taxID used:135614 OG00049|WP\_004087462.1 | type I restriction endonuclease subunit R | taxID used:135614 OG00004|WP\_088577762.1 | bacteriocin | taxID used:135614 OG00500|WP\_010892880.1 | FAD-binding oxidoreductase | taxID used:135614 OG00501|WP\_010893157.1 | hypothetical protein | taxID used:135614 OG00502|WP\_004085324.1 | cytochrome c oxidase subunit II | taxID used:135614 OG00503|WP\_004083516.1 | CBS domain-containing protein | taxID used:135614 OG00504|WP\_031336760.1 | DUF2252 domain-containing protein | taxID used:135614 OG00505|WP\_004084964.1 | serine hydrolase | taxID used:135614 OG00506|WP\_046419690.1 | UDP-glucose/GDP-mannose dehydrogenase family protein | taxID used:135614 OG00507|WP\_010893685.1 | preprotein translocase subunit SecY | taxID used:135614 OG00508|WP\_046419044.1 | bifunctional N-acetylglucosamine-1-phosphate uridyltransferase/glucosamine-1-phosphate acetyltransferase | taxID used:135614 OG00509|WP\_027700773.1 | DUF445 domain-containing protein | taxID used:135614 OG00050|WP\_058564715.1 | efflux RND transporter permease subunit | taxID used:135614 OG00510|WP\_011097858.1 | hypothetical protein | taxID used:135614 OG00511|WP\_075584677.1 | DUF1073 domain-containing protein | taxID used:135614 OG00512|WP\_027700554.1 | adenylosuccinate lyase | taxID used:135614 OG00513|WP\_004085298.1 | NADH oxidoreductase (quinone) subunit F | taxID used:135614 OG00514|WP\_023906726.1 | replication-associated recombination protein A | taxID used:135614 OG00516|WP\_088578468.1 | phosphate regulon sensor histidine kinase PhoR | taxID used:135614 OG00518|WP\_011097494.1 | 3-deoxy-D-manno-octulosonic acid transferase | taxID used:135614 OG00519|WP\_010892943.1 | DNA recombination protein RmuC | taxID used:135614 OG00051|WP\_023906846.1 | membrane protein | taxID used:135614 OG00520|WP\_020852871.1 | signal recognition particle protein | taxID used:135614 OG00521|WP\_020852941.1 | virulence factor family protein | taxID used:135614 OG00522|WP\_046420849.1 | MATE family multidrug exporter | taxID used:135614 OG00523|WP\_012337677.1 | metalloprotease PmbA | taxID used:135614 OG00524|WP\_004086224.1 | phosphomannomutase/phosphoglucomutase | taxID used:135614 OG00525|WP\_023906937.1 | sugar transferase | taxID used:135614 OG00526|WP\_027700285.1 | aminodeoxychorismate synthase component I | taxID used:135614 OG00527|WP\_060871510.1 | hypothetical protein | taxID used:135614 OG00528|WP\_058569253.1 | aromatic hydrocarbon degradation protein | taxID used:135614 OG00529|WP\_057683102.1 | allantoinase | taxID used:135614 OG00052|WP\_088577640.1 | efflux RND transporter permease subunit | taxID used:135614 OG00530|WP\_058569391.1 | argininosuccinate lyase | taxID used:135614 OG00534|WP\_060870116.1 | zonular occludens toxin | taxID used:135614 OG00535|WP\_027700579.1 | glutamate-1-semialdehyde 2,1-aminomutase | taxID used:135614 OG00536|WP\_060870151.1 | zonular occludens toxin | taxID used:135614 OG00537|WP\_004086025.1 | N-ethylammeline chlorohydrolase | taxID used:135614 OG00538|WP\_010895004.1 | type I secretion system protein TolC | taxID used:135614 OG00539|WP\_085808105.1 | Zonular occludens toxin | taxID used:135614 OG00053|WP\_004087455.1 | type I restriction endonuclease subunit R | taxID used:135614 OG00542|WP\_004084867.1 | GTP-binding protein | taxID used:135614 OG00543|WP\_058564377.1 | NADH-quinone oxidoreductase subunit D | taxID used:135614 OG00544|WP\_085808112.1 | zonular occludens toxin | taxID used:135614 OG00545|WP\_058564484.1 | mechanosensitive ion channel family protein | taxID used:135614 OG00546|WP\_031336524.1 | Xaa-Pro aminopeptidase | taxID used:135614 OG00548|WP\_060871715.1 | sensor histidine kinase | taxID used:135614 OG00549|WP\_010895211.1 | conjugal transfer protein | taxID used:135614 OG00054|WP\_057683707.1 | AcrB/AcrD/AcrF family protein | taxID used:135614 OG00550|WP\_031336966.1 | dicarboxylate/amino acid:cation symporter | taxID used:135614 OG00551|WP\_071869510.1 | restriction endonuclease subunit S | taxID used:135614 OG00552|WP\_010892635.1 | GTPase HflX | taxID used:135614 OG00553|WP\_046418128.1 | tRNA lysidine(34) synthetase TilS | taxID used:135614 OG00554|WP\_046417781.1 | RNA helicase | taxID used:135614 OG00555|WP\_004085214.1 | RIP metalloprotease RseP | taxID used:135614 OG00556|WP\_057683582.1 | tRNA uridine-5-carboxymethylaminomethyl(34) synthesis GTPase MnmE | taxID used:135614 OG00557|WP\_011098083.1 | 23S rRNA (uracil(1939)-C(5))-methyltransferase RlmD | taxID used:135614 OG00558|WP\_038228626.1 | exodeoxyribonuclease VII large subunit | taxID used:135614 OG00559|WP\_058569562.1 | membrane protein | taxID used:135614 OG00055|WP\_004087603.1 | valine--tRNA ligase | taxID used:135614 OG00560|WP\_046418225.1 | chromosomal replication initiator protein DnaA | taxID used:135614 OG00561|WP\_024748939.1 | citrate transporter | taxID used:135614 OG00562|WP\_023906495.1 | Tol-Pal system beta propeller repeat protein TolB | taxID used:135614 OG00563|WP\_004085241.1 | acetylglutamate kinase | taxID used:135614 OG00564|WP\_038211076.1 | alkaline phosphatase family protein | taxID used:135614 OG00565|WP\_010893969.1 | phosphoglucosamine mutase | taxID used:135614 OG00566|WP\_042466566.1 | ABC transporter substrate-binding protein | taxID used:135614 OG00568|WP\_012337989.1 | bacteriocin, partial | taxID used:135614 OG00569|WP\_010893371.1 | D-amino acid dehydrogenase | taxID used:135614 OG00570|WP\_004084023.1 | HlyC/CorC family transporter | taxID used:135614 OG00571|WP\_057683263.1 | 3-phosphoshikimate 1-carboxyvinyltransferase | taxID used:135614 OG00572|WP\_010894209.1 | MFS transporter | taxID used:135614 OG00574|WP\_023906462.1 | phosphoribosylamine--glycine ligase | taxID used:135614 OG00576|WP\_085808104.1 | hypothetical protein | taxID used:135614 OG00577|WP\_027700199.1 | tryptophan--tRNA ligase | taxID used:135614 OG00578|WP\_004090446.1 | MBL fold metallo-hydrolase | taxID used:135614 OG00579|WP\_020851469.1 | DUF418 domain-containing protein | taxID used:135614 OG00057|WP\_027700384.1 | serine protease | taxID used:135614 OG00580|WP\_020850881.1 | hypothetical protein | taxID used:135614 OG00582|WP\_038200899.1 | peptidase M23 | taxID used:135614 OG00583|WP\_021358468.1 | acyl-CoA synthetase | taxID used:135614 OG00584|WP\_126715050.1 | filamentous hemagglutinin N-terminal domain-containing protein, partial | taxID used:135614 OG00585|WP\_020851473.1 | dicarboxylate/amino acid:cation symporter | taxID used:135614 OG00586|WP\_020852306.1 | cupin domain-containing protein | taxID used:135614 OG00587|WP\_010894035.1 | citrate synthase | taxID used:135614 OG00588|WP\_004086549.1 | nucleotide sugar dehydrogenase | taxID used:135614 OG00058|WP\_058564652.1 | serine protease | taxID used:135614 OG00590|WP\_057682345.1 | glutamyl-tRNA reductase | taxID used:135614 OG00592|WP\_021358704.1 | hypothetical protein | taxID used:135614 OG00593|WP\_004083580.1 | adenylosuccinate synthase | taxID used:135614 OG00595|WP\_051372664.1 | alpha/beta hydrolase | taxID used:135614 OG00596|WP\_058569342.1 | dicarboxylate/amino acid:cation symporter | taxID used:135614 OG00598|WP\_004083457.1 | serine--tRNA ligase | taxID used:135614 OG00599|WP\_004086551.1 | trigger factor | taxID used:135614 OG00059|WP\_040123317.1 | peptidase M23 | taxID used:135614 OG00005|WP\_038210541.1 | calcium-binding protein | taxID used:135614 OG00600|WP\_010894812.1 | polysaccharide biosynthesis protein GumE | taxID used:135614 OG00601|WP\_020851831.1 | nitrate ABC transporter ATP-binding protein | taxID used:135614 OG00602|WP\_027700705.1 | bifunctional tetrahydrofolate synthase/dihydrofolate synthase | taxID used:135614 OG00603|WP\_046418703.1 | cytochrome bc complex cytochrome b subunit | taxID used:135614 OG00605|WP\_023906922.1 | ribosomal RNA small subunit methyltransferase B | taxID used:135614 OG00606|WP\_004088628.1 | alpha/beta hydrolase | taxID used:135614 OG00607|WP\_058564442.1 | threonine synthase | taxID used:135614 OG00608|WP\_046419799.1 | hypothetical protein | taxID used:135614 OG00609|WP\_004089920.1 | alpha/beta hydrolase, partial | taxID used:135614 OG00060|WP\_038211001.1 | glycine dehydrogenase (aminomethyl-transferring) | taxID used:135614 OG00610|WP\_038210213.1 | histidinol dehydrogenase | taxID used:135614 OG00611|WP\_020850931.1 | glucose/galactose MFS transporter | taxID used:135614 OG00612|WP\_020851700.1 | glucose/galactose MFS transporter | taxID used:135614 OG00613|WP\_038284169.1 | phage tail protein | taxID used:135614 OG00614|WP\_010894994.1 | flavodoxin-dependent (E)-4-hydroxy-3-methylbut-2-enyl-diphosphate synthase | taxID used:135614 OG00616|WP\_038232954.1 | phage tail protein | taxID used:135614 OG00617|WP\_057683365.1 | phosphopyruvate hydratase | taxID used:135614 OG00618|WP\_038228891.1 | ATP-dependent Clp protease ATP-binding subunit ClpX | taxID used:135614 OG00619|WP\_020850937.1 | Fe-S cluster assembly protein SufD | taxID used:135614 OG00061|WP\_071869720.1 | TonB-dependent receptor | taxID used:135614 OG00620|WP\_038211284.1 | putative lipid II flippase FtsW | taxID used:135614 OG00621|WP\_020851306.1 | pyridoxal phosphate-dependent aminotransferase | taxID used:135614 OG00623|WP\_004083770.1 | UDP-N-acetylglucosamine 1-carboxyvinyltransferase | taxID used:135614 OG00624|WP\_046419397.1 | multifunctional CCA tRNA nucleotidyl transferase/2'3'-cyclic phosphodiesterase/2'nucleotidase/phosphatase | taxID used:135614 OG00625|WP\_071869913.1 | glutamate-5-semialdehyde dehydrogenase | taxID used:135614 OG00626|WP\_031337004.1 | serine hydroxymethyltransferase | taxID used:135614 OG00627|WP\_004084972.1 | AGE family epimerase/isomerase | taxID used:135614 OG00629|WP\_038283864.1 | SufS family cysteine desulfurase | taxID used:135614 OG00062|WP\_058569525.1 | excinuclease ABC subunit A | taxID used:135614 OG00630|WP\_004086282.1 | polynucleotide adenylyltransferase PcnB | taxID used:135614 OG00631|WP\_011097661.1 | HlyD family efflux transporter periplasmic adaptor subunit | taxID used:135614 OG00632|WP\_004086146.1 | septal ring lytic transglycosylase RlpA family protein | taxID used:135614 OG00633|WP\_058565044.1 | tyrosine--tRNA ligase | taxID used:135614 OG00634|WP\_021358303.1 | 6-phosphofructokinase | taxID used:135614 OG00635|WP\_023908066.1 | hypothetical protein | taxID used:135614 OG00063|WP\_058564615.1 | insulinase family protein | taxID used:135614 OG00642|WP\_057683712.1 | integrase | taxID used:135614 OG00643|WP\_004083566.1 | pilus assembly protein PilW | taxID used:135614 OG00644|WP\_010893810.1 | threonine/serine exporter family protein | taxID used:135614 OG00646|WP\_076613264.1 | hypothetical protein | taxID used:135614 OG00647|WP\_004085192.1 | ABC transporter permease | taxID used:135614 OG00649|WP\_012382717.1 | 23S rRNA (adenine(2503)-C(2))-methyltransferase RlmN | taxID used:135614 OG00064|WP\_088569744.1 | serine protease | taxID used:135614 OG00651|WP\_004083471.1 | beta-ketoacyl-[acyl-carrier-protein] synthase II | taxID used:135614 OG00652|WP\_088578506.1 | hypothetical protein | taxID used:135614 OG00653|WP\_004084469.1 | cell division protein FtsA | taxID used:135614 OG00654|WP\_058569384.1 | MFS transporter | taxID used:135614 OG00655|WP\_010894654.1 | phosphoglycerate dehydrogenase | taxID used:135614 OG00657|WP\_046419213.1 | hypothetical protein | taxID used:135614 OG00658|WP\_027700586.1 | hypothetical protein | taxID used:135614 OG00659|WP\_031336297.1 | outer membrane protein assembly factor BamB | taxID used:135614 OG00065|WP\_020852507.1 | isoleucine--tRNA ligase | taxID used:135614 OG00660|WP\_046420691.1 | efflux RND transporter periplasmic adaptor subunit | taxID used:135614 OG00661|WP\_038228585.1 | sensor histidine kinase | taxID used:135614 OG00662|WP\_004083967.1 | integrase | taxID used:135614 OG00663|WP\_038211657.1 | hypothetical protein | taxID used:135614 OG00664|WP\_004083761.1 | acetylornithine transaminase | taxID used:135614 OG00665|WP\_038227899.1 | cytochrome P450 | taxID used:135614 OG00666|WP\_057682310.1 | restriction endonuclease subunit S | taxID used:135614 OG00667|WP\_058569584.1 | bifunctional phosphopantothenoylcysteine decarboxylase/phosphopantothenate--cysteine ligase CoaBC | taxID used:135614 OG00668|WP\_046417619.1 | NO-inducible flavohemoprotein | taxID used:135614 OG00066|WP\_046419622.1 | 2-oxoglutarate dehydrogenase E1 component | taxID used:135614 OG00670|WP\_060870120.1 | hypothetical protein | taxID used:135614 OG00671|WP\_011097568.1 | porin | taxID used:135614 OG00672|WP\_031336812.1 | tryptophan synthase subunit beta | taxID used:135614 OG00673|WP\_004572970.1 | aminopeptidase P family protein | taxID used:135614 OG00674|WP\_080679706.1 | integrase | taxID used:135614 OG00675|WP\_004090511.1 | cell division protein FtsZ | taxID used:135614 OG00676|WP\_010894211.1 | PQQ-dependent sugar dehydrogenase | taxID used:135614 OG00677|WP\_088569743.1 | MFS transporter | taxID used:135614 OG00679|WP\_010892922.1 | S-adenosylmethionine synthase | taxID used:135614 OG00067|WP\_004087263.1 | VWA domain-containing protein | taxID used:135614 OG00680|WP\_046420202.1 | O-antigen polymerase | taxID used:135614 OG00682|WP\_011098220.1 | cytochrome P450 | taxID used:135614 OG00684|WP\_011097910.1 | enoyl-[acyl-carrier-protein] reductase FabV | taxID used:135614 OG00686|WP\_020851546.1 | 2-methylaconitate cis-trans isomerase PrpF | taxID used:135614 OG00687|WP\_024749268.1 | hypothetical protein | taxID used:135614 OG00688|WP\_004085243.1 | argininosuccinate synthase | taxID used:135614 OG00689|WP\_046419234.1 | replication initiation factor | taxID used:135614 OG00068|WP\_010894568.1 | VWA domain-containing protein | taxID used:135614 OG00690|WP\_023906270.1 | UDP-glucuronate--glycolipid 2-beta-glucuronosyltransferase | taxID used:135614 OG00691|WP\_088578379.1 | O-succinylhomoserine (thiol)-lyase | taxID used:135614 OG00692|WP\_010894678.1 | D-alanyl-D-alanine carboxypeptidase | taxID used:135614 OG00693|WP\_038228799.1 | nicotinate phosphoribosyltransferase | taxID used:135614 OG00694|WP\_088577586.1 | aspartate/tyrosine/aromatic aminotransferase | taxID used:135614 OG00695|WP\_010894134.1 | beta-ketoacyl-[acyl-carrier-protein] synthase family protein | taxID used:135614 OG00697|WP\_020852201.1 | TraB/GumN family protein | taxID used:135614 OG00698|WP\_027700666.1 | DUF2213 domain-containing protein | taxID used:135614 OG00699|WP\_020851735.1 | 2-octaprenyl-6-methoxyphenyl hydroxylase | taxID used:135614 OG00006|WP\_071869490.1 | calcium-binding protein | taxID used:135614 OG00700|WP\_020851976.1 | 8-amino-7-oxononanoate synthase | taxID used:135614 OG00702|WP\_058569775.1 | GTP cyclohydrolase II RibA | taxID used:135614 OG00704|WP\_060871827.1 | hypothetical protein | taxID used:135614 OG00705|WP\_004088002.1 | MFS transporter | taxID used:135614 OG00706|WP\_038229685.1 | cell division protein ZapE | taxID used:135614 OG00707|WP\_038210951.1 | type II secretion system F family protein | taxID used:135614 OG00708|WP\_080679700.1 | cation:proton antiporter | taxID used:135614 OG00709|WP\_046419351.1 | polyketide cyclase | taxID used:135614 OG00070|WP\_004083942.1 | VWA domain-containing protein | taxID used:135614 OG00710|WP\_041572637.1 | hypothetical protein | taxID used:135614 OG00711|WP\_031336363.1 | lipopolysaccharide assembly protein LapB | taxID used:135614 OG00712|WP\_004084684.1 | elongation factor Tu | taxID used:135614 OG00713|WP\_004083737.1 | class I SAM-dependent methyltransferase | taxID used:135614 OG00715|WP\_081370386.1 | replication initiation factor | taxID used:135614 OG00716|WP\_020851944.1 | tetratricopeptide repeat protein | taxID used:135614 OG00717|WP\_031337462.1 | patatin | taxID used:135614 OG00718|WP\_004085455.1 | chemotaxis protein CheB | taxID used:135614 OG00719|WP\_046420832.1 | 5-(carboxyamino)imidazole ribonucleotide synthase | taxID used:135614 OG00071|WP\_058569647.1 | TonB-dependent receptor | taxID used:135614 OG00720|WP\_004091288.1 | phage replication protein | taxID used:135614 OG00721|WP\_011097701.1 | DNA cytosine methyltransferase | taxID used:135614 OG00722|WP\_010893494.1 | porin | taxID used:135614 OG00723|WP\_031336977.1 | DUF2213 domain-containing protein | taxID used:135614 OG00724|WP\_004084431.1 | Ubiquinone biosynthesis hydroxylase UbiH/UbiF/VisC/COQ6 | taxID used:135614 OG00725|WP\_042463557.1 | ImmA/IrrE family metallo-endopeptidase | taxID used:135614 OG00729|WP\_004083615.1 | class I SAM-dependent rRNA methyltransferase | taxID used:135614 OG00072|WP\_012382448.1 | autotransporter domain-containing protein | taxID used:135614 OG00730|WP\_041581125.1 | filamentous phage Cf1c related protein | taxID used:135614 OG00731|WP\_004091217.1 | MexE family multidrug efflux RND transporter periplasmic adaptor subunit | taxID used:135614 OG00732|WP\_010894266.1 | heme biosynthesis protein HemY | taxID used:135614 OG00733|WP\_010894376.1 | signal recognition particle-docking protein FtsY | taxID used:135614 OG00734|WP\_010894210.1 | MBL fold metallo-hydrolase | taxID used:135614 OG00735|WP\_004089326.1 | 1-deoxy-D-xylulose-5-phosphate reductoisomerase | taxID used:135614 OG00736|WP\_004085873.1 | ABC transporter permease | taxID used:135614 OG00738|WP\_061278094.1 | phage tail sheath family protein | taxID used:135614 OG00739|WP\_010893863.1 | heme A synthase | taxID used:135614 OG00073|WP\_071869546.1 | DNA polymerase I | taxID used:135614 OG00741|WP\_004090622.1 | 3-dehydroquinate synthase | taxID used:135614 OG00742|WP\_088577634.1 | tRNA guanosine(34) transglycosylase Tgt | taxID used:135614 OG00743|WP\_020852152.1 | YggW family oxidoreductase | taxID used:135614 OG00744|WP\_027699985.1 | glycosyltransferase family 1 protein | taxID used:135614 OG00745|WP\_010894048.1 | dihydrolipoyllysine-residue succinyltransferase | taxID used:135614 OG00748|WP\_027699992.1 | GNAT family N-acetyltransferase | taxID used:135614 OG00749|WP\_011097736.1 | tRNA 2-thiouridine(34) synthase MnmA | taxID used:135614 OG00074|WP\_010893327.1 | protein translocase subunit SecA | taxID used:135614 OG00751|WP\_109161035.1 | hypothetical protein | taxID used:135614 OG00754|WP\_010894179.1 | addiction module antidote protein, HigA family | taxID used:135614 OG00755|WP\_057682878.1 | cation tolerance protein CutA | taxID used:135614 OG00756|WP\_004089775.1 | molybdopterin biosynthesis protein MoeB | taxID used:135614 OG00757|WP\_088578350.1 | LysM peptidoglycan-binding domain-containing protein | taxID used:135614 OG00759|WP\_088578627.1 | hypothetical protein | taxID used:135614 OG00075|WP\_011097554.1 | aconitate hydratase AcnA | taxID used:135614 OG00760|WP\_104993207.1 | lipid-A-disaccharide synthase | taxID used:135614 OG00761|WP\_010894810.1 | glycosyl transferase family 1 | taxID used:135614 OG00762|WP\_088371305.1 | hypothetical protein | taxID used:135614 OG00763|WP\_081033494.1 | DUF2066 domain-containing protein | taxID used:135614 OG00764|WP\_010893115.1 | AI-2E family transporter | taxID used:135614 OG00765|WP\_004087889.1 | glutamate 5-kinase | taxID used:135614 OG00766|WP\_004083731.1 | N-acetylglucosamine-6-phosphate deacetylase | taxID used:135614 OG00768|WP\_088578148.1 | type IV pili twitching motility protein PilT | taxID used:135614 OG00769|WP\_004088476.1 | pyridoxal phosphate-dependent aminotransferase | taxID used:135614 OG00076|WP\_057683342.1 | pyruvate dehydrogenase (acetyl-transferring), homodimeric type | taxID used:135614 OG00770|WP\_031337074.1 | phosphoglycerate kinase | taxID used:135614 OG00771|WP\_004090371.1 | succinyl-CoA ligase subunit beta | taxID used:135614 OG00772|WP\_038229707.1 | anhydro-N-acetylmuramic acid kinase | taxID used:135614 OG00773|WP\_023907390.1 | site-specific DNA-methyltransferase, partial | taxID used:135614 OG00774|WP\_038229914.1 | DNA-protecting protein DprA | taxID used:135614 OG00775|WP\_004083681.1 | general secretion pathway protein GspL | taxID used:135614 OG00776|WP\_010893775.1 | lipoyl synthase | taxID used:135614 OG00777|WP\_046417955.1 | OmpA family protein | taxID used:135614 OG00778|WP\_080679666.1 | triacylglycerol lipase | taxID used:135614 OG00779|WP\_010894665.1 | bifunctional histidinol-phosphatase/imidazoleglycerol-phosphate dehydratase | taxID used:135614 OG00077|WP\_004088171.1 | beta-mannosidase | taxID used:135614 OG00780|WP\_011097870.1 | hypothetical protein | taxID used:135614 OG00781|WP\_088371931.1 | DUF1073 domain-containing protein | taxID used:135614 OG00782|WP\_057682978.1 | succinyl-diaminopimelate desuccinylase | taxID used:135614 OG00783|WP\_021358595.1 | rod shape-determining protein RodA | taxID used:135614 OG00784|WP\_010892648.1 | glycosyl transferase group 1 | taxID used:135614 OG00785|WP\_057683266.1 | energy transducer TonB | taxID used:135614 OG00786|WP\_031336924.1 | carbamoyl-phosphate synthase small subunit | taxID used:135614 OG00787|WP\_004091077.1 | chorismate mutase | taxID used:135614 OG00788|WP\_011098075.1 | Amino acid-binding ACT:Prephenate dehydrogenase | taxID used:135614 OG00078|WP\_012382579.1 | serine protease | taxID used:135614 OG00790|WP\_038228106.1 | homoserine O-acetyltransferase | taxID used:135614 OG00791|WP\_046418021.1 | FtsH protease activity modulator HflK | taxID used:135614 OG00793|WP\_038229930.1 | 3,4-dihydroxy-2-butanone-4-phosphate synthase | taxID used:135614 OG00794|WP\_004089604.1 | DUF4105 domain-containing protein | taxID used:135614 OG00796|WP\_071869544.1 | peptide chain release factor 2 | taxID used:135614 OG00798|WP\_088371226.1 | PLP-dependent cysteine synthase family protein | taxID used:135614 OG00799|WP\_071869578.1 | glycosyltransferase family 1 protein | taxID used:135614 OG00079|WP\_058564560.1 | fimbrial biogenesis outer membrane usher protein | taxID used:135614 OG00007|WP\_057682684.1 | response regulator | taxID used:135614 OG00800|WP\_004086145.1 | lytic murein transglycosylase B | taxID used:135614 OG00803|WP\_023906755.1 | chorismate synthase | taxID used:135614 OG00804|WP\_057683270.1 | molecular chaperone DnaJ | taxID used:135614 OG00806|WP\_004091489.1 | Plasmid encoded RepA protein | taxID used:135614 OG00807|WP\_088577723.1 | aminoglycoside phosphotransferase | taxID used:135614 OG00808|WP\_010892552.1 | DNA replication and repair protein RecF | taxID used:135614 OG00809|WP\_046417730.1 | LPS export ABC transporter permease LptG | taxID used:135614 OG00080|WP\_010895193.1 | protein containing caspase domain | taxID used:135614 OG00810|WP\_060870354.1 | Replication initiation factor | taxID used:135614 OG00812|WP\_012382440.1 | NADH-quinone oxidoreductase subunit NuoH | taxID used:135614 OG00813|WP\_010892726.1 | glycine cleavage system aminomethyltransferase GcvT | taxID used:135614 OG00814|WP\_038231772.1 | agamatine deiminase | taxID used:135614 OG00815|WP\_004083972.1 | ABC transporter permease | taxID used:135614 OG00816|WP\_012382673.1 | acyltransferase 3 | taxID used:135614 OG00817|WP\_010894590.1 | phosphate ABC transporter substrate-binding protein PstS | taxID used:135614 OG00081|WP\_004086138.1 | leucine--tRNA ligase | taxID used:135614 OG00820|WP\_011097703.1 | DUF262 domain-containing protein | taxID used:135614 OG00821|WP\_010892575.1 | 3-deoxy-7-phosphoheptulonate synthase | taxID used:135614 OG00822|WP\_038228624.1 | ribonuclease D | taxID used:135614 OG00823|WP\_057683074.1 | AI-2E family transporter | taxID used:135614 OG00825|WP\_012382577.1 | hypothetical protein | taxID used:135614 OG00826|WP\_080729492.1 | toprim domain-containing protein | taxID used:135614 OG00827|WP\_004088182.1 | alanine racemase | taxID used:135614 OG00828|WP\_060870296.1 | DUF2075 domain-containing protein | taxID used:135614 OG00829|WP\_011098070.1 | phosphoserine transaminase | taxID used:135614 OG00082|WP\_004084616.1 | DNA gyrase subunit A | taxID used:135614 OG00830|WP\_080673512.1 | XRE family transcriptional regulator | taxID used:135614 OG00833|WP\_004085494.1 | branched-chain amino acid aminotransferase | taxID used:135614 OG00834|WP\_004083630.1 | site-specific integrase | taxID used:135614 OG00835|WP\_088371543.1 | zonular occludens toxin | taxID used:135614 OG00836|WP\_050812598.1 | efflux RND transporter periplasmic adaptor subunit | taxID used:135614 OG00838|WP\_004085242.1 | peptidase M20 | taxID used:135614 OG00839|WP\_010892551.1 | DNA polymerase III subunit beta | taxID used:135614 OG00083|WP\_088578386.1 | beta-glucosidase | taxID used:135614 OG00840|WP\_027700229.1 | cellulase | taxID used:135614 OG00843|WP\_011097599.1 | sn-glycerol-3-phosphate ABC transporter ATP-binding protein UgpC | taxID used:135614 OG00844|WP\_012337946.1 | histidinol-phosphate aminotransferase | taxID used:135614 OG00845|WP\_058569486.1 | undecaprenyldiphospho-muramoylpentapeptide beta-N-acetylglucosaminyltransferase | taxID used:135614 OG00848|WP\_010893316.1 | phospho-N-acetylmuramoyl-pentapeptide-transferase | taxID used:135614 OG00849|WP\_023908051.1 | glycosyl hydrolase | taxID used:135614 OG00084|WP\_027700368.1 | alanine--tRNA ligase | taxID used:135614 OG00851|WP\_004089089.1 | LacI family transcriptional regulator | taxID used:135614 OG00852|WP\_012337564.1 | LPS export ABC transporter permease LptF | taxID used:135614 OG00853|WP\_004087401.1 | peptide chain release factor 1 | taxID used:135614 OG00854|WP\_010894537.1 | efflux RND transporter periplasmic adaptor subunit | taxID used:135614 OG00855|WP\_004089675.1 | bifunctional diaminohydroxyphosphoribosylaminopyrimidine deaminase/5-amino-6-(5-phosphoribosylamino)uracil reductase RibD | taxID used:135614 OG00858|WP\_071869748.1 | YafY family transcriptional regulator | taxID used:135614 OG00859|WP\_058570025.1 | DNA primase | taxID used:135614 OG00085|WP\_010892776.1 | translation initiation factor IF-2 | taxID used:135614 OG00861|WP\_046420808.1 | redox-regulated ATPase YchF | taxID used:135614 OG00862|WP\_023907303.1 | DNA primase | taxID used:135614 OG00863|WP\_023907540.1 | DNA primase | taxID used:135614 OG00865|WP\_004086116.1 | lipopolysaccharide heptosyltransferase family protein | taxID used:135614 OG00866|WP\_004084564.1 | 23S rRNA (cytidine(2498)-2'-O)-methyltransferase RlmM | taxID used:135614 OG00867|WP\_057683309.1 | GTPase ObgE | taxID used:135614 OG00869|WP\_031336560.1 | A/G-specific adenine glycosylase | taxID used:135614 OG00086|WP\_058564342.1 | TonB-dependent receptor | taxID used:135614 OG00870|WP\_010894206.1 | sorbosone dehydrogenase family protein | taxID used:135614 OG00871|WP\_088371287.1 | NAD(P)-dependent alcohol dehydrogenase | taxID used:135614 OG00872|WP\_031345936.1 | DNA topoisomerase | taxID used:135614 OG00873|WP\_010893837.1 | uroporphyrinogen decarboxylase | taxID used:135614 OG00875|WP\_020851248.1 | S-methyl-5-thioribose-1-phosphate isomerase | taxID used:135614 OG00876|WP\_004085791.1 | 3-isopropylmalate dehydrogenase | taxID used:135614 OG00877|WP\_104996237.1 | intercellular spreading VacJ lipoprotein | taxID used:135614 OG00878|WP\_010894364.1 | cell envelope integrity protein TolA | taxID used:135614 OG00879|WP\_010893694.1 | lipase secretion chaperone | taxID used:135614 OG00087|WP\_088578262.1 | Fe/S-dependent 2-methylisocitrate dehydratase AcnD | taxID used:135614 OG00880|WP\_060872081.1 | phage integrase | taxID used:135614 OG00881|WP\_010894217.1 | NAD(P)-dependent alcohol dehydrogenase | taxID used:135614 OG00882|WP\_004090975.1 | NAD(P)-dependent alcohol dehydrogenase | taxID used:135614 OG00884|WP\_088578335.1 | alpha/beta hydrolase | taxID used:135614 OG00885|WP\_004084645.1 | UDP-N-acetylenolpyruvoylglucosamine reductase | taxID used:135614 OG00886|WP\_038227265.1 | dTDP-glucose 4,6-dehydratase | taxID used:135614 OG00888|WP\_010894198.1 | NADP-alcohol dehydrogenase | taxID used:135614 OG00889|WP\_004090192.1 | ribonucleotide-diphosphate reductase subunit beta | taxID used:135614 OG00088|WP\_088578321.1 | glycerol-3-phosphate 1-O-acyltransferase PlsB | taxID used:135614 OG00892|WP\_027700304.1 | phosphoribosylformylglycinamidine cyclo-ligase | taxID used:135614 OG00893|WP\_023906427.1 | hypothetical protein | taxID used:135614 OG00894|WP\_049767181.1 | endolytic transglycosylase MltG | taxID used:135614 OG00895|WP\_004085486.1 | CDP-glycerol glycerophosphotransferase family protein | taxID used:135614 OG00898|WP\_010894214.1 | alpha/beta hydrolase | taxID used:135614 OG00899|WP\_010892665.1 | right-handed parallel beta-helix repeat-containing protein | taxID used:135614 OG00008|WP\_058564967.1 | NAD-glutamate dehydrogenase | taxID used:135614 OG00900|WP\_004083513.1 | GDP-mannose 4,6-dehydratase | taxID used:135614 OG00901|WP\_010895227.1 | hypothetical protein | taxID used:135614 OG00902|WP\_004085848.1 | heat-inducible transcriptional repressor HrcA | taxID used:135614 OG00903|WP\_004090333.1 | quinone-dependent dihydroorotate dehydrogenase | taxID used:135614 OG00904|WP\_080715115.1 | PhoH family protein | taxID used:135614 OG00905|WP\_080654484.1 | two-component sensor histidine kinase | taxID used:135614 OG00906|WP\_010894576.1 | hypothetical protein | taxID used:135614 OG00907|WP\_010895208.1 | conjugal transfer protein | taxID used:135614 OG00908|WP\_020852236.1 | threonine dehydratase | taxID used:135614 OG00090|WP\_060871960.1 | bifunctional aspartate kinase/diaminopimelate decarboxylase | taxID used:135614 OG00910|WP\_042463646.1 | S-adenosylmethionine:tRNA ribosyltransferase-isomerase | taxID used:135614 OG00911|WP\_010893851.1 | sulfate ABC transporter ATP-binding protein | taxID used:135614 OG00912|WP\_038211538.1 | biotin synthase BioB | taxID used:135614 OG00913|WP\_004084948.1 | type IV pili twitching motility protein PilT | taxID used:135614 OG00914|WP\_004085284.1 | ABC transporter substrate-binding protein | taxID used:135614 OG00915|WP\_004089911.1 | type IV pilus assembly protein PilM | taxID used:135614 OG00916|WP\_060871962.1 | tetraacyldisaccharide 4'-kinase | taxID used:135614 OG00918|WP\_058569213.1 | aspartate-semialdehyde dehydrogenase | taxID used:135614 OG00919|WP\_004086557.1 | DUF475 domain-containing protein | taxID used:135614 OG00091|WP\_088577652.1 | bifunctional aconitate hydratase 2/2-methylisocitrate dehydratase | taxID used:135614 OG00920|WP\_004083595.1 | tRNA (adenosine(37)-N6)-threonylcarbamoyltransferase complex transferase subunit TsaD | taxID used:135614 OG00921|WP\_023906619.1 | aldo/keto reductase | taxID used:135614 OG00922|WP\_057683107.1 | N-acetylornithine carbamoyltransferase | taxID used:135614 OG00924|WP\_038228019.1 | ribosome small subunit-dependent GTPase A | taxID used:135614 OG00925|WP\_071869658.1 | rod shape-determining protein | taxID used:135614 OG00926|WP\_046417698.1 | DNA recombination/repair protein RecA | taxID used:135614 OG00928|WP\_023906078.1 | anthranilate phosphoribosyltransferase | taxID used:135614 OG00929|WP\_020852809.1 | tRNA dihydrouridine(20/20a) synthase DusA | taxID used:135614 OG00092|WP\_046419728.1 | bifunctional lysylphosphatidylglycerol flippase/synthetase MprF | taxID used:135614 OG00930|WP\_004084959.1 | sensor histidine kinase | taxID used:135614 OG00931|WP\_011097624.1 | phage integrase | taxID used:135614 OG00932|WP\_011097905.1 | integrase | taxID used:135614 OG00933|WP\_027700744.1 | glycerol-3-phosphate dehydrogenase | taxID used:135614 OG00934|WP\_058569358.1 | magnesium and cobalt transport protein CorA | taxID used:135614 OG00935|WP\_020851363.1 | DNA polymerase III subunit delta | taxID used:135614 OG00936|WP\_011097966.1 | integrase | taxID used:135614 OG00937|WP\_012337868.1 | phage integrase | taxID used:135614 OG00938|WP\_046419629.1 | integrase | taxID used:135614 OG00940|WP\_004086678.1 | chromosome partitioning protein ParB | taxID used:135614 OG00941|WP\_010894216.1 | alpha/beta hydrolase | taxID used:135614 OG00942|WP\_060872183.1 | integrase | taxID used:135614 OG00943|WP\_011097774.1 | integrase | taxID used:135614 OG00944|WP\_010894952.1 | integrase | taxID used:135614 OG00945|WP\_012382597.1 | hypothetical protein | taxID used:135614 OG00946|WP\_027700281.1 | EF-P beta-lysylation protein EpmB | taxID used:135614 OG00948|WP\_080939595.1 | sulfate ABC transporter substrate-binding protein | taxID used:135614 OG00949|WP\_004085423.1 | Holliday junction branch migration DNA helicase RuvB | taxID used:135614 OG00094|WP\_088578586.1 | DNA mismatch repair protein MutS | taxID used:135614 OG00950|WP\_004084974.1 | carbohydrate kinase | taxID used:135614 OG00952|WP\_088577873.1 | SIS domain-containing protein | taxID used:135614 OG00953|WP\_004083996.1 | SMP-30/gluconolactonase/LRE family protein | taxID used:135614 OG00955|WP\_012382741.1 | homoserine acetyltransferase | taxID used:135614 OG00957|WP\_057683427.1 | glucokinase | taxID used:135614 OG00958|WP\_080715094.1 | terminase | taxID used:135614 OG00959|WP\_004085216.1 | UDP-3-O-(3-hydroxymyristoyl)glucosamine N-acyltransferase | taxID used:135614 OG00095|WP\_038210757.1 | glycosyltransferase family 1 protein | taxID used:135614 OG00960|WP\_004085471.1 | 3-oxoacyl-ACP synthase III | taxID used:135614 OG00961|WP\_004085533.1 | nitronate monooxygenase | taxID used:135614 OG00963|WP\_031336169.1 | glycosyltransferase | taxID used:135614 OG00964|WP\_046419300.1 | alpha/beta hydrolase | taxID used:135614 OG00965|WP\_058569918.1 | porphobilinogen synthase | taxID used:135614 OG00966|WP\_004084873.1 | ketol-acid reductoisomerase | taxID used:135614 OG00967|WP\_023906581.1 | 3-beta hydroxysteroid dehydrogenase/isomerase | taxID used:135614 OG00968|WP\_058569478.1 | fructose-bisphosphate aldolase class I | taxID used:135614 OG00970|WP\_020851191.1 | type I glyceraldehyde-3-phosphate dehydrogenase | taxID used:135614 OG00971|WP\_011097577.1 | N-acetyl-gamma-glutamyl-phosphate reductase | taxID used:135614 OG00972|WP\_038229855.1 | glucokinase | taxID used:135614 OG00973|WP\_038228746.1 | phenylalanine--tRNA ligase subunit alpha | taxID used:135614 OG00974|WP\_010893935.1 | peptidase | taxID used:135614 OG00975|WP\_004084859.1 | nucleoside-diphosphate sugar epimerase | taxID used:135614 OG00977|WP\_038200914.1 | DUF2272 domain-containing protein | taxID used:135614 OG00978|WP\_031337050.1 | methionine ABC transporter ATP-binding protein | taxID used:135614 OG00979|WP\_004083792.1 | trans-hexaprenyltranstransferase | taxID used:135614 OG00980|WP\_046419683.1 | ABC transporter permease | taxID used:135614 OG00981|WP\_046420659.1 | beta-N-acetylhexosaminidase | taxID used:135614 OG00982|WP\_023906989.1 | pteridine-dependent deoxygenase | taxID used:135614 OG00983|WP\_042462818.1 | FAD:protein FMN transferase | taxID used:135614 OG00984|WP\_004087529.1 | NAD-dependent isocitrate dehydrogenase | taxID used:135614 OG00985|WP\_060872028.1 | lipopolysaccharide biosynthesis protein | taxID used:135614 OG00987|WP\_058569912.1 | DUF2184 domain-containing protein | taxID used:135614 OG00988|WP\_020852888.1 | SMC-Scp complex subunit ScpB | taxID used:135614 OG00989|WP\_057683626.1 | phage integrase | taxID used:135614 OG00990|WP\_027700003.1 | KpsF/GutQ family sugar-phosphate isomerase | taxID used:135614 OG00991|WP\_088572610.1 | DUF2184 domain-containing protein, partial | taxID used:135614 OG00992|WP\_023907690.1 | DUF2184 domain-containing protein | taxID used:135614 OG00993|WP\_031336424.1 | nucleoside-diphosphate sugar epimerase | taxID used:135614 OG00994|WP\_057682775.1 | oxidoreductase | taxID used:135614 OG00996|WP\_069106985.1 | copper resistance protein CopB | taxID used:135614 OG00997|WP\_071869668.1 | LLM class flavin-dependent oxidoreductase | taxID used:135614 OG00998|WP\_038228905.1 | malate dehydrogenase | taxID used:135614 OG00999|WP\_031337572.1 | ferrochelatase | taxID used:135614 OG00009|WP\_038274152.1 | alpha-2-macroglobulin family protein | taxID used:135614 OG01449|WP\_046417487.1 | conjugal transfer protein TraL | taxID used:135614 OG02068|WP\_020852695.1 | pilin | taxID used:135614 OG02124|WP\_010895235.1 | conjugal transfer protein TrbH | taxID used:135614 OG02157|WP\_060872114.1 | endolysin | taxID used:135614 OG02334|WP\_004085693.1 | hypothetical protein | taxID used:135614 OG02515|WP\_016024085.1 | hypothetical protein | taxID used:135614 OG02643|WP\_046419268.1 | phage coat protein | taxID used:135614 OG02681|WP\_011097873.1 | hypothetical protein | taxID used:135614 OG02787|WP\_075584660.1 | hypothetical protein | taxID used:135614 OG02993|WP\_010894342.1 | DUF2523 domain-containing protein | taxID used:135614 OG03173|WP\_038211688.1 | hypothetical protein | taxID used:135614 OG03198|WP\_004084254.1 | hypothetical protein | taxID used:135614 OG03206|WP\_012337848.1 | hypothetical protein | taxID used:135614 OG03222|WP\_010894056.1 | DUF4224 domain-containing protein | taxID used:135614 OG03278|WP\_060872192.1 | hypothetical protein | taxID used:135614 OG03436|WP\_010894516.1 | hypothetical protein | taxID used:135614 OG03586|WP\_004085135.1 | hypothetical protein | taxID used:135614 OG03592|WP\_020851634.1 | hypothetical protein | taxID used:135614 OG03636|WP\_080513453.1 | hypothetical protein | taxID used:135614 OG00494|WP\_011098374.1 | hypothetical protein | taxID used:135614 OG00589|WP\_060871510.1 | hypothetical protein | taxID used:135614 OG00727|WP\_024749110.1 | Replication initiation factor | taxID used:135614 OG00864|WP\_060871821.1 | DNA primase | taxID used:135614 OG00089|WP\_038232753.1 | autotransporter domain-containing protein | taxID used:135614 
order

5
135625
order

712
5
family

416916
1
genus

732 


OG01730|WP\_020852402.1 | hypothetical protein | taxID used:732 
species
1

genus
3
1960084

1


OG03228|WP\_076613304.1 | lysozyme | taxID used:1908260 
species
1908260 

species


OG02999|WP\_010895180.1 | hypothetical protein | taxID used:1906745 OG03639|WP\_076613311.1 | lysozyme | taxID used:1906745 
2
1906745 

genus
1
75984

1432056 


OG03658|WP\_088572583.1 | hypothetical protein | taxID used:1432056 
species
1

class
2008785
2

order
119069
2

family
206349
2

203470
2
genus

876478 
species


OG01562|WP\_081089805.1 | transposase | taxID used:876478 OG00681|WP\_042462688.1 | transposase | taxID used:876478 
2

4
1807140
class

order
225057
4

4
225058
family

genus
4
119977

160808 
1


OG01762|WP\_010895246.1 | hypothetical protein | taxID used:160808 
species

930 
3
species


OG02860|WP\_038229226.1 | BrnT family toxin | taxID used:930 OG03659|WP\_081046888.1 | translation repressor RelB | taxID used:930 OG00541|WP\_080507215.1 | GNAT family N-acetyltransferase | taxID used:930 

144
28211
class

204457
10
order

family
10
41297

1
165697
genus


OG03423|WP\_004087156.1 | hypothetical protein | taxID used:1736474 
species
1
1736474 

13687
2
genus


OG02034|WP\_060870169.1 | peptidase | taxID used:1768786 
species
1
1768786 

1


OG02765|WP\_004083642.1 | hypothetical protein | taxID used:473781 
species
473781 

genus
165696
6

1
species


OG02028|WP\_060870392.1 | hypothetical protein | taxID used:48935 
48935 

species


OG02762|WP\_038231154.1 | hypothetical protein | taxID used:1420591 
1
1420591 

1850347 
4
species


OG02671|WP\_010894063.1 | hypothetical protein | taxID used:1850347 OG02747|WP\_023906664.1 | hypothetical protein | taxID used:1850347 OG03380|WP\_060872193.1 | hypothetical protein, partial | taxID used:1850347 OG03387|WP\_060872140.1 | hypothetical protein, partial | taxID used:1850347 

genus
1
165695

1884365 
1


OG01522|WP\_080654445.1 | sulfite exporter TauE/SafE family protein | taxID used:1884365 
species

3
204455
order

family
3
31989

265
1
genus

1


OG01504|WP\_082355558.1 | RepB family plasmid replication initiator protein | taxID used:34004 
species
34004 

genus
1158296
1

871651 


OG00470|WP\_080939628.1 | restriction endonuclease subunit S | taxID used:871651 
species
1

genus
1
875170

1411902 
1
species


OG02637|WP\_004088650.1 | phage baseplate protein | taxID used:1411902 

356
128
order

1
772
family

1
773
genus

species


OG00683|WP\_038230535.1 | hypothetical protein | taxID used:545617 
1
545617 

family
41294
2

709797 


OG03627|WP\_038230947.1 | hypothetical protein | taxID used:709797 
species
1

genus
374
1

1
species


OG02805|WP\_023907895.1 | superoxide dismutase | taxID used:1882760 
1882760 

119045
9
family

9


OG01397|WP\_058565017.1 | hypothetical protein | taxID used:407 OG01759|WP\_076613287.1 | acyl-ACP--UDP-N-acetylglucosamine O-acyltransferase | taxID used:407 
genus
407
2

1


OG03065|WP\_023906433.1 | hypothetical protein | taxID used:1730094 
species
1730094 

269660 
6
species


OG01302|WP\_042463195.1 | UDP-3-O-(3-hydroxymyristoyl)glucosamine N-acyltransferase | taxID used:269660 OG01345|WP\_071869619.1 | hypothetical protein | taxID used:269660 OG01775|WP\_004083960.1 | UDP-3-O-(3-hydroxymyristoyl)glucosamine N-acyltransferase | taxID used:269660 OG00591|WP\_051606239.1 | hypothetical protein | taxID used:269660 OG00856|WP\_081089786.1 | UDP-3-O-(3-hydroxymyristoyl)glucosamine N-acyltransferase | taxID used:269660 OG00857|WP\_060871450.1 | hypothetical protein | taxID used:269660 

genus
1145345
1

665467 
1
species


OG02390|WP\_004090276.1 | hypothetical protein | taxID used:665467 

69277
2
family

2


OG01535|WP\_004086766.1 | hypothetical protein | taxID used:68287 OG01536|WP\_011097776.1 | hypothetical protein | taxID used:68287 
genus
68287 

82115
113
family

genus
357
3


OG02839|WP\_080502622.1 | hypothetical protein | taxID used:373 OG03073|WP\_023906425.1 | type II toxin-antitoxin system antitoxin, RelB/DinJ family | taxID used:373 OG03149|WP\_010894534.1 | type II toxin-antitoxin system RelB/DinJ family antitoxin | taxID used:373 
species
3
373 

2
genus


OG01299|WP\_058564977.1 | hypothetical protein | taxID used:28105 OG01948|WP\_080703209.1 | hypothetical protein | taxID used:28105 
28105 

genus
1
34019

1


OG02032|WP\_004089511.1 | hypothetical protein | taxID used:34021 
species
34021 

genus
2
106591

106592 
species


OG03307|WP\_075584656.1 | terminase | taxID used:106592 
1

species


OG02983|WP\_038230657.1 | antitoxin | taxID used:1057002 
1
1057002 

genus


OG01010|WP\_088578582.1 | quinolinate synthase NadA | taxID used:379 
OG01010|WP\_088578582.1 | quinolinate synthase NadA | taxID used:379 OG01971|WP\_011098371.1 | hypothetical protein | taxID used:379 
15
12
379 

species


OG01989|WP\_004083561.1 | hypothetical protein | taxID used:78527 OG02433|WP\_004083560.1 | DNA methyltransferase | taxID used:78527 
2
78527 

1


OG03229|WP\_004083558.1 | hypothetical protein | taxID used:1144310 
species
1144310 


OG02253|WP\_004083559.1 | hypothetical protein | taxID used:1229204 
species
1
1229204 

204441
2
order

family
1
41295

191
1
genus

34010 
1


OG03030|WP\_014607458.1 | hypothetical protein | taxID used:34010 
species

1
433
family

genus
522
1

1043206 
species


OG03089|WP\_013087943.1 | antitoxin | taxID used:1043206 
1

phylum
1239
7

2
186801
class

order
2
186802

186803
1
family

33042
1
genus

1


OG02278|WP\_075584666.1 | XRE family transcriptional regulator | taxID used:33043 
species
33043 

186807
1
family

79206
1
genus

1


OG02560|WP\_080507203.1 | hypothetical protein | taxID used:1969834 
species
1969834 

class
91061
4

order
2
1385

186817
1
family

1386 
1
genus


OG00726|WP\_042463298.1 | hypothetical protein | taxID used:1386 

1
186824
family

332100
1
genus

1


OG02910|WP\_010894491.1 | hypothetical protein | taxID used:201973 
species
201973 

186826
2
order

1
33958
family

1
1578
genus

1598 
1


OG03172|WP\_072866333.1 | hypothetical protein | taxID used:1598 
species

family
1
1300

genus
1357
1

1


OG03533|WP\_080507226.1 | methyltransferase | taxID used:1363 
species
1363 

class
1737404
1

1
1737405
order

1
1570339
family

1
162289
genus

1472765 
1


OG00746|WP\_080939629.1 | restriction endonuclease subunit S | taxID used:1472765 
species

phylum
6
201174

6
1760
class

order
85006
2

1
85017
family

genus
1
157920

1710 
1


OG00818|WP\_010894203.1 | alkene reductase | taxID used:1710 
species

family
1
85023

genus
1573
1

1
species


OG00947|WP\_046419227.1 | peptidase | taxID used:28447 
28447 

1
85014
order

1
85034
family

genus
58113
1

380244 
species


OG02745|WP\_080507181.1 | hypothetical protein | taxID used:380244 
1

order
85011
2

family
2
2062

1883
2
genus

1
species


OG03655|WP\_004084060.1 | site-specific DNA-methyltransferase | taxID used:68194 
68194 


OG00474|WP\_031337748.1 | hypothetical protein | taxID used:1463833 
species
1
1463833 

order
85010
1

family
2070
1

genus
1
39845

species


OG02954|WP\_038210462.1 | hypothetical protein | taxID used:103730 
1
103730 

1855372 
1


OG00418|WP\_057683745.1 | SAM-dependent methyltransferase | taxID used:1855372 
species

phylum
57723
1

204432
1
class

order
204433
1

204434
1
family

genus
940557
1

940614 


OG00951|WP\_020852638.1 | Peptidase S24, S26A and S26B | taxID used:940614 
species
1

phylum
74201
2

class
1
203494

order
1
48461

1
203557
family

genus
1
2735

species


OG02152|WP\_046419782.1 | hypothetical protein | taxID used:1882831 
1
1882831 

478741 
species


OG02585|WP\_010892859.1 | MerR family DNA-binding transcriptional regulator | taxID used:478741 
1

phylum
1
1297

188787
1
class

68933
1
order

family
1
188786

65551
1
genus

277 


OG00628|WP\_046420928.1 | restriction endonuclease subunit S | taxID used:277 
species
1

1117
10
phylum

7
1301283
subclass

order
6
1118

family
1890449
1

genus
1
1125


OG03013|WP\_038274576.1 | hypothetical protein | taxID used:1126 
species
1
1126 

family
1890450
5

genus
1121
5

543815 
5
species


OG02104|WP\_020852349.1 | RTX toxin | taxID used:543815 OG02109|WP\_004087881.1 | RTX toxin | taxID used:543815 OG02201|WP\_023907937.1 | hypothetical protein | taxID used:543815 OG02305|WP\_088371878.1 | RTX toxin | taxID used:543815 OG03355|WP\_088578327.1 | RTX toxin | taxID used:543815 

order
1
1150

1892252
1
family

54304
1
genus

1729650 
1


OG02092|WP\_004089816.1 | site-specific DNA-methyltransferase | taxID used:1729650 
species

order
3
1890424

family
1890426
3

2
1129
genus

2
species


OG02113|WP\_060872331.1 | RTX toxin, partial | taxID used:169670 OG02197|WP\_023906854.1 | RTX toxin | taxID used:169670 
169670 

genus
1
13034

292566 
species


OG03324|WP\_080502625.1 | hypothetical protein | taxID used:292566 
1

203691
3
phylum

class
3
203692

family
170
3

genus
3
171

28182 
species


OG01870|WP\_046419172.1 | hypothetical protein | taxID used:28182 OG01879|WP\_060872166.1 | hypothetical protein | taxID used:28182 OG02549|WP\_088578438.1 | hypothetical protein | taxID used:28182 
3

976
7
phylum

3
117743
class

order
200644
3

3
49546
family

1
290174
genus

1


OG03430|WP\_080502609.1 | hypothetical protein | taxID used:980584 
species
980584 

genus
286104
1


OG03672|WP\_088578514.1 | hypothetical protein | taxID used:262004 
species
1
262004 

1016
1
genus

1945657 
1


OG02932|WP\_082357548.1 | hypothetical protein | taxID used:1945657 
species

class
117747
2

order
2
200666

family
2
84566

genus
2
423349

2


OG01958|WP\_031337700.1 | peptidase | taxID used:1234841 OG02700|WP\_046419203.1 | hypothetical protein | taxID used:1234841 
species
1234841 

class
1
1937959

order
1936988
1

family
1
1937960

genus
1
70994

1
species


OG02834|WP\_076613191.1 | hypothetical protein | taxID used:70998 
70998 

1
1100069
order

1
563843
family

genus
29548
1

633813 


OG01540|WP\_004084202.1 | hypothetical protein | taxID used:633813 
species
1
