# Supplementary material for: Orthology-Based Estimate of the Contribution of Horizontal Gene Transfer from Distantly Related Bacteria to the Intraspecific Diversity and Differentiation of Xylella fastidiosa
Source: Pathogens. 2021 Jan 7;10(1):46. doi: 10.3390/pathogens10010046 (PMC7828034; doi:10.3390/pathogens10010046)
Supplement: Supplementary file 1 [file pathogens-10-00046-s001.zip › pathogens-1031631-supplementary-final/pathogens-1031631-Table S3_enzyme.docx]

**Table S3.** List of the annotation of degradative and synthetic enzyme encoding genes in the pan-genome of Xy. fastidiosa that have no homologues in the Xanthomonadales. The column on the right reports the detected number of groups of ortholog genes found analyzing 45 genomes of Xy. fastidiosa.

| **Annotation** | **#OGs** |
| --- | --- |
| Peptidases of various families | 16 |
| Lytic enzymes similar to lysozyme | 5 |
| Acyltransferases | 2 |
| Alpha/beta hydrolases | 3 |
| Acetyltransferases | 8 |
| Reductases | 4 |
| Arginine deaminase | 1 |
| Carboxymuconolactone decarboxylase | 1 |
| Glutamate-1-semialdehyde 2,1-aminomutase | 1 |
| SMP-30/gluconolactonase/LRE family protein | 1 |
| Glutamine amidotransferase | 1 |
| Glycosyltransferase | 1 |
| Anthranilate synthases | 2 |
| Colicin synthesis proteins | 4 |
| Quinolinate synthase | 1 |
